# Metallaphotoredox deuteroalkylation utilizing thianthrenium salts

**DOI:** 10.1038/s41467-024-48590-w

**Published:** 2024-06-13

**Authors:** Mengjie Jiao, Jie Zhang, Minyan Wang, Hongjian Lu, Zhuangzhi Shi

**Affiliations:** State Key Laboratory of Coordination Chemistry, Chemistry and Biomedicine Innovation Center (ChemBIC), School of Chemistry and Chemical Engineering, Nanjing University, Nanjing, Jiangsu 210093 China

**Keywords:** Synthetic chemistry methodology, Photocatalysis, Homogeneous catalysis

## Abstract

Deuterium labeling compounds play a crucial role in organic and pharmaceutical chemistry. The synthesis of such compounds typically involves deuterated building blocks, allowing for the incorporation of deuterium atoms and functional groups into a target molecule in a single step. Unfortunately, the limited availability of synthetic approaches to deuterated synthons has impeded progress in this field. Here, we present an approach utilizing alkyl-substituted thianthrenium salts that efficiently and selectively introduce deuterium at the α position of alkyl chains through a pH-dependent HIE process, using D_2_O as the deuterium source. The resulting α-deuterated alkyl thianthrenium salts, which bear two deuterium atoms, exhibit excellent selectivity and deuterium incorporation in electrophilic substitution reactions. Through in situ formation of isotopically labelled alkyl halides, these thianthrenium salts demonstrate excellent compatibility in a series of metallaphotoredox cross-electrophile coupling with (hetero)aryl, alkenyl, alkyl bromides, and other alkyl thianthrenium salts. Our technique allows for a wide range of substrates, high deuterium incorporation, and precise control over the site of deuterium insertion within a molecule such as the benzyl position, allylic position, or any alkyl chain in between, as well as neighboring heteroatoms. This makes it invaluable for synthesizing various deuterium-labeled compounds, especially those with pharmaceutical significance.

## Abstract (web summary)

Deuterium labeling compounds play a crucial role in organic and pharmaceutical chemistry but the limited availability of synthetic approaches to deuterated synthons has impeded progress in this field. Here, the authors present an approach utilizing alkyl-substituted aryl thianthrenium salts that efficiently and selectively introduce deuterium at the α position of alkyl chains through a pH-dependent hydrogen isotope exchange process, using D2O as the deuterium source.

## Introduction

**Fig. 1:**
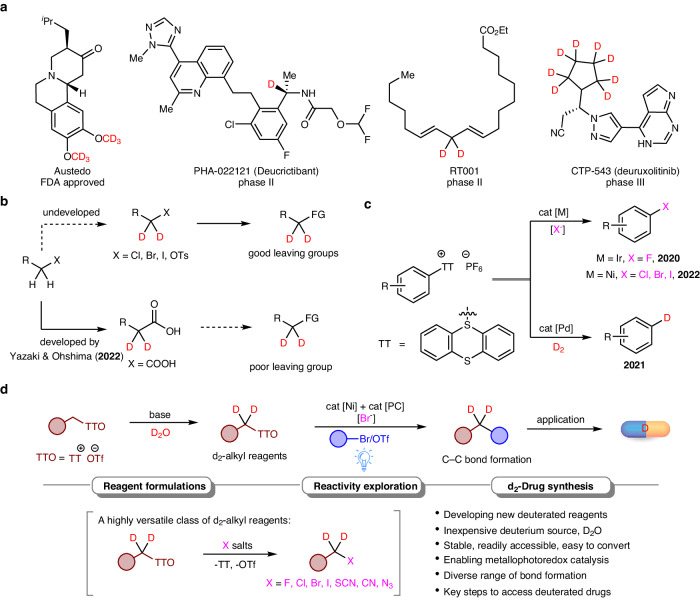
Background and new strategy to access deuterium-containing molecules. **a** Deuterium-containing drugs. **b** Deuteration of alkyl (pseudo)halides and carboxylic acids. **c** Reduction of aryl TT salts to access isotopically labelled arenes. **d** Metallaphotoredox-catalyzed cross-electrophile coupling of d2-labeled TT salts (This work).

Deuterium is a naturally occurring stable isotope of hydrogen containing only an additional neutron. Due to the higher stability of C−D bonds than C−H bonds, drug molecules labeled with deuterium are expected to have longer half-lives, increased efficacy and reduced side effects, making them possibly more desirable than the parent compounds^1,2^. Recently, deuterium-containing molecules such as Austedo (deutetrabenzine- d6)^3^ have been approved for use as new drugs, and other drugs like PHA-022121, RT001, and CTP-543 are currently being tested (Fig. 1a)^4^. Furthermore, deuterium-labeled compounds are valuable for studying kinetic isotope effects during mechanistic investigations and are widely used as internal standards in quantitative mass spectrometry^5^. Therefore, exploring the chemical space of deuterium-labelled molecules can open new doors to functional-molecule innovation and discovery in several scientific fields. Since that most top-selling commercial drugs contain at least one alkyl moiety, a general synthetic method for achieving isotopically labelled functionalized alkanes would be highly valuable^6–8^. The most popular and efficient synthetic methods, such as hydrogen isotope exchange (HIE)^9–12^, reductive deuteration^13^, and dehalogenative deuteration^14,15^, have been developed to introduce deuterium atoms directly to target molecules. However, synthesizing functionalized alkanes with precise control over deuterated sites, high efficiency and high deuterium incorporation remains challenging.

The use of deuterated building blocks in synthetic approaches can lead to the efficient production of deuterium labeled compounds. This technique enables the introduction of both deuterium atoms and functional groups in a single step, resulting in the construction of a target molecule with easily controllable deuterated sites and a high level of deuterium incorporation. Although this method has been utilized in the synthesis of deuterium-containing drugs^16–20^, its progress has been hindered by the limited availability of readily accessible deuterated reagents. Alkyl halides (I, Br, and Cl) and pseudohalides (OTs, OTf) are widely applied and abundant electrophilic alkylation reagents within the realm of organic synthesis. Due to the low pKa of α C–H bonds and the reactivity of C–X bonds, HIE of alkyl (pseudo)halides to prepare isotopically labeled alkyl electrophiles can be synthetically challenging (Fig. 1b, up). Typically, indirect methods to access deuterated alkyl electrophiles are employed. The state-of-the-art progress involves the utilization of a ternary catalytic system, enabling the efficient α-deuteration of carboxylic acids (Fig. 1b, down)^21^. However, alkyl carboxylic acids, which possess low electrophilicity, necessitate the formation of redox activated esters to enable decarboxylative substitution. Therefore, chemists need to choose a functional group that possesses a good balance, capable of promoting HIE process while also exhibiting excellent leaving ability.

Sulfonium salts are highly versatile compounds characterized by the presence of positively charged sulfur ions with three organic substituents^22–30^. They serve as immensely useful aryl and alkyl electrophiles in a wide range of chemical reactions. Among these compounds, aryl thianthrenium (TT) salts are particularly renowned for their exceptional versatility as reactive intermediates^31–38^. Recently, Ritter and coworkers have successfully utilized these compounds for halogenation (F^39^, Cl, Br, and I^40^ and hydrogen isotope labeling^41^ by breaking carbon–sulfur bonds with the assistance of transition metals catalysis (Fig. 1c). Furthermore, alkyl TT salts have been investigated by our group^42,43^ and other researchers^44,45^, resulting in a noteworthy expansion of the chemical landscape for alkyl (pseudo)halides.

Here, we introduce a highly adaptable platform utilizing TT salts for the convenient generation of deuterium-labeled alkyl electrophiles. Through a simple pH-dependent hydrogen isotope exchange (HIE) process, a range of d2-labeled alkyl TT salts can be easily synthesized in a modular manner. These compounds possess distinctive benefits in contrast to aryl TT salts, as they can readily engage in electrophilic substitution reactions with a diverse array of inorganic salts, enabling the formation of alkyl halides, cyanides, and azides without the need for catalysts. In the presence of a nickel catalyst and a photocatalyst^46–49^, these salts can also undergo cross-electrophile couplings without the need for preformed organometallic reagents^50–53^. Such groundbreaking approach involves the formation of d2-labeled alkyl halides in situ, enabling the creation of carbon–carbon bonds with a broad range of aryl, heteroaryl, vinyl, and alkyl halides. Consequently, the precise control over the incorporation of the d2-methylene motif at specific positions in an organic molecule, such as the benzyl or allylic position, as well as within the alkyl chains or neighboring heteroatoms, has revolutionized the synthesis of isotopically labeled compounds.

## Results

### Reaction design

**Fig. 2:**
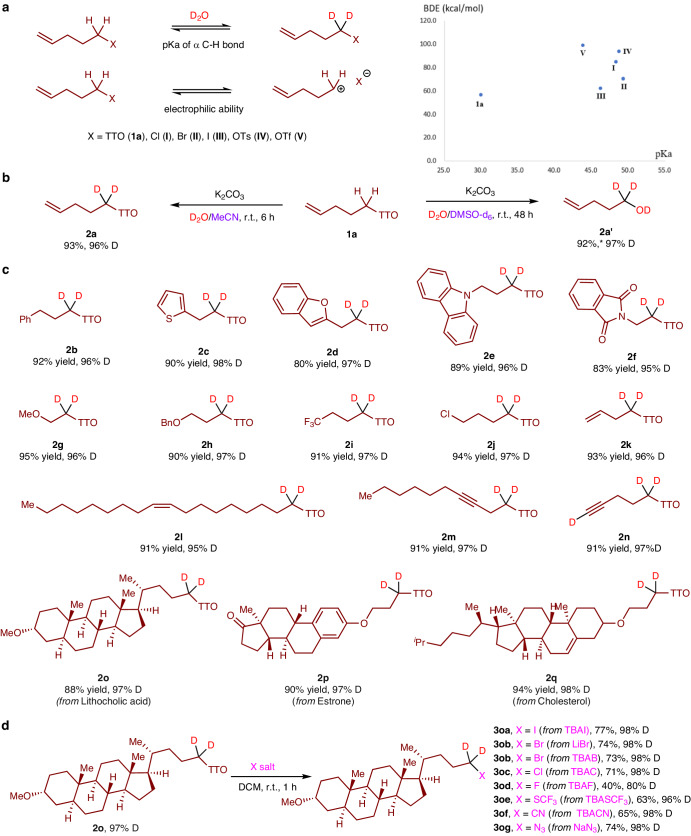
Exploring feasibility and challenges in constructing deuterated alkyl TT salts *via* pH-dependent HIE reactions. **a** Absolute pKa values of α C–H bond in water and the corresponding bond dissociation energy (BDE) of the C–X bond using the theory of M062X/6-311+g(2d,p), SMD solvation calculation (for details, see Supplementary Date). **b** HIE of TT salt 1a with D_2_O to 2a and substitution reaction to alcohol 2a’. **c** Facile construction of a library of d2-alkyl TT salts. d, Reaction of TT salt 2o with various inorganic salts for the production of isotopically labelled compounds. Deuterium incorporation was determined by ^1^H NMR spectroscopy and/or HRMS. *The yield determined by ^1^H NMR spectrum with CH_2_Br_2_ as a standard.

The unique reactivity of alkyl TT salts can be attributed to the positive charge located at the sulfur ring, which also contributes to the acidity of the α-hydrogen of TT salts. Computational studies have investigated the characteristics of an olefin-containing alkyl TT salt **1a**, representing a typical exampleg (see Supplementary Table 2 and 3). These studies have revealed that the pKa value of the α-hydrogen in **1a**, as well as the bond dissociation energy (BDE), are comparatively lower than those of conventional alkyl (pseudo)halides, including Cl (I), Br (II), I (III), OTs (IV), and OTf (VI) (Fig. 2a). These findings suggest that TT salts offer reactivity advantages for pH-dependent HIE of alkyl groups in equilibrium with D_2_O, as well as serving as attractive electrophiles. Based on these theoretical findings, we investigated the HIE reaction conditions using TT salt **1a** as a model substrate (Fig. 2b). It was determined that the HIE reaction of **1a** is most effective when conducted in MeCN solvent at room temperature (r.t.) for 6 h, using D_2_O as a readily available source of deuterium and 2.0 equivalents of K_2_CO_3_ as a base. The desired d2-TT salt **2a** was obtained in 93% yield and 96% deuterium incorporation, while the formation of alcohol byproduct **2a’** was efficiently suppressed. Interestingly, we also discovered a switchable process that promotes the generation of alcohol **2a’** by simply changing the solvent to DMSO. As shown in Fig. 2c, we conducted further investigations to explore the reaction’s applicability. Remarkably, under the simple reaction conditions employed, we achieved highly efficient H/D exchange of alkyl TT salts encompassing diverse functional groups, including aryl (**2b**), heteroaryl (**2c–e**), phthalimid (**2f**), ether (**2g–h**), and trifluoroethyl (**2i**). Notably, the chlorine leaving group was effectively preserved in the resulting product **2j**, showcasing its potential for subsequent nucleophilic substitution reactions. Moreover, we examined alkene-substituted TT salts (**2k–l**) and an internally alkyne-substituted TT salt **2m** in detail. In these cases, the selective deuteration predominantly occurred at α position of the TT group. Notably, for the substrate **2n**, featuring a terminal alkyne, exchange took place not only at the position adjacent to the TT moiety but also at the acetylenic C–H bond. Furthermore, we successfully synthesized d2-alkyl compounds (**2o–q**) with impressive efficiency from alkyl TT salts (**1o**–**q**) derived from natural sources such as lithocholic acid, estrone, and cholesterol. Due to their excellent leaving ability, these TT salts have been found to exhibit high reactivity in halogenation reactions, leading to the formation of isotopically labeled alkyl halides (Fig. 2d). For instance, considering the complex molecule **2o** as an example, the reaction readily undergoes iodination (**3oa**), bromination (**3ob**), chlorination (**3oc**), and even fluorination (**3od**) with the related halide salts, resulting in good yields and deuterium incorporation. Moreover, other transformations such as trifluoromethylthiolation (**3oe**), cyanation (**3of**), and azidation (**3og**) can be also accomplished adeptly utilizing the related inorganic salts.

**Fig. 3:**
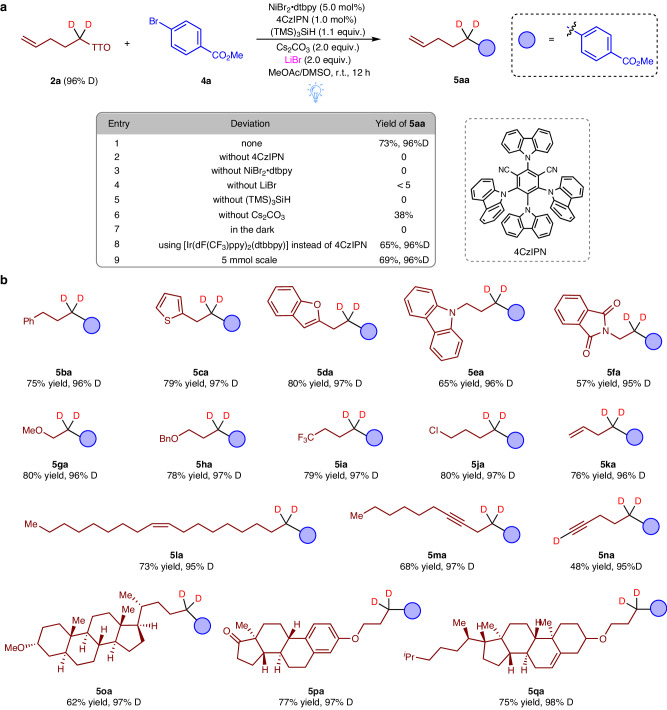
Reaction condition optimization and the substrate scope of d2-alkyl TT salts in metallaphotoredox deuteroalkylation. **a** Optimizations of the reaction of aryl bromide 4a using TT salt 2a. **b** The scope of d2-TT salts coupled with aryl bromide 4a. Standard reaction conditions: aryl bromide 1c (0.2 mmol), TT salt (0.4 mmol), 4CzIPN (1.0 mol%), NiBr_2_•dtbpy (5.0 mol%), TMS_3_SiH (0.22 mmol), Cs_2_CO_3_ (0.4 mmol), LiBr (0.4 mmol), MeOAc (1.6 mL)/DMSO (0.4 mL), Blue LEDs, r.t., 24 h, isolated yields, deuterium incorporation was determined by ^1^H NMR spectroscopy and/or HRMS.

Based on the above results, we then focused on the cross-electrophile coupling of TT salt **2a** (96% D) with aryl bromide **4a**. A crucial step in this process involved the in situ formation of isotopically labelled alkyl halides (Fig. 3a). We conducted a systematic screening of reaction conditions and successfully obtained the desired coupling product **5aa** with a remarkable 73% isolated yield and excellent deuterium incorporation (96% D). To achieve these results, we employed a catalytic system comprising a mixture of NiBr_2_· dtbpy (5.0 mol%) and 4CzIPN (1.0 mol%) as the photocatalyst. The reductant used was (TMS)_3_SiH, and we utilized 2.0 equivalents of Cs_2_CO_3_ as the base along with 2.0 equivalents of LiBr as a key additive. The reaction was carried out in a MeOAc/DMSO solvent mixture and exposed to blue LED light (440 nm) irradiation at room temperature for 12 h (entry 1). Control experiments were performed to assess the individual contributions of each component in the catalytic system. The absence of the photocatalyst (entry 2), nickel complex (entry 3), LiBr (entry 4), TMS_3_SiH (entry 5), Cs_2_CO_3_ (entry 6), or visible light (entry 7) resulted in poor outcomes, confirming the vital role played by each component. Notably, when we substituted the 4CzIPN photocatalyst with an iridium counterpart, product **5aa** was still obtained, albeit with a slightly lower yield of 65% (entry 8). Ultimately, this sequential reaction can be scaled up to the gram level with a significant decrease in yield (entry 9). Furthermore, the resulting TT reagent shows nearly perfect recovery, almost reaching quantitative retrieval. Having optimized the reaction conditions, we conducted a comprehensive examination of the substrate scope for the cross-electrophile coupling reaction. The results are summarized in Fig. 3b. To evaluate the reactivity of different d2-alkyl TT salts, we selected methyl 4-bromobenzoate (**4a**) as the substrate. Remarkably, the reaction conditions exhibited excellent tolerance towards a wide range of functional groups. Various groups, including phenyl (**5ba**), thiophene (**5ca**), benzofuran (**5da**), carbazole (**5ea**), phthalimide (**5fa**), methoxy (**5ga**), benzyloxy (**5ha**), trifluoromethyl (**5ia**), and chloride (**5ja**), were easily accommodated. Furthermore, d2-alkyl TT salts carrying carbon-carbon double bonds (**5ka,** **5la**) and triple bonds (**5ma,** **5na**) could also be efficiently converted into the desired coupling products. Notably, when complex d2-alkyl TT salts derived from natural compounds were employed, the corresponding deuterated products (**5oa–qa**) were obtained without any significant impact on C–C bond formation and deuterium incorporation. These results underscore the practicality and versatility of this method.

**Fig. 4:**
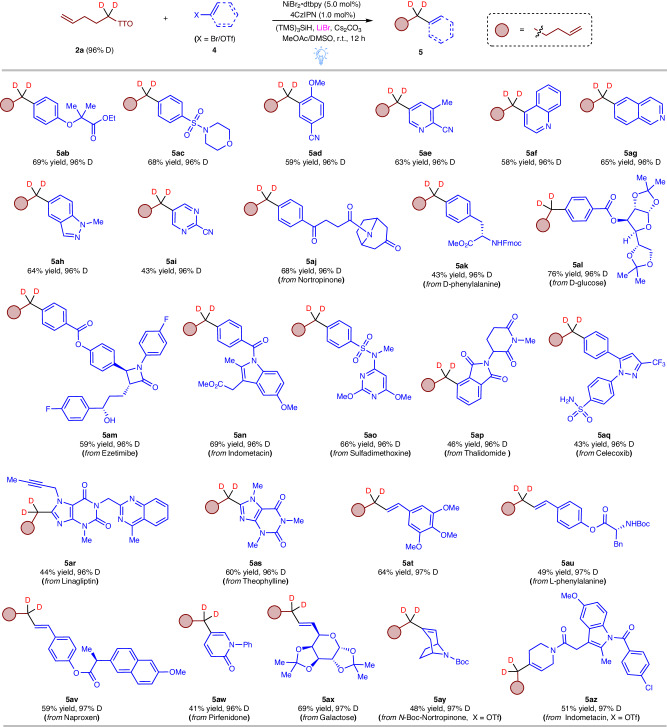
Substrate scope of (hetero)aryl bromides and enol triflates in Csp^3^−Csp^2^ coupling reactions. Standard reaction conditions: aryl bromide (0.2 mmol), TT salt (0.4 mmol), 4CzIPN (1.0 mol%), NiBr_2_•dtbpy (5.0 mol%), TMS_3_SiH (0.22 mmol), Cs_2_CO_3_ (0.4 mmol), LiBr (0.4 mmol), MeOAc (1.6 mL)/DMSO (0.4 mL), Blue LEDs, r.t., 24 h, isolated yields, deuterium incorporation was determined by ^1^H NMR spectroscopy and/or HRMS.

Encouraged by these results, we conducted further investigations into the reactivity of aryl bromides with d2-TT salt **2a** (Fig. 4). Both electron-deficient (**4b**) and electron-rich (**4c**) aryl bromides proved to be suitable substrates, yielding the desired coupling products **5ab** and **5ac** in good yields with high deuterium incorporation. Even the steric hindrance of *ortho*-substituted aryl bromides did not impede the smooth progress of the reaction (**5ad**). Additionally, a range of heteroaryl bromides, such as pyridinyl (**4e**), quinolinyl (**4f**), isoquinolinyl (**4g**), indazolyl (**4h**), and pyrimidinyl (**4i**) bromides, displayed compatibility with this reaction, affording the desired d2-alkyl substituted heteroarenes **5ae-ai** in good yields. The impressive functional group compatibility and excellent deuterium incorporation observed in these molecules prompted us to evaluate the reaction using commercially available pharmaceuticals and biologically active compounds. Substrates bearing amide or ester derivatives of natural products, such as nortropinone (**4j**), d-phenylalanine (**4k**), and d-glucose (**4l**), which possess carbonyl, ester, and amide functional groups susceptible to sensitivity, proved to be amenable substrates, yielding the corresponding products **5aj–al** in good yields. Furthermore, drug molecule derivatives, such as Ezetimibe (**3m**), Indomethacin (**4n**), Sulfadimethoxine (**4o**), Thalidomide (**4p**), and the Celecoxib derivative **4q** featuring an electron-neutral aryl bromide unit, all successfully underwent the reaction with d2-TT salt **2a**, resulting in the formation of products **5am–aq** with excellent deuterium incorporation. Expanding the substrate scope to derivatives of drugs containing heteroaryl bromide units, such as Linagliptin (**4r**) and Theophylline (**4s**), proved to be compatible with this transformation. Remarkably, even vinyl bromides showed reactivity as coupling partners. Both (*E*)-5-(2-bromovinyl)-1,2,3-trimethoxybenzene (**4t**) and styryl-containing complex molecules **4u–v** participated effectively in the coupling reaction with **2a**. Furthermore, treatment of brominated pirfenidone (**4w**) and Galactose (**4x**) under these conditions led to the formation of products with exceptional deuterium incorporation. Lastly, enol triflates derived from Pirfenidone (**4y**) and Indometacin (**4z**) also exhibited successive cross-coupling reactions.

**Fig. 5:**
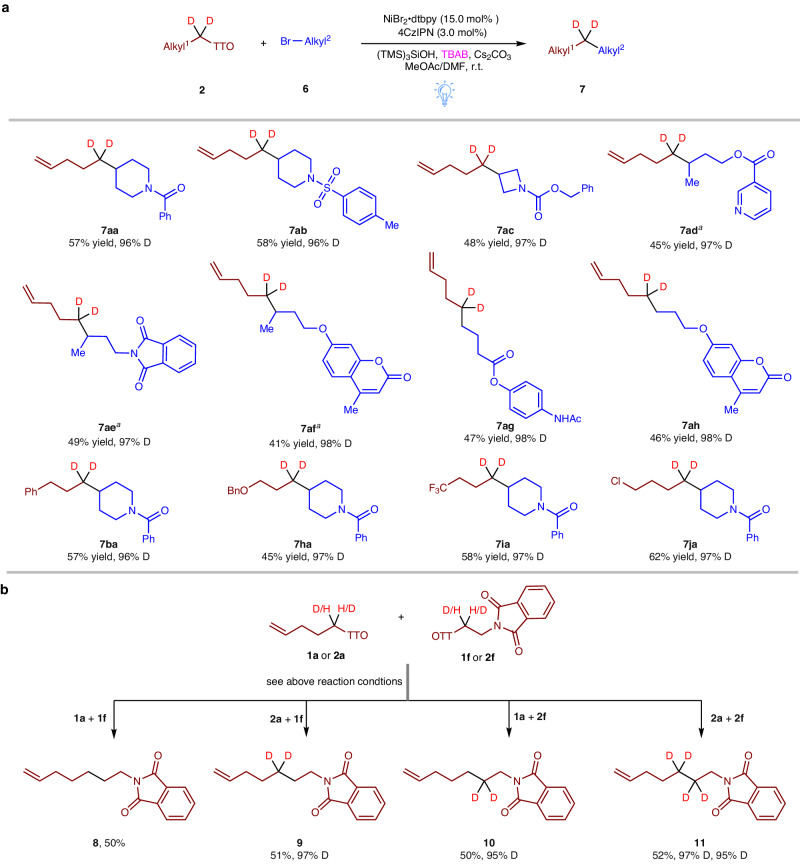
Substrate scope of Csp^3^−Csp^3^ coupling reactions. **a** Substrate scope of coupling alkyl bromides with d2-alkyl TT salts. **b** Cross-electrophile couplings between two alkyl TT salts. Reaction conditions: [6 (0.2 mmol), 2 (0.8 mmol)] or [1a/2a (0.2 mmol), 1f/2f (0.8 mmol)], 4CzIPN (3.0 mol%), NiBr_2_•dtbpy (15.0 mol%), (TMS)_3_SiOH (0.3 mmol), Cs_2_CO_3_ (0.4 mmol), Bu_4_NBr (0.8 mmol), MeOAc (1.6 mL)/DMF (0.4 mL), Blue LEDs, r.t., 16 h, isolated yields, deuterium incorporation was determined by ^1^H NMR spectroscopy and/or HRMS. **a** Using NiBr_2_•DME (15.0 mol%) and 1,3-Bis(4,5-dihydro-2-oxazolyl)benzene (15.0 mol%) instead of NiBr_2_•dtbpy (15.0 mol%).

Methods for the coupling of two alkyl centers are still relatively uncommon in the field of cross-electrophile coupling^54^. Our specific aim was to synthesize functionalized alkanes with a deuterium label located internally by utilizing alkyl bromides and d2-alkyl TT salts (Fig. 5a. For the optimization conditions, see details in Table S2 of SI). Our approach proved to be remarkably effective in coupling heterocyclic bromides, such as piperidines (**6a–b**) and azetidines (**6c**), which are commonly found in medicinal chemistry. These reactions resulted in the formation of the desired deuterated adducts **7aa–ac**, displaying moderate yields but with high deuterium incorporation. Regarding acyclic systems, we discovered that both secondary (**6d–f**) and primary (**6g–h**) alkyl bromides were amenable to coupling with d2-TT salt **2a**, allowing for the synthesis of the desired alkanes **7ad–ah**. Notably, these reactions exhibited over 96% deuterium incorporation at specific positions. Additionally, other d2-alkyl TT salts, including **2b,** **2h–j**, were employed for reaction with alkyl bromide **6a**, leading to the formation of desired products with high yields, alongside 96% deuterium incorporation. These findings underscore the remarkable potential and versatility of our metallaphotoredox-catalyzed cross-electrophile coupling reaction in the synthesis of functionalized alkanes bearing deuterium labels at internal positions. Based on the above results, we further investigated whether cross-electrophile coupling could occur between two alkyl TT salts. As shown in Fig. 5b, we conducted a coupling between alkyl TT salt **1a** and **1f** under optimized conditions. As a result, we obtained the desired coupling product **8** in a modest yield, simultaneously by-products of hydrogenation and self-coupling were formed. To achieve controlled distinct deuterated sites and high deuterium incorporation, we employed d2-TT salt **2a** or **2f** as reagent. This allowed us to obtain two isomers, **9** and **10**. Additionally, when compound **2a** and **2f** was used as the substrates, we obtained the neighbouring d4-alkane **11** with high deuterium incorporation. Such finding opens up new possibilities for synthetic methodologies and the development of deuterated compounds with specific isotopic labelling patterns.

### Synthetic applications

**Fig. 6:**
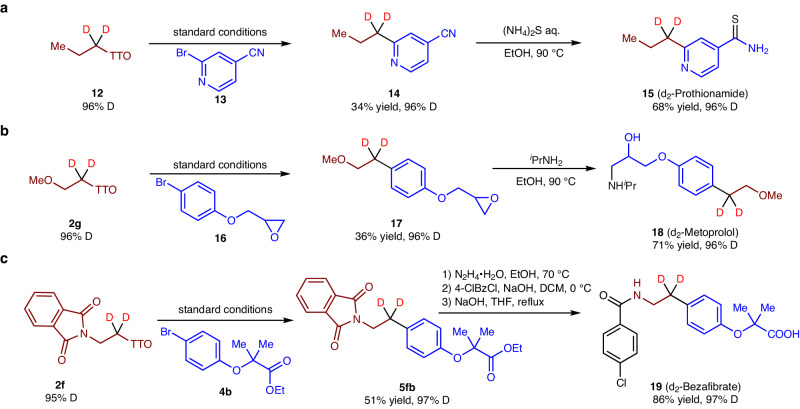
Total synthesis of deuterated drugs. **a** Synthesis of d2-Prothionamide from TT salt 11. **b** Synthesis of d2-Metoprolol from TT salt 2g. **c** Synthesis of d2-Bezafibrate from TT salt 2f.

The approval of deuterium-modified drugs such as Austedo and Donafenib has generated interest in the synthesis of drugs incorporating deuterium. To demonstrate the practicality of the metallaphotoredox process, we conducted a thorough investigation of the cross-electrophile coupling protocol by synthesizing deuterium-containing drugs using commercially available starting materials (Fig. 6). We successfully synthesized deuterated Prothionamide **15**^55^, an orally administered antitubercular drug, by coupling d2-alkyl salt **12** with pyridinyl bromide **13**, followed by sulfidation of the cyano group (Fig. 6a). Likewise, we achieved efficient synthesis of deuterated Metoprolol (**18**)^56^, a drug used for lung cancer treatment. In these syntheses, the d2-TT salt **2g** was coupled with aryl bromide **16** to form the central C–C bond, which subsequently reacted with propan-2-amine to yield the desired molecule (Fig. 6b). Additionally, we utilized the developed method to access deuterated Bezafibrate (**19**)^57^, a medication used to regulate lipid levels in the blood. Treatment of d2-TT salt **2f** with aryl bromide **4b** resulted in the efficient formation of the **5fb** product. Subsequent deprotection and benzoylation afforded the final product with satisfactory yield (Fig. 6c). The successful synthesis of these drugs with high deuterium incorporation underscores the compatibility and practicality of this method in terms of functional group transformations.

## Discussion

### Mechanistic investigation

**Fig. 7:**
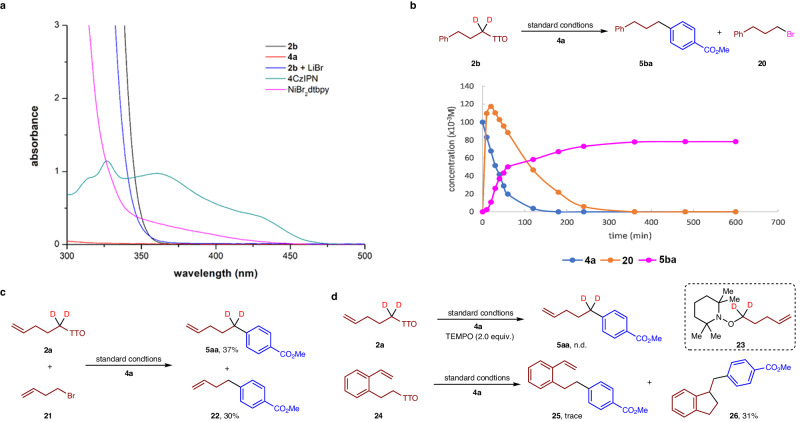
Mechanism experiments. **a** UV/vis study of the reaction components. **b** Tracing the reaction between **2b** and **4a**. **c** Competition reaction between alkyl TT salt **2a** and alkyl bromide **21**. **d** Radical trapping experiments.

Several experiments were then conducted to offer insights into the metallaphotocatalytic cross-coupling (Fig. 7). We first conducted an analysis to determine which species in the solution can absorb blue LED light at around 450 nm. By obtaining UV/vis absorption spectra of the components and their combinations, we identified the light-absorbing properties (Fig. 7a). The data clearly shows that a solution containing 4CzIPN can absorb light at 450 nm, while the nickel complex solution cannot. Furthermore, the TT salt **2b** exhibited no discernible absorption within the spectrum of visible light, and the introduction of LiBr induced a blue shift, elucidating the emergence of a new compound. To further substantiate the assertion, a reaction profile between the reactions of TT salt **2b** and aryl bromide **4a** under standard conditions was conducted (Fig. 7b). As the product **5ba** emerged, substrate **4a** gradually diminished, concomitant with the rapid generation of a significant quantity of alkyl bromide **20** right from the outset^58^. A competition reaction between TT salt **2a** and alkyl bromide **21** resulted in the formation of a product mixture containing **5aa** and **22** in a ratio of 1.2/1 (Fig. 7c). This observation further suggests that the bromination process is rapid, exerting no influence on the overall rate of the reaction. When TEMPO was used as a radical scavenger in the reaction, the TEMPO-captured product **23** was detected by HRMS, while the coupling product **5aa** was not observed (Fig. 7d). Furthermore, when a well-designed TT salt **24**, containing a styrene unit, was used as a substrate, a trace amount of direct coupling product **25** was observed. Additionally, compound **26**, resulting from the cascade of a 5-exo cyclization followed by coupling with aryl bromide **4a**, was isolated in 31% yield. These observations strongly support the formation of alkyl radicals during the reaction.

**Fig. 8:**
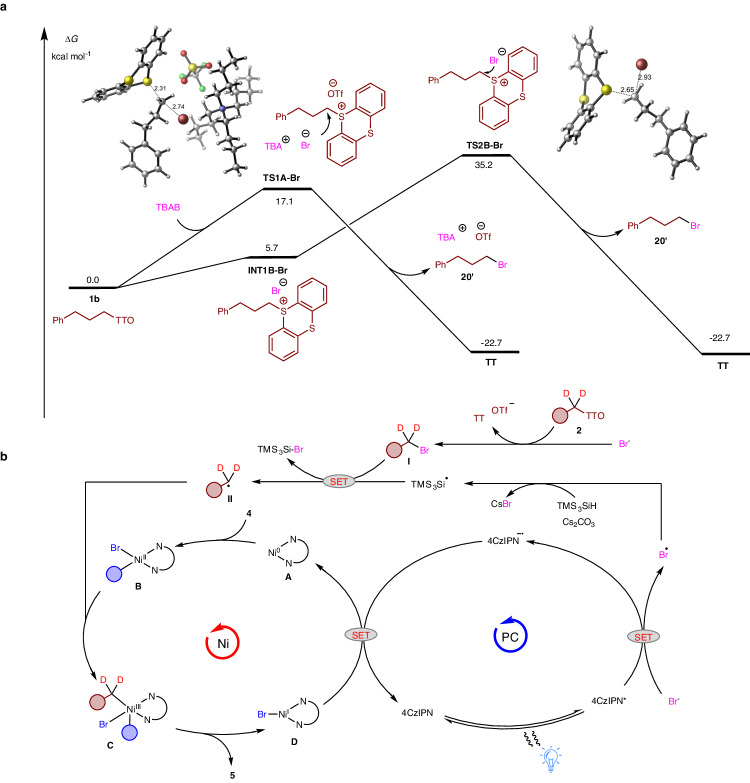
Proposed mechanism. **a** DFT-calculated energy profile for the bromination of alkyl TT salt **1b** with TBAB. **b** Full elucidated mechanism of the nickel cycle (left) and photoredox cycle (right).

In order to delve deeper into the conversion process of TT salts into alkyl bromides, we first conducted density functional theory (DFT) calculations pertaining in into the bromination of TT salt **1b** (Fig. 8a). TBAB attacks **1n** through the transition state **TS1A** in an S_*N*_2 manner, with an energy barrier of 17.1 kcal mol^−1^, which is much lower compared to the intramolecular S_*N*_2 nucleophilic substitution via transition state **TS2B** (17.1 vs 35.2 kcal mol^−1^). The mechanism involving S_*N*_1-typed nucleophilic attack is well excluded, as the C–S bond cleavage requires a high activation free energy of 36.9 kcal mol^−1^ (see Supplementary Fig. 10 for details). Based on the above results and previous studies^59^, a proposed mechanism for the reaction is presented in Fig. 8b. The photoredox catalyst 4CzlPN absorbs photons and gets excited to its photoexcited state (4CzlPN*). This species further undergoes single-electron oxidation of a bromine anion, generating a bromine radical (Br^•^) and the reduced photocomplex 4CzlPN^•−^. The electrophilic bromine radical (Br^•^) abstracts a hydrogen atom from TMS_3_SiH, resulting in the formation of a stabilized silyl radical (TMS_3_Si^•^). Meanwhile, bromination of alkyl TT salt **2** leads to the formation of the alkyl bromide **I**. This compound then undergoes bromine atom abstraction by the silyl radical (TMS_3_Si^•^), producing the corresponding nucleophilic alkyl radical species **II**. Simultaneously, oxidative addition of a Ni^0^ complex **A** to the aryl bromide **4** yields an intermediate **B**. This species undergoes radical addition with **II**, giving rise to the intermediate **C**. Importantly, an excess amount of alkyl source and silane is utilized during this step to enhance the selectivity for the desired cross-coupling product^60^. Further reductive elimination of **C** generates the desired product **5**, and nickel complex **D**. Single-electron reduction of **D** by the reduced organic photocatalyst (4CzlPN^•−^) completes both the photocatalytic cycle and the nickel catalytic cycle, leading to the restoration of the starting Ni^0^ catalyst **A** and photocatalysis.

In summary, a robust method has been developed for the synthesis of versatile alkanes containing two deuterium atoms. A library of novel deuteroalkyl reagents was generated by subjecting alkyl-substituted TT salts to a pH-dependent HIE process using D_2_O as the source of deuterium. These readily available d_2_-TT salts can then undergo a metallaphotoredox-catalyzed cross-electrophile coupling with a range of electrophiles, including aryl, heteroaryl, alkenyl, and alkyl bromides, as well as other alkyl TT salts. Our approach possesses several notable strengths. Firstly, it employs commercially available catalysts, rendering it practical and accessible for wide-ranging applications. Secondly, it provides precise control over the placement of deuterium atoms, ensuring cross selectivity and regioselectivity. Importantly, the reaction conditions do not necessitate the use of metal reductants, thereby simplifying the process and circumventing the need for additional steps. Additionally, our strategy accomplishes high levels of deuterium incorporation, enabling accurate positioning of deuterium atoms within the synthesized compounds. We anticipate that this powerful platform will facilitates research in medicine, biology, and chemistry.

## Methods

### General procedures for metallaphotoredox-catalyzed Csp^2^-Csp^3^ coupling

In a nitrogen-filled glove box, alkyl TT salt (0.4 mmol, 2.0 equiv.), Cs_2_CO_3_ (130.3 mg, 0.4 mmol, 2.0 equiv.), LiBr (34.7 mg, 0.4 mmol, 2.0 equiv.), 4CzIPN (1.8 mg, 1 mol%), and NiBr_2_•dtbpy (4.9 mg, 5 mol%) were added to an 8 mL oven-dried vial equipped with a stir bar. Then, anhydrous MeOAc (1.6 mL) and anhydrous DMSO (0.4 mL) were added using a syringe, followed by the addition of (hetero)aryl bromide (0.2 mmol, 1.0 equiv.) and tris(trimethylsilyl)silane (68 µL, 0.22 mmol, 1.1 equiv.). The vial was sealed and removed from the glovebox. Subsequently, the reaction mixture was stirred and irradiated with a 40 W blue LED lamp for 12 h. The final reaction mixture was diluted with EtOAc (60 mL) and saturated aqueous LiCl solution (20 mL). The organic layer was washed with brine (2 × 20 mL) and concentrated. The aryl alkylation product was purified by flash column chromatography on silica gel.

### General procedures for metallaphotoredox-catalyzed Csp^3^-Csp^3^ coupling

In a nitrogen-filled glove box, alkyl TT salts (0.8 mmol, 4.0 equiv), Cs_2_CO_3_ (130.3 mg, 0.4 mmol, 2.0 equiv), TBAB (257.9 mg, 0.8 mmol, 4.0 equiv), 4CzIPN (4.8 mg, 3 mol%), and NiBr_2_•dtbpy (14.6 mg, 15 mol%) were added to an 8 mL oven-dried vial equipped with a stir bar. Anhydrous MeOAc (1.6 mL) and anhydrous DMF (0.4 mL) were then added *via* a syringe, followed by the addition of an alkyl bromide (0.2 mmol, 1.0 equiv) and tris(trimethylsilyl)silanol (95 µL, 0.3 mmol, 1.5 equiv). The vial was sealed and removed from the glovebox. Subsequently, the reaction mixture was stirred and irradiated with a 40 W blue LED lamp for 16 h. The final reaction mixture was diluted with EtOAc (60 mL) and saturated aqueous LiCl solution (20 mL). The organic layer was washed with brine (2 × 20 mL) and concentrated. The alkyl alkylation product was purified by flash column chromatography on silica gel.

### Supplementary information

Supplementary Information

Peer Review File

### Source data

Source Data

## Data Availability

The data supporting the findings of this study are available within the paper and its Supplementary Information files. Coordinates of the optimized structures are provided in a source data file. Raw data are available from the corresponding author on request. Source data are provided with this paper.
