# Supplementary material for: Metallaphotoredox deuteroalkylation utilizing thianthrenium salts
Source: Nat Commun. 2024 Jun 13;15:5067. doi: 10.1038/s41467-024-48590-w (PMC11176366; doi:10.1038/s41467-024-48590-w)
Supplement: Supplementary file 1 — Supplementary Information [file 41467_2024_48590_MOESM1_ESM.pdf]

Supplementary information for

Metallaphotoredox deuteroalkylation utilizing thianthrenium salts

Mengjie Jiao[1,†], Jie Zhang[1,†], Minyan Wang[1], Hongjian Lu[1,*] and Zhuangzhi Shi[1,*]

[1]State Key Laboratory of Coordination Chemistry, Chemistry and Biomedicine Innovation Center (ChemBIC), School of Chemistry and Chemical Engineering, Nanjing University, Nanjing 210093, China.

[†]These authors contributed equally to this work.

*e-mail: hongjianlu@nju.edu.cn; shiz@nju.edu.cn

Table of Contents

Supplementary Methods **2**

1. General information 2
2. General procedure for deuteration of S-(alkyl)thianthrenium salts 3
3. Reaction optimization 24
4. Procedure for metallaphotoredox-catalyzed sp^3C-sp^2C and sp^3C-sp^3C couling reaction 25
5. Cross-electrophile coupling reactions between two alkyl thianthrenium salts 67
6. Total synthesis of deuterated drugs 69
7. Mechanistic experiments 76
8. The calculation of aqueous pKa values and BDE 81
9. NMR Spectra 85

Supplementary References **283**

Supplementary Methods

1. General information

All new compounds were fully characterized. NMR spectra were recorded on Bruker 400 MHz, Varian 500 and calibrated using residual undeuterated solvent ($CHCl_3$ = 7.26 ppm ^{1}H NMR, 77.00 ppm ^{13}C NMR; DMSO = 2.50 ppm ^{1}H NMR, 39.50 ppm ^{13}C NMR; CH_3CN = 1.94 ppm ^{1}H NMR, 1.32 ppm ^{13}C NMR) or TMS as an internal reference. ^{1}H NMR coupling constants were reported in Hz, and multiplicity was indicated as follows: s (singlet); d (doublet); t (triplet); q(quartet); m (multiplet); dd (doublet of doublets); tt (triplet of triplets), ddd (doublet of doublet of doublets); dt (doublet of triplets); td (triplet of doublets); br (broad). Mass spectra were conducted at Mass spectra were conducted at Thermo Scientific LCQ Fleet ESI Mass Spectrometer. For the ReactIR kinetic experiments, the reaction spectra were recorded using an IC 10 and IC 15 from Mettler-Toledo AutoChem. Data manipulation was carried out using the iC IR software, version 4.2. Anhydrous solvents, such as dichloromethane (DCM), dimethyl sulfoxide (DMSO), were purchased from Aldrich. Flash column chromatography was carried out using silica gel (Silicycle, SiliaFlash® P60, 40-63 μm). Unless otherwise noted, materials were obtained from commercial suppliers (Alfa, TCI, J&K Chemical, Energy Chemical, Adamas-beta, Bidepharm, Toronto Research Chemicals, Aladdin, leyan etc.) and used without further purification.

All Photoredox reactions were subjected to irradiation from a 40W Kessil blue LED bulb (440 nm), with the reaction tube placed approximately ~ 1 cm from the bulb in water bath to keep the temperature at 25 °C (Supplementary Figure 1).

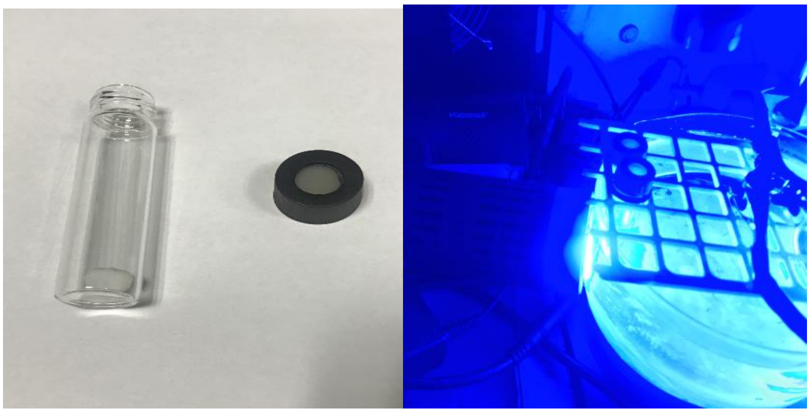

Supplementary Figure 1. A dried 8 mL tube (left). Photoreaction setup (right).

2. General procedure for deuteration of S-(alkyl)thianthrenium salts

(a) Synthesis of d₂-alkyl TT salts

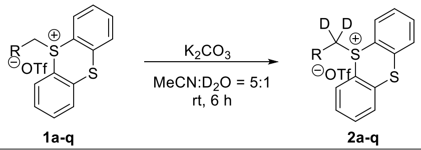

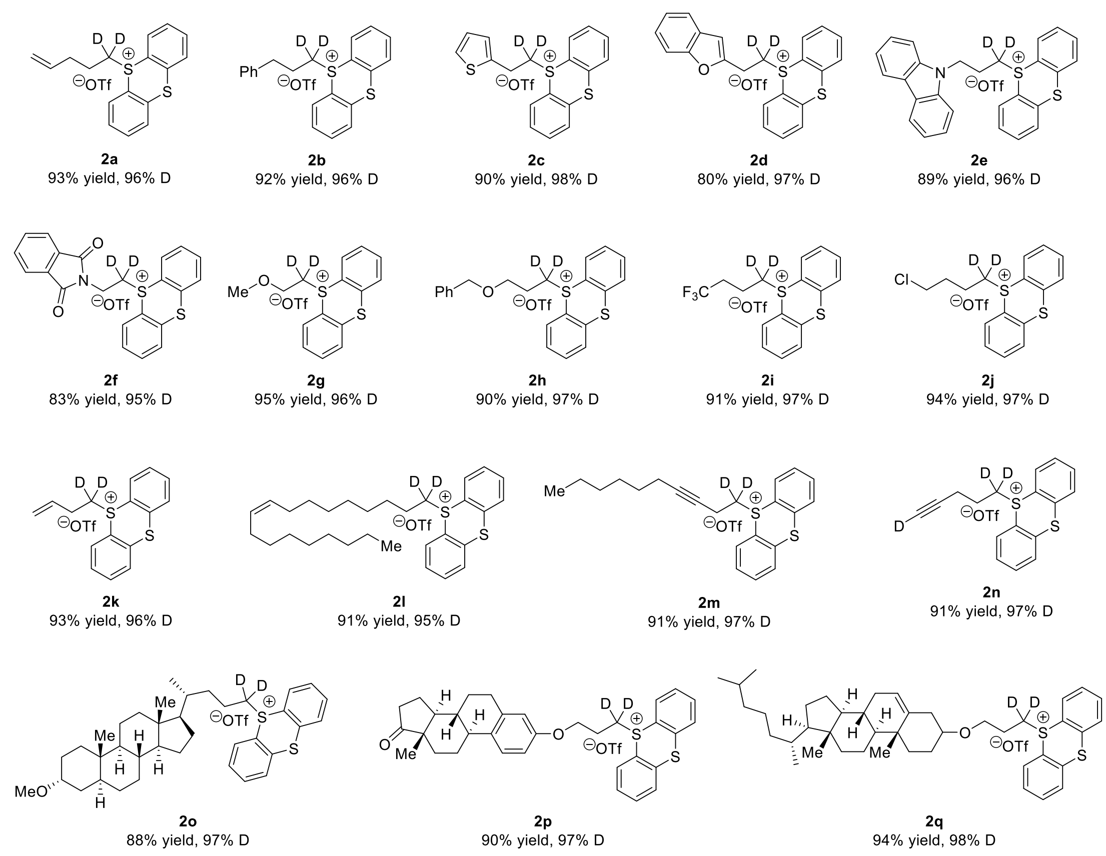

General procedure A: To a flame-dried 50 mL flask with a stir bar was added the S-(alkyl)thianthrenium salts[1] (2.0 mmol, 1.0 equiv.), K_2CO_3 (4.0 mmol, 2.0 equiv.), MeCN (10 mL) and D_2O (2 mL). The reaction was stirred at room temperature. After 6 h, the reaction mixture was dried over anhydrous $MgSO_4$, then filtered and concentrated under reduced pressure. Finally, the product was purified by precipitation with Et_2O/DCM. Deuterium incorporation of the product was determined by ^1H NMR spectroscopy.

5-(pent-4-en-1-yl-1,1-*d*₂)-5*H*-thianthren-5-ium trifluoromethanesulfonate (2a)

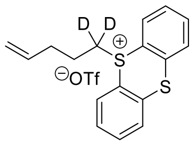

According to General procedure A, **2a** was prepared as a white solid (811.2 mg, 93% yield, 96% D): **^{1}H NMR (400 MHz, CDCl$_3$)** δ 8.29 (dd, *J* = 7.9, 1.3 Hz, 2H), 7.83 (dd, *J* = 7.9, 1.3 Hz, 2H), 7.76 (td, *J* = 7.7, 1.4 Hz, 2H), 7.66 (td, *J* = 7.7, 1.4 Hz, 2H), 5.58 (ddt, *J* = 17.0, 10.3, 6.7 Hz, 1H), 5.00 – 4.86 (m, 2H), 2.13 (q, *J* = 7.1 Hz, 2H), 1.65 (t, *J* = 7.2 Hz, 2H); **^{13}C NMR (101 MHz, CDCl$_3$)** δ 135.7, 135.2, 134.7, 134.5, 130.0, 129.9, 120.8 (q, *J* = 320.8 Hz), 117.2, 117.1, 31.4, 23.2; **^{19}F NMR (376 MHz, CDCl$_3$)** *δ* -78.1; **ATR-FTIR (cm $^{-1}$):** 3088, 2930, 1637, 1570, 1449, 1253, 1150, 1024, 932, 759, 634, 515, 453; **HRMS m/z (ESI)** calculated for $C_{17}H_{15}D_2S_2^+$ $[M – O_3SCF_3]^+$ 287.0892, found 287.0898.

The characterization data of the corresponding non-deuterated product are as follows:

5-(pent-4-en-1-yl)-5*H*-thianthren-5-ium trifluoromethanesulfonate (1a)

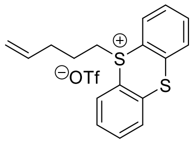

^{1}H NMR (400 MHz, CDCl$_3$) δ 8.26 (dd, *J* = 7.9, 1.2 Hz, 2H), 7.83 (dd, *J* = 7.9, 1.1 Hz, 2H), 7.75 (td, *J* = 7.7, 1.4 Hz, 2H), 7.64 (td, *J* = 7.7, 1.4 Hz, 2H), 5.57 (ddt, *J* = 17.0, 10.3, 6.7 Hz, 1H), 5.07 – 4.83 (m, 2H), 3.85 – 3.62 (m, 2H), 2.12 (q, *J* = 7.1 Hz, 2H), 1.77 – 1.52 (m, 2H); **^{13}C NMR (101 MHz, CDCl$_3$)** δ 135.6, 135.1, 134.5, 130.1, 129.8, 120.8 (d, *J* = 320.5 Hz), 117.1, 39.7, 31.4, 23.4; **^{19}F NMR (376 MHz, CDCl$_3$)** δ -78.1; **ATR-FTIR (cm $^{-1}$):** 3088, 2930, 1637, 1570, 1449, 1253, 1149, 1024, 933, 759, 634, 515, 455; **HRMS m/z (ESI)** calculated for $C_{17}H_{17}S_2^+$ $[M – O_3SCF_3]^+$ 285.0766, found 285.0770.

5-(3-phenylpropyl-1,1-*d*$_2$)-5*H*-thianthren-5-ium trifluoromethanesulfonate (2b)

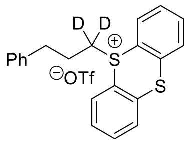

According to General procedure A, **2b** was prepared as a white solid (894.2 mg, 92% yield, 96% D): **^{1}H NMR (500 MHz, CDCl$_3$)** δ 8.21 (d, *J* = 8.0 Hz, 2H), 7.81 – 7.69 (m, 4H), 7.66 – 7.59 (m, 2H), 7.23 – 7.13 (m, 3H), 7.00 (d, *J* = 7.0 Hz, 2H), 2.69 (t, *J* = 7.5 Hz, 2H), 1.86 (t, *J* = 7.5 Hz, 2H); **^{13}C NMR (126 MHz, CDCl$_3$)** δ 139.0, 135.7, 134.7, 134.5, 130.1, 129.9, 128.7, 128.4, 126.7, 120.90 (q, *J* = 321.3 Hz), 117.1, 33.5, 25.6; **^{19}F NMR (471 MHz, CDCl$_3$)** δ -78.0; **ATR-FTIR (cm $^{-1}$):** 3493, 3061, 2995, 2925, 1258, 1028, 726, 459; **HRMS m/z (ESI)** calculated for $C_{21}H_{17}D_2S_2^+$ $[M – O_3SCF_3]^+$ 337.1048, found 337.1044.

The characterization data of the corresponding non-deuterated product are as follows:

5-(3-phenylpropyl)-5*H*-thianthren-5-ium trifluoromethanesulfonate (1b)

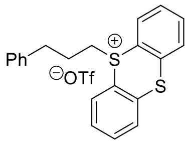

^{1}H NMR (500 MHz, $CDCl_3$) δ 8.21 (d, *J* = 7.9 Hz, 2H), 7.78 (dd, *J* = 7.9, 1.2 Hz, 2H), 7.73 (td, *J* = 7.7, 1.2 Hz, 2H), 7.62 (t, *J* = 8.1 Hz, 2H), 7.18 (dq, *J* = 14.4, 7.1 Hz, 3H), 7.00 (d, *J* = 6.9 Hz, 2H), 3.78 – 3.72 (m, 2H), 2.69 (t, *J* = 7.4 Hz, 2H), 1.96 – 1.82 (m, 2H); **^{13}C NMR (126 MHz, $CDCl_3$)** δ 138.9, 135.6, 134.6, 134.5, 130.1, 129.8, 128.6, 128.3, 126.6, 120.8 (q, *J* = 320.0 Hz), 117.0, 39.3, 33.4, 25.7; **^{19}F NMR (471 MHz, $CDCl_3$)** δ -78.0; **ATR-FTIR (cm $^{-1}$):** 3117, 2934, 1530, 1081, 765, 635; **HRMS m/z (ESI)** calculated for $C_{21}H_{19}S_2^+$ $[M – O_3SCF_3]^+$ 335.0923, found 335.0924.

5-(2-(thiophen-2-yl)ethyl-1,1-*d*$_2$)-5*H*-thianthren-5-ium trifluoromethanesulfonate (2c)

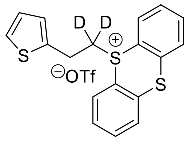

According to General procedure A, **2c** was prepared as a white solid (861.3 mg, 90% yield, 98% D): **^{1}H NMR (400 MHz, $CDCl_3$)** δ 8.12 (d, *J* = 7.6 Hz, 2H), 7.83 (d, *J* = 7.6 Hz, 2H), 7.73 (t, *J* = 7.6 Hz, 2H), 7.60 (t, *J* = 7.6 Hz, 2H), 7.08 (d, *J* = 5.2 Hz, 1H), 6.97 (d, *J* = 3.2 Hz, 1H), 6.86 (dd, *J* = 5.2, 3.6 Hz, 1H), 3.30 (s, 2H); **^{13}C NMR (101 MHz, $CDCl_3$)** δ 137.0, 135.8, 134.4, 130.1, 129.8, 127.4, 127.2, 125.5, 120.8 (q, *J* = 322.2 Hz), 117.4, 25.0; **^{19}F NMR (376 MHz, $CDCl_3$)** δ -78.0; **ATR-FTIR (cm $^{-1}$):** 2998, 1573, 1460, 1278, 1160, 1084, 1034, 764; **HRMS m/z (ESI)** calculated for $C_{18}H_{13}D_2S_3^+$ $[M – O_3SCF_3]^+$ 329.0456, found 329.0457.

The characterization data of the corresponding non-deuterated product are as follows:

5-(2-(thiophen-2-yl)ethyl)-5*H*-thianthren-5-ium trifluoromethanesulfonate (1c)

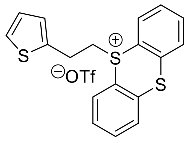

^{1}H NMR (400 MHz, $CDCl_3$) δ 8.07 (dd, *J* = 7.9, 1.1 Hz, 2H), 7.80 (dd, *J* = 7.9, 1.0 Hz, 2H), 7.70 (td, *J* = 7.8, 1.3 Hz, 2H), 7.56 (td, *J* = 7.8, 1.2 Hz, 2H), 7.05 (dd, *J* = 5.1, 1.1 Hz, 1H), 6.93 (d, *J* = 2.7 Hz, 1H), 6.82 (dd, *J* = 5.1, 3.5 Hz, 1H), 4.05 (t, *J* = 7.2 Hz, 2H), 3.27 (t, *J* = 7.2 Hz, 2H); **^{13}C NMR (101 MHz, $CDCl_3$)** δ 137.0, 135.7, 134.4, 134.2, 130.1, 129.8, 127.4, 127.2, 125.5, 120.7 (q, *J* = 322.2 Hz), 117.3, 42.3, 25.1; **^{19}F NMR (376 MHz, $CDCl_3$)** δ -78.1; **ATR-FTIR (cm $^{-1}$):** 3077, 1255, 1152, 1028, 757, 635; **HRMS m/z (ESI)** calculated for $C_{18}H_{15}S_3^+$ $[M – O_3SCF_3]^+$ 327.0330, found 327.0330.

5-(2-(benzofuran-2-yl)ethyl-1,1-d_2)-5*H*-thianthren-5-ium trifluoromethanesulfonate (2d)

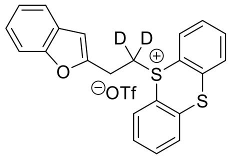

According to General procedure A, **2d** was prepared as a white solid (820.5 mg, 80% yield, 97% D): **^{1}H NMR (400 MHz, $CDCl_3$)** δ 8.01 (dd, *J* = 8.0, 1.2 Hz, 2H), 7.71 (dd, *J* = 8.0, 1.1 Hz, 2H), 7.53 (td, *J* = 7.8, 1.3 Hz, 2H), 7.44 – 7.37 (m, 1H), 7.32 (td, *J* = 7.8, 1.2 Hz, 2H), 7.21 – 7.05 (m, 3H), 6.62 (d, *J* = 0.7 Hz, 1H), 3.40 (s, 2H); **^{13}C NMR (101 MHz, $CDCl_3$)** δ 154.4, 151.3, 135.8, 134.2, 134.0, 130.0, 129.4, 127.9, 124.2, 123.0, 120.9, 120.8 (q, *J* = 320.6 Hz), 117.2, 110.7, 105.9, 24.0; **^{19}F NMR (376 MHz, $CDCl_3$)** δ -78.0; **ATR-FTIR (cm $^{-1}$):** 2971, 1460, 1281, 1160, 1075, 947, 876, 758; **HRMS m/z (ESI)** calculated for $C_{22}H_{15}D_2OS_2^+$ [M – O_3SCF_3]$^+$ 363.0841, found 363.0840.

The characterization data of the corresponding non-deuterated product are as follows:

5-(2-(benzofuran-2-yl)ethyl)-5*H*-thianthren-5-ium trifluoromethanesulfonate (1d)

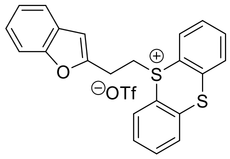

^{1}H NMR (400 MHz, $CDCl_3$) δ 7.97 (d, *J* = 7.8 Hz, 2H), 7.69 (d, *J* = 7.8 Hz, 2H), 7.52 (t, *J* = 7.7 Hz, 2H), 7.40 – 7.34 (m, 1H), 7.30 (t, *J* = 7.7 Hz, 2H), 7.16 – 7.04 (m, 3H), 6.59 (s, 1H), 4.16 (t, *J* = 6.6 Hz, 2H), 3.36 (t, *J* = 6.6 Hz, 2H); **^{13}C NMR (101 MHz, $CDCl_3$)** δ 154.3, 151.2, 135.7, 134.0, 134.0, 129.8, 129.3, 127.8, 124.1, 122.9, 120.8, 120.7 (q, *J* = 320.7 Hz), 117.0, 110.6, 105.8, 39.6, 24.1; **^{19}F NMR (376 MHz, $CDCl_3$)** δ -78.0; **ATR-FTIR (cm $^{-1}$):** 3062, 1263, 1156, 1029, 753, 637; **HRMS m/z (ESI)** calculated for $C_{22}H_{17}OS_2^+$ [M – O_3SCF_3]$^+$ 361.0715, found 361.0718.

5-(3-(9*H*-carbazol-9-yl)propyl-1,1-d_2)-5*H*-thianthren-5-ium trifluoromethanesulfonate (2e)

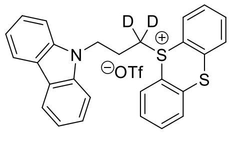

According to General procedure A, **2e** was prepared as a white solid (1024.4 mg, 89% yield, 96% D): **^{1}H NMR (400 MHz, $CDCl_3$)** δ 8.07 (d, *J* = 7.7 Hz, 2H), 7.53 – 7.40 (m, 6H), 7.37 (d, *J* = 8.1 Hz, 2H), 7.34 – 7.23 (m, 4H), 7.00 (d, *J* = 7.8 Hz, 2H), 4.47 (t, *J* = 6.0 Hz, 2H), 2.11 (t, *J* = 5.9 Hz, 2H); **^{13}C NMR (101 MHz, $CDCl_3$)** δ 139.6, 135.1, 134.2, 133.4, 129.7, 129.2, 126.3, 122.8, 120.7 (q, *J* = 320.8 Hz), 120.5, 119.5, 115.6, 108.9, 40.4, 22.4; **^{19}F NMR (376 MHz, $CDCl_3$)** δ -78.0; **ATR-FTIR (cm $^{-1}$):** 2361, 1451, 1259, 1151, 1025, 751, 636, 516; **HRMS m/z (ESI)** calculated for $C_{27}H_{20}D_2NS_2^+$ [M – O_3SCF_3]$^+$ 426.1314, found 426.1310.

The characterization data of the corresponding non-deuterated product are as follows:

5-(3-(9*H*-carbazol-9-yl)propyl)-5*H*-thianthren-5-ium trifluoromethanesulfonate (1e)

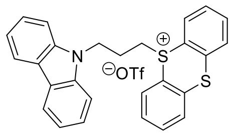

^{1}H NMR (400 MHz, $CDCl_3$) δ 8.10 (d, *J* = 7.7 Hz, 2H), 7.54 – 7.42 (m, 6H), 7.40 – 7.31 (m, 4H), 7.29 (d, *J* = 7.7 Hz, 2H), 7.22 – 7.15 (m, 2H), 4.51 (t, *J* = 6.0 Hz, 2H), 3.73 (t, *J* = 6.9 Hz, 2H), 2.28 – 2.11 (m, 2H); **^{13}C NMR (101 MHz, $CDCl_3$)** δ 139.8, 135.3, 134.2, 133.8, 129.8, 129.3, 126.4, 123.0, 120.8 (d, *J* = 320.5 Hz), 120.6, 119.6, 116.0, 109.0, 40.6, 37.8, 22.9; **^{19}F NMR (376 MHz, $CDCl_3$)** δ -78.0; **ATR-FTIR (cm $^{-1}$):** 2361, 1451, 1259, 1151, 1026, 752, 635, 516; **HRMS m/z (ESI)** calculated for $C_{27}H_{22}NS_2^+$ [M – O_3SCF_3]$^+$ 424.1188, found 424.1193.

5-(2-(1,3-dioxoisoindolin-2-yl)ethyl-1,1-*d*$_2$)-5*H*-thianthren-5-ium trifluoromethanesulfonate (2f)

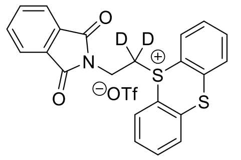

According to General procedure A, **2f** was prepared as a white solid (900.1 mg, 83% yield, 95% D): **^{1}H NMR (400 MHz, CD_3CN)** δ 8.23 (dd, *J* = 8.0, 1.2 Hz, 2H), 7.94 (dd, *J* = 8.0, 1.2 Hz, 2H), 7.92 – 7.84 (m, 4H), 7.78 (td, *J* = 7.6, 1.2 Hz, 2H), 7.67 (td, *J* = 7.6, 1.2 Hz, 2H), 3.81 (s, 2H); **^{13}C NMR (101 MHz, CD_3CN)** δ 169.0, 137.1, 135.8, 135.7, 132.8, 131.3, 130.6, 124.4, 122.2 (q, *J* = 321.1 Hz), 117.3, 33.4.; **^{19}F NMR (376 MHz, CD_3CN)** δ -79.1; **ATR-FTIR (cm $^{-1}$):** 2997, 1782, 1722, 1463, 1365, 1263, 1155, 978; **HRMS m/z (ESI)** calculated for $C_{22}H_{14}D_2NO_2S_2^+$ [M – O_3SCF_3]$^+$ 392.0743, found 392.0743.

The characterization data of the corresponding non-deuterated product are as follows:

5-(2-(1,3-dioxoisoindolin-2-yl)ethyl)-5*H*-thianthren-5-ium trifluoromethanesulfonate (1f)

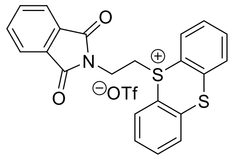

^{1}H NMR (400 MHz, CD_3CN) δ 8.23 (dd, *J* = 8.0, 1.2 Hz, 2H), 7.93 (dd, *J* = 8.0, 0.8 Hz, 2H), 7.91 – 7.83 (m, 4H), 7.78 (td, *J* = 8.0, 1.2 Hz, 2H), 7.67 (td, *J* = 8.0, 1.2 Hz, 2H), 4.02 – 3.95 (m, 2H), 3.85 – 3.75 (m, 2H); **^{13}C NMR (101 MHz, CD_3CN)** δ 169.1, 137.2, 135.9, 135.8, 135.8, 133.0, 131.4, 130.7, 124.5, 122.3 (q, *J* = 322.2 Hz), 117.5, 40.5, 33.6; **^{19}F NMR (376 MHz, CD_3CN)** δ -79.2; **ATR-FTIR (cm $^{-1}$):** 2910, 1780, 1722, 1463, 1368, 1260, 1166, 936; **HRMS m/z (ESI)** calculated for $C_{22}H_{16}NO_2S_2^+$ [M –

$O_3SCF_3]^+$ 390.0617, found 390.0618.

5-(2-methoxyethyl-1,1-d_2)-5*H*-thianthren-5-ium trifluoromethanesulfonate (2g)

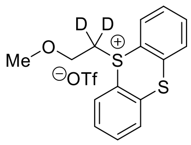

According to General procedure A, **2g** was prepared as a white solid (810.6 mg, 95% yield, 96% D): **^{1}H NMR (400 MHz, CDCl$_3$)** δ 8.19 (d, J = 7.9 Hz, 2H), 7.82 (d, J = 7.9 Hz, 2H), 7.78 – 7.69 (m, 2H), 7.67 – 7.60 (m, 2H), 3.65 (s, 2H), 3.19 (s, 3H); **^{13}C NMR (101 MHz, CDCl$_3$)** δ 136.1, 134.4, 134.3, 130.1, 129.7, 120.6 (q, J = 320.7 Hz), 117.4, 65.7, 58.7; **^{19}F NMR (376 MHz, CDCl$_3$)** δ -78.2; **ATR-FTIR (cm $^{-1}$):** 3002, 2360, 1568, 1450, 1261, 1149, 1030, 770, 636, 456; **HRMS m/z (ESI)** calculated for $C_{15}H_{13}D_2OS_2^+$ [M – $O_3SCF_3]^+$ 277.0684, found 277.0679.

The characterization data of the corresponding non-deuterated product are as follows:

5-(2-methoxyethyl)-5*H*-thianthren-5-ium trifluoromethanesulfonate (1g)

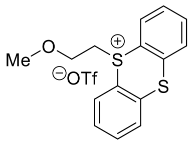

^{1}H NMR (400 MHz, CDCl$_3$) δ 8.22 (dd, J = 7.9, 1.3 Hz, 2H), 7.83 (dd, J = 7.9, 1.2 Hz, 2H), 7.73 (td, J = 7.7, 1.4 Hz, 2H), 7.64 (td, J = 7.7, 1.4 Hz, 2H), 4.05 (t, J = 5.2 Hz, 2H), 3.69 (t, J = 5.2 Hz, 2H), 3.20 (s, 3H); **^{13}C NMR (101 MHz, CDCl$_3$)** δ 136.1, 134.4, 134.3, 130.1, 129.7, 120.7 (q, J = 320.7 Hz), 117.6, 65.9, 58.7, 42.1; **^{19}F NMR (376 MHz, CDCl$_3$)** δ -78.1; **ATR-FTIR (cm $^{-1}$):** 3002, 2361, 1568, 1450, 1260, 1148, 1028, 772, 635, 458; **HRMS m/z (ESI)** calculated for $C_{15}H_{15}OS_2^+$ [M – $O_3SCF_3]^+$ 275.0559, found 275.0558.

5-(3-(benzyloxy)propyl-1,1-d_2)-5*H*-thianthren-5-ium trifluoromethanesulfonate (2h)

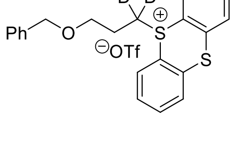

According to General procedure A, **2h** was prepared as a white solid (928.8 mg, 90% yield, 97% D): **^{1}H NMR (400 MHz, CDCl$_3$)** δ 8.05 (dd, J = 7.9, 1.0 Hz, 2H), 7.76 (dd, J = 7.9, 1.0 Hz, 2H), 7.68 (td, J = 7.8, 1.2 Hz, 2H), 7.53 (td, J = 7.8, 1.2 Hz, 2H), 7.43 – 7.23 (m, 5H), 4.53 (s, 2H), 3.66 (t, J = 5.4 Hz, 2H), 1.78 (t, J = 5.4 Hz, 2H); **^{13}C NMR (101 MHz, CDCl$_3$)** δ 137.4, 135.4, 134.7, 134.1, 129.9, 129.4, 128.2, 127.7, 120.7 (q, J = 322.2 Hz), 117.0, 73.1, 67.8, 24.5; **^{19}F NMR (376 MHz, CDCl$_3$)** δ -78.0; **ATR-FTIR (cm $^{-1}$):** 3057, 2869, 1570, 1452, 1262, 1158, 895, 635; **HRMS m/z (ESI)** calculated for

$C_{22}H_{19}D_2OS_2^+$ [M – O_3SCF_3]$^+$ 367.1154, found 367.1150.

The characterization data of the corresponding non-deuterated product are as follows:

5-(3-(benzyloxy)propyl)-5*H*-thianthren-5-ium trifluoromethanesulfonate (1h)

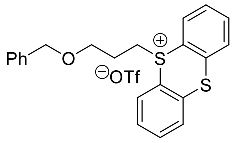

^{1}H NMR (400 MHz, $CDCl_3$) δ 8.06 (d, J = 7.8 Hz, 2H), 7.76 (d, J = 7.7 Hz, 2H), 7.68 (t, J = 7.5 Hz, 2H), 7.54 (t, J = 7.5 Hz, 2H), 7.42 – 7.24 (m, 5H), 4.53 (s, 2H), 3.92 (t, J = 6.5 Hz, 2H), 3.66 (t, J = 5.2 Hz, 2H), 1.88 – 1.73 (m, 2H); **^{13}C NMR (101 MHz, $CDCl_3$)** δ 137.5, 135.5, 134.8, 134.2, 129.9, 129.4, 128.3, 127.8, 120.7 (q, J = 322.2 Hz), 117.2, 73.2, 67.9, 39.2, 24.8; **^{19}F NMR (376 MHz, $CDCl_3$)** δ -78.0; **ATR-FTIR (cm $^{-1}$):** 3062, 2900, 1535, 1275, 1130, 1081, 755, 636; **HRMS m/z (ESI)** calculated for $C_{22}H_{21}OS_2^+$ [M – O_3SCF_3]$^+$ 365.1034, found 365.1035.

5-(4,4,4-trifluorobutyl-1,1-*d*$_2$)-5*H*-thianthren-5-ium trifluoromethanesulfonate (2i)

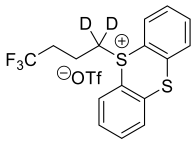

According to General procedure A, **2i** was prepared as a white solid (871.1 mg, 91% yield, 97% D): **^{1}H NMR (400 MHz, CD_3CN)** δ 8.14 (dd, J = 8.0, 1.2 Hz, 2H), 7.94 (dd, J = 8.0, 1.2 Hz, 2H), 7.83 (td, J = 8.0, 1.2 Hz, 2H), 7.70 (td, J = 7.6, 1.2 Hz, 2H), 2.35 – 2.22 (m, 2H), 1.76 (t, J = 8.0 Hz, 2H); **^{13}C NMR (101 MHz, CD_3CN)** δ 137.0, 135.8, 135.3, 131.3, 130.7, 127.6 (q, J = 276.4 Hz), 122.1 (q, J = 322.2 Hz), 117.3, 32.0 (q, J = 29.3 Hz), 18.3 (d, J = 3.0 Hz); **^{19}F NMR (376 MHz, CD_3CN)** δ -67.0, -79.2; **ATR-FTIR (cm $^{-1}$):** 2978, 1459, 1327, 1255, 1173, 1129, 1029, 875; **HRMS m/z (ESI)** calculated for $C_{16}H_{12}D_2F_3S_2^+$ [M – O_3SCF_3]$^+$ 329.0609, found 329.0611.

The characterization data of the corresponding non-deuterated product are as follows:

5-(4,4,4-trifluorobutyl)-5*H*-thianthren-5-ium trifluoromethanesulfonate (1i)

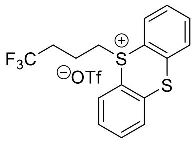

^{1}H NMR (400 MHz, CD_3CN) *δ* 8.14 (dd, J = 8.0, 1.2 Hz, 2H), 7.95 (dd, J = 8.0, 1.2 Hz, 2H), 7.83 (td, J = 8.0, 1.2 Hz, 2H), 7.71 (td, J = 8.0, 1.2 Hz, 2H), 3.71 (t, J = 8.0, 2H), 2.37 – 2.21 (m, 2H), 1.84 – 1.72 (m, 2H); **^{13}C NMR (101 MHz, CD_3CN)** *δ* 137.1, 135.9, 135.4, 131.4, 130.7, 127.7 (q, J = 276.7 Hz), 122.2 (q, J = 323.2 Hz), 117.4, 40.0, 32.0 (q, J = 30.3 Hz), 18.5 (d, J = 4.0 Hz); **^{19}F NMR (376 MHz, CD_3CN)** *δ* -67.0, -79.2;

ATR-FTIR (cm $^{-1}$): 2967, 1459, 1337, 1260, 1171, 1092, 1004, 876; **HRMS m/z (ESI)** calculated for $C_{16}H_{14}F_3S_2^+$ [M – O_3SCF_3]$^+$ 327.0484, found 327.0482.

5-(4-chlorobutyl-1,1-*d*$_2$)-5*H*-thianthren-5-ium trifluoromethanesulfonate (2j)

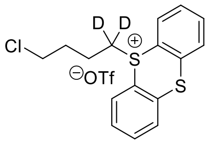

According to General procedure A, **2j** was prepared as a white solid (862.8 mg, 94% yield, 98% D): **^{1}H NMR (400 MHz, CDCl$_3$)** δ 8.20 (d, *J* = 7.8 Hz, 2H), 7.82 (d, *J* = 7.8 Hz, 2H), 7.75 (t, *J* = 7.5 Hz, 2H), 7.63 (t, *J* = 7.5 Hz, 2H), 3.44 (t, *J* = 5.8 Hz, 2H), 2.00 – 1.75 (m, 2H), 1.66 (t, *J* = 7.1 Hz, 2H); **^{13}C NMR (101 MHz, CDCl$_3$)** δ 135.6, 134.6, 134.4, 130.1, 129.8, 120.6 (q, *J* = 322.2 Hz), 116.6, 43.5, 29.9, 21.5; **^{19}F NMR (376 MHz, CDCl$_3$)** δ -78.1; **ATR-FTIR (cm $^{-1}$):** 1458, 1293, 1227, 1166, 1032, 765, 640; **HRMS m/z (ESI)** calculated for $C_{16}H_{14}D_2ClS_2^+$ [M – O_3SCF_3]$^+$ 309.0502, found 309.0497.

The characterization data of the corresponding non-deuterated product are as follows:

5-(4-chlorobutyl)-5*H*-thianthren-5-ium trifluoromethanesulfonate (1j)

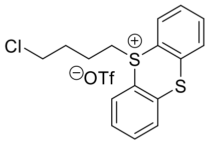

^{1}H NMR (400 MHz, CDCl$_3$) δ 8.18 (d, *J* = 7.8 Hz, 2H), 7.81 (d, *J* = 7.8 Hz, 2H), 7.73 (t, *J* = 7.5 Hz, 2H), 7.61 (t, *J* = 7.5 Hz, 2H), 3.81 – 3.72 (t, *J* = 7.6 Hz,, 2H), 3.42 (t, *J* = 6.1 Hz, 2H), 1.96 – 1.76 (m, 2H), 1.74 – 1.56 (m, 2H); **^{13}C NMR (101 MHz, CDCl$_3$)** δ 135.6, 134.5, 134.2, 130.1, 129.7, 120.6 (q, *J* = 322.2 Hz), 116.5, 43.4, 39.5, 29.8, 21.6; **^{19}F NMR (376 MHz, CDCl$_3$)** δ -78.1; **ATR-FTIR (cm $^{-1}$):** 1405, 1262, 1153, 1028, 756, 636; **HRMS m/z (ESI)** calculated for $C_{16}H_{16}ClS_2^+$ [M – O_3SCF_3]$^+$ 307.0376, found 307.0376.

5-(but-3-en-1-yl-1,1-*d*$_2$)-5*H*-thianthren-5-ium trifluoromethanesulfonate (2k)

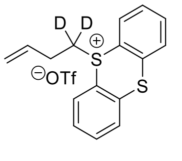

According to General procedure A, **2k** was prepared as a white solid (786.5 mg, 93% yield, 96% D): **^{1}H NMR (400 MHz, CDCl$_3$)** δ 8.22 (d, *J* = 7.9 Hz, 2H), 7.84 (d, *J* = 7.9 Hz, 2H), 7.79 – 7.71 (m, 2H), 7.69 – 7.59 (m, 2H), 5.70 (ddt, *J* = 16.9, 10.2, 6.7 Hz, 1H), 5.22 – 4.98 (m, 2H), 2.31 (d, *J* = 6.5 Hz, 2H); **^{13}C NMR (101 MHz, CDCl$_3$)** δ 135.8, 134.6, 134.5, 131.6, 130.1, 129.8, 120.7 (q, *J* = 320.5 Hz), 119.8, 116.7, 28.4; **^{19}F NMR (376 MHz, CDCl$_3$)** δ -78.1; **ATR-FTIR (cm $^{-1}$):** 3071, 2361, 1451, 1260, 1148, 1028, 928, 765, 634, 516, 456; **HRMS m/z (ESI)** calculated for $C_{16}H_{13}D_2S_2^+$ [M – O_3SCF_3]$^+$ 273.0735, found 273.0732.

The characterization data of the corresponding non-deuterated product are as follows:

5-(but-3-en-1-yl)-5*H*-thianthren-5-ium trifluoromethanesulfonate (1k)

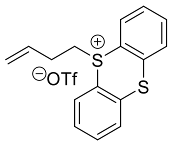

^{1}H NMR (400 MHz, CDCl$_3$) δ 8.23 – 8.15 (m, 2H), 7.86 – 7.80 (m, 2H), 7.78 – 7.71 (m, 2H), 7.68 – 7.58 (m, 2H), 5.68 (ddt, *J* = 16.9, 10.2, 6.6 Hz, 1H), 5.18 – 4.99 (m, 2H), 3.84 (t, *J* = 7.3 Hz, 2H), 2.30 (q, *J* = 7.0 Hz, 2H); **^{13}C NMR (101 MHz, CDCl$_3$)** δ 135.7, 134.5, 134.5, 131.6, 130.1, 129.7, 120.6 (q, *J* = 320.6 Hz), 119.8, 116.6, 39.4, 28.5; **^{19}F NMR (376 MHz, CDCl$_3$)** δ -78.1; **ATR-FTIR (cm $^{-1}$):** 3071, 2360, 1451, 1259, 1147, 1027, 929, 765, 633, 515, 457; **HRMS m/z (ESI)** calculated for $C_{16}H_{15}S_2^+$ [M – O_3SCF_3]$^+$ 271.0610, found 271.0612.

(*Z*)-5-(octadec-9-en-1-yl-1,1-*d*$_2$)-5*H*-thianthren-5-ium trifluoromethanesulfonate (2l)

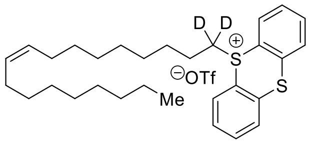

According to General procedure A, **2l** was prepared as a brown oil (1123.4 mg, 91% yield, 95% D): **^{1}H NMR (400 MHz, CDCl$_3$)** δ 8.29 (d, *J* = 7.8 Hz, 2H), 7.83 (d, *J* = 7.9 Hz, 2H), 7.75 (t, *J* = 7.6 Hz, 2H), 7.65 (t, *J* = 7.7 Hz, 2H), 5.39 – 5.18 (m, 2H), 2.04 – 1.83 (m, 2H), 1.59 – 1.44 (m, 2H), 1.40 – 1.06 (m, 24H), 0.85 (t, *J* = 6.7 Hz, 3H); **^{13}C NMR (101 MHz, CDCl$_3$)** δ 135.6, 134.7, 134.4, 130.0, 129.9, 129.9, 129.6, 120.8 (q, *J* = 320.7 Hz), 117.3, 31.8, 29.7, 29.5, 29.4, 29.2, 29.2, 28.9, 28.7, 27.7, 27.1, 27.0, 24.2, 22.6, 14.0; **^{19}F NMR (376 MHz, CDCl$_3$)** δ -78.1; **ATR-FTIR (cm $^{-1}$):** 3054, 2985, 2306, 1418, 1263, 895, 735, 701; **HRMS m/z (ESI)** calculated for $C_{30}H_{41}D_2S_2^+$ [M – O_3SCF_3]$^+$ 469.2926, found 469.2924.

The characterization data of the corresponding non-deuterated product are as follows:

(*Z*)-5-(octadec-9-en-1-yl)-5*H*-thianthren-5-ium trifluoromethanesulfonate (1l)

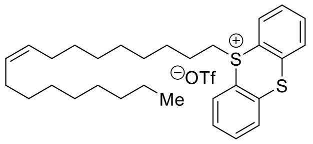

^{1}H NMR (400 MHz, CDCl$_3$) δ 8.32 (dd, *J* = 7.9, 1.1 Hz, 2H), 7.82 (dd, *J* = 7.9, 1.2 Hz, 2H), 7.75 (td, *J* = 7.7, 1.3 Hz, 2H), 7.66 (td, *J* = 7.7, 1.3 Hz, 2H), 5.34 – 5.26 (m, 2H), 3.80 – 3.73 (m, 2H), 1.98 – 1.94 (m, 2H), 1.53 (p, *J* = 7.6 Hz, 2H), 1.44 – 0.99 (m, 24H), 0.86 (t, *J* = 6.8 Hz, 3H); **^{13}C NMR (101 MHz, CDCl$_3$)** δ 135.6, 134.8, 134.4, 130.0, 129.9, 129.6, 120.8 (q, *J* = 321.2 Hz), 117.5, 40.4, 31.8,

29.7, 29.6, 29.5, 29.3, 29.0, 28.7, 27.8, 27.2, 27.1, 24.4, 22.6, 14.1; **^{19}F NMR (376 MHz, $CDCl_3$)** δ -78.2; **ATR-FTIR (cm $^{-1}$):** 2923, 2853, 1256, 1154, 1029, 637; **HRMS m/z (ESI)** calculated for $C_{30}H_{43}S_2^+$ [M – O_3SCF_3]$^+$ 467.2801, found 467.2791.

5-(dec-3-yn-1-yl-1,1-*d*$_2$)-5*H*-thianthren-5-ium trifluoromethanesulfonate (2m)

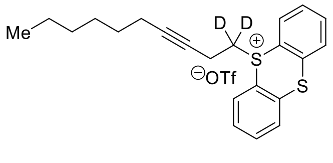

According to General procedure A, **2m** was prepared as a white solid (915.0 mg, 91% yield, 97% D): **^{1}H NMR (400 MHz, $CDCl_3$)** δ 8.35 – 8.25 (m, 2H), 7.84 (dd, *J* = 7.9, 1.1 Hz, 2H), 7.75 (td, *J* = 7.7, 1.3 Hz, 2H), 7.65 (t, *J* = 7.7 Hz, 2H), 2.62 (s, 2H), 2.03 – 1.96 (m, 2H), 1.43 – 1.32 (m, 2H), 1.31 – 1.18 (m, 6H), 0.87 (t, *J* = 6.6 Hz, 3H); **^{13}C NMR (101 MHz, $CDCl_3$)** δ 136.0, 134.6, 134.4, 130.1, 129.6, 120.6 (q, *J* = 320.9, 320.5 Hz), 116.6, 86.0, 73.0, 31.0, 28.3, 28.2, 22.2, 18.4, 15.2, 13.8; **^{19}F NMR (376 MHz, $CDCl_3$)** δ -78.1; **ATR-FTIR (cm $^{-1}$):** 3054, 2985, 2306, 1419, 1263, 895, 734, 701; **HRMS m/z (ESI)** calculated for $C_{22}H_{23}D_2S_2^+$ [M – O_3SCF_3]$^+$ 355.1518, found 355.1514.

The characterization data of the corresponding non-deuterated product are as follows:

5-(dec-3-yn-1-yl)-5*H*-thianthren-5-ium trifluoromethanesulfonate (1m)

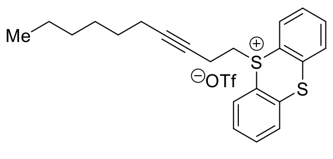

^{1}H NMR (400 MHz, $CDCl_3$) δ 8.20 (d, *J* = 7.9 Hz, 2H), 7.82 (d, *J* = 7.9 Hz, 2H), 7.73 (t, *J* = 7.7 Hz, 2H), 7.61 (t, *J* = 7.6 Hz, 2H), 3.84 (t, *J* = 6.4 Hz, 2H), 2.54 (t, *J* = 6.0 Hz, 2H), 2.01 – 1.90 (m, 2H), 1.34 (m, 2H), 1.27 – 1.13 (m, 6H), 0.81 (t, *J* = 6.8 Hz, 3H); **^{13}C NMR (101 MHz, $CDCl_3$)** δ 136.0, 134.6, 134.4, 130.1, 129.6, 120.6 (q, *J* = 322.2 Hz), 116.6, 86.0, 73.0, 40.1, 31.0, 28.3, 28.2, 22.3, 18.4, 15.3, 13.8; **^{19}F NMR (376 MHz, $CDCl_3$)** δ -78.1; **ATR-FTIR (cm $^{-1}$):** 2930, 1450, 1253, 1028, 761, 636; **HRMS m/z (ESI)** calculated for $C_{22}H_{25}S_2^+$ [M – O_3SCF_3]$^+$ 353.1392, found 353.1393.

5-(pent-4-yn-1-yl-1,1,5-*d*$_3$)-5*H*-thianthren-5-ium trifluoromethanesulfonate (2n)

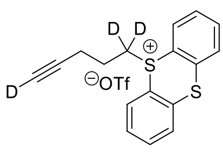

According to General procedure A, **2n** was prepared as a white solid (792.5 mg, 91% yield, 97% D): **^{1}H NMR (400 MHz, $CDCl_3$)** δ 8.22 (d, *J* = 7.8 Hz,

2H), 7.84 (d, J = 7.7 Hz, 2H), 7.75 (t, J = 7.4 Hz, 2H), 7.63 (t, J = 7.4 Hz, 2H), 2.28 (t, J = 6.7 Hz, 2H), 1.74 (t, J = 6.6 Hz, 2H); **^{13}C NMR (101 MHz, $CDCl_3$)** δ 135.7, 134.6, 134.3, 130.1, 129.8, 120.6 (q, J = 320.7 Hz), 116.6, 80.2, 22.8, 16.7; **^{19}F NMR (376 MHz, $CDCl_3$)** δ -78.1; **ATR-FTIR (cm $^{-1}$):** 2924, 1459, 1263, 1159, 1035, 872, 764, 639; **HRMS m/z (ESI)** calculated for $C_{17}H_{12}D_3S_2^+$ $[M – O_3SCF_3]^+$ 286.0798, found 286.0796.

The characterization data of the corresponding non-deuterated product are as follows:

5-(pent-4-yn-1-yl)-5*H*-thianthren-5-ium trifluoromethanesulfonate (1n)

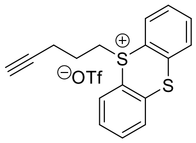

^{1}H NMR (400 MHz, $CDCl_3$) δ 8.25 (d, J = 7.8 Hz, 2H), 7.85 (d, J = 7.7 Hz, 2H), 7.76 (t, J = 7.3 Hz, 2H), 7.65 (t, J = 7.3 Hz, 2H), 3.91 – 3.82 (m, 2H), 2.30 (td, J = 6.6, 2.4 Hz, 2H), 1.96 (t, J = 2.5 Hz, 1H), 1.85 – 1.71 (m, 2H); **^{13}C NMR (101 MHz, $CDCl_3$)** δ 135.7, 134.6, 134.5, 130.1, 129.9, 120.7 (q, J = 320.5 Hz), 116.8, 80.7, 70.9, 39.2, 23.1, 16.9; **^{19}F NMR (376 MHz, $CDCl_3$)** δ -78.1; **ATR-FTIR (cm $^{-1}$):** 2945, 1456, 1283, 1157, 1032, 968, 877, 763; **HRMS m/z (ESI)** calculated for $C_{17}H_{15}S_2^+$ $[M – O_3SCF_3]^+$ 283.0610, found 283.0612.

5-((*R*)-4-((3*R*,5*S*,8*R*,9*S*,10*S*,13*R*,14*S*,17*R*)-3-methoxy-10,13-dimethylhexadecahydro-1*H*-cyclopenta[*a*]phenanthren-17-yl)pentyl-1,1-*d*$_2$)-5*H*-thianthren-5-ium trifluoromethanesulfonate (2o)

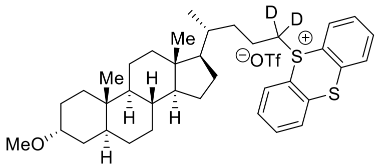

According to General procedure A, **2o** was prepared as a white solid (1279.9 mg, 88% yield, 97% D): **^{1}H NMR (400 MHz, CD_3CN)** δ 8.12 (d, J = 7.9 Hz, 2H), 7.97 (d, J = 7.9 Hz, 2H), 7.85 (t, J = 7.2 Hz, 2H), 7.73 (t, J = 7.7 Hz, 2H), 3.28 (s, 3H), 3.19 – 3.08 (m, 1H), 1.96 – 1.84 (m, 2H), 1.84 – 1.65 (m, 3H), 1.65 – 1.49 (m, 3H), 1.49 – 1.30 (m, 8H), 1.30 – 0.94 (m, 12H), 0.93 (s, 3H), 0.81 (d, J = 6.5 Hz, 3H), 0.63 (s, 3H); **^{13}C NMR(101 MHz, CD_3CN)** δ 136.8, 135.6, 131.2, 130.6, 122.1 (q, J = 321.1 Hz), 118.3, 117.7, 81.1, 57.2, 56.7, 55.6, 43.5, 42.9, 41.2, 40.8, 36.7, 36.1, 36.0, 35.6, 34.5, 33.6, 28.9, 28.1, 27.6, 27.2, 24.9, 23.9, 21.9, 21.6, 18.7, 12.4; **^{19}F NMR (376 MHz, CD_3CN)** δ -79.3; **ATR-FTIR (cm $^{-1}$):** 2927, 2862, 2361, 1452, 1260, 1154, 1030, 763, 636, 516, 460; **HRMS m/z (ESI)** calculated for $C_{37}H_{49}D_2OS_2^+$ $[M – O_3SCF_3]^+$ 577.3501, found 577.3501.

The characterization data of the corresponding non-deuterated product are as follows:

5-((*R*)-4-((3*R*,5*S*,8*R*,9*S*,10*S*,13*R*,14*S*,17*R*)-3-methoxy-10,13-dimethylhexadecahydro-1*H*-cyclopenta[*a*]phenanthren-17-yl)pentyl)-5*H*-thianthren-5-ium trifluoromethanesulfonate (1o)

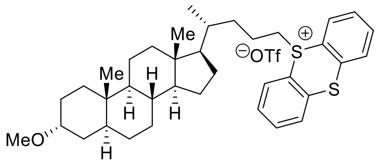

^{1}H NMR (400 MHz, CD_3CN) δ 8.10 (d, *J* = 7.8 Hz, 2H), 7.92 (d, *J* = 7.8 Hz, 2H), 7.81 (t, *J* = 7.5 Hz, 2H), 7.69 (t, *J* = 7.6 Hz, 2H), 3.76 – 3.63 (m, 1H), 3.61 – 3.51 (m, 1H), 3.24 (s, 3H), 3.16 – 3.01 (m, 1H), 1.93 – 1.79 (m, 2H), 1.78 – 1.61 (m, 3H), 1.61 – 1.48 (m, 3H), 1.48 – 1.26 (m, 8H), 1.25 – 0.92 (m, 12H), 0.88 (s, 3H), 0.77 (d, *J* = 6.4 Hz, 3H), 0.58 (s, 3H); **^{13}C NMR(101 MHz, CD_3CN)** δ 136.8, 135.6, 135.2, 135.2, 131.2, 130.6, 122.1 (q, *J* = 321.1 Hz), 117.8, 81.1, 57.2, 56.7, 55.6, 43.5, 42.9, 41.8, 41.2, 40.8, 36.7, 36.1, 36.0, 35.6, 34.5, 33.6, 28.9, 28.1, 27.6, 27.2, 24.9, 23.9, 22.1, 21.6, 18.7, 12.4; **^{19}F NMR (376 MHz, CD_3CN)** δ -79.3; **ATR-FTIR (cm $^{-1}$):** 2926, 2861, 2361, 1453, 1261, 1154, 1030, 761, 636, 516, 461; **HRMS m/z (ESI)** calculated for $C_{37}H_{51}OS_2^+$ [M – O_3SCF_3]$^+$ 575.3376, found 575.3378.

5-(3-(((8*R*,9*S*,13*S*,14*S*)-13-methyl-17-oxo-7,8,9,11,12,13,14,15,16,17-decahydro-6*H*-cyclopenta[*a*]phenanthren-3-yl)oxy)propyl-1,1-*d*$_2$)-5*H*-thianthren-5-ium trifluoromethanesulfonate (2p)

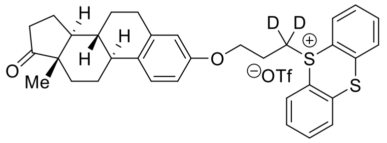

According to General procedure A, **2p** was prepared as a pale yellow solid (1222.0 mg, 90% yield, 97% D): **^{1}H NMR (400 MHz, $CDCl_3$)** δ8.29 (dd, *J* = 7.9, 0.9 Hz, 2H), 7.84 (dd, *J* = 7.9, 1.0 Hz, 2H), 7.75 (td, *J* = 7.7, 1.2 Hz, 2H), 7.66 (td, *J* = 7.7, 1.2 Hz, 2H), 7.14 (d, *J* = 8.6 Hz, 1H), 6.62 (dd, *J* = 8.5, 2.6 Hz, 1H), 6.57 (d, *J* = 2.4 Hz, 1H), 4.03 (t, *J* = 5.6 Hz, 2H), 2.90 – 2.79 (m, 2H), 2.49 (dd, *J* = 18.7, 8.7 Hz, 1H), 2.40 – 2.31 (m, 1H), 2.26 – 1.87 (m, 7H), 1.69 – 1.32 (m, 6H), 0.89 (s, 3H); **^{13}C NMR(101 MHz, $CDCl_3$)** δ 155.8, 137.9, 135.7, 134.7, 134.4, 132.8, 130.0, 129.8, 126.3, 120.7 (q, *J* = 320.7 Hz), 117.1, 114.5, 112.3, 65.3, 50.3, 47.8, 43.9, 38.2, 35.8, 31.5, 29.4, 26.4, 25.8, 24.3, 21.4, 13.8; **^{19}F NMR (376 MHz, $CDCl_3$)** δ -78.1; **ATR-FTIR (cm $^{-1}$):** 2927, 1731, 1451, 1254, 1151, 1029, 757, 636, 516; **HRMS m/z (ESI)** calculated for $C_{33}H_{33}D_2O_2S_2^+$ [M – O_3SCF_3]$^+$ 529.2199, found 529.2200.

The characterization data of the corresponding non-deuterated product are as follows:

5-(3-(((8*R*,9*S*,13*S*,14*S*)-13-methyl-17-oxo-7,8,9,11,12,13,14,15,16,17-decahydro-6*H*-cyclopenta[*a*]phenanthren-3-yl)oxy)propyl)-5*H*-thianthren-5-ium trifluoromethanesulfonate (1p)

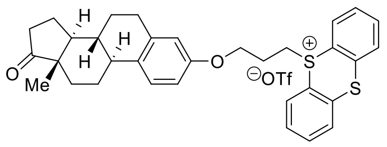

^{1}H NMR (400 MHz, CDCl$_3$) δ 8.15 (d, *J* = 7.4 Hz, 2H), 7.77 (d, *J* = 7.3 Hz, 2H), 7.73 – 7.64 (m, 2H), 7.62 – 7.52 (m, 2H), 7.04 (d, *J* = 8.7 Hz, 1H), 6.56 (dd, *J* = 8.5, 2.2 Hz, 1H), 6.50 (d, *J* = 1.9 Hz, 1H), 3.95 (t, *J* = 5.5 Hz, 2H), 3.90 (t, *J* = 7.1 Hz, 2H), 2.84 – 2.68 (m, 2H), 2.38 (dd, *J* = 18.3, 8.7 Hz, 1H), 2.23 (d, *J* = 5.4 Hz, 1H), 2.11 – 1.86 (m, 6H), 1.80 (d, *J* = 7.0 Hz, 1H), 1.58 – 1.21 (m, 6H), 0.78 (s, 3H); **^{13}C NMR(101 MHz, CDCl$_3$)** δ 155.8, 137.9, 135.7, 134.7, 134.4, 132.8, 130.0, 129.8, 126.3, 120.7 (q, *J* = 320.6 Hz), 117.2, 114.5, 112.3, 65.3, 50.3, 47.9, 43.9, 38.5, 38.2, 35.8, 31.5, 29.4, 26.4, 25.8, 24.5, 21.5, 13.8; **^{19}F NMR (376 MHz, CDCl$_3$)** δ -78.1; **ATR-FTIR (cm $^{-1}$):** 2928, 1728, 1452, 1244, 1163, 1029, 760, 638, 517; **HRMS m/z (ESI)** calculated for $C_{33}H_{35}O_2S_2^+$ [M – O_3SCF_3]$^+$ 527.2073, found 527.2078.

5-(3-(((8*S*,9*S*,10*R*,13*R*,14*S*,17*R*)-10,13-dimethyl-17-((*R*)-6-methylheptan-2-yl)-2,3,4,7,8,9,10,11,12,13,14,15,16,17-tetradecahydro-1*H*-cyclopenta[*a*]phenanthren-3-yl)oxy)propyl-1,1-*d*$_2$)-5*H*-thianthren-5-ium trifluoromethanesulfonate (2q)

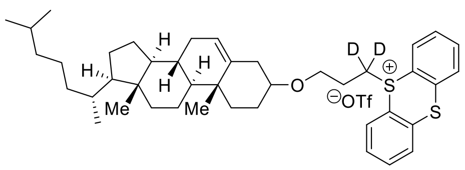

According to General procedure A, **2q** was prepared as a white solid (1495.7 mg, 94% yield, 98% D): **^{1}H NMR (400 MHz, CDCl$_3$)** δ 8.33 – 8.22 (m, 2H), 7.82 (d, *J* = 7.9 Hz, 2H), 7.74 (t, *J* = 7.6 Hz, 2H), 7.64 (t, *J* = 7.6 Hz, 2H), 5.30 (d, *J* = 4.8 Hz, 1H), 3.55 (t, *J* = 5.4 Hz, 2H), 3.19 – 3.05 (m, 1H), 2.29 (dd, *J* = 13.0, 2.5 Hz, 1H), 2.10 (t, *J* = 11.3 Hz, 1H), 2.01 – 1.70 (m, 7H), 1.61 – 1.01 (m, 18H), 1.01 – 0.93 (m, 5H), 0.93 – 0.86 (m, 4H), 0.84 (dd, *J* = 6.6, 1.4 Hz, 6H), 0.64 (s, 3H); **^{13}C NMR(101 MHz, CDCl$_3$)** δ 140.2, 135.7, 135.6, 134.9, 134.3, 130.0, 129.7, 121.9, 120.7 (d, *J* = 320.7 Hz), 117.4, 117.3, 79.5, 65.2, 56.6, 56.0, 50.0, 42.2, 39.6, 39.4, 38.9, 36.9, 36.7, 36.1, 35.7, 31.8, 31.8, 28.3, 28.1, 27.9, 24.9, 24.2, 23.7, 22.7, 22.5, 20.9, 19.3, 18.6, 11.8; **^{19}F NMR (376 MHz, CDCl$_3$)** δ -78.1; **ATR-FTIR (cm $^{-1}$):** 2934, 2897, 2867, 2361, 1455, 1255, 1152, 1028, 760, 637, 516, 457; **HRMS m/z (ESI)** calculated for $C_{42}H_{57}D_2OS_2^+$ [M – O_3SCF_3]$^+$ 645.4127, found 645.4129.

The characterization data of the corresponding non-deuterated product are as follows:

5-(3-(((8*S*,9*S*,10*R*,13*R*,14*S*,17*R*)-10,13-dimethyl-17-((*R*)-6-methylheptan-2-yl)-2,3,4,7,8,9,10,11,12,13,14,15,16,17-tetradecahydro-1*H*-cyclopenta[*a*]phenanthren-3-yl)oxy)propyl)-5*H*-thianthren-5-ium trifluoromethanesulfonate (1q)

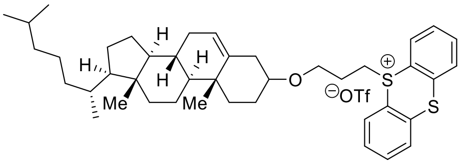

^{1}H NMR (400 MHz, $CDCl_3$) δ 8.27 (ddd, *J* = 7.6, 5.1, 0.9 Hz, 2H), 7.82 (d, *J* = 7.9 Hz, 2H), 7.74 (t, *J* = 7.7 Hz, 2H), 7.64 (t, *J* = 7.5 Hz, 2H), 5.30 (d, *J* = 5.0 Hz, 1H), 3.89 (t, *J* = 7.0 Hz, 2H), 3.55 (t, *J* = 5.5 Hz, 2H), 3.19 – 3.05 (m, 1H), 2.28 (dd, *J* = 13.0, 2.5 Hz, 1H), 2.17 – 2.03 (m, 1H), 2.01 – 1.74 (m, 7H), 1.60 – 0.99 (m, 18H), 0.99 – 0.93 (m, 5H), 0.89 (d, *J* = 6.5 Hz, 4H), 0.84 (dd, *J* = 6.6, 1.6 Hz, 6H), 0.64 (s, 3H); **^{13}C NMR(101 MHz, $CDCl_3$)** δ 140.2, 135.7, 135.6, 134.9, 134.3, 130.0, 129.7, 121.9, 120.8 (d, *J* = 320.6 Hz), 117.4, 117.3, 79.5, 65.3, 56.6, 56.0, 50.0, 42.2, 39.6, 39.4, 39.0, 38.9, 36.9, 36.7, 36.1, 35.7, 31.8, 31.8, 28.3, 28.1, 27.9, 25.1, 24.2, 23.7, 22.7, 22.5, 20.9, 19.3, 18.6, 11.8; **^{19}F NMR (376 MHz, $CDCl_3$)** δ -78.0; **ATR-FTIR (cm^{-1}):** 2932, 2897, 2867, 2361, 1455, 1253, 1152, 1028, 758, 637, 516, 457; **HRMS m/z (ESI)** calculated for $C_{42}H_{59}OS_2^+$ [M – O_3SCF_3]$^+$ 643.4002, found 643.4010.

(b) Synthesis of 2a'

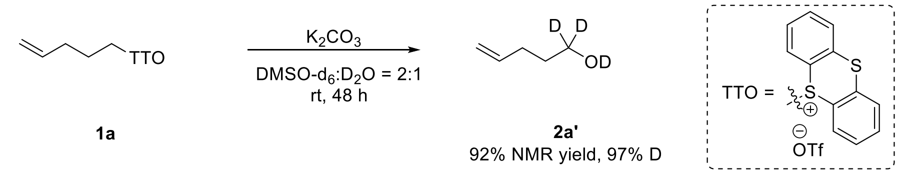

To a flame-dried 10 mL flask with a stir bar was added the **1a** (173.6 mg, 0.4 mmol, 1.0 equiv.), K_2CO_3 (110.4 mg, 0.8 mmol, 2.0 equiv.), DMSO-d_6 (1 mL) and D_2O (0.5 mL). The reaction was stirred at room temperature. After 48 h, CH_2Br_2 (69.5 mg, 0.4 mmol, 1.0 equiv.) was added to the reaction mixture. The reaction was stirred for 5 min, then filtered. The yield of **2a'** was determined by crude ^{1}H NMR using CH_2Br_2 as internal standard (Supplementary Figure 2).

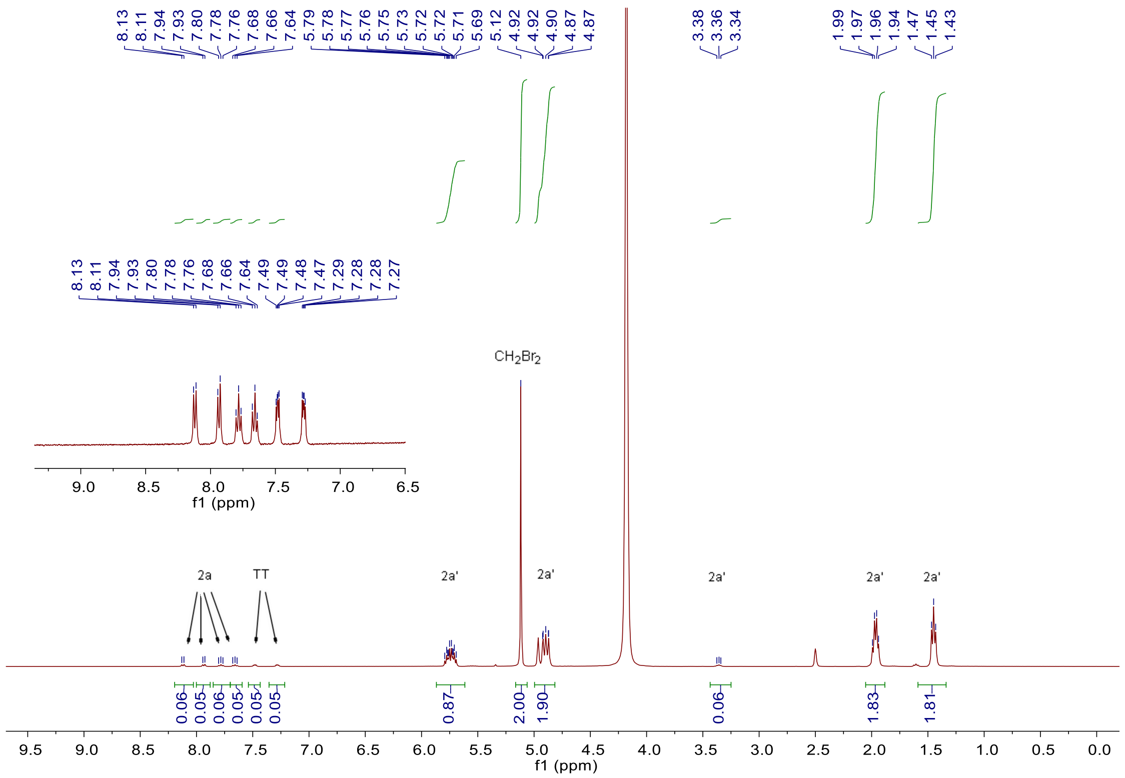

Supplementary Figure 2. Crude ^{1}H NMR (400 MHz, DMSO-d_6) of the reaction

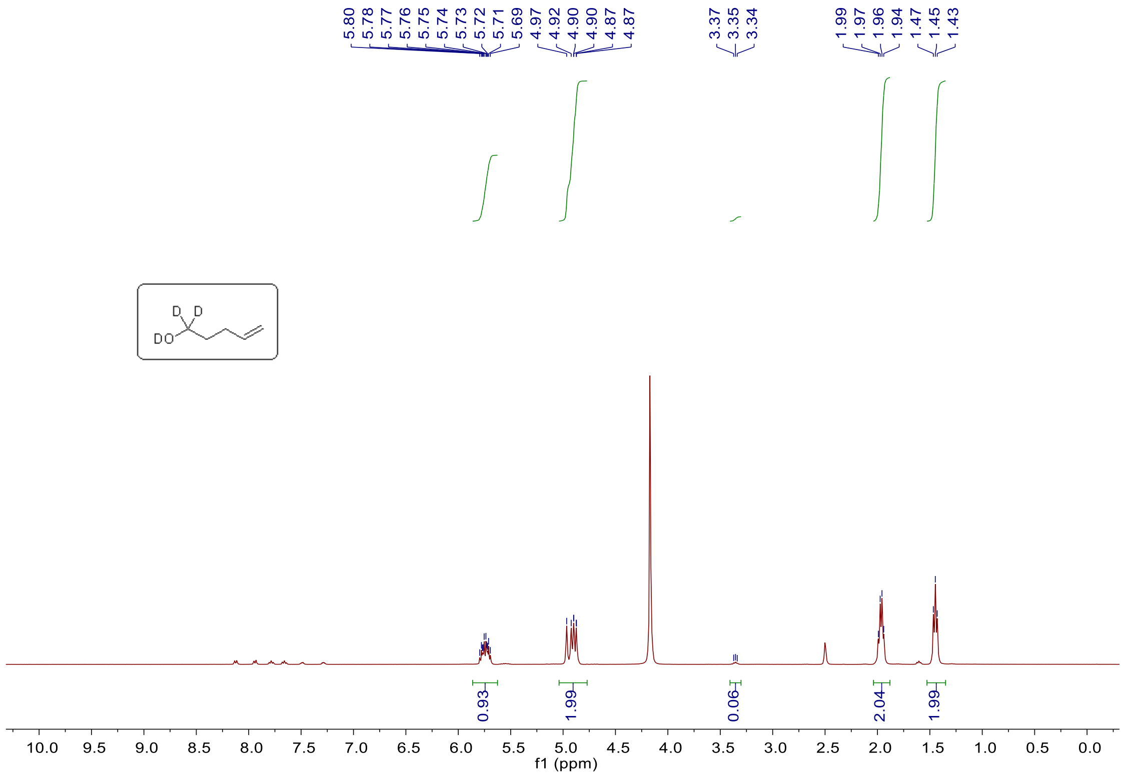

Supplementary Figure 3. ^{1}H NMR (400 MHz, DMSO-d_6) of **2a'**

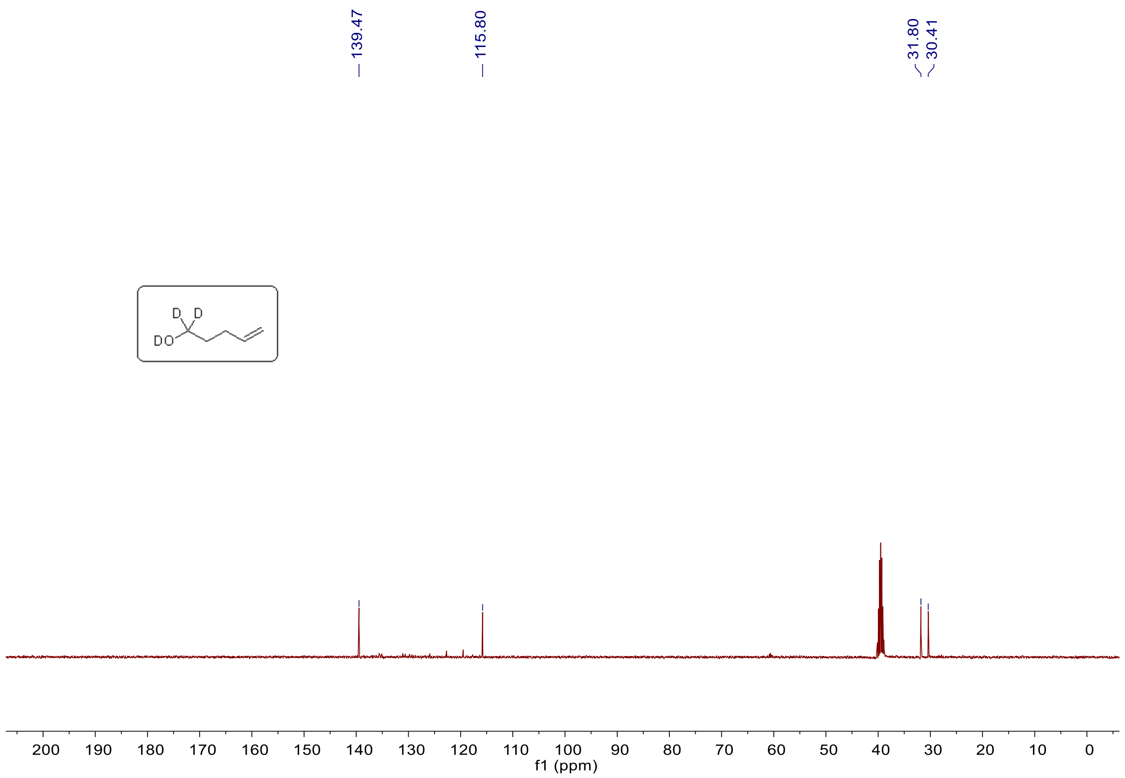

Supplementary Figure 4. ^{13}C NMR (400 MHz, DMSO-d_6) of **2a'**

(c) Reaction of TT salt 2o with various inorganic salts for the production of isotopically labelled compounds

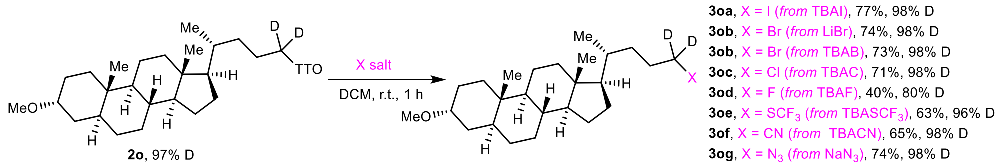

General procedure B: To a flame-dried 10 mL flask with a stir bar was added the **2o** (72.7 mg, 0.1 mmol, 1.0 equiv.), x salt (0.2 mmol, 2.0 equiv.) and DCM (2 mL). The reaction was stirred at room temperature for 2 minutes. The crude product was concentrated in vacuo and purified by flash chromatography.

(3*R*,5*S*,8*R*,9*S*,10*S*,13*R*,14*S*,17*R*)-17-((*R*)-5-iodopentan-2-yl-5,5-d_2)-3-methoxy-10,13-dimethylhexadecahydro-1*H*-cyclopenta[*a*]phenanthrene (3oa)

According to General procedure B, the crude product was purified by flash column chromatography

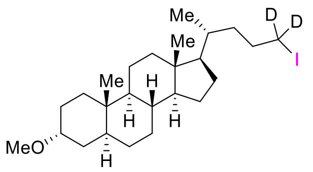

on silica gel (PE : EA = 70:1) to afford **3oa** (36.0 mg, 74% yield, 98% D) as a white solid: **^{1}H NMR (500 MHz, CDCl$_3$)** δ 3.34 (s, 3H), 3.21 – 3.13 (m, 1H), 1.98 – 1.69 (m, 8H), 1.64 – 1.56 (m, 2H), 1.50 – 1.35 (m, 7H), 1.29 – 1.03 (m, 11H), 0.95 – 0.92 (m, 6H), 0.65 (s, 3H); **^{13}C NMR (126 MHz, CDCl$_3$)** δ 80.4, 56.5, 56.1, 55.6, 42.7, 42.1, 40.4, 40.2, 36.8, 35.9, 35.3, 35.1, 34.9, 32.8, 30.2, 28.3, 27.4, 26.8, 26.4, 24.2, 23.4, 20.8, 18.7, 12.1; **ATR-FTIR (cm^{-1}):** 2931, 1446, 1374, 1263, 1172, 1096, 984, 839, 735; **HRMS m/z (ESI)** calculated for $C_{25}H_{41}D_2IONa^+$ $[M+Na]^+$ 511.2376, found 511.2381.

The characterization data of the corresponding non-deuterated product are as follows:

(3*R*,5*S*,8*R*,9*S*,10*S*,13*R*,14*S*,17*R*)-17-((*R*)-5-iodopentan-2-yl)-3-methoxy-10,13-dimethylhexadecahydro-1*H*-cyclopenta[*a*]phenanthrene (3oa')

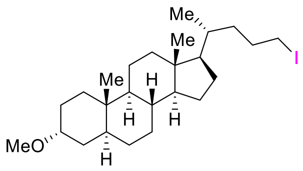

^{1}H NMR (500 MHz, CDCl$_3$) δ 3.34 (s, 3H), 3.22 – 3.08 (m, 3H), 1.97 – 1.66 (m, 8H), 1.62 – 1.53 (m, 2H), 1.48 – 1.31 (m, 7H), 1.29 – 0.99 (m, 11H), 0.93 – 0.89 (m, 6H), 0.63 (s, 3H); **^{13}C NMR (126 MHz, CDCl$_3$)** δ 80.4, 56.4, 56.0, 55.5, 42.7, 42.0, 40.3, 40.1, 36.8, 35.8, 35.3, 35.1, 34.9, 32.7, 30.4, 28.3, 27.3, 26.8, 26.4, 24.2, 23.4, 20.8, 18.7, 12.0; **ATR-FTIR (cm^{-1}):** 2930, 2856, 1446, 1373, 1231, 1171, 1096,782; **HRMS m/z (ESI)** calculated for $C_{25}H_{43}IONa^+$ $[M+Na]^+$ 509.2251, found 509.2255.

(3*R*,5*S*,8*R*,9*S*,10*S*,13*R*,14*S*,17*R*)-17-((*R*)-5-bromopentan-2-yl-5,5-*d*$_2$)-3-methoxy-10,13-dimethylhexadecahydro-1*H*-cyclopenta[*a*]phenanthrene (3ob)

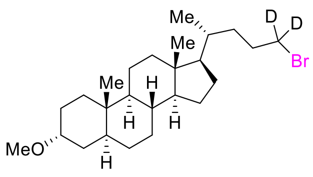

According to General procedure B, the crude product was purified by flash column chromatography on silica gel (PE : EA = 70:1) to afford **3ob** (31.6 mg, 72% yield, 98% D) as a white solid: **^{1}H NMR (500 MHz, CDCl$_3$)** δ 3.34 (s, 3H), 3.19 – 3.11 (m, 1H), 1.97 – 1.67 (m, 8H), 1.61 – 1.46 (m, 3H), 1.43 – 1.30 (m, 6H), 1.28 – 1.00 (m, 11H), 0.92 – 0.89 (m, 6H), 0.63 (s, 3H); **^{13}C NMR (126 MHz, CDCl$_3$)** δ 80.4, 56.4, 56.0, 55.5, 42.7, 42.0, 40.3, 40.1, 35.8, 35.3, 35.2, 34.9, 34.4, 32.8, 29.4, 28.3, 27.3, 26.8, 26.4, 24.2, 23.4, 20.8, 18.6, 12.0; **ATR-FTIR (cm^{-1}):** 2929, 1449, 1374, 1264, 1173, 1093, 993, 741; **HRMS m/z (ESI)** calculated for $C_{25}H_{41}D_2BrONa^+$ $[M+Na]^+$ 463.2515, found 463.2513.

The characterization data of the corresponding non-deuterated product are as follows:

(3*R*,5*S*,8*R*,9*S*,10*S*,13*R*,14*S*,17*R*)-17-((*R*)-5-bromopentan-2-yl)-3-methoxy-10,13-dimethylhexadecahydro-1*H*-cyclopenta[*a*]phenanthrene (3ob')

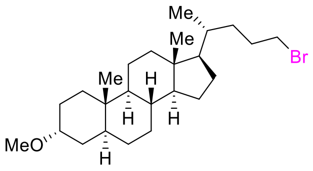

^{1}H NMR (500 MHz, $CDCl_3$) δ 3.42 – 3.32 (m, 5H), 3.19 – 3.11 (m, 1H), 1.98 – 1.69 (m, 8H), 1.60 – 1.46 (m, 3H), 1.43 – 1.32 (m, 6H), 1.28 – 1.00 (m, 11H), 0.93 – 0.90 (m, 6H), 0.63 (s, 3H); **^{13}C NMR (126 MHz, $CDCl_3$)** δ 80.4, 56.4, 56.0, 55.5, 42.7, 42.0, 40.3, 40.1, 35.8, 35.3, 35.2, 34.9, 34.5, 34.5, 32.8, 29.6, 28.3, 27.3, 26.8, 26.4, 24.2, 23.4, 20.8, 18.6, 12.0; **ATR-FTIR (cm $^{-1}$):** 2930, 1447, 1374, 1299, 1171, 1094, 936, 741; **HRMS m/z (ESI)** calculated for $C_{25}H_{43}BrONa^{+}$ $[M+Na]^{+}$ 461.2389, found 461.2390.

(3*R*,5*S*,8*R*,9*S*,10*S*,13*R*,14*S*,17*R*)-17-((*R*)-5-chloropentan-2-yl-5,5-*d*$_2$)-3-methoxy-10,13-dimethylhexadecahydro-1*H*-cyclopenta[*a*]phenanthrene (3oc)

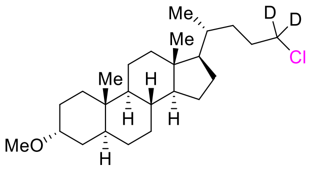

According to General procedure B, the crude product was purified by flash column chromatography on silica gel (PE : EA = 70:1) to afford **3oc** (28.1 mg, 71% yield, 98% D) as a white solid: **^{1}H NMR (500 MHz, $CDCl_3$)** δ 3.35 (s, 3H), 3.19 – 3.12 (m, 1H), 1.98 – 1.92 (m, 1H), 1.88 – 1.72 (m, 5H), 1.70 – 1.48 (m, 5H), 1.43 – 1.32 (m, 6H), 1.30 – 0.98 (m, 11H), 0.92 – 0.89 (m, 6H), 0.64 (s, 3H); **^{13}C NMR (126 MHz, $CDCl_3$)** δ 80.4, 56.5, 56.0, 55.5, 42.7, 42.0, 40.3, 40.2, 35.8, 35.3, 35.3, 34.9, 33.1, 32.8, 29.2, 28.3, 27.3, 26.8, 26.4, 24.2, 23.4, 20.8, 18.6, 12.0; **ATR-FTIR (cm $^{-1}$):** 2932, 1449, 1373, 1264, 1173, 1095, 1005, 936; **HRMS m/z (ESI)** calculated for $C_{25}H_{41}D_2ClONa^{+}$ $[M+Na]^{+}$ 419.3020, found 419.3016.

The characterization data of the corresponding non-deuterated product are as follows:

(3*R*,5*S*,8*R*,9*S*,10*S*,13*R*,14*S*,17*R*)-17-((*R*)-5-chloropentan-2-yl)-3-methoxy-10,13-dimethylhexadecahydro-1*H*-cyclopenta[*a*]phenanthrene (3oc')

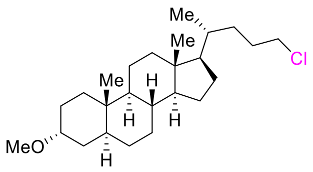

^{1}H NMR (500 MHz, $CDCl_3$) δ 3.56 – 3.44 (m, 2H), 3.34 (s, 3H), 3.19 – 3.11 (m, 1H), 1.97 – 1.92 (m, 1H), 1.87 – 1.72 (m, 5H), 1.70 – 1.47 (m, 5H), 1.42 – 1.31 (m, 6H), 1.29 – 1.01 (m, 11H), 0.93 – 0.90 (m, 6H), 0.63 (s, 3H); **^{13}C NMR (126 MHz, $CDCl_3$)** δ 80.39, 56.4, 56.0, 55.5, 45.7, 42.7, 42.0, 40.3, 40.2, 35.8, 35.3, 35.3, 34.9, 33.2, 32.8, 29.4, 28.2, 27.3, 26.8, 26.4, 24.2, 23.4, 20.8, 18.6, 12.0; **ATR-FTIR (cm $^{-1}$):** 2930, 1446, 1374, 1173, 1098, 937, 757, 719; **HRMS m/z (ESI)** calculated for $C_{25}H_{43}ClONa^{+}$ $[M+Na]^{+}$ 417.2895, found 417.2893.

(3*R*,5*S*,8*R*,9*S*,10*S*,13*R*,14*S*,17*R*)-17-((*R*)-5-fluoropentan-2-yl-5,5-*d*$_2$)-3-methoxy-10,13-dimethylhexadecahydro-1*H*-cyclopenta[*a*]phenanthrene (3od)

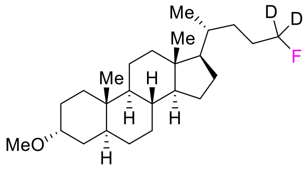

According to General procedure B, the crude product was purified by flash column chromatography on silica gel (PE : EA = 70:1) to afford **3od** (14.9 mg, 45% yield, 80% D) as a white solid: **^{1}H NMR (500 MHz, $CDCl_3$)** δ 3.35 (s, 3H), 3.19 – 3.11 (m, 1H), 1.98 – 1.93 (m, 1H), 1.89 – 1.67 (m, 6H), 1.62 – 1.47 (m, 5H), 1.43 – 1.35 (m, 5H), 1.29 – 1.18 (m, 5H), 1.16 – 1.00 (m, 6H), 0.93 – 0.91 (m, 6H), 0.64 (s, 3H); **^{13}C NMR (126 MHz, $CDCl_3$)** δ 80.4, 56.5, 56.1, 55.5, 42.7, 42.0, 40.0, 40.2, 35.8, 35.4, 35.3, 34.9, 32.8, 31.1 (d, *J* = 5.2 Hz), 28.2, 27.3, 27.2 – 26.9 (m), 26.8, 26.4, 24.2, 23.4, 20.8, 18.5, 12.0; **^{19}F NMR (471 MHz, $CDCl_3$)** δ -217.3, -218.0 (m), -218.6 (m); **ATR-FTIR (cm $^{-1}$):** 3052, 1447, 1374, 1264, 1095, 943, 736; **HRMS m/z (ESI)** calculated for $C_{25}H_{41}D_2FONa^{+}$ $[M+Na]^{+}$ 403.3316, found 403.3318.

The characterization data of the corresponding non-deuterated product are as follows:

(3*R*,5*S*,8*R*,9*S*,10*S*,13*R*,14*S*,17*R*)-17-((*R*)-5-fluoropentan-2-yl)-3-methoxy-10,13-dimethylhexadecahydro-1*H*-cyclopenta[*a*]phenanthrene (3od')

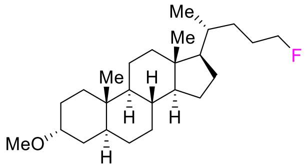

^{1}H NMR (400 MHz, $CDCl_3$) δ 4.47 (t, *J* = 6.3 Hz, 1H), 4.35 (t, *J* = 6.2 Hz, 1H), 3.35 (s, 3H), 3.21 – 3.10 (m, 1H), 1.98 – 1.92 (m, 1H), 1.88 – 1.69 (m, 6H), 1.61 – 1.45 (m, 5H), 1.42 – 1.34 (m, 5H), 1.32 – 1.17 (m, 5H), 1.15 – 1.02 (m, 6H), 0.94 – 0.90 (m, 6H), 0.64 (s, 3H); **^{13}C NMR (101 MHz, $CDCl_3$)** δ 84.7 (d, *J* = 164.3 Hz), 80.4, 56.5, 56.1, 55.5, 42.7, 42.0, 40.3, 40.2, 35.8, 35.4, 35.3, 34.9, 32.8, 31.1 (d, *J* = 5.2 Hz), 28.2, 27.3, 27.1 (d, *J* = 19.2 Hz), 26.8, 26.4, 24.2, 23.4, 20.8, 18.5, 12.0; **^{19}F**

NMR (471 MHz, $CDCl_3$) δ -217.3; **ATR-FTIR (cm $^{-1}$):** 3053, 1448, 1375, 1264, 1097, 907, 733; **HRMS m/z (ESI)** calculated for $C_{25}H_{43}FONa^+$ $[M+Na]^+$ 401.3190, found 401.3185.

((*R*)-4-((3*R*,5*R*,8*R*,9*S*,10*S*,13*R*,14*S*,17*R*)-3-methoxy-10,13-dimethylhexadecahydro-1*H*-cyclopenta[*a*]phenanthren-17-yl)pentyl-1,1-*d*$_2$)(trifluoromethyl)sulfane (3oe)

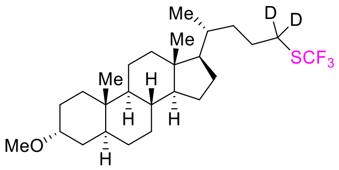

According to General procedure B, the crude product was purified by flash column chromatography on silica gel (PE : EA = 50:1) to afford **3oe** (29.2 mg, 63% yield, 98% D) as a colorless oil: **^{1}H NMR (400 MHz, $CDCl_3$)** δ 3.35 (s, 3H), 3.20 – 3.11 (m, 1H), 1.97 – 1.67 (m, 7H), 1.61 – 1.55 (m, 3H), 1.45 – 1.30 (m, 7H), 1.29 – 1.18 (m, 5H), 1.16 – 1.01 (m, 6H), 0.93 – 0.90 (m, 6H), 0.64 (s, 3H); **^{13}C NMR (101 MHz, $CDCl_3$)** δ 131.2 (q, *J* = 307.1 Hz), 80.4, 56.4, 56.0, 55.5, 42.7, 42.0, 40.3, 40.2, 35.8, 35.4, 35.3, 34.9, 34.8, 32.8, 28.3, 27.3, 26.8, 26.4, 25.9, 24.2, 23.4, 20.8, 18.5, 12.0; **^{19}F NMR (376 MHz, $CDCl_3$)** δ -41.2; **ATR-FTIR (cm $^{-1}$):** 3053, 1448, 1375, 1264, 1154, 1093, 1016, 734; **HRMS m/z (ESI)** calculated for $C_{26}H_{41}D_2F_3OSNa^+$ $[M+Na]^+$ 485.3004, found 485.3004.

The characterization data of the corresponding non-deuterated product are as follows:

((*R*)-4-((3*R*,5*R*,8*R*,9*S*,10*S*,13*R*,14*S*,17*R*)-3-methoxy-10,13-dimethylhexadecahydro-1*H*-cyclopenta[*a*]phenanthren-17-yl)pentyl-1,1-*d*$_2$)(trifluoromethyl)sulfane (3oe')

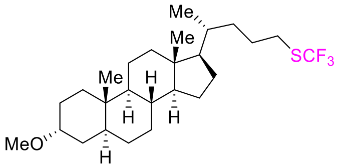

^{1}H NMR (400 MHz, $CDCl_3$) δ 3.35 (s, 3H), 3.20 – 3.11 (m, 1H), 2.91 – 2.76 (m, 2H), 1.98 – 1.67 (m, 7H), 1.61 – 1.55 (m, 3H), 1.46 – 1.31 (m, 7H), 1.29 – 1.16 (m, 5H), 1.15 – 1.00 (m, 6H), 0.93 – 0.90 (m, 6H), 0.64 (s, 3H); **^{13}C NMR (101 MHz, $CDCl_3$)** δ 131.2 (q, *J* = 305.5 Hz), 80.4, 56.4, 56.0, 55.5, 42.7, 42.0, 40.3, 40.2, 35.8, 35.4, 35.3, 34.9, 34.8, 32.8, 30.4, 28.3, 27.3, 26.8, 26.4, 26.1, 24.2, 23.4, 20.8, 18.5, 12.0; **^{19}F NMR (376 MHz, $CDCl_3$)** δ -41.2; **ATR-FTIR (cm $^{-1}$):** 2932, 1446, 1375, 1264, 1152, 1115; **HRMS m/z (ESI)** calculated for $C_{26}H_{43}F_3OSNa^+$ $[M+Na]^+$ 483.2879, found 483.2875.

(*R*)-5-((3*R*,5*S*,8*R*,9*S*,10*S*,13*R*,14*S*,17*R*)-3-methoxy-10,13-dimethylhexadecahydro-1*H*-cyclopenta[*a*]phenanthren-17-yl)hexanenitrile-2,2-*d*$_2$ (3of)

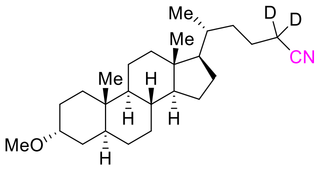

According to General procedure B, the crude product was purified by flash column chromatography on silica gel (PE : EA = 30:1) to afford **3of** (25.2 mg, 65% yield, 98% D) as a white solid: **^{1}H NMR (400 MHz, CDCl$_3$)** δ 3.35 (s, 3H), 3.20 – 3.11 (m, 1H), 1.97 – 1.92 (m, 1H), 1.88 – 1.66 (m, 6H), 1.64 – 1.49 (m, 5H), 1.43 – 1.35 (m, 5H), 1.28 – 1.14 (m, 6H), 1.13 – 1.00 (m, 5H), 0.93 – 0.90 (m, 6H), 0.64 (s, 3H); **^{13}C NMR (101 MHz, CDCl$_3$)** δ 119.9, 80.4, 56.4, 55.9, 55.5, 42.7, 42.0, 40.3, 40.2, 35.8, 35.3, 35.2, 34.9, 34.9, 32.8, 28.3, 27.3, 26.8, 26.4, 24.2, 23.4, 22.0, 20.8, 18.4, 12.0; **ATR-FTIR (cm $^{-1}$):** 2930, 2087, 1447, 1375, 1267, 1099, 756; **HRMS m/z (ESI)** calculated for $C_{26}H_{42}D_2NO^+$ [M+H]$^+$ 388.3543, found 388.3542.

The characterization data of the corresponding non-deuterated product are as follows:

(*R*)-5-((3*R*,5*S*,8*R*,9*S*,10*S*,13*R*,14*S*,17*R*)-3-methoxy-10,13-dimethylhexadecahydro-1*H*-cyclopenta[*a*]phenanthren-17-yl)hexanenitrile (3of')

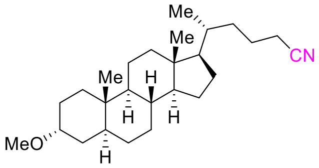

^{1}H NMR (400 MHz, CDCl$_3$) δ 3.35 (s, 3H), 3.20 – 3.10 (m, 1H), 2.35 – 2.25 (m, 2H), 1.97 – 1.91 (m, 1H), 1.87 – 1.66 (m, 6H), 1.62 – 1.51 (m, 5H), 1.42 – 1.34 (m, 5H), 1.28 – 1.18 (m, 6H), 1.13 – 1.01 (m, 5H), 0.93 – 0.90 (m, 6H), 0.64 (s, 3H); **^{13}C NMR (126 MHz, CDCl$_3$)** δ 119.9, 80.4, 56.4, 55.9, 55.5, 42.7, 42.0, 40.3, 40.1, 35.8, 35.3, 35.2, 35.0, 34.9, 32.7, 28.3, 27.3, 26.8, 26.4, 24.2, 23.4, 22.2, 20.8, 18.4, 17.5, 12.0; **ATR-FTIR (cm $^{-1}$):** 2938, 2243, 1467, 1443, 1375, 1169, 1094; **HRMS m/z (ESI)** calculated for $C_{26}H_{44}NO^+$ [M+H]$^+$ 386.3417, found 386.3410.

(3*R*,5*S*,8*R*,9*S*,10*S*,13*R*,14*S*,17*R*)-17-((*R*)-5-azidopentan-2-yl-5,5-*d*$_2$)-3-methoxy-10,13-dimethylhexadecahydro-1*H*-cyclopenta[*a*]phenanthrene (3og)

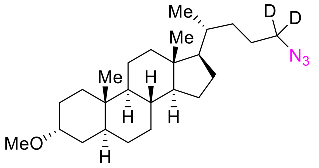

According to General procedure B, the crude product was purified by flash column chromatography on silica gel (PE : EA = 50:1) to afford **3og** (30.0 mg, 74% yield, 98% D) as a colorless oil: **^{1}H NMR (400 MHz, CDCl$_3$)** δ 3.35 (s, 3H), 3.20 – 3.10 (m, 1H), 1.95 (m, *J* = 12.4, 3.1 Hz, 1H), 1.88 – 1.73 (m, 4H), 1.72 – 1.53 (m, 5H), 1.45 – 1.33 (m, 7H), 1.30 – 1.16 (m, 5H), 1.15 – 1.01 (m, 6H), 0.93 – 0.90 (m, 6H), 0.64 (s, 3H); **^{13}C NMR (101 MHz, CDCl$_3$)** δ 80.4, 56.4, 56.0, 55.5, 42.7, 42.0, 40.3, 40.2, 35.8, 35.5, 35.3, 34.9, 32.8, 32.8, 28.3, 27.3, 26.8, 26.4, 25.3, 24.2, 23.4, 20.8, 18.6, 12.0; **ATR-**

FTIR (cm $^{-1}$): 2931, 2090, 1446, 1374, 1256, 1172, 1097, 738; **HRMS m/z (ESI)** calculated for $C_{25}H_{41}D_2N_3ONa^+$ $[M+Na]^+$ 426.3424, found 426.3422.

The characterization data of the corresponding non-deuterated product are as follows:

(3*R*,5*S*,8*R*,9*S*,10*S*,13*R*,14*S*,17*R*)-17-((*R*)-5-azidopentan-2-yl)-3-methoxy-10,13-dimethylhexadecahydro-1*H*-cyclopenta[*a*]phenanthrene (3og')

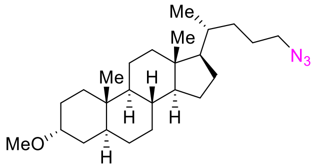

^{1}H NMR (400 MHz, $CDCl_3$) δ 3.35 (s, 3H), 3.25 – 3.04 (m, 3H), 1.91 – 1.85 (m, 1H), 1.81 – 1.66 (m, 4H), 1.64 – 1.47 (m, 5H), 1.41 – 1.28 (m, 7H), 1.24 – 1.11 (m, 5H), 1.08 – 0.95 (m, 6H), 0.88 – 0.83 (m, 7H), 0.64 (s, 3H); **^{13}C NMR (101 MHz, $CDCl_3$)** δ 80.4, 56.4, 56.0, 55.5, 52.0, 42.7, 42.0, 40.3, 40.2, 35.8, 35.5, 35.3, 34.9, 32.9, 32.7, 28.3, 27.3, 26.8, 26.4, 25.5, 24.2, 23.4, 20.8, 18.6, 12.0; **ATR-FTIR (cm $^{-1}$):** 2939, 2857, 2120, 1447, 1374, 1272, 1174, 1099; **HRMS m/z (ESI)** calculated for $C_{25}H_{43}N_3ONa^+$ $[M+Na]^+$ 424.3298, found 424.3292.

3. Reaction optimization

Supplementary Table 1: Optimization of the reaction conditions of aryl alkylation.

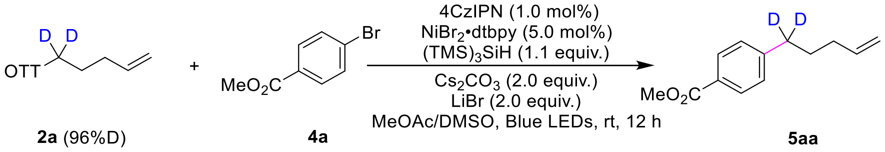

2a (96%D) **4a** **5aa**

Entry	Deviation	Yield of **5aa**
1	none	73%, 96% D
2	without 4CzIPN or in the dark	0
3	without $NiBr_2$•dtbpy	0
4	without LiBr	<5
5	without TMS_3SiH	0
6	without Cs_2CO_3	38%, 96% D
7	using MeOAc as solvent	69%, 96% D
8	using MeCN as solvent	23%, 96% D
9	using MTBE as solvent	55%, 96% D
10	using PhMe or DCM as solvent	trace
11	using TMS_3SiOH instead of TMS_3SiH	45%, 96% D

12	using DIPEA instead of Cs_2CO_3	0
13	using K_2CO_3 instead of Cs_2CO_3	71%, 96% D
14	using $[Ir(dF(CF_3)ppy)_2(dtbbpy)]PF_6$ instead of 4CzIPN	65%, 96% D

Standard conditions: **2a** (0.4 mmol), **4a** (0.2 mmol), 4CzIPN (1.0 mol%), $NiBr_2$•dtbpy (5.0 mol%), $(TMS)_3SiH$ (0.22 mmol), Cs_2CO_3 (0.4 mmol), LiBr (0.4 mmol), MeOAc (1.6 mL)/DMSO (0.4 mL), Blue LEDs, room temperature (rt), 12 h, isolated yields, deuterium incorporation was determined by ^{1}H NMR spectroscopy and/or HRMS.

4. Procedure for metallaphotoredox-catalyzed sp^{3}C-sp^{2}C and sp^{3}C-sp^{3}C coupling reaction

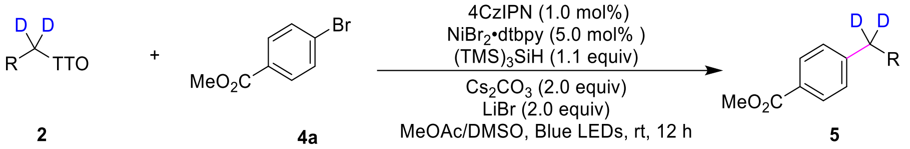

General procedure C: In a nitrogen-filled glove box, to an 8 mL oven-dried vial equipped with a stir bar was added **2** (0.4 mmol, 2 equiv.), **4a** (43.0 mg, 0.2 mmol, 1 equiv.), Cs_2CO_3 (130.3 mg, 0.4 mmol, 2 equiv.), LiBr (34.7 mg, 0.4 mmol, 2 equiv.), 4CzIPN (1.8 mg, 1 mol%) and $NiBr_2$•dtbpy (4.9 mg, 5 mol%). Then, anhydrous MeOAc (1.6 mL) and anhydrous DMSO (0.4 mL) were added via syringe, followed by addition of tris(trimethylsilyl)silane (68 µL, 0.22 mmol, 1.1 equiv.). The vial was sealed and removed from the glovebox. Subsequently, the reaction mixture was stirred and irradiated with a 40 W blue LED lamp for 12 hours. The final reaction mixture was diluted with EtOAc (60 mL) and saturated aqueous LiCl solution (20 mL). The organic layer was washed with brine (2 x 20 mL) and concentrated. Purification by flash column chromatography on silica gel to afford the aryl alkylation product.

methyl 4-(pent-4-en-1-yl-1,1-*d*$_2$)benzoate (5aa)

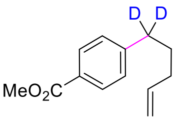

According to general procedure C, the crude product was purified by flash column chromatography on silica gel (PE : EA = 50:1) to afford **5aa** (30.0 mg, 73% yield, 96% D) as a colorless oil: **^{1}H NMR (400 MHz, $CDCl_3$)** δ 8.10 – 7.69 (m, 2H), 7.50 – 7.02 (m, 2H), 5.82 (ddt, J = 16.9, 10.2, 6.6 Hz, 1H), 5.20 – 4.75 (m, 2H), 3.90 (s, 1H), 2.41

– 1.95 (m, 2H), 1.72 (t, *J* = 7.4 Hz, 2H); **^{13}C NMR (101 MHz, $CDCl_3$)** δ 167.1, 147.9, 138.2, 129.6, 128.4, 127.8, 115.0, 51.9, 33.1, 30.0; **ATR-FTIR (cm $^{-1}$):** 3076, 2927, 1718, 1612, 1434, 1274, 1178, 1104, 1019, 911, 830, 751, 705; **HRMS m/z (ESI)** calculated for $C_{13}H_{15}D_2O_2^+$ $[M+H]^+$ 207.1349, found 207.1341.

The characterization data of the corresponding non-deuterated product are as follows:

methyl 4-(pent-4-en-1-yl)benzoate (5aa')

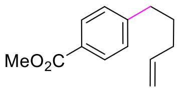

^{1}H NMR (400 MHz, $CDCl_3$) δ 7.95 (d, *J* = 8.2 Hz, 2H), 7.24 (d, *J* = 8.2 Hz, 2H), 5.82 (ddt, *J* = 16.9, 10.2, 6.7 Hz, 1H), 5.34 – 4.75 (m, 2H), 3.90 (s, 3H), 3.02 – 2.46 (m, 2H), 2.09 (q, *J* = 7.1 Hz, 2H), 1.73 (m, 2H); **^{13}C NMR (101 MHz, $CDCl_3$)** δ 167.1, 148.0, 138.2, 129.6, 128.4, 127.8, 115.0, 51.9, 35.3, 33.2, 30.2; **ATR-FTIR (cm $^{-1}$):** 3076, 2931, 1718, 1610, 1435, 1273, 1178, 1107, 1020, 911, 833, 762, 704; **HRMS m/z (ESI)** calculated for $C_{13}H_{17}O_2^+$ $[M+H]^+$ 205.1223, found 205.1215. Spectroscopic data are in agreement with the literature.[2]

methyl 4-(3-phenylpropyl-1,1-*d*$_2$)benzoate (5ba)

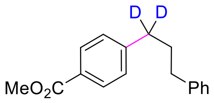

According to general procedure C, the crude product was purified by flash column chromatography on silica gel (PE : EA = 30:1) to afford **5ba** (38.5 mg, 75% yield, 96% D) as a colorless oil: **^{1}H NMR (400 MHz, $CDCl_3$)** δ 8.05 – 7.88 (m, 2H), 7.39 – 7.20 (m, 4H), 7.19 – 7.13 (m, 3H), 3.89 (s, 3H), 2.82 – 2.52 (m, 2H), 1.95 (t, *J* = 7.7 Hz, 2H); **^{13}C NMR (101 MHz, $CDCl_3$)** δ 167.1, 147.7, 141.9, 129.6, 128.4, 128.4, 128.3, 127.8, 125.8, 51.9, 35.3, 32.4; **ATR-FTIR (cm $^{-1}$):** 3027, 2928, 2859, 1716, 1611, 1434, 1274, 1178, 1104, 1019, 967, 747, 698; **HRMS m/z (ESI)** calculated for $C_{17}H_{17}D_2O_2^+$ $[M+H]^+$ 257.1505, found 257.1506.

The characterization data of the corresponding non-deuterated product are as follows:

methyl 4-(3-phenylpropyl)benzoate (5ba')

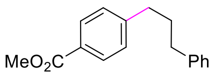

^{1}H NMR (400 MHz, $CDCl_3$) δ 7.95 (d, *J* = 8.2 Hz, 2H), 7.37 – 7.22 (m, 4H), 7.19 – 7.13 (m, 3H), 3.89 (s, 3H), 2.74 – 2.67 (m, 2H), 2.67 – 2.60 (m, 2H), 2.04 – 1.90 (m, 2H); **^{13}C NMR (101 MHz, $CDCl_3$)** δ 167.1, 147.8, 141.9, 129.7, 128.4, 128.4, 128.3, 127.8, 125.8, 51.9, 35.4, 35.3, 32.6; **ATR-FTIR (cm $^{-1}$):** 3027, 2940, 2857, 1717, 1608, 1434, 1274,

1178, 1107, 1019, 967, 761, 699; **HRMS m/z (ESI)** calculated for $C_{17}H_{19}O_2^+$ $[M+H]^+$ 255.1380, found 255.1383. Spectroscopic data are in agreement with the literature.[3]

methyl 4-(2-(thiophen-2-yl)ethyl-1,1-*d*$_2$)benzoate (5ca)

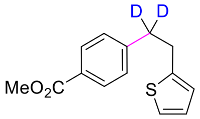

According to general procedure C, the crude product was purified by flash column chromatography on silica gel (PE : EA = 30:1) to afford **5ca** (39.3 mg, 79% yield, 97% D) as a colorless oil: **^{1}H NMR (400 MHz, CDCl$_3$)** δ 8.01 – 7.91 (m, 2H), 7.32 – 7.20 (m, 2H), 7.11 (dd, *J* = 5.1, 1.1 Hz, 1H), 6.89 (dd, *J* = 5.1, 3.4 Hz, 1H), 6.78 – 6.67 (m, 1H), 3.90 (s, 3H), 3.13 (s, 2H); **^{13}C NMR (101 MHz, CDCl$_3$)** δ 167.0, 146.4, 143.7, 129.7, 128.5, 128.1, 126.7, 124.5, 123.2, 52.0, 31.2; **ATR-FTIR (cm $^{-1}$):** 2950, 2850, 1716, 1612, 1434, 1274, 1179, 1105, 1018, 967, 84c9, 752, 694; **HRMS m/z (ESI)** calculated for $C_{14}H_{13}D_2O_2S^+$ $[M+H]^+$ 249.0913, found 249.0908.

The characterization data of the corresponding non-deuterated product are as follows:

methyl 4-(2-(thiophen-2-yl)ethyl)benzoate (5ca')

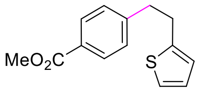

^{1}H NMR (400 MHz, CDCl$_3$) δ 7.95 (d, *J* = 8.1 Hz, 2H), 7.24 (d, *J* = 7.9 Hz, 2H), 7.11 (d, *J* = 4.8 Hz, 1H), 6.98 – 6.85 (m, 1H), 6.73 (d, *J* = 2.6 Hz, 1H), 3.90 (s, 3H), 3.33 – 3.09 (m, 2H), 3.09 – 2.95 (m, 2H); **^{13}C NMR (101 MHz, CDCl$_3$)** δ 167.0, 146.4, 143.7, 129.7, 128.5, 128.1, 126.7, 124.5, 123.2, 52.0, 38.0, 31.3; **ATR-FTIR (cm $^{-1}$):** 2949, 2850, 1715, 1610, 1434, 1274, 1178, 1108, 1020, 966, 849, 764, 695; **HRMS m/z (ESI)** calculated for $C_{14}H_{15}O_2S^+$ $[M+H]^+$ 247.0787, found 247.0783.

methyl 4-(2-(benzofuran-2-yl)ethyl-1,1-*d*$_2$)benzoate (5da)

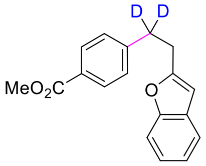

According to general procedure C, the crude product was purified by flash column chromatography on silica gel (PE : EA = 30:1) to afford **5da** (45.3 mg, 80% yield, 97% D) as a white solid: **^{1}H NMR (400 MHz, CDCl$_3$)** δ 8.09 – 7.83 (m, 2H), 7.56 – 7.35 (m, 2H), 7.25 (d, *J* = 8.4 Hz, 2H), 7.25 – 7.14 (m, 2H), 6.49 – 5.98 (m, 1H), 3.88 (s, 3H), 3.07 (s, 2H); **^{13}C NMR (101 MHz, CDCl$_3$)** δ 167.0, 157.7, 154.6, 146.1, 129.8, 128.7, 128.4, 128.2, 123.3, 122.5, 120.3, 110.7, 102.6, 51.9, 29.8; **ATR-FTIR (cm $^{-1}$):** 3053, 2949, 2843,

1716, 1612, 1454, 1434, 1279, 1265, 1179, 1106, 1018, 940, 826, 734, 703; **HRMS m/z (ESI)** calculated for $C_{18}H_{15}D_2O_3^+$ $[M+H]^+$ 283.1298, found 283.1296.

The characterization data of the corresponding non-deuterated product are as follows:

methyl 4-(2-(benzofuran-2-yl)ethyl)benzoate (5da')

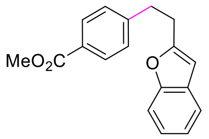

^{1}H NMR (400 MHz, CDCl$_3$) δ 7.95 (d, J = 8.3 Hz, 2H), 7.53 – 7.38 (m, 2H), 7.26 (d, J = 8.2 Hz, 2H), 7.24 – 7.13 (m, 2H), 6.33 (s, 1H), 3.89 (s, 3H), 3.16 – 3.04 (m, 4H); **^{13}C NMR (101 MHz, CDCl$_3$)** δ 167.0, 157.7, 154.7, 146.2, 129.8, 128.7, 128.4, 128.2, 123.4, 122.5, 120.4, 110.7, 102.6, 51.9, 33.9, 29.9; **ATR-FTIR (cm $^{-1}$):** 3056, 2949, 2842, 1706, 1610, 1453, 1434, 1283, 1254, 1183, 1108, 1021, 956, 807, 751, 716; **HRMS m/z (ESI)** calculated for $C_{18}H_{17}O_3^+$ $[M+H]^+$ 281.1172, found 281.1170.

methyl 4-(3-(9*H*-carbazol-9-yl)propyl-1,1-*d*$_2$)benzoate (5ea)

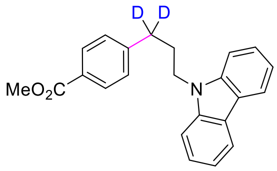

According to general procedure C, the crude product was purified by flash column chromatography on silica gel (PE : EA = 30:1) to afford **5ea** (45.1 mg, 65% yield, 96% D) as a brown solid: **^{1}H NMR (400 MHz, CDCl$_3$)** δ 8.09 (d, J = 7.7 Hz, 2H), 8.03 – 7.88 (m, 2H), 7.49 – 7.38 (m, 2H), 7.30 (d, J = 8.2 Hz, 2H), 7.26 – 7.17 (m, 4H), 4.31 (t, J = 7.2 Hz, 2H), 3.89 (s, 3H), 2.20 (t, J = 7.1 Hz, 2H); **^{13}C NMR (101 MHz, CDCl$_3$)** δ 167.0, 146.4, 140.3, 129.8, 128.3, 128.2, 125.6, 122.9, 120.4, 118.9, 108.5, 52.0, 42.3, 29.7; **ATR-FTIR (cm $^{-1}$):** 3049, 2948, 1714, 1611, 1595, 1452, 1434, 1275, 1179, 1107, 1018, 966, 748, 722, 703, 423; **HRMS m/z (ESI)** calculated for $C_{23}H_{20}D_2NO_2^+$ $[M+H]^+$ 346.1771, found 346.1765.

The characterization data of the corresponding non-deuterated product are as follows:

methyl 4-(3-(9*H*-carbazol-9-yl)propyl)benzoate (5ea')

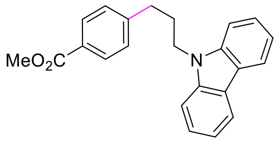

^{1}H NMR (400 MHz, CDCl$_3$) δ 8.08 (d, J = 7.8 Hz, 2H), 7.93 (d, J = 8.2 Hz, 2H), 7.48 – 7.37 (m, 2H), 7.29 (d, J = 8.2 Hz, 2H), 7.26 – 7.15 (m, 4H), 4.28 (t, J = 7.2 Hz, 2H), 3.88 (s, 3H), 2.89 – 2.54 (m, 2H), 2.19 (p, J = 7.4 Hz, 2H); **^{13}C NMR (101 MHz, CDCl$_3$)** δ 167.0, 146.4, 140.3, 129.8, 128.3, 128.1, 125.6,

122.9, 120.4, 118.9, 108.5, 51.9, 42.3, 33.3, 29.8; **ATR-FTIR (cm -1):** 3049, 2947, 1714, 1608, 1596, 1452, 1434, 1275, 1178, 1105, 1019, 966, 748, 722, 702, 423; **HRMS m/z (ESI)** calculated for $C_{23}H_{22}NO_2^+$ $[M+H]^+$ 344.1645, found 344.1641.

methyl 4-(2-(1,3-dioxoisoindolin-2-yl)ethyl-1,1-*d*2)benzoate (5fa)

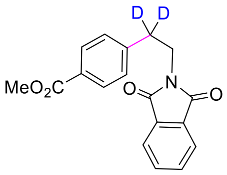

According to general procedure C, the crude product was purified by flash column chromatography on silica gel (PE : EA = 5:1) to afford **5fa** (35.7 mg, 57% yield, 95% D) as a white solid: **^{1}H NMR (400 MHz, CDCl3)** δ 7.99 – 7.91 (m, 2H), 7.87 – 7.79 (m, 2H), 7.76 – 7.67 (m, 2H), 7.36 – 7.28 (m, 2H), 3.94 (s, 2H), 3.89 (s, 3H); **^{13}C NMR (101 MHz, CDCl3)** δ 168.0, 166.9, 143.3, 134.0, 132.0, 129.9, 128.9, 128.6, 123.3, 52.0, 38.6; **ATR-FTIR (cm -1):** 2953, 1772, 1700, 1614, 1434, 1394, 1275, 1179, 1102, 994, 862, 756, 712, 531; **HRMS m/z (ESI)** calculated for $C_{18}H_{13}D_2NO_4Na^+$ $[M+Na]^+$ 334.1019, found 334.1018.

The characterization data of the corresponding non-deuterated product are as follows:

methyl 4-(2-(1,3-dioxoisoindolin-2-yl)ethyl)benzoate (5fa')

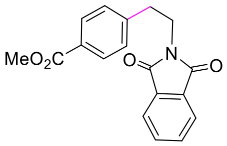

^{1}H NMR (400 MHz, CDCl3) δ 7.98 – 7.92 (m, 2H), 7.85 – 7.79 (m, 2H), 7.74 – 7.67 (m, 2H), 7.31 (d, *J* = 8.3 Hz, 2H), 3.99 – 3.92 (m, 2H), 3.89 (s, 3H), 3.10 – 3.02 (m, 2H); **^{13}C NMR (101 MHz, CDCl3)** δ 168.0, 166.9, 143.3, 134.0, 131.9, 129.8, 128.9, 128.6, 123.2, 52.0, 38.8, 34.5; **ATR-FTIR (cm -1):** 2949, 1770, 1705, 1609, 1433, 1394, 1273, 1178, 1106, 995, 869, 765, 712, 533; **HRMS m/z (ESI)** calculated for $C_{18}H_{15}NO_4Na^+$ $[M+Na]^+$ 332.0893, found 332.0892. Spectroscopic data are in agreement with the literature.[4]

methyl 4-(2-methoxyethyl-1,1-*d*2)benzoate (5ga)

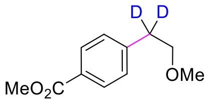

According to general procedure C, the crude product was purified by flash column chromatography on silica gel (PE : EA = 40:1) to afford **5ga** (31.3 mg, 80% yield, 96% D) as a colorless oil: **^{1}H NMR (400 MHz, CDCl3)** δ 7.96 (d, *J* = 7.9 Hz, 2H), 7.29 (d, *J* = 7.9 Hz, 2H), 3.90 (s, 3H), 3.61 (s, 2H), 3.34 (s, 3H); **^{13}C NMR (101 MHz, CDCl3)** δ 167.0,

144.5, 129.7, 128.8, 128.2, 72.9, 58.7, 51.9; **ATR-FTIR (cm $^{-1}$):** 2952, 2874, 1720, 1613, 1435, 1278, 1178, 1111, 1020, 967, 754, 704; **HRMS m/z (ESI)** calculated for $C_{11}H_{13}D_2O_3^+$ [M+H]$^+$ 197.1141, found 197.1139.

The characterization data of the corresponding non-deuterated product are as follows:

methyl 4-(2-methoxyethyl)benzoate (5ga')

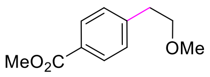

^{1}H NMR (400 MHz, CDCl$_3$) δ 7.96 (d, *J* = 8.2 Hz, 2H), 7.29 (d, *J* = 8.1 Hz, 2H), 3.90 (s, 3H), 3.62 (t, *J* = 6.8 Hz, 2H), 3.34 (s, 3H), 2.93 (t, *J* = 6.8 Hz, 2H); **^{13}C NMR (101 MHz, CDCl$_3$)** δ 167.0, 144.6, 129.7, 128.8, 128.2, 72.9, 58.6, 51.9, 36.2; **ATR-FTIR (cm $^{-1}$):** 2951, 2872, 1718, 1611, 1435, 1274, 1179, 1103, 1020, 968, 753, 704; **HRMS m/z (ESI)** calculated for $C_{11}H_{15}O_3^+$ [M+H]$^+$ 195.1016, found 195.1012. Spectroscopic data are in agreement with the literature.[5]

methyl 4-(3-(benzyloxy)propyl-1,1-*d*$_2$)benzoate (5ha)

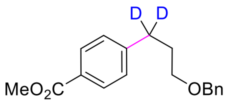

According to general procedure C, the crude product was purified by flash column chromatography on silica gel (PE : EA = 40:1) to afford **5ha** (44.7 mg, 78% yield, 97% D) as a colorless oil: **^{1}H NMR (400 MHz, CDCl$_3$)** δ 8.05 – 7.88 (m, 2H), 7.34 (d, *J* = 3.5 Hz, 4H), 7.33 – 7.26 (m, 1H), 7.25 – 7.19 (m, 2H), 4.49 (s, 2H), 3.89 (s, 3H), 3.47 (t, *J* = 6.2 Hz, 2H), 1.92 (t, *J* = 6.2 Hz, 2H); **^{13}C NMR (101 MHz, CDCl$_3$)** δ 167.1, 147.4, 138.4, 129.6, 128.4, 128.3, 127.8, 127.6, 127.5, 72.9, 69.1, 51.9, 30.8; **ATR-FTIR (cm $^{-1}$):** 2949, 2856, 1716, 1611, 1435, 1275, 1178, 1103, 1019, 967, 749, 697; **HRMS m/z (ESI)** calculated for $C_{18}H_{19}D_2O_3^+$ [M+H]$^+$ 287.1611, found 287.1610.

The characterization data of the corresponding non-deuterated product are as follows:

methyl 4-(3-(benzyloxy)propyl)benzoate (5ha')

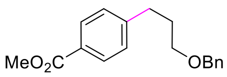

^{1}H NMR (400 MHz, CDCl$_3$) δ 7.94 (d, *J* = 8.0 Hz, 2H), 7.40 – 7.26 (m, 5H), 7.23 (d, *J* = 8.2 Hz, 2H), 4.49 (s, 2H), 3.89 (s, 3H), 3.47 (t, *J* = 6.2 Hz, 2H), 2.77 (t, *J* = 7.7 Hz, 2H), 1.99 – 1.88 (m, 2H); **^{13}C NMR (101 MHz, CDCl$_3$)** δ 167.1, 147.5, 138.4, 129.6, 128.5, 128.3, 127.8, 127.6, 127.5, 72.9, 69.2, 51.9, 32.4, 31.0; **ATR-FTIR (cm $^{-1}$):** 2949,

2855, 1717, 1610, 1434, 1274, 1178, 1102, 1019, 967, 735, 697; **HRMS m/z (ESI)** calculated for $C_{18}H_{21}O_3^+$ [M+H]$^+$ 285.1485, found 285.1485.

methyl 4-(4,4,4-trifluorobutyl-1,1-*d*$_2$)benzoate (5ia)

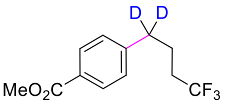

According to general procedure C, the crude product was purified by flash column chromatography on silica gel (PE : EA = 40:1) to afford **5ia** (39.4 mg, 79% yield, 97% D) as a colorless oil: **^{1}H NMR (400 MHz, CDCl$_3$)** δ 8.00 (d, *J* = 7.4 Hz, 2H), 7.27 (d, *J* = 7.4 Hz, 2H), 3.93 (s, 3H), 2.35 – 1.99 (m, 2H), 2.05 – 1.81 (m, 2H); **^{13}C NMR (101 MHz, CDCl$_3$)** δ 166.9, 146.0, 129.9, 128.4, 127.0 (q, *J* = 276.2 Hz), 125.7, 52.0, 33.0 (q, *J* = 28.7 Hz), 23.0 (q, *J* = 3.0 Hz); **^{19}F NMR (376 MHz, CDCl$_3$)** δ -66.2; **ATR-FTIR (cm $^{-1}$):** 2953, 1718, 1613, 1437, 1386, 1277, 1252, 1138, 1107, 968, 832, 752, 705; **HRMS m/z (ESI)** calculated for $C_{12}H_{12}D_2F_3O_2^+$ [M+H]$^+$ 249.1066, found 249.1062.

The characterization data of the corresponding non-deuterated product are as follows:

methyl 4-(4,4,4-trifluorobutyl)benzoate (5ia')

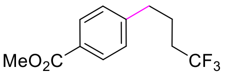

^{1}H NMR (400 MHz, CDCl$_3$) δ 7.98 (d, *J* = 8.3 Hz, 2H), 7.25 (d, *J* = 8.3 Hz, 2H), 3.91 (s, 3H), 2.75 (t, *J* = 7.6 Hz, 2H), 2.25 – 1.97 (m, 2H), 1.97 – 1.86 (m, 2H); **^{13}C NMR (101 MHz, CDCl$_3$)** δ 167.0, 146.0, 129.9, 128.4, 127.0 (q, *J* = 276.3 Hz), 125.6, 52.0, 34.6, 33.0 (q, *J* = 28.7 Hz), 23.2 (q, *J* = 3.0 Hz); **^{19}F NMR (376 MHz, CDCl$_3$)** δ -66.2; **ATR-FTIR (cm $^{-1}$):** 2953, 1718, 1611, 1437, 1275, 1251, 1130, 1107, 967, 854, 763, 705; **HRMS m/z (ESI)** calculated for $C_{12}H_{14}F_3O_2^+$ [M+H]$^+$ 247.0940, found 247.0939.

methyl 4-(4-chlorobutyl-1,1-*d*$_2$)benzoate (5ja)

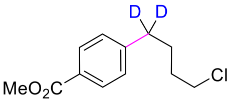

According to general procedure C, the crude product was purified by flash column chromatography on silica gel (PE : EA = 40:1) to afford **5ja** (36.5 mg, 80% yield, 97% D) as a colorless oil: **^{1}H NMR (400 MHz, CDCl$_3$)** δ 7.96 (d, *J* = 7.9 Hz, 2H), 7.24 (d, *J* = 7.9 Hz, 2H), 3.90 (s, 3H), 3.54 (t, *J* = 5.6 Hz, 2H), 1.86 – 1.72 (m, 4H); **^{13}C NMR (101 MHz, CDCl$_3$)** δ 167.0, 147.2, 129.7, 128.3, 128.0, 51.9, 44.6, 31.9, 28.0; **ATR-FTIR (cm $^{-1}$):** 2951, 2864, 1716, 1612, 1434, 1274, 1178, 1105, 1019, 967, 858, 751, 703; **HRMS m/z (ESI)** calculated

for $C_{12}H_{14}D_2ClO_2^+$ $[M+H]^+$ 229.0959, found 229.0955.

The characterization data of the corresponding non-deuterated product are as follows:

methyl 4-(4-chlorobutyl)benzoate (5ja')

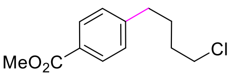

^{1}H NMR (400 MHz, CDCl$_3$) δ 7.96 (d, *J* = 8.2 Hz, 2H), 7.25 (d, *J* = 8.1 Hz, 2H), 3.90 (s, 3H), 3.54 (t, *J* = 6.0 Hz, 2H), 2.70 (t, *J* = 6.9 Hz, 2H), 1.88 – 1.72 (m, 4H); **^{13}C NMR (101 MHz, CDCl$_3$)** δ 167.0, 147.3, 129.7, 128.4, 127.9, 52.0, 44.7, 35.1, 31.9, 28.1; **ATR-FTIR (cm $^{-1}$):** 2949, 2862, 1716, 1610, 1434, 1273, 1178, 1108, 1019, 967, 856, 762, 704; **HRMS m/z (ESI)** calculated for $C_{12}H_{16}ClO_2^+$ $[M+H]^+$ 227.0833, found 227.0833. Spectroscopic data are in agreement with the literature.[6]

methyl 4-(but-3-en-1-yl-1,1-*d*$_2$)benzoate (5ka)

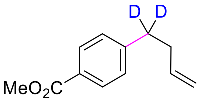

According to general procedure C, the crude product was purified by flash column chromatography on silica gel (PE : EA = 50:1) to afford **5ka** (29.3 mg, 76% yield, 96% D) as a colorless oil: **^{1}H NMR (400 MHz, CDCl$_3$)** δ 7.98 (d, *J* = 8.3 Hz, 2H), 7.27 (d, *J* = 8.4 Hz, 2H), 5.85 (ddt, *J* = 16.9, 10.2, 6.6 Hz, 1H), 5.12 – 4.93 (m, 2H), 3.92 (s, 3H), 2.39 (d, *J* = 6.6 Hz, 2H); **^{13}C NMR (101 MHz, CDCl$_3$)** δ 167.1, 147.2, 137.4, 129.6, 128.4, 127.8, 115.3, 51.9, 34.9; **ATR-FTIR (cm $^{-1}$):** 3078, 2952, 2845, 1719, 1612, 1435, 1275, 1179, 1105, 1019, 970, 913, 830, 753, 703; **HRMS m/z (ESI)** calculated for $C_{12}H_{13}D_2O_2^+$ $[M+H]^+$ 193.1192, found 193.1187.

The characterization data of the corresponding non-deuterated product are as follows:

methyl 4-(but-3-en-1-yl)benzoate (5ka')

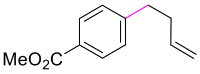

^{1}H NMR (400 MHz, CDCl$_3$) δ 7.98 (d, *J* = 8.1 Hz, 2H), 7.27 (d, *J* = 8.0 Hz, 2H), 5.85 (ddt, *J* = 16.9, 10.2, 6.6 Hz, 1H), 5.11 – 4.95 (m, 2 H), 3.92 (s, 3H), 2.79 (t, *J* = 7.7 Hz, 2H), 2.41 (q, *J* = 7.2 Hz, 2H); **^{13}C NMR (101 MHz, CDCl$_3$)** δ 167.1, 147.3, 137.4, 129.6, 128.5, 127.8, 115.3, 51.9, 35.4, 35.0; **ATR-FTIR (cm $^{-1}$):** 3077, 2950, 2856, 1719, 1610, 1435, 1274, 1178, 1107, 1020, 968, 911, 839, 762, 703; **HRMS m/z (ESI)** calculated for $C_{12}H_{15}O_2^+$ $[M+H]^+$ 191.1067, found 191.1060. Spectroscopic data are in agreement with the

literature.[7]

methyl (*Z*)-4-(octadec-9-en-1-yl-1,1-d_2)benzoate (5la)

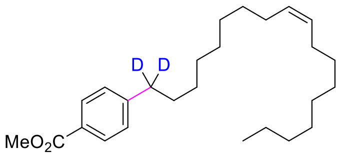

According to general procedure C, the crude product was purified by flash column chromatography on silica gel (PE : EA = 50:1) to afford **5la** (56.6 mg, 73% yield, 95% D) as a colorless oil: **^{1}H NMR (400 MHz, $CDCl_3$)** δ 7.97 (d, J = 7.9 Hz, 2H), 7.26 (d, J = 7.9 Hz, 2H), 5.43 – 5.29 (m, 2H), 3.92 (s, 3H), 2.13 – 1.92 (m, 4H), 1.63 (t, J = 6.6 Hz, 2H), 1.43 – 1.21 (m, 22H), 0.91 (t, J = 6.6 Hz, 3H); **^{13}C NMR (101 MHz, $CDCl_3$)** δ 167.1, 148.4, 129.9, 129.8, 129.6, 128.4, 127.6, 51.9, 32.6, 31.9, 31.0, 29.8, 29.7, 29.7, 29.6, 29.5, 29.4, 29.3, 29.2, 29.2, 27.2, 22.7, 14.1; **ATR-FTIR (cm $^{-1}$):** 3004, 2922, 2853, 1723, 1612, 1462, 1434, 1275, 1178, 1107, 1020, 968, 829, 751, 703; **HRMS m/z (ESI)** calculated for $C_{26}H_{41}D_2O_2^+$ [M+H]$^+$ 389.3383, found 389.3376.

The characterization data of the corresponding non-deuterated product are as follows:

methyl (*Z*)-4-(octadec-9-en-1-yl)benzoate (5la')

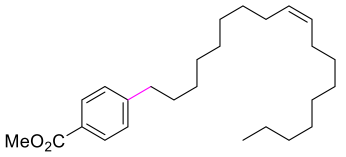

^{1}H NMR (400 MHz, $CDCl_3$) δ 7.95 (d, J = 8.2 Hz, 2H), 7.24 (d, J = 8.1 Hz, 2H), 5.44 – 5.27 (m, 2H), 3.90 (s, 3H), 2.72 – 2.58 (m, 2H), 2.08 – 1.91 (m, 4H), 1.70 – 1.59 (m, 2H), 1.42 – 1.21 (m, 22H), 0.88 (t, J = 6.7 Hz, 3H); **^{13}C NMR (101 MHz, $CDCl_3$)** δ 167.2, 148.5, 129.9, 129.8, 129.6, 128.4, 127.6, 51.9, 36.0, 32.6, 31.9, 31.1, 29.8, 29.7, 29.6, 29.6, 29.5, 29.4, 29.3, 29.2, 29.2, 27.2, 22.7, 14.1; **ATR-FTIR (cm $^{-1}$):** 3005, 2923, 2853, 1724, 1611, 1463, 1434, 1276, 1178, 1108, 1021, 969, 853, 750, 703; **HRMS m/z (ESI)** calculated for $C_{26}H_{43}O_2^+$ [M+H]$^+$ 387.3258, found 387.3251.

methyl 4-(dec-3-yn-1-yl-1,1-d_2)benzoate (5ma)

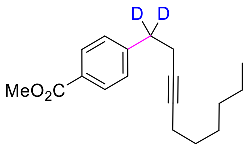

According to general procedure C, the crude product was purified by flash column chromatography on silica gel (PE : EA = 50:1) to afford **5ma** (37.1 mg, 68% yield, 97% D) as a white solid: **^{1}H NMR (400 MHz, $CDCl_3$)** δ 7.96 (d, J = 8.2 Hz, 2H), 7.29 (d, J = 8.2 Hz, 2H), 3.90 (s, 3H), 2.45 (s, 2H), 2.11 (t, J = 7.0 Hz, 2H), 1.49 – 1.37 (m, 2H), 1.37 – 1.18 (m, 6H), 0.88 (t, J = 6.8 Hz, 3H); **^{13}C NMR (101 MHz, $CDCl_3$)** δ

167.1, 146.3, 129.6, 128.6, 128.1, 81.5, 78.8, 52.0, 31.4, 28.9, 28.5, 22.6, 20.4, 18.7, 14.1; **ATR-FTIR (cm $^{-1}$):** 2928, 2857, 2172, 1721, 1613, 1434, 1275, 1179, 1107, 1019, 968, 829, 751, 701; **HRMS m/z (ESI)** calculated for $C_{18}H_{23}D_2O_2^+$ $[M+H]^+$ 275.1975, found 275.1969. The characterization data of the corresponding non-deuterated product are as follows:

methyl 4-(dec-3-yn-1-yl)benzoate (5ma')

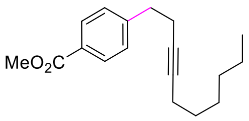

^{1}H NMR (400 MHz, CDCl$_3$) δ 7.96 (d, *J* = 8.3 Hz, 2H), 7.28 (d, *J* = 8.2 Hz, 2H), 3.90 (s, 3H), 2.84 (t, *J* = 7.4 Hz, 2H), 2.50 – 2.41 (m, 2H), 2.11 (ddd, *J* = 7.1, 4.7, 2.4 Hz, 2H), 1.49 – 1.38 (m, 2H), 1.37 – 1.18 (m, 6H), 0.88 (t, *J* = 6.9 Hz, 3H); **^{13}C NMR (101 MHz, CDCl$_3$)** δ 167.1, 146.4, 129.6, 128.5, 128.1, 81.5, 78.8, 51.9, 35.4, 31.4, 28.9, 28.5, 22.5, 20.6, 18.7, 14.0; **ATR-FTIR (cm $^{-1}$):** 2930, 2858, 2171, 1719, 1612, 1435, 1277, 1180, 1108, 1020, 966, 852, 765, 705; **HRMS m/z (ESI)** calculated for $C_{18}H_{25}O_2^+$ $[M+H]^+$ 273.1849, found 273.1842.

methyl 4-(pent-4-yn-1-yl-1,1,5-*d*$_3$)benzoate (5na)

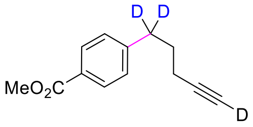

According to general procedure C, the crude product was purified by flash column chromatography on silica gel (PE : EA = 50:1) to afford **5na** (19.8 mg, 48% yield, 97% D) as a colorless oil: **^{1}H NMR (400 MHz, CDCl$_3$)** δ 7.99 – 7.93 (m, 2H), 7.29 – 7.24 (m, 2H), 3.90 (s, 3H), 2.20 (td, *J* = 7.0, 2.1 Hz, 2H), 1.84 (t, *J* = 6.9 Hz, 2H); **^{13}C NMR (101 MHz, CDCl$_3$)** δ 167.1, 146.9, 129.7, 128.5, 128.0, 68.9, 52.0, 29.5, 17.7; **ATR-FTIR (cm $^{-1}$):** 3298, 2950, 2116, 1718, 1612, 1435, 1278, 1179, 1109, 1020, 968, 855, 751, 635; **HRMS m/z (ESI)** calculated for $C_{13}H_{12}D_3O_2^+$ $[M+H]^+$ 206.1255, found 206.1248. The characterization data of the corresponding non-deuterated product are as follows:

methyl 4-(pent-4-yn-1-yl)benzoate (5na')

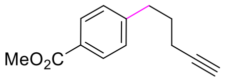

^{1}H NMR (400 MHz, CDCl$_3$) δ 7.96 (d, *J* = 8.2 Hz, 2H), 7.26 (d, *J* = 8.2 Hz, 2H), 3.90 (s, 3H), 2.79 (t, *J* = 7.6 Hz, 2H), 2.20 (td, *J* = 7.0, 2.6 Hz, 2H), 2.00 (t, *J* = 2.6 Hz, 1H), 1.92 – 1.80 (m, 2H); **^{13}C NMR (101 MHz, CDCl$_3$)** δ 167.1, 147.0, 129.7, 128.5, 128.0, 83.8, 69.0, 52.0, 34.6, 29.6, 17.8; **ATR-FTIR (cm $^{-1}$):** 3298, 2950, 2118, 1717, 1610, 1435,

1276, 1179, 1102, 1020, 967, 842, 762, 636; **HRMS m/z (ESI)** calculated for $C_{13}H_{15}O_2^+$ [M+H]$^+$ 203.1067, found 203.1059.

methyl 4-((*R*)-4-((3*R*,5*S*,8*R*,9*S*,10*S*,13*R*,14*S*,17*R*)-3-methoxy-10,13-dimethylhexadecahydro-1*H*-cyclopenta[a]phenanthren-17-yl)pentyl-1,1-*d*$_2$)benzoate (5oa)

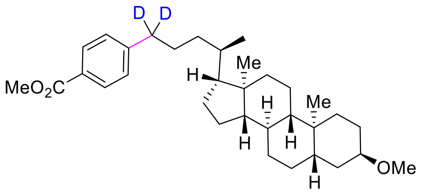

According to general procedure C, the crude product was purified by flash column chromatography on silica gel (PE : EA = 30:1) to afford **5oa**(61.8 mg, 62% yield, 97% D) as a white solid : **^{1}H NMR (400 MHz, CDCl$_3$)** δ 7.94 (d, *J* = 8.1 Hz, 2H), 7.23 (d, *J* = 8.1 Hz, 2H), 3.89 (s, 3H), 3.34 (s, 3H), 3.23 – 3.07 (m, 1H), 1.94 (d, *J* = 12.2 Hz, 1H), 1.89 – 1.46 (m, 9H), 1.47 – 0.97 (m, 18H), 0.95 – 0.85 (m, 6H), 0.62 (s, 3H); **^{13}C NMR (101 MHz, CDCl$_3$)** δ 167.2, 148.4, 129.6, 128.4, 127.6, 80.4, 56.4, 56.1, 55.5, 51.8, 42.7, 42.1, 40.4, 40.2, 35.8, 35.6, 35.5, 35.3, 34.9, 32.8, 28.2, 27.5, 27.3, 26.8, 26.4, 24.2, 23.4, 20.8, 18.6, 12.0; **ATR-FTIR (cm^{-1}):** 2927, 2863, 1721, 1612, 1447, 1435, 1372, 1275, 1177, 1099, 1019, 937, 830, 737, 704; **HRMS m/z (ESI)** calculated for $C_{33}H_{49}D_2O_3^+$ [M+H]$^+$ 497.3958, found 497.3957.

The characterization data of the corresponding non-deuterated product are as follows:

methyl 4-((*R*)-4-((3*R*,5*S*,8*R*,9*S*,10*S*,13*R*,14*S*,17*R*)-3-methoxy-10,13-dimethylhexadecahydro-1*H*-cyclopenta[a]phenanthren-17-yl)pentyl)benzoate (5oa')

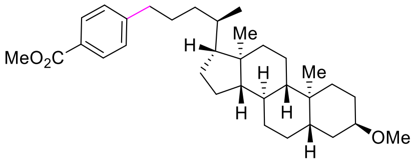

^{1}H NMR (400 MHz, CDCl$_3$) δ 7.93 (d, *J* = 8.1 Hz, 2H), 7.23 (d, *J* = 8.1 Hz, 2H), 3.89 (s, 3H), 3.33 (s, 3H), 3.21 – 3.06 (m, 1H), 2.76 – 2.49 (m, 2H), 1.93 (d, *J* = 12.2 Hz, 1H), 1.89 – 1.45 (m, 9H), 1.44 – 1.30 (m, 7H), 1.29 – 1.15 (m, 4H), 1.14 – 0.97 (m, 6H), 0.96 – 0.84 (m, 7H), 0.62 (s, 3H); **^{13}C NMR (101 MHz, CDCl$_3$)** δ 167.1, 148.5, 129.6, 128.3, 127.6, 80.4, 56.4, 56.1, 55.5, 51.8, 42.7, 42.0, 40.3, 40.2, 36.4, 35.8, 35.6, 35.5, 35.3, 34.8, 32.8, 28.2, 27.6, 27.3, 26.8, 26.4, 24.2, 23.4, 20.8, 18.6, 12.0; **ATR-FTIR (cm^{-1}):** 2928, 2863, 1721, 1610, 1447, 1435, 1372, 1275, 1177, 1099, 1020, 938, 834, 736, 703; **HRMS m/z (ESI)** calculated for $C_{33}H_{51}O_3^+$ [M+H]$^+$ 495.3833, found 495.3830.

methyl 4-(3-(((8*R*,9*S*,13*S*,14*S*)-13-methyl-17-oxo-7,8,9,11,12,13,14,15,16,17-decahydro-6*H*-

cyclopenta[*a*]phenanthren-3-yl)oxy)propyl-1,1-*d*$_2$)benzoate (5pa)

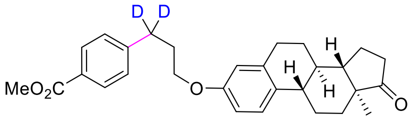

According to general procedure C, the crude product was purified by flash column chromatography on silica gel (PE : EA = 5:1) to afford **5pa** (69.2 mg, 77% yield, 97% D) as a white solid: **^{1}H NMR (400 MHz, CDCl$_3$)** δ 7.96 (d, *J* = 8.3 Hz, 2H), 7.28 (d, *J* = 8.3 Hz, 2H), 7.19 (d, *J* = 8.6 Hz, 1H), 6.73 – 6.67 (m, 1H), 6.63 (d, *J* = 2.5 Hz, 1H), 3.92 (t, *J* = 6.2 Hz, 2H), 3.90 (s, 3H), 2.93 – 2.81 (m, 2H), 2.50 (dd, *J* = 18.8, 8.5 Hz, 1H), 2.44 – 2.34 (m, 1H), 2.30 – 2.20 (m, 1H), 2.21 – 1.90 (m, 6H), 1.77 – 1.35 (m, 6H), 0.91 (s, 3H); **^{13}C NMR (101 MHz, CDCl$_3$)** δ 220.9, 167.1, 156.9, 147.0, 137.7, 132.1, 129.7, 128.6, 127.9, 126.3, 114.5, 112.1, 66.5, 52.0, 50.4, 48.0, 44.0, 38.4, 35.8, 31.6, 30.4, 29.6, 26.5, 25.9, 21.6, 13.8; **ATR-FTIR (cm $^{-1}$):** 2930, 2874, 1732, 1717, 1609, 1496, 1434, 1274, 1255, 1103, 1057, 887, 749, 739; **HRMS m/z (ESI)** calculated for $C_{29}H_{33}D_2O_4^+$ [M+H]$^+$ 449.2655, found 449.2646.

The characterization data of the corresponding non-deuterated product are as follows:

methyl 4-(3-(((8*R*,9*S*,13*S*,14*S*)-13-methyl-17-oxo-7,8,9,11,12,13,14,15,16,17-decahydro-6*H*-cyclopenta[*a*]phenanthren-3-yl)oxy)propyl)benzoate (5pa')

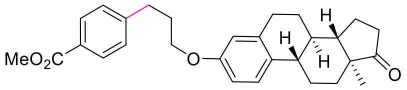

^{1}H NMR (400 MHz, CDCl$_3$) δ 7.96 (d, *J* = 8.3 Hz, 2H), 7.28 (d, *J* = 8.3 Hz, 2H), 7.19 (d, *J* = 8.6 Hz, 1H), 6.71 (dd, *J* = 8.6, 2.7 Hz, 1H), 6.63 (d, *J* = 2.6 Hz, 1H), 3.93 (t, *J* = 6.2 Hz, 2H), 3.90 (s, 3H), 2.95 – 2.81 (m, 4H), 2.50 (dd, *J* = 18.8, 8.5 Hz, 1H), 2.45 – 2.35 (m, 1H), 2.30 – 2.20 (m, 1H), 2.20 – 1.89 (m, 6H), 1.68 – 1.36 (m, 6H), 0.91 (s, 3H); **^{13}C NMR (101 MHz, CDCl$_3$)** δ 220.9, 167.1, 156.9, 147.1, 137.7, 132.0, 129.7, 128.5, 127.9, 126.3, 114.5, 112.1, 66.5, 51.9, 50.4, 48.0, 43.9, 38.3, 35.8, 32.2, 31.5, 30.5, 29.6, 26.5, 25.9, 21.5, 13.8; **ATR-FTIR (cm $^{-1}$):** 2930, 2874, 1732, 1718, 1608, 1496, 1432, 1273, 1254, 1103, 1056, 887, 768, 736; **HRMS m/z (ESI)** calculated for $C_{29}H_{35}O_4^+$ [M+H]$^+$ 447.2530, found 447.2520.

methyl 4-(3-(((8*S*,9*S*,10*R*,13*R*,14*S*,17*R*)-10,13-dimethyl-17-((*R*)-6-methylheptan-2-yl)-2,3,4,7,8,9,10,11,12,13,14,15,16,17-tetradecahydro-1*H*-cyclopenta[*a*]phenanthren-3-yl)oxy)propyl-1,1-*d*$_2$)benzoate (5qa)

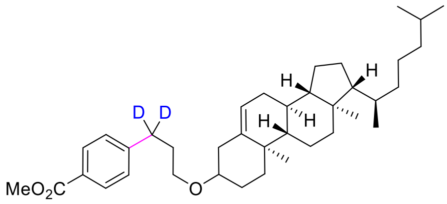

According to general procedure C, the crude product was purified by flash column chromatography on silica gel (PE : EA = 30:1) to afford **5qa** (84.4 mg, 75% yield, 98% D) as a white solid : **^{1}H NMR (400 MHz, CDCl$_3$)** δ 7.95 (d, *J* = 8.1 Hz, 2H), 7.25 (d, *J* = 8.1 Hz, 2H), 5.33 (d, *J* = 5.0 Hz, 1H), 3.89 (s, 3H), 3.50 – 3.37 (m, 2H), 3.22 – 3.02 (m, 1H), 2.38 – 2.30 (m, 1H), 2.26 – 2.14 (m, 1H), 2.05 – 1.92 (m, 2H), 1.92 – 1.76 (m, 5H), 1.65 – 0.96 (m, 24H), 0.91 (d, *J* = 6.5 Hz, 3H), 0.86 (dd, *J* = 6.6, 1.3 Hz, 6H), 0.68 (s, 3H); **^{13}C NMR (101 MHz, CDCl$_3$)** δ 167.1, 147.6, 141.0, 129.6, 128.5, 127.8, 121.5, 79.1, 66.7, 56.8, 56.2, 51.9, 50.2, 42.3, 39.8, 39.5, 39.2, 37.2, 36.9, 36.2, 35.8, 31.9, 31.9, 31.2, 28.5, 28.2, 28.0, 24.3, 23.8, 22.8, 22.5, 21.0, 19.4, 18.7, 11.8; **ATR-FTIR (cm $^{-1}$):** 2932, 2866, 2849, 1722, 1613, 1466, 1435, 1377, 1276, 1178, 1108, 1020, 959, 751, 705; **HRMS m/z (ESI)** calculated for $C_{38}H_{56}D_2O_3Na^+$ $[M+ Na]^+$ 587.4404, found 587.4403.

The characterization data of the corresponding non-deuterated product are as follows:

methyl 4-(3-(((8*S*,9*S*,10*R*,13*R*,14*S*,17*R*)-10,13-dimethyl-17-((*R*)-6-methylheptan-2-yl)-2,3,4,7,8,9,10,11,12,13,14,15,16,17-tetradecahydro-1*H*-cyclopenta[*a*]phenanthren-3-yl)oxy)propyl-1,1-*d*$_2$)benzoate (5qa')

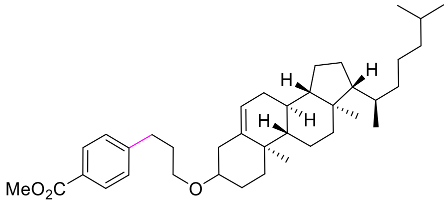

^{1}H NMR (400 MHz, CDCl$_3$) δ 7.95 (d, *J* = 8.2 Hz, 2H), 7.26 (d, *J* = 8.2 Hz, 2H), 5.40 – 5.27 (m, 1H), 3.89 (s, 3H), 3.45 (td, *J* = 6.3, 1.5 Hz, 2H), 3.11 (tt, *J* = 11.2, 4.3 Hz, 1H), 2.84 – 2.65 (m, 2H), 2.34 (ddd, *J* = 13.1, 4.5, 1.9 Hz, 1H), 2.28 – 2.12 (m, 1H), 2.08 – 1.76 (m, 7H), 1.62 – 0.96 (m, 24H), 0.91 (d, *J* = 6.5 Hz, 3H), 0.86 (dd, *J* = 6.6, 1.7 Hz, 6H), 0.68 (s, 3H); **^{13}C NMR (101 MHz, CDCl$_3$)** δ 167.1, 147.6, 141.0, 129.6, 128.5, 127.7, 121.5, 79.1, 66.8, 56.8, 56.1, 51.1, 50.2, 42.3, 39.8, 39.5, 39.2, 37.2, 36.9, 36.2, 35.8, 32.4, 31.9, 31.9, 31.3, 28.5, 28.2, 28.0, 24.3, 23.8, 22.8, 22.5, 21.0, 19.4, 18.7, 11.8; **ATR-FTIR (cm $^{-1}$):** 2932, 2866, 2849, 1722, 1610, 1466, 1434, 1377, 1275, 1178, 1104, 1021, 960, 765, 704; **HRMS m/z (ESI)** calculated for $C_{38}H_{58}O_3Na^+$ $[M+ Na]^+$ 585.4278, found 585.4285.

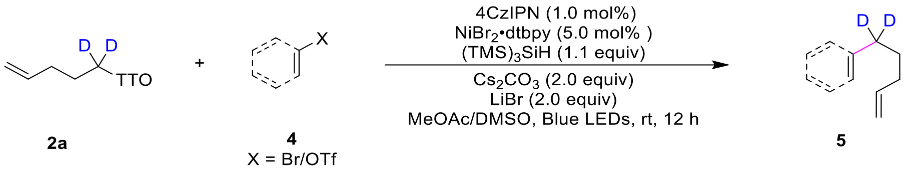

General procedure D: In a nitrogen-filled glove box, to an 8 mL oven-dried vial equipped with a stir bar was added **2a** (173.6 mg, 0.4 mmol, 2 equiv.), Cs_2CO_3 (130.3 mg, 0.4 mmol, 2 equiv.), LiBr (34.7 mg, 0.4 mmol, 2 equiv.), 4CzIPN (1.8 mg, 1 mol%) and $NiBr_2$•dtbpy (4.9 mg, 5 mol%). Then, anhydrous MeOAc (1.6 mL) and anhydrous DMSO (0.4 mL) were added via syringe, followed by addition of **4** (0.2 mmol, 1 equiv.) and tris(trimethylsilyl)silane (68 µL, 0.22 mmol, 1.1 equiv.). The vial was sealed and removed from the glovebox. The reaction mixture was stirred and irradiated with a 40 W blue LED lamp for 12 hours. The final reaction mixture was diluted with EtOAc (60 mL) and saturated aqueous LiCl solution (20 mL). The organic layer was washed with brine (2 x 20 mL) and concentrated. Purification by flash column chromatography on silica gel to afford the aryl alkylation product.

ethyl 2-methyl-2-(4-(pent-4-en-1-yl-1,1-*d*$_2$)phenoxy)propanoate (5ab)

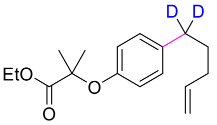

According to General procedure D, the crude product was purified by flash column chromatography on silica gel (PE : EA = 20:1) to afford **5ab** (38.3 mg, 69% yield, 96% D) as a colorless oil: **^{1}H NMR (400 MHz, CDCl$_3$)** δ 7.03 (d, *J* = 8.4 Hz, 2H), 6.77 (d, *J* = 8.4 Hz, 2H), 5.82 (ddt, *J* = 16.9, 10.2, 6.6 Hz, 1H), 5.07 – 4.91 (m, 2H), 4.23 (q, *J* = 7.1 Hz, 2H), 2.06 (q, *J* = 7.2 Hz, 2H), 1.66 (t, *J* = 7.4 Hz, 2H), 1.57 (s, 6H), 1.25 (t, *J* = 7.1 Hz, 3H); **^{13}C NMR (101 MHz, CDCl$_3$)** δ 174.4, 153.4, 138.6, 136.1, 129.0, 119.3, 114.6, 79.1, 61.3, 33.2, 30.5, 25.4, 14.1; **ATR-FTIR (cm $^{-1}$):** 2984, 2928, 2858, 1732, 1508, 1280, 1231, 1136, 1024, 910, 840, 751; **HRMS m/z (ESI)** calculated for $C_{17}H_{23}D_2O_3^+$ [M+H]$^+$ 279.1924, found 279.1933.

The characterization data of the corresponding non-deuterated product are as follows:

ethyl 2-methyl-2-(4-(pent-4-en-1-yl)phenoxy)propanoate (5ab')

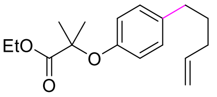

^{1}H NMR (400 MHz, CDCl$_3$) δ 7.06 (d, *J* = 8.5 Hz, 2H), 6.79 (d, *J* = 8.5 Hz, 2H), 5.84 (ddt, *J* = 16.9, 10.2, 6.6 Hz, 1H), 5.09 – 4.93 (m, 2H), 4.26 (q, *J* =

7.1 Hz, 2H), 2.65 – 2.50 (m, 2H), 2.09 (q, J = 7.1 Hz, 2H), 1.78 – 1.64 (m, 2H), 1.60 (s, 6H), 1.27 (t, J = 7.1 Hz, 3H); **^{13}C NMR (101 MHz, CDCl$_3$)** δ 174.4, 153.4, 138.6, 136.2, 129.0, 119.2, 114.6, 79.0, 61.3, 34.4, 33.2, 30.7, 25.4, 14.1; **ATR-FTIR (cm $^{-1}$):** 2984, 2932, 2857, 1732, 1508, 1280, 1231, 1135, 1024, 910, 835, 751; **HRMS m/z (ESI)** calculated for $C_{17}H_{25}O_3^+$ [M+H]$^+$ 277.1798, found 277.1802.

4-((4-(pent-4-en-1-yl-1,1-*d*$_2$)phenyl)sulfonyl)morpholine (5ac)

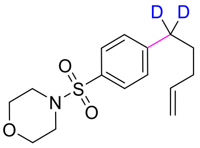

According to General procedure D, the crude product was purified by flash column chromatography on silica gel (PE : EA = 3:1) to afford **5ac** (40.4 mg, 68% yield, 96% D) as a colorless oil: **^{1}H NMR (400 MHz, CDCl$_3$)** δ 7.64 (d, J = 8.4 Hz, 2H), 7.33 (d, J = 8.4 Hz, 2H), 5.80 (ddt, J = 16.9, 10.2, 6.6 Hz, 1H), 5.13 – 4.92 (m, 2H), 3.76 – 3.64 (m, 4H), 3.04 – 2.89 (m, 4H), 2.13 – 2.00 (m, 2H), 1.72 (t, J = 7.3 Hz, 2H); **^{13}C NMR (101 MHz, CDCl$_3$)** δ 148.2, 137.9, 132.4, 129.0, 127.9, 115.1, 66.0, 45.9, 33.0, 29.9; **ATR-FTIR (cm $^{-1}$):** 3074, 2921, 2857, 1599, 1453, 1347, 1261, 1164, 1112, 941, 722, 612, 531; **HRMS m/z (ESI)** calculated for $C_{15}H_{20}D_2NO_3S^+$ [M+H]$^+$ 298.1441, found 298.1451.

The characterization data of the corresponding non-deuterated product are as follows:

4-((4-(pent-4-en-1-yl)phenyl)sulfonyl)morpholine (5ac')

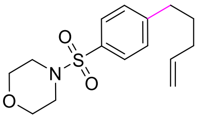

^{1}H NMR (400 MHz, CDCl$_3$) δ 7.66 (d, J = 8.3 Hz, 2H), 7.35 (d, J = 8.4 Hz, 2H), 5.82 (ddt, J = 16.9, 10.2, 6.6 Hz, 1H), 5.14 – 4.91 (m, 2H), 3.80 – 3.66 (m, 4H), 3.06 – 2.94 (m, 4H), 2.75 – 2.67 (m, 2H), 2.11 (q, J = 7.1 Hz, 2H), 1.82 – 1.69 (m, 2H); **^{13}C NMR (101 MHz, CDCl$_3$)** δ 148.3, 137.9, 132.5, 129.1, 128.0, 115.2, 66.1, 46.0, 35.1, 33.1, 30.1; **ATR-FTIR (cm $^{-1}$):** 3074, 2922, 2857, 1597, 1453, 1347, 1261, 1163, 1112, 941, 727, 613, 535; **HRMS m/z (ESI)** calculated for $C_{15}H_{22}NO_3S^+$ [M+H]$^+$ 296.1315, found 296.1327.

4-methoxy-3-(pent-4-en-1-yl-1,1-*d*$_2$)benzonitrile (5ad)

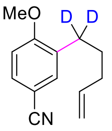

According to General procedure D, the crude product was purified by flash column chromatography on silica gel (PE : EA = 15:1) to afford **5ad** (24.0 mg, 59% yield, 96% D) as a colorless oil: **^{1}H NMR (400 MHz, CDCl$_3$)** δ 7.48 (dd, J = 8.5, 2.1 Hz, 1H), 7.38

(d, J = 2.1 Hz, 1H), 6.86 (d, J = 8.5 Hz, 1H), 5.82 (ddt, J = 16.9, 10.2, 6.6 Hz, 1H), 5.09 – 4.92 (m, 2H), 3.87 (s, 3H), 2.17 – 2.00 (m, 2H), 1.64 (t, J = 7.4 Hz, 2H); **^{13}C NMR (101 MHz, CDCl$_3$)** δ 160.8, 138.3, 133.2, 132.2, 131.9, 119.4, 114.8, 110.5, 103.5, 55.5, 33.2, 28.2; **ATR-FTIR (cm $^{-1}$):** 3076, 2928, 2859, 2224, 1602, 1495, 1256, 1143, 1024, 910, 815, 682; **HRMS m/z (ESI)** calculated for $C_{13}H_{14}D_2NO^+$ [M+H]$^+$ 204.1352, found 204.1355.

The characterization data of the corresponding non-deuterated product are as follows:

4-methoxy-3-(pent-4-en-1-yl)benzonitrile (5ad')

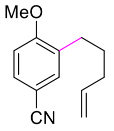

^{1}H NMR (400 MHz, CDCl$_3$) δ 7.49 (dd, J = 8.5, 2.1 Hz, 1H), 7.39 (d, J = 2.0 Hz, 1H), 6.87 (d, J = 8.5 Hz, 1H), 5.83 (ddt, J = 16.9, 10.2, 6.6 Hz, 1H), 5.25 – 4.81 (m, 2H), 3.87 (s, 3H), 2.66 – 2.55 (m, 2H), 2.09 (q, J = 7.1 Hz, 2H), 1.73 – 1.61 (m, 2H); **^{13}C NMR (101 MHz, CDCl$_3$)** δ 160.8, 138.3, 133.2, 132.3, 131.9, 119.5, 114.8, 110.5, 103.5, 55.5, 33.3, 29.2, 28.4; **ATR-FTIR (cm $^{-1}$):** 3076, 2927, 2858, 2224, 1603, 1497, 1256, 1134, 1025, 909, 815, 684; **HRMS m/z (ESI)** calculated for $C_{13}H_{16}NO^+$ [M+H]$^+$ 202.1226, found 202.1230.

3-methyl-5-(pent-4-en-1-yl-1,1-*d*$_2$)picolinonitrile (5ae)

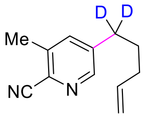

According to General procedure D, the crude product was purified by flash column chromatography on silica gel (PE : EA = 5:1) to afford **4ae** (23.8 mg, 63% yield, 96% D) as a colorless oil: **^{1}H NMR (400 MHz, CDCl$_3$)** δ 8.33 (d, J = 1.4 Hz, 1H), 7.45 (d, J = 0.8 Hz, 1H), 5.78 (ddt, J = 16.9, 10.2, 6.7 Hz, 1H), 5.10 – 4.92 (m, 2H), 2.51 (s, 3H), 2.08 (q, J = 7.0 Hz, 2H), 1.70 (t, J = 7.3 Hz, 2H); **^{13}C NMR (101 MHz, CDCl$_3$)** δ 148.8, 141.5, 137.9, 137.6, 137.5, 131.4, 116.5, 115.5, 32.9, 29.5, 18.5; **ATR-FTIR (cm $^{-1}$):** 3077, 2926, 2858, 2230, 1594, 1455, 1401, 1132, 992, 910, 844, 755; **HRMS m/z (ESI)** calculated for $C_{12}H_{13}D_2N_2^+$ [M+H]$^+$ 189.1355, found 189.1358.

The characterization data of the corresponding non-deuterated product are as follows:

3-methyl-5-(pent-4-en-1-yl)picolinonitrile (5ae')

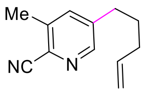

^{1}H NMR (400 MHz, CDCl$_3$) δ 8.36 (s, 1H), 7.47 (s, 1H), 5.80 (ddt, J = 16.9, 10.2, 6.7 Hz, 1H), 5.14 – 4.95 (m, 2H), 2.74 – 2.60 (m, 2H), 2.53 (s, 3H), 2.11 (q, J = 7.1

Hz, 2H), 1.82 – 1.67 (m, 2H); **^{13}C NMR (101 MHz, $CDCl_3$)** δ 148.8, 141.6, 138.0, 137.6, 137.5, 131.5, 116.6, 115.5, 32.9, 32.2, 29.7, 18.5; **ATR-FTIR (cm $^{-1}$):** 3076, 2927, 2859, 2230, 1595, 1455, 1413, 1127, 992, 907, 844, 752; **HRMS m/z (ESI)** calculated for $C_{12}H_{15}N_2^+$ $[M+H]^+$ 187.1230, found 187.1232.

4-(pent-4-en-1-yl-1,1-*d*$_2$)quinoline (5af)

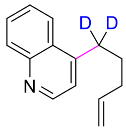

According to General procedure D, the crude product was purified by flash column chromatography on silica gel (PE : EA = 4:1) to afford **5af** (23.2 mg, 58% yield, 96% D) as a colorless oil: **^{1}H NMR (400 MHz, $CDCl_3$)** δ 8.80 (d, *J* = 4.4 Hz, 1H), 8.11 (d, *J* = 8.4 Hz, 1H), 8.02 (d, *J* = 8.3 Hz, 1H), 7.69 (t, *J* = 7.3 Hz, 1H), 7.55 (t, *J* = 7.5 Hz, 1H), 7.23 (d, *J* = 4.4 Hz, 1H), 5.86 (ddt, *J* = 16.9, 10.2, 6.7 Hz, 1H), 5.12 – 4.99 (m, 2H), 2.19 (q, *J* = 7.1 Hz, 2H), 1.85 (t, *J* = 7.3 Hz, 2H); **^{13}C NMR (101 MHz, $CDCl_3$)** δ 150.1, 148.3, 148.2, 137.9, 130.2, 129.0, 127.5, 126.2, 123.5, 120.8, 115.4, 33.4, 29.0; **ATR-FTIR (cm $^{-1}$):** 3074, 2927, 2861, 1590, 1507, 1261, 911, 829, 759, 429; **HRMS m/z (ESI)** calculated for $C_{14}H_{14}D_2N^+$ $[M+H]^+$ 200.1403, found 200.1408.

The characterization data of the corresponding non-deuterated product are as follows:

4-(pent-4-en-1-yl)quinoline (5af')

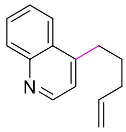

^{1}H NMR (400 MHz, $CDCl_3$) δ 8.80 (d, *J* = 4.4 Hz, 1H), 8.11 (d, *J* = 8.3 Hz, 1H), 8.02 (d, *J* = 8.4 Hz, 1H), 7.69 (ddd, *J* = 8.3, 6.9, 1.3 Hz, 1H), 7.55 (ddd, *J* = 8.2, 6.9, 1.1 Hz, 1H), 7.22 (d, *J* = 4.4 Hz, 1H), 5.86 (ddt, *J* = 16.9, 10.2, 6.7 Hz, 1H), 5.13 – 4.99 (m, 2H), 3.10 – 3.03 (m, 2H), 2.19 (q, *J* = 7.2 Hz, 2H), 1.92 – 1.80 (m, 2H); **^{13}C NMR (101 MHz, $CDCl_3$)** δ 150.1, 148.3, 148.2, 137.9, 130.2, 128.9, 127.5, 126.2, 123.5, 120.8, 115.4, 33.5, 31.4, 29.1; **ATR-FTIR (cm $^{-1}$):** 3074, 2931, 2863, 1591, 1508, 1257, 910, 834, 758, 431; **HRMS m/z (ESI)** calculated for $C_{14}H_{16}N^+$ $[M+H]^+$ 198.1277, found 198.1280.

6-(pent-4-en-1-yl-1,1-*d*$_2$)isoquinoline (5ag)

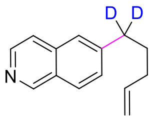

According to General procedure D, the crude product was purified by flash column chromatography on silica gel (PE : EA = 4:1) to afford **5ag** (25.7 mg, 65% yield,

96% D) as a colorless oil: **^{1}H NMR (400 MHz, CDCl$_3$)** δ 9.19 (s, 1H), 8.48 (d, *J* = 5.7 Hz, 1H), 7.88 (d, *J* = 8.4 Hz, 1H), 7.57 (d, *J* = 6.2 Hz, 2H), 7.45 (d, *J* = 8.4 Hz, 1H), 5.84 (ddt, *J* = 16.9, 10.2, 6.6 Hz, 1H), 5.12 – 4.92 (m, 2H), 2.19 – 2.07 (m, 2H), 1.80 (t, *J* = 7.3 Hz, 2H); **^{13}C NMR (101 MHz, CDCl$_3$)** δ 152.1, 145.0, 143.0, 138.2, 136.1, 128.8, 127.5, 127.4, 124.9, 120.1, 115.0, 33.1, 30.0; **ATR-FTIR (cm $^{-1}$):** 3052, 2925, 2856, 1630, 1491, 1279, 907, 826, 631, 464; **HRMS m/z (ESI)** calculated for $C_{14}H_{14}D_2N^+$ $[M+H]^+$ 200.1403, found 200.1408.

The characterization data of the corresponding non-deuterated product are as follows:

6-(pent-4-en-1-yl)isoquinoline (5ag')

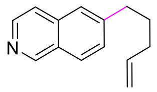

^{1}H NMR (400 MHz, CDCl$_3$) δ 9.19 (s, 1H), 8.48 (d, *J* = 5.5 Hz, 1H), 7.89 (d, *J* = 8.4 Hz, 1H), 7.58 (d, *J* = 6.1 Hz, 2H), 7.46 (d, *J* = 8.3 Hz, 1H), 5.85 (ddt, *J* = 16.9, 10.2, 6.6 Hz, 1H), 5.14 – 4.91 (m, 2H), 2.91 – 2.70 (m, 2H), 2.14 (q, *J* = 7.1 Hz, 2H), 1.91 – 1.75 (m, 2H); **^{13}C NMR (101 MHz, CDCl$_3$)** δ 152.1, 145.1, 143.0, 138.2, 136.1, 128.8, 127.6, 127.4, 124.9, 120.1, 115.0, 35.6, 33.2, 30.2; **ATR-FTIR (cm $^{-1}$):** 3053, 2926, 2856, 1630, 1494, 1275, 910, 828, 637, 469; **HRMS m/z (ESI)** calculated for $C_{14}H_{16}N^+$ $[M+H]^+$ 198.1277, found 198.1281.

1-methyl-5-(pent-4-en-1-yl-1,1-*d*$_2$)-1H-indazole (5ah)

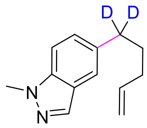

According to General procedure D, the crude product was purified by flash column chromatography on silica gel (PE : EA = 7:1) to afford **5ah** (25.8 mg, 64% yield, 96% D) as a colorless oil: **^{1}H NMR (400 MHz, CDCl$_3$)** δ 7.91 (d, *J* = 0.7 Hz, 1H), 7.49 (dd, *J* = 1.3, 0.8 Hz, 1H), 7.31 (d, *J* = 8.6 Hz, 1H), 7.23 (dd, *J* = 8.6, 1.5 Hz, 1H), 5.85 (ddt, *J* = 16.9, 10.2, 6.6 Hz, 1H), 5.12 – 4.93 (m, 2H), 4.05 (s, 3H), 2.17 – 2.03 (m, 2H), 1.75 (t, *J* = 7.4 Hz, 2H); **^{13}C NMR (101 MHz, CDCl$_3$)** δ 138.8, 138.6, 134.4, 132.1, 127.6, 124.3, 119.5, 114.6, 108.6, 35.4, 33.1, 30.8; **ATR-FTIR (cm $^{-1}$):** 3075, 2924, 2855, 1639, 1508, 1451, 1407, 1222, 1151, 988, 909, 795, 605, 431; **HRMS m/z (ESI)** calculated for $C_{13}H_{15}D_2N_2^+$ $[M+H]^+$ 203.1512, found 203.1513.

The characterization data of the corresponding non-deuterated product are as follows:

1-methyl-5-(pent-4-en-1-yl)-1H-indazole (5ah')

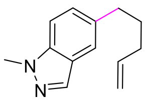

^{1}H NMR (400 MHz, CDCl$_3$) δ 7.91 (d, *J* = 0.6 Hz, 1H), 7.50 (d, *J* = 0.5 Hz, 1H), 7.31 (d, *J* = 8.6 Hz, 1H), 7.24 (dd, *J* = 8.6, 1.4 Hz, 1H), 5.85 (ddt, *J* = 16.9, 10.2, 6.6 Hz, 1H), 5.09 – 4.94 (m, 2H), 4.05 (s, 3H), 2.77 – 2.69 (m, 2H), 2.11 (q, *J* = 6.9 Hz, 2H), 1.81 – 1.71 (m, 2H); **^{13}C NMR (101 MHz, CDCl$_3$)** δ 138.8, 138.6, 134.5, 132.1, 127.7, 124.3, 119.6, 114.7, 108.6, 35.5, 35.1, 33.2, 31.0; **ATR-FTIR (cm $^{-1}$):** 3075, 2927, 2855, 1640, 1509, 1453, 1407, 1224, 1140, 988, 909, 798, 606, 431; **HRMS m/z (ESI)** calculated for $C_{13}H_{17}N_2^+$ [M+H]$^+$ 201.1386, found 201.1390.

5-(pent-4-en-1-yl-1,1-*d*$_2$)pyrimidine-2-carbonitrile (5ai)

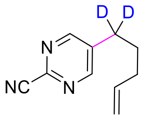

According to General procedure D, the crude product was purified by flash column chromatography on silica gel (PE : EA = 6:1) to afford **4ai** (15.2 mg, 43% yield, 96% D) as a colorless oil: **^{1}H NMR (400 MHz, CDCl$_3$)** δ 8.66 (s, 2H), 5.78 (ddt, *J* = 18.0, 9.5, 6.7 Hz, 1H), 5.16 – 4.96 (m, 2H), 2.21 – 2.02 (m, 2H), 1.76 (t, *J* = 7.2 Hz, 2H); **^{13}C NMR (101 MHz, CDCl$_3$)** δ 157.7, 143.0, 138.3, 137.0, 116.0, 115.7, 32.8, 29.2; **ATR-FTIR (cm $^{-1}$):** 3078, 2926, 2857, 2243, 1547, 1414, 1261, 993, 914, 791, 657, 550; **HRMS m/z (ESI)** calculated for $C_{10}H_{10}D_2N_3^+$ [M+H]$^+$ 176.1151, found 176.1149.

The characterization data of the corresponding non-deuterated product are as follows:

5-(pent-4-en-1-yl)pyrimidine-2-carbonitrile (5ai')

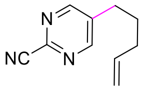

^{1}H NMR (400 MHz, CDCl$_3$) δ 8.66 (s, 2H), 5.89 – 5.64 (m, 1H), 5.11 – 4.94 (m, 2H), 2.76 – 2.63 (m, 2H), 2.13 (q, *J* = 7.1 Hz, 2H), 1.83 – 1.71 (m, 2H); **^{13}C NMR (101 MHz, CDCl$_3$)** δ 157.7, 143.0, 138.4, 137.0, 116.0, 115.7, 32.8, 29.7, 29.3; **ATR-FTIR (cm $^{-1}$):** 3078, 2931, 2860, 2244, 1552, 1413, 1267, 994, 914, 792, 661, 552; **HRMS m/z (ESI)** calculated for $C_{10}H_{12}N_3^+$ [M+H]$^+$ 174.1026, found 174.1022.

1-(3-oxo-8-azabicyclo[3.2.1]octan-8-yl)-4-(4-(pent-4-en-1-yl-1,1-*d*$_2$)phenyl)butane-1,4-dione (5aj)

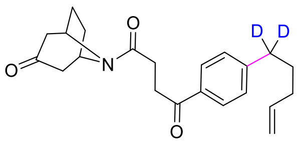

According to General procedure D, the crude product was purified by flash column chromatography on silica gel (PE : EA = 1:2) to afford

5aj (48.3 mg, 68% yield, 96% D) as a colorless oil: **^{1}H NMR (400 MHz, $CDCl_3$)** δ 7.91 (d, *J* = 8.3 Hz, 2H), 7.25 (d, *J* = 8.2 Hz, 2H), 5.80 (ddt, *J* = 16.9, 10.2, 6.6 Hz, 1H), 5.09 – 4.94 (m, 2H), 4.93 – 4.87 (m, 1H), 4.65 – 4.57 (m, 1H), 3.49 (dt, *J* = 18.1, 6.7 Hz, 1H), 3.31 (dt, *J* = 18.1, 6.0 Hz, 1H), 2.93 – 2.76 (m, 3H), 2.72 (dd, *J* = 15.9, 3.2 Hz, 1H), 2.46 (d, *J* = 15.9 Hz, 1H), 2.36 (d, *J* = 15.9 Hz, 1H), 2.31 – 2.13 (m, 1H), 2.13 – 1.98 (m, 3H), 1.90 – 1.75 (m, 1H), 1.70 (t, *J* = 7.3 Hz, 3H); **^{13}C NMR (101 MHz, $CDCl_3$)** δ 207.6, 198.5, 168.6, 148.3, 138.2, 134.4, 128.6, 128.2, 115.0, 53.7, 51.2, 49.5, 48.9, 33.3, 33.0, 30.0, 29.9, 27.8, 27.5; **ATR-FTIR (cm $^{-1}$):** 3075, 2922, 1713, 1639, 1431, 1361, 1245, 1181, 991, 907, 750, 559; **HRMS m/z (ESI)** calculated for $C_{22}H_{26}D_2NO_3^+$ $[M+H]^+$ 356.2189, found 356.2197.

The characterization data of the corresponding non-deuterated product are as follows:

1-(3-oxo-8-azabicyclo[3.2.1]octan-8-yl)-4-(4-(pent-4-en-1-yl)phenyl)butane-1,4-dione (5aj')

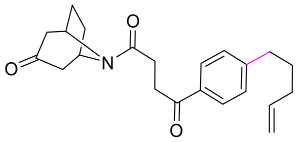

^{1}H NMR (400 MHz, $CDCl_3$) δ 7.92 (d, *J* = 8.2 Hz, 2H), 7.26 (d, *J* = 8.1 Hz, 2H), 5.81 (ddt, *J* = 16.9, 10.2, 6.6 Hz, 1H), 5.08 – 4.95 (m, 2H), 4.94 – 4.88 (m, 1H), 4.66 – 4.55 (m, 1H), 3.49 (dt, *J* = 18.1, 6.7 Hz, 1H), 3.31 (dt, *J* = 18.1, 6.0 Hz, 1H), 2.93 – 2.77 (m, 3H), 2.73 (dd, *J* = 15.9, 3.2 Hz, 1H), 2.70 – 2.64 (m, 2H), 2.46 (d, *J* = 15.9 Hz, 1H), 2.37 (d, *J* = 15.9 Hz, 1H), 2.29 – 2.14 (m, 1H), 2.12 – 1.99 (m, 3H), 1.89 – 1.77 (m, 1H), 1.77 – 1.64 (m, 3H); **^{13}C NMR (101 MHz, $CDCl_3$)** δ 207.6, 198.6, 168.6, 148.4, 138.2, 134.5, 128.6, 128.2, 115.0, 53.7, 51.2, 49.6, 48.9, 35.2, 33.3, 33.1, 30.2, 30.0, 27.9, 27.5; **ATR-FTIR (cm $^{-1}$):** 3074, 2924, 1713, 1638, 1432, 1362, 1246, 1179, 990, 908, 751, 562; **HRMS m/z (ESI)** calculated for $C_{22}H_{28}NO_3^+$ $[M+H]^+$ 354.2064, found 354.2074.

methyl (S)-2-((((9H-fluoren-9-yl)methoxy)carbonyl)amino)-3-(4-(pent-4-en-1-yl-1,1-*d*$_2$)phenyl)propanoate (5ak)

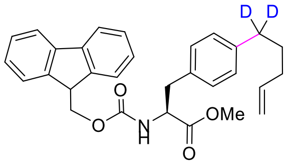

According to General procedure D, the crude product was purified by flash column chromatography on silica gel (PE : EA = 4:1) to afford **5ak** (40.7 mg, 43% yield, 96% D) as a colorless oil: **^{1}H NMR (400 MHz, $CDCl_3$)** δ 7.78 (d, *J* = 7.5 Hz, 2H), 7.58 (t, *J* = 7.4 Hz, 2H), 7.41 (t, *J* = 7.4 Hz, 2H), 7.32 (tt, *J* = 7.4, 1.3 Hz, 2H), 7.11 (d, *J* = 7.8 Hz, 2H), 7.02 (d, *J* = 7.8 Hz, 2H), 5.83 (ddt, *J* = 16.9, 10.2, 6.6 Hz, 1H), 5.28 (d, *J* = 8.1 Hz, 1H), 5.09 – 4.92 (m, 2H), 4.67 (q, *J* = 5.9 Hz, 1H), 4.44 (dd, *J* = 10.6, 7.1

Hz, 1H), 4.35 (dd, J = 10.4, 7.1 Hz, 1H), 4.21 (t, J = 7.0 Hz, 1H), 3.74 (s, 3H), 3.19 – 3.01 (m, 2H), 2.09 (q, J = 6.9 Hz, 2H), 1.69 (t, J = 7.3 Hz, 2H); **^{13}C NMR (101 MHz, CDCl$_3$)** δ 172.0, 155.5, 143.8, 141.3, 138.5, 132.9, 129.2, 128.6, 127.7, 127.0, 125.1, 125.0, 120.0, 114.7, 67.0, 54.8, 52.3, 47.2, 37.8, 33.2, 30.3; **ATR-FTIR (cm $^{-1}$):** 3334, 3065, 2925, 2855, 1716, 1512, 1447, 1210, 1051, 912, 740, 510, 427; **HRMS m/z (ESI)** calculated for $C_{30}H_{30}D_2NO_4^+$ [M+H]$^+$ 472.2451, found 472.2447.

The characterization data of the corresponding non-deuterated product are as follows:

methyl (S)-2-((((9H-fluoren-9-yl)methoxy)carbonyl)amino)-3-(4-(pent-4-en-1-yl)phenyl)propanoate (5ak')

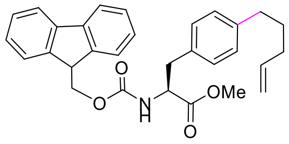

^{1}H NMR (400 MHz, CDCl$_3$) δ 7.79 (d, J = 7.5 Hz, 2H), 7.59 (t, J = 7.6 Hz, 2H), 7.42 (t, J = 7.4 Hz, 2H), 7.33 (tt, J = 7.4, 1.3 Hz, 2H), 7.12 (d, J = 7.8 Hz, 2H), 7.04 (d, J = 7.8 Hz, 2H), 5.85 (ddt, J = 16.9, 10.2, 6.6 Hz, 1H), 5.34 (d, J = 8.2 Hz, 1H), 5.12 – 4.93 (m, 2H), 4.75 – 4.63 (m, 1H), 4.46 (dd, J = 10.6, 7.1 Hz, 1H), 4.36 (dd, J = 10.5, 7.1 Hz, 1H), 4.23 (t, J = 7.0 Hz, 1H), 3.75 (s, 3H), 3.21 – 3.02 (m, 2H), 2.67 – 2.55 (m, 2H), 2.10 (q, J = 7.0 Hz, 2H), 1.83 – 1.62 (m, 2H); **^{13}C NMR (101 MHz, CDCl$_3$)** δ 172.1, 155.6, 143.8, 141.4, 138.6, 133.0, 129.3, 128.7, 127.8, 127.1, 125.2, 125.1, 120.0, 114.8, 67.0, 54.9, 52.4, 47.2, 37.9, 34.9, 33.3, 30.5; **ATR-FTIR (cm $^{-1}$):** 3339, 3064, 2928, 2855, 1718, 1511, 1448, 1179, 1051, 912, 734, 510, 426; **HRMS m/z (ESI)** calculated for $C_{30}H_{32}NO_4^+$ [M+H]$^+$ 470.2326, found 470.2325.

(3a*S*,5*S*,6*R*,6a*S*)-5-((*S*)-2,2-dimethyl-1,3-dioxolan-4-yl)-2,2-dimethyltetrahydrofuro[2,3-*d*][1,3]dioxol-6-yl 4-(pent-4-en-1-yl-1,1-*d*$_2$)benzoate (5al)

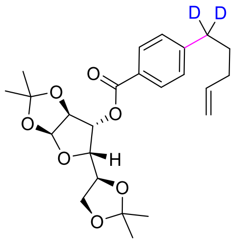

According to General procedure D, the crude product was purified by flash column chromatography on silica gel (PE : EA = 5:1) to afford **5al** (66.1 mg, 76% yield, 96% D) as a colorless oil: **^{1}H NMR (400 MHz, CDCl$_3$)** δ 7.94 (d, J = 8.4 Hz, 2H), 7.26 (d, J = 8.4 Hz, 2H), 5.94 (d, J = 3.7 Hz, 1H), 5.82 (ddt, J = 16.9, 10.2, 6.6 Hz, 1H), 5.49 (d, J = 2.6 Hz, 1H), 5.08 – 4.94 (m, 2H), 4.62 (d, J = 3.7 Hz, 1H), 4.42 – 4.29 (m, 2H), 4.19 – 4.03 (m, 2H), 2.15 – 2.02 (m, 2H), 1.72 (t, J = 7.4 Hz, 2H), 1.55 (s, 3H), 1.42 (s, 3H), 1.32 (s, 3H), 1.27 (s, 3H); **^{13}C NMR (101 MHz,**

$CDCl_3$) δ 165.2, 148.6, 138.1, 129.8, 128.6, 127.1, 115.0, 112.3, 109.3, 105.1, 83.4, 80.0, 76.4, 72.6, 67.2, 33.1, 30.0, 26.8, 26.7, 26.2, 25.2; **ATR-FTIR (cm^{-1}):** 3075, 2987, 2933, 1723, 1611, 1373, 1265, 1165, 1074, 1019, 844, 750, 513; **HRMS m/z (ESI)** calculated for $C_{24}H_{30}D_2O_7Na^+$ $[M+Na]^+$ 457.2166, found 457.2165.

The characterization data of the corresponding non-deuterated product are as follows:

(3a*S*,5*S*,6*R*,6a*S*)-5-((*S*)-2,2-dimethyl-1,3-dioxolan-4-yl)-2,2-dimethyltetrahydrofuro[2,3-*d*][1,3]dioxol-6-yl 4-(pent-4-en-1-yl)benzoate (5al')

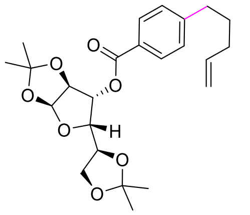

^{1}H NMR (400 MHz, $CDCl_3$) δ 7.94 (d, J = 8.2 Hz, 2H), 7.26 (d, J = 8.1 Hz, 2H), 5.94 (d, J = 3.6 Hz, 1H), 5.82 (ddt, J = 16.9, 10.2, 6.6 Hz, 1H), 5.49 (d, J = 2.5 Hz, 1H), 5.08 – 4.95 (m, 2H), 4.62 (d, J = 3.6 Hz, 1H), 4.42 – 4.29 (m, 2H), 4.16 – 4.05 (m, 2H), 2.75 – 2.64 (m, 2H), 2.09 (q, J = 7.1 Hz, 2H), 1.78 – 1.69 (m, 2H), 1.56 (s, 3H), 1.42 (s, 3H), 1.32 (s, 3H), 1.27 (s, 3H); **^{13}C NMR (101 MHz, $CDCl_3$)** δ 165.2, 148.7, 138.1, 129.8, 128.6, 127.1, 115.0, 112.3, 109.3, 105.1, 83.4, 80.0, 76.4, 72.6, 67.2, 35.3, 33.1, 30.2, 26.8, 26.7, 26.2, 25.2; **ATR-FTIR (cm^{-1}):** 3075, 2987, 2934, 1722, 1611, 1373, 1263, 1164, 1073, 1017, 842, 760, 511; **HRMS m/z (ESI)** calculated for $C_{24}H_{32}O_7Na^+$ $[M+Na]^+$ 455.2040, found 455.2032.

4-((2*S*,3*R*)-1-(4-fluorophenyl)-3-((*S*)-3-(4-fluorophenyl)-3-hydroxypropyl)-4-oxoazetidin-2-yl)phenyl 4-(pent-4-en-1-yl-1,1-*d*$_2$)benzoate (5am)

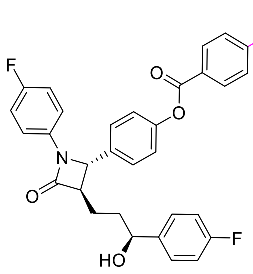

According to General procedure D, the crude product was purified by flash column chromatography on silica gel (PE : EA = 3:1) to afford **5am** (68.6 mg, 59% yield, 96% D) as a colorless oil: **^{1}H NMR (400 MHz, $CDCl_3$)** δ 8.09 (d, J = 8.2 Hz, 2H), 7.38 (d, J = 8.5 Hz, 2H), 7.33 – 7.28 (m, 4H), 7.27 – 7.20 (m, 4H), 7.02 (t, J = 8.7 Hz, 2H), 6.95 (t, J = 8.7 Hz, 2H), 5.83 (ddt, J = 16.9, 10.2, 6.6 Hz, 1H), 5.15 – 4.95 (m, 2H), 4.72 (s, 1H), 4.66 (d, J = 2.1 Hz, 1H), 3.12 (dt, J = 7.7, 3.6 Hz, 1H), 2.28 (d, J = 3.2 Hz, 1H), 2.11 (q, J = 7.0 Hz, 2H), 2.06 – 1.86 (m, 4H), 1.75 (t, J = 7.4 Hz, 2H); **^{13}C NMR (101 MHz, $CDCl_3$)** δ 167.4, 165.1, 162.2 (d, J = 245.5 Hz), 159.1 (d, J = 243.7 Hz), 151.2, 149.1, 140.0 (d, J = 2.6 Hz), 138.2, 135.0, 133.8 (d, J = 2.0 Hz), 130.3, 128.8, 127.4 (d, J = 8.0 Hz), 127.0, 126.8, 122.7, 118.4 (d, J = 7.7 Hz), 115.9 (d,

J = 22.7 Hz), 115.4 (d, J = 21.3 Hz), 115.1, 73.1, 60.9, 60.4, 36.6, 33.1, 30.1, 25.0; **^{19}F NMR (376 MHz, CDCl$_3$)** δ -114.9, -117.8; **ATR-FTIR (cm $^{-1}$):** 3437, 2926, 2859, 1731, 1607, 1507, 1386, 1264, 1199, 1177, 1066, 1015, 832, 735, 516; **HRMS m/z (ESI)** calculated for $C_{36}H_{31}D_2F_2NO_4Na^+$ [M+Na]$^+$ 606.2395, found 606.2386.

The characterization data of the corresponding non-deuterated product are as follows:

4-((2*S*,3*R*)-1-(4-fluorophenyl)-3-((*S*)-3-(4-fluorophenyl)-3-hydroxypropyl)-4-oxoazetidin-2-yl)phenyl 4-(pent-4-en-1-yl)benzoate (5am')

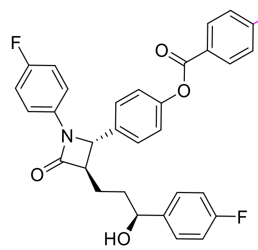

^{1}H NMR (400 MHz, CDCl$_3$) δ 8.09 (d, J = 8.2 Hz, 2H), 7.37 (d, J = 8.5 Hz, 2H), 7.34 – 7.26 (m, 4H), 7.26 – 7.20 (m, 4H), 7.02 (t, J = 8.7 Hz, 2H), 6.94 (t, J = 8.7 Hz, 2H), 5.83 (ddt, J = 16.9, 10.2, 6.6 Hz, 1H), 5.12 – 4.95 (m, 2H), 4.72 (t, J = 5.8 Hz, 1H), 4.65 (d, J = 2.2 Hz, 1H), 3.18 – 3.06 (m, 1H), 2.79 – 2.65 (m, 2H), 2.40 (s, 1H), 2.11 (q, J = 7.0 Hz, 2H), 2.06 – 1.86 (m, 4H), 1.82 – 1.70 (m, 2H); **^{13}C NMR (101 MHz, CDCl$_3$)** δ 167.4, 165.0, 162.2 (d, J = 245.6 Hz), 159.0 (d, J = 243.6 Hz), 151.2, 149.1, 140.0 (d, J = 3.1 Hz), 138.1, 135.0, 133.7 (d, J = 2.5 Hz), 130.3, 128.7, 127.4 (d, J = 8.0 Hz), 126.9, 126.8, 122.6, 118.4 (d, J = 7.8 Hz), 115.9 (d, J = 22.7 Hz), 115.3 (d, J = 21.4 Hz), 115.1, 73.0, 60.9, 60.4, 36.6, 35.3, 33.1, 30.2, 25.0; **^{19}F NMR (376 MHz, CDCl$_3$)** δ -114.9, -117.8; **ATR-FTIR (cm $^{-1}$):** 3437, 2927, 2859, 1733, 1607, 1508, 1386, 1265, 1217, 1177, 1068, 1016, 834, 758, 520; **HRMS m/z (ESI)** calculated for $C_{36}H_{33}F_2NO_4Na^+$ [M+Na]$^+$ 604.2270, found 604.2260.

methyl 2-(5-methoxy-2-methyl-1-(4-(pent-4-en-1-yl-1,1-*d*$_2$)benzoyl)-1H-indol-3-yl)acetate (5an)

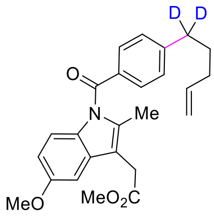

According to General procedure D, the crude product was purified by flash column chromatography on silica gel (PE : EA = 5:1) to afford **5an** (56.3 mg, 69% yield, 96% D) as a yellow oil: **^{1}H NMR (400 MHz, CDCl$_3$)** δ 7.63 (d, J = 8.0 Hz, 2H), 7.28 (d, J = 8.0 Hz, 2H), 6.96 (d, J = 2.2 Hz, 1H), 6.89 (d, J = 9.0 Hz, 1H), 6.65 (dd, J = 9.0, 2.2 Hz, 1H), 5.83 (ddt, J = 16.9, 10.1, 6.7 Hz, 1H), 5.10 – 4.95 (m, 2H), 3.83 (s, 3H), 3.70 (s, 3H), 3.67 (s, 2H), 2.38 (s, 3H), 2.11 (q, J = 7.1 Hz, 2H), 1.76 (t, J = 7.3 Hz, 2H); **^{13}C NMR (101 MHz, CDCl$_3$)** δ 171.4, 169.4, 155.9, 148.1, 138.1, 136.1, 133.1, 131.1,

130.5, 130.0, 128.8, 115.1, 115.0, 111.9, 111.4, 101.1, 55.7, 52.1, 33.1, 30.2, 30.0, 13.2; **ATR-FTIR (cm $^{-1}$):** 3075, 2927, 2855, 1736, 1678, 1607, 1477, 1311, 1222, 1142, 1066, 911, 832, 750, 600; **HRMS m/z (ESI)** calculated for $C_{25}H_{26}D_2NO_4^+$ $[M+H]^+$ 408.2138, found 408.2146.

The characterization data of the corresponding non-deuterated product are as follows:

methyl 2-(5-methoxy-2-methyl-1-(4-(pent-4-en-1-yl)benzoyl)-1H-indol-3-yl)acetate (5an')

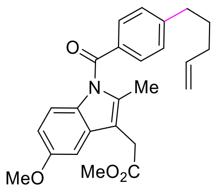

^{1}H NMR (400 MHz, $CDCl_3$) δ 7.63 (d, *J* = 8.1 Hz, 2H), 7.29 (d, *J* = 8.1 Hz, 2H), 6.96 (d, *J* = 2.4 Hz, 1H), 6.89 (d, *J* = 9.0 Hz, 1H), 6.65 (dd, *J* = 9.0, 2.5 Hz, 1H), 5.83 (ddt, *J* = 16.9, 10.2, 6.7 Hz, 1H), 5.15 – 4.89 (m, 2H), 3.83 (s, 3H), 3.70 (s, 3H), 3.67 (s, 2H), 2.80 – 2.65 (m, 2H), 2.38 (s, 3H), 2.12 (q, *J* = 7.1 Hz, 2H), 1.84 – 1.70 (m, 2H); **^{13}C NMR (101 MHz, $CDCl_3$)** δ 171.4, 169.4, 155.8, 148.1, 138.1, 136.1, 133.0, 131.0, 130.4, 130.0, 128.8, 115.1, 115.0, 111.8, 111.4, 101.0, 55.7, 52.1, 35.3, 33.1, 30.2, 13.2; **ATR-FTIR (cm $^{-1}$):** 3074, 2928, 2855, 1736, 1678, 1606, 1477, 1310, 1222, 1141, 1066, 911, 833, 758, 602; **HRMS m/z (ESI)** calculated for $C_{25}H_{28}NO_4^+$ $[M+H]^+$ 406.2013, found 406.2020.

***N*-(2,6-dimethoxypyrimidin-4-yl)-*N*-methyl-4-(pent-4-en-1-yl-1,1-d_2)benzenesulfonamide (5ao)**

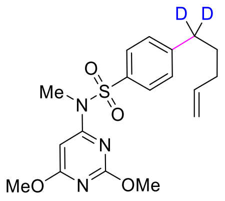

According to General procedure D, the crude product was purified by flash column chromatography on silica gel (PE : EA = 5:1) to afford **5ao** (50.3 mg, 66% yield, 96% D) as a colorless oil: **^{1}H NMR (400 MHz, $CDCl_3$)** δ 7.69 (d, *J* = 8.5 Hz, 2H), 7.27 (d, *J* = 8.4 Hz, 2H), 6.64 (s, 1H), 5.78 (ddt, *J* = 16.9, 10.2, 6.6 Hz, 1H), 5.11 – 4.88 (m, 2H), 3.91 (s, 3H), 3.84 (s, 3H), 3.44 (s, 3H), 2.16 – 1.99 (m, 2H), 1.68 (t, *J* = 7.4 Hz, 2H); **^{13}C NMR (101 MHz, $CDCl_3$)** δ 172.5, 164.2, 161.4, 148.6, 137.9, 135.9, 129.1, 127.2, 115.1, 89.8, 54.6, 54.0, 34.4, 33.0, 29.8; **ATR-FTIR (cm $^{-1}$):** 3075, 2933, 2859, 1567, 1457, 1347, 1206, 1158, 1089, 1028, 975, 779, 680, 565; **HRMS m/z (ESI)** calculated for $C_{18}H_{22}D_2N_3O_4S^+$ $[M+H]^+$ 380.1608, found 380.1616.

The characterization data of the corresponding non-deuterated product are as follows:

***N*-(2,6-dimethoxypyrimidin-4-yl)-*N*-methyl-4-(pent-4-en-1-yl)benzenesulfonamide (5ao')**

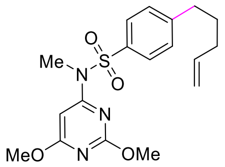

^{1}H NMR (400 MHz, $CDCl_3$) δ 7.70 (d, *J* = 8.3 Hz, 2H), 7.28 (d, *J* = 8.0 Hz, 2H), 6.65 (s, 1H), 5.80 (ddt, *J* = 16.9, 10.2, 6.6 Hz, 1H), 5.09 – 4.88 (m, 2H), 3.93 (s, 3H), 3.86 (s, 3H), 3.45 (s, 3H), 2.71 – 2.60 (m, 2H), 2.07 (q, *J* = 7.1 Hz, 2H), 1.77 – 1.66 (m, 2H); **^{13}C NMR (101 MHz, $CDCl_3$)** δ 172.5, 164.2, 161.4, 148.7, 137.9, 135.9, 129.1, 127.2, 115.2, 89.8, 54.6, 54.0, 35.1, 34.4, 33.0, 30.0; **ATR-FTIR (cm $^{-1}$):** 3074, 2929, 2859, 1567, 1456, 1348, 1207, 1159, 1090, 1028, 975, 774, 614, 560; **HRMS m/z (ESI)** calculated for $C_{18}H_{24}N_3O_4S^+$ $[M+H]^+$ 378.1482, found 378.1497.

2-(1-methyl-2,6-dioxopiperidin-3-yl)-4-(pent-4-en-1-yl-1,1-*d*$_2$)isoindoline-1,3-dione (5ap)

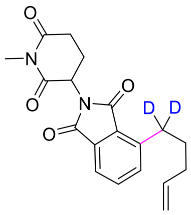

According to General procedure D, the crude product was purified by flash column chromatography on silica gel (PE : EA = 3:1) to afford **5ap** (31.6 mg, 46% yield, 96% D) as a colorless oil: **^{1}H NMR (400 MHz, $CDCl_3$)** δ 7.71 (d, *J* = 7.3 Hz, 1H), 7.62 (t, *J* = 7.5 Hz, 1H), 7.51 (d, *J* = 7.7 Hz, 1H), 5.83 (ddt, *J* = 16.9, 10.2, 6.6 Hz, 1H), 5.14 – 4.81 (m, 3H), 3.20 (s, 3H), 3.04 – 2.91 (m, 1H), 2.88 – 2.69 (m, 2H), 2.23 – 2.06 (m, 3H), 1.74 (t, *J* = 7.4 Hz, 2H); **^{13}C NMR (101 MHz, $CDCl_3$)** δ 171.1, 168.7, 167.9, 167.4, 143.2, 138.0, 135.8, 134.0, 132.3, 128.1, 121.5, 115.0, 49.8, 33.3, 31.9, 29.7, 27.2, 22.0; **ATR-FTIR (cm $^{-1}$):** 3075, 2926, 2859, 1708, 1676, 1388, 1287, 1115, 1029, 902, 733, 607, 477; **HRMS m/z (ESI)** calculated for $C_{19}H_{19}D_2N_2O_4^+$ $[M+H]^+$ 343.1621, found 343.1621.

The characterization data of the corresponding non-deuterated product are as follows:

2-(1-methyl-2,6-dioxopiperidin-3-yl)-4-(pent-4-en-1-yl)isoindoline-1,3-dione (5ap')

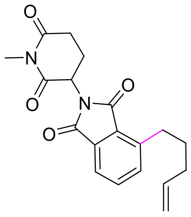

^{1}H NMR (400 MHz, $CDCl_3$) δ 7.70 (dd, *J* = 7.3, 0.9 Hz, 1H), 7.62 (t, *J* = 7.5 Hz, 1H), 7.51 (d, *J* = 7.6 Hz, 1H), 5.83 (ddt, *J* = 16.9, 10.2, 6.6 Hz, 1H), 5.09 – 4.89 (m, 3H), 3.20 (s, 3H), 3.15 – 3.01 (m, 2H), 3.02 – 2.91 (m, 1H), 2.89 – 2.67 (m, 2H), 2.23 – 2.06 (m, 3H), 1.82 – 1.69 (m, 2H); **^{13}C NMR (101 MHz, $CDCl_3$)** δ 171.1, 168.7, 167.9, 167.4, 143.2, 138.0, 135.8, 134.0, 132.3, 128.1, 121.5, 115.1, 49.8, 33.4, 31.8, 30.7, 29.8, 27.2, 21.9; **ATR-FTIR (cm $^{-1}$):** 3063, 2926, 2861, 1709, 1678, 1389, 1287, 1116, 1029, 909, 734, 610, 475; **HRMS m/z (ESI)** calculated for $C_{19}H_{21}N_2O_4^+$ $[M+H]^+$ 341.1496, found 341.1501.

4-(5-(4-(pent-4-en-1-yl-1,1-*d*$_2$)phenyl)-3-(trifluoromethyl)-1*H*-pyrazol-1-yl)benzenesulfonamide (5aq)

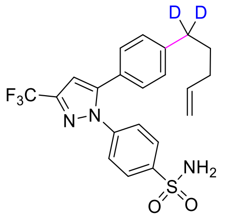

According to General procedure D, the crude product was purified by flash column chromatography on silica gel (PE : EA = 1:1) to afford **5aq** (37.5 mg, 43% yield, 96% D) as a colorless oil: **^{1}H NMR (400 MHz, CDCl$_3$)** δ 7.88 (d, *J* = 8.7 Hz, 2H), 7.46 (d, *J* = 8.7 Hz, 2H), 7.18 (d, *J* = 8.3 Hz, 2H), 7.13 (d, *J* = 8.3 Hz, 2H), 6.74 (s, 1H), 5.82 (ddt, *J* = 16.9, 10.2, 6.6 Hz, 1H), 5.16 (s, 2H), 5.10 – 4.86 (m, 2H), 2.17 – 1.99 (m, 2H), 1.78 – 1.67 (m, 2H); **^{13}C NMR (101 MHz, CDCl$_3$)** δ 145.2, 144.3, 144.1 (q, *J* = 36.9 Hz), 142.5, 141.4, 138.2, 129.1, 128.7, 127.4, 126.0, 125.5, 121.0 (q, *J* = 269.2 Hz), 115.0, 106.4, 33.1, 30.0; **^{19}F NMR (376 MHz, CDCl$_3$)** δ -62.4; **ATR-FTIR (cm $^{-1}$):** 3265, 3078, 2928, 2859, 1471, 1337, 1235, 1159, 976, 911, 841, 617, 563; **HRMS m/z (ESI)** calculated for $C_{21}H_{19}D_2F_3N_3O_2S^+$ [M+H]$^+$ 438.1427, found 438.1432.

The characterization data of the corresponding non-deuterated product are as follows:

4-(5-(4-(pent-4-en-1-yl)phenyl)-3-(trifluoromethyl)-1*H*-pyrazol-1-yl)benzenesulfonamide (5aq')

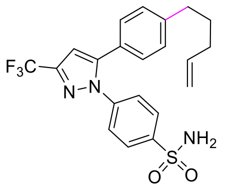

^{1}H NMR (400 MHz, CDCl$_3$) δ 7.88 (d, *J* = 8.8 Hz, 2H), 7.46 (d, *J* = 8.8 Hz, 2H), 7.18 (d, *J* = 8.2 Hz, 2H), 7.13 (d, *J* = 8.3 Hz, 2H), 6.74 (s, 1H), 5.82 (ddt, *J* = 16.9, 10.2, 6.6 Hz, 1H), 5.17 (s, 2H), 5.10 – 4.92 (m, 2H), 2.70 – 2.59 (m, 2H), 2.10 (q, *J* = 7.1 Hz, 2H), 1.82 – 1.68 (m, 2H); **^{13}C NMR (101 MHz, CDCl$_3$)** δ 145.2, 144.3, 144.1 (q, *J* = 38.6 Hz), 142.5, 141.4, 138.2, 129.1, 128.7, 127.4, 121.0 (d, *J* = 269.2 Hz), 119.7, 117.0, 115.0, 106.4, 35.0, 33.2, 30.2; **^{19}F NMR (376 MHz, CDCl$_3$)** δ -62.4; **ATR-FTIR (cm $^{-1}$):** 3267, 3076, 2932, 2860, 1471, 1337, 1235, 1159, 975, 911, 841, 615, 563; **HRMS m/z (ESI)** calculated for $C_{21}H_{21}F_3N_3O_2S^+$ [M+H]$^+$ 436.1301, found 436.1308.

7-(but-2-yn-1-yl)-3-methyl-1-((4-methylquinazolin-2-yl)methyl)-8-(pent-4-en-1-yl-1,1-*d*$_2$)-3,7-dihydro-1*H*-purine-2,6-dione (5ar)

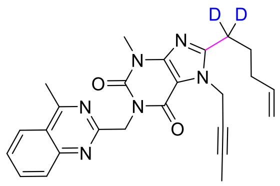

According to General procedure D, the crude product was purified by flash column chromatography on silica gel (DCM : MeOH = 50:1) to afford **5ar** (39.3 mg, 44% yield, 96% D) as a pale yellow solid: **^{1}H NMR (400 MHz, CDCl$_3$)** δ 8.00 (d, *J* = 8.3 Hz, 1H), 7.85 (d, *J* = 8.5 Hz, 1H), 7.74 (t, *J* = 7.7 Hz, 1H), 7.50 (t, *J* = 7.6 Hz, 1H), 5.85 (ddt, *J* = 16.9, 10.1, 6.6 Hz, 1H), 5.56 (s, 2H), 5.16 – 4.98 (m, 4H), 3.60 (s, 3H), 2.87 (s, 3H), 2.22 (q, *J* = 7.0 Hz, 2H), 1.95 (t, *J* = 7.1 Hz, 2H), 1.77 (s, 3H); **^{13}C NMR (101 MHz, CDCl$_3$)** δ 168.4, 160.9, 155.0, 154.4, 151.7, 149.9, 148.4, 137.5, 133.1, 128.9, 126.6, 124.8, 123.1, 115.5, 106.3, 81.8, 72.4, 46.3, 34.6, 33.2, 29.7, 26.4, 21.7, 3.5; **ATR-FTIR (cm $^{-1}$):** 3072, 2924, 2855, 1702, 1653, 1569, 1480, 1435, 1350, 1211, 1136, 914, 762; **HRMS m/z (ESI)** calculated for $C_{25}H_{25}D_2N_6O_2^+$ [M+H]$^+$ 445.2316, found 445.2322.

The characterization data of the corresponding non-deuterated product are as follows:

7-(but-2-yn-1-yl)-3-methyl-1-((4-methylquinazolin-2-yl)methyl)-8-(pent-4-en-1-yl)-3,7-dihydro-1*H*-purine-2,6-dione (5ar')

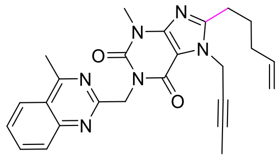

^{1}H NMR (400 MHz, CDCl$_3$) δ 8.00 (dd, *J* = 8.3, 0.7 Hz, 1H), 7.85 (d, *J* = 8.1 Hz, 1H), 7.74 (ddd, *J* = 8.4, 6.9, 1.4 Hz, 1H), 7.50 (ddd, *J* = 8.2, 6.9, 1.2 Hz, 1H), 5.85 (ddt, *J* = 16.9, 10.2, 6.6 Hz, 1H), 5.56 (s, 2H), 5.17 – 4.98 (m, 4H), 3.60 (s, 3H), 2.92 – 2.77 (m, 5H), 2.22 (dd, *J* = 14.2, 7.1 Hz, 2H), 2.01 – 1.92 (m, 2H), 1.77 (t, *J* = 2.4 Hz, 3H); **^{13}C NMR (101 MHz, CDCl$_3$)** δ 168.5, 160.9, 155.0, 154.4, 151.7, 149.9, 148.3, 137.5, 133.2, 128.9, 126.7, 124.8, 123.1, 115.5, 106.3, 81.8, 72.4, 46.3, 34.6, 33.2, 29.7, 26.6, 26.3, 21.7, 3.5; **ATR-FTIR (cm $^{-1}$):** 3072, 2949, 2854, 1703, 1657, 1569, 1482, 1430, 1349, 1214, 1133, 914, 763; **HRMS m/z (ESI)** calculated for $C_{25}H_{27}N_6O_2^+$ [M+H]$^+$ 443.2190, found 443.2188.

1,3,7-trimethyl-8-(pent-4-en-1-yl-1,1-*d*$_2$)-3,7-dihydro-1*H*-purine-2,6-dione (5as)

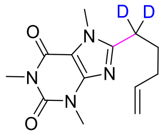

According to General procedure D, the crude product was purified by flash column chromatography on silica gel (DCM : MeOH = 50:1) to afford **5as** (31.8 mg, 60% yield, 96% D) as a white solid: **^{1}H NMR (400 MHz, DMSO-*d*$_6$)** δ 5.76 (ddt, *J* = 16.9, 10.2, 6.6 Hz, 1H), 5.07 – 4.83 (m, 2H), 3.72 (s, 3H), 3.27 (s, 3H), 3.08 (s, 3H), 2.10 – 1.98 (m, 2H), 1.67 (t, *J* = 7.4 Hz, 2H); **^{13}C NMR (101 MHz, DMSO-*d*$_6$)** δ 154.2, 154.0, 150.8, 147.3, 138.0,

115.4, 106.2, 32.5, 31.3, 29.3, 27.4, 25.7; **ATR-FTIR (cm $^{-1}$):** 3076, 2945, 2857, 1699, 1651, 1542, 1434, 1218, 1035, 978, 913, 746, 499; **HRMS m/z (ESI)** calculated for $C_{13}H_{17}D_2N_4O_2^+$ $[M+H]^+$ 265.1628, found 265.1635.

The characterization data of the corresponding non-deuterated product are as follows:

1,3,7-trimethyl-8-(pent-4-en-1-yl)-3,7-dihydro-1*H*-purine-2,6-dione (5as')

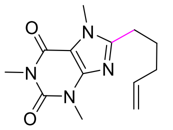

^{1}H NMR (400 MHz, DMSO-*d*$_6$) δ 5.84 (ddt, *J* = 16.9, 10.2, 6.6 Hz, 1H), 5.11 – 4.92 (m, 2H), 3.82 (s, 3H), 3.38 (s, 3H), 3.19 (s, 3H), 2.72 (t, *J* = 7.7 Hz, 2H), 2.12 (q, *J* = 7.1 Hz, 2H), 1.83 – 1.71 (M, 2H); **^{13}C NMR (101 MHz, DMSO-*d*$_6$)** δ 154.3, 154.1, 150.9, 147.3, 138.0, 115.4, 106.3, 32.6, 31.3, 29.3, 27.4, 25.9, 25.2; **ATR-FTIR (cm $^{-1}$):** 3076, 2929, 2853, 1699, 1651, 1543, 1430, 1219, 1037, 978, 913, 747, 503; **HRMS m/z (ESI)** calculated for $C_{13}H_{19}N_4O_2^+$ $[M+H]^+$ 263.1503, found 263.1509.

(*E*)-5-(hepta-1,6-dien-1-yl-3,3-*d*$_2$)-1,2,3-trimethoxybenzene (5at)

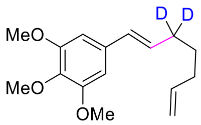

According to General procedure D, the crude product was purified by flash column chromatography on silica gel (PE : EA = 10:1) to afford **5at** (34.0 mg, 64% yield, 97% D) as a colorless oil: **^{1}H NMR (400 MHz, CDCl$_3$)** δ 6.57 (s, 2H), 6.31 (d, *J* = 15.7 Hz, 1H), 6.12 (d, *J* = 15.7 Hz, 1H), 5.83 (ddt, *J* = 16.9, 10.2, 6.6 Hz, 1H), 5.09 – 4.93 (m, 2H), 3.87 (s, 6H), 3.83 (s, 3H), 2.16 – 2.06 (m, 2H), 1.56 (t, *J* = 7.4 Hz, 2H); **^{13}C NMR (101 MHz, CDCl$_3$)** δ 153.3, 138.6, 137.4, 133.6, 130.1, 130.0, 114.6, 103.1, 60.9, 56.0, 33.1, 28.4; **ATR-FTIR (cm $^{-1}$):** 2926, 2837, 1580, 1505, 1415, 1337, 1234, 1123, 1007, 909, 832; **HRMS m/z (ESI)** calculated for $C_{16}H_{21}D_2O_3^+$ $[M+H]^+$ 265.1767, found 265.1758.

The characterization data of the corresponding non-deuterated product are as follows:

(*E*)-5-(hepta-1,6-dien-1-yl)-1,2,3-trimethoxybenzene (5at')

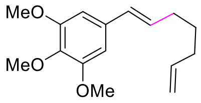

^{1}H NMR (400 MHz, CDCl$_3$) δ 6.57 (s, 2H), 6.31 (d, *J* = 15.8 Hz, 1H), 6.13 (dt, *J* = 15.7, 6.9 Hz, 1H), 5.83 (ddt, *J* = 16.9, 10.2, 6.6 Hz, 1H), 5.09 – 4.93 (m, 2H), 3.87 (s, 6H), 3.83 (s, 3H), 2.27 – 2.17 (m, 2H), 2.11 (q, *J* = 7.0 Hz, 2H), 1.62 – 1.53 (p, *J* = 7.5 Hz, 2H); **^{13}C NMR (101 MHz, CDCl$_3$)** δ 153.3, 138.5, 137.4, 133.6, 130.2, 130.0, 114.6, 103.1,

60.8, 56.0, 33.2, 32.3, 28.5; **ATR-FTIR (cm $^{-1}$):** 2929, 2834, 1580, 1505, 1416, 1324, 1236, 1123, 1007, 909, 838; **HRMS m/z (ESI)** calculated for $C_{16}H_{23}O_3^+$ $[M+H]^+$ 263.1642, found 263.1635.

(*E*)-4-(hepta-1,6-dien-1-yl-3,3-*d*$_2$)phenyl (tert-butoxycarbonyl)-*L*-phenylalaninate (5au)

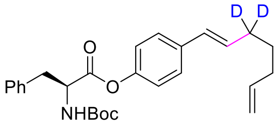

According to General procedure D, the crude product was purified by flash column chromatography on silica gel (PE : EA = 8:1) to afford **5au** (42.7 mg, 49% yield, 97% D) as a white solid: **^{1}H NMR (400 MHz, CDCl$_3$)** δ 7.37 – 7.27 (m, 5H), 7.26 – 7.21 (m, 2H), 6.91 (d, *J* = 8.6 Hz, 2H), 6.35 (d, *J* = 15.8 Hz, 1H), 6.15 (d, *J* = 15.8 Hz, 1H), 5.82 (ddt, *J* = 16.9, 10.2, 6.7 Hz, 1H), 5.14 – 4.92 (m, 3H), 4.87 – 4.73 (m, 1H), 3.23 (d, *J* = 5.7 Hz, 2H), 2.17 – 2.03 (m, 2H), 1.55 (t, *J* = 7.4 Hz, 2H), 1.44 (s, 9H); **^{13}C NMR (101 MHz, CDCl$_3$)** δ 170.5, 155.1, 149.1, 138.6, 135.9, 135.8, 131.1, 129.4, 129.1, 128.7, 127.2, 126.8, 121.2, 114.6, 80.1, 54.6, 38.4, 33.1, 28.3, 28.3; **ATR-FTIR (cm $^{-1}$):** 3392, 2926, 1713, 1504, 1366, 1162, 966, 860, 699; **HRMS m/z (ESI)** calculated for $C_{27}H_{32}D_2NO_4^+$ $[M+H]^+$438.2608, found 438.2606.

The characterization data of the corresponding non-deuterated product are as follows:

(*E*)-4-(hepta-1,6-dien-1-yl)phenyl (tert-butoxycarbonyl)-*L*-phenylalaninate (5au')

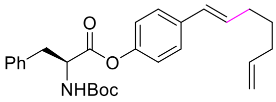

^{1}H NMR (400 MHz, CDCl$_3$) δ 7.37 – 7.26 (m, 5H), 7.26 – 7.21 (m, 2H), 6.91 (d, *J* = 8.6 Hz, 2H), 6.35 (d, *J* = 15.9 Hz, 1H), 6.16 (dt, *J* = 15.7, 6.9 Hz, 1H), 5.83 (ddt, *J* = 16.9, 10.2, 6.7 Hz, 1H), 5.11 – 4.92 (m, 3H), 4.87 – 4.73 (m, 1H), 3.23 (d, *J* = 5.7 Hz, 2H), 2.22 (q, *J* = 6.9 Hz, 2H), 2.10 (q, *J* = 7.0 Hz, 2H), 1.63 – 1.51 (m, 2H), 1.44 (s, 9H); **^{13}C NMR (101 MHz, CDCl$_3$)** δ 170.6, 155.1, 149.1, 138.6, 136.0, 135.8, 131.2, 129.5, 129.0, 128.7, 127.2, 126.8, 121.2, 114.7, 80.1, 54.6, 38.4, 33.2, 32.3, 28.5, 28.3; **ATR-FTIR (cm $^{-1}$):** 3436, 2928, 1713, 1504, 1366, 1162, 964, 859, 699; **HRMS m/z (ESI)** calculated for $C_{27}H_{34}NO_4^+$ $[M+H]^+$436.2482, found 436.2483.

(*E*)-4-(hepta-1,6-dien-1-yl-3,3-*d*$_2$)phenyl (*S*)-2-(6-methoxynaphthalen-2-yl)propanoate (5av)

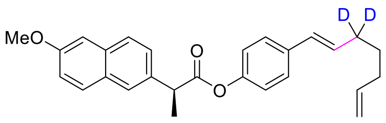

According to General procedure D, the crude product was purified by flash column chromatography on silica gel (PE :

EA = 10:1) to afford **5av** (47.5 mg, 59% yield, 97% D) as a white solid: **^{1}H NMR (400 MHz, CDCl$_3$)** δ 7.81 – 7.66 (m, 3H), 7.49 (d, *J* = 8.5 Hz, 1H), 7.26 (d, *J* = 9.8 Hz, 2H), 7.15 (d, *J* = 11.4 Hz, 2H), 6.90 (d, *J* = 8.3 Hz, 2H), 6.33 (d, *J* = 15.8 Hz, 1H), 6.12 (d, *J* = 15.8 Hz, 1H), 5.81 (ddt, *J* = 16.9, 10.2, 6.6 Hz, 1H), 4.98 (dd, *J* = 21.3, 13.7 Hz, 2H), 4.08 (q, *J* = 7.1 Hz, 1H), 3.92 (s, 3H), 2.09 (q, *J* = 7.1 Hz, 2H), 1.68 (d, *J* = 7.1 Hz, 3H), 1.57 – 1.48 (m, 2H); **^{13}C NMR (101 MHz, CDCl$_3$)** δ 173.1, 157.8, 149.7, 138.6, 135.6, 135.2, 133.8, 130.8, 129.3, 129.2, 129.0, 127.3, 126.7, 126.1, 121.3, 119.0, 114.6, 105.7, 55.3, 45.6, 33.1, 28.4, 18.5; **ATR-FTIR (cm $^{-1}$):** 2983, 2934, 1748, 1604, 1504, 1163, 1145, 1031, 853, 477; **HRMS m/z (ESI)** calculated for $C_{27}H_{27}D_2O_3^+$ $[M+H]^+$ 403.2237, found 403.2228.

The characterization data of the corresponding non-deuterated product are as follows:

(*E*)-4-(hepta-1,6-dien-1-yl)phenyl (*S*)-2-(6-methoxynaphthalen-2-yl)propanoate (5av')

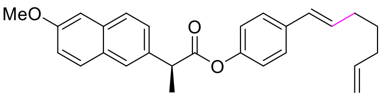

^{1}H NMR (400 MHz, CDCl$_3$) δ 7.82 – 7.71 (m, 3H), 7.52 (dd, *J* = 8.5, 1.8 Hz, 1H), 7.29 (d, *J* = 8.6 Hz, 2H), 7.22 – 7.13 (m, 2H), 6.93 (d, *J* = 8.6 Hz, 2H), 6.35 (d, *J* = 15.8 Hz, 1H), 6.15 (dt, *J* = 15.8, 6.9 Hz, 1H), 5.84 (ddt, *J* = 16.9, 10.1, 6.6 Hz, 1H), 5.07 – 4.92 (m, 2H), 4.10 (q, *J* = 7.1 Hz, 1H), 3.93 (s, 3H), 2.22 (q, *J* = 6.9 Hz, 2H), 2.11 (q, *J* = 7.0 Hz, 2H), 1.71 (d, *J* = 7.1 Hz, 3H), 1.62 – 1.49 (m, 2H); **^{13}C NMR (101 MHz, CDCl$_3$)** δ 173.1, 157.8, 149.7, 138.6, 135.6, 135.2, 133.8, 130.8, 129.3, 129.1, 129.0, 127.3, 126.7, 126.1, 121.3, 119.0, 114.6, 105.7, 55.3, 45.6, 33.2, 32.3, 28.5, 18.5; **ATR-FTIR (cm $^{-1}$):** 2976, 2931, 1752, 1605, 1505, 1164, 1131, 1032, 852, 475; **HRMS m/z (ESI)** calculated for $C_{27}H_{29}O_3^+$ $[M+H]^+$ 401.2111, found 401.2104.

5-(pent-4-en-1-yl-1,1-*d*$_2$)-1-phenylpyridin-2(1*H*)-one (5aw)

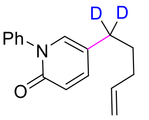

According to General procedure D, the crude product was purified by flash column chromatography on silica gel (PE : EA = 1:1) to afford **5aw** (19.8 mg, 41% yield, 96% D) as a colorless oil: **^{1}H NMR (400 MHz, CDCl$_3$)** δ 7.52 – 7.45 (m, 2H), 7.44 – 7.33 (m, 3H), 7.31 – 7.26 (m, 1H), 7.10 (s, 1H), 6.62 (d, *J* = 9.4 Hz, 1H), 5.89 – 5.68 (m, 1H), 5.08 – 4.91 (m, 2H), 2.09 (q, *J* = 7.0 Hz, 2H), 1.64 (t, *J* = 7.3 Hz, 2H); **^{13}C NMR (101 MHz, CDCl$_3$)** δ 161.8, 141.6, 141.1, 137.9, 135.2, 129.2, 128.3, 126.5, 121.6, 119.2, 115.1, 32.8, 29.2; **ATR-FTIR (cm $^{-1}$):** 3072, 2922, 2854, 1667, 1608, 1527, 1454, 1281, 993, 826, 756, 693, 585; **HRMS m/z**

(ESI) calculated for $C_{16}H_{16}D_2NO^+$ $[M+H]^+$ 242.1508, found 242.1517.

The characterization data of the corresponding non-deuterated product are as follows:

5-(pent-4-en-1-yl)-1-phenylpyridin-2(1*H*)-one (5aw')

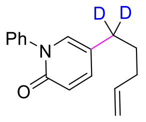

^{1}H NMR (400 MHz, $CDCl_3$) δ 7.53 – 7.45 (m, 2H), 7.44 – 7.35 (m, 3H), 7.29 (dd, J = 9.4, 2.5 Hz, 1H), 7.10 (d, J = 2.1 Hz, 1H), 6.63 (d, J = 9.4 Hz, 1H), 5.80 (ddt, J = 16.9, 10.2, 6.7 Hz, 1H), 5.10 – 4.90 (m, 2H), 2.47 – 2.29 (m, 2H), 2.10 (q, J = 7.1 Hz, 2H), 1.71 – 1.58 (m, 2H); **^{13}C NMR (101 MHz, $CDCl_3$)** δ 161.8, 141.6, 141.2, 137.9, 135.2, 129.3, 128.3, 126.6, 121.6, 119.3, 115.2, 32.9, 30.8, 29.4; **ATR-FTIR (cm $^{-1}$):** 3072, 2923, 2853, 1668, 1608, 1529, 1454, 1278, 992, 868, 756, 693, 588; **HRMS m/z (ESI)** calculated for $C_{16}H_{18}NO^+$ $[M+H]^+$ 240.1383, found 240.1388.

(3a*R*,5*R*,5a*S*,8a*S*,8b*R*)-5-((*E*)-hepta-1,6-dien-1-yl-3,3-*d*$_2$)-2,2,7,7-tetramethyltetrahydro-5*H*-bis([1,3]dioxolo)[4,5-*b*:4',5'-d]pyran (5ax)

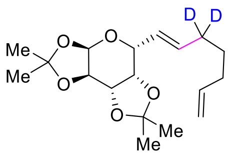

According to General procedure D, the crude product was purified by flash column chromatography on silica gel (PE : EA = 15:1) to afford **5ax** (45.0 mg, 69% yield, 97% D) as colorless oil: **^{1}H NMR (400 MHz, $CDCl_3$)** δ 5.86 – 5.70 (m, 2H), 5.60 (dd, J = 15.5, 7.1 Hz, 1H), 5.54 (d, J = 5.0 Hz, 1H), 5.05 – 4.89 (m, 2H), 4.58 (dd, J = 7.8, 2.2 Hz, 1H), 4.28 (dd, J = 5.0, 2.2 Hz, 1H), 4.22 (d, J = 7.2 Hz, 1H), 4.16 (dd, J = 7.9, 1.6 Hz, 1H), 2.05 (q, J = 7.1 Hz, 2H), 1.53 (s, 3H), 1.51 – 1.43 (m, 5H), 1.33 (d, J = 2.5 Hz, 6H); **^{13}C NMR (101 MHz, $CDCl_3$)** δ 138.6, 134.6, 125.8, 114.5, 109.1, 108.3, 96.5, 73.8, 70.9, 70.4, 69.1, 33.2, 28.0, 26.1, 26.0, 24.9, 24.4; **ATR-FTIR (cm $^{-1}$):** 2987, 2921, 1381, 1210, 1065, 971, 898, 511; **HRMS m/z (ESI)** calculated for $C_{18}H_{27}D_2O_5^+$ $[M+H]^+$ 327.2135, found 327.2122.

The characterization data of the corresponding non-deuterated product are as follows:

(3a*R*,5*R*,5a*S*,8a*S*,8b*R*)-5-((*E*)-hepta-1,6-dien-1-yl)-2,2,7,7-tetramethyltetrahydro-5*H*-bis([1,3]dioxolo)[4,5-*b*:4',5'-d]pyran (5ax')

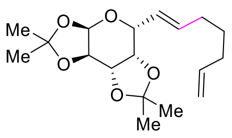

^{1}H NMR (400 MHz, $CDCl_3$) δ 5.85 – 5.69 (m, 2H), 5.60 (dd, J = 15.5, 7.2 Hz, 1H), 5.54 (d, J = 5.1 Hz, 1H), 5.06 – 4.87 (m, 2H), 4.58 (dd, J = 7.8, 2.3

Hz, 1H), 4.28 (dd, *J* = 5.0, 2.3 Hz, 1H), 4.21 (d, *J* = 7.3 Hz, 1H), 4.15 (dd, *J* = 7.9, 1.9 Hz, 1H), 2.13 – 2.00 (m, 4H), 1.53 (s, 3H), 1.52 – 1.43 (m, 5H), 1.33 (d, *J* = 2.7 Hz, 6H); **^{13}C NMR (101 MHz, $CDCl_3$)** δ 138.6, 134.7, 125.8, 114.5, 109.1, 108.3, 96.5, 73.8, 70.9, 70.4, 69.1, 33.2, 31.8, 28.1, 26.1, 26.0, 24.9, 24.4; **ATR-FTIR (cm $^{-1}$):** 2988, 2933, 1382, 1211, 1066, 993, 899, 734, 511; **HRMS m/z (ESI)** calculated for $C_{18}H_{29}O_5^+$ $[M+H]^+$ 325.2010, found 325.2000.

tert-butyl (1*R*,5*S*)-3-(pent-4-en-1-yl-1,1-*d*$_2$)-8-azabicyclo[3.2.1]oct-2-ene-8-carboxylate (5ay)

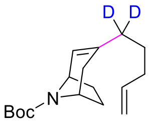

According to General procedure D, the crude product was purified by flash column chromatography on silica gel (PE : EA = 15:1) to afford **5ay** (26.8 mg, 48% yield, 97% D) as a colorless oil: **^{1}H NMR (400 MHz, $CDCl_3$)** δ 5.86 – 5.62 (m, 2H), 5.04 – 4.88 (m, 2H), 4.26 (d, *J* = 29.0 Hz, 2H), 2.68 (dd, *J* = 40.3, 15.5 Hz, 1H), 2.18 – 2.08 (m, 1H), 2.02 – 1.94 (m, 2H), 1.94 – 1.77 (m, 2H), 1.65 (d, *J* = 17.2 Hz, 1H), 1.62 – 1.50 (m, 1H), 1.49 – 1.38 (m, 11H); **^{13}C NMR (101 MHz, $CDCl_3$)** δ 154.2, 138.6, 126.8, 126.3, 114.5, 79.0, 53.4, 52.7, 52.4, 51.7, 37.4, 36.9, 34.9, 34.3, 33.0, 30.1, 29.4, 28.4, 26.3; **ATR-FTIR (cm $^{-1}$):** 2974, 2928, 1694, 1388, 1329, 1169, 1099, 908, 774; **HRMS m/z (ESI)** calculated for $C_{17}H_{26}D_2NO_2^+$ $[M+H]^+$ 280.2240, found 280.2231.

The characterization data of the corresponding non-deuterated product are as follows:

tert-butyl (1*R*,5*S*)-3-(pent-4-en-1-yl)-8-azabicyclo[3.2.1]oct-2-ene-8-carboxylate (5ay')

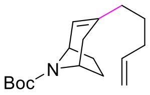

^{1}H NMR (400 MHz, $CDCl_3$) δ 5.89 – 5.61 (m, 2H), 5.08 – 4.88 (m, 2H), 4.26 (d, *J* = 29.1 Hz, 2H), 2.82 – 2.52 (m, 1H), 2.19 – 2.06 (m, 1H), 1.99 (q, *J* = 6.9 Hz, 2H), 1.97 – 1.76 (m, 4H), 1.65 (d, *J* = 17.4 Hz, 1H), 1.62 – 1.51 (m, 1H), 1.49 – 1.38 (m, 11H); **^{13}C NMR (101 MHz, $CDCl_3$)** δ 154.2, 138.6, 126.8, 126.3, 114.5, 79.0, 53.4, 52.7, 52.4, 51.7, 37.4, 36.9, 34.9, 34.3, 33.0, 30.1, 29.4, 28.4, 26.3; **ATR-FTIR (cm $^{-1}$):** 2974, 2930, 1694, 1388, 1328, 1168, 1098, 909, 773; **HRMS m/z (ESI)** calculated for $C_{17}H_{28}NO_2^+$ $[M+H]^+$ 278.2115, found 278.2106.

2-(1-(4-chlorobenzoyl)-5-methoxy-2-methyl-1*H*-indol-3-yl)-1-(4-(pent-4-en-1-yl-1,1-*d*$_2$)-3,6-dihydropyridin-1(2*H*)-yl)ethan-1-one (5az)

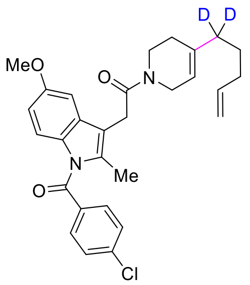

According to General procedure D, the crude product was purified by flash column chromatography on silica gel (PE : EA = 2:1) to afford **5az** (50.6 mg, 51% yield, 97% D) as a yellow oil: **^{1}H NMR (400 MHz, CDCl$_3$)** δ 7.65 (d, *J* = 8.5 Hz, 2H), 7.46 (d, *J* = 8.4 Hz, 2H), 6.99 (dd, *J* = 7.4, 2.3 Hz, 1H), 6.83 (dd, *J* = 9.0, 5.4 Hz, 1H), 6.64 (dd, *J* = 9.0, 2.5 Hz, 1H), 5.86 – 5.70 (m, 1H), 5.36 (d, *J* = 31.8 Hz, 1H), 5.04 – 4.91 (m, 2H), 4.02 (dd, *J* = 28.4, 2.2 Hz, 2H), 3.81 (s, 3H), 3.76 – 3.64 (m, 3H), 3.55 (t, *J* = 5.7 Hz, 1H), 2.37 (d, *J* = 5.9 Hz, 3H), 2.12 – 1.89 (m, 4H), 1.52 – 1.38 (m, 2H); **^{13}C NMR (101 MHz, CDCl$_3$)** δ 168.9, 168.8, 168.2, 156.0, 139.2, 139.2, 138.4, 138.2, 135.8, 135.2, 135.1, 134.1, 134.0, 131.2, 130.9, 130.8, 129.1, 118.2, 116.5, 114.8, 114.7, 113.4, 111.6, 111.5, 101.7, 101.6, 55.7, 45.1, 43.0, 42.2, 39.0, 33.2, 33.1, 30.6, 28.6, 28.0, 26.3, 13.4, 13.4; **ATR-FTIR (cm $^{-1}$):** 2927, 2835, 1677, 1476, 1314, 1222, 1088, 911, 832, 733, 481; **HRMS m/z (ESI)** calculated for $C_{29}H_{30}D_2ClN_2O_3^+$ [M+H]$^+$ 493.2222, found 493.2213.

The characterization data of the corresponding non-deuterated product are as follows:

2-(1-(4-chlorobenzoyl)-5-methoxy-2-methyl-1*H*-indol-3-yl)-1-(4-(pent-4-en-1-yl)-3,6-dihydropyridin-1(2*H*)-yl)ethan-1-one (5az')

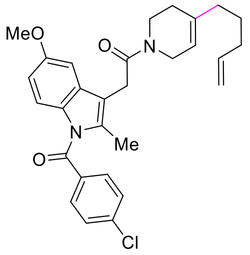

^{1}H NMR (400 MHz, CDCl$_3$) δ 7.65 (d, *J* = 8.4 Hz, 2H), 7.46 (d, *J* = 8.4 Hz, 2H), 6.99 (dd, *J* = 7.3, 2.1 Hz, 1H), 6.83 (dd, *J* = 8.9, 5.3 Hz, 1H), 6.64 (dd, *J* = 9.0, 2.4 Hz, 1H), 5.86 – 5.70 (m, 1H), 5.36 (d, *J* = 32.2 Hz, 1H), 5.04 – 4.91 (m, 2H), 4.02 (d, *J* = 28.5 Hz, 2H), 3.81 (s, 3H), 3.78 – 3.64 (m, 3H), 3.55 (t, *J* = 5.6 Hz, 1H), 2.37 (d, *J* = 5.8 Hz, 3H), 2.12 – 1.89 (m, 6H), 1.52 – 1.38 (m, 2H); **^{13}C NMR (101 MHz, CDCl$_3$)** δ 168.9, 168.8, 168.2, 156.0, 139.2, 139.2, 138.4, 138.2, 135.9, 135.2, 135.1, 134.1, 134.0, 131.2, 130.9, 130.8, 129.1, 118.2, 116.5, 114.8, 114.7, 113.4, 111.6, 111.5, 101.7, 101.6, 55.7, 45.1, 43.0, 42.2, 39.0, 36.4, 36.3, 33.2, 33.2, 30.6, 28.7, 28.1, 26.5, 13.4, 13.4; **ATR-FTIR (cm $^{-1}$):** 2926, 2835, 1677, 1476, 1315, 1221, 1088, 912, 833, 734, 481; **HRMS m/z (ESI)** calculated for $C_{29}H_{32}ClN_2O_3^+$ [M+H]$^+$ 491.2096, found 491.2087.

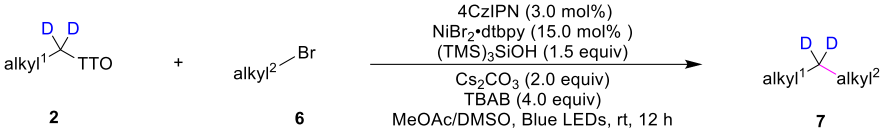

General procedure E: In a nitrogen-filled glove box, to an 8 mL oven-dried vial equipped with a stir bar was added **2** (0.8 mmol, 4 equiv.), Cs_2CO_3 (130.3 mg, 0.4 mmol, 2 equiv.), TBAB (257.9 mg, 0.8 mmol, 4 equiv.), 4CzIPN (4.8 mg, 3 mol%) and $NiBr_2$•dtbpy (14.6 mg, 15 mol%). Then, anhydrous MeOAc (1.6 mL) and anhydrous DMF (0.4 mL) were added via syringe, followed by addition of **6** (0.2 mmol, 1 equiv.) and tris(trimethylsilyl)silanol (95 µL, 0.3 mmol, 1.5 equiv.). The vial was sealed and removed from the glovebox. Subsequently, the reaction mixture was stirred and irradiated with a 40 W blue LED lamp for 16 hours. The final reaction mixture was diluted with EtOAc (60 mL) and saturated aqueous LiCl solution (20 mL). The organic layer was washed with brine (2 x 20 mL) and concentrated. Purification by flash column chromatography on silica gel to afford the alkyl alkylation product.

Note: When using acyclic secondary bromides as substrates (**6d-f**), $NiBr_2$•DME (9.2 mg, 15.0 mol%) and 1,3-Bis(4,5-dihydro-2-oxazolyl)benzene (6.5 mg, 15.0 mol%) were used instead of $NiBr_2$•dtbpy (15.0 mol%) to afford the product (**7ad-af**).

(4-(pent-4-en-1-yl-1,1-*d*$_2$)piperidin-1-yl)(phenyl)methanone (7aa)

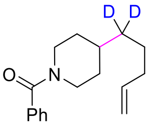

According to General procedure E, the crude product was purified by flash column chromatography on silica gel (PE : EA = 2:1) to afford **7aa** (29.6 mg, 57% yield, 96% D) as a colorless oil: **^{1}H NMR (400 MHz, CDCl$_3$)** δ 7.37 (s, 5H), 5.79 (ddt, *J* = 16.9, 10.2, 6.7 Hz, 1H), 5.08 – 4.83 (m, 2H), 4.69 (d, *J* = 10.0 Hz, 1H), 3.71 (d, *J* = 9.8 Hz, 1H), 3.04 – 2.88 (m, 1H), 2.80 – 2.64 (m, 1H), 2.03 (q, *J* = 7.0 Hz, 2H), 1.85 – 1.73 (m, 1H), 1.67 – 1.56 (m, 1H), 1.49 (tt, *J* = 8.2, 3.4 Hz, 1H), 1.38 (t, *J* = 7.3 Hz, 2H), 1.29 – 1.14 (m, 1H), 1.15 – 0.99 (m, 1H); **^{13}C NMR (101 MHz, CDCl$_3$)** δ 170.2, 138.7, 136.4, 129.3, 128.3, 126.7, 114.4, 48.0, 42.5, 35.8, 33.7, 32.8, 31.9, 25.6; **ATR-FTIR (cm^{-1}):** 2922, 2854, 1628, 1429, 1274, 908, 707; **HRMS m/z (ESI)** calculated for $C_{17}H_{22}D_2NO^+$ $[M+H]^+$ 260.1978, found 260.1983.

(4-(pent-4-en-1-yl)piperidin-1-yl)(phenyl)methanone (7aa')

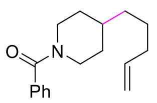

^{1}H NMR (400 MHz, CDCl$_3$) δ 7.37 (s, 5H), 5.78 (ddt, J = 16.9, 10.2, 6.7 Hz, 1H), 5.09 – 4.85 (m, 2H), 4.69 (d, J = 10.3 Hz, 1H), 3.71 (d, J = 11.1 Hz, 1H), 2.95 (t, J = 11.2 Hz, 1H), 2.73 (t, J = 11.0 Hz, 1H), 2.03 (q, J = 7.0 Hz, 2H), 1.80 (d, J = 10.5 Hz, 1H), 1.68 – 1.57 (m, 1H), 1.49 (ddt, J = 11.0, 7.9, 3.8 Hz, 1H), 1.45 – 1.34 (m, 2H), 1.31 – 1.15 (m, 3H), 1.14 – 1.03 (m, 1H); **^{13}C NMR (101 MHz, CDCl$_3$)** δ 170.1, 138.6, 136.4, 129.3, 128.3, 126.7, 114.4, 48.0, 42.4, 36.0, 35.7, 33.8, 32.8, 31.9, 25.8; **ATR-FTIR (cm $^{-1}$):** 2925, 2851, 1628, 1429, 1281, 908, 707; **HRMS m/z (ESI)** calculated for $C_{17}H_{24}NO^+$ [M+H]$^+$ 258.1858, found 258.1855.

4-(pent-4-en-1-yl-1,1-*d*$_2$)-1-tosylpiperidine (7ab)

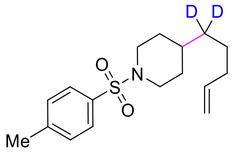

According to General procedure E, the crude product was purified by flash column chromatography on silica gel (PE : EA = 10:1) to afford **7ab** (35.8 mg, 58% yield, 96% D) as a white solid: **^{1}H NMR (400 MHz, CDCl$_3$)** δ 7.63 (d, J = 8.2 Hz, 2H), 7.31 (d, J = 8.0 Hz, 2H), 5.75 (ddt, J = 16.9, 10.2, 6.7 Hz, 1H), 5.05 – 4.85 (m, 2H), 3.74 (d, J = 11.6 Hz, 2H), 2.42 (s, 3H), 2.19 (td, J = 11.8, 2.3 Hz, 2H), 2.06 – 1.89 (m, 2H), 1.77 – 1.64 (m, 2H), 1.38 – 1.19 (m, 4H), 1.17 – 1.05 (m, 1H); **^{13}C NMR (101 MHz, CDCl$_3$)** δ 143.2, 138.6, 133.2, 129.5, 127.7, 114.4, 46.5, 34.8, 33.7, 31.4, 25.6, 21.5; **ATR-FTIR (cm $^{-1}$):** 2922, 2846, 1338, 1162, 928, 723, 546; **HRMS m/z (ESI)** calculated for $C_{17}H_{24}D_2NO_2S^+$ [M+H]$^+$ 310.1804, found 310.1799.

The characterization data of the corresponding non-deuterated product are as follows:

4-(pent-4-en-1-yl)-1-tosylpiperidine (7ab')

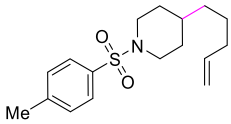

^{1}H NMR (400 MHz, CDCl$_3$) δ 7.63 (d, J = 8.2 Hz, 2H), 7.31 (d, J = 8.1 Hz, 2H), 5.75 (ddt, J = 16.9, 10.2, 6.7 Hz, 1H), 5.01 – 4.86 (m, 2H), 3.74 (d, J = 11.6 Hz, 2H), 2.42 (s, 3H), 2.19 (td, J = 11.8, 2.1 Hz, 2H), 1.98 (q, J = 7.1 Hz, 2H), 1.69 (d, J = 12.6 Hz, 2H), 1.41 – 1.07 (m, 7H); **^{13}C NMR (101 MHz, CDCl$_3$)** δ 143.2, 138.6, 133.2, 129.5, 127.7, 114.4, 46.5, 35.4, 35.0, 33.7, 31.5, 25.8, 21.5; **ATR-FTIR (cm $^{-1}$):** 2921, 2848, 1337, 1162, 926, 723, 547; **HRMS m/z (ESI)** calculated for $C_{17}H_{26}NO_2S^+$ [M+H]$^+$ 308.1679, found 308.1672. Spectroscopic data are in agreement with the literature.[8]

benzyl 3-(pent-4-en-1-yl-1,1-d_2)azetidine-1-carboxylate (7ac)

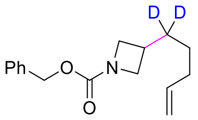

According to General procedure E, the crude product was purified by flash column chromatography on silica gel (PE : EA = 6:1) to afford **7ac** (25.2 mg, 48% yield, 97% D) as a colorless oil: **^{1}H NMR (400 MHz, $CDCl_3$)** δ 7.39 – 7.27 (m, 5H), 5.78 (ddt, *J* = 16.9, 10.2, 6.7 Hz, 1H), 5.09 (s, 2H), 5.05 – 4.91 (m, 2H), 4.07 (t, *J* = 8.4 Hz, 2H), 3.61 (dd, *J* = 8.6, 5.6 Hz, 2H), 2.60 – 2.45 (m, 1H), 2.04 (q, *J* = 7.0 Hz, 2H), 1.32 (t, *J* = 7.4 Hz, 2H); **^{13}C NMR (101 MHz, $CDCl_3$)** δ 156.4, 138.3, 136.9, 128.4, 127.9, 127.9, 114.8, 66.4, 54.6, 33.3, 29.1, 26.0; **ATR-FTIR (cm $^{-1}$):** 2925, 2878, 1704, 1411, 1356, 1121, 910, 766, 696; **HRMS m/z (ESI)** calculated for $C_{16}H_{19}D_2NO_2Na^+$ $[M+H]^+$ 284.1590, found 284.1581.

The characterization data of the corresponding non-deuterated product are as follows:

benzyl 3-(pent-4-en-1-yl)azetidine-1-carboxylate (7ac')

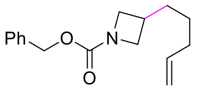

^{1}H NMR (400 MHz, $CDCl_3$) δ 7.39 – 7.27 (m, 5H), 5.78 (ddt, *J* = 16.9, 10.2, 6.7 Hz, 1H), 5.09 (s, 2H), 5.05 – 4.91 (m, 2H), 4.08 (t, *J* = 8.4 Hz, 2H), 3.61 (dd, *J* = 8.5, 5.6 Hz, 2H), 2.64 – 2.41 (m, 1H), 2.05 (q, *J* = 7.1 Hz, 2H), 1.59 (q, *J* = 7.7 Hz, 2H), 1.42 – 1.26 (m, 2H); **^{13}C NMR (101 MHz, $CDCl_3$)** δ 156.4, 138.3, 136.9, 128.4, 127.9, 127.9, 114.8, 66.4, 54.7, 33.8, 33.4, 29.3, 26.2; **ATR-FTIR (cm $^{-1}$):** 2926, 2878, 1704, 1412, 1347, 1124, 910, 766, 696; **HRMS m/z (ESI)** calculated for $C_{16}H_{21}NO_2Na^+$ $[M+H]^+$ 282.1465, found 282.1469.

3-methyloct-7-en-1-yl-4,4-d_2 nicotinate (7ad)

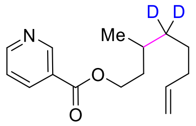

According to General procedure E, the reaction was carried out with $NiBr_2$•DME (9.2 mg, 15.0 mol%) and 1,3-Bis(4,5-dihydro-2-oxazolyl)benzene (6.5 mg, 15.0 mol%). The crude product which was purified by flash column chromatography on silica gel (PE : EA = 3:1) to afford **7ad** (22.5 mg, 45% yield, 97% D) as a colorless oil: **^{1}H NMR (400 MHz, $CDCl_3$)** δ 9.21 (d, *J* = 1.4, 1H), 8.76 (dd, *J* = 4.8, 1.4 Hz, 1H), 8.27 (dt, *J* = 7.9, 1.9 Hz, 1H), 7.37 (dd, *J* = 7.9, 4.9 Hz, 1H), 5.79 (ddt, *J* = 16.9, 10.2, 6.7 Hz, 1H), 5.06 – 4.84 (m, 2H), 4.44 – 4.31 (m, 2H), 2.07 – 1.98 (m, 2H), 1.87 – 1.72 (m, 1H), 1.71 – 1.51 (m, 2H), 1.48 – 1.30 (m, 2H), 0.95 (d, *J* = 6.4 Hz, 3H); **^{13}C NMR (101 MHz, $CDCl_3$)** δ 165.3, 153.3, 150.9, 138.8, 136.9, 126.3, 123.2, 114.4, 64.0, 35.4, 33.8, 29.7, 26.0, 19.4; **ATR-FTIR (cm $^{-1}$):** 2925, 2870, 1721, 1591, 1279,

1111, 1024, 909, 740, 702; **HRMS m/z (ESI)** calculated for $C_{15}H_{20}D_2NO_2^+$ $[M+H]^+$ 250.1771, found 250.1770.

The characterization data of the corresponding non-deuterated product are as follows:

3-methyloct-7-en-1-yl nicotinate (7ad')

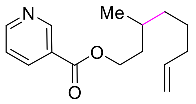

^{1}H NMR (400 MHz, CDCl$_3$) δ 9.21 (d, J = 1.6 Hz, 1H), 8.75 (dd, J = 4.8, 1.6 Hz, 1H), 8.27 (dt, J = 7.9, 1.9 Hz, 1H), 7.37 (dd, J = 7.9, 4.9 Hz, 1H), 5.79 (ddt, J = 16.9, 10.2, 6.7 Hz, 1H), 5.04 – 4.86 (m, 2H), 4.44 – 4.31 (m, 2H), 2.09 – 1.97 (m, 2H), 1.87 – 1.75 (m, 1H), 1.71 – 1.52 (m, 2H), 1.49 – 1.31 (m, 3H), 1.28 – 1.15 (m, 1H), 0.95 (d, J = 6.4 Hz, 3H); **^{13}C NMR (101 MHz, CDCl$_3$)** δ 165.3, 153.3, 150.9, 138.8, 136.9, 126.3, 123.2, 114.4, 64.0, 36.3, 35.5, 33.9, 29.9, 26.2, 19.5; **ATR-FTIR (cm^{-1}):** 2928, 2859, 1721, 1591, 1277, 1111, 1023, 909, 740, 702; **HRMS m/z (ESI)** calculated for $C_{15}H_{22}NO_2^+$ $[M+H]^+$ 248.1645, found 248.1645.

2-(3-methyloct-7-en-1-yl-4,4-d_2)isoindoline-1,3-dione (7ae)

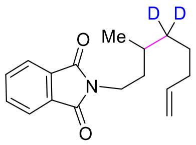

According to General procedure E, the reaction was carried out with NiBr$_2$•DME (9.2 mg, 15.0 mol%) and 1,3-Bis(4,5-dihydro-2-oxazolyl)benzene (6.5 mg, 15.0 mol%). The crude product was purified by flash column chromatography on silica gel (PE : EA = 10:1) to afford **7ae** (26.6 mg, 49% yield, 97% D) as a colorless oil: **^{1}H NMR (400 MHz, CDCl$_3$)** δ 7.82 (dd, J = 5.4, 3.0 Hz, 2H), 7.69 (dd, J = 5.4, 3.1 Hz, 2H), 5.78 (ddt, J = 16.9, 10.2, 6.7 Hz, 1H), 5.08 – 4.83 (m, 2H), 3.74 – 3.63 (m, 2H), 2.09 – 1.93 (m, 2H), 1.77 – 1.60 (m, 1H), 1.54 – 1.43 (m, 2H), 1.42 – 1.27 (m, 2H), 0.96 (d, J = 6.3 Hz, 3H); **^{13}C NMR (101 MHz, CDCl$_3$)** δ 168.3, 139.0, 133.8, 132.2, 123.1, 114.3, 36.3, 35.4, 33.9, 30.4, 25.9, 19.3; **ATR-FTIR (cm^{-1}):** 2926, 2868, 1706, 1395, 1368, 1052, 716, 529; **HRMS m/z (ESI)** calculated for $C_{17}H_{20}D_2NO_2^+$ $[M+H]^+$ 274.1771, found 274.1759.

The characterization data of the corresponding non-deuterated product are as follows:

2-(3-methyloct-7-en-1-yl)isoindoline-1,3-dione (7ae')

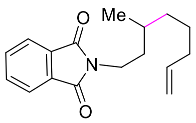

^{1}H NMR (400 MHz, CDCl$_3$) δ 7.82 (dd, J = 5.4, 3.1 Hz, 2H), 7.69 (dd, J = 5.4, 3.0 Hz, 2H), 5.78 (ddt, J = 16.9, 10.2, 6.7 Hz, 1H), 5.02 – 4.86 (m, 2H), 3.75 –

3.62 (m, 2H), 2.06 – 1.95 (m, 2H), 1.76 – 1.61 (m, 1H), 1.53 – 1.29 (m, 5H), 1.23 – 1.11 (m, 1H), 0.96 (d, J = 6.2 Hz, 3H); **^{13}C NMR (101 MHz, $CDCl_3$)** δ 168.3, 138.9, 133.8, 132.2, 123.1, 114.3, 36.3, 36.2, 35.5, 33.9, 30.6, 26.1, 19.3; **ATR-FTIR (cm $^{-1}$):** 2929, 2859, 1706, 1395, 1367, 1061, 717, 529; **HRMS m/z (ESI)** calculated for $C_{17}H_{22}NO_2^+$ $[M+H]^+$ 272.1645, found 272.1635.

4-methyl-7-((3-methyloct-7-en-1-yl-4,4-*d*$_2$)oxy)-2*H*-chromen-2-one (7af)

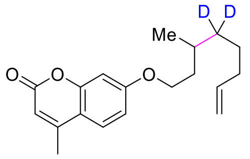

According to General procedure E, the reaction was carried out with $NiBr_2$•DME (9.2 mg, 15.0 mol%) and 1,3-Bis(4,5-dihydro-2-oxazolyl)benzene (6.5 mg, 15.0 mol%). The crude product was purified by flash column chromatography on silica gel (PE : EA = 4:1) to afford **7af** (25.1 mg, 41% yield, 98% D) as a pale yellow oil: **^{1}H NMR (400 MHz, $CDCl_3$)** δ 7.48 (d, J = 8.8 Hz, 1H), 6.84 (dd, J = 8.8, 2.5 Hz, 1H), 6.80 (d, J = 2.4 Hz, 1H), 6.21 – 6.02 (m, 1H), 5.81 (ddt, J = 16.9, 10.2, 6.7 Hz, 1H), 5.07 – 4.88 (m, 2H), 4.10 – 3.98 (m, 2H), 2.39 (d, J = 1.0 Hz, 3H), 2.10 – 1.99 (m, 2H), 1.91 – 1.76 (m, 1H), 1.72 – 1.54 (m, 2H), 1.51 – 1.32 (m, 2H), 0.95 (d, J = 6.5 Hz, 3H); **^{13}C NMR (101 MHz, $CDCl_3$)** δ 162.2, 161.3, 155.3, 152.5, 138.9, 125.4, 114.4, 113.4, 112.7, 111.8, 101.3, 66.9, 35.8, 33.9, 29.5, 26.0, 19.5, 18.6; **ATR-FTIR (cm $^{-1}$):** 2924, 2871, 1717, 1610, 1386, 1263, 1137, 1069, 846; **HRMS m/z (ESI)** calculated for $C_{19}H_{23}D_2O_3^+$ $[M+H]^+$ 303.1924, found 303.1923.

The characterization data of the corresponding non-deuterated product are as follows:

4-methyl-7-((3-methyloct-7-en-1-yl)oxy)-2*H*-chromen-2-one (7af')

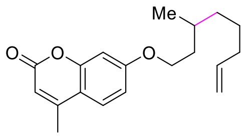

^{1}H NMR (400 MHz, $CDCl_3$) δ 7.47 (d, J = 8.8 Hz, 1H), 6.83 (dd, J = 8.8, 2.4 Hz, 1H), 6.78 (d, J = 2.4 Hz, 1H), 6.10 (d, J = 0.8 Hz, 1H), 5.80 (ddt, J = 16.9, 10.2, 6.7 Hz, 1H), 5.04 – 4.89 (m, 2H), 4.09 – 3.97 (m, 2H), 2.38 (d, J = 0.8 Hz, 3H), 2.09 – 1.97 (m, 2H), 1.90 – 1.78 (m, 1H), 1.72 – 1.53 (m, 2H), 1.51 – 1.30 (m, 3H), 1.27 – 1.13 (m, 1H), 0.94 (d, J = 6.5 Hz, 3H); **^{13}C NMR (101 MHz, $CDCl_3$)** δ 162.1, 161.2, 155.2, 152.5, 138.8, 125.4, 114.4, 113.3, 112.6, 111.8, 101.3, 66.8, 36.3, 35.8, 33.9, 29.6, 26.1, 19.5, 18.6; **ATR-FTIR (cm $^{-1}$):** 2926, 2869, 1717, 1611, 1386, 1263, 1136, 1069, 846; **HRMS m/z (ESI)** calculated for $C_{19}H_{25}O_3^+$ $[M+H]^+$ 301.1798, found 301.1802.

4-acetamidophenyl non-8-enoate-5,5-*d*$_2$ (7ag)

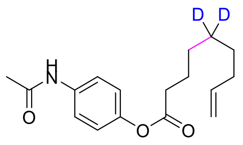

According to General procedure E, the crude product was purified by flash column chromatography on silica gel (PE : EA = 1:2) to afford **7ag** (27.6 mg, 47% yield, 98% D) as a white solid: **^{1}H NMR (400 MHz, CDCl$_3$)** δ 7.73 (s, 1H), 7.44 (d, *J* = 8.5 Hz, 2H), 6.97 (d, *J* = 8.6 Hz, 2H), 5.81 (ddt, *J* = 16.9, 10.2, 6.7 Hz, 1H), 4.97 (dd, *J* = 23.1, 13.6 Hz, 2H), 2.54 (t, *J* = 7.5 Hz, 2H), 2.10 (s, 3H), 2.05 (dd, *J* = 14.5, 7.1 Hz, 2H), 1.78 – 1.68 (m, 2H), 1.40 (t, *J* = 7.4 Hz, 4H); **^{13}C NMR (101 MHz, CDCl$_3$)** δ 172.6, 168.5, 146.8, 138.9, 135.6, 121.8, 120.9, 114.3, 34.3, 33.6, 28.7, 28.4, 24.8, 24.3; **ATR-FTIR (cm $^{-1}$):** 3296, 2924, 2854, 1750, 1655, 1509, 1202, 1143, 909, 847, 706, 517; **HRMS m/z (ESI)** calculated for $C_{17}H_{22}D_2NO_3^+$ [M+H]$^+$ 292.1876, found 292.1867.

The characterization data of the corresponding non-deuterated product are as follows:

4-acetamidophenyl non-8-enoate (7ag')

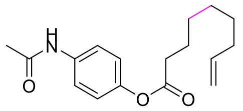

^{1}H NMR (400 MHz, CDCl$_3$) δ 7.83 (s, 1H), 7.44 (d, *J* = 8.6 Hz, 2H), 6.97 (d, *J* = 8.6 Hz, 2H), 5.80 (ddt, *J* = 16.9, 10.1, 6.7 Hz, 1H), 4.97 (dd, *J* = 23.1, 13.6 Hz, 2H), 2.54 (t, *J* = 7.5 Hz, 2H), 2.13 – 1.97 (m, 5H), 1.80 – 1.68 (m, 2H), 1.48 – 1.28 (m, 6H); **^{13}C NMR (101 MHz, CDCl$_3$)** δ 172.6, 168.5, 146.8, 138.8, 135.7, 121.8, 120.9, 114.3, 34.3, 33.6, 28.9, 28.6, 24.8, 24.2; **ATR-FTIR (cm $^{-1}$):** 3296, 2927, 2853, 1750, 1655, 1508, 1198, 1137, 910, 848, 705, 517; **HRMS m/z (ESI)** calculated for $C_{17}H_{24}NO_3^+$ [M+H]$^+$ 290.1751, found 290.1744.

4-methyl-7-((oct-7-en-1-yl-4,4-*d*$_2$)oxy)-2*H*-chromen-2-one (7ah)

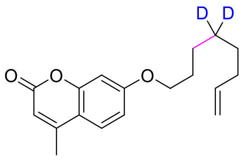

According to General procedure E, the crude product was purified by flash column chromatography on silica gel (PE : EA = 4:1) to afford **7ah** (26.5 mg, 46% yield, 98% D) as a pale yellow oil: **^{1}H NMR (400 MHz, CDCl$_3$)** δ 7.46 (d, *J* = 8.8 Hz, 1H), 6.83 (dd, *J* = 8.8, 2.4 Hz, 1H), 6.77 (d, *J* = 2.4 Hz, 1H), 6.10 (d, *J* = 0.8 Hz, 1H), 5.80 (ddt, *J* = 16.9, 10.2, 6.7 Hz, 1H), 5.15 – 4.80 (m, 2H), 3.99 (t, *J* = 6.5 Hz, 2H), 2.37 (d, *J* = 0.8 Hz, 3H), 2.05 (q, *J* = 6.9 Hz, 2H), 1.84 – 1.72 (m, 2H), 1.49 – 1.41 (m, 2H), 1.40 (t, *J* = 7.7 Hz, 2H); **^{13}C NMR (101 MHz, CDCl$_3$)** δ 162.2, 161.2, 155.2, 152.5, 138.8, 125.4, 114.3, 113.3, 112.6, 111.7, 101.3, 68.5, 33.5, 28.8, 28.5, 25.6, 18.6; **ATR-FTIR (cm $^{-1}$):** 2922, 2856, 1716, 1610, 1386, 1262, 1137, 1068, 846; **HRMS m/z (ESI)** calculated for $C_{18}H_{21}D_2O_3^+$ [M+H]$^+$ 289.1767,

found 289.1766.

The characterization data of the corresponding non-deuterated product are as follows:

4-methyl-7-((oct-7-en-1-yl)oxy)-2*H*-chromen-2-one (7ah')

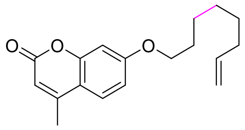

^{1}H NMR (400 MHz, $CDCl_3$) δ 7.47 (d, *J* = 8.8 Hz, 1H), 6.83 (dd, *J* = 8.8, 2.5 Hz, 1H), 6.78 (d, *J* = 2.4 Hz, 1H), 6.11 (d, *J* = 1.0 Hz, 1H), 5.80 (ddt, *J* = 16.9, 10.2, 6.7 Hz, 1H), 5.05 – 4.87 (m, 2H), 3.99 (t, *J* = 6.5 Hz, 2H), 2.38 (d, *J* = 1.0 Hz, 3H), 2.06 (q, *J* = 6.8 Hz, 2H), 1.85 – 1.74 (m, 2H), 1.53 – 1.29 (m, 6H); **^{13}C NMR (101 MHz, $CDCl_3$)** δ 162.2, 161.3, 155.2, 152.5, 138.8, 125.4, 114.3, 113.4, 112.6, 111.8, 101.3, 68.5, 33.6, 28.9, 28.7, 25.8, 18.6; **ATR-FTIR (cm $^{-1}$):** 2946, 2856, 1716, 1610, 1386, 1263, 1136, 1069, 846; **HRMS m/z (ESI)** calculated for $C_{18}H_{23}O_3^+$ $[M+H]^+$ 287.1642, found 287.1645.

phenyl(4-(3-phenylpropyl-1,1-*d*$_2$)piperidin-1-yl)methanone (7ba)

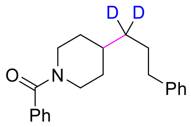

According to General procedure E, the crude product was purified by flash column chromatography on silica gel (PE : EA = 2:1) to afford **7ba** (35.0 mg, 57% yield, 96% D) as a colorless oil: **^{1}H NMR (400 MHz, $CDCl_3$)** δ 7.45 – 7.37 (m, 5H), 7.34 – 7.26 (m, 2H), 7.25 – 7.16 (m, 3H), 4.72 (d, *J* = 9.1 Hz, 1H), 3.74 (d, *J* = 10.1 Hz, 1H), 2.97 (d, *J* = 10.2 Hz, 1H), 2.85 – 2.68 (m, 1H), 2.66 – 2.59 (m, 2H), 1.92 – 1.76 (m, 1H), 1.71 – 1.61 (m, 3H), 1.60 – 1.48 (m, 1H), 1.35 – 1.01 (m, 2H); **^{13}C NMR (101 MHz, $CDCl_3$)** δ 170.2, 142.4, 136.5, 129.3, 128.3, 128.3, 128.3, 126.8, 125.7, 48.0, 42.5, 36.0, 35.9, 32.8, 31.9, 28.2; **ATR-FTIR (cm $^{-1}$):** 2924, 2854, 1627, 1429, 1274, 960, 730, 697; **HRMS m/z (ESI)** calculated for $C_{21}H_{24}D_2NO^+$ $[M+H]^+$ 310.2134, found 310.2134.

The characterization data of the corresponding non-deuterated product are as follows:

phenyl(4-(3-phenylpropyl)piperidin-1-yl)methanone (7ba')

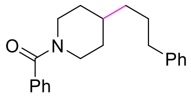

^{1}H NMR (400 MHz, $CDCl_3$) δ 7.41 – 7.34 (m, 5H), 7.31 – 7.23 (m, 2H), 7.22 – 7.12 (m, 3H), 4.68 (s, 1H), 3.71 (d, *J* = 7.3 Hz, 1H), 2.94 (s, 1H), 2.80 – 2.63 (m, 1H), 2.60 (t, *J* = 7.7 Hz, 2H), 1.79 (s, 1H), 1.70 – 1.57 (m, 3H), 1.56 – 1.45 (m, 1H), 1.37 – 1.26 (m, 2H), 1.25 – 1.01 (m, 2H); **^{13}C NMR (101 MHz, $CDCl_3$)** δ 170.2, 142.4, 136.5, 129.4, 128.4, 128.4,

128.3, 126.8, 125.8, 48.1, 42.5, 36.1, 36.1, 36.0, 32.9, 32.1, 28.5; **ATR-FTIR (cm $^{-1}$):** 2927, 2852, 1627, 1429, 1277, 968, 731, 697; **HRMS m/z (ESI)** calculated for $C_{21}H_{26}NO^+$ $[M+H]^+$ 308.2009, found 308.2008.

(4-(3-(benzyloxy)propyl-1,1-*d*$_2$)piperidin-1-yl)(phenyl)methanone (7ha)

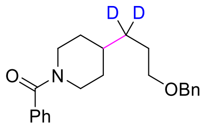

According to General procedure E, the crude product was purified by flash column chromatography on silica gel (PE : EA = 2:1) to afford **7ha** (30.6 mg, 45% yield, 97% D) as a colorless oil: **^{1}H NMR (400 MHz, CDCl$_3$)** δ 7.41 – 7.36 (m, 5H), 7.36 – 7.30 (m, 4H), 7.30 – 7.25 (m, 1H), 4.71 (s, 1H), 4.50 (s, 2H), 3.72 (s, 1H), 3.46 (t, *J* = 6.5 Hz, 2H), 2.95 (s, 1H), 2.74 (s, 1H), 1.79 (s, 1H), 1.62 (t, *J* = 6.5 Hz, 3H), 1.55 – 1.44 (m, 1H), 1.22 (s, 1H), 1.11 (s, 1H); **^{13}C NMR (101 MHz, CDCl$_3$)** δ 170.2, 138.5, 136.5, 129.3, 128.3, 128.3, 127.6, 127.5, 126.8, 72.9, 70.3, 48.0, 42.4, 35.8, 32.8, 31.9, 26.7; **ATR-FTIR (cm $^{-1}$):** 2927, 2850, 1627, 1430, 1274, 1096, 733, 696; **HRMS m/z (ESI)** calculated for $C_{22}H_{26}D_2NO_2^+$ $[M+H]^+$ 340.2240, found 340.2236.

The characterization data of the corresponding non-deuterated product are as follows:

(4-(3-(benzyloxy)propyl)piperidin-1-yl)(phenyl)methanone (7ha')

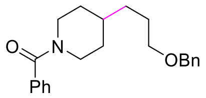

^{1}H NMR (400 MHz, CDCl$_3$) δ 7.41 – 7.36 (m, 5H), 7.36 – 7.30 (m, 4H), 7.30 – 7.25 (m, 1H), 4.69 (s, 1H), 4.50 (s, 2H), 3.72 (s, 1H), 3.47 (t, *J* = 6.5 Hz, 2H), 2.94 (s, 1H), 2.74 (s, 1H), 1.79 (s, 1H), 1.69 – 1.58 (m, 3H), 1.57 – 1.45 (m, 1H), 1.36 (dd, *J* = 15.8, 6.8 Hz, 2H), 1.21 (s, 1H), 1.12 (s, 1H); **^{13}C NMR (101 MHz, CDCl$_3$)** δ 170.2, 138.5, 136.5, 129.3, 128.3, 128.3, 127.6, 127.5, 126.8, 72.9, 70.4, 48.0, 42.4, 36.0, 32.8, 32.8, 31.9, 26.8; **ATR-FTIR (cm $^{-1}$):** 2931, 2852, 1626, 1431, 1275, 1099, 731, 696; **HRMS m/z (ESI)** calculated for $C_{22}H_{28}NO_2^+$ $[M+H]^+$ 338.2115, found 338.2115.

phenyl(4-(4,4,4-trifluorobutyl-1,1-*d*$_2$)piperidin-1-yl)methanone (7ia)

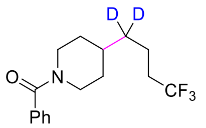

According to General procedure E, the crude product was purified by flash column chromatography on silica gel (PE : EA = 2:1) to afford **6ia** (35.1 mg, 58% yield, 97% D) as a colorless oil: **^{1}H NMR (400 MHz, CDCl$_3$)** δ 7.41 –

7.34 (m, 5H), 4.69 (s, 1H), 3.72 (s, 1H), 2.95 (s, 1H), 2.73 (s, 1H), 2.13 – 1.97 (m, 2H), 1.87 – 1.59 (m, 2H), 1.59 – 1.52 (m, 2H), 1.51 – 1.45 (m, 1H), 1.33 – 0.99 (m, 2H); **^{13}C NMR (101 MHz, $CDCl_3$)** δ 170.2, 136.3, 129.4, 128.3, 127.0 (q, J = 276.4 Hz), 126.7, 47.9, 42.3, 35.6, 33.7 (q, J = 28.5 Hz), 32.5, 31.7, 18.8 (q, J = 2.7 Hz); **^{19}F NMR (376 MHz, $CDCl_3$)** δ -66.4; **ATR-FTIR (cm $^{-1}$):** 2933, 2855, 1627, 1431, 1252, 1135, 1005, 707; **HRMS m/z (ESI)** calculated for $C_{16}H_{19}D_2F_3NO^+$ $[M+H]^+$ 302.1695, found 302.1699.

The characterization data of the corresponding non-deuterated product are as follows:

phenyl(4-(4,4,4-trifluorobutyl)piperidin-1-yl)methanone (7ia')

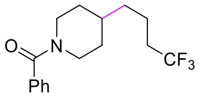

^{1}H NMR (400 MHz, $CDCl_3$) δ 7.41 – 7.34 (m, 5H), 4.70 (s, 1H), 3.73 (s, 1H), 2.95 (s, 1H), 2.74 (s, 1H), 2.13 – 1.97 (m, 2H), 1.78 (s, 1H), 1.71 – 1.44 (m, 4H), 1.33 (dd, J = 15.7, 6.8 Hz, 2H), 1.26 – 1.01 (m, 2H); **^{13}C NMR (101 MHz, $CDCl_3$)** δ 170.2, 136.3, 129.4, 128.3, 127.0 (q, J = 276.5 Hz), 126.8, 47.8, 42.2, 35.9, 35.3, 33.8 (q, J = 28.4 Hz), 32.6, 31.8, 19.1 (q, J = 2.9 Hz); **^{19}F NMR (376 MHz, $CDCl_3$)** δ -66.4; **ATR-FTIR (cm $^{-1}$):** 2936, 2855, 1625, 1435, 1252, 1132, 732, 706; **HRMS m/z (ESI)** calculated for $C_{16}H_{21}F_3NO^+$ $[M+H]^+$ 300.1570, found 300.1569.

(4-(4-chlorobutyl-1,1-d_2)piperidin-1-yl)(phenyl)methanone (7ja)

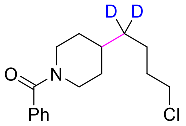

According to General procedure E, the crude product was purified by flash column chromatography on silica gel (PE : EA = 2:1) to afford **7ja** (34.8 mg, 62% yield, 97% D) as a colorless oil: **^{1}H NMR (400 MHz, $CDCl_3$)** δ 7.41 – 7.34 (m, 5H), 4.68 (s, 1H), 3.71 (s, 1H), 3.52 (t, J = 6.6 Hz, 2H), 2.94 (s, 1H), 2.74 (s, 1H), 1.81 – 1.68 (m, 3H), 1.66 (s, 1H), 1.55 – 1.37 (m, 3H), 1.30 – 0.98 (m, 2H); **^{13}C NMR (101 MHz, $CDCl_3$)** δ 170.2, 136.4, 129.3, 128.3, 126.8, 48.0, 44.9, 42.4, 35.8, 32.7, 32.6, 31.9, 23.7; **ATR-FTIR (cm $^{-1}$):** 2931, 2857, 1625, 1431, 1274, 731, 706; **HRMS m/z (ESI)** calculated for $C_{16}H_{21}D_2ClNO^+$ $[M+H]^+$ 282.1588, found 282.1584.

The characterization data of the corresponding non-deuterated product are as follows:

(4-(4-chlorobutyl)piperidin-1-yl)(phenyl)methanone (7ja')

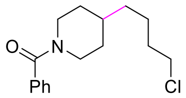

^{1}H NMR (400 MHz, $CDCl_3$) δ 7.41 – 7.34 (m, 5H), 4.68 (s, 1H), 3.70 (s, 1H), 3.52 (t, J = 6.6 Hz, 2H), 2.94 (s, 1H), 2.74 (s, 1H), 1.83 – 1.69 (m, 3H), 1.66 (s, 1H), 1.58 – 1.41 (m, 3H), 1.34 – 1.25 (m, 2H), 1.24 – 1.01 (m, 2H); **^{13}C NMR (101 MHz, $CDCl_3$)** δ 170.2, 136.4, 129.3, 128.3, 126.7, 48.0, 44.8, 42.4, 36.0, 35.5, 32.8, 32.6, 32.0, 23.8; **ATR-FTIR (cm $^{-1}$):** 2931, 2853, 1625, 1430, 1277, 731, 706; **HRMS m/z (ESI)** calculated for $C_{16}H_{23}ClNO^+$ $[M+H]^+$ 280.1463, found 280.1461.

5. Cross-electrophile coupling reactions between two alkyl thianthrenium salts

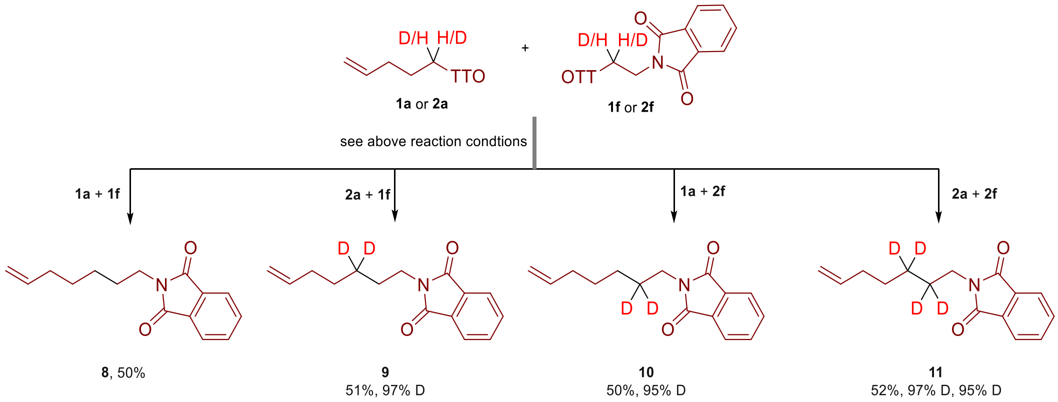

General procedure F: In a nitrogen-filled glove box, to an 8 mL oven-dried vial equipped with a stir bar was added **1a** or **2a** (0.8 mmol, 4 equiv.), **1f** or **2f** (0.2 mmol, 1 equiv.), Cs_2CO_3 (130.3 mg, 0.4 mmol, 2 equiv.), TBAB (322.4 mg, 1.0 mmol, 5 equiv.), 4CzIPN (4.8 mg, 3 mol%) and $NiBr_2$•dtbpy (14.6 mg, 15 mol%). Then, anhydrous MeOAc (1.6 mL) and anhydrous DMF (0.4 mL) were added via syringe, followed by addition of tris(trimethylsilyl)silanol (95 μL, 0.3 mmol, 1.5 equiv.). The vial was sealed and removed from the glovebox. Subsequently, the reaction mixture was stirred and irradiated with a 40 W blue LED lamp for 16 hours. The final reaction mixture was diluted with EtOAc (60 mL) and saturated aqueous LiCl solution (20 mL). The organic layer was washed with brine (2 x 20 mL) and concentrated. Purification by flash column chromatography on silica gel to afford the prduct.

2-(hept-6-en-1-yl)isoindoline-1,3-dione (8)

According to General procedure F, the crude product was purified by flash column chromatography

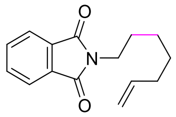

on silica gel (PE : EA = 10:1) to afford **8** (24.0 mg, 50% yield) as a colorless oil: **^{1}H NMR (400 MHz, CDCl$_3$)** δ 7.82 (dd, *J* = 5.4, 3.1 Hz, 2H), 7.69 (dd, *J* = 5.4, 3.1 Hz, 2H), 5.77 (ddt, *J* = 16.9, 10.2, 6.7 Hz, 1H), 5.02 – 4.85 (m, 2H), 3.66 (t, *J* = 7.3 Hz, 2H), 2.03 (q, *J* = 7.0 Hz, 2H), 1.72 – 1.61 (m, 2H), 1.48 – 1.27 (m, 4H); **^{13}C NMR (101 MHz, CDCl$_3$)** δ 168.4, 138.6, 133.8, 132.2, 123.1, 114.4, 37.9, 33.5, 28.4, 26.2; **ATR-FTIR (cm $^{-1}$):** 2931, 2857, 1704, 1394, 1364, 1044, 910, 716, 529; **HRMS m/z (ESI)** calculated for $C_{15}H_{18}NO_2^+$ [M+H]$^+$ 244.1332, found 244.1326. Spectroscopic data are in agreement with the literature.[9]

2-(hept-6-en-1-yl-3,3-*d*$_2$)isoindoline-1,3-dione (9)

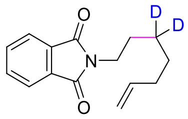

According to General procedure F, the crude product was purified by flash column chromatography on silica gel (PE : EA = 10:1) to afford **9** (24.8 mg, 51% yield, 97% D) as a colorless oil: **^{1}H NMR (400 MHz, CDCl$_3$)** δ 7.82 (dd, *J* = 5.4, 3.1 Hz, 2H), 7.69 (dd, *J* = 5.4, 3.0 Hz, 2H), 5.77 (ddt, *J* = 16.9, 10.2, 6.7 Hz, 1H), 5.01 – 4.86 (m, 2H), 3.71 – 3.60 (m, 2H), 2.10 – 1.95 (m, 2H), 1.65 (t, *J* = 7.3 Hz, 2H), 1.40 (t, *J* = 7.5 Hz, 2H); **^{13}C NMR (101 MHz, CDCl$_3$)** δ 168.4, 138.7, 133.8, 132.1, 123.1, 114.4, 37.9, 33.4, 28.2, 28.2; **ATR-FTIR (cm $^{-1}$):** 2924, 2857, 1705, 1394, 1365, 1030, 909, 716, 529; **HRMS m/z (ESI)** calculated for $C_{15}H_{16}D_2NO_2^+$ [M+H]$^+$ 246.1458, found 246.1450.

2-(hept-6-en-1-yl-2,2-*d*$_2$)isoindoline-1,3-dione (10)

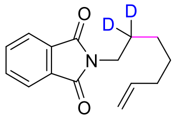

According to General procedure F, the crude product was purified by flash column chromatography on silica gel (PE : EA = 10:1) to afford **10** (24.5 mg, 50% yield, 95% D) as a colorless oil: **^{1}H NMR (400 MHz, CDCl$_3$)** δ 7.83 (dd, *J* = 5.2, 3.1 Hz, 2H), 7.70 (dd, *J* = 5.2, 3.0 Hz, 2H), 5.77 (dq, *J* = 10.3, 6.7 Hz, 1H), 4.94 (dd, *J* = 23.9, 13.6 Hz, 2H), 3.65 (s, 2H), 2.03 (q, *J* = 6.9 Hz, 2H), 1.47 – 1.29 (m, 2H), 1.37 – 1.28 (m, 2H); **^{13}C NMR (101 MHz, CDCl$_3$)** δ 168.4, 138.7, 133.8, 132.2, 123.1, 114.4, 37.8, 33.5, 28.3, 26.1; **ATR-FTIR (cm $^{-1}$):** 2925, 2857, 1706, 1391, 1353, 995, 911, 717, 530; **HRMS m/z (ESI)** calculated for $C_{15}H_{16}D_2NO_2^+$ [M+H]$^+$ 246.1458, found 246.1450.

2-(hept-6-en-1-yl-2,2,3,3-*d*$_4$)isoindoline-1,3-dione (11)

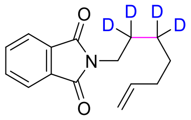

According to General procedure F, the crude product was purified by flash column chromatography on silica gel (PE : EA = 10:1) to afford **11** (25.5 mg, 52% yield, 95% D, 97% D) as a colorless oil: **^{1}H NMR (400 MHz, CDCl$_3$)** δ 7.85 (dd, J = 5.3, 3.1 Hz, 2H), 7.72 (dd, J = 5.2, 3.1 Hz, 2H), 5.80 (ddt, J = 16.9, 10.2, 6.7 Hz, 1H), 4.97 (dd, J = 23.8, 13.6 Hz, 2H), 3.68 (s, 2H), 2.06 (q, J = 7.0 Hz, 2H), 1.43 (t, J = 7.4 Hz, 2H); **^{13}C NMR (101 MHz, CDCl$_3$)** δ 168.4, 138.7, 133.8, 132.2, 123.1, 114.4, 37.8, 33.5, 28.1; **ATR-FTIR (cm $^{-1}$):** 2923, 2855, 1706, 1391, 1353, 994, 910, 713, 530; **HRMS m/z (ESI)** calculated for $C_{15}H_{14}D_4NO_2^+$ [M+H]$^+$ 248.1583, found 248.1576.

6. Total synthesis of deuterated drugs

(a) The 5 mmol scale reaction of 2a and 4a

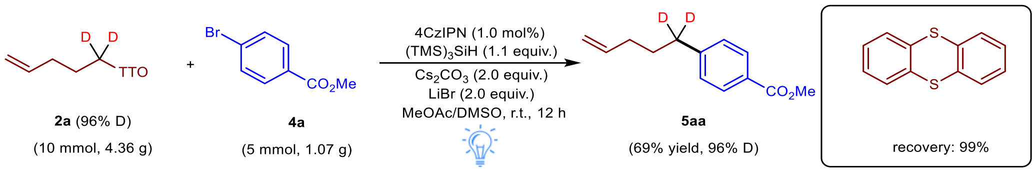

In a nitrogen-filled glove box, to a 100 mL Schlenk flask equipped with a stir bar was added **2a** (4.36 g, 10 mmol, 2 equiv.), **4a** (1.07 g, 5 mmol, 1 equiv.), Cs_2CO_3 (3.25 g, 10 mmol, 2 equiv.), LiBr (868.5 mg, 10 mmol, 2 equiv.), 4CzIPN (39.4 mg, 1 mol%) and $NiBr_2$•dtbpy (121.8 mg, 5 mol%). Then, anhydrous MeOAc (40 mL) and anhydrous DMSO (10 mL) were added via syringe, followed by addition of tris(trimethylsilyl)silane (1.7 mL, 5.5 mmol, 1.1 equiv.). The flask was sealed and removed from the glovebox. Subsequently, the reaction mixture was stirred and irradiated with a 40 W blue LED lamp for 12 hours. The final reaction mixture was diluted with EtOAc (200 mL) and saturated aqueous LiCl solution (60 mL). The organic layer was washed with brine (2 x 60 mL) and concentrated. Purification by flash column chromatography on silica gel (PE to PE: EA = 50:1) to afford 5aa (711.0 mg, 69% yield, 96% D) as a colorless oil and recover thianthrene (2.15 g) as a white solid.

(b) Synthesis of d$_2$-Prothionamide

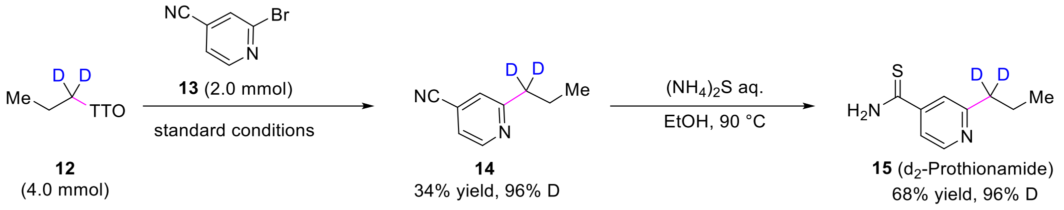

Step 1: In a nitrogen-filled glove box, to an oven-dried schlenk tube equipped with a stir bar was added **12** (1641.9 mg, 4 mmol, 2 equiv.), **13** (366.0 mg, 2 mmol, 1 equiv.), Cs_2CO_3 (1303.2 mg, 4 mmol, 2 equiv.), LiBr (347.4 mg, 4 mmol, 2 equiv.), 4CzIPN (15.8 mg, 1 mol%) and $NiBr_2$•dtbpy (48.7 mg, 5 mol%). Then, anhydrous MeOAc (16 mL) and anhydrous DMSO (4 mL) were added via syringe, followed by addition of tris(trimethylsilyl)silane (680 µL, 2.2 mmol, 1.1 equiv.). The tube was sealed and removed from the glovebox. Subsequently, the reaction mixture was stirred and irradiated with a 40 W blue LED lamp for 12 hours. The final reaction mixture was diluted with EtOAc (120 mL) and saturated aqueous LiCl solution (40 mL). The organic layer was washed with brine (2 x 40 mL) and concentrated. Purification by flash column chromatography on silica gel (PE : Et_2O = 2:1) to afford **14** (101.9 mg, 34% yield, 96% D) as a colorless oil.

Step 2: Compound **14** was dissolved in absolute ethanol (10 mL), 20 WT.% ammonium sulfide (1.1 mL, 5 eq) was added dropwise and the solution was refluxed at 90 °C for 3 hours. The completion of the reaction was monitored by TLC. The reaction mixture was diluted with EtOAc (80 mL) and brine (20 mL). The organic layer was washed with brine (2 x 20 mL) and concentrated. Purification by flash column chromatography on silica gel (DCM: MeOH = 20:1) to afford **15** (84.2 mg, 68% yield, 96% D) as a yellow solid.

5-(butyl-1,1-*d*$_2$)-5*H*-thianthren-5-ium trifluoromethanesulfonate (12)

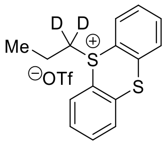

^{1}H NMR (400 MHz, CDCl$_3$) δ 8.28 (dd, *J* = 7.9, 1.1 Hz, 2H), 7.87 (dd, *J* = 7.9, 1.1 Hz, 2H), 7.79 (td, *J* = 7.7, 1.4 Hz, 2H), 7.68 (td, *J* = 7.8, 1.3 Hz, 2H), 1.59 (q, *J* = 7.3 Hz, 2H), 1.04 (t, *J* = 7.4 Hz, 3H); **^{13}C NMR (101 MHz, CDCl$_3$)** δ 135.6, 134.5, 134.5, 130.1, 129.8, 120.8 (q, *J* = 320.6 Hz), 117.0, 18.0, 12.1; **^{19}F NMR (376 MHz, CDCl$_3$)** δ -78.1; **ATR-FTIR (cm $^{-1}$):** 2971, 2361, 1453, 1254, 1153, 1026, 763, 633, 516, 455; **HRMS m/z (ESI)** calculated for $C_{15}H_{13}D_2S_2^+$ $[M - O_3SCF_3]^+$ 261.0735, found 261.0745.

5-butyl-5*H*-thianthren-5-ium trifluoromethanesulfonate (12')

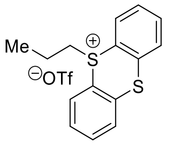

^{1}H NMR (400 MHz, CDCl$_3$) δ 8.20 (dd, *J* = 7.9, 1.2 Hz, 2H), 7.81 (dd, *J* = 8.0, 1.2 Hz, 2H), 7.74 (td, *J* = 7.7, 1.4 Hz, 2H), 7.62 (td, *J* = 7.7, 1.4 Hz, 2H), 3.75 – 3.65 (m, 2H), 1.62 – 1.45 (m, 2H), 0.98 (t, *J* = 7.4 Hz, 3H); **^{13}C NMR (101 MHz, CDCl$_3$)** δ 135.6, 134.5, 134.3, 130.0, 129.7, 120.7 (q, *J* = 320.8 Hz), 116.8, 41.9, 18.2, 12.1; **^{19}F NMR (376 MHz, CDCl$_3$)** δ -78.1; **ATR-FTIR (cm $^{-1}$):** 2995, 2361, 1453, 1253, 1152, 1026, 764, 633, 516, 456; **HRMS m/z (ESI)** calculated for $C_{15}H_{15}S_2^+$ [M – O_3SCF_3]$^+$ 259.0610, found 259.0618.

2-(propyl-1,1-*d*$_2$)isonicotinonitrile (14)

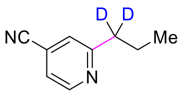

^{1}H NMR (400 MHz, CDCl$_3$) δ 8.70 (dd, *J* = 5.0, 1.0 Hz, 1H), 7.39 – 7.36 (m, 1H), 7.34 (dd, *J* = 5.0, 1.5 Hz, 1H), 1.76 (q, *J* = 7.3 Hz, 2H), 0.97 (t, *J* = 7.4 Hz, 3H); **^{13}C NMR (101 MHz, CDCl$_3$)** δ 164.0, 150.2, 124.3, 122.3, 120.6, 116.7, 22.6, 13.6; **ATR-FTIR (cm $^{-1}$):** 2961, 2931, 2873, 2238, 1593, 1548, 1471, 1394, 906, 839, 728, 468; **HRMS m/z (ESI)** calculated for $C_9H_9D_2N_2^+$ [M+H]$^+$ 149.1042, found 149.1043. Spectroscopic data of the corresponding non-deuterated product agree with those reported in literature.[10]

2-(propyl-1,1-*d*$_2$)pyridine-4-carbothioamide (15)

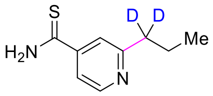

^{1}H NMR (400 MHz, DMSO-*d*$_6$) δ 10.15 (brs, 1H), 9.72 (brs, 1H), 8.53 (dd, *J* = 5.1, 0.8 Hz, 1H), 7.56 (dd, *J* = 1.7, 0.8 Hz, 1H), 7.51 (dd, *J* = 5.2, 1.8 Hz, 1H), 1.68 (q, *J* = 7.3 Hz, 2H), 0.90 (t, *J* = 7.4 Hz, 3H); **^{13}C NMR (101 MHz, DMSO-*d*$_6$)** δ 198.6, 161.9, 149.1, 146.6, 119.6, 118.3, 22.2, 13.6; **ATR-FTIR (cm $^{-1}$):** 3262, 2959, 2873, 1670, 1594, 1416, 1279, 923, 808, 750, 536, 406; **HRMS m/z (ESI)** calculated for $C_9H_{11}D_2N_2S^+$ [M+H]$^+$ 183.0920, found 183.0919.

The characterization data of the corresponding non-deuterated product are as follows:

2-(propyl)pyridine-4-carbothioamide (15')

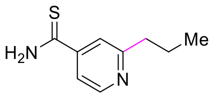

^{1}H NMR (400 MHz, DMSO-*d*$_6$) δ 10.14 (brs, 1H), 9.72 (brs, 1H), 8.53 (dd, *J* = 5.1, 0.6 Hz, 1H), 7.56 (d, *J* = 1.0 Hz, 1H), 7.51 (dd, *J* = 5.1, 1.8 Hz, 1H),

2.78 – 2.69 (m, 2H), 1.77 – 1.63 (m, 2H), 0.91 (t, J = 7.4 Hz, 3H); **^{13}C NMR (101 MHz, DMSO-*d*$_6$)** δ 198.6, 161.9, 149.1, 146.6, 119.6, 118.3, 39.5, 22.3, 13.7; **ATR-FTIR (cm $^{-1}$):** 3233, 2956, 2867, 1666, 1600, 1428, 1279, 1122, 834, 697, 515, 413; **HRMS m/z (ESI)** calculated for $C_9H_{13}N_2S^+$ $[M+H]^+$ 181.0794, found 181.0795. Spectroscopic data are in agreement with the literature.[11]

(c) Synthesis of d$_2$-Metoprolol

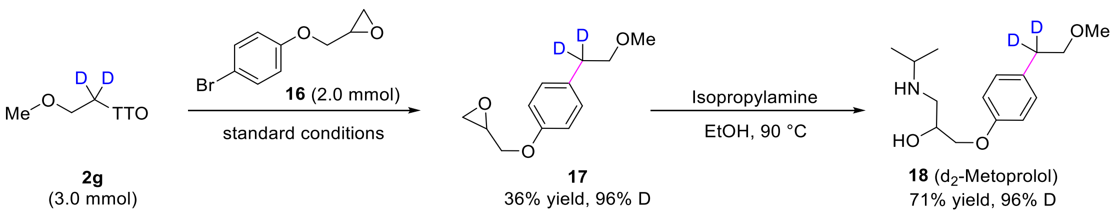

Step 1: In a nitrogen-filled glove box, to an oven-dried schlenk tube equipped with a stir bar was added **16** (458.2 mg, 2 mmol, 1 equiv.), **2g** (1.28 g, 3 mmol, 2 equiv.), Cs_2CO_3 (1303.2 mg, 4 mmol, 2 equiv.), LiBr (347.4 mg, 4 mmol, 2 equiv.), 4CzIPN (15.8 mg, 1 mol%) and $NiBr_2$•dtbpy (48.7 mg, 5 mol%). Then, anhydrous MeOAc (16 mL) and anhydrous DMSO (4 mL) were added via syringe, followed by addition of tris(trimethylsilyl)silane (680 μL, 2.2 mmol, 1.1 equiv.). The tube was sealed and removed from the glovebox. Subsequently, the reaction mixture was stirred and irradiated with a 40 W blue LED lamp for 12 hours. The final reaction mixture was diluted with EtOAc (120 mL) and saturated aqueous LiCl solution (40 mL). The organic layer was washed with brine (2 x 40 mL) and concentrated. Purification by flash column chromatography on silica gel (PE : EA = 6:1) to afford **17** (153.2 mg, 36% yield, 96% D) as a colorless oil.

Step 2: Compound **17** and propan-2-amine (425.6 mg, 7.2 mmol, 10 equiv.) were combined in EtOH (8 mL) and refluxed at 90 °C for 12 h. The completion of the reaction was monitored by TLC. The reaction mixture was cooled to room temperature and concentrated under reduced pressure. Purification by flash column chromatography on silica gel (DCM : MeOH = 20:1, 5% Et_3N) to afford **18** (137.8 mg, 71% yield, 96% D) as a white solid.

2-((4-(2-methoxyethyl-1,1-*d*$_2$)phenoxy)methyl)oxirane (17)

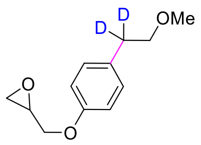

^{1}H NMR (400 MHz, CDCl$_3$) δ 7.17 – 7.10 (m, 2H), 6.90 – 6.83 (m, 2H), 4.18 (dd, J = 11.0, 3.3 Hz, 1H), 3.95 (dd, J = 11.0, 5.6 Hz, 1H), 3.55 (s, 2H), 3.37 – 3.31 (m, 4H), 2.90 (t, J = 4.5 Hz, 1H), 2.75 (dd, J = 4.9, 2.6 Hz, 1H); **^{13}C NMR (101 MHz, CDCl$_3$)** δ 157.0, 131.6, 129.8, 114.6, 73.7, 68.8, 58.7, 50.2, 44.8; **ATR-FTIR (cm $^{-1}$):** 2924, 2872, 1612, 1510, 1237, 1123, 1034, 916, 838, 567; **HRMS m/z (ESI)** calculated for $C_{12}H_{14}D_2O_3Na^+$ $[M+Na]^+$ 233.1117, found 233.1114.

The characterization data of the corresponding non-deuterated product are as follows:

2-((4-(2-methoxyethyl)phenoxy)methyl)oxirane (17')

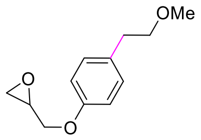

^{1}H NMR (400 MHz, CDCl$_3$) δ 7.17 – 7.08 (m, 2H), 6.89 – 6.81 (m, 2H), 4.18 (dd, J = 11.1, 3.2 Hz, 1H), 3.94 (dd, J = 11.1, 5.6 Hz, 1H), 3.56 (t, J = 7.1 Hz, 2H), 3.38 – 3.29 (m, 4H), 2.93 – 2.87 (m, 1H), 2.82 (t, J = 7.1 Hz, 2H), 2.74 (dd, J = 5.0, 2.6 Hz, 1H); **^{13}C NMR (101 MHz, CDCl$_3$)** δ 157.0, 131.6, 129.8, 114.6, 73.7, 68.8, 58.6, 50.1, 44.6, 35.2; **ATR-FTIR (cm $^{-1}$):** 2923, 2868, 1612, 1510, 1240, 1110, 1033, 915, 829, 577; **HRMS m/z (ESI)** calculated for $C_{12}H_{16}O_3Na^+$ $[M+Na]^+$ 231.0990, found 231.0992. Spectroscopic data are in agreement with the literature.[12]

1-(isopropylamino)-3-(4-(2-methoxyethyl-1,1-*d*$_2$)phenoxy)propan-2-ol (18)

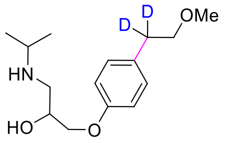

^{1}H NMR (400 MHz, CDCl$_3$) δ 7.14 – 7.08 (m, 2H), 6.86 – 6.80 (m, 2H), 4.09 – 3.99 (m, 1H), 3.98 – 3.87 (m, 2H), 3.54 (s, 2H), 3.33 (s, 3H), 3.07 (brs, 2H), 2.91 – 2.80 (m, 2H), 2.72 (dd, J = 12.0, 8.2 Hz, 1H), 1.09 (d, J = 6.3 Hz, 6H); **^{13}C NMR (101 MHz, CDCl$_3$)** δ 157.1, 131.2, 129.7, 114.4, 73.6, 70.6, 68.2, 58.5, 49.3, 49.0, 22.6, 22.6; **ATR-FTIR (cm $^{-1}$):** 3298, 3034, 2922, 2867, 1612, 1511, 1240, 1121, 1034, 806, 553; **HRMS m/z (ESI)** calculated for $C_{15}H_{24}D_2NO_3^+$ $[M+H]^+$ 270.2033, found 270.2028.

The characterization data of the corresponding non-deuterated product are as follows:

1-(isopropylamino)-3-(4-(2-methoxyethyl)phenoxy)propan-2-ol (18')

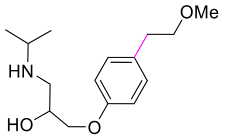

^{1}H NMR (400 MHz, CDCl$_3$) δ 7.14 (d, J = 8.3 Hz, 2H), 6.86 (d, J = 8.3 Hz, 2H), 4.09 – 4.00 (m, 1H), 4.00 – 3.90 (m, 2H), 3.57 (t, J = 7.1 Hz, 2H), 3.36

(s, 3H), 2.93 – 2.66 (m, 7H), 1.10 (d, *J* = 6.3 Hz, 6H); **^{13}C NMR (101 MHz, $CDCl_3$)** δ 157.1, 131.3, 129.7, 114.4, 73.8, 70.6, 68.4, 58.5, 49.4, 48.8, 35.2, 22.9, 22.9; **ATR-FTIR (cm $^{-1}$):** 3297, 3034, 2922, 2866, 1612, 1511, 1242, 1111, 1039, 824, 565; **HRMS m/z (ESI)** calculated for $C_{15}H_{26}NO_3^+$ $[M+H]^+$ 268.1913, found 268.1907. Spectroscopic data are in agreement with the literature.[13]

(d) Synthesis of d_2-Bezafibrate

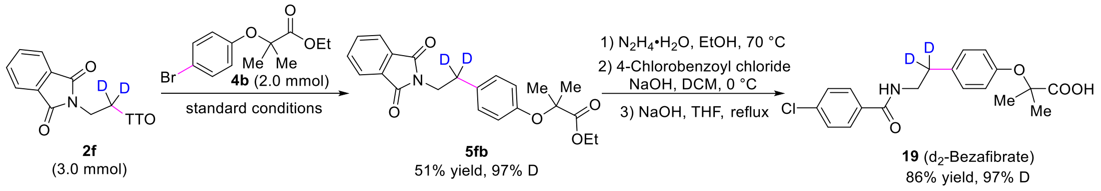

Step 1: In a nitrogen-filled glove box, to an oven-dried schlenk tube equipped with a stir bar was added **4b** (574.4.2 mg, 2 mmol, 1 equiv.), **2f** (1.62 g, 3 mmol, 1.5 equiv.), Cs_2CO_3 (1303.2 mg, 4 mmol, 2 equiv.), LiBr (347.4 mg, 4 mmol, 2 equiv.), 4CzIPN (15.8 mg, 1 mol%) and $NiBr_2$•dtbpy (48.7 mg, 5 mol%). Then, anhydrous MeOAc (16 mL) and anhydrous DMSO (4 mL) were added via syringe, followed by addition of tris(trimethylsilyl)silane (680 µL, 2.2 mmol, 1.1 equiv.). The tube was sealed and removed from the glovebox. Subsequently, the reaction mixture was stirred and irradiated with a 40 W blue LED lamp for 12 hours. The final reaction mixture was diluted with EtOAc (120 mL) and saturated aqueous LiCl solution (40 mL). The organic layer was washed with brine (2 x 40 mL) and concentrated. Purification by flash column chromatography on silica gel (PE : EA = 5:1) to afford **5fb** (387.0 mg, 51% yield, 97% D) as a colorless oil.

Step 2: N_2H_4·xH_2O (1 mL) was added to a solution of **5fb** (387.0 mg) in absolute EtOH (10 mL). The reaction mixture was stirred for 2 h at reflux and overnight at rt. The suspension was filtered, and the organic solvent was evaporated in vacuo to give a yellow solid which was dissolved in ethyl acetate. The solution was washed with brine, dried over Na_2SO_4, and the organic solvent was evaporated to afford the desired amine without any further purification. Then 4-chlorobenzoyl chloride (525.0 mg, 3 mmol) was added to a mixture of the crude amine, DCM (10 mL) and aqueous NaOH (4M, 0.5 mL) at 0 ºC. The mixture was stirred at 0 ºC for 30 min. The organic layer was separated by separatory funnel. And the organic layer was washed with aqueous HCl (1M, 5 mL), brine (10 mL) and dried over Na_2SO_4. The solvent was removed under reduced pressure to give a

crude product and purified by flash column chromatography on silica gel (PE : EA = 3:1) to afford ethyl 2-(4-(2-(4-chlorobenzamido)ethyl-1,1-d_2)phenoxy)-2-methylpropanoate (353.9 mg, 90% yield , 97% D) as a white solid. Next, to a solution of ethyl 2-(4-(2-(4-chlorobenzamido)ethyl-1,1-d_2)phenoxy)-2-methylpropanoate (353.9 mg) in THF (20 mL) was added aqueous NaOH (4 M, 6 mL) and the resulting mixture was stirred at reflux for 8 h. Further portions of NaOH (300 mg, 7.50 mmol) were added at 2 h intervals throughout the course of the reaction. The reaction mixture was cooled to room temperature and concentrated *in vacuo*. The residue was dissolved in water (20 mL) and concentrated HCl was added dropwise. The resulting precipitate was filtered, washed with water and dried under high vacuum to afford the crude product. Purification by flash column chromatography on silica gel (DCM : MeOH = 20:1) to afford **19** (312.1 mg, 86% yield, 97% D) as a white solid.

ethyl 2-(4-(2-(1,3-dioxoisoindolin-2-yl)ethyl-1,1-*d*₂)phenoxy)-2-methylpropanoate (5fb)

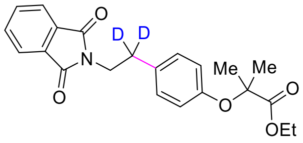

^{1}H NMR (400 MHz, CDCl$_3$) δ 7.81 (dd, *J* = 5.5, 3.0 Hz, 2H), 7.69 (dd, *J* = 5.5, 3.0 Hz, 2H), 7.14 – 7.08 (m, 2H), 6.80 – 6.73 (m, 2H), 4.21 (q, *J* = 7.1 Hz, 2H), 3.87 (s, 2H), 1.55 (s, 6H), 1.23 (t, *J* = 7.1 Hz, 3H); **^{13}C NMR (101 MHz, CDCl$_3$)** δ 174.2, 168.1, 154.0, 133.8, 132.0, 131.6, 129.4, 123.1, 119.4, 79.1, 61.3, 39.1, 25.3, 14.0; **ATR-FTIR (cm $^{-1}$):** 2987, 2939, 1708, 1508, 1392, 1135, 992, 715, 530; **HRMS m/z (ESI)** calculated for $C_{22}H_{21}D_2NO_5Na^+$ $[M+Na]^+$ 406.1594, found 406.1588.

The characterization data of the corresponding non-deuterated product are as follows:

ethyl 2-(4-(2-(1,3-dioxoisoindolin-2-yl)ethyl)phenoxy)-2-methylpropanoate (5fb')

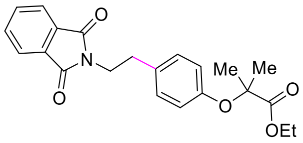

^{1}H NMR (400 MHz, CDCl$_3$) δ 7.81 (dd, *J* = 5.5, 3.0 Hz, 2H), 7.69 (dd, *J* = 5.4, 3.0 Hz, 2H), 7.14 – 7.08 (m, 2H), 6.79 – 6.73 (m, 2H), 4.21 (q, *J* = 7.1 Hz, 2H), 3.92 – 3.83 (m, 2H), 2.92 (dd, *J* = 8.6, 6.9 Hz, 2H), 1.55 (s, 6H), 1.23 (t, *J* = 7.1 Hz, 3H); **^{13}C NMR (101 MHz, CDCl$_3$)** δ 174.2, 168.1, 154.1, 133.8, 132.1, 131.7, 129.5, 123.1, 119.5, 79.1, 61.3, 39.3, 33.7, 25.3, 14.0; **ATR-FTIR (cm $^{-1}$):** 2986, 2939, 1707, 1508, 1393, 1136, 995, 717, 530; **HRMS m/z (ESI)** calculated for $C_{22}H_{23}NO_5Na^+$ $[M+Na]^+$ 404.1468, found 404.1460.

2-(4-(2-(4-chlorobenzamido)ethyl-1,1-d_2)phenoxy)-2-methylpropanoic acid (19)

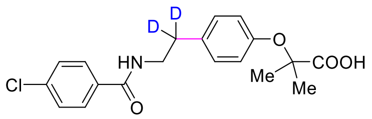

^{1}H NMR (400 MHz, DMSO-d_6) δ 12.96 (brs, 1H), 8.64 (t, J = 5.5 Hz, 1H), 7.89 – 7.76 (m, 2H), 7.58 – 7.46 (m, 2H), 7.19 – 7.06 (m, 2H), 6.84 – 6.65 (m, 2H), 3.44 (d, J = 5.6 Hz, 2H), 1.49 (s, 6H); **^{13}C NMR (101 MHz, DMSO-d_6)** δ 175.2, 165.1, 153.7, 135.9, 133.4, 132.5, 129.4, 129.1, 128.4, 118.6, 78.3, 41.0, 25.1; **ATR-FTIR (cm $^{-1}$):** 3358, 2989, 2863, 1716, 1608, 1547, 1263, 1149, 762, 528; **HRMS m/z (ESI)** calculated for $C_{19}H_{19}D_2ClNO_4^+$ [M+H]$^+$ 364.1279, found 364.1275.

The characterization data of the corresponding non-deuterated product are as follows:

2-(4-(2-(4-chlorobenzamido)ethyl)phenoxy)-2-methylpropanoic acid (19')

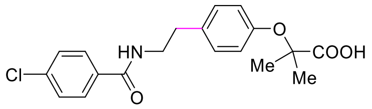

^{1}H NMR (400 MHz, DMSO-d_6) δ 12.95 (brs, 1H), 8.62 (t, J = 5.5 Hz, 1H), 7.83 (d, J = 8.6 Hz, 2H), 7.52 (d, J = 8.6 Hz, 2H), 7.12 (d, J = 8.6 Hz, 2H), 6.76 (d, J = 8.6 Hz, 2H), 3.56 – 3.34 (m, 2H), 2.77 (t, J = 7.5 Hz, 2H), 1.48 (s, 6H); **^{13}C NMR (101 MHz, DMSO-d_6)** δ 175.0, 165.0, 153.6, 135.8, 133.3, 132.6, 129.3, 129.0, 128.2, 118.6, 78.3, 41.0, 34.1, 25.0; **ATR-FTIR (cm $^{-1}$):** 3356, 2989, 2861, 1715, 1607, 1548, 1261, 1146, 760, 528; **HRMS m/z (ESI)** calculated for $C_{19}H_{21}ClNO_4^+$ [M+H]$^+$ 362.1154, found 362.1148. Spectroscopic data are in agreement with the literature.[14]

7. Mechanistic experiments

(a) UV-vis absorption study of the reaction components

UV-vis absorption of a solution of 4CzIPN (5 × 10^{-5} M in DMSO/MeOAc), $NiBr_2$•dtbpy (2.5 × 10^{-3} M in DMSO/MeOAc), **2b** (0.25 M in DMSO/MeOAc), **4a** (0.25 M in DMSO/MeOAc), **2b** with **4a** (0.25 M in DMSO/MeOAc), **2b** with LiBr (0.25 M in DMSO/MeOAc), and **2b** with TTMSSiH (0.25 M in DMSO/MeOAc) were prepared and measured respectively (Supplementary Figure 5).

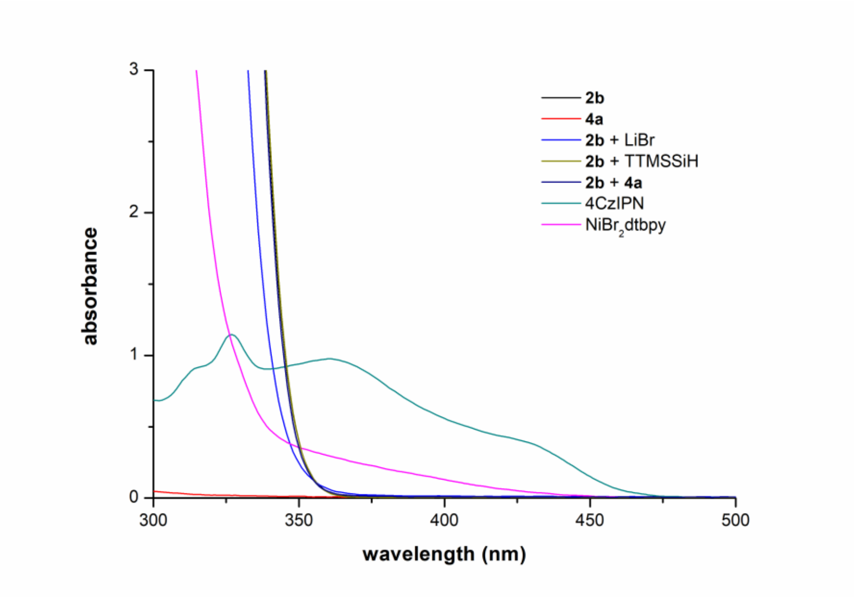

Supplementary Figure 5. UV-vis absorption spectra of the reaction components

(b) Tracing the reaction between 2b and 4a

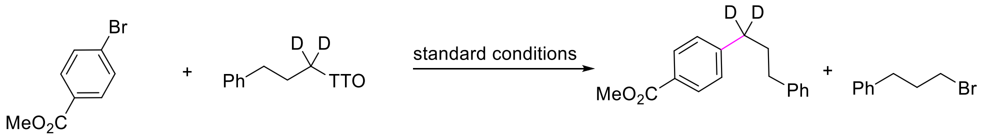

4a **2b** **5ba** **20**

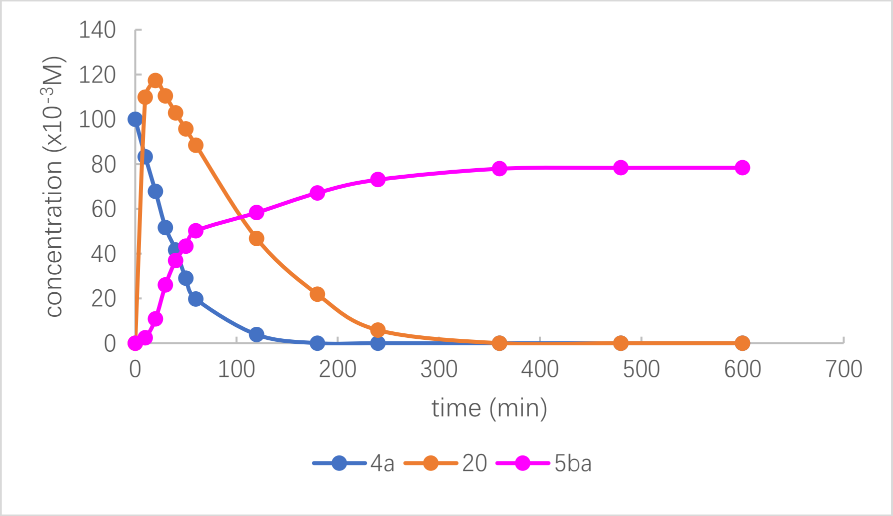

Supplementary Figure 6. Process and reaction monitoring by GC-MS

In a nitrogen-filled glove box, to an 8 mL oven-dried vial equipped with a stir bar was added **4a** (0.2 mmol, 1 equiv.), **2b** (0.4 mmol, 2 equiv.), Cs_2CO_3 (130.3 mg, 0.4 mmol, 2 equiv.), LiBr (34.7 mg, 0.4 mmol, 2 equiv.), 4CzIPN (1.8 mg, 1 mol%) and $NiBr_2$•dtbpy (4.9 mg, 5 mol%). Then, anhydrous MeOAc (1.6 mL) and anhydrous DMSO (0.4 mL) were added via syringe, followed by addition of tris(trimethylsilyl)silane (68 µL, 0.22 mmol, 1.1 equiv.). The vial was sealed and removed from the glovebox. Subsequently, the reaction mixture was stirred and irradiated with a 40 W blue LED lamp. After t min (t = 10, 20, 30, 40, 50, 60…), the reaction mixture was quenched exposure to air and monitored by Gas Chromatography-Mass Spectrometer (GC-MS) to determine the product yield with CH_2Br_2 as internal standard (Supplementary Figure 6). The result indicated that a large amount of 3-phenyl propyl bromide **20** was generated in the begin of the reaction. The rapidly converting alkyl thianthrenium salt to alkyl bromide may be important to the success of the reaction. Then, the desired crossing-coupling product **5ba** was formed by the consumption of 3-phenyl propyl bromide **20** and aryl bromide **4a**.

(c) Competition reaction between alkyl TT salt 2a and alkyl bromide 21

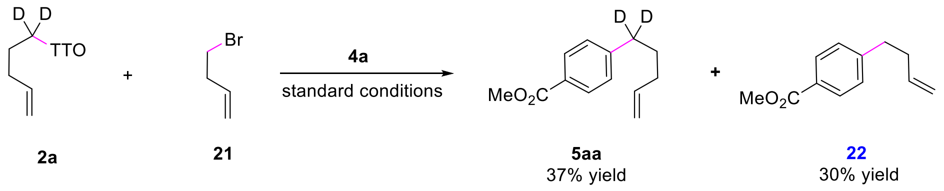

In a nitrogen-filled glove box, to an 8 mL oven-dried vial equipped with a stir bar was added **2a** (0.2 mmol, 1 equiv.), **4a** (0.2 mmol, 1 equiv.), **21** (0.2 mmol, 1 equiv.), Cs_2CO_3 (130.3 mg, 0.4 mmol, 2 equiv.), LiBr (34.7 mg, 0.4 mmol, 2 equiv.), 4CzIPN (1.8 mg, 1 mol%) and $NiBr_2$•dtbpy (4.9 mg, 5 mol%). Then, anhydrous MeOAc (1.6 mL) and anhydrous DMSO (0.4 mL) were added via syringe, followed by addition of tris(trimethylsilyl)silane (68 µL, 0.22 mmol, 1.1 equiv.). The vial was sealed and removed from the glovebox. Subsequently, the reaction mixture was stirred and irradiated with a 40 W blue LED lamp for 12 hours. The final reaction mixture was diluted with EtOAc (60 mL) and saturated aqueous LiCl solution (20 mL). The organic layer was washed with brine (2 x 20 mL) and concentrated. Yields of **5aa** and **22** were determined by crude ^1H NMR using CH_2Br_2 as internal standard (Supplementary Figure 7).

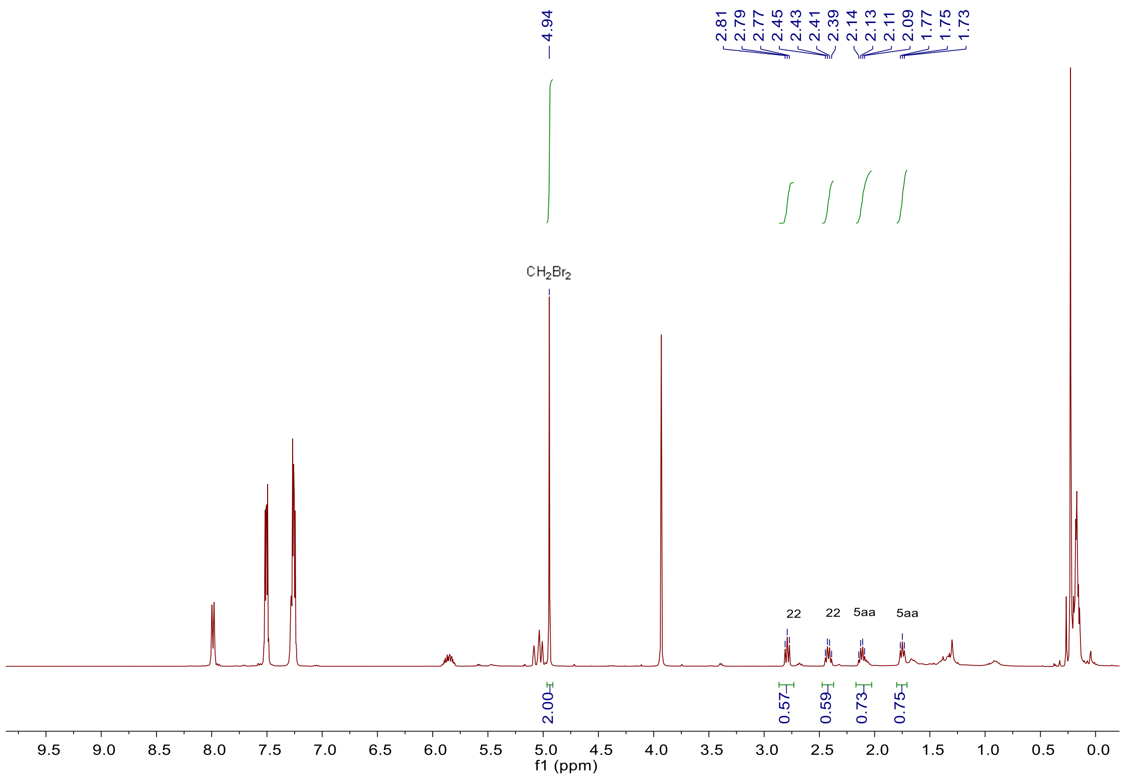

Supplementary Figure 7. ^{1}H NMR Mixture of **5aa** and **22**

(c) Radical trapping experiments

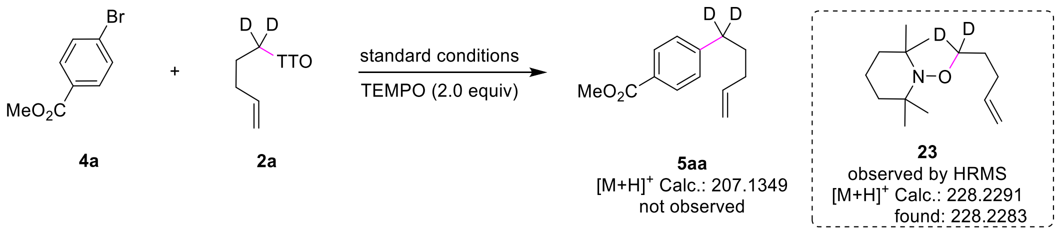

The reaction was conducted with **4a** (0.2 mmol) and **2a** (0.4 mmol) under the standard conditions with TEMPO (2,2,6,6-tetramethylpiperidine-1-oxyl, 2.0 equiv.). After 12 h, a drop of the reaction mixture (about 50 μL) was collected for HRMS (ESI) analysis without further work-up (Supplementary Figure 8). The adduct **23** was detected by HRMS (ESI): calcd for $C_{14}H_{28}NO^+$ $[M+H]^+$ 228.2291, found 228.2283. There was no corresponding product **5aa** detected.

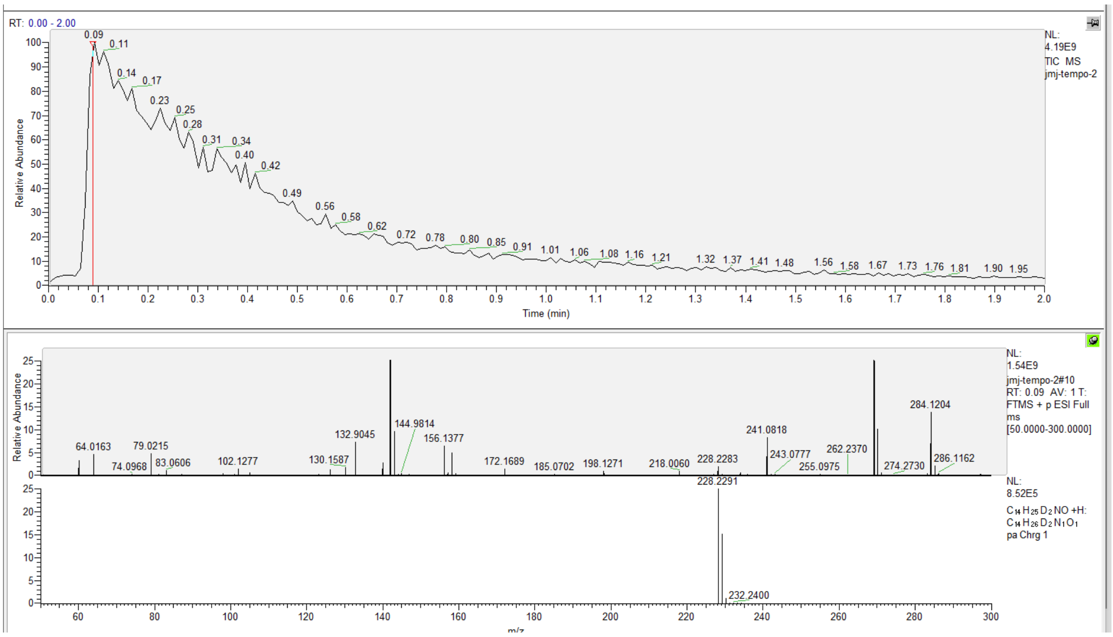

Supplementary Figure 8. HRMS analysis of **23.**

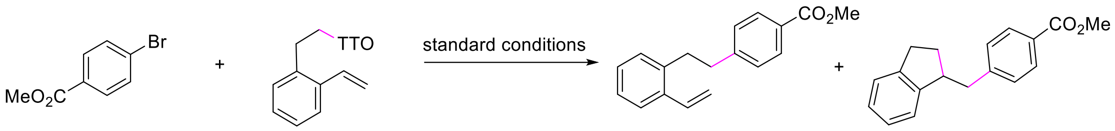

4a **24** **25** trace **26** 31% yield

The reaction was conducted with **4a** (0.2 mmol) and **24** (0.4 mmol) under the standard conditions for 12 h. The final reaction mixture was diluted with EtOAc (60 mL) and saturated aqueous LiCl solution (20 mL). The organic layer was washed with brine (2 x 20 mL) and concentrated. Purification by flash column chromatography on silica gel (PE : EA = 30:1) to afford **26** (16.5 mg, 31% yield) as a colorless oil. The trace amount of direct coupling product **25** could be observed by GC-MS.

methyl 4-((2,3-dihydro-1*H*-inden-1-yl)methyl)benzoate (26)

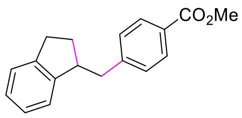

^{1}H NMR (400 MHz, CDCl$_3$) δ 7.99 (d, *J* = 8.2 Hz, 2H), 7.28 (d, *J* = 8.1 Hz, 2H), 7.24 (d, *J* = 6.8 Hz, 1H), 7.21 – 7.11 (m, 2H), 7.09 (d, *J* = 6.9 Hz, 1H), 3.93 (s, 3H), 3.56 – 3.40 (m, 1H), 3.19 (dd, *J* = 13.5, 5.9 Hz, 1H), 2.95 – 2.71 (m, 3H), 2.20 – 2.08 (m, 1H), 1.82 – 1.70 (m, 1H); **^{13}C NMR (101 MHz, CDCl$_3$)** δ 167.1, 146.4, 146.3, 144.0, 129.6, 129.1, 127.9, 126.6, 126.0, 124.6, 123.7, 52.0, 46.1, 41.4, 31.8, 31.1;

ATR-FTIR (cm^{-1}): 2949, 2848, 1719, 1609, 1435, 1276, 1178, 1109, 836, 757; **HRMS m/z (ESI)** calculated for $C_{18}H_{19}O_2^+$ $[M+H]^+$ 267.1380, found 267.1377.

8. The calculation of aqueous pKa values and BDE

Density functional theory (DFT) calculations are performed using the Gaussian 09 program package[15]. In principle, the absolute pKa values can be calculated with the following thermodynamic cycle[16]:

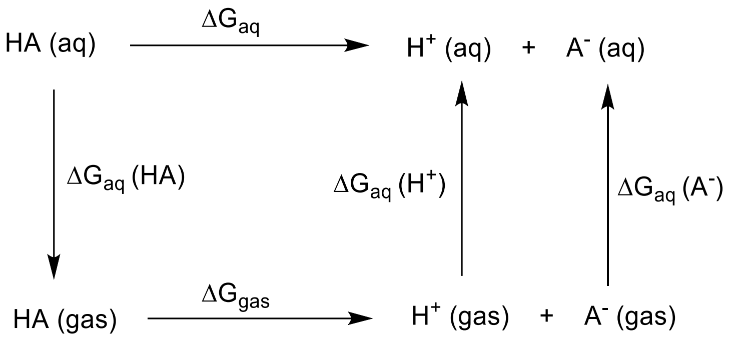

where

$$\Delta G_{aq} = \Delta G_{gas} + \Delta G_{aq}(H^+) + \Delta G_{aq}(A^-) - \Delta G_{aq}(HA)$$

Given that

$$pKa = -\log Ka$$

and

$$\Delta G_{aq} = -2.303RT\log Ka$$

Thus

$pKa = \Delta G_{aq}/2.303RT$

$\quad = [\Delta G_{gas} + \Delta G_{aq}(H^+) + \Delta G_{aq}(A^-) - \Delta G_{aq}(HA)]/2.303RT$

The values for ΔG_{gas} is calculated at the level of M062X[17] with a mixed basis set of SDD[18] for I atom and 6-311+g(2d,p)[19] for all other atoms in the gas phase, the $\Delta G_{aq}(A^-)$ and $\Delta G_{aq}(HA)$ values are calculated at the same level using a continuum model SMD[20] in aqueous phase. In the calculation process, ΔG_{gas} uses a reference state of 1 atm, while the ΔG_{aq} use a reference state of 1 M. Therefore, we use the following equation to convert the ΔG_{gas} from the reference state of 1 atm (24.46 L at 298.15 K) to 1 M:

$$\Delta G_{gas} = \Delta G_{gas}(1\ atm) + RT\ln(24.46)$$

The value of -6.28 and -264.61 kcal/mol derived from experiments were used for $\Delta G_{gas}(H^+)$ and

ΔG_{aq} (H^+), respectively [16a, 21].

$\therefore$ pKa = [ΔG_{gas} (1 atm) + RT ln (24.46) + ΔG_{aq} (H^+) + ΔG_{aq} (A^-) - ΔG_{aq} (HA)]/2.303RT

= [ΔG_{gas} (H^+) + ΔG_{gas} (A^-) - ΔG_{gas} (HA) + RT ln (24.46) + ΔG_{aq} (H^+) + ΔG_{aq} (A^-) - ΔG_{aq} (HA)]/2.303RT

= [ΔG_{gas} (A^-) - ΔG_{gas} (HA) + ΔG_{aq} (A^-) - ΔG_{aq} (HA) – 269.0]/1.3644

Supplementary Table 2: The calculated Gibbs Free energies of **1a** and alkyl (pseudo)halides

structure	G_{gas} (Hartree)	G_{aq} (kcal/mol)	pKa
**1a**	-1453.802262	-37.95	30.0
**1a-deprotonation**	-1453.368696	-0.03	
**I**	-655.990370	-0.54	48.3
**I-deprotonation**	-655.370388	-54.62	
**II**	-2769.960026	-0.25	49.3
**II-deprotonation**	-2769.347054	-48.69	
**III**	-207.161929	-1.7	46.2
**III-deprotonation**	-206.553839	-51.25	
**IV**	-1090.409405	-4.24	48.7
**IV-deprotonation**	-1089.792667	-55.78	
**V**	-1157.215463	-0.69	43.8
**V-deprotonation**	-1156.618114	-46.71	

For the reaction as follow:

$$A-B \longrightarrow A\bullet + B\bullet$$

the BDE was calculated as follow[22]

$$BDE = \Delta_f H_T(A\cdot) + \Delta_f H_T(B\cdot) - \Delta_f H_T(AB)$$

Supplementary Table 3: The calculated enthalpy of **1a** and alkyl (pseudo)halides

structure	$\Delta_f H_T$ (Hartree)	BDE (kcal/mol)
**1a**	-1453.737901	56.6
**alkyl-radical**	-195.684198	

$TT^{+\cdot}$	-1257.963528	
**I**	-655.949312	84.5
Cl·	-460.130457	
**II**	-2769.917873	70.2
Br·	-2574.121752	
**III**	-207.118642	62.1
I·	-11.335486	
**IV**	-1090.343324	93.6
·OTs	-894.509986	
**V**	-1157.157929	98.9
·OTf	-961.316186	

Geometry optimizations for all the intermediates and transition structures were calculated with B3LYP[23] level of theory and a mix basis set of SDD (I atom) and the 6-31G(d)[24] (C, H, O, S, F, Cl, and Br) in gas phase. Vibrational frequency calculations were carried out for all stationary points at the same level to obtain the thermal corrections for the free energies and to confirm if each optimized structure is a local minimum or a transition state. The solvation single-point energies were calculated at the M062X functional with large basis set of 6-311+G(2d,p) and SDD in dichloromethane solvent (SMD). The sum of the thermal correction to free energy and the single-point energy is taken as the Gibbs free energy for intermediates or transition states. The calculated optimized structures are visualized using CYLview. [25]

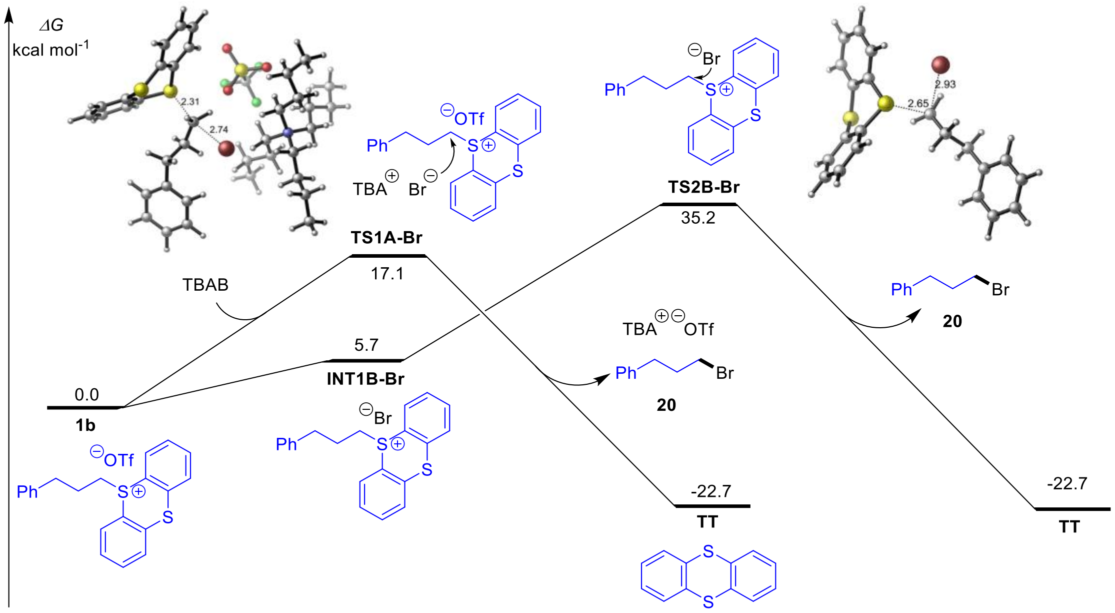

Supplementary Figure 9. DFT-calculated energy profile for the reaction of alkyl TT salt **1b** and TBAB.

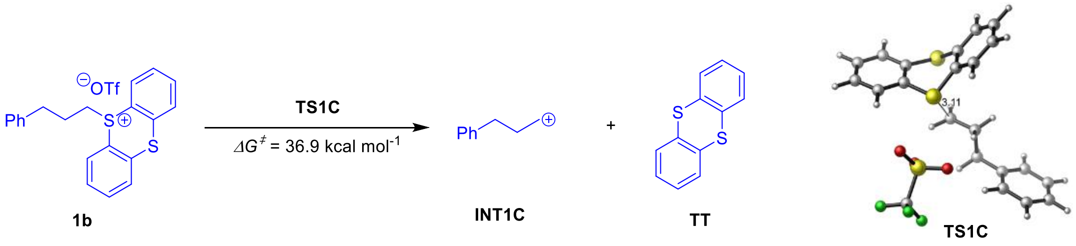

Supplementary Figure 10. The direct C-S bond cleavage.

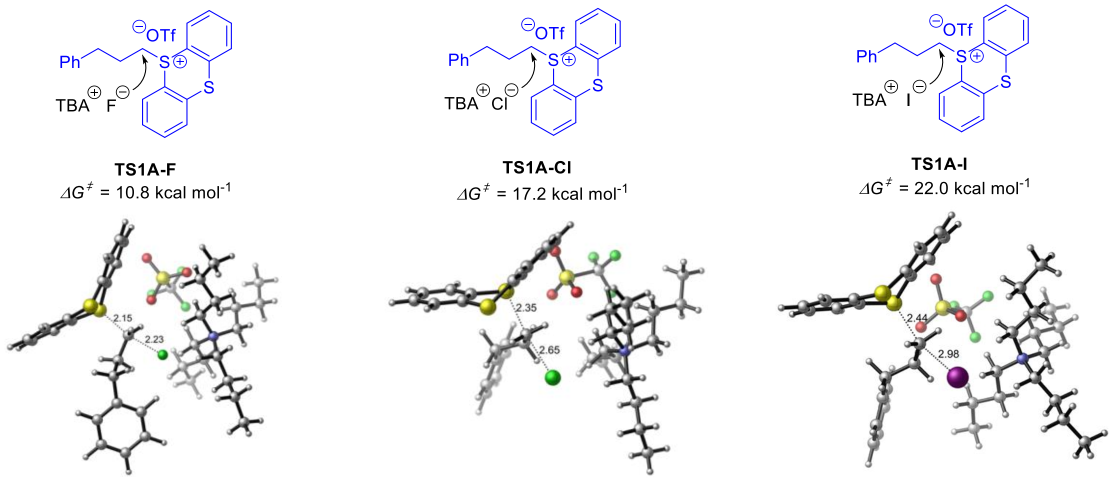

Supplementary Figure 11. The key transition states for other halogenated reagents.

9. NMR Spectra

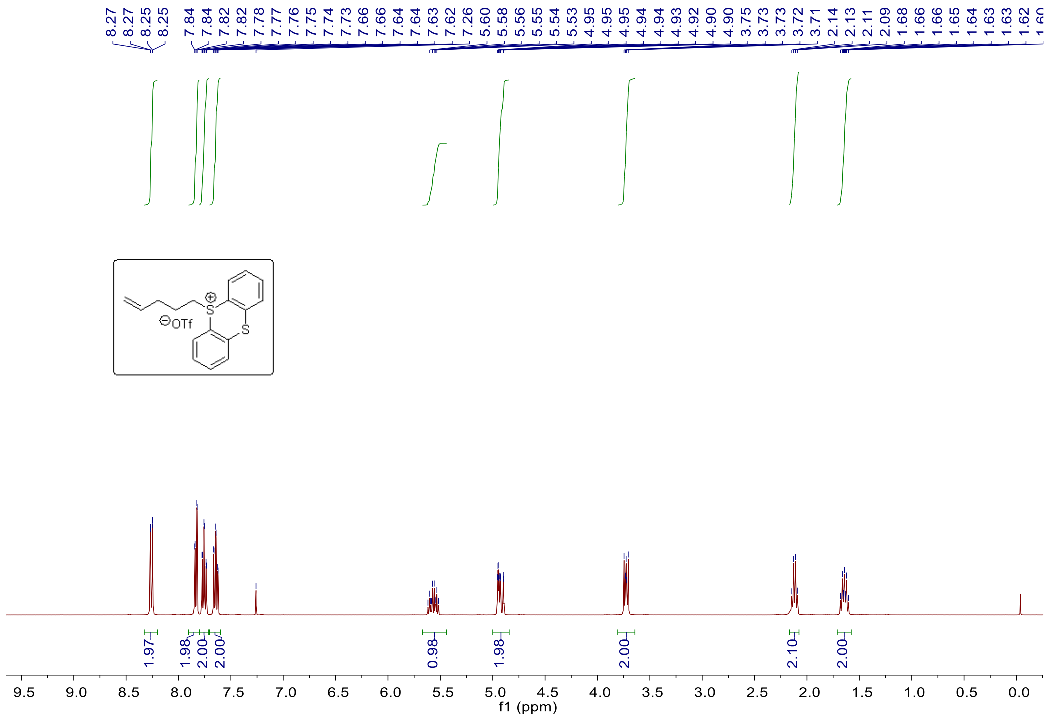

Supplementary Figure 12. ^{1}H NMR (400 MHz, $CDCl_3$) of **1a**

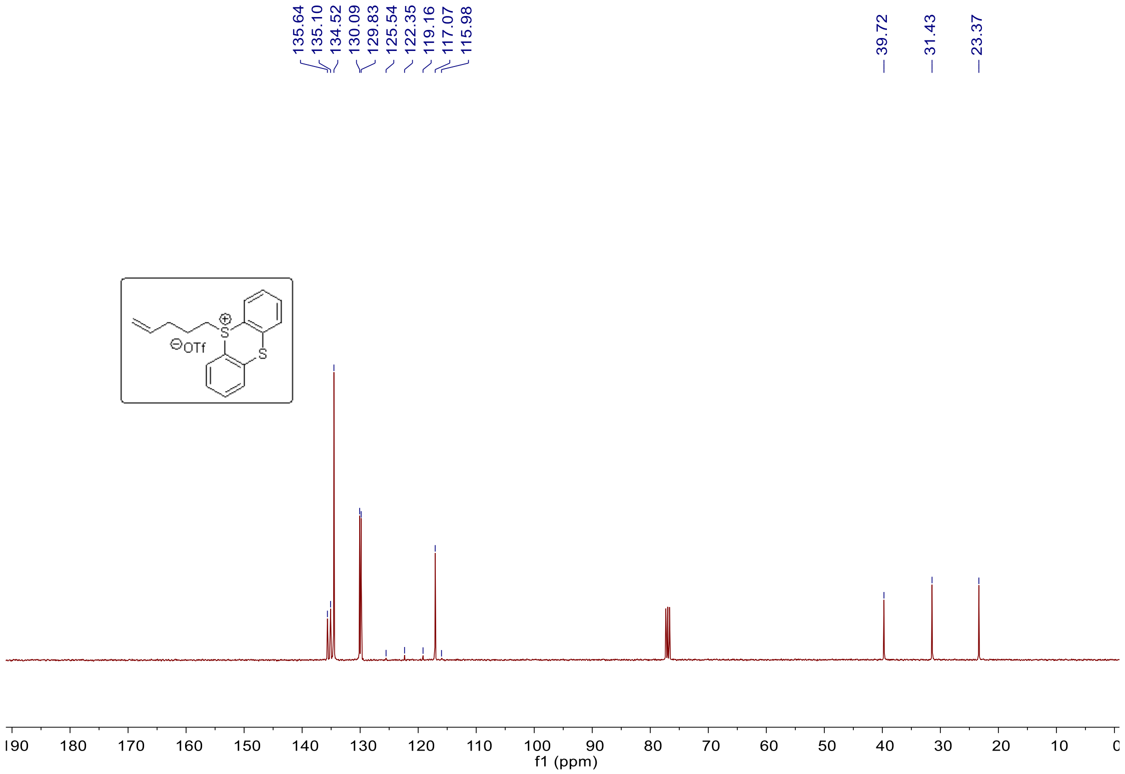

Supplementary Figure 13. ^{13}C NMR (101 MHz, $CDCl_3$) of **1a**

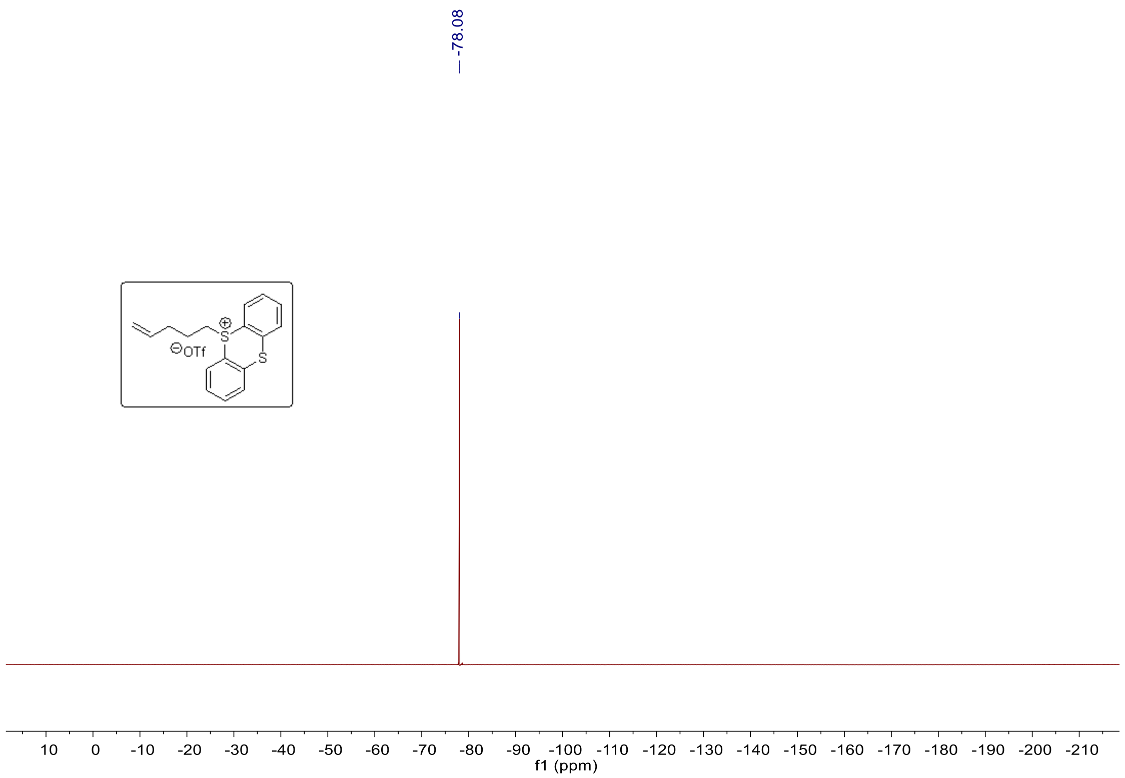

Supplementary Figure 14. ^{19}F NMR (376 MHz, $CDCl_3$) of **1a**

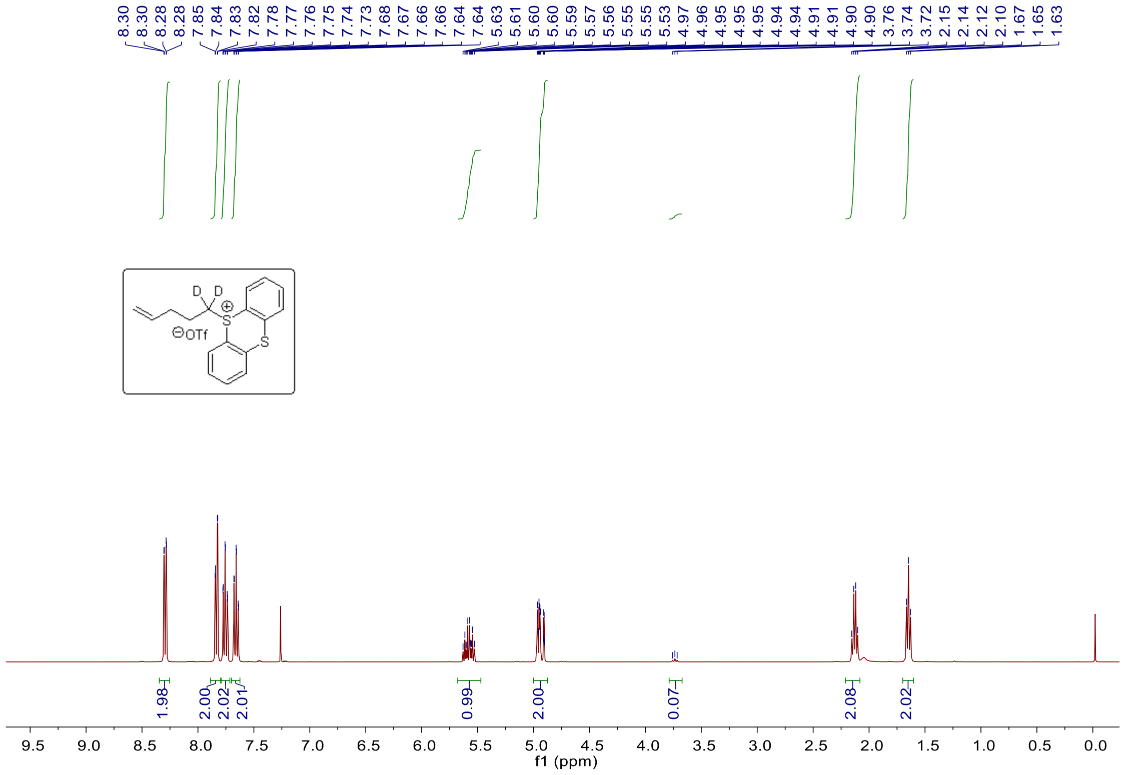

Supplementary Figure 15. ^{1}H NMR (400 MHz, $CDCl_3$) of **2a**

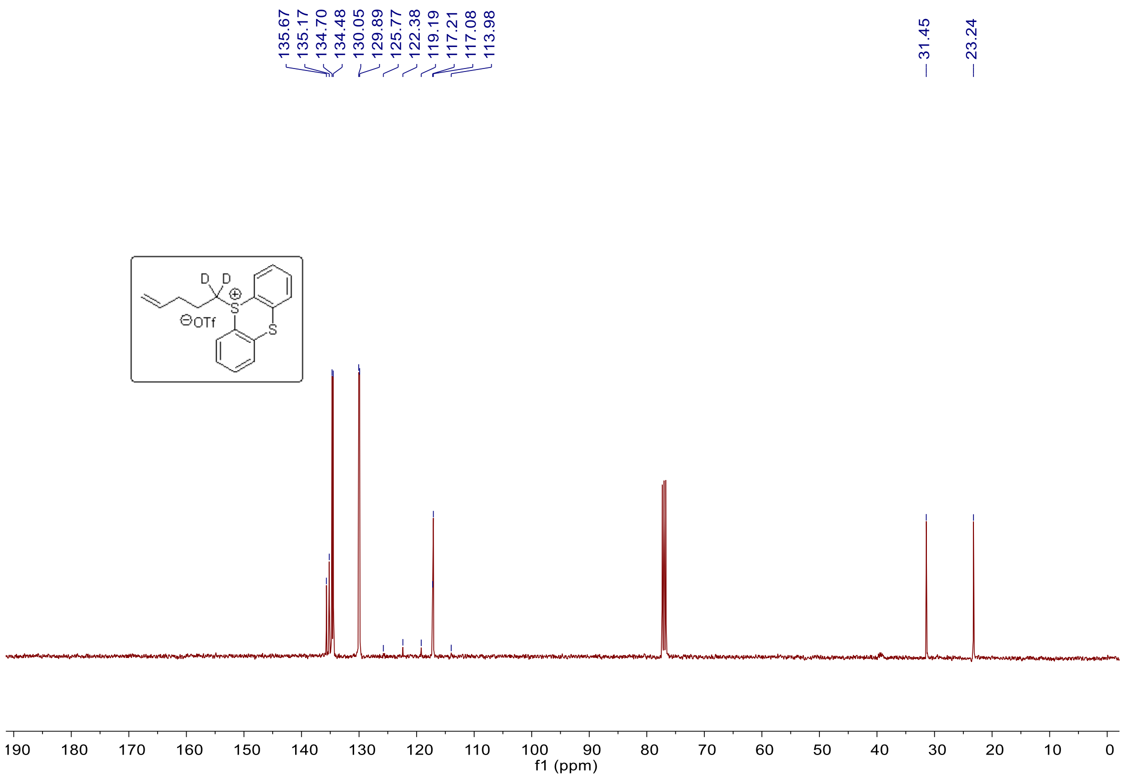

Supplementary Figure 16. ^{13}C NMR (101 MHz, $CDCl_3$) of **2a**

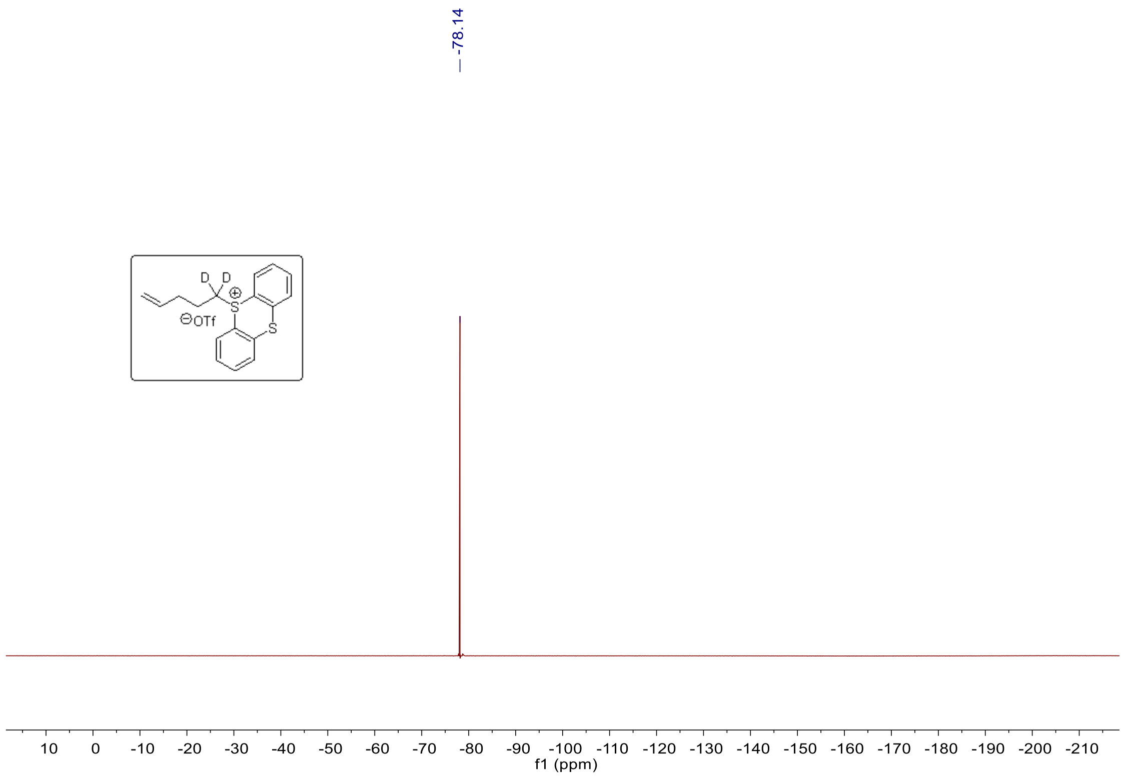

Supplementary Figure 17. ^{19}F NMR (376 MHz, $CDCl_3$) of **2a**

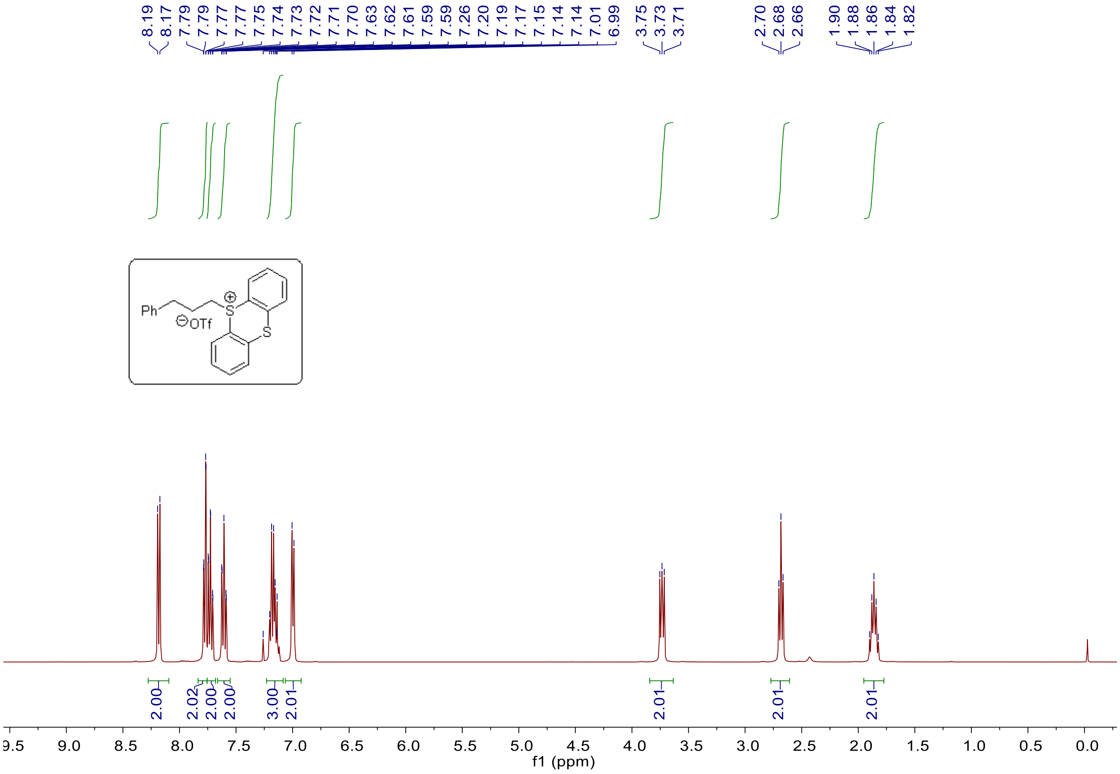

Supplementary Figure 18. ^{1}H NMR (400 MHz, $CDCl_3$) of **1b**

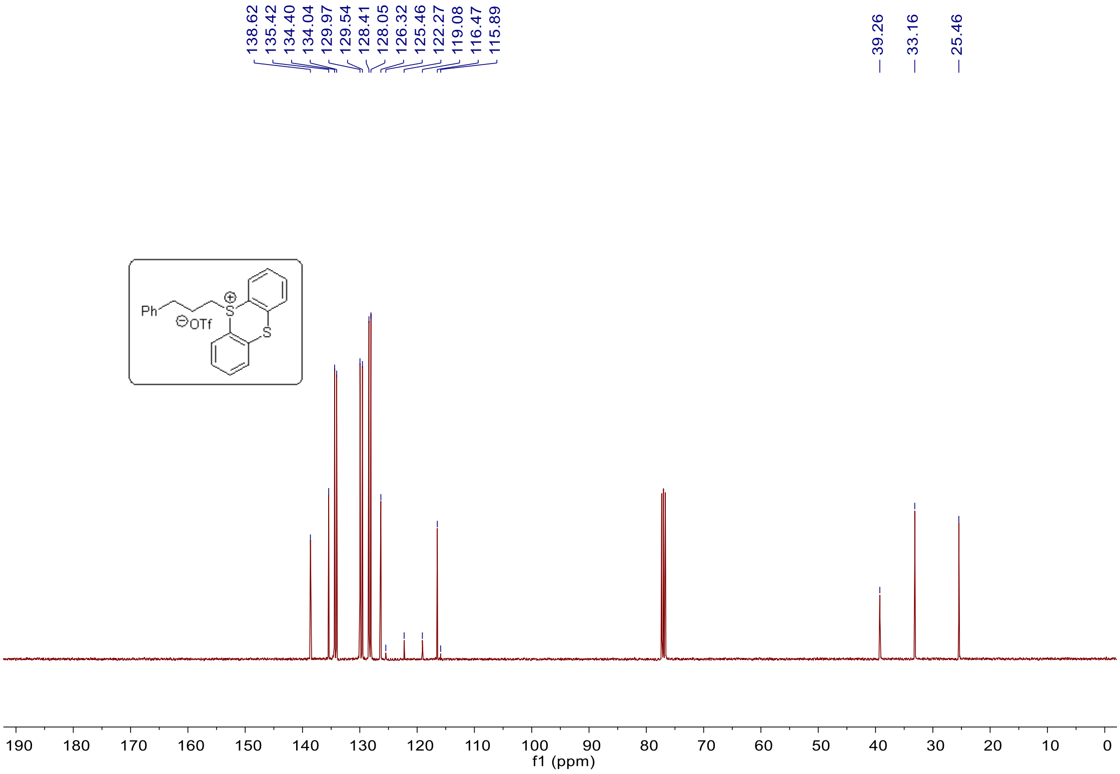

Supplementary Figure 19. ^{13}C NMR (101 MHz, $CDCl_3$) of **1b**

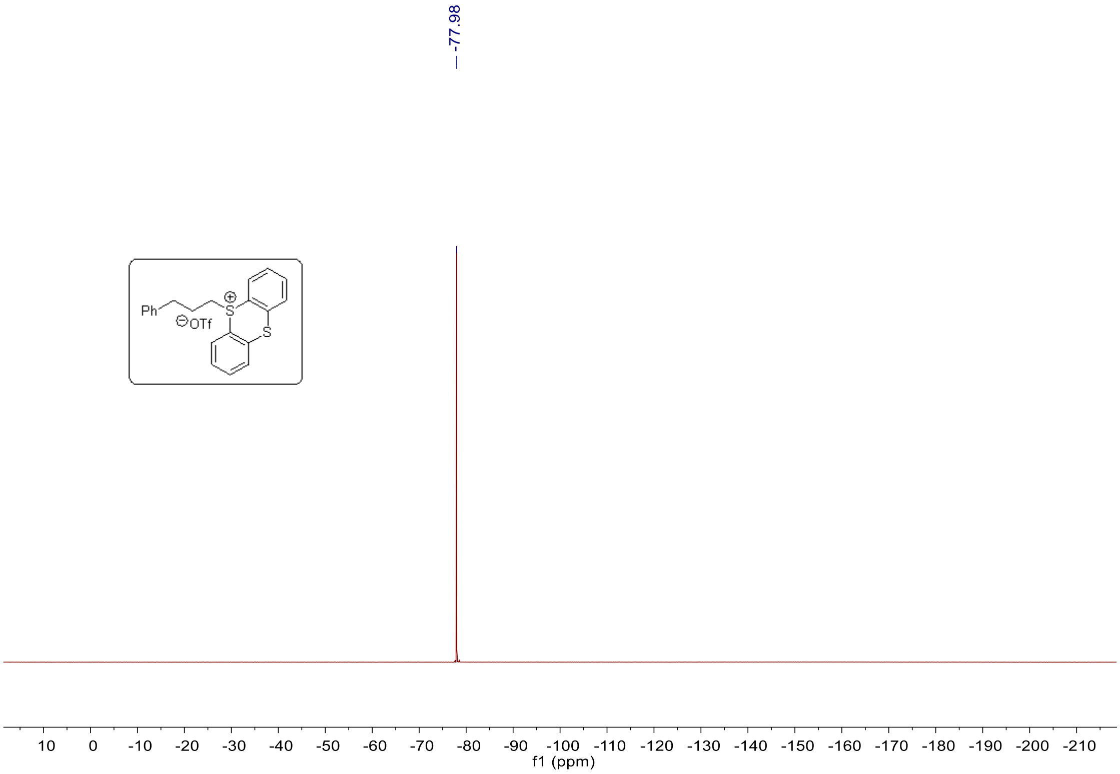

Supplementary Figure 20. ^{19}F NMR (376 MHz, $CDCl_3$) of **1b**

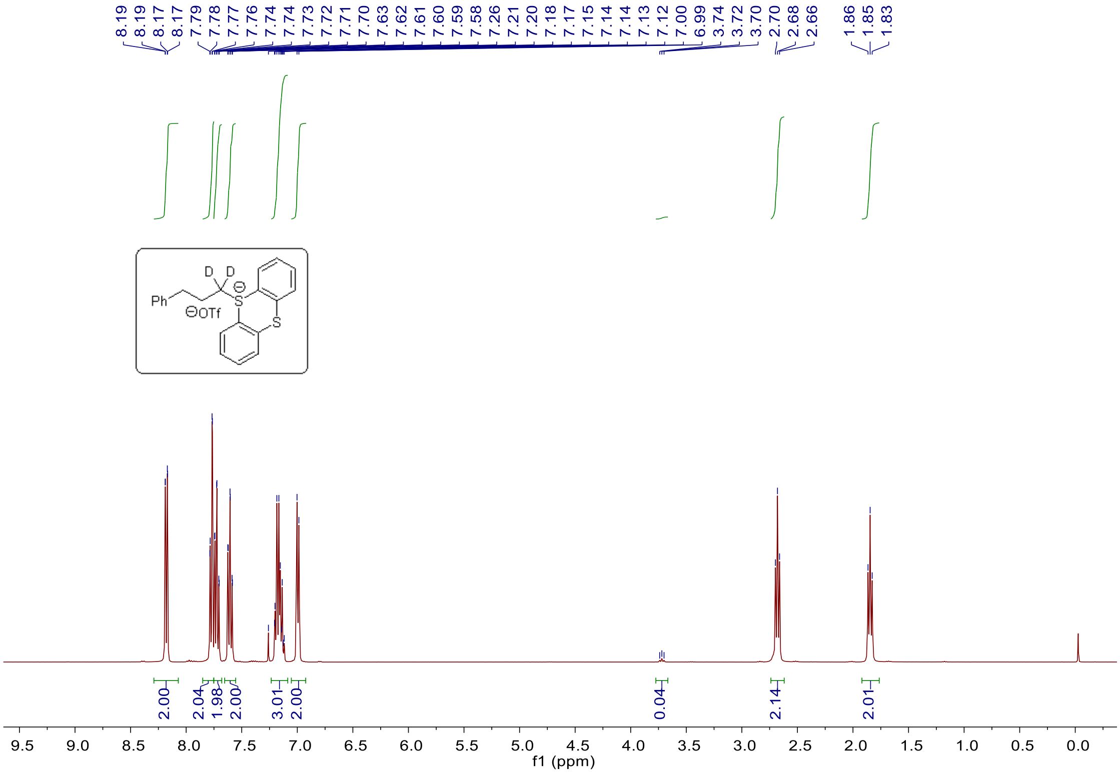

Supplementary Figure 21. ^{1}H NMR (400 MHz, $CDCl_3$) of **2b**

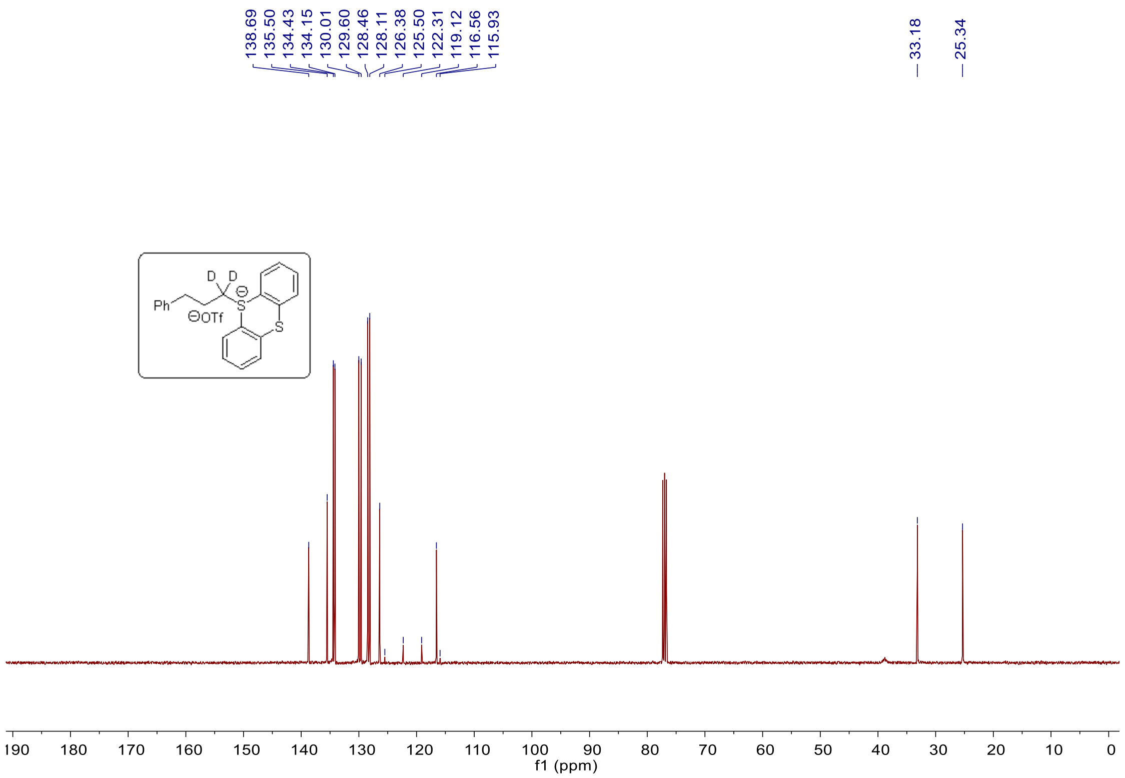

Supplementary Figure 22. ^{13}C NMR (101 MHz, $CDCl_3$) of **2b**

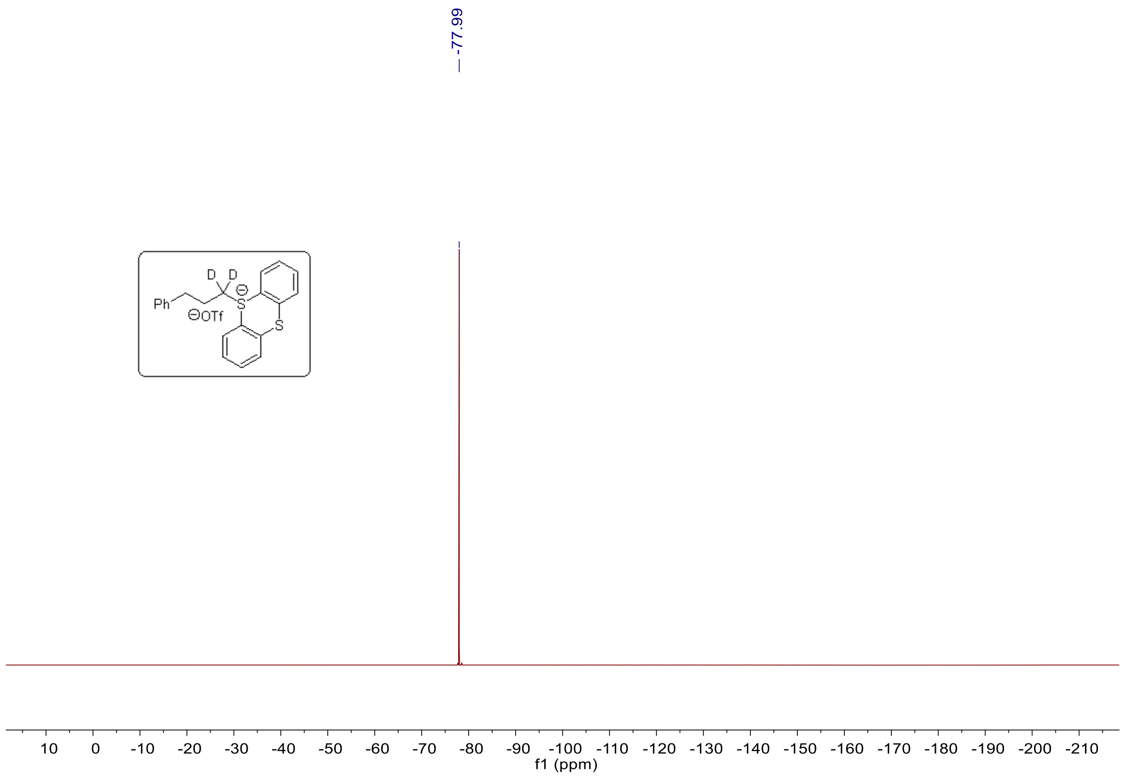

Supplementary Figure 23. ^{19}F NMR (376 MHz, $CDCl_3$) of **2b**

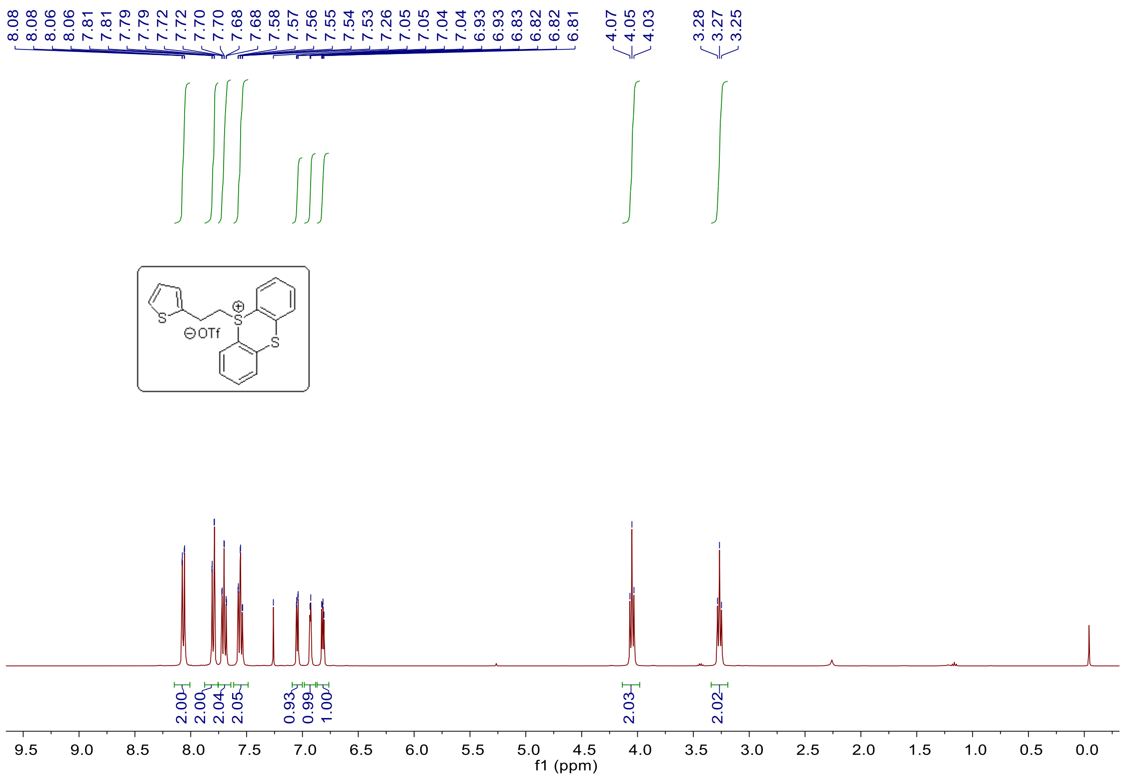

Supplementary Figure 24. ^{1}H NMR (400 MHz, $CDCl_3$) of **1c**

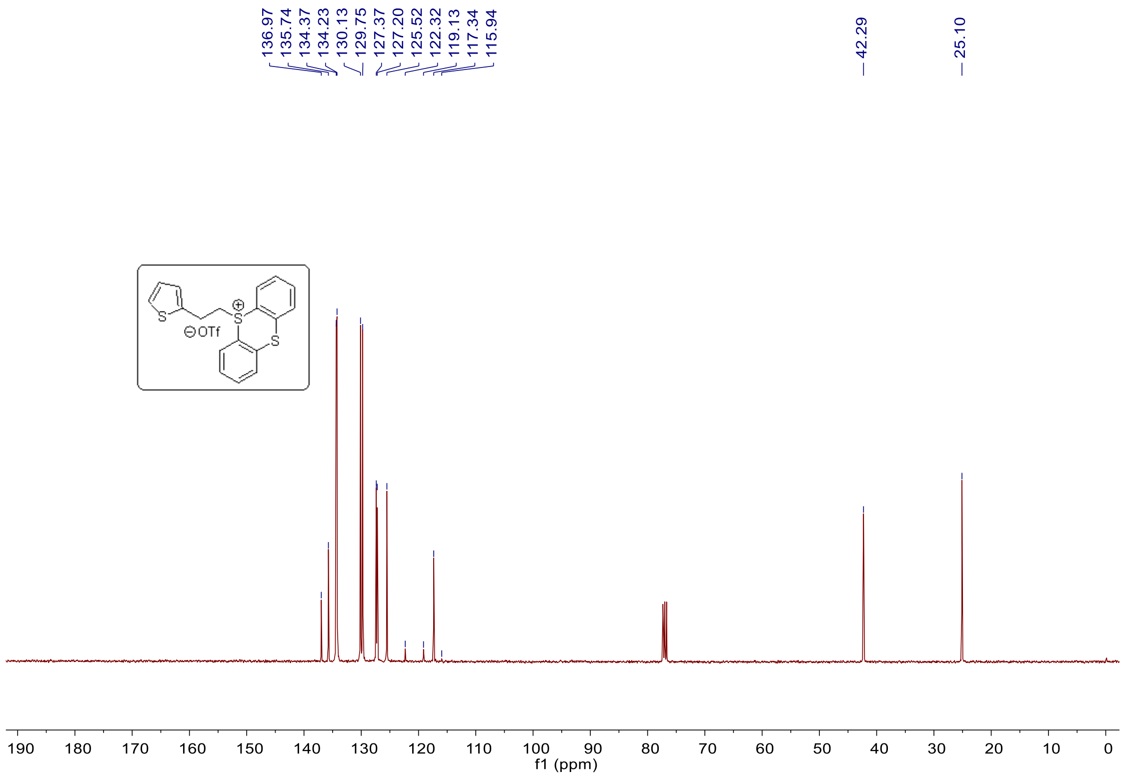

Supplementary Figure 25. ^{13}C NMR (101 MHz, $CDCl_3$) of **1c**

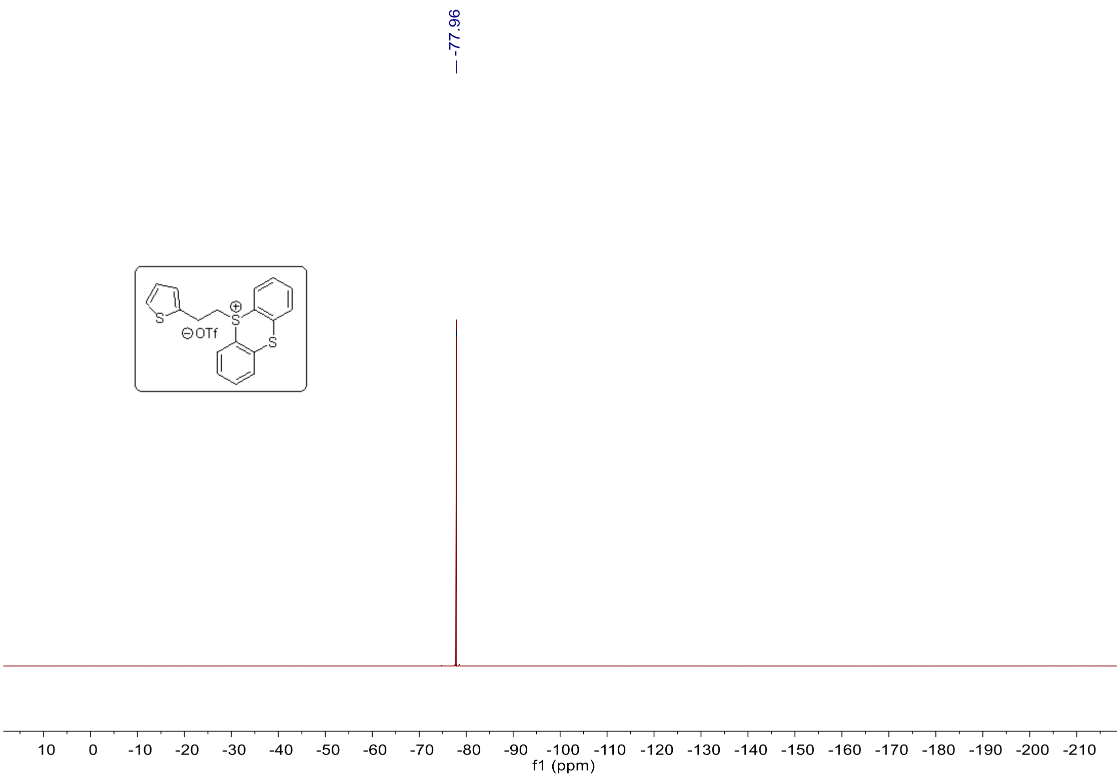

Supplementary Figure 26. ^{19}F NMR (376 MHz, $CDCl_3$) of **1c**

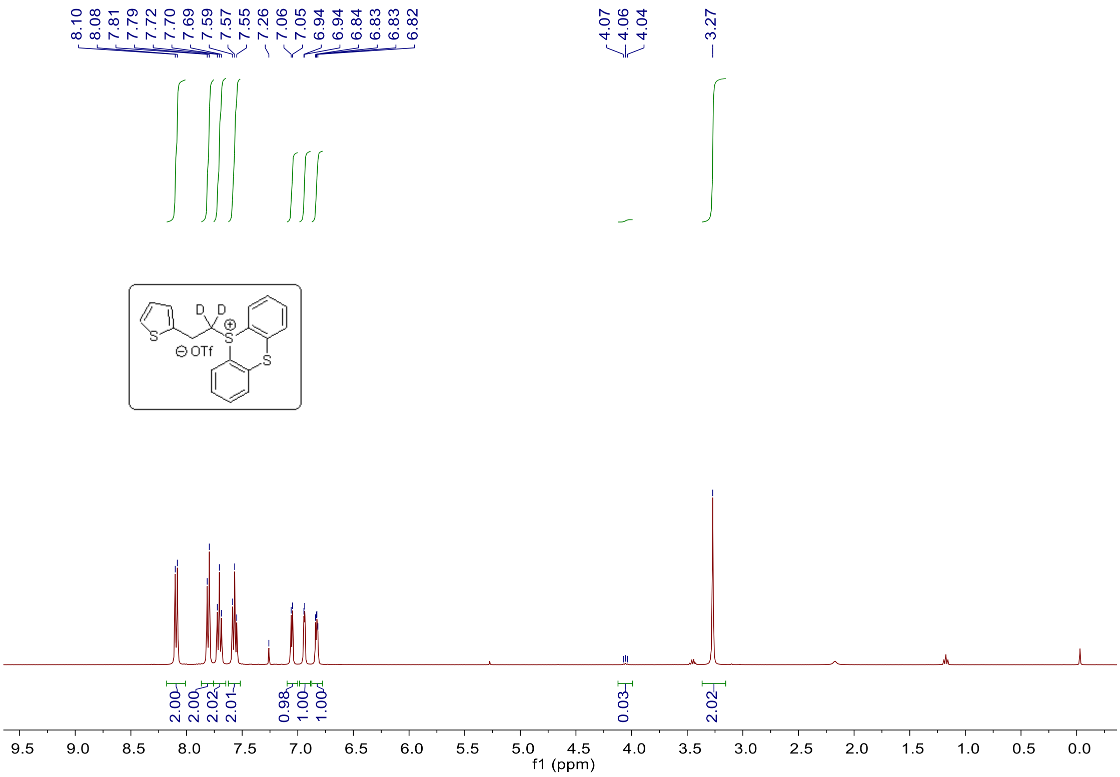

Supplementary Figure 27. ^{1}H NMR (400 MHz, $CDCl_3$) of **2c**

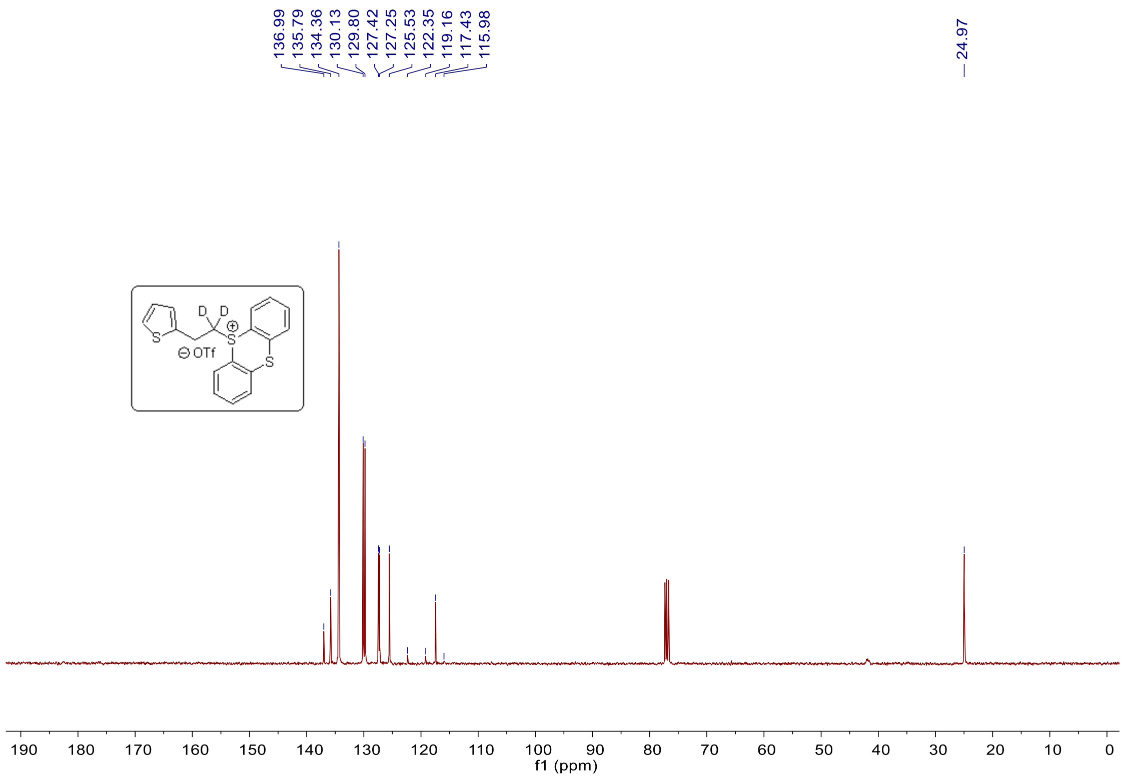

Supplementary Figure 28. ^{13}C NMR (101 MHz, $CDCl_3$) of **2c**

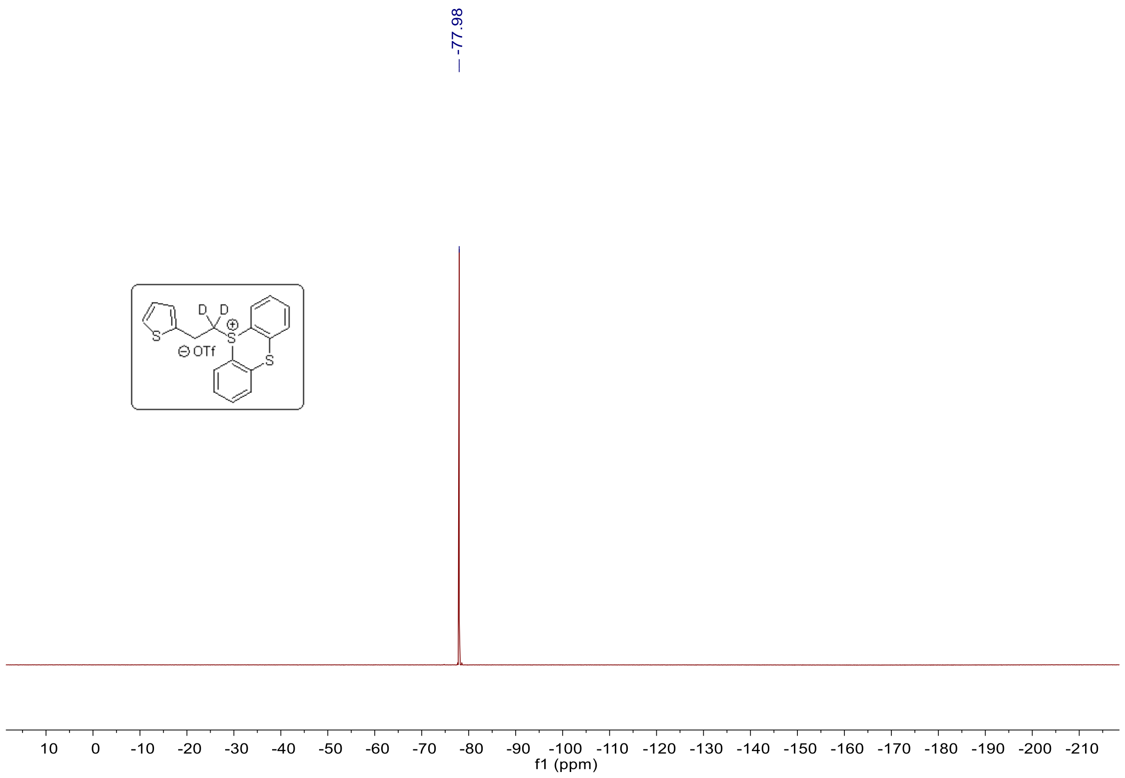

Supplementary Figure 29. ^{19}F NMR (376 MHz, $CDCl_3$) of **2c**

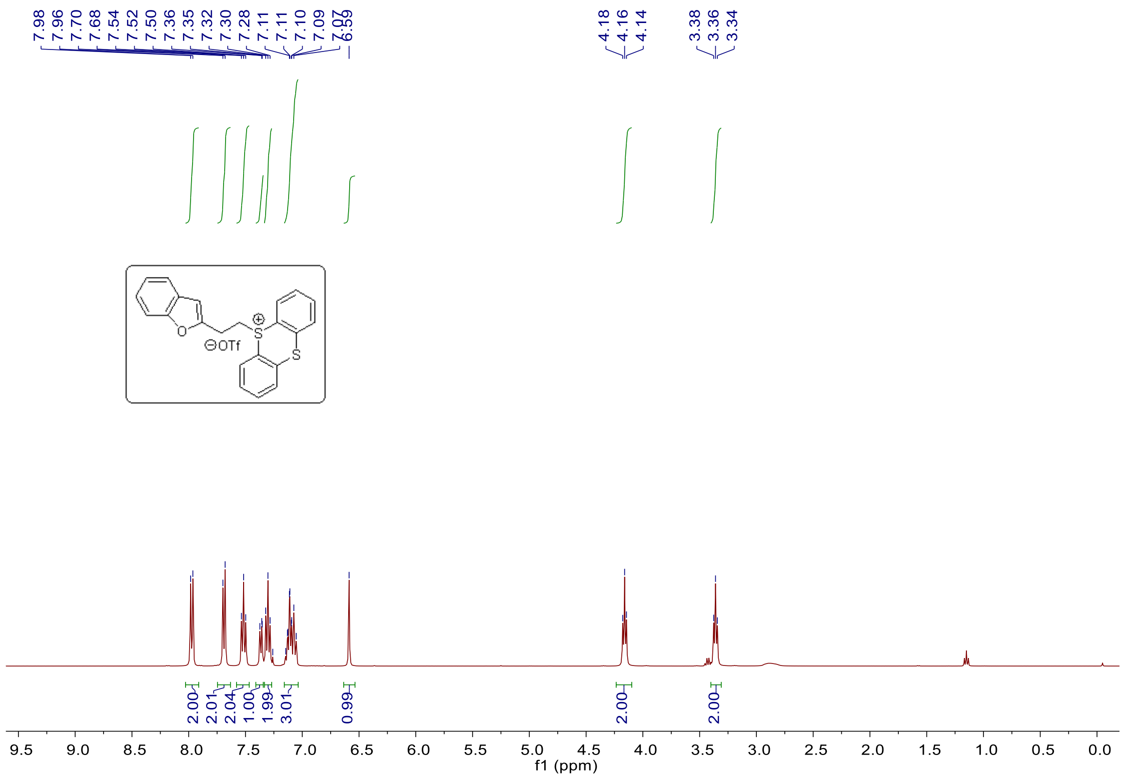

Supplementary Figure 30. ^{1}H NMR (400 MHz, $CDCl_3$) of **1d**

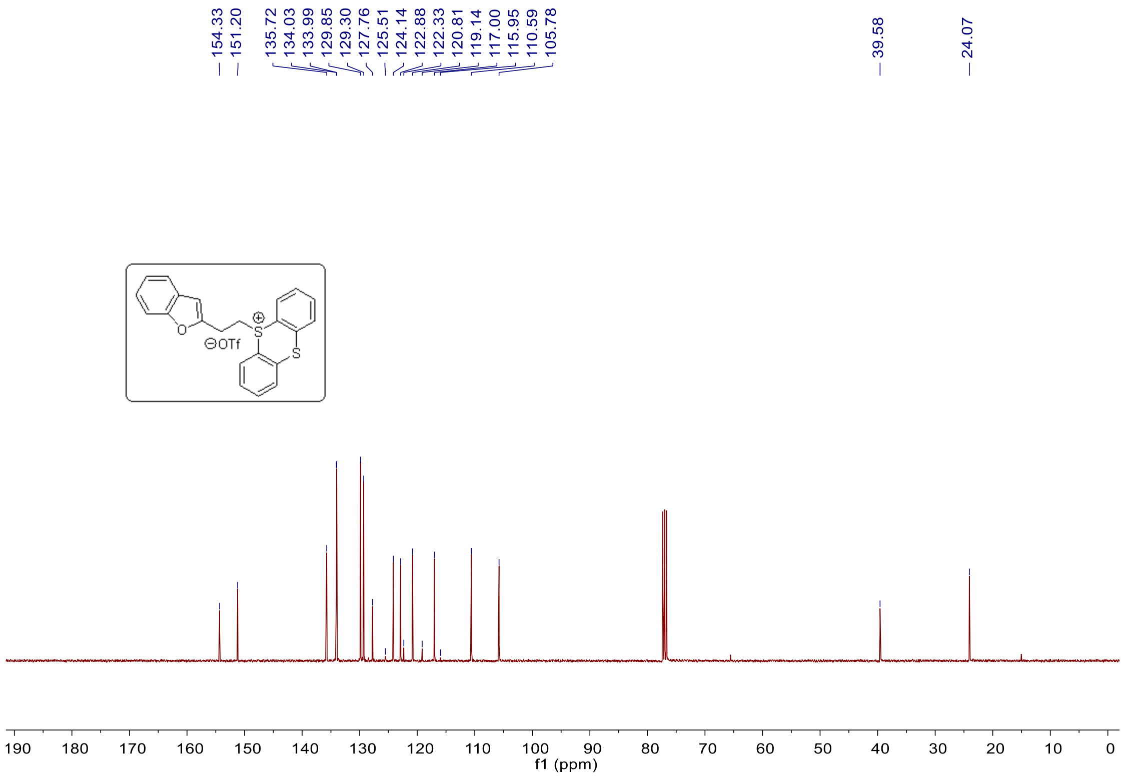

Supplementary Figure 31. ^{13}C NMR (101 MHz, $CDCl_3$) of **1d**

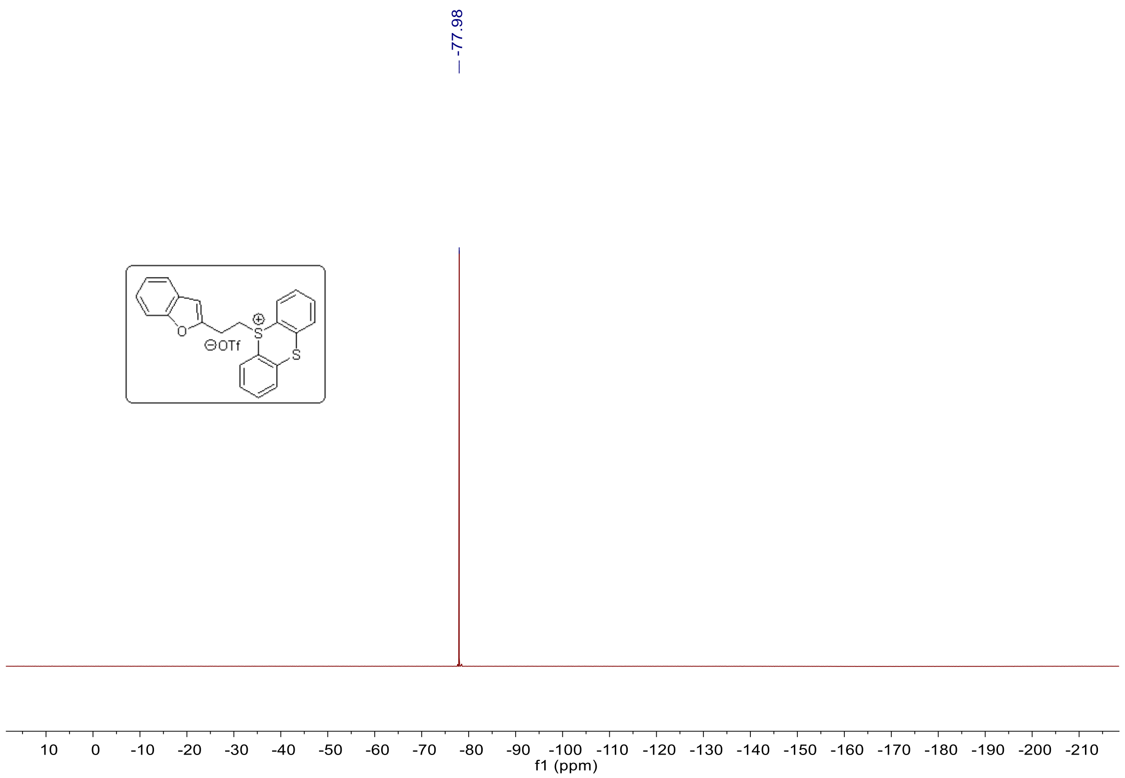

Supplementary Figure 32. ^{19}F NMR (376 MHz, $CDCl_3$) of **1d**

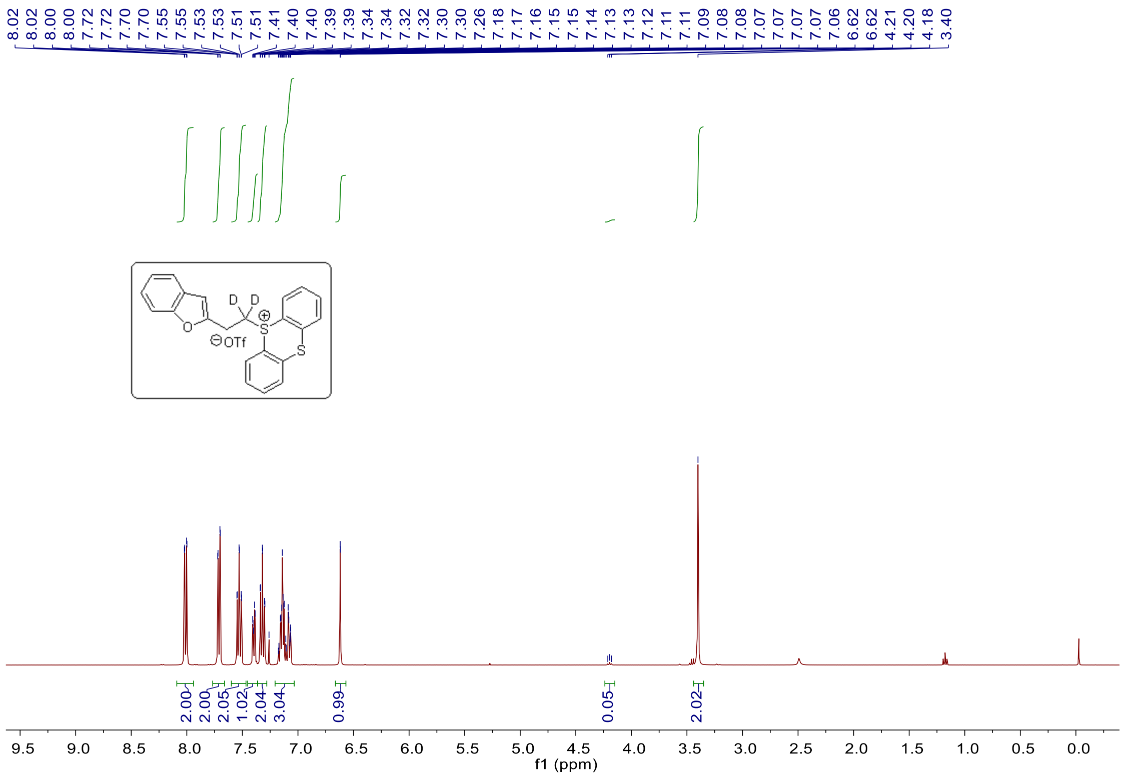

Supplementary Figure 33. ^{1}H NMR (400 MHz, $CDCl_3$) of **2d**

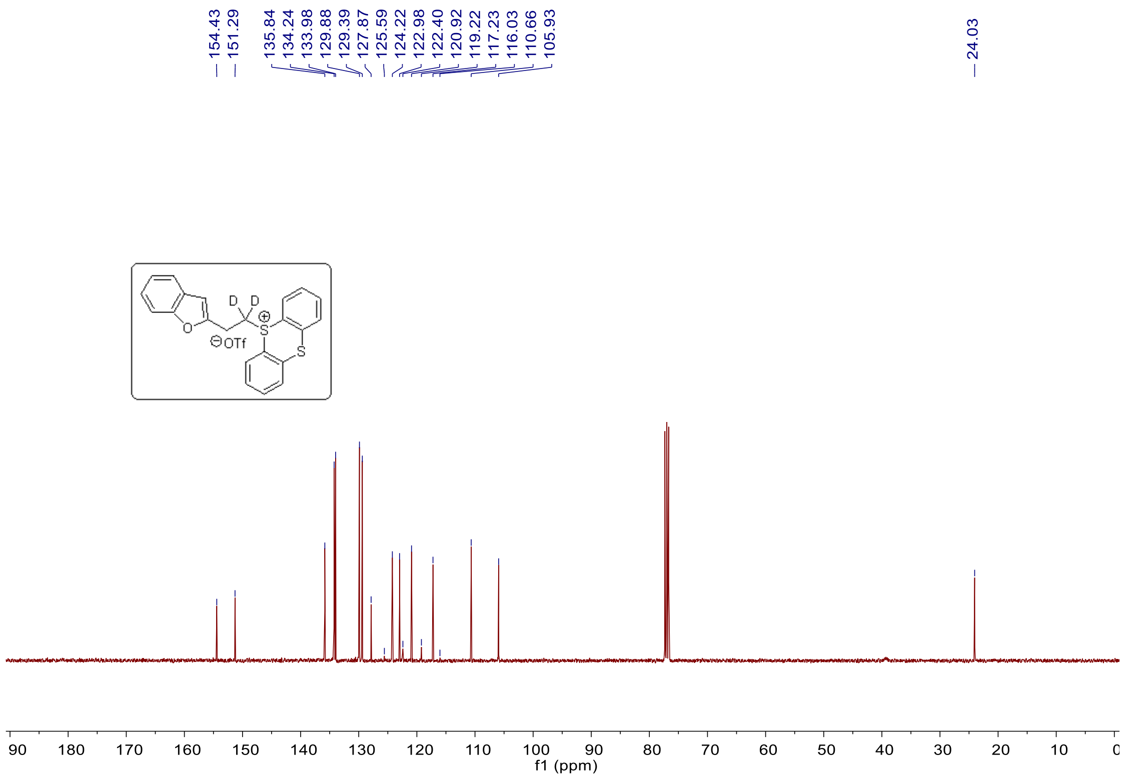

Supplementary Figure 34. ^{13}C NMR (101 MHz, $CDCl_3$) of **2d**

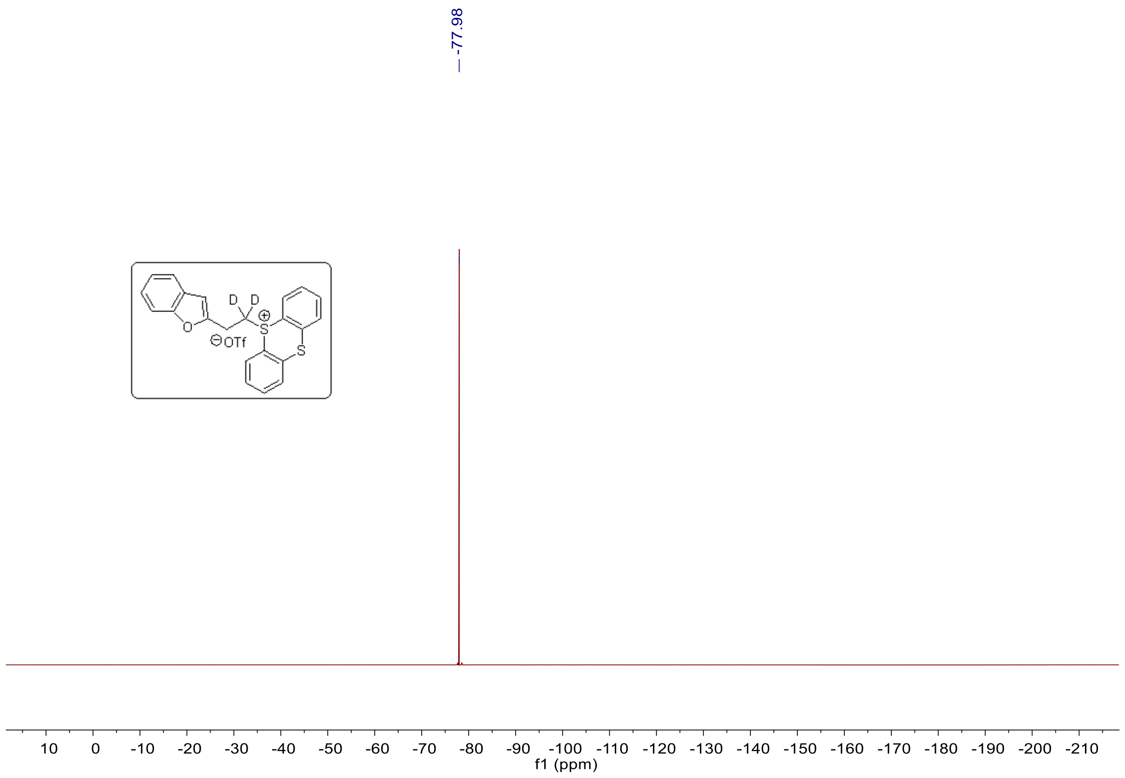

Supplementary Figure 35. ^{19}F NMR (376 MHz, $CDCl_3$) of **2d**

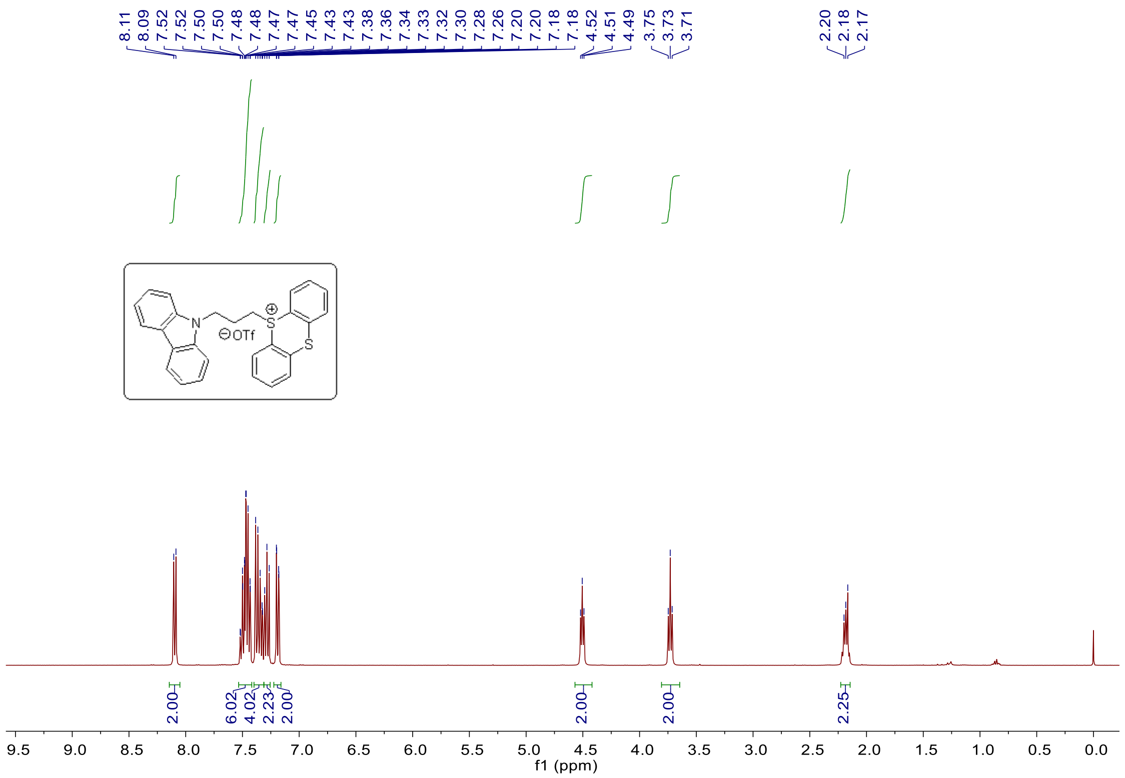

Supplementary Figure 36. ^{1}H NMR (400 MHz, $CDCl_3$) of **1e**

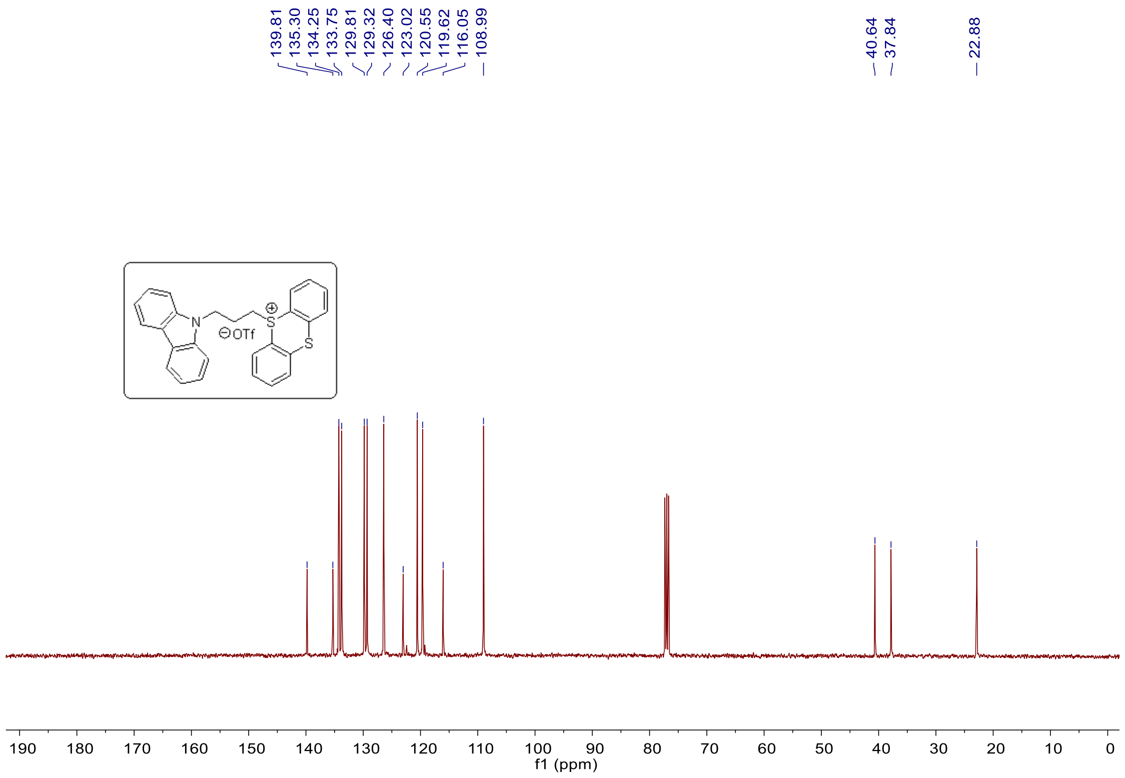

Supplementary Figure 37. ^{13}C NMR (101 MHz, $CDCl_3$) of **1e**

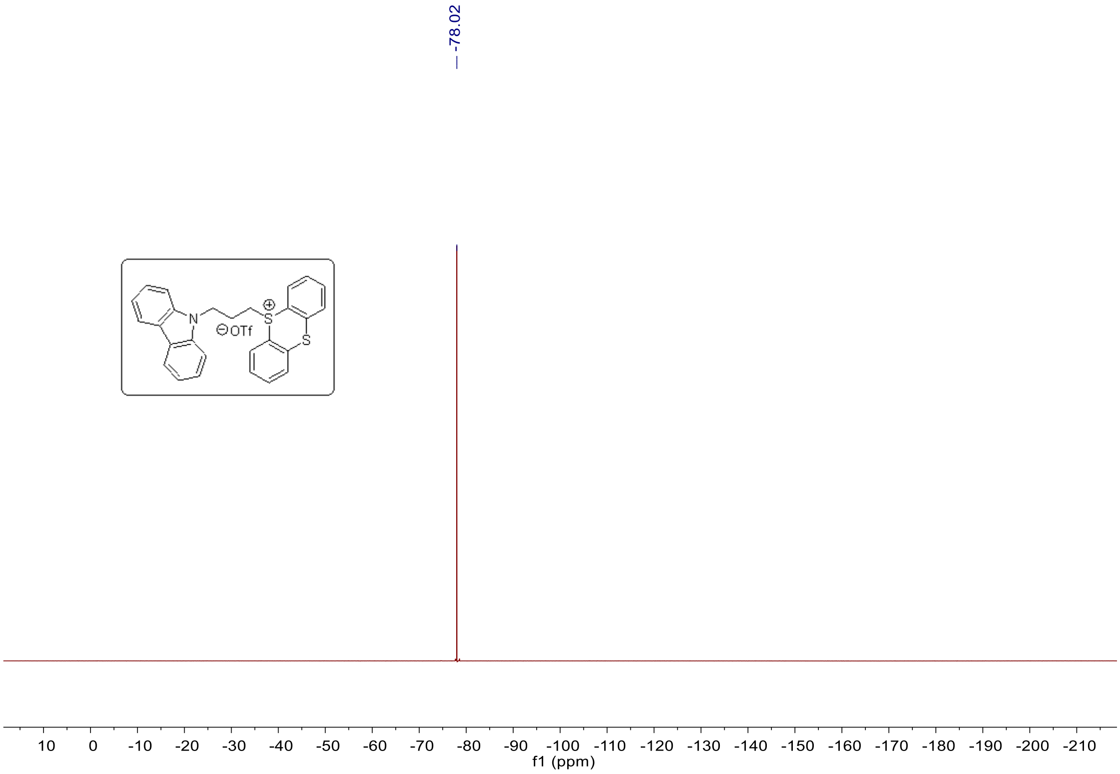

Supplementary Figure 38. ^{19}F NMR (376 MHz, $CDCl_3$) of **1e**

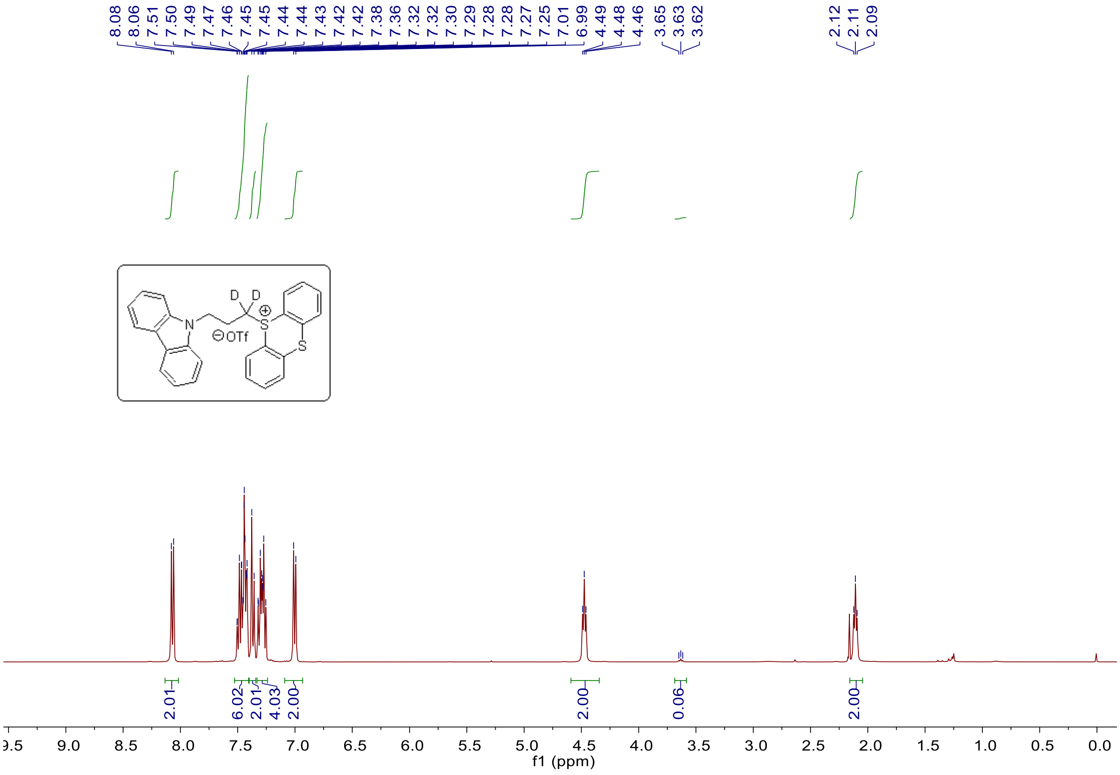

Supplementary Figure 39. ^{1}H NMR (400 MHz, $CDCl_3$) of **2e**

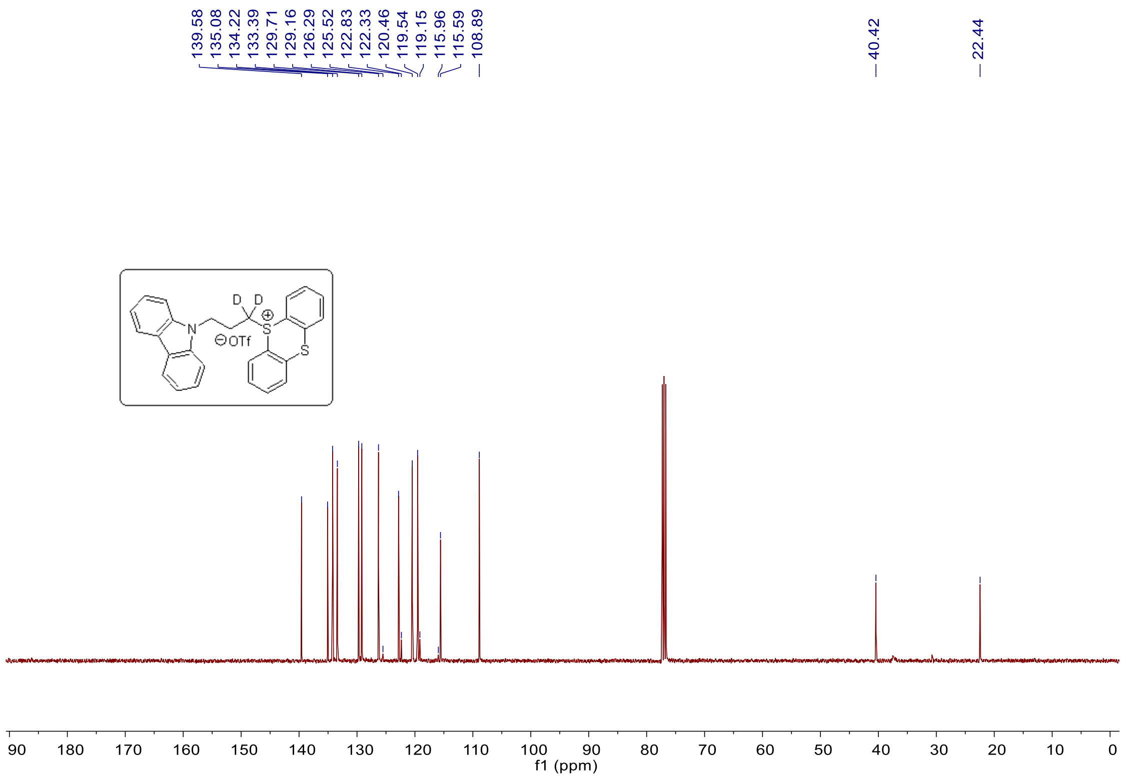

Supplementary Figure 40. ^{13}C NMR (101 MHz, $CDCl_3$) of **2e**

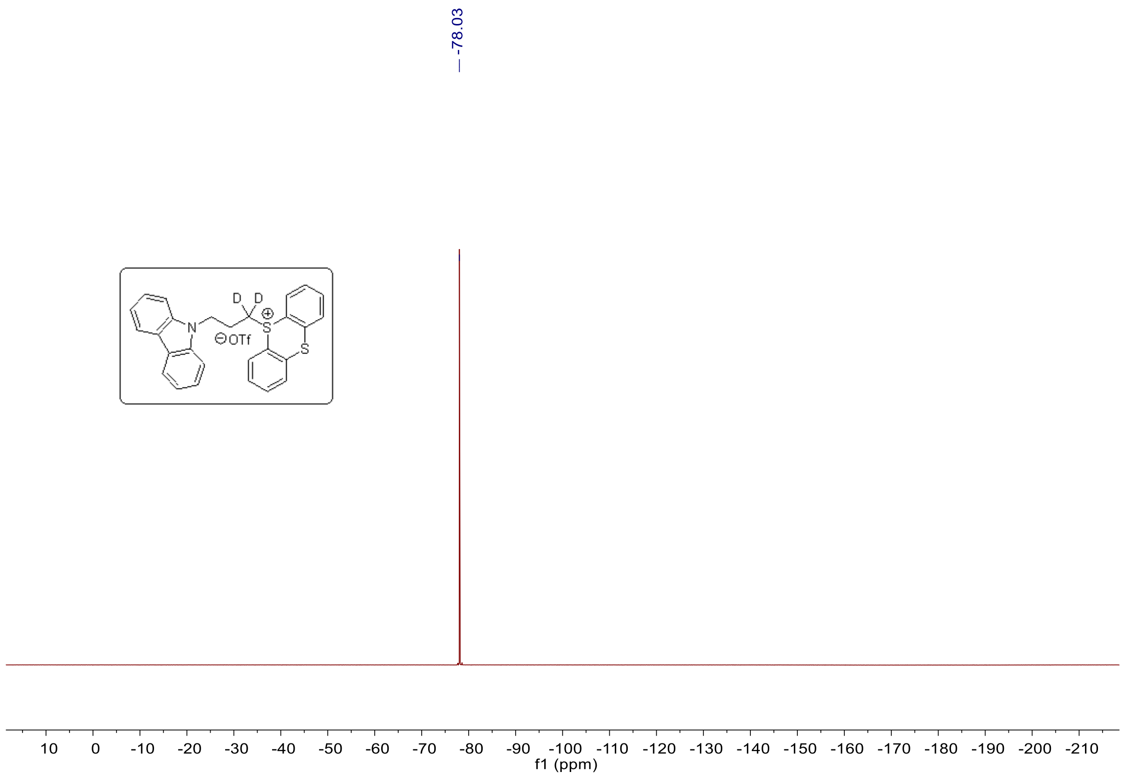

Supplementary Figure 41. ^{19}F NMR (376 MHz, $CDCl_3$) of **2e**

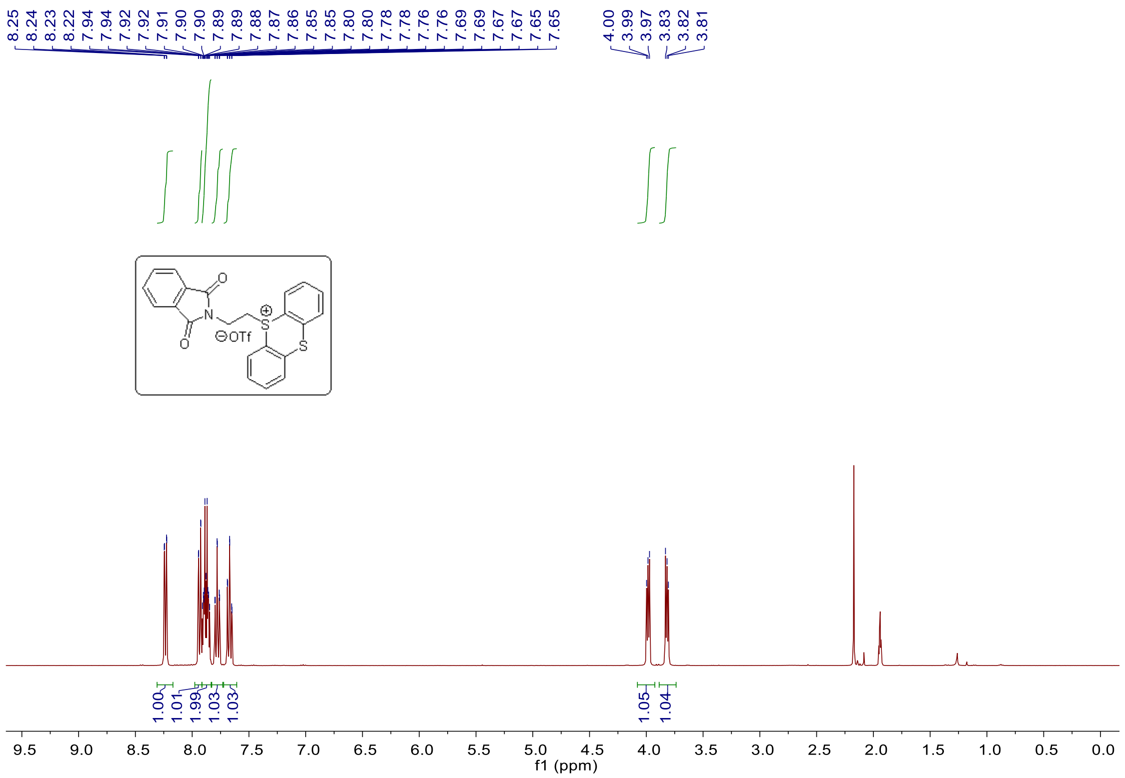

Supplementary Figure 42. ^{1}H NMR (400 MHz, CD_3CN) of **1f**

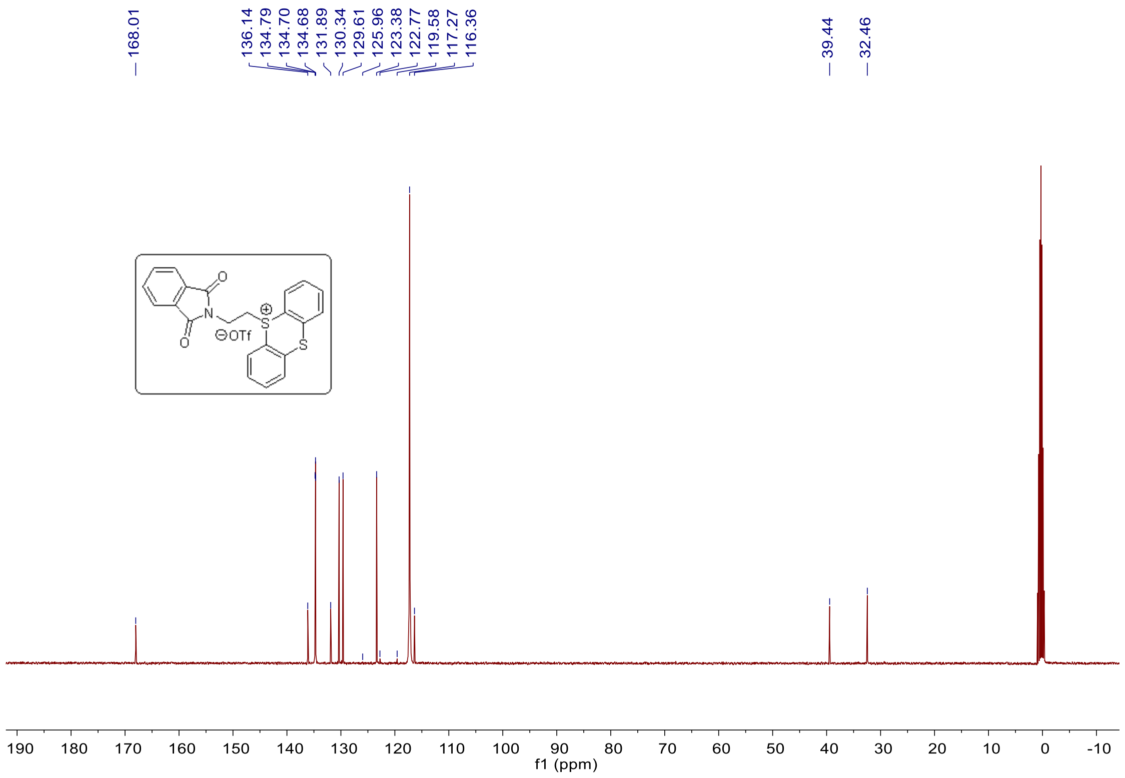

Supplementary Figure 43. ^{13}C NMR (101 MHz, CD_3CN) of **1f**

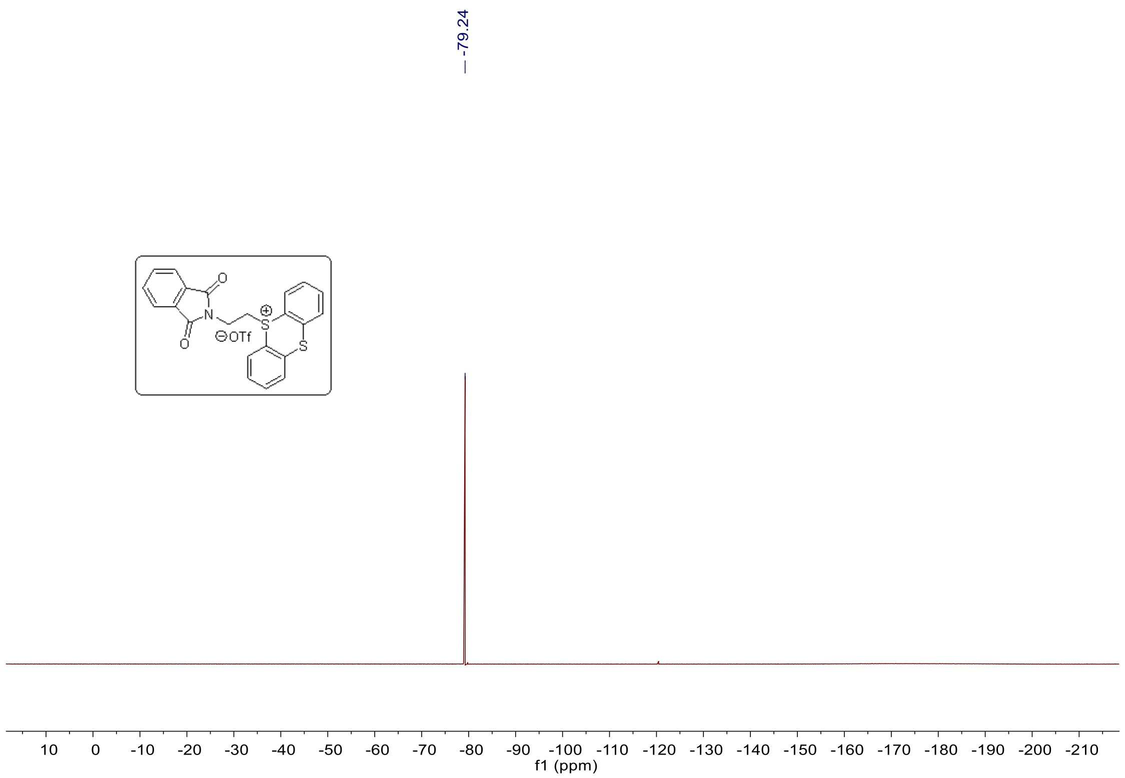

Supplementary Figure 44. ^{19}F NMR (376 MHz, CD_3CN) of **1f**

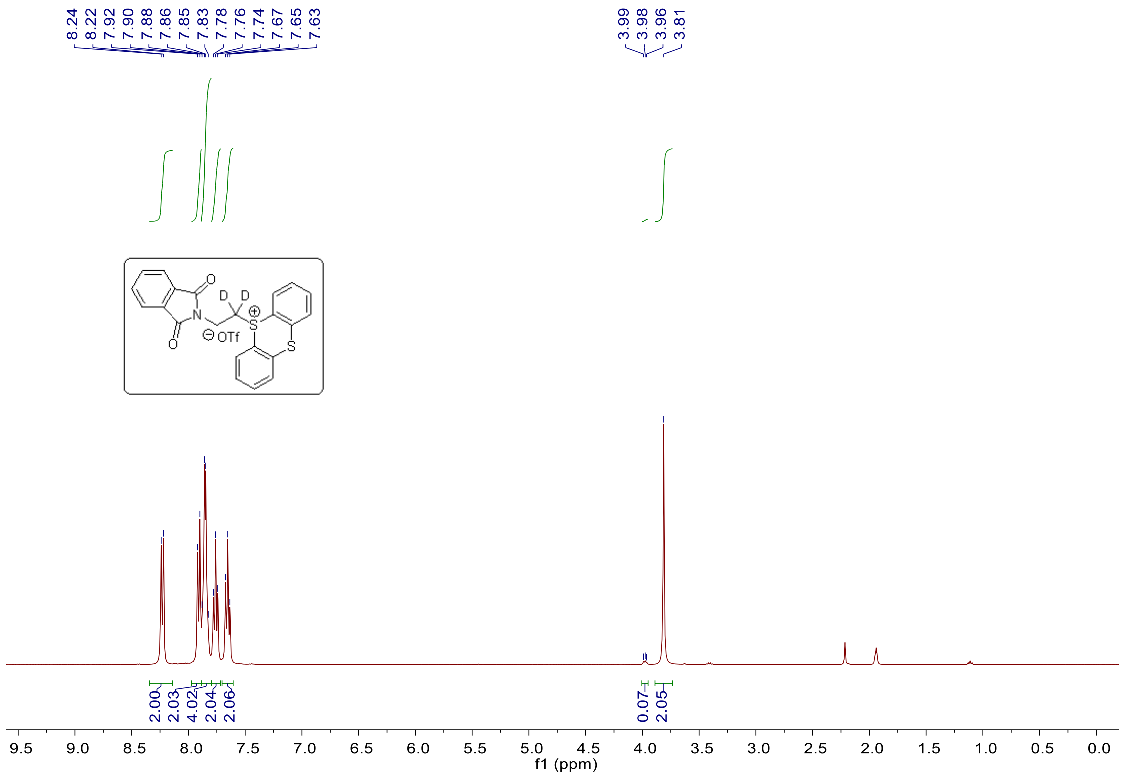

Supplementary Figure 45. ^{1}H NMR (400 MHz, CD_3CN) of **2f**

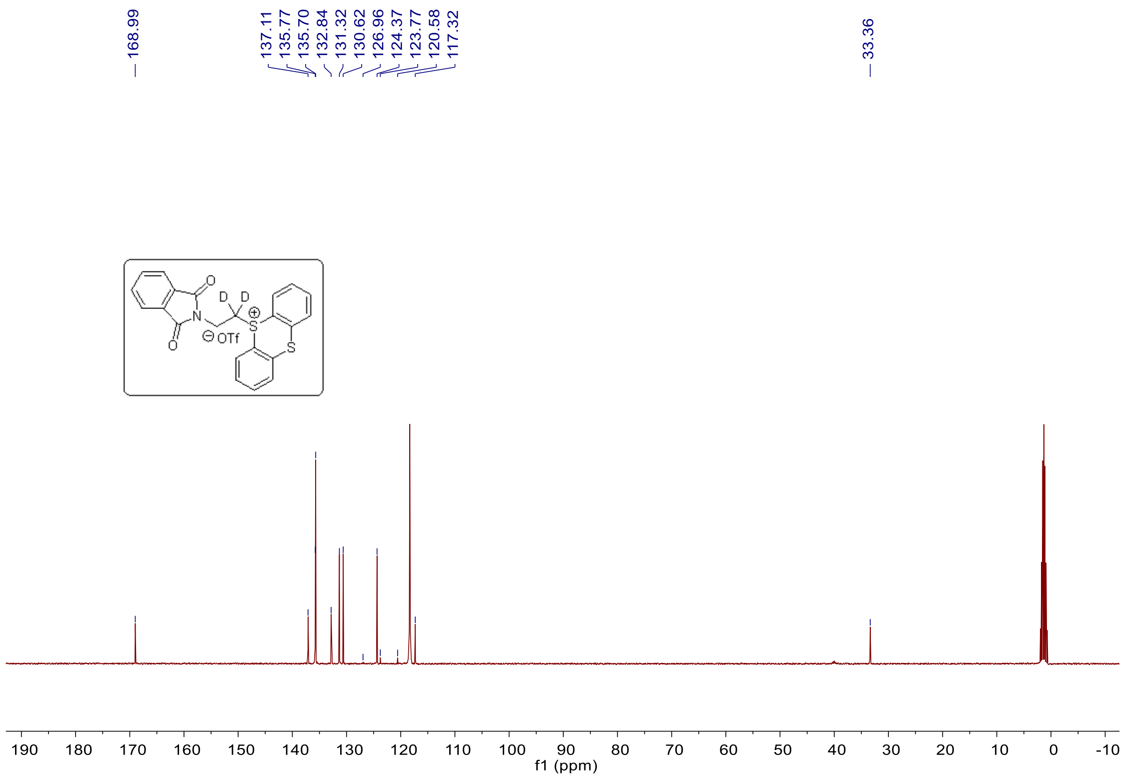

Supplementary Figure 46. ^{13}C NMR (101 MHz, CD_3CN) of **2f**

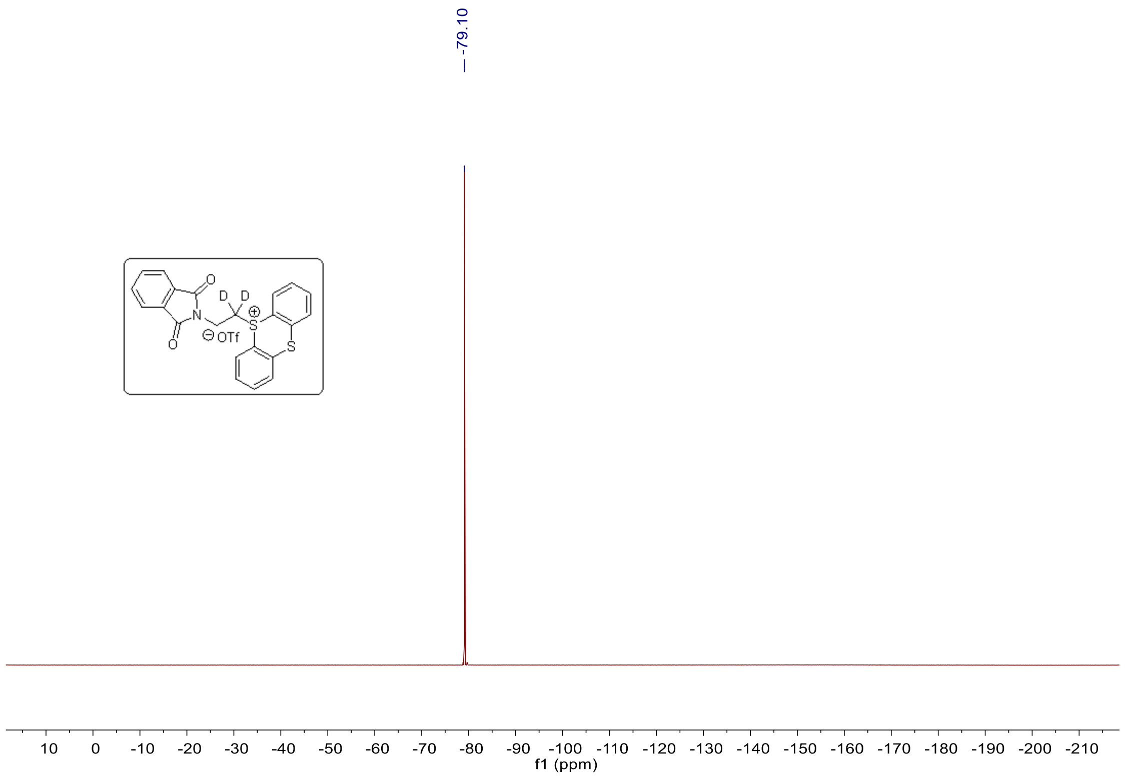

Supplementary Figure 47. ^{19}F NMR (376 MHz, CD_3CN) of **2f**

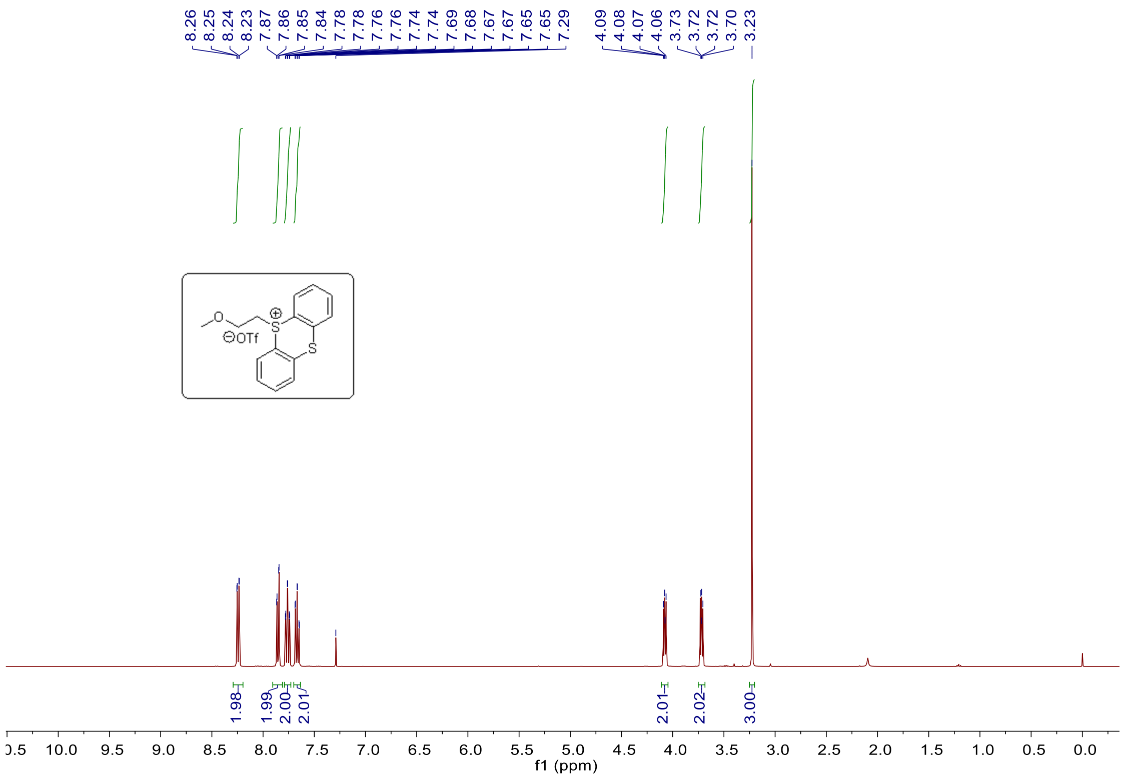

Supplementary Figure 48. ^{1}H NMR (400 MHz, $CDCl_3$) of **1g**

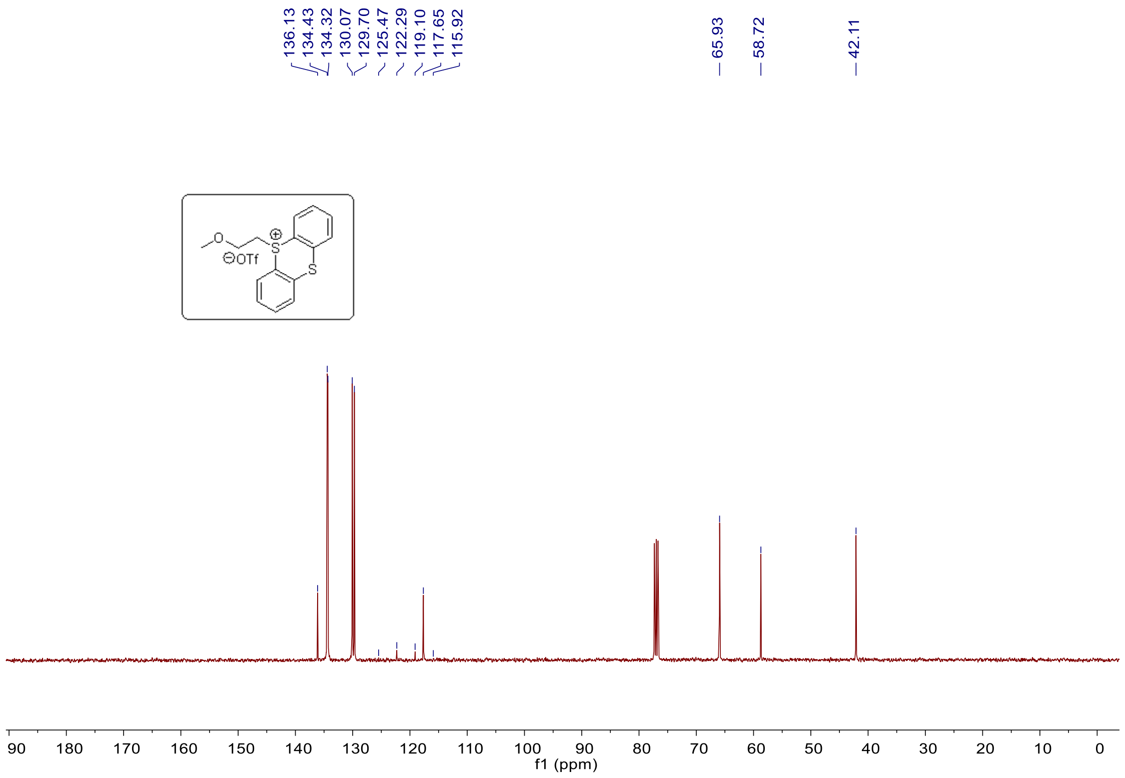

Supplementary Figure 49. ^{13}C NMR (101 MHz, $CDCl_3$) of **1g**

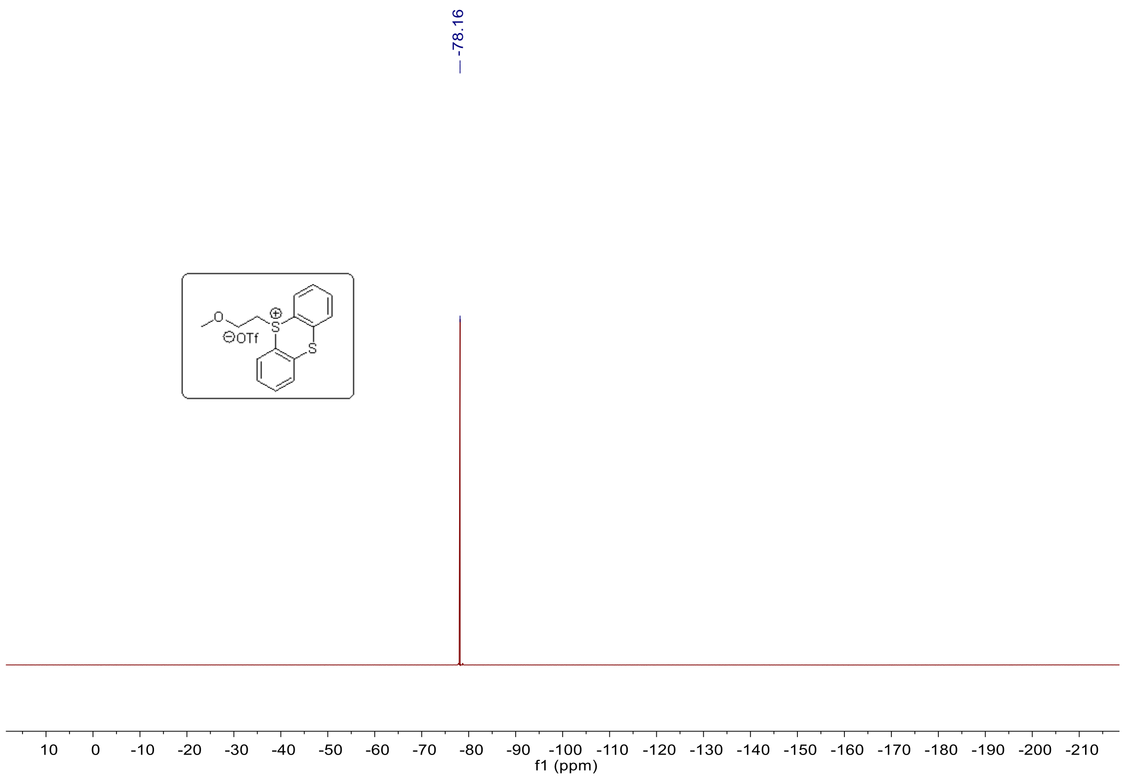

Supplementary Figure 50. ^{19}F NMR (376 MHz, $CDCl_3$) of **1g**

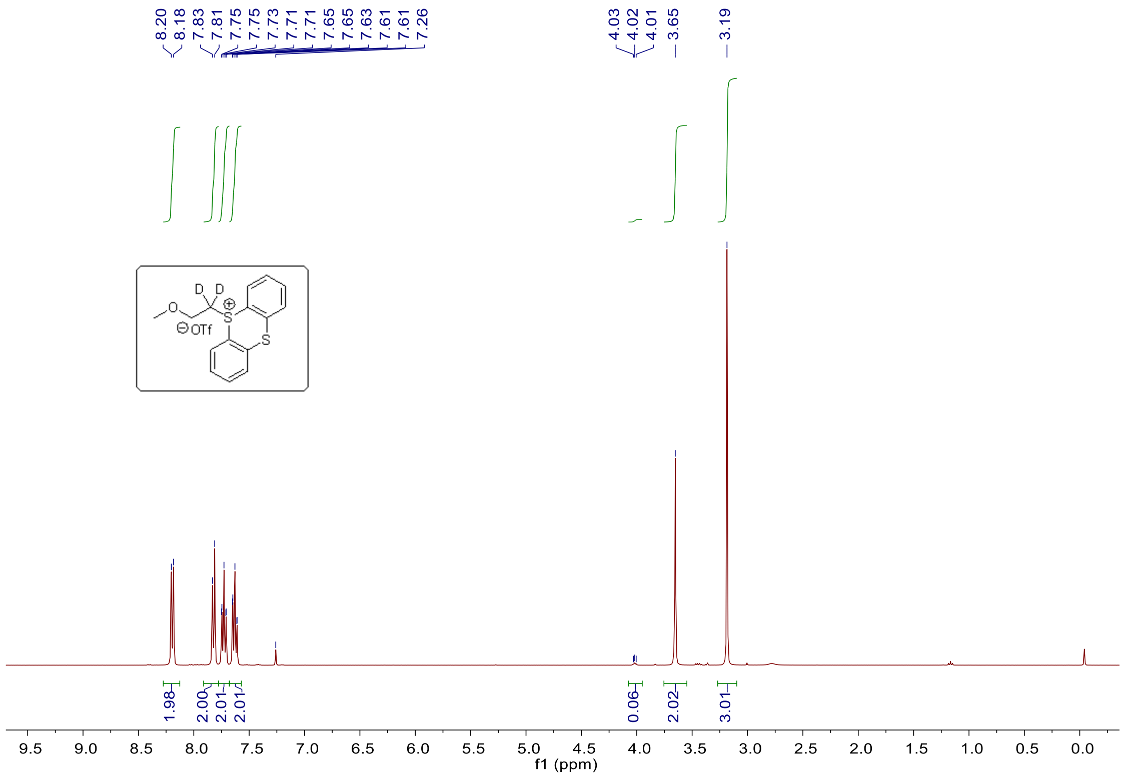

Supplementary Figure 51. ^{1}H NMR (400 MHz, $CDCl_3$) of **2g**

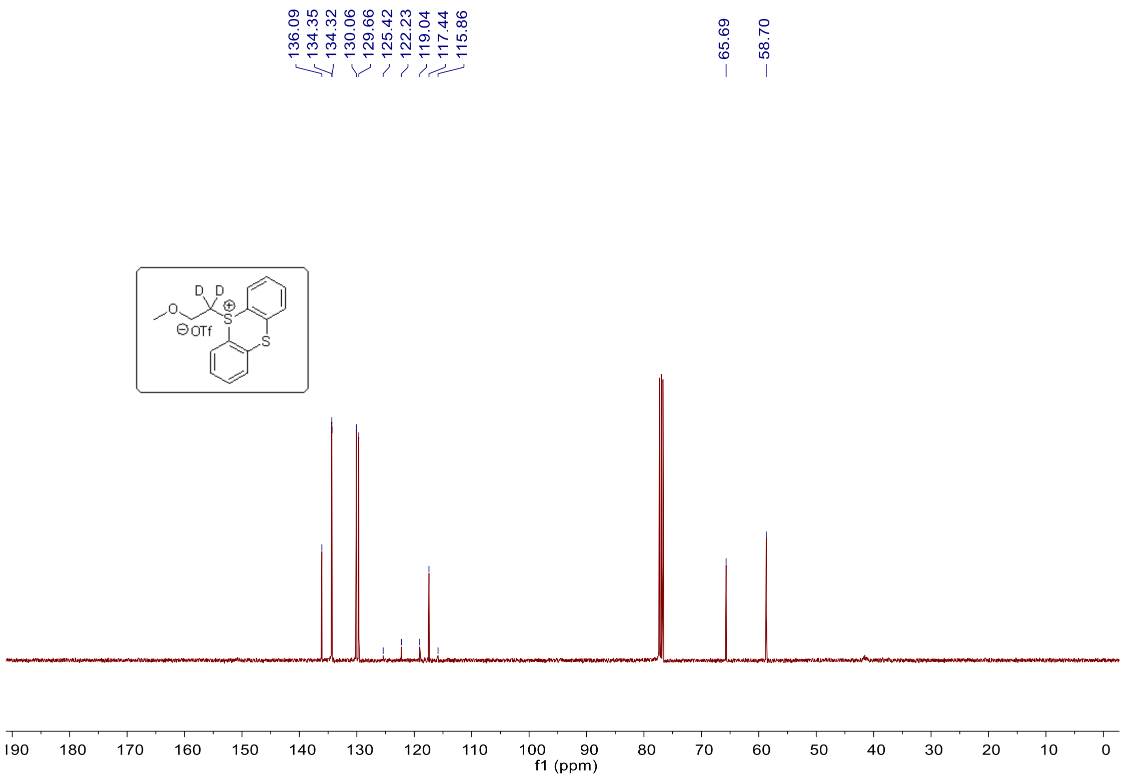

Supplementary Figure 52. ^{13}C NMR (101 MHz, $CDCl_3$) of **2g**

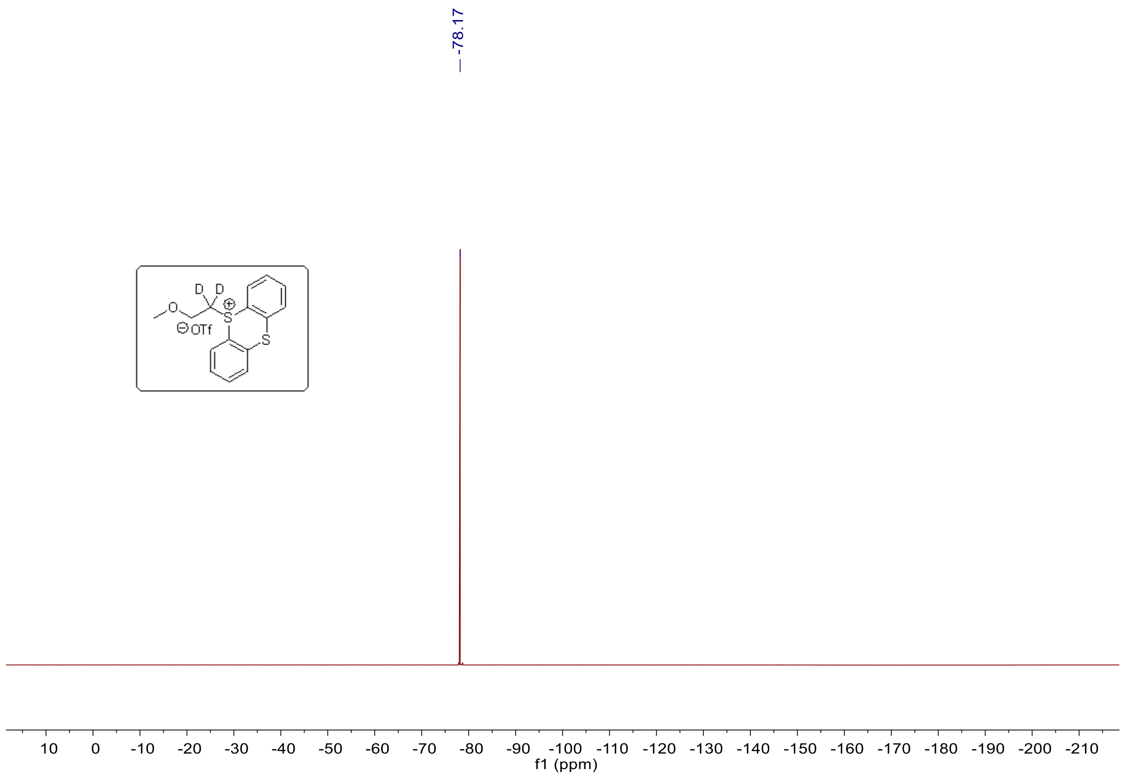

Supplementary Figure 53. ^{19}F NMR (376 MHz, $CDCl_3$) of **2g**

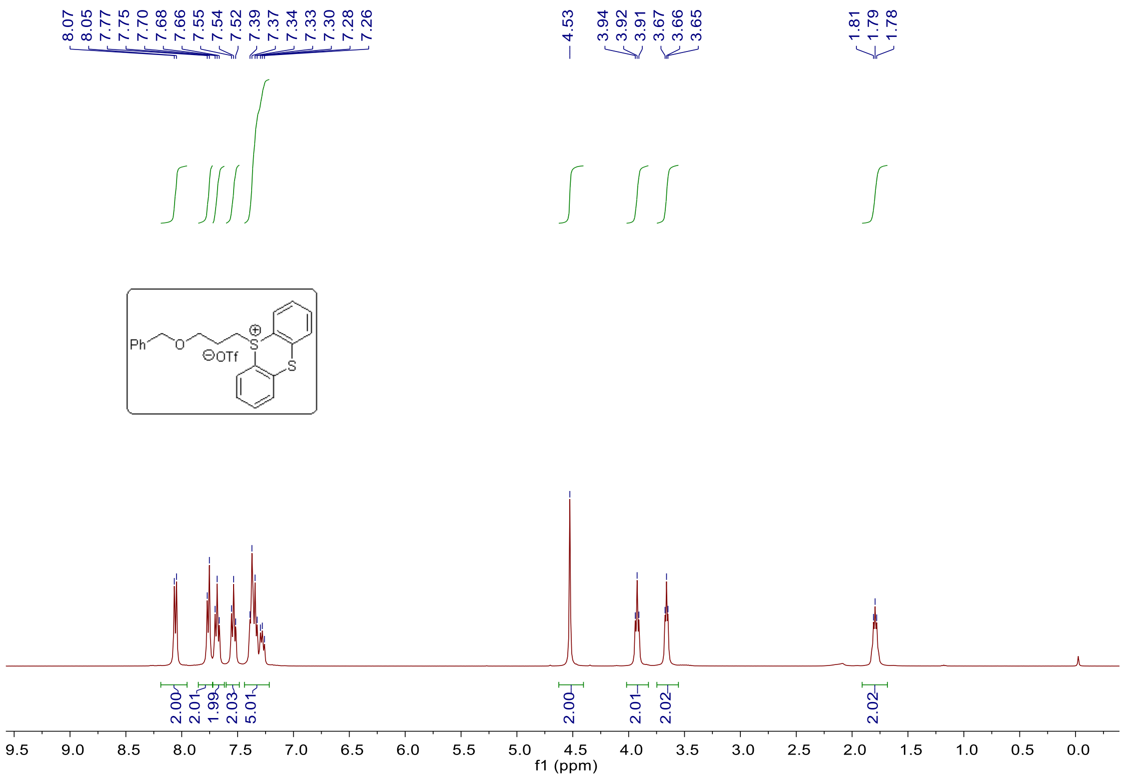

Supplementary Figure 54. ^{1}H NMR (400 MHz, $CDCl_3$) of **1h**

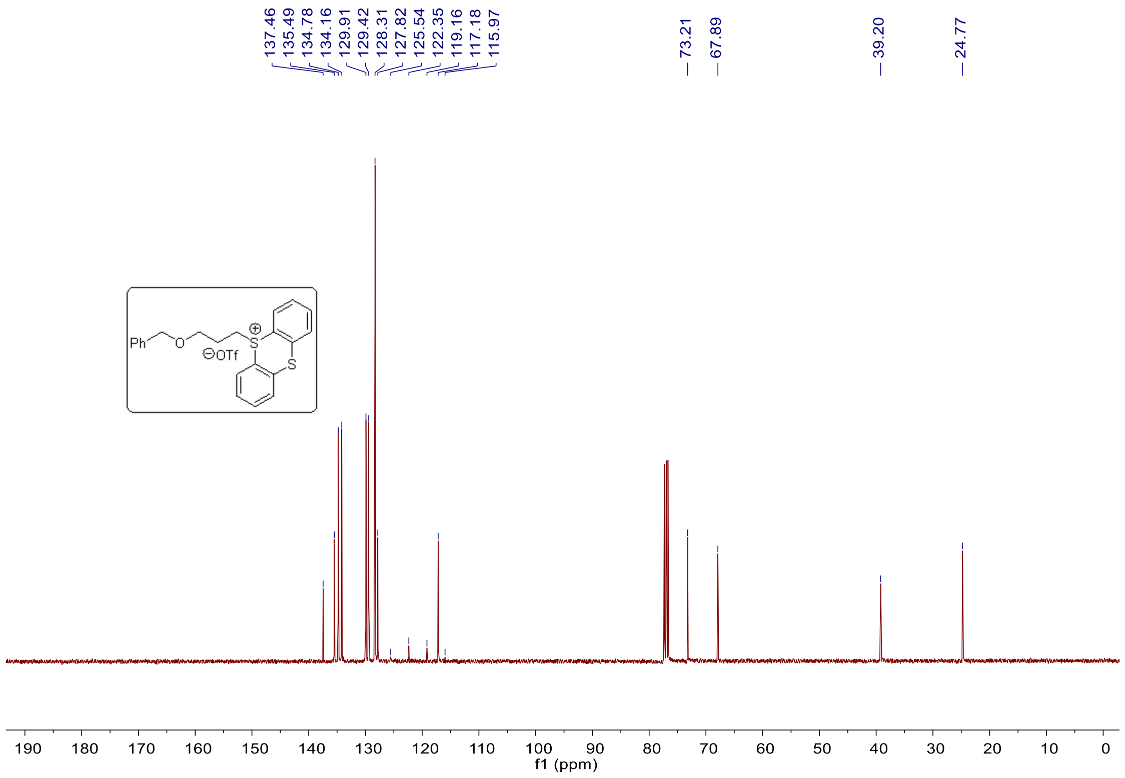

Supplementary Figure 55. ^{13}C NMR (101 MHz, $CDCl_3$) of **1h**

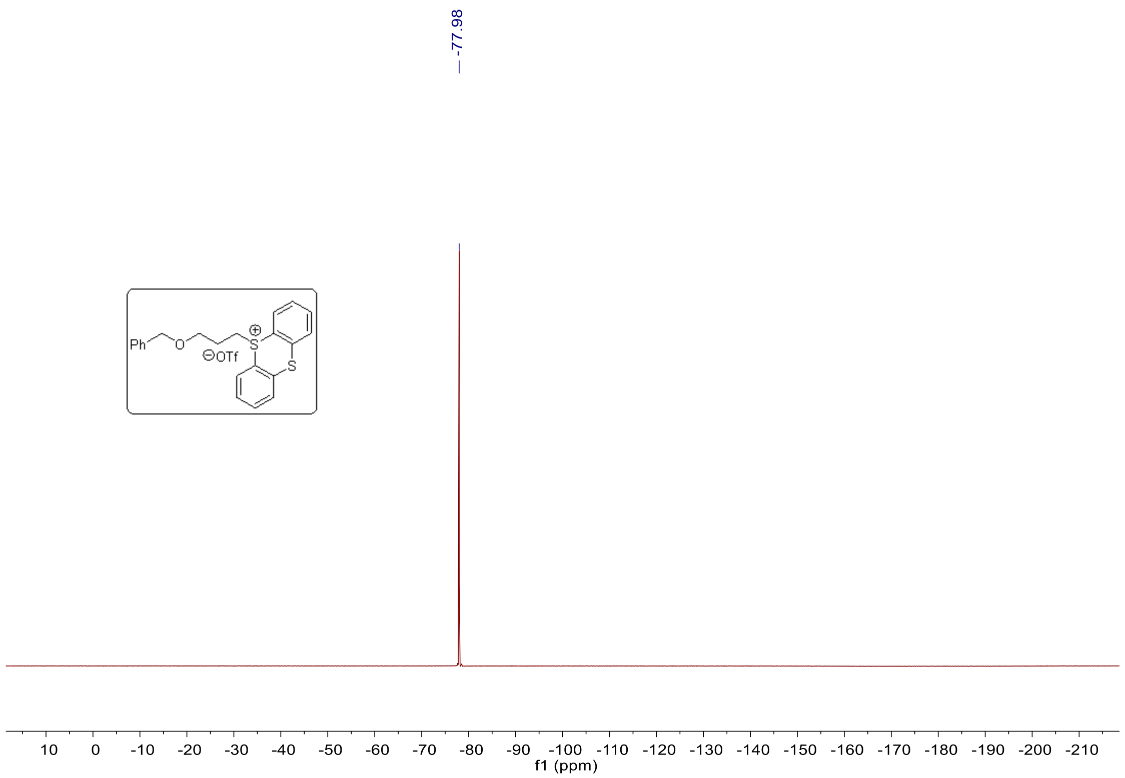

Supplementary Figure 56. ^{19}F NMR (376 MHz, $CDCl_3$) of **1h**

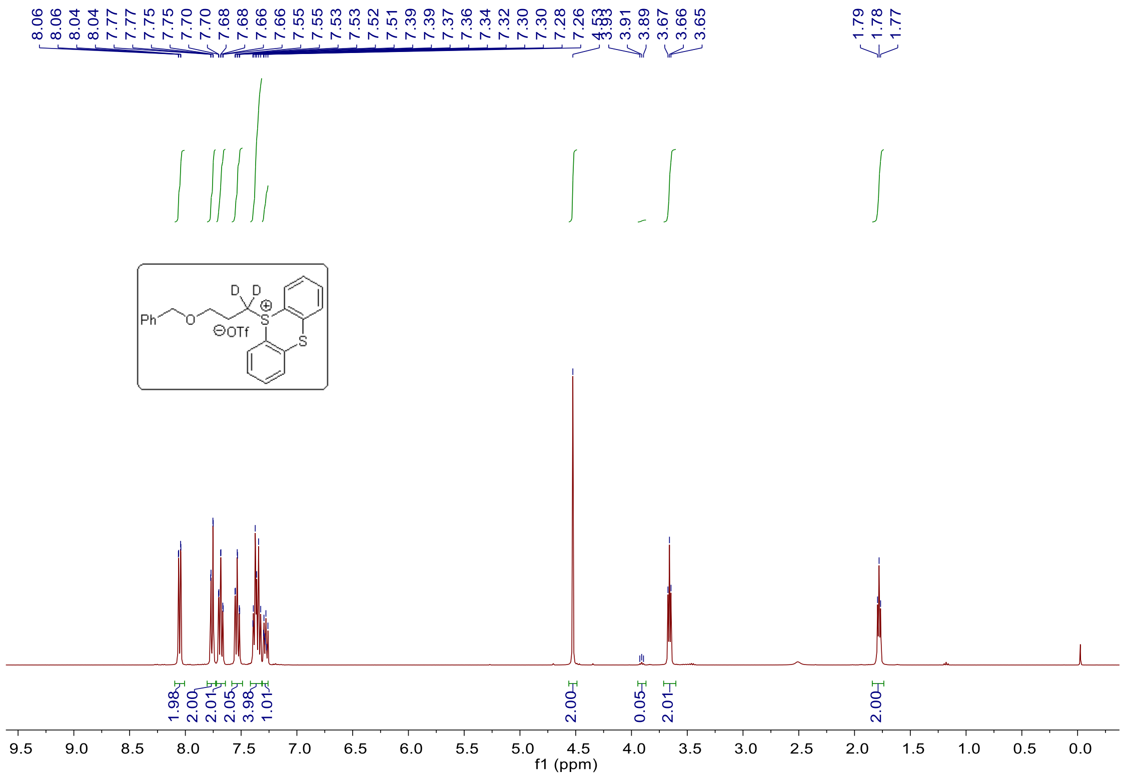

Supplementary Figure 57. ^{1}H NMR (400 MHz, $CDCl_3$) of **2h**

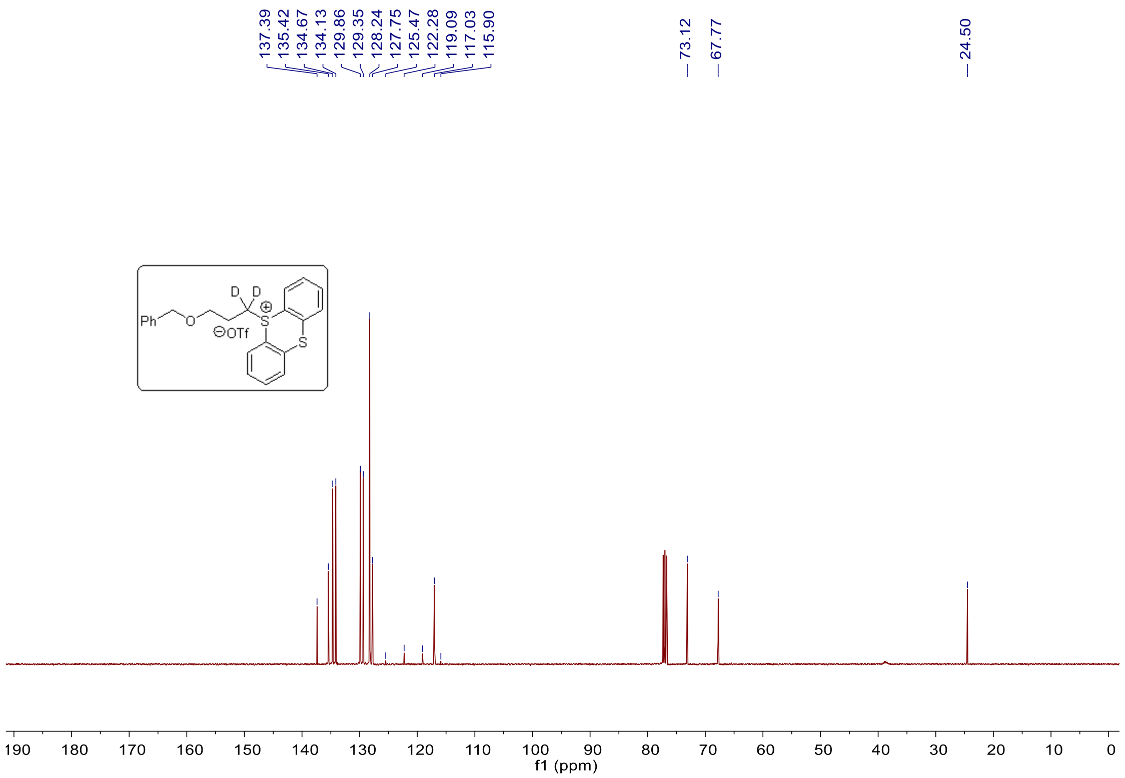

Supplementary Figure 58. ^{13}C NMR (101 MHz, $CDCl_3$) of **2h**

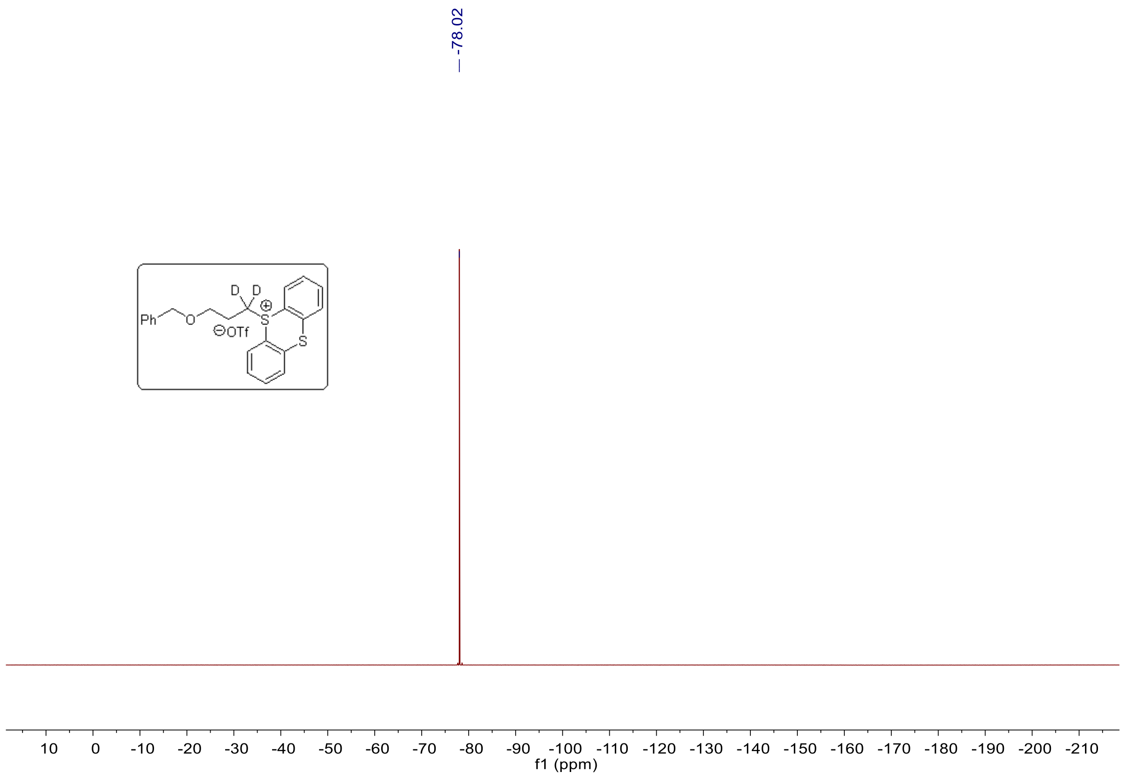

Supplementary Figure 59. ^{19}F NMR (376 MHz, $CDCl_3$) of **2h**

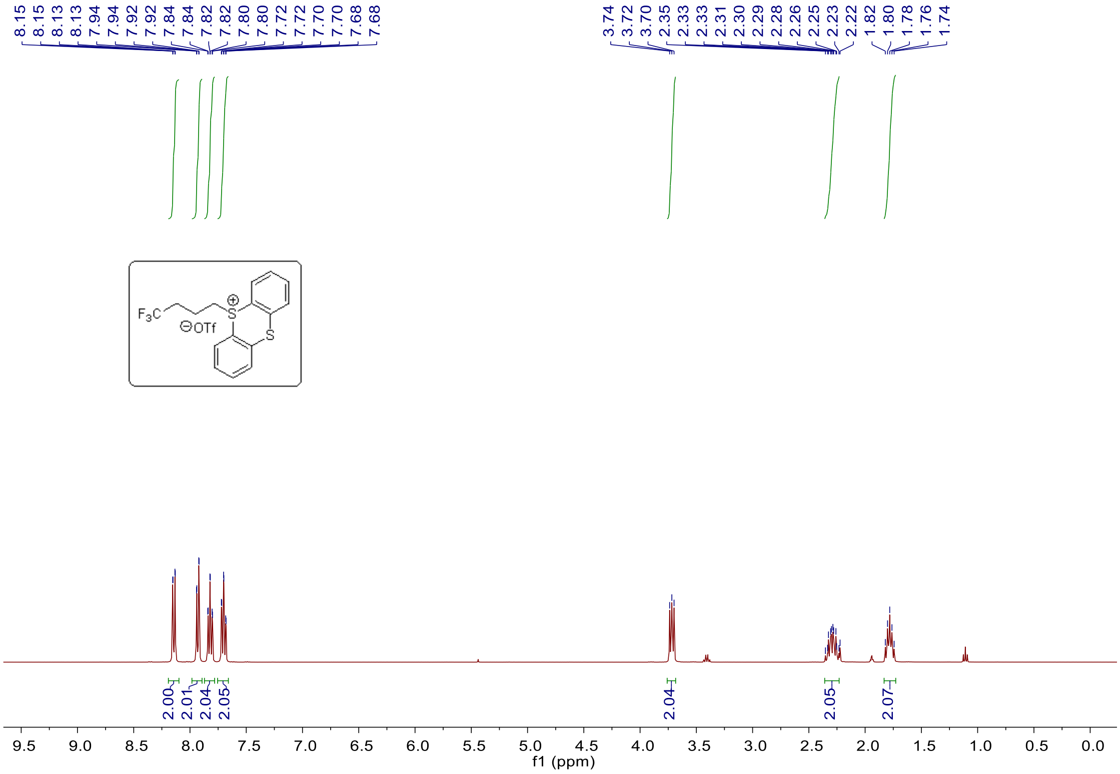

Supplementary Figure 60. ^{1}H NMR (400 MHz, CD_3CN) of **1i**

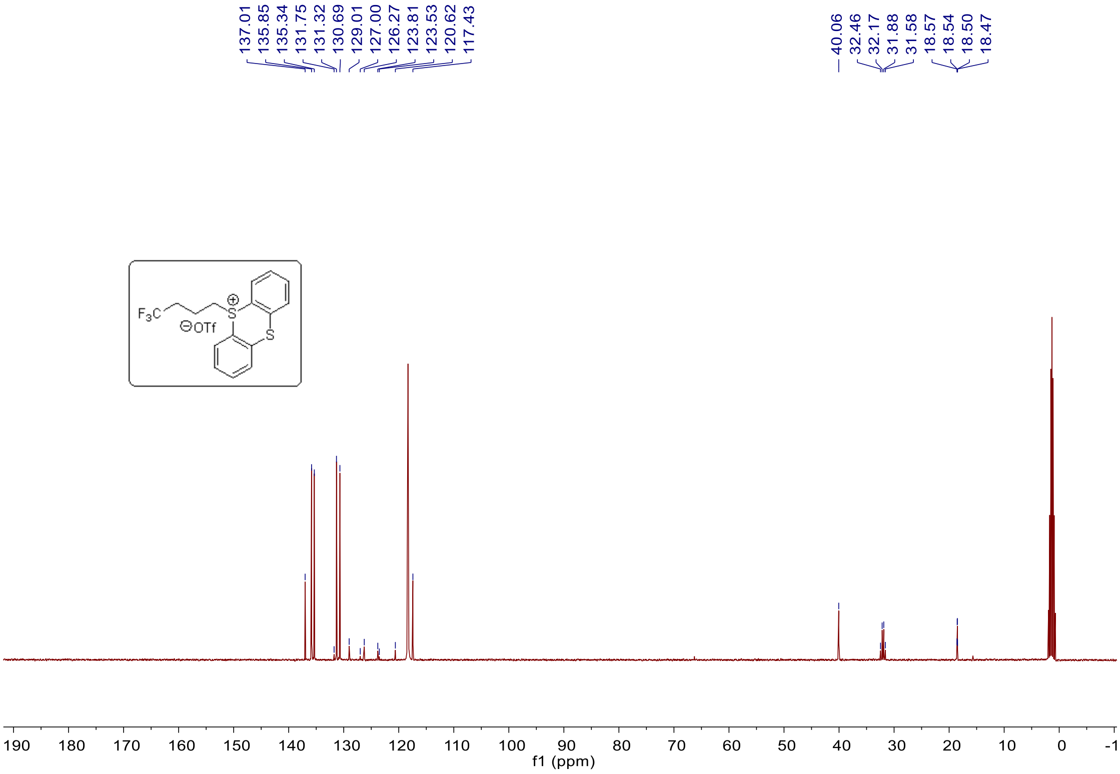

Supplementary Figure 61. ^{13}C NMR (101 MHz, CD_3CN) of **1i**

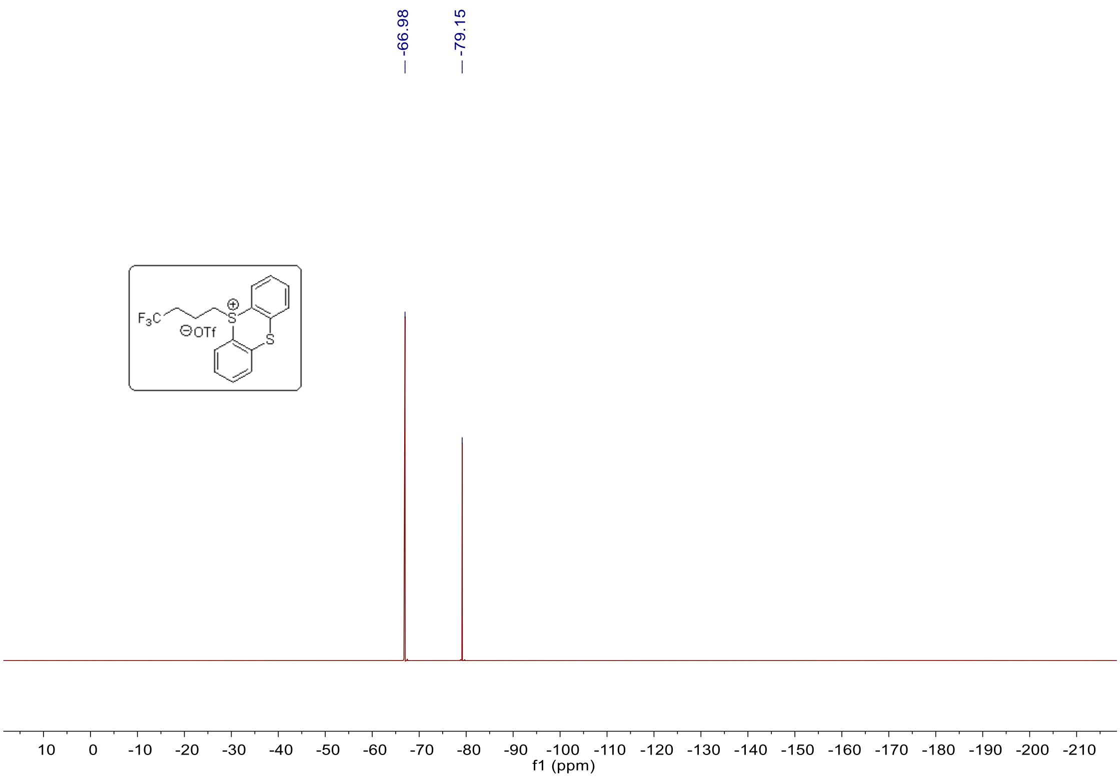

Supplementary Figure 62. ^{19}F NMR (376 MHz, CD_3CN) of **1i**

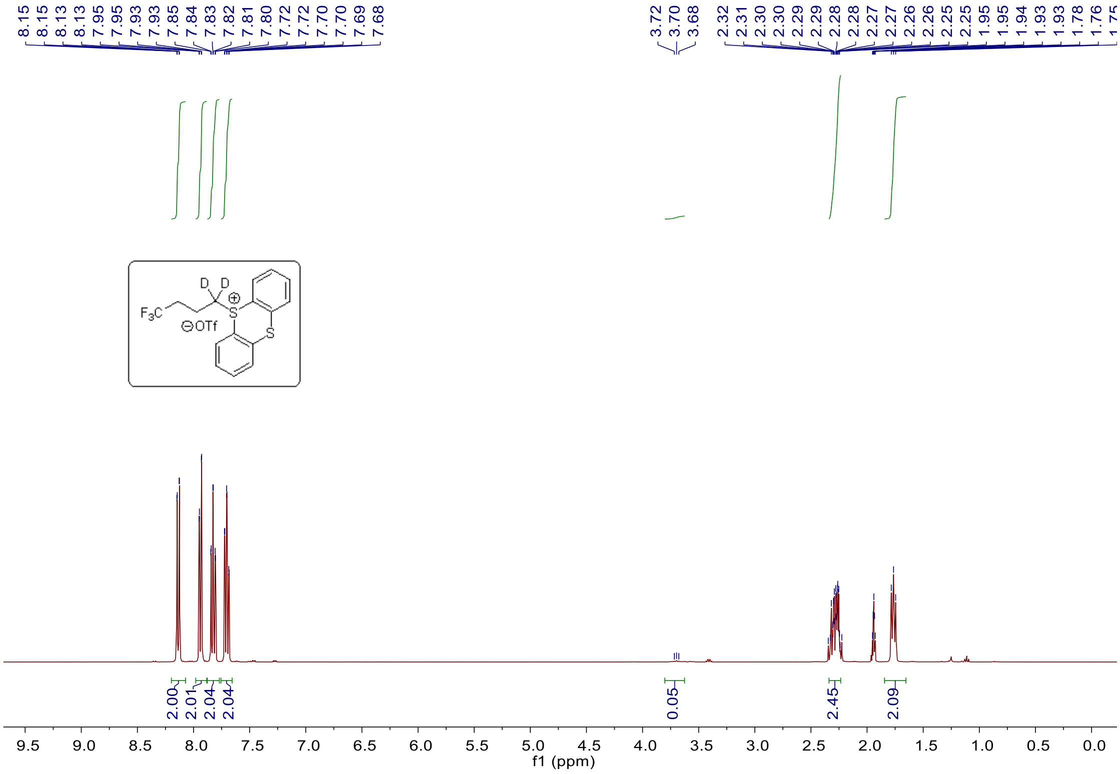

Supplementary Figure 63. ^{1}H NMR (400 MHz, CD_3CN) of **2i**

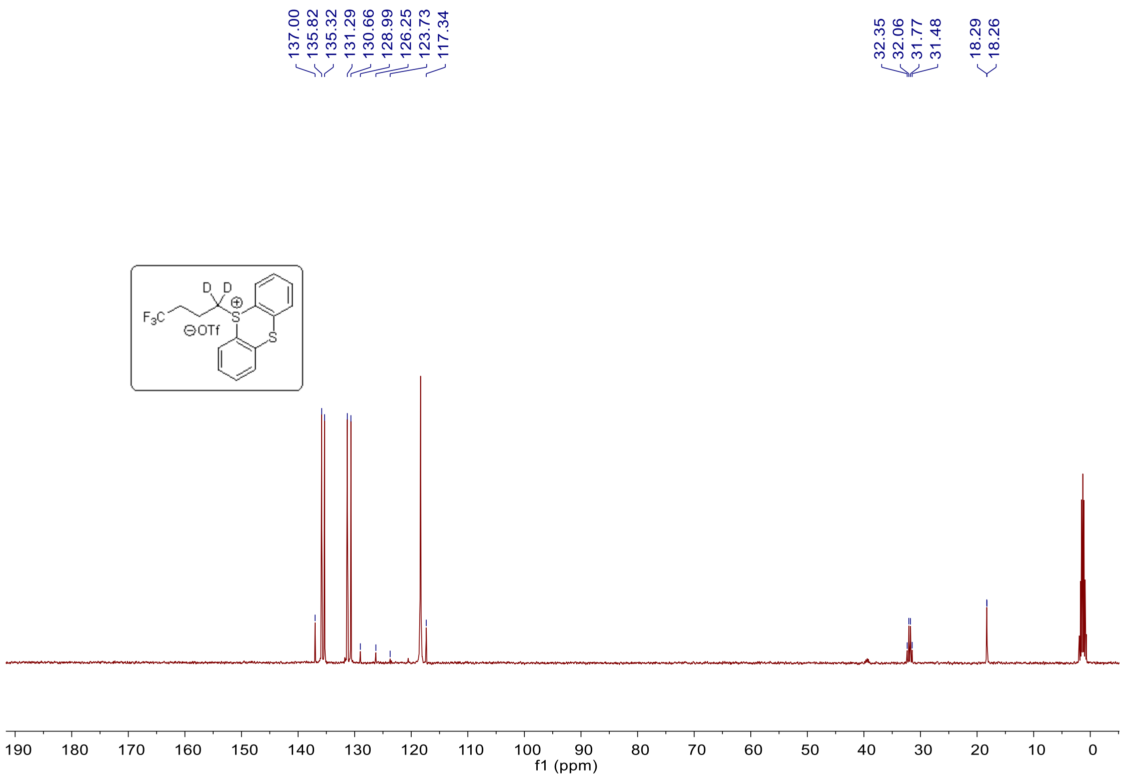

Supplementary Figure 64. ^{13}C NMR (101 MHz, CD_3CN) of **2i**

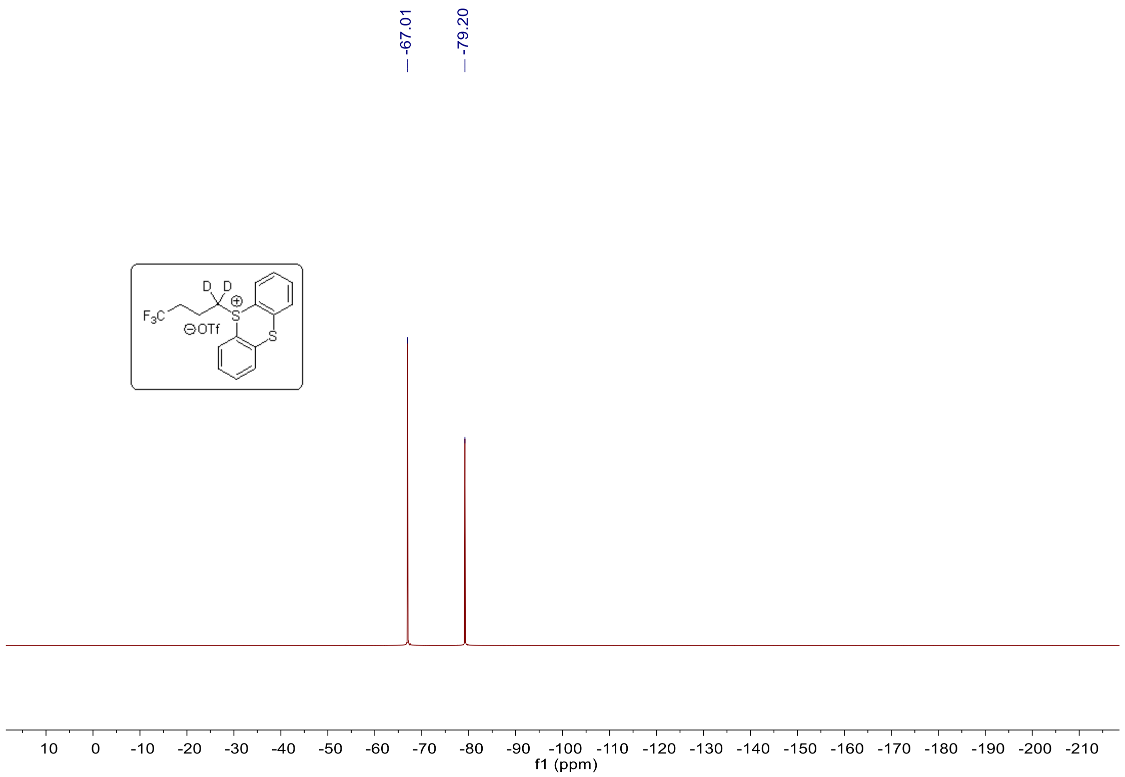

Supplementary Figure 65. ^{19}F NMR (376 MHz, CD_3CN) of **2i**

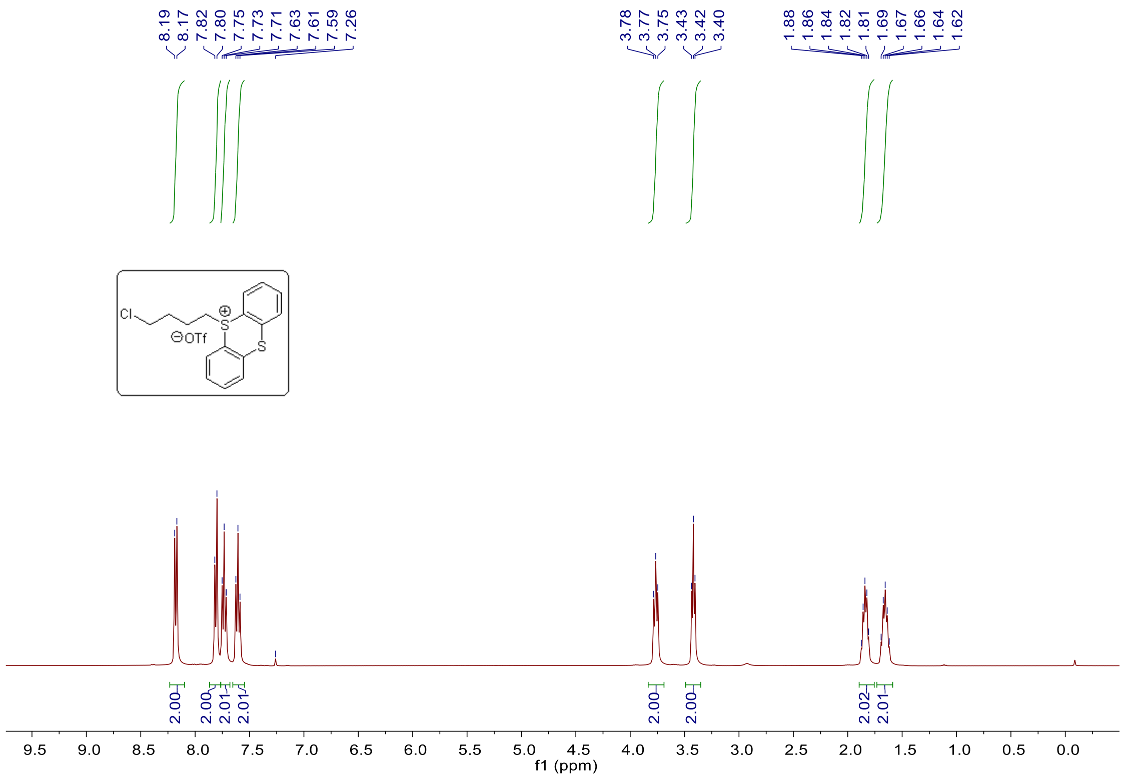

Supplementary Figure 66. ^{1}H NMR (400 MHz, $CDCl_3$) of **1j**

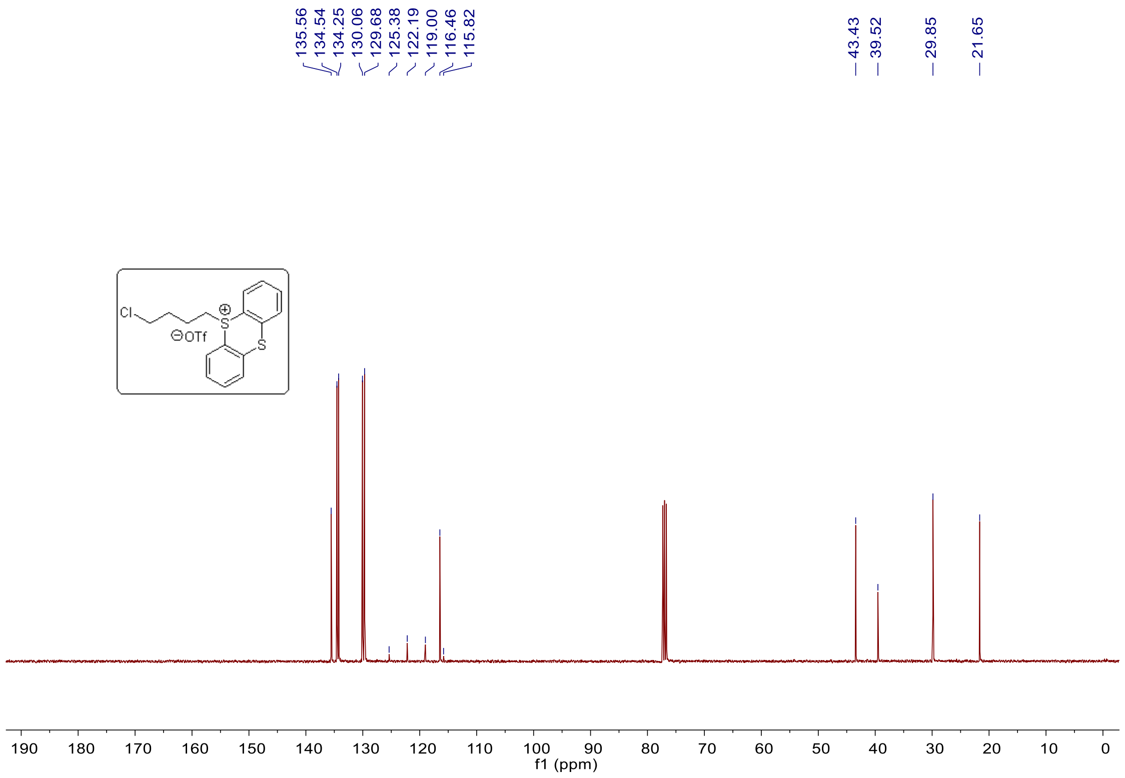

Supplementary Figure 67. ^{13}C NMR (101 MHz, $CDCl_3$) of **1j**

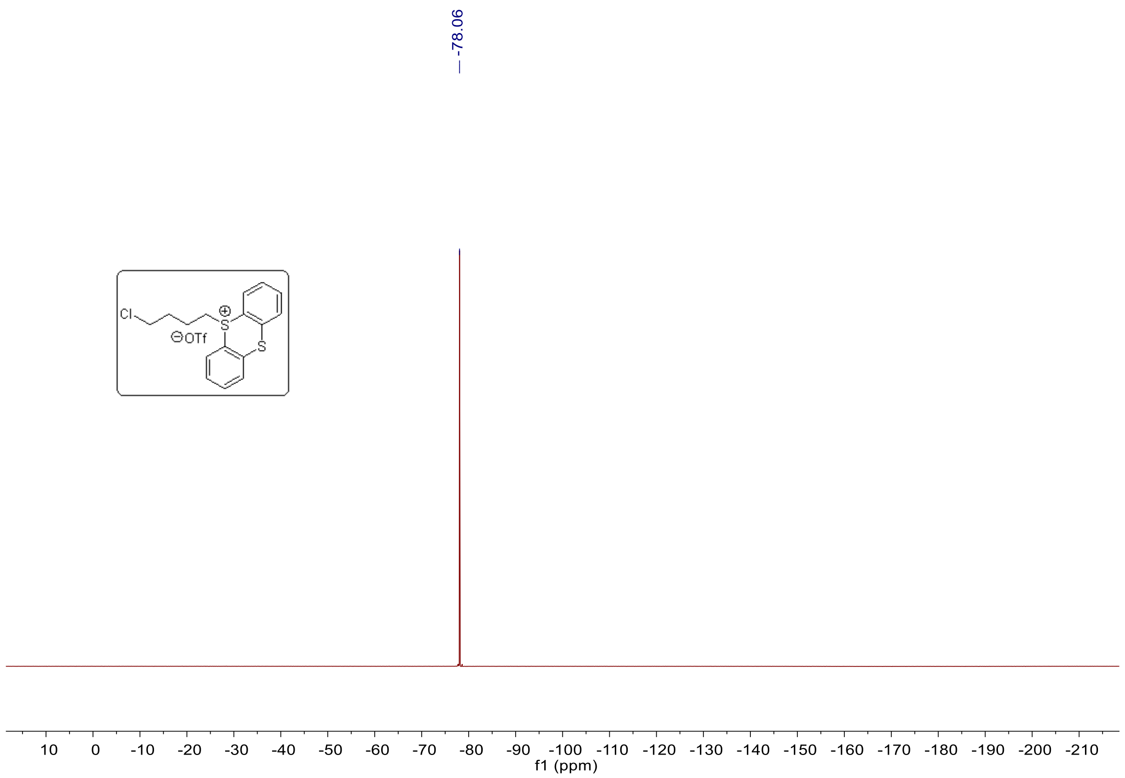

Supplementary Figure 68. ^{19}F NMR (376 MHz, $CDCl_3$) of **1j**

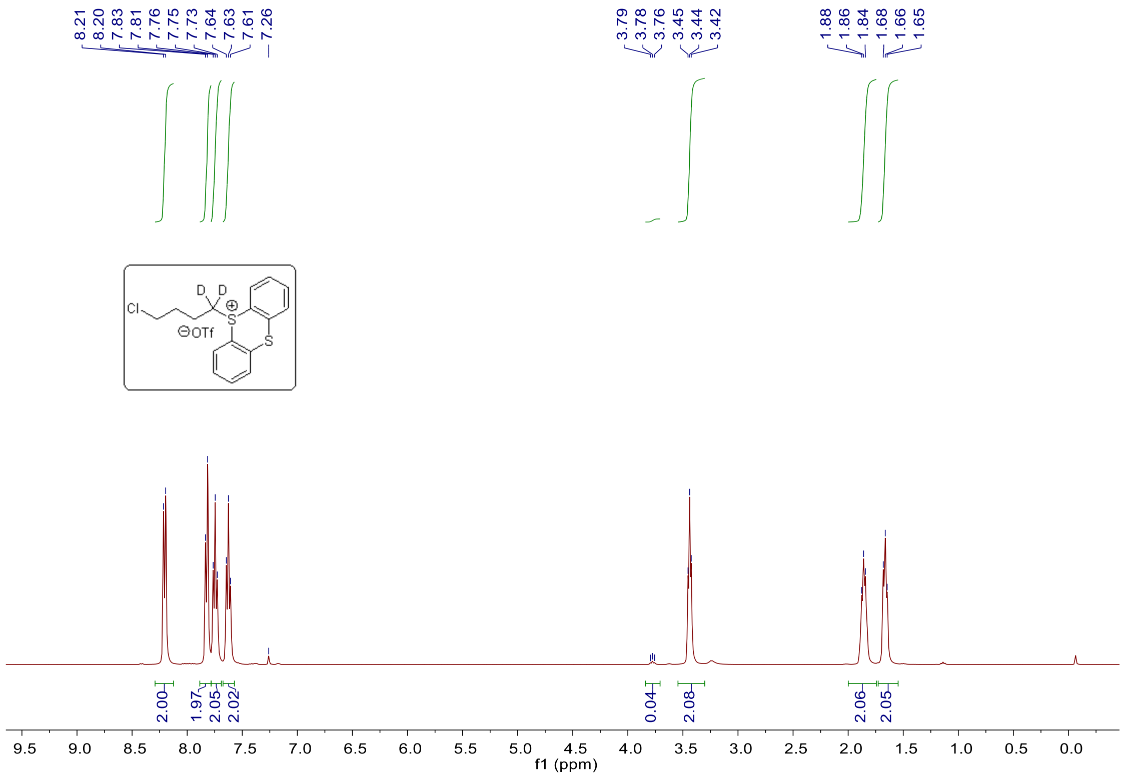

Supplementary Figure 69. ^{1}H NMR (400 MHz, $CDCl_3$) of **2j**

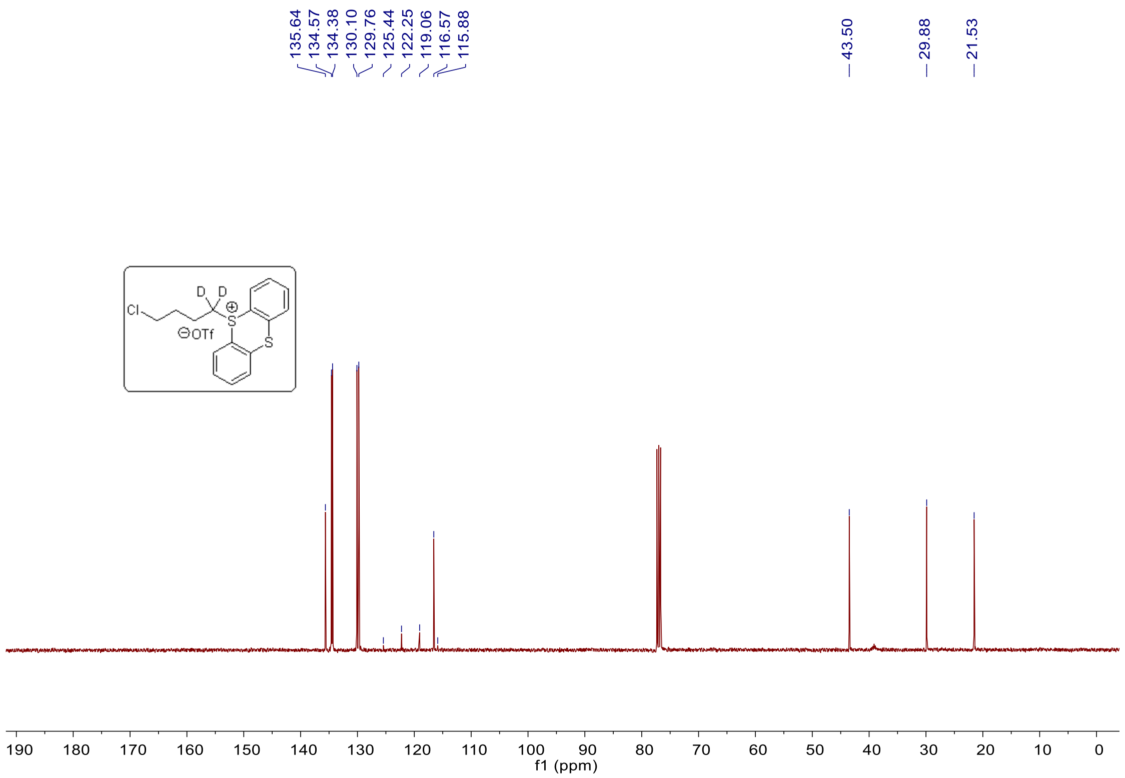

Supplementary Figure 70. ^{13}C NMR (101 MHz, $CDCl_3$) of **2j**

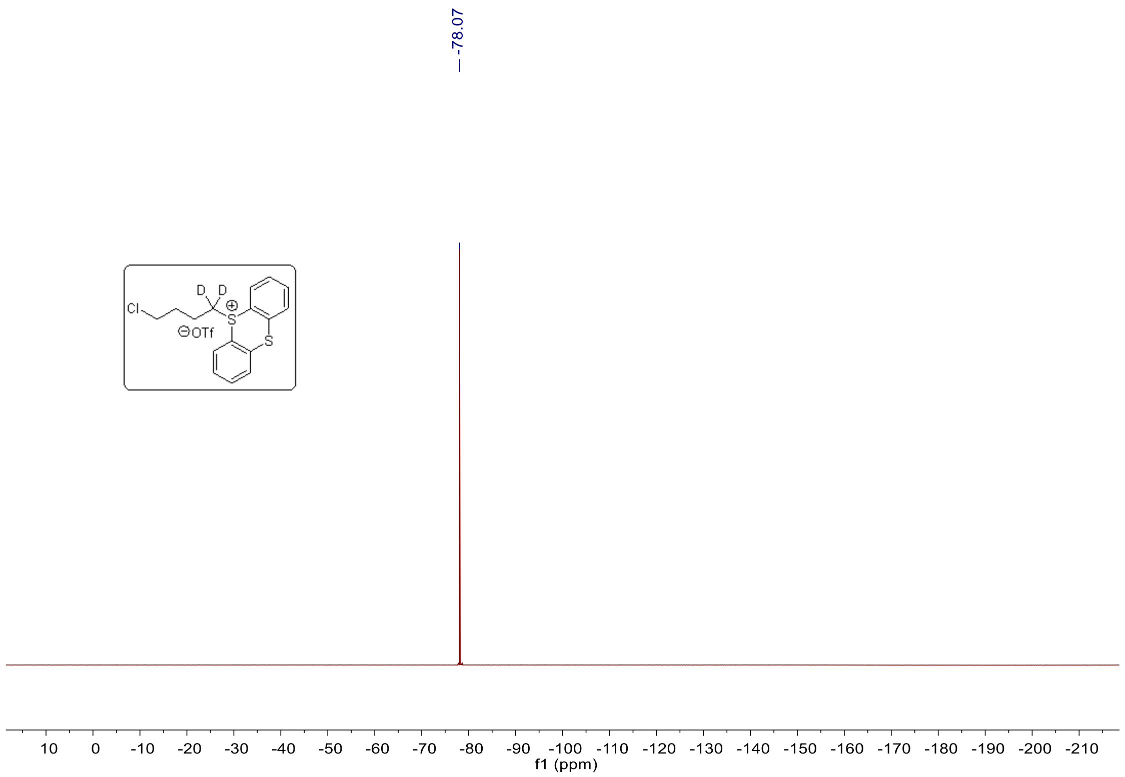

Supplementary Figure 71. ^{19}F NMR (376 MHz, $CDCl_3$) of **2j**

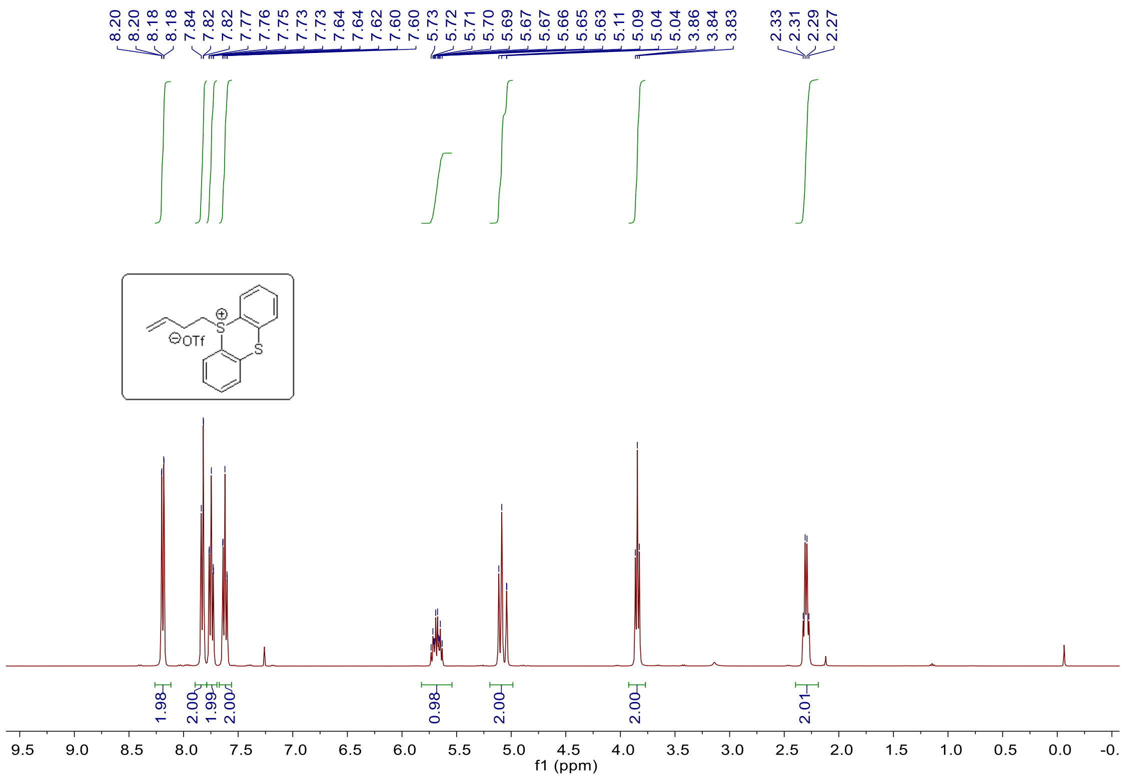

Supplementary Figure 72. ^{1}H NMR (400 MHz, $CDCl_3$) of **1k**

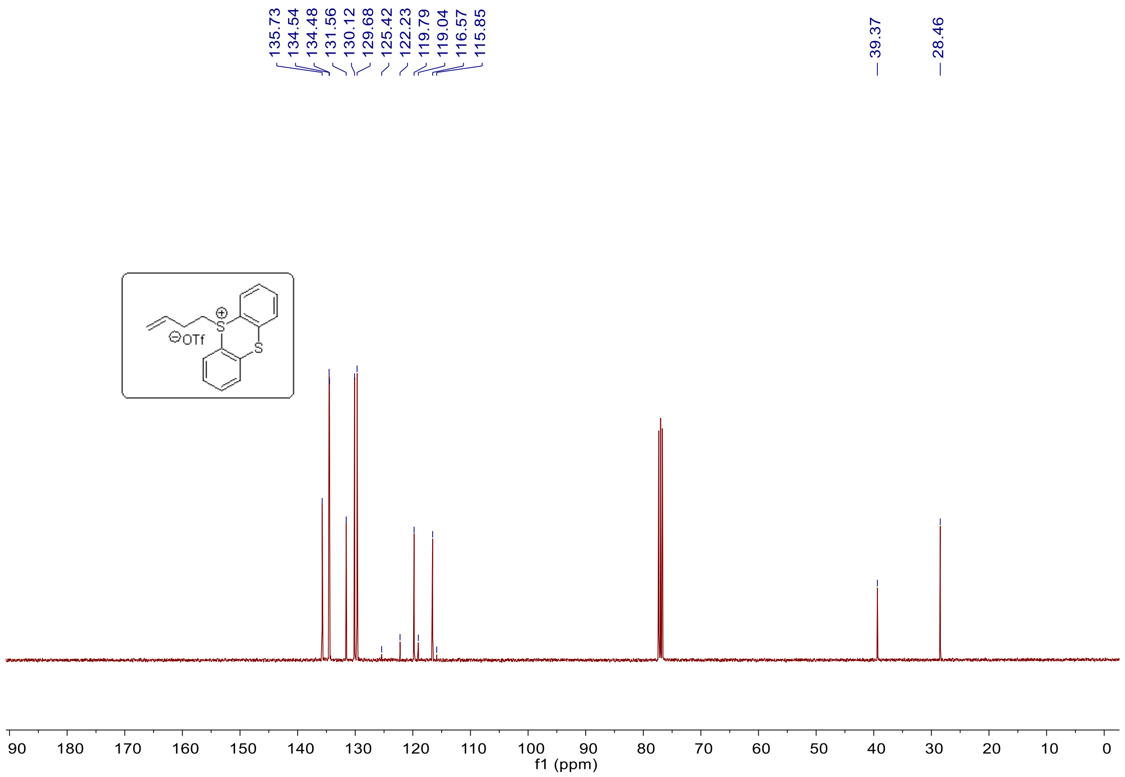

Supplementary Figure 73. ^{13}C NMR (101 MHz, $CDCl_3$) of **1k**

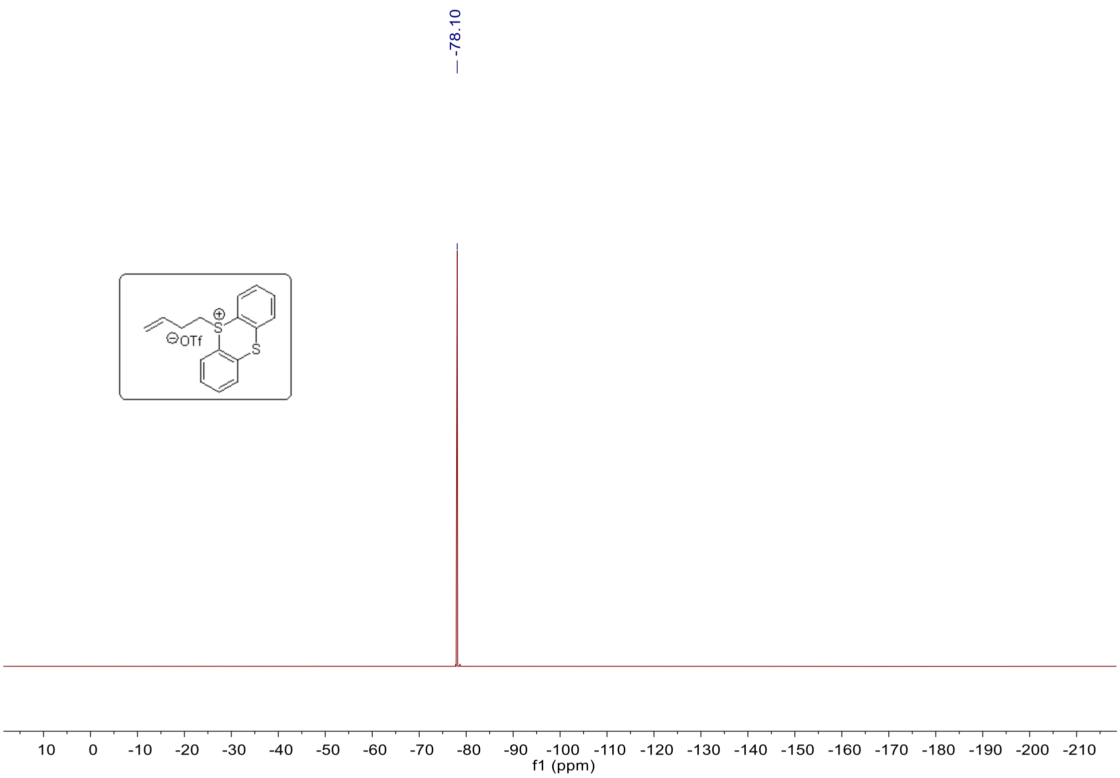

Supplementary Figure 74. ^{19}F NMR (376 MHz, $CDCl_3$) of **1k**

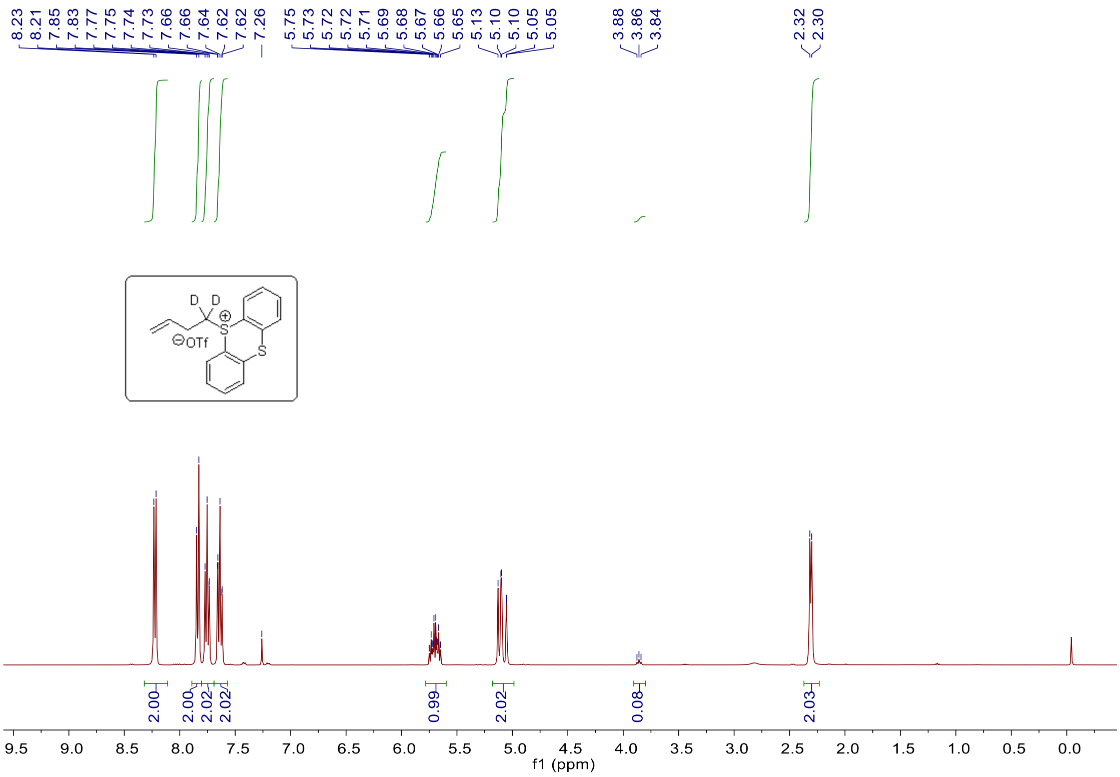

Supplementary Figure 75. ^{1}H NMR (400 MHz, $CDCl_3$) of **2k**

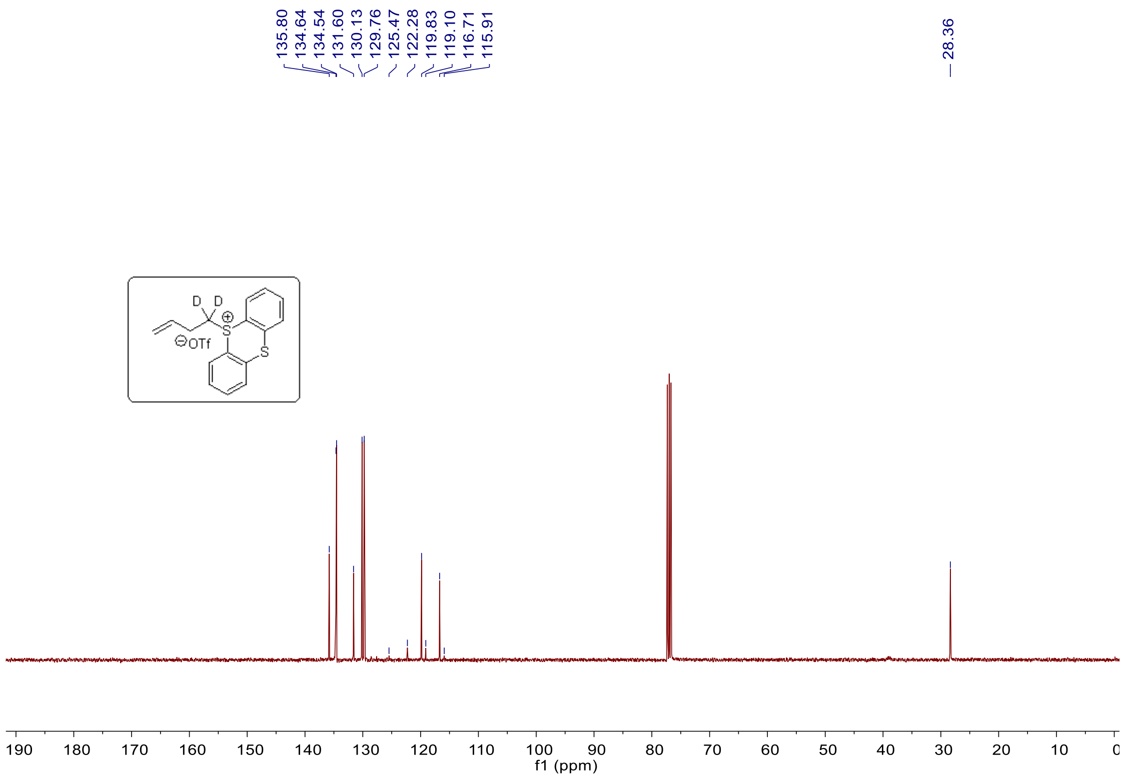

Supplementary Figure 76. ^{13}C NMR (101 MHz, $CDCl_3$) of **2k**

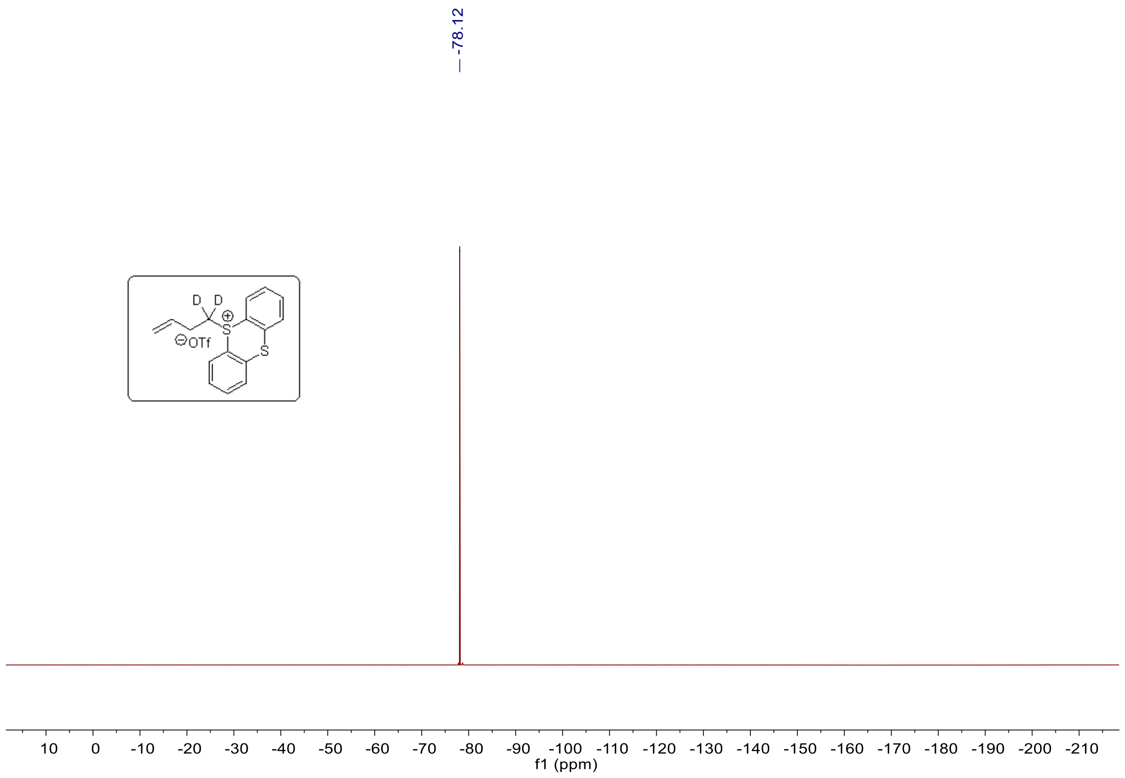

Supplementary Figure 77. ^{19}F NMR (376 MHz, $CDCl_3$) of **2k**

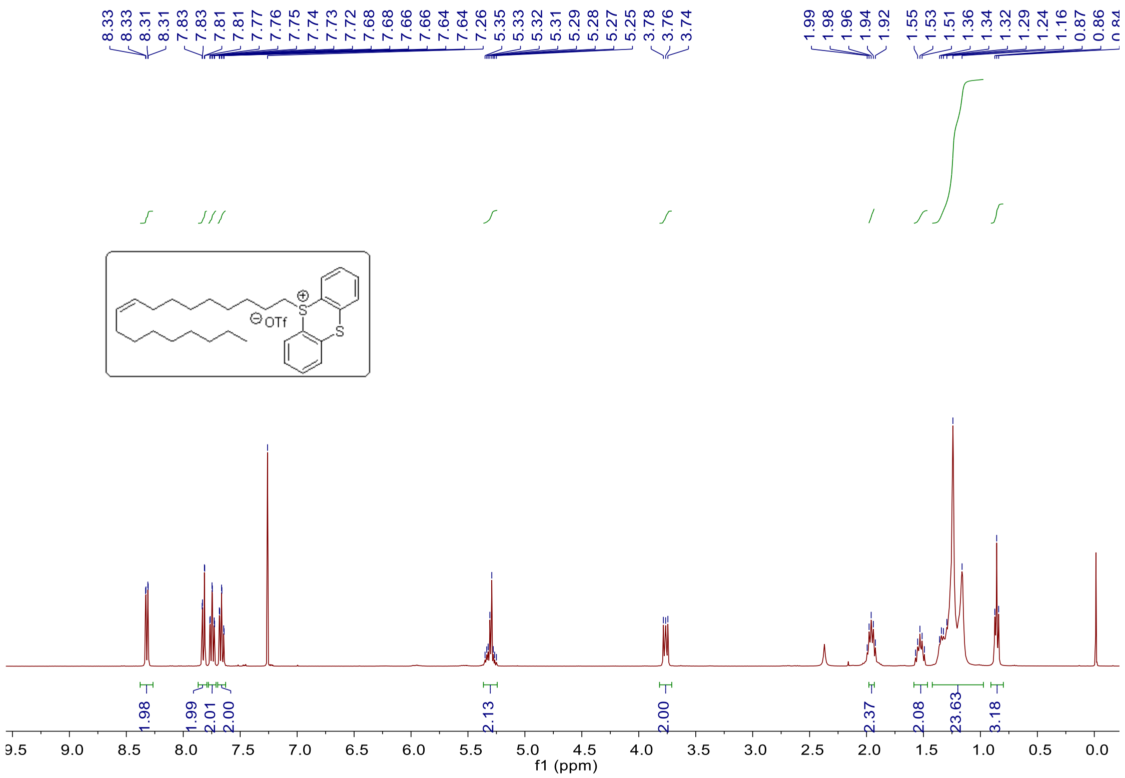

Supplementary Figure 78. ^{1}H NMR (400 MHz, $CDCl_3$) of **1l**

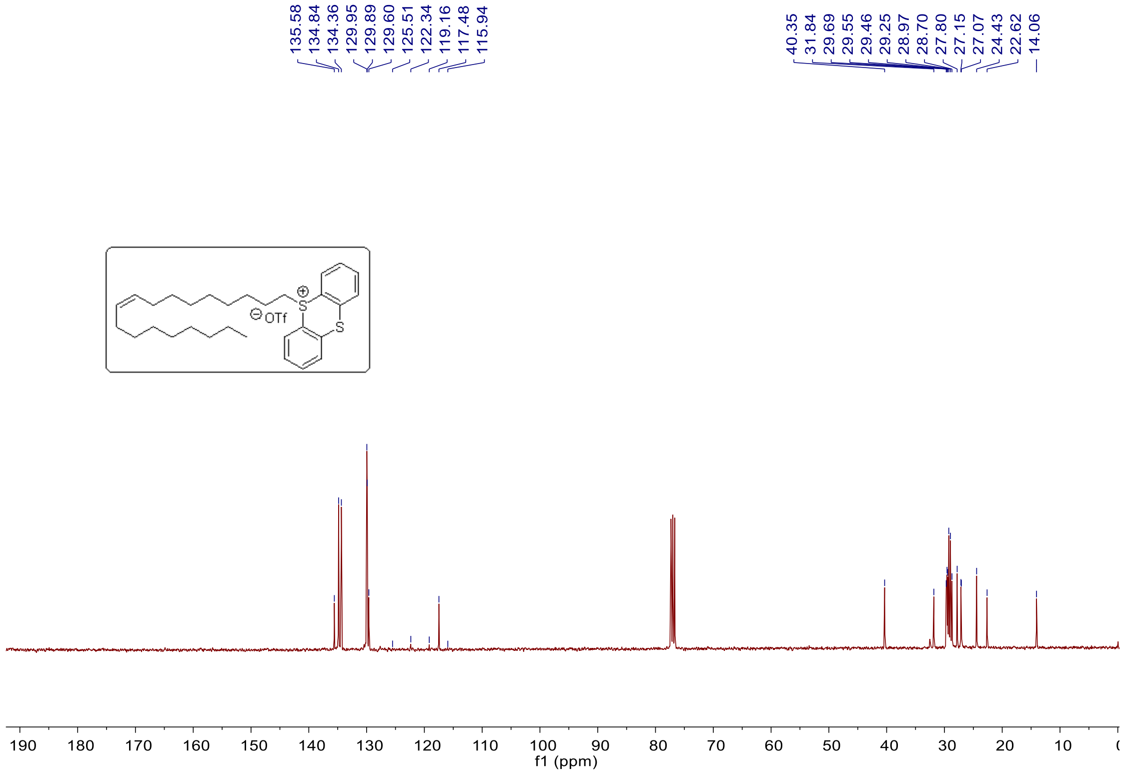

Supplementary Figure 79. ^{13}C NMR (101 MHz, $CDCl_3$) of **1l**

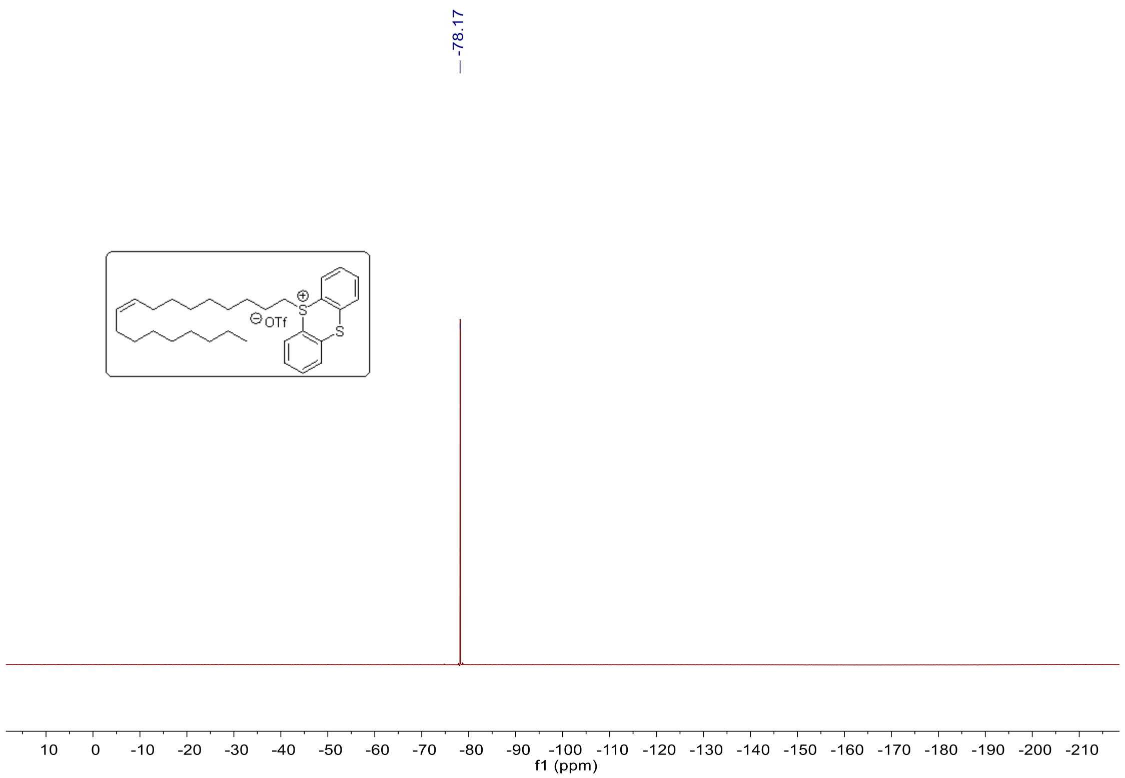

Supplementary Figure 80. ^{19}F NMR (376 MHz, $CDCl_3$) of **1l**

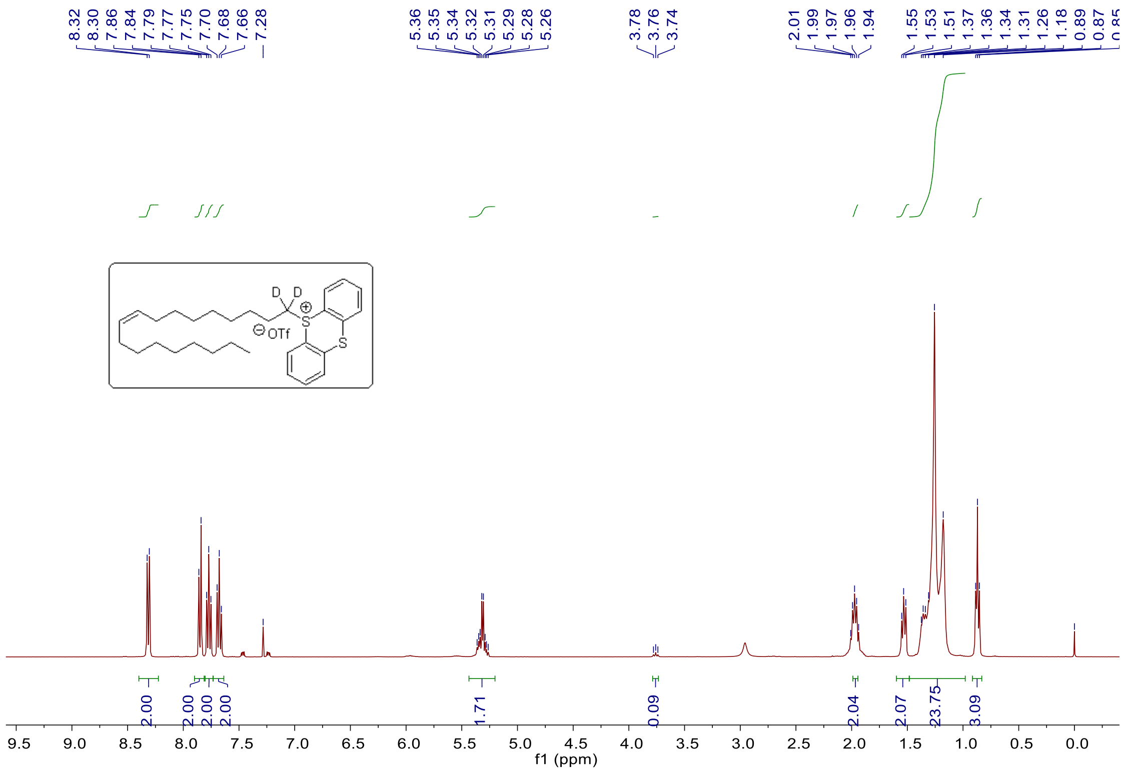

Supplementary Figure 81. ^{1}H NMR (400 MHz, $CDCl_3$) of **2l**

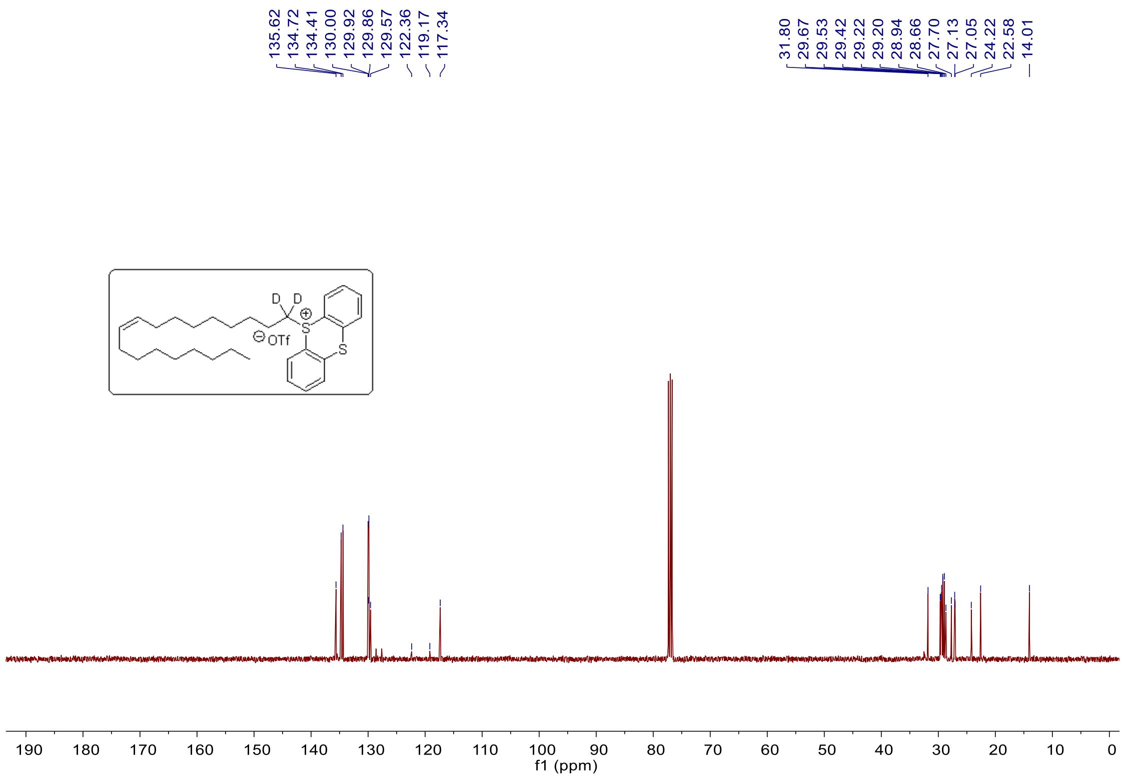

Supplementary Figure 82. ^{13}C NMR (101 MHz, $CDCl_3$) of **2l**

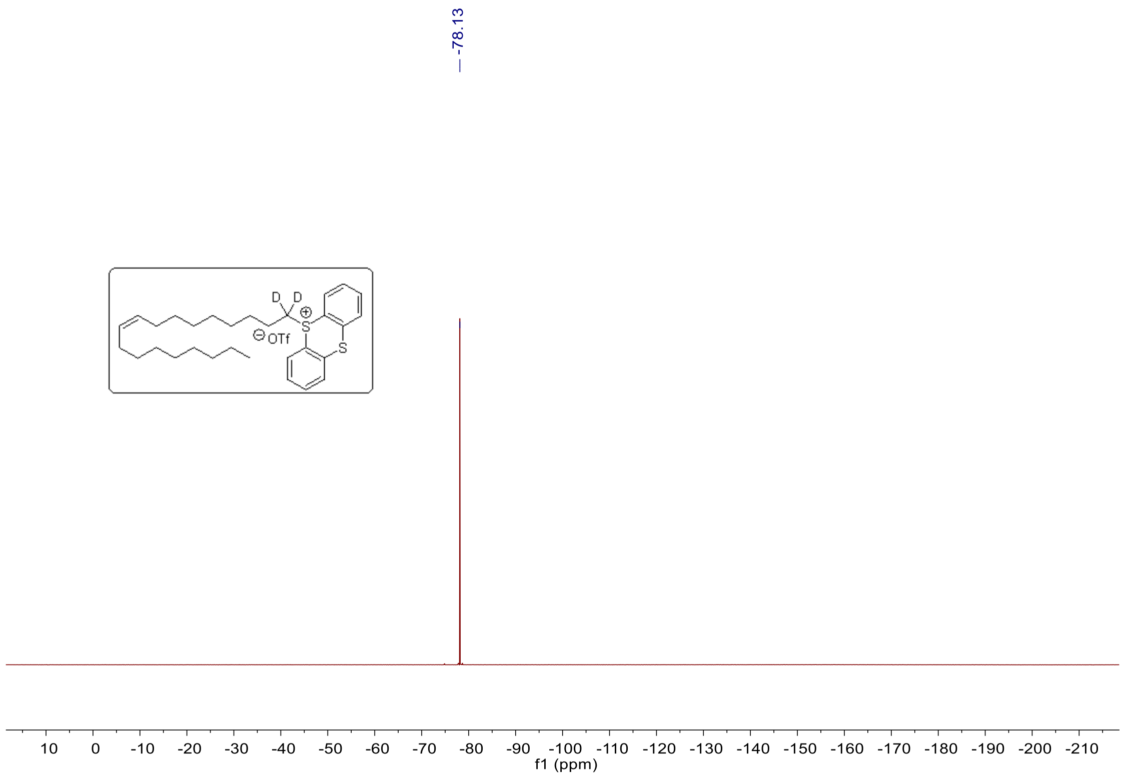

Supplementary Figure 83. ^{19}F NMR (376 MHz, $CDCl_3$) of **2l**

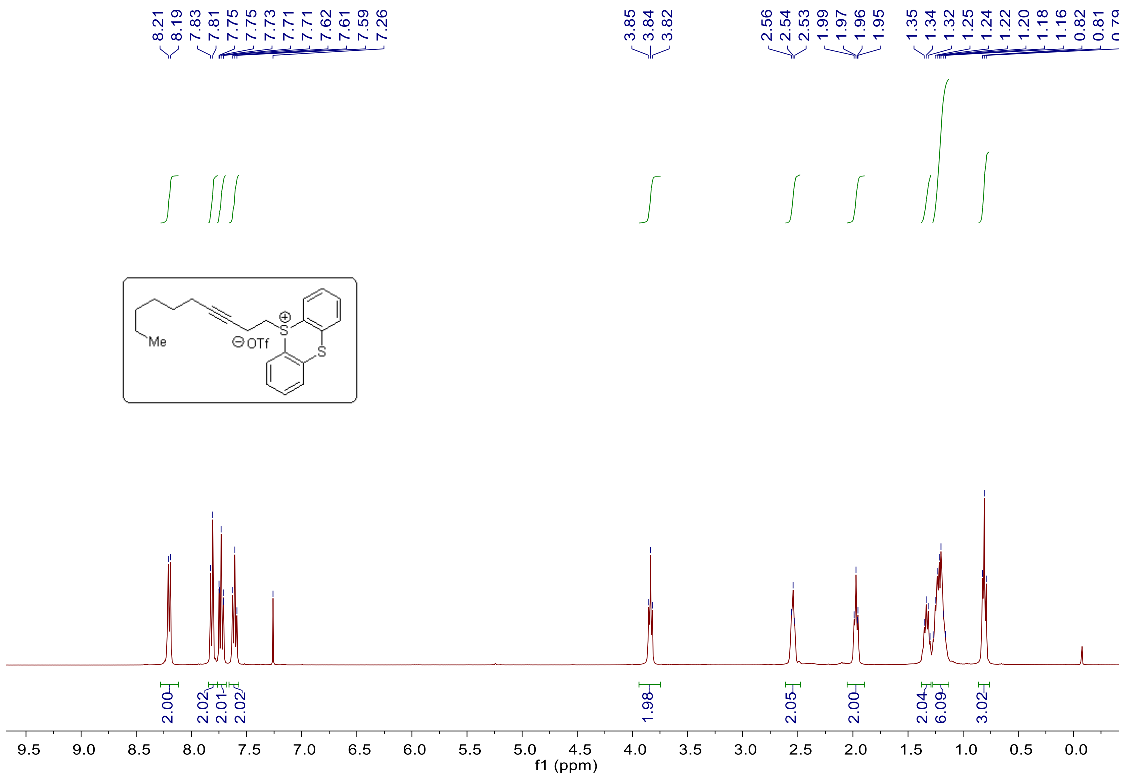

Supplementary Figure 84. ^{1}H NMR (400 MHz, $CDCl_3$) of **1m**

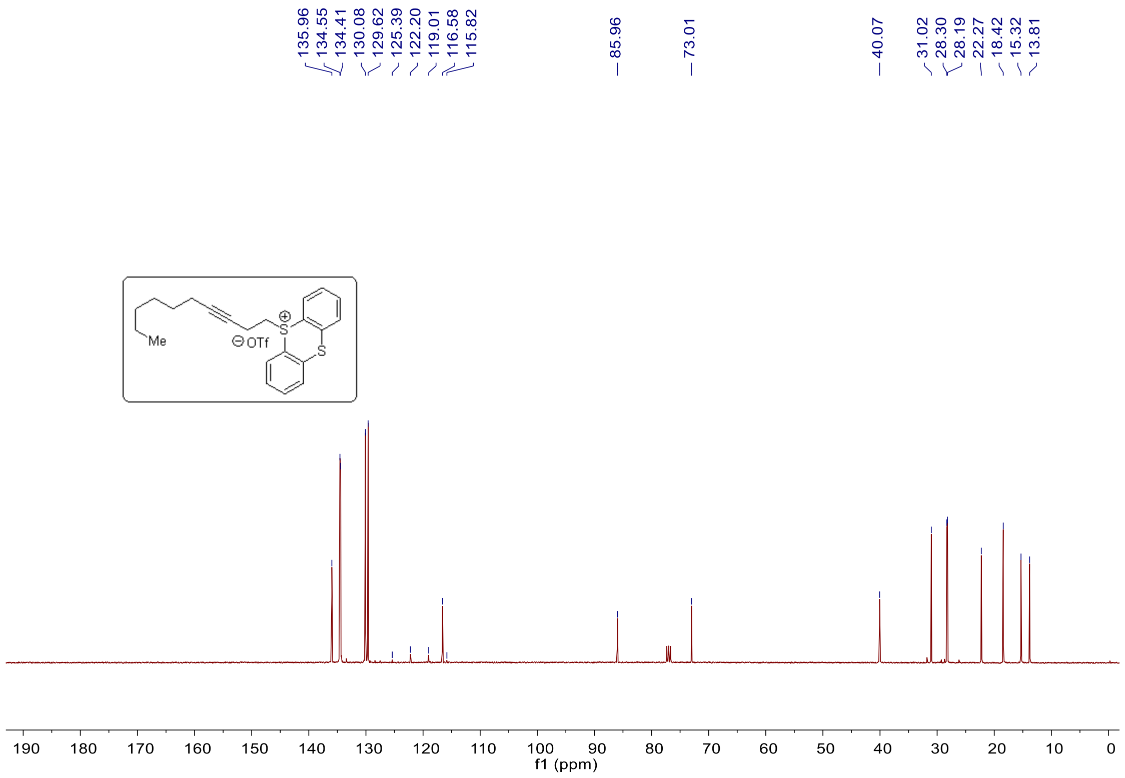

Supplementary Figure 85. ^{13}C NMR (101 MHz, $CDCl_3$) of **1m**

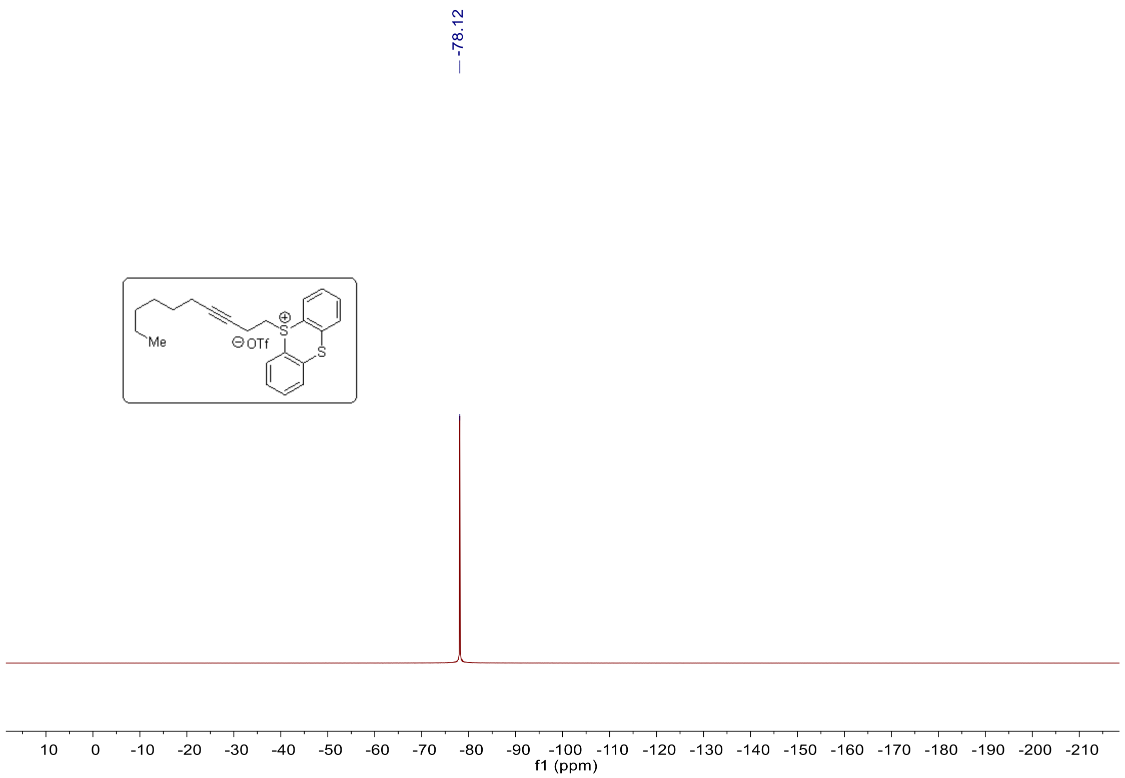

Supplementary Figure 86. ^{19}F NMR (376 MHz, $CDCl_3$) of **1m**

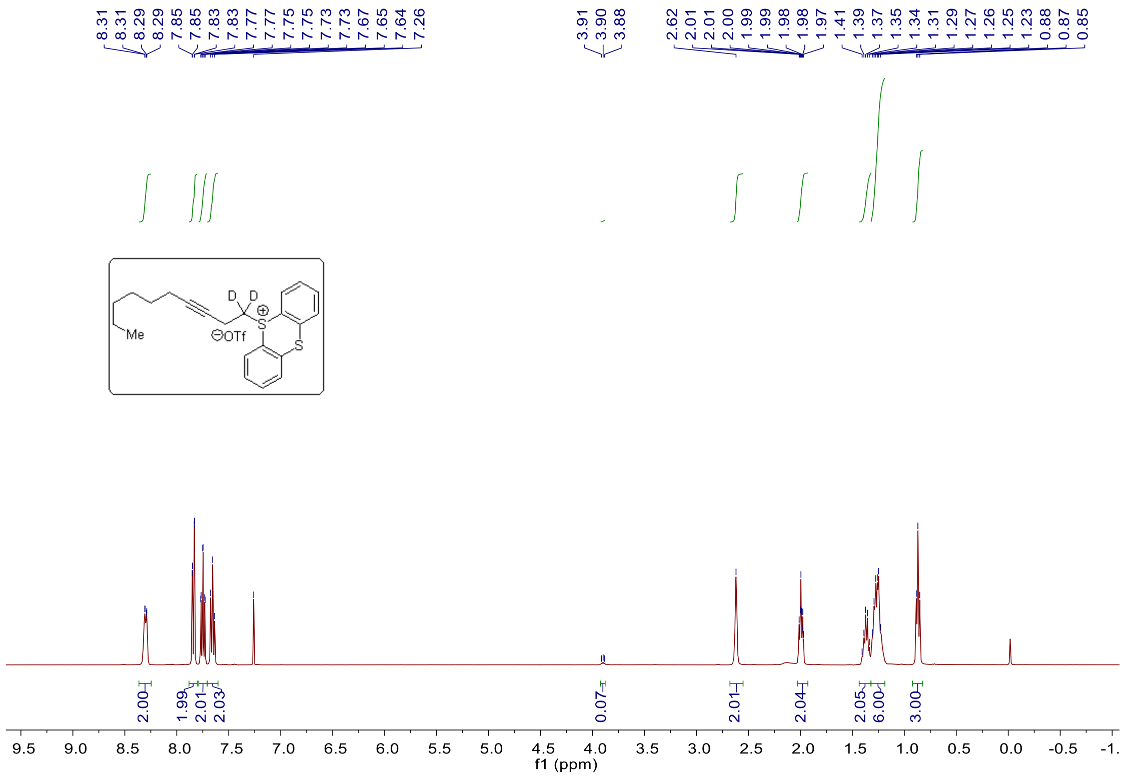

Supplementary Figure 87. ^{1}H NMR (400 MHz, $CDCl_3$) of **2m**

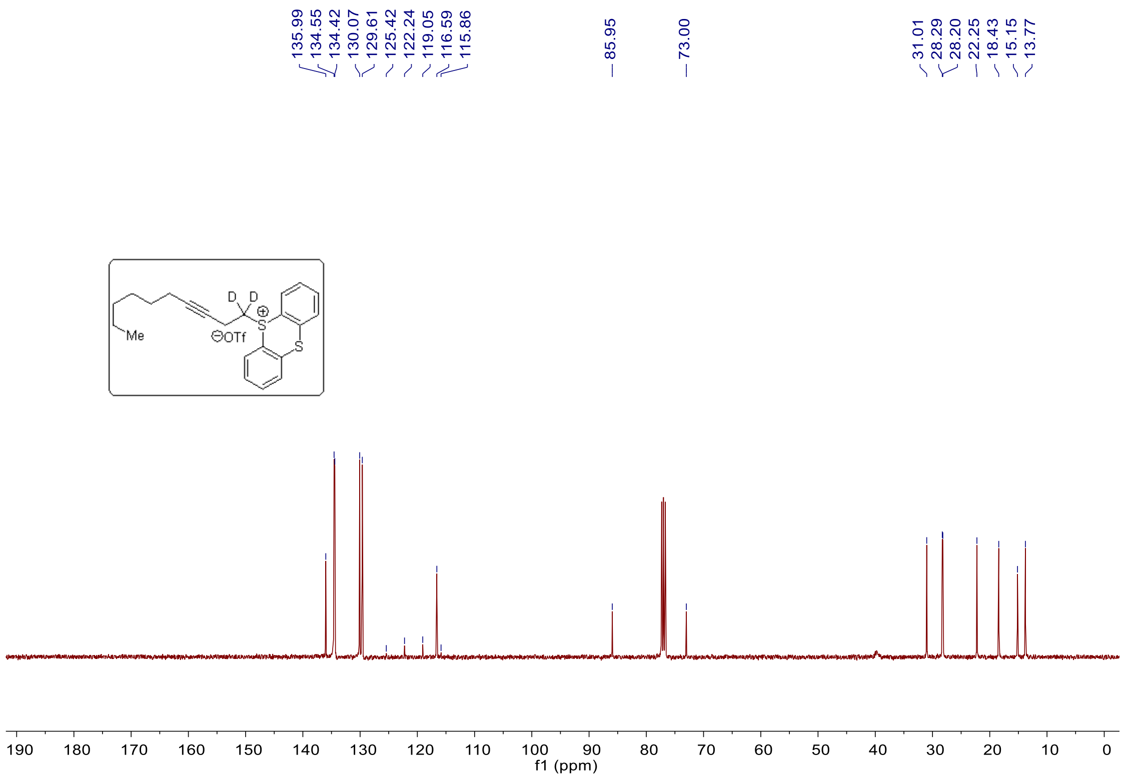

Supplementary Figure 88. ^{13}C NMR (101 MHz, $CDCl_3$) of **2m**

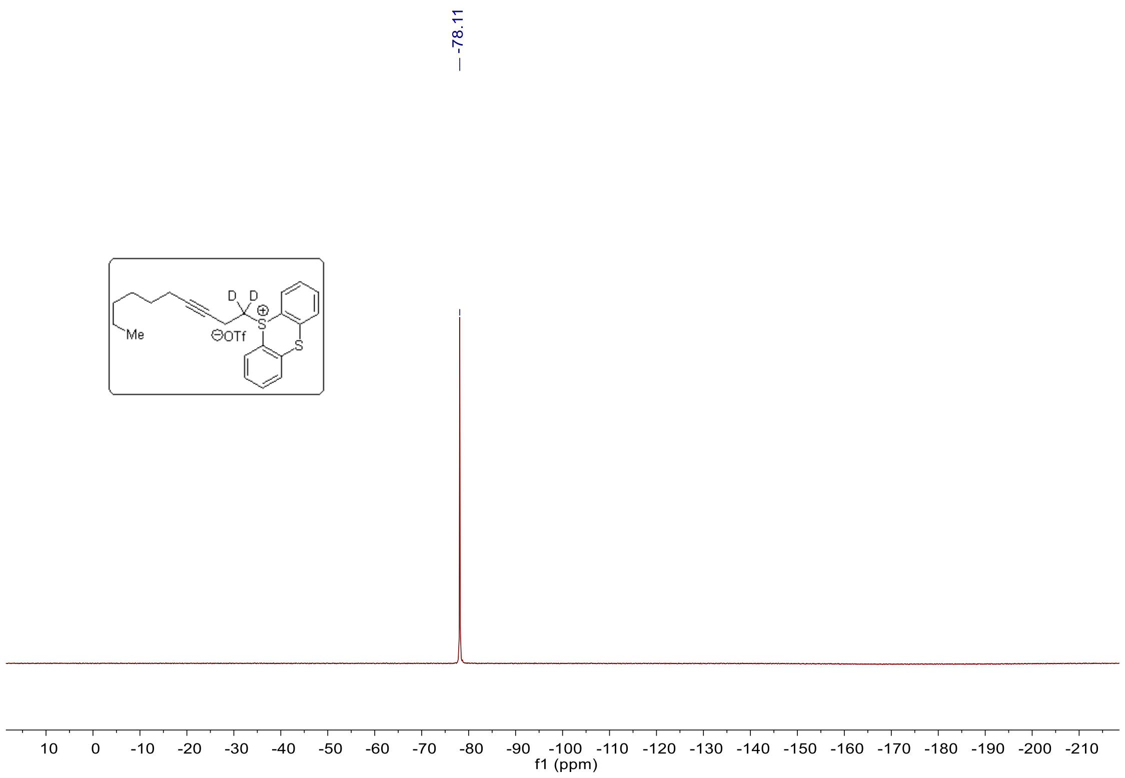

Supplementary Figure 89. ^{19}F NMR (376 MHz, $CDCl_3$) of **2m**

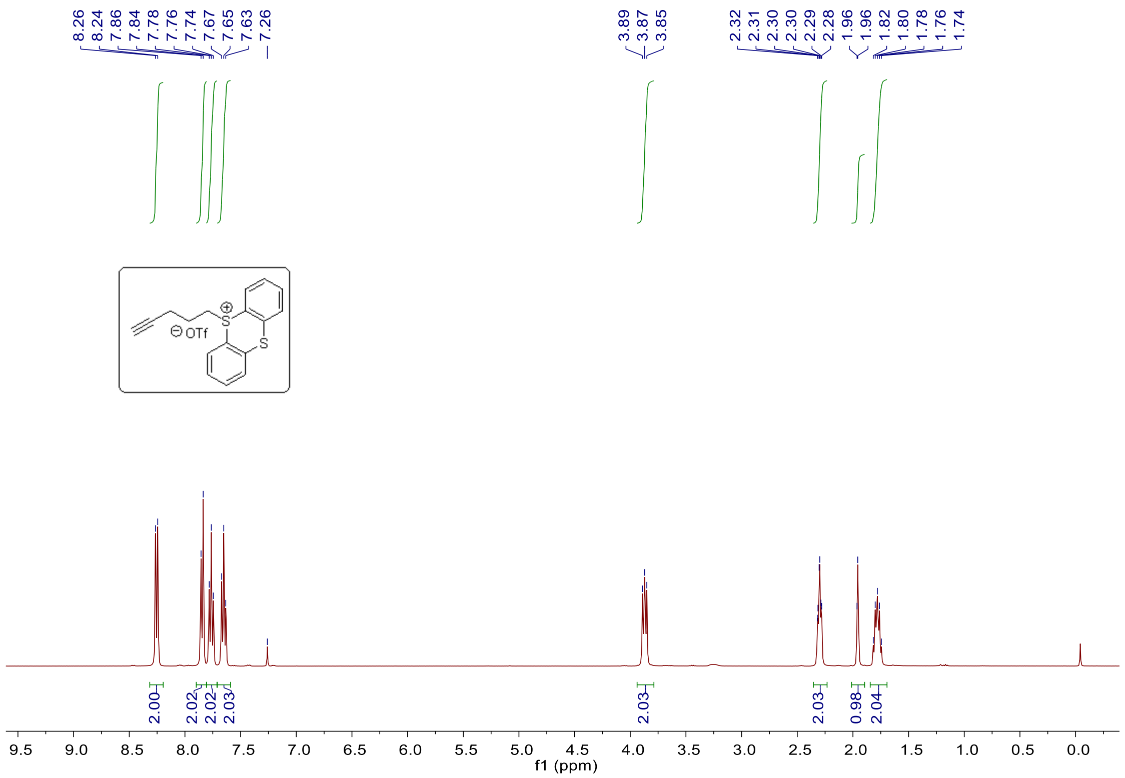

Supplementary Figure 90. ^{1}H NMR (400 MHz, $CDCl_3$) of **1n**

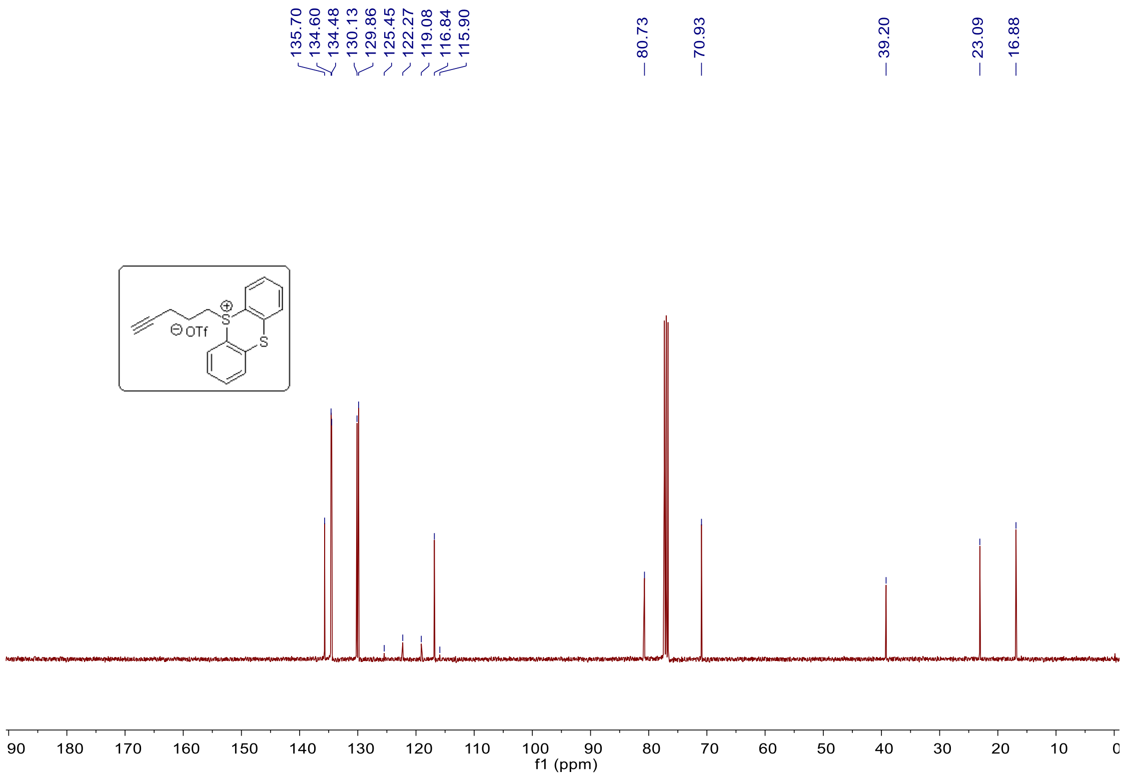

Supplementary Figure 91. ^{13}C NMR (101 MHz, $CDCl_3$) of **1n**

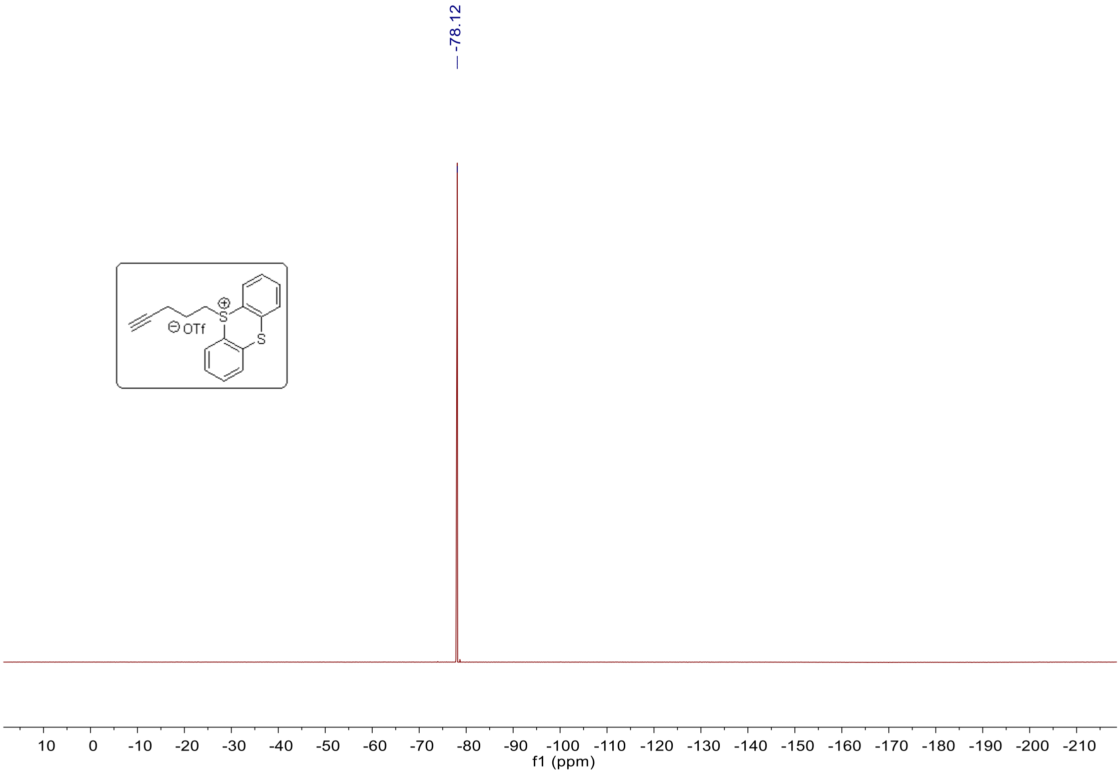

Supplementary Figure 92. ^{19}F NMR (376 MHz, CDCl$_3$) of **1n**

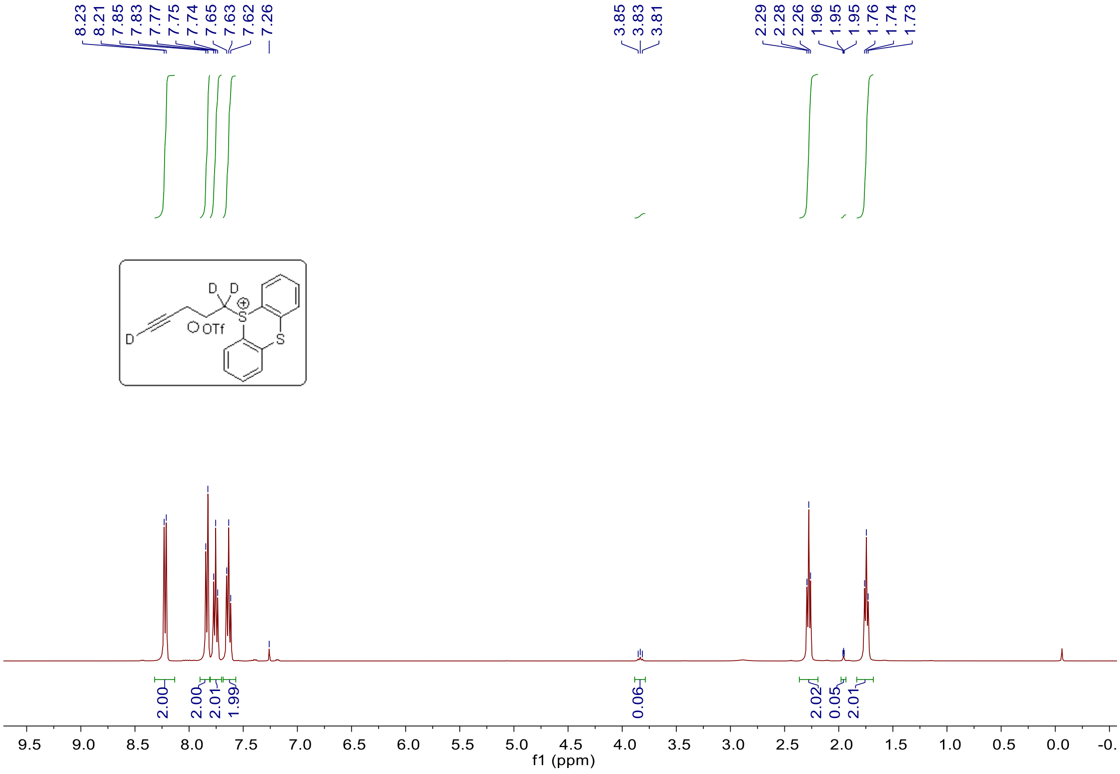

Supplementary Figure 93. ^{1}H NMR (400 MHz, CDCl$_3$) of **2n**

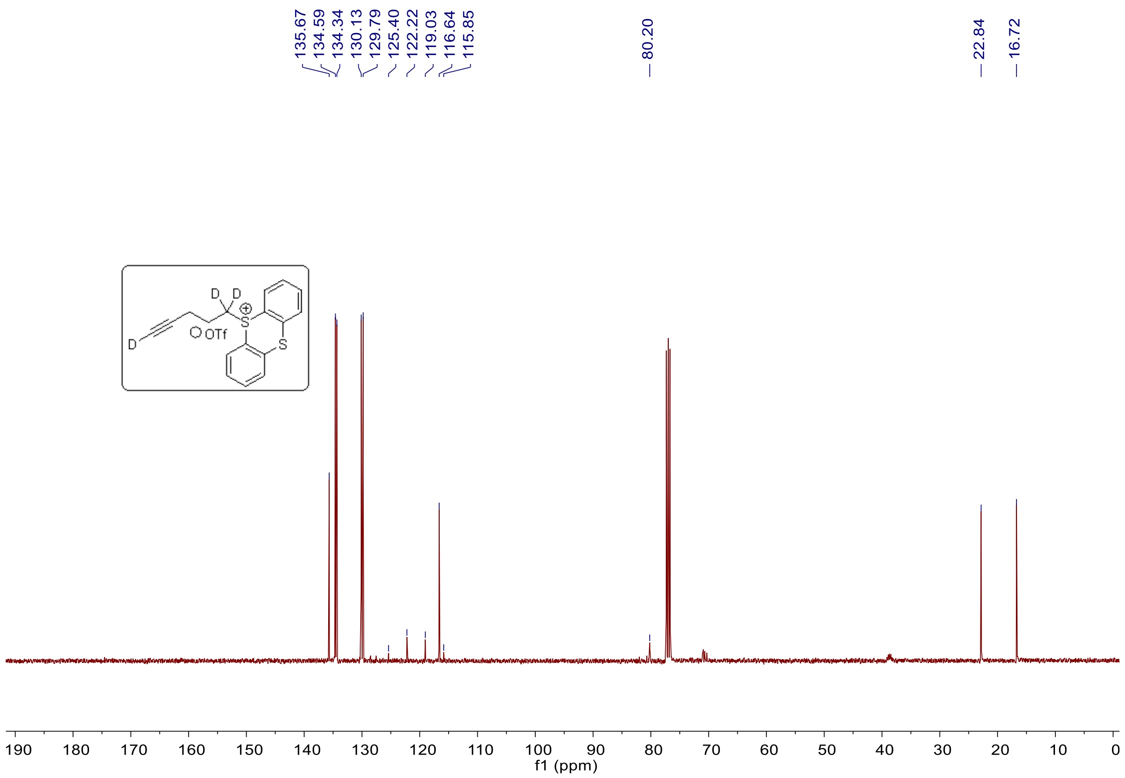

Supplementary Figure 94. ^{13}C NMR (101 MHz, $CDCl_3$) of **2n**

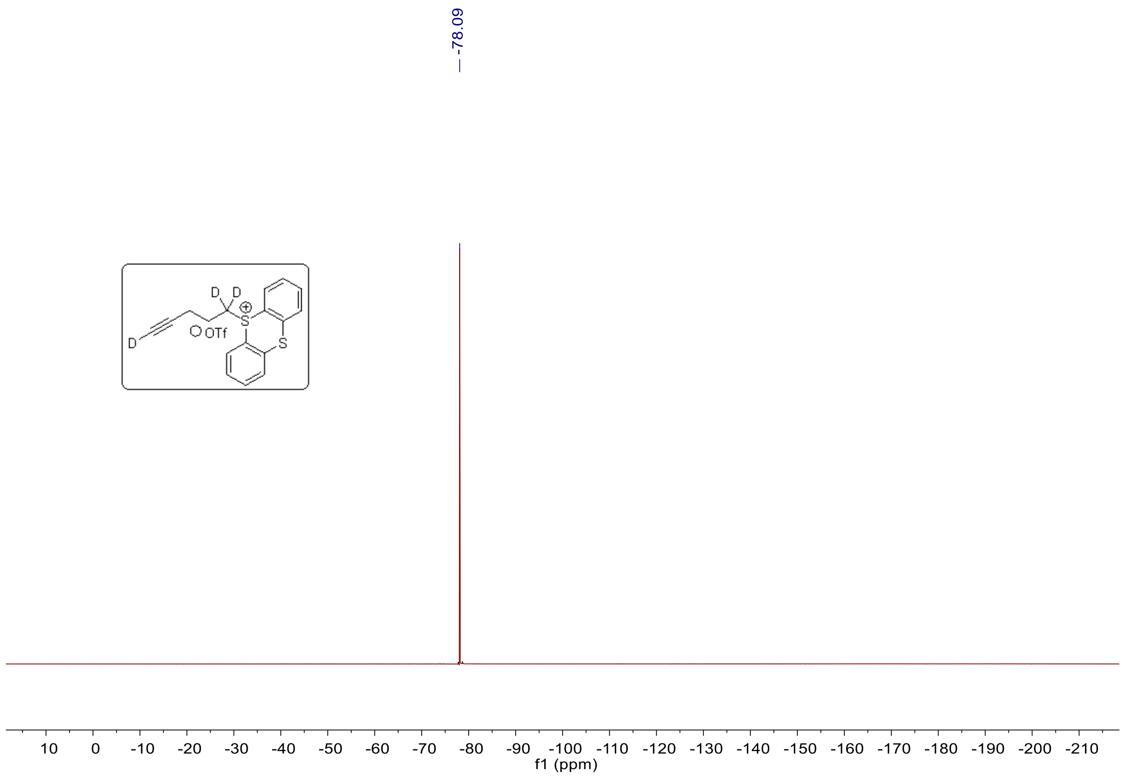

Supplementary Figure 95. ^{19}F NMR (376 MHz, $CDCl_3$) of **2n**

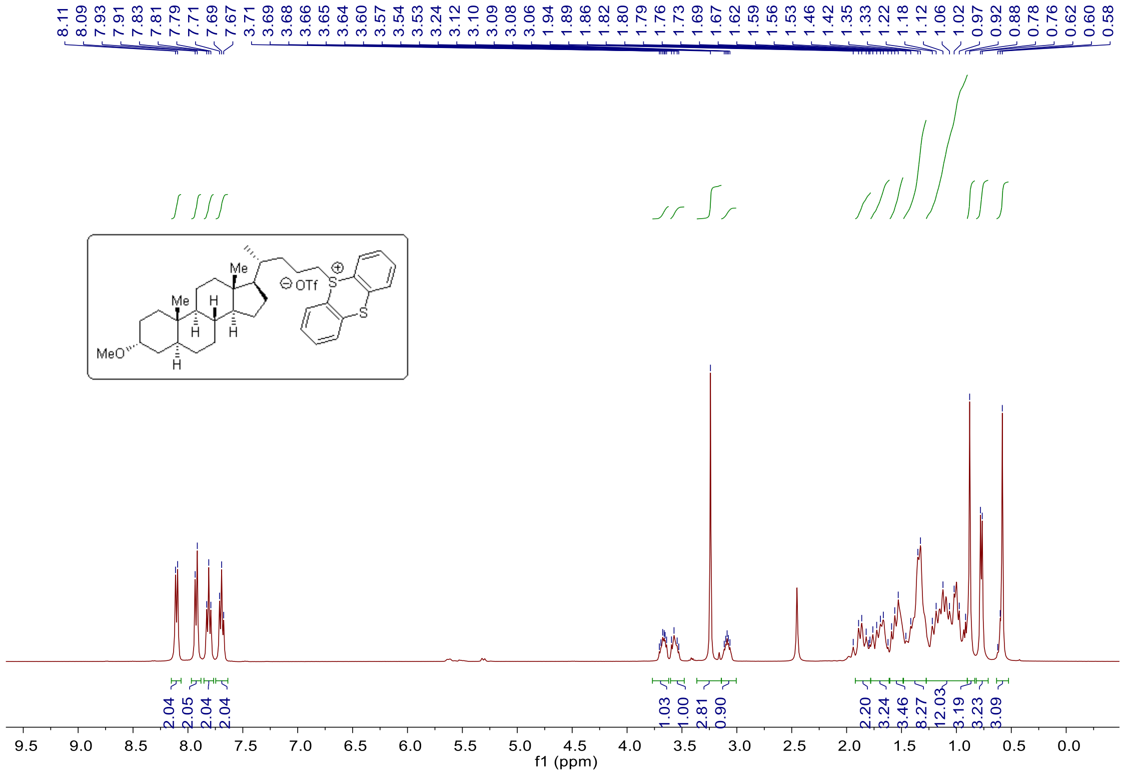

Supplementary Figure 96. ^{1}H NMR (400 MHz, CD_3CN) of **1o**

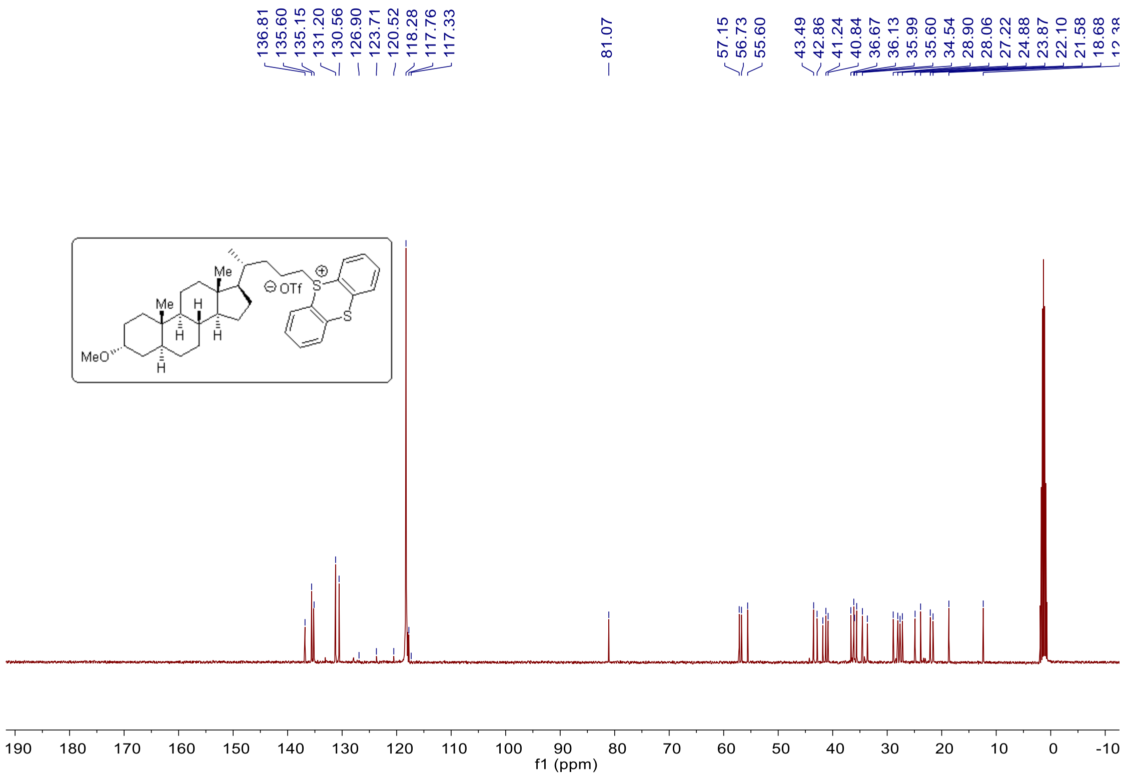

Supplementary Figure 97. ^{13}C NMR (101 MHz, CD_3CN) of **1o**

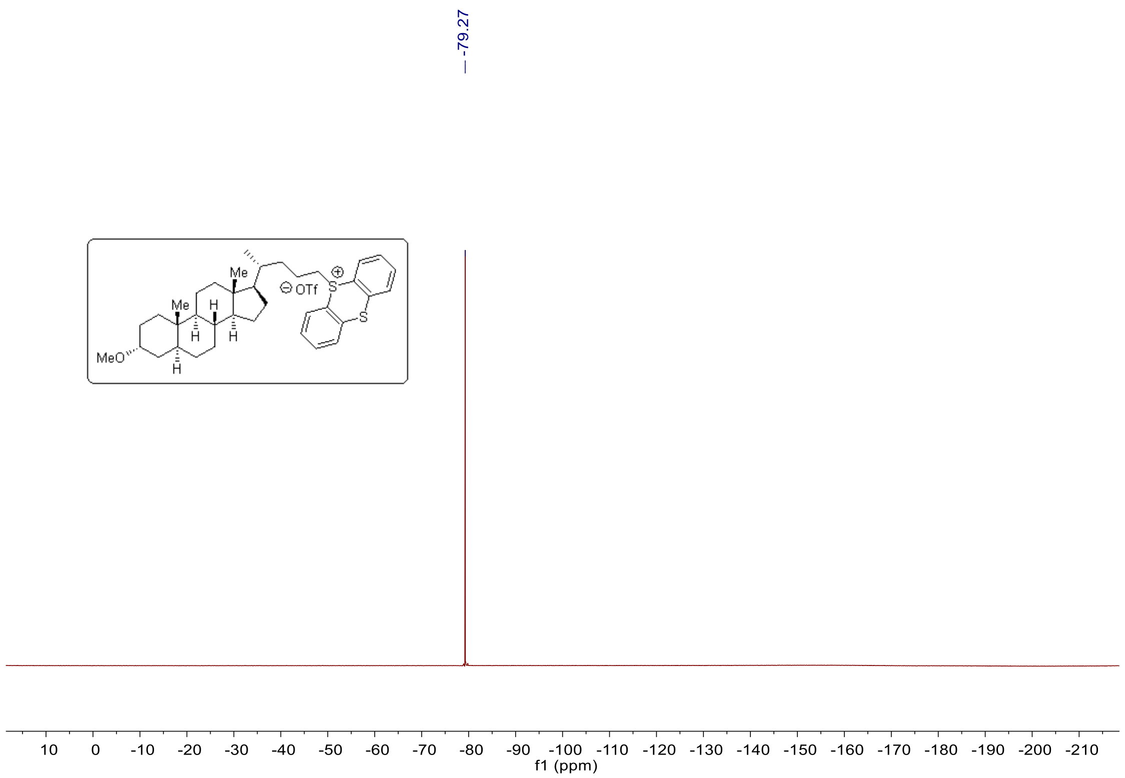

Supplementary Figure 98. ^{19}F NMR (376 MHz, CD_3CN) of **1o**

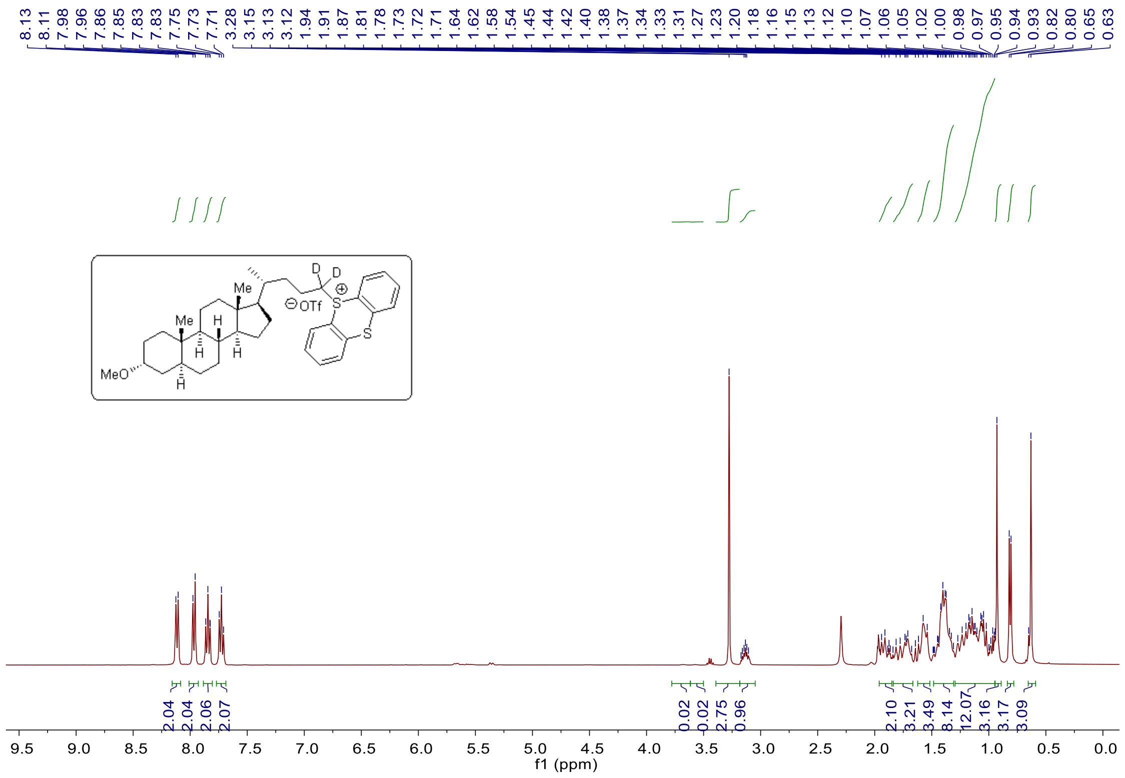

Supplementary Figure 99. ^{1}H NMR (400 MHz, CD_3CN) of **2o**

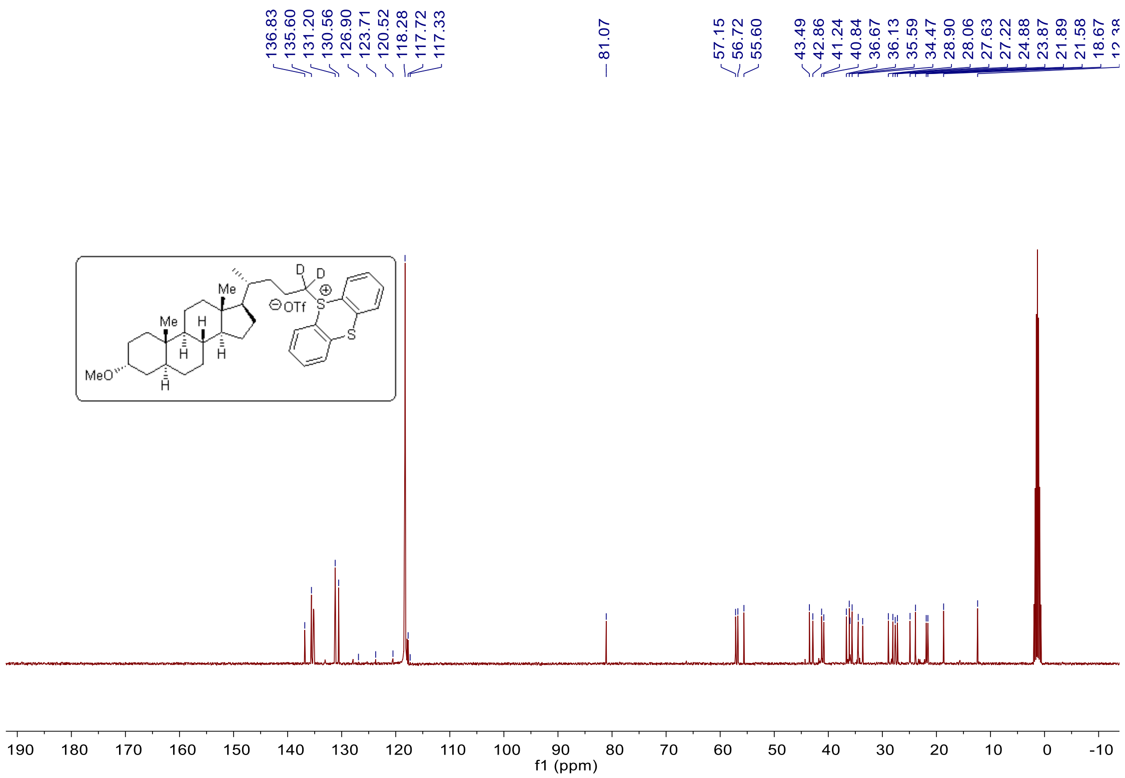

Supplementary Figure 100. ^{13}C NMR (101 MHz, CD_3CN) of **2o**

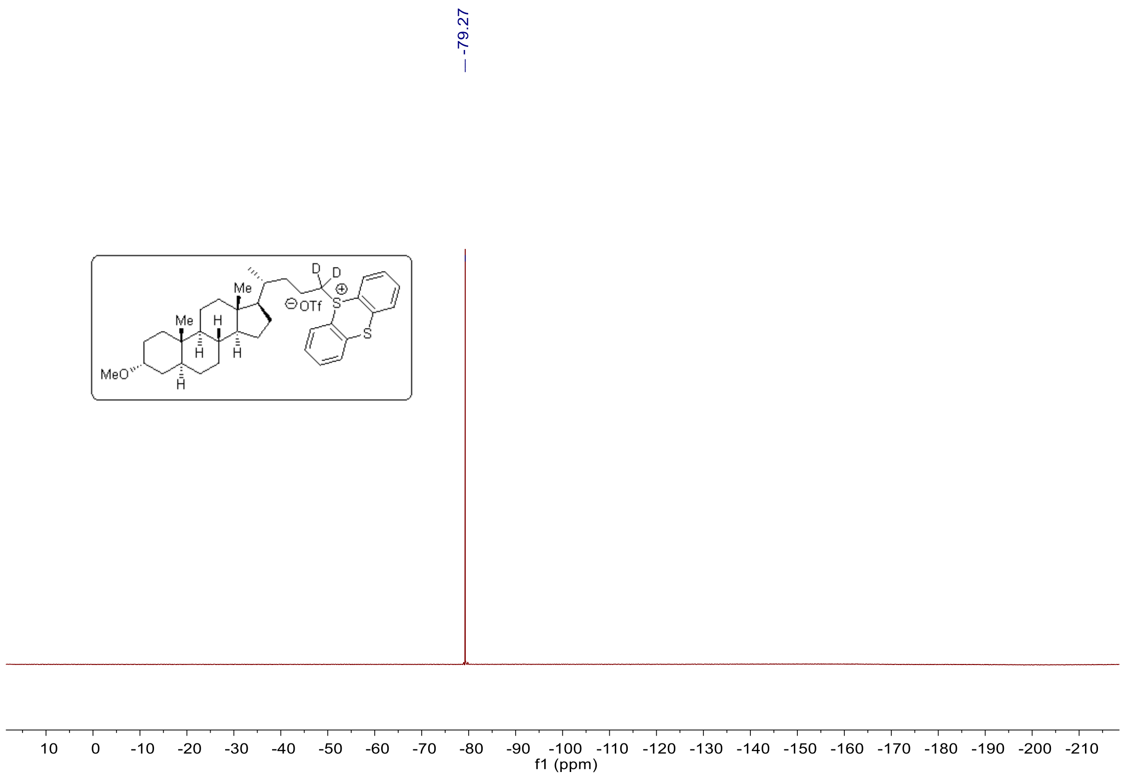

Supplementary Figure 101. ^{19}F NMR (376 MHz, CD_3CN) of **2o**

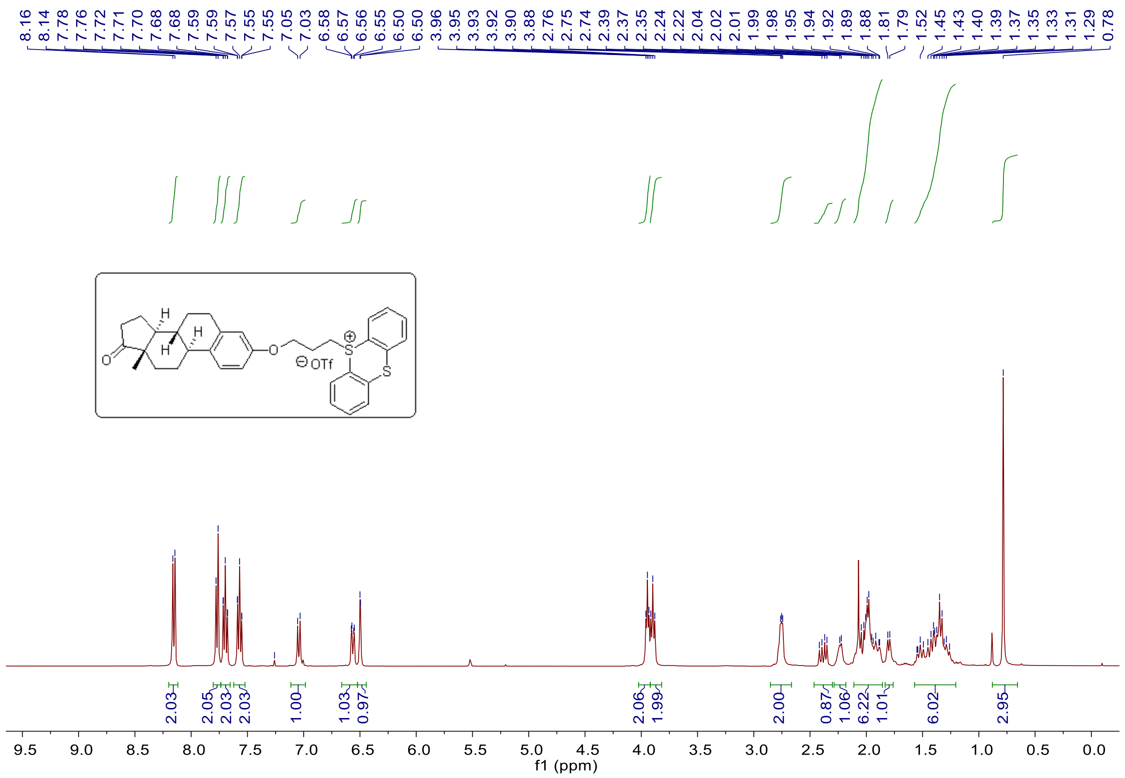

Supplementary Figure 102. ^{1}H NMR (400 MHz, $CDCl_3$) of **1p**

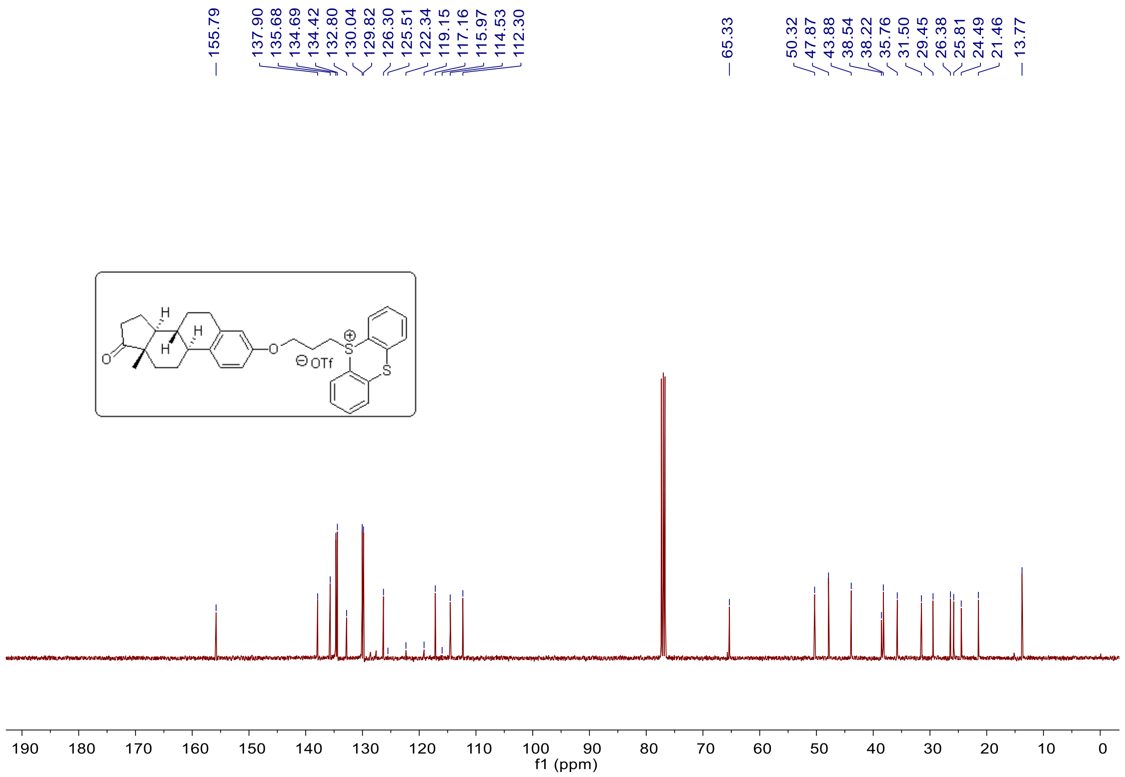

Supplementary Figure 103. ^{13}C NMR (101 MHz, $CDCl_3$) of **1p**

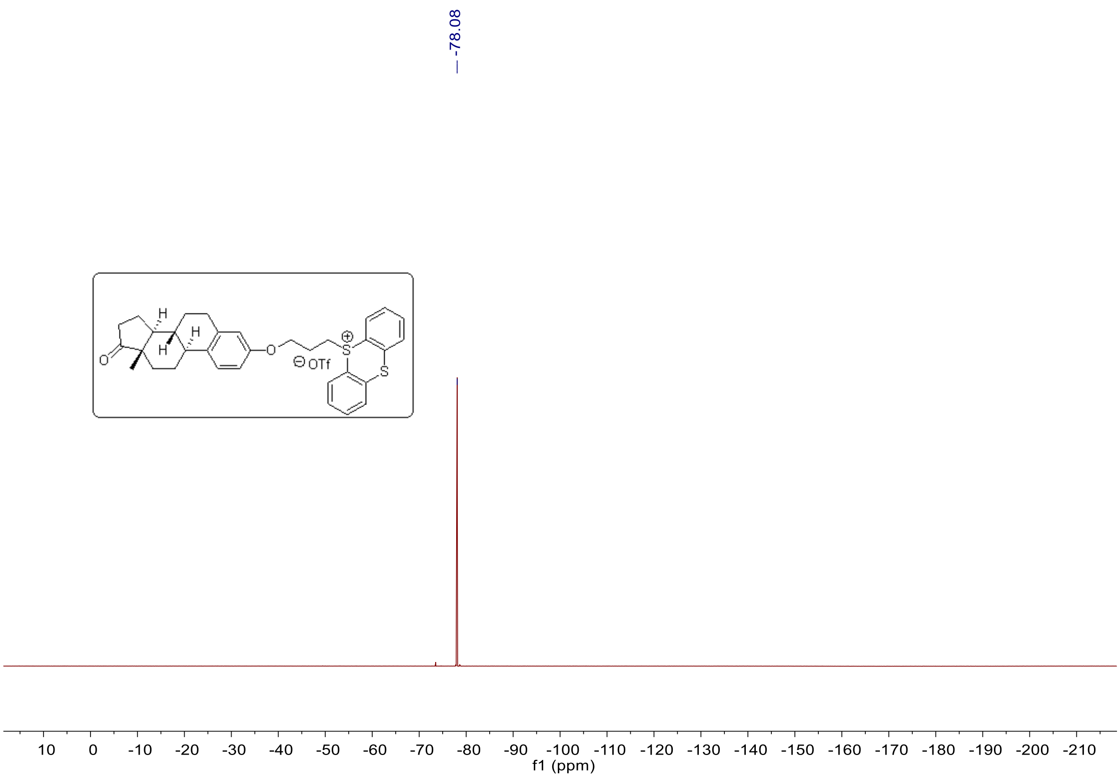

Supplementary Figure 104. ^{19}F NMR (376 MHz, CDCl$_3$) of **1p**

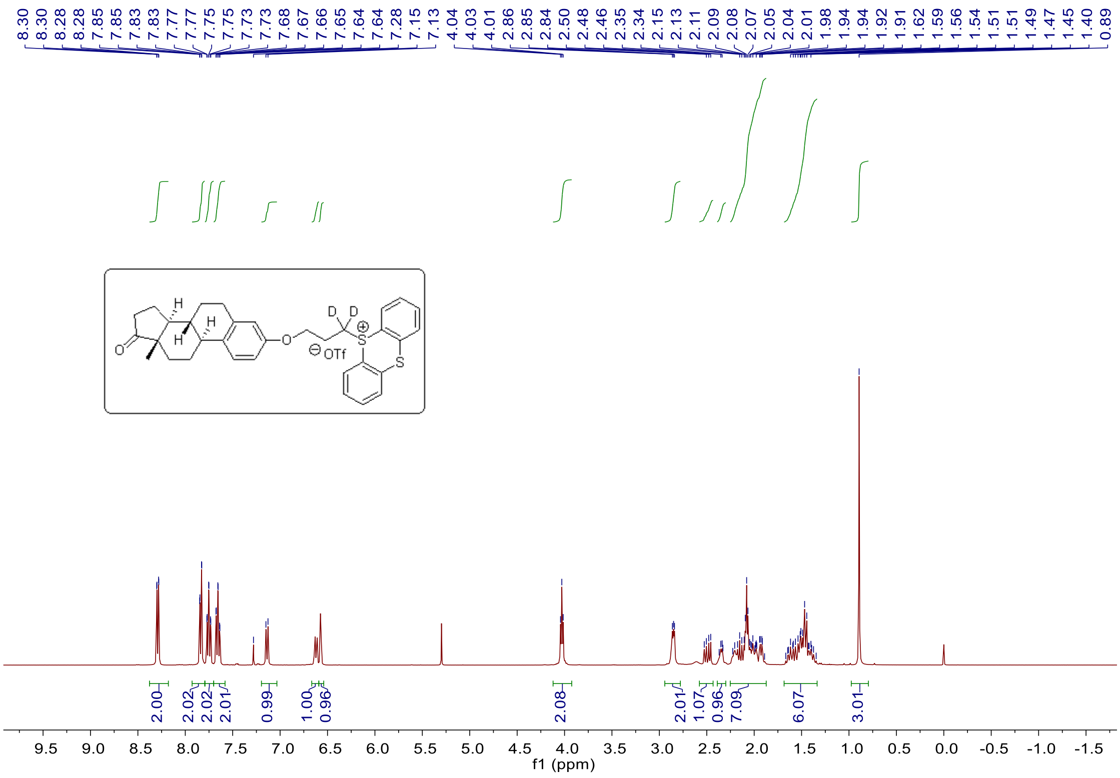

Supplementary Figure 105. ^{1}H NMR (400 MHz, CDCl$_3$) of **2p**

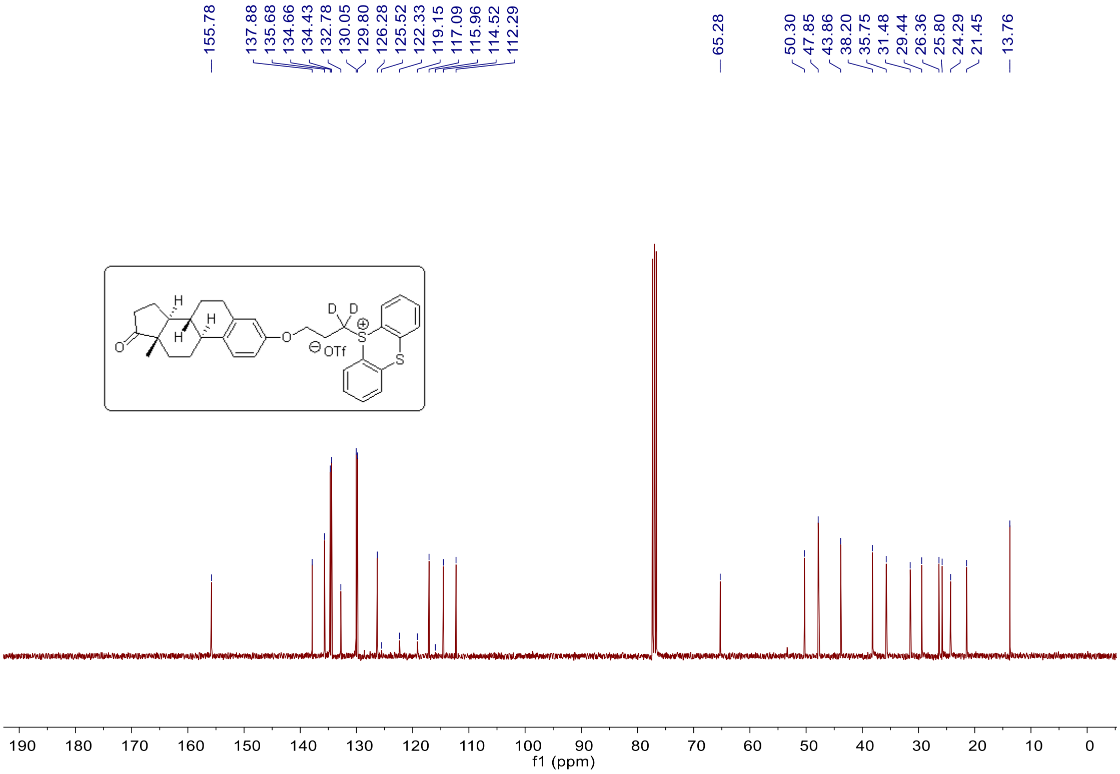

Supplementary Figure 106. ^{13}C NMR (101 MHz, $CDCl_3$) of **2p**

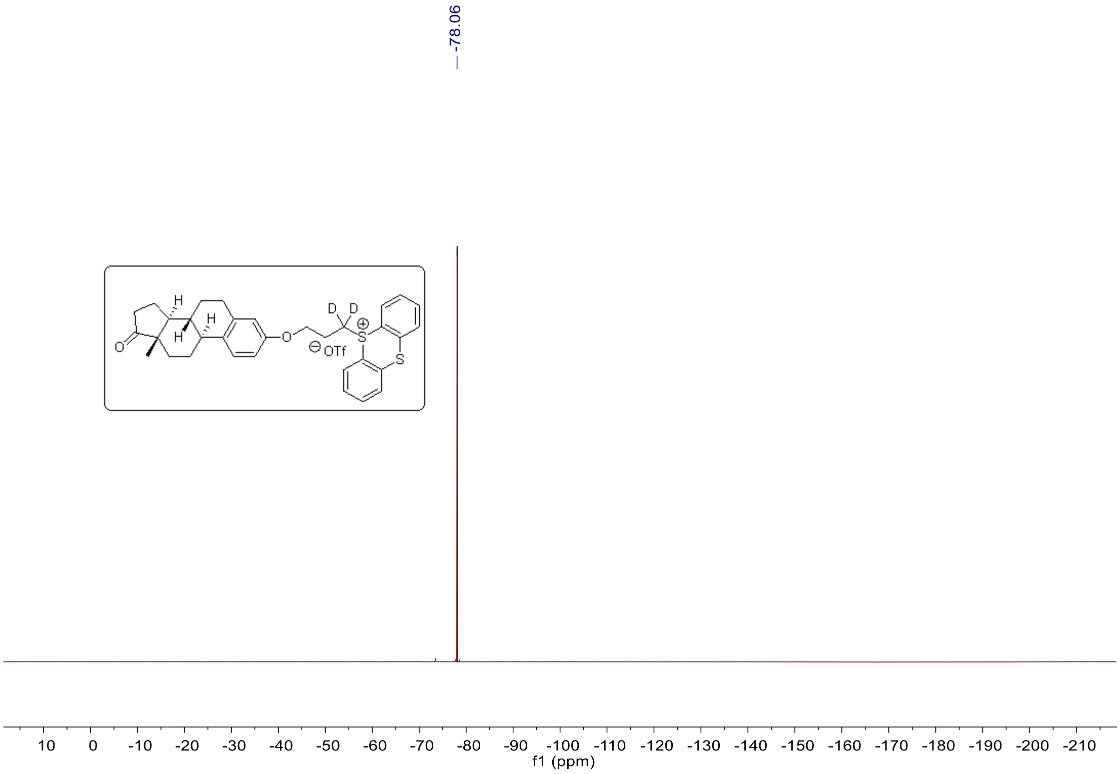

Supplementary Figure 107. ^{19}F NMR (376 MHz, $CDCl_3$) of **2p**

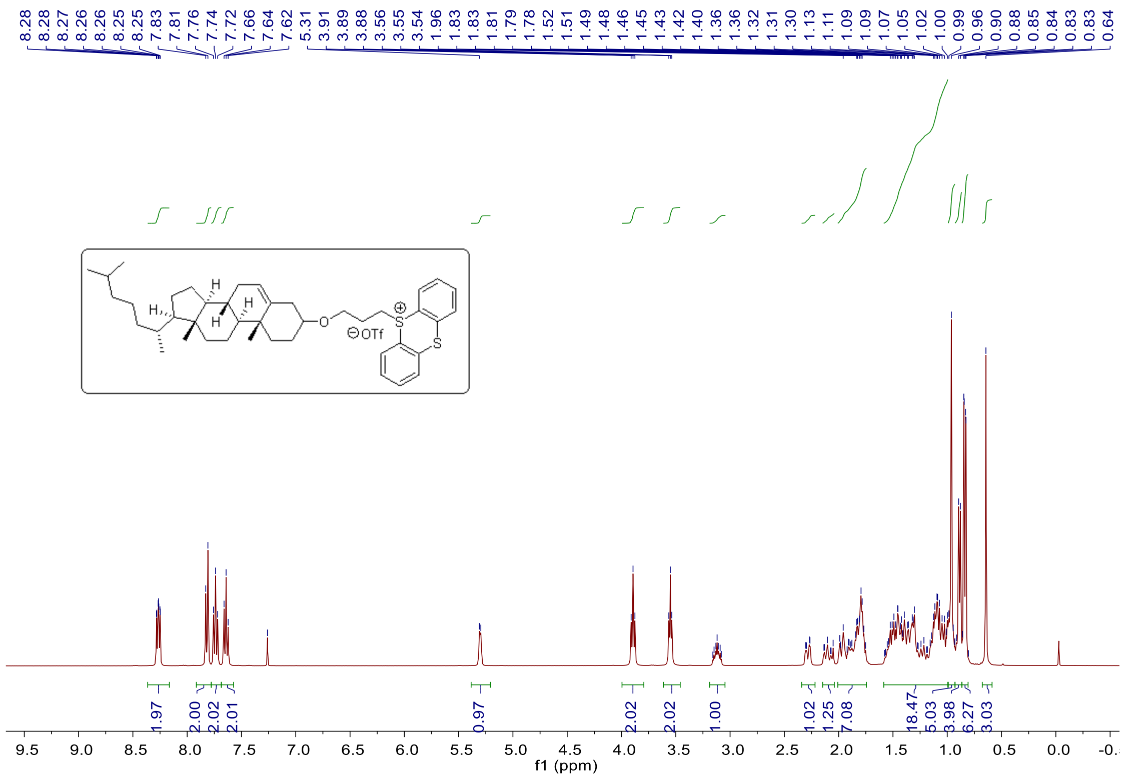

Supplementary Figure 108. ^{1}H NMR (400 MHz, $CDCl_3$) of **1q**

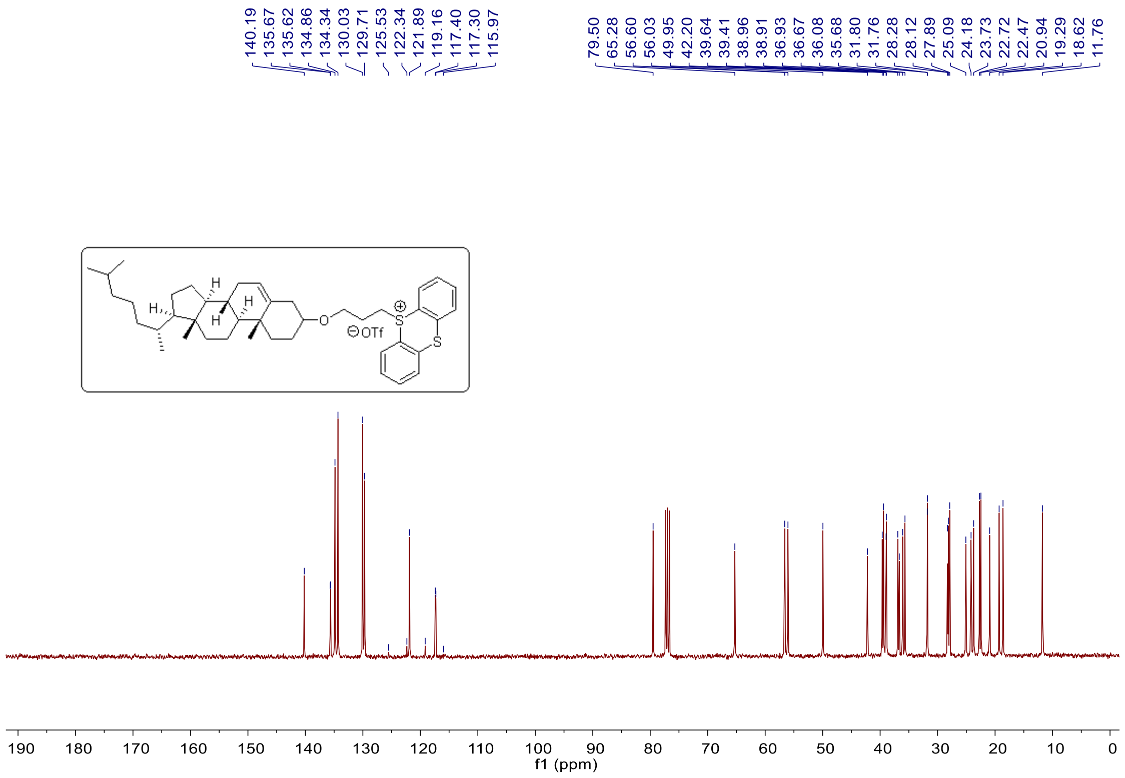

Supplementary Figure 109. ^{13}C NMR (101 MHz, $CDCl_3$) of **1q**

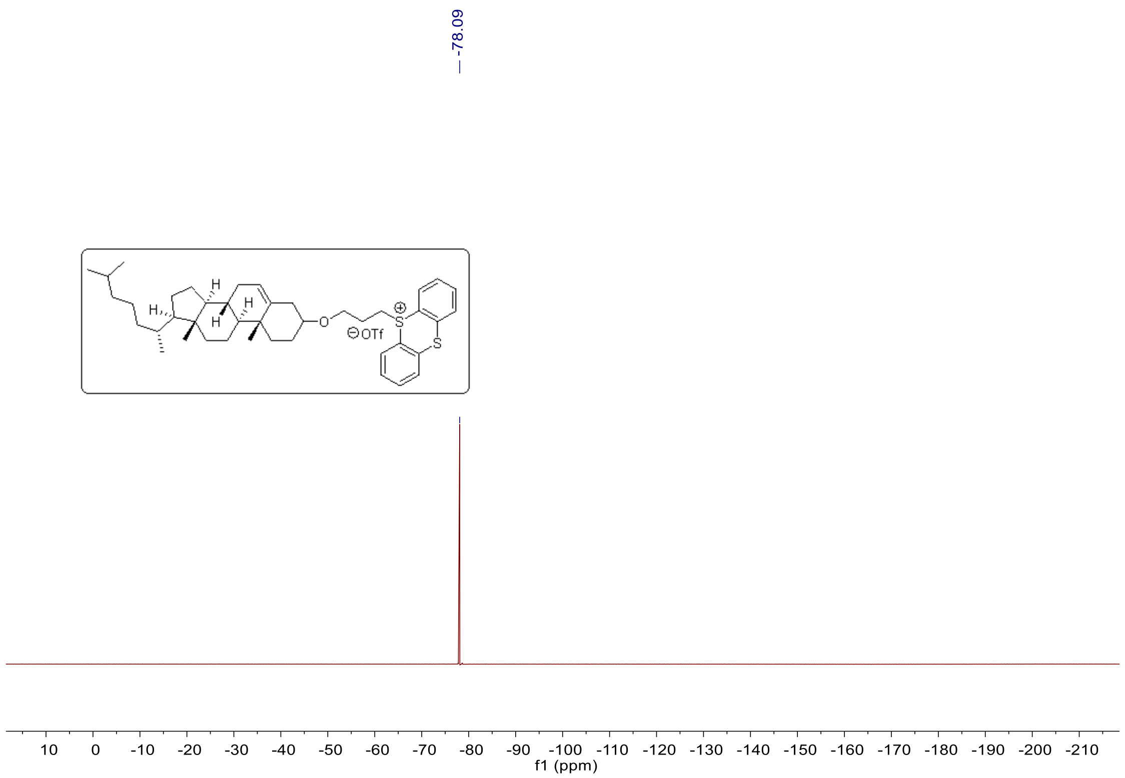

Supplementary Figure 110. ^{19}F NMR (376 MHz, $CDCl_3$) of **1q**

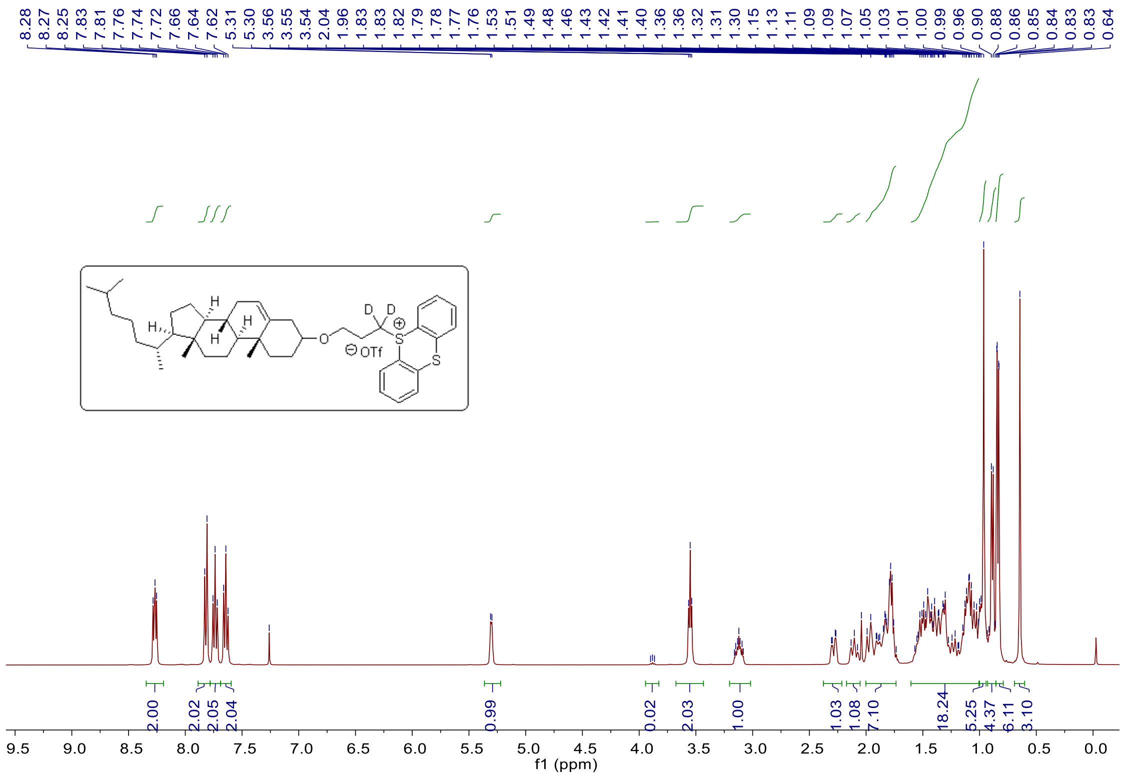

Supplementary Figure 111. ^{1}H NMR (400 MHz, $CDCl_3$) of **2q**

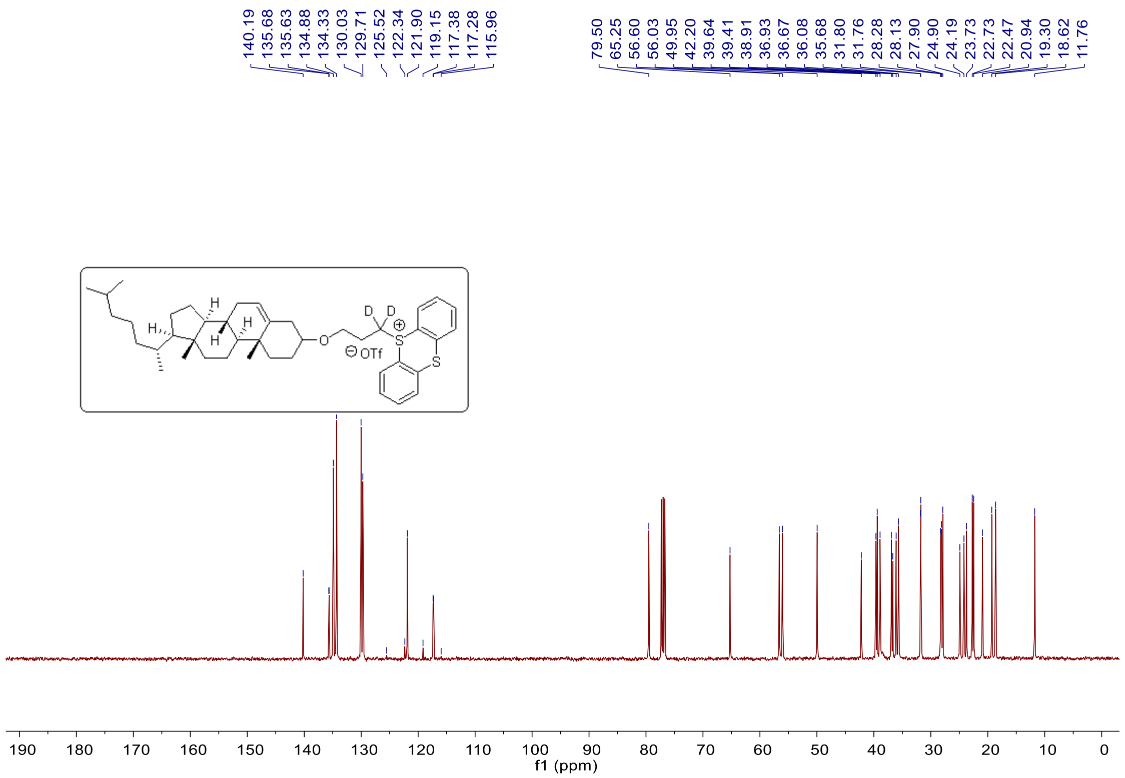

Supplementary Figure 112.^{13}C NMR (101 MHz, $CDCl_3$) of **2q**

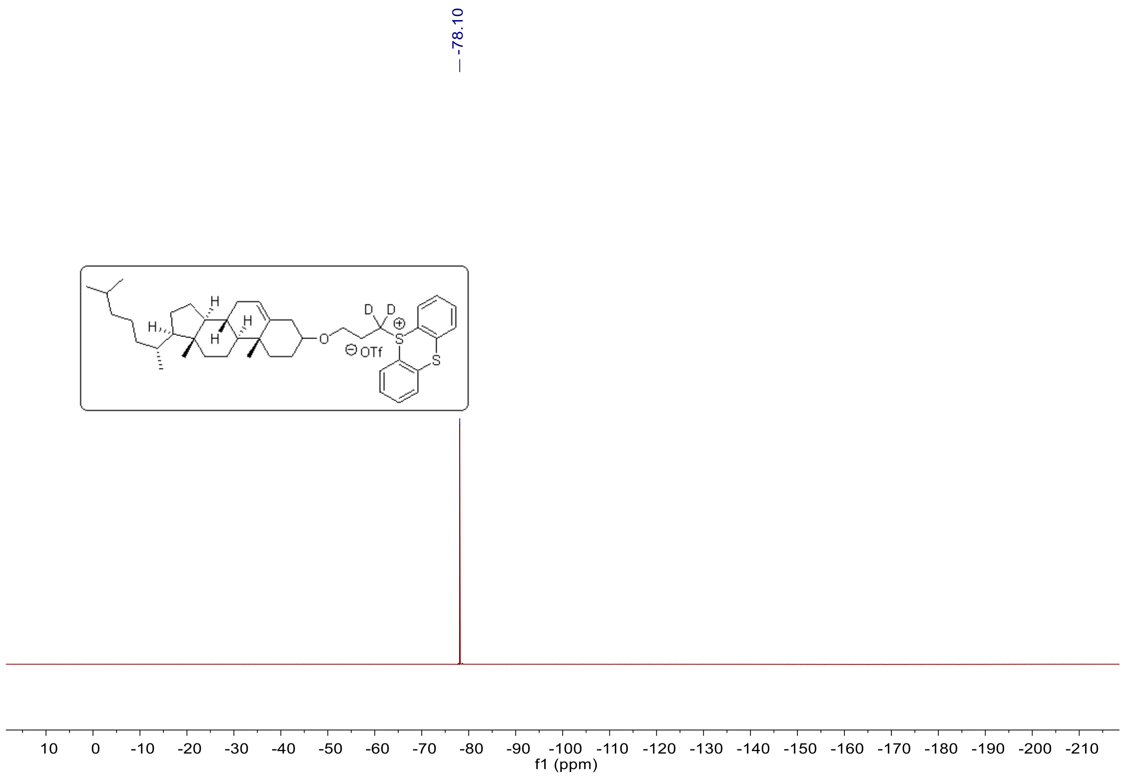

Supplementary Figure 113. ^{19}F NMR (376 MHz, $CDCl_3$) of **2q**

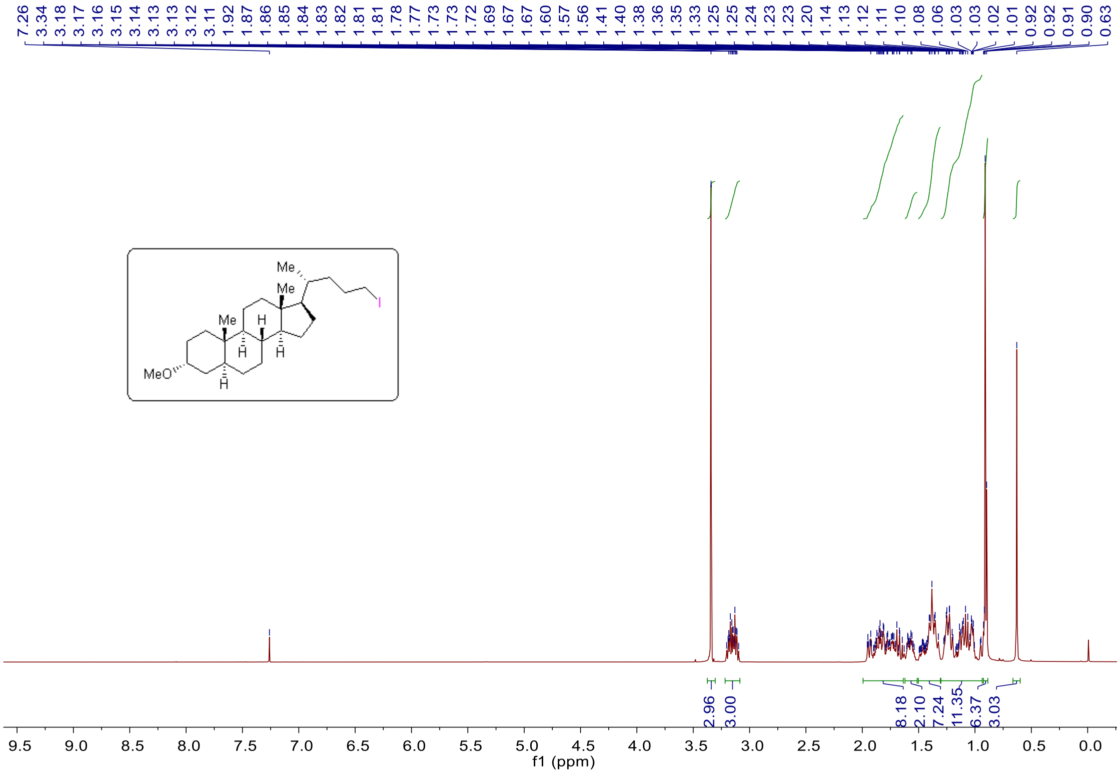

Supplementary Figure 114. ^{1}H NMR (500 MHz, $CDCl_3$) of **3oa'**

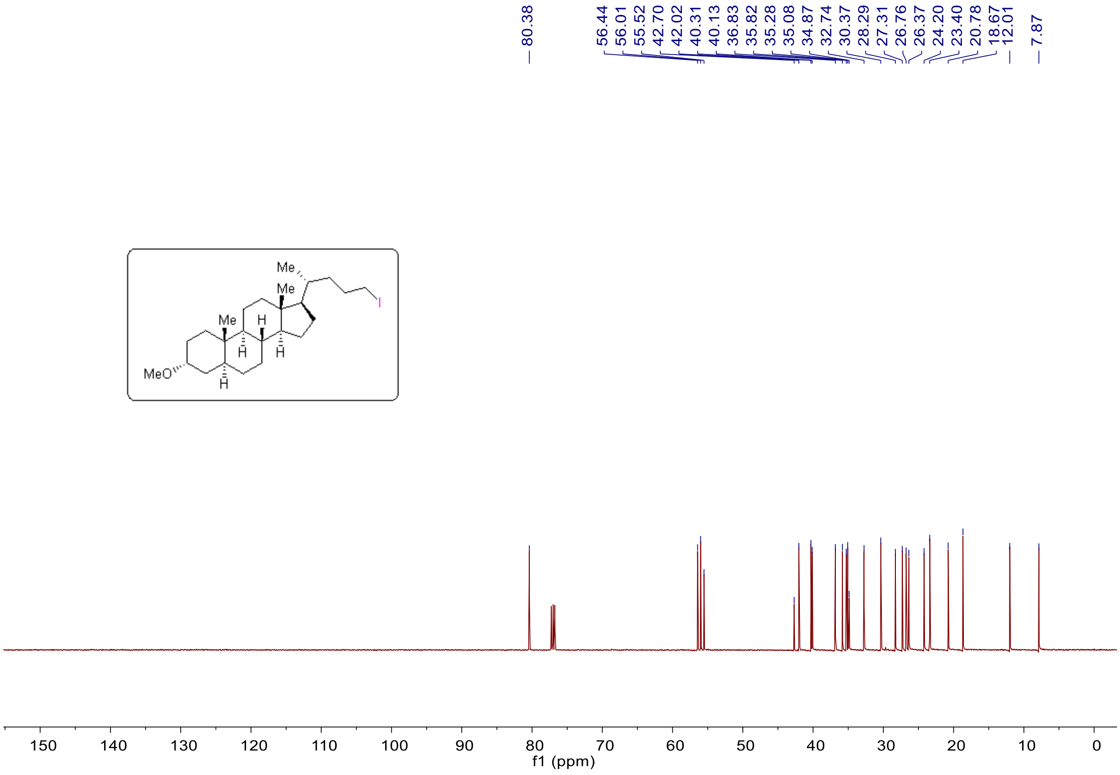

Supplementary Figure 115. ^{13}C NMR (126 MHz, $CDCl_3$) of **3oa'**

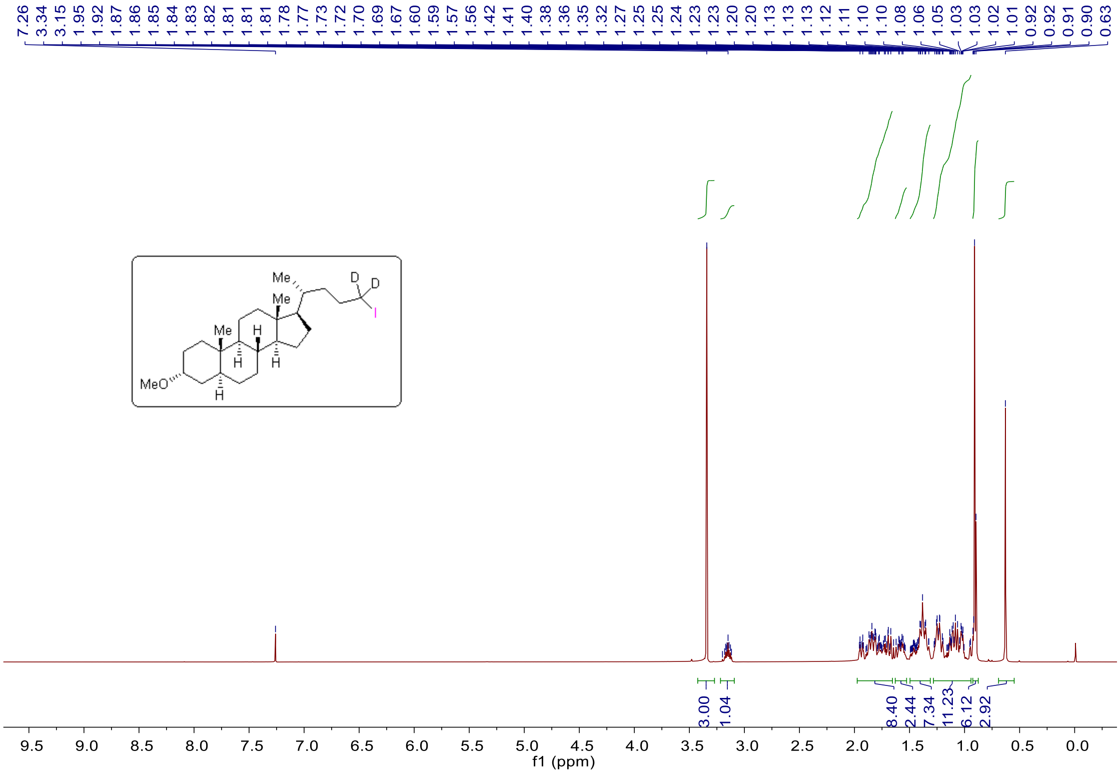

Supplementary Figure 116. ^{1}H NMR (500 MHz, $CDCl_3$) of **3oa**

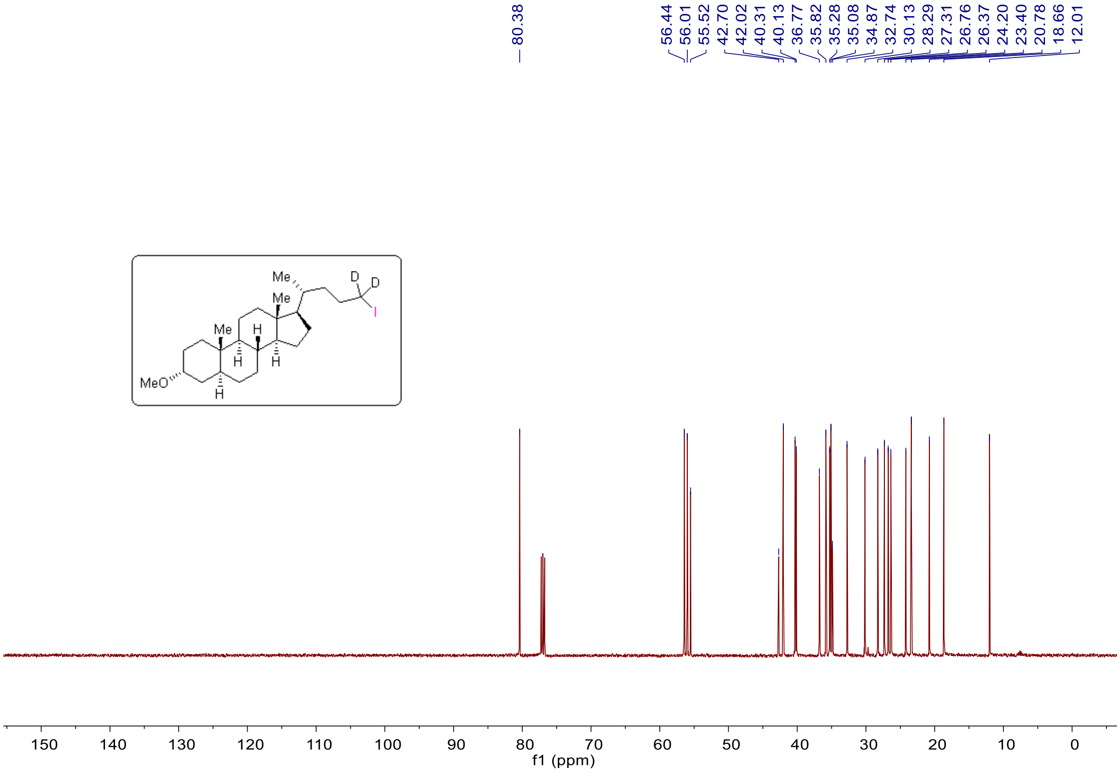

Supplementary Figure 117. ^{13}C NMR (126 MHz, $CDCl_3$) of **3oa**

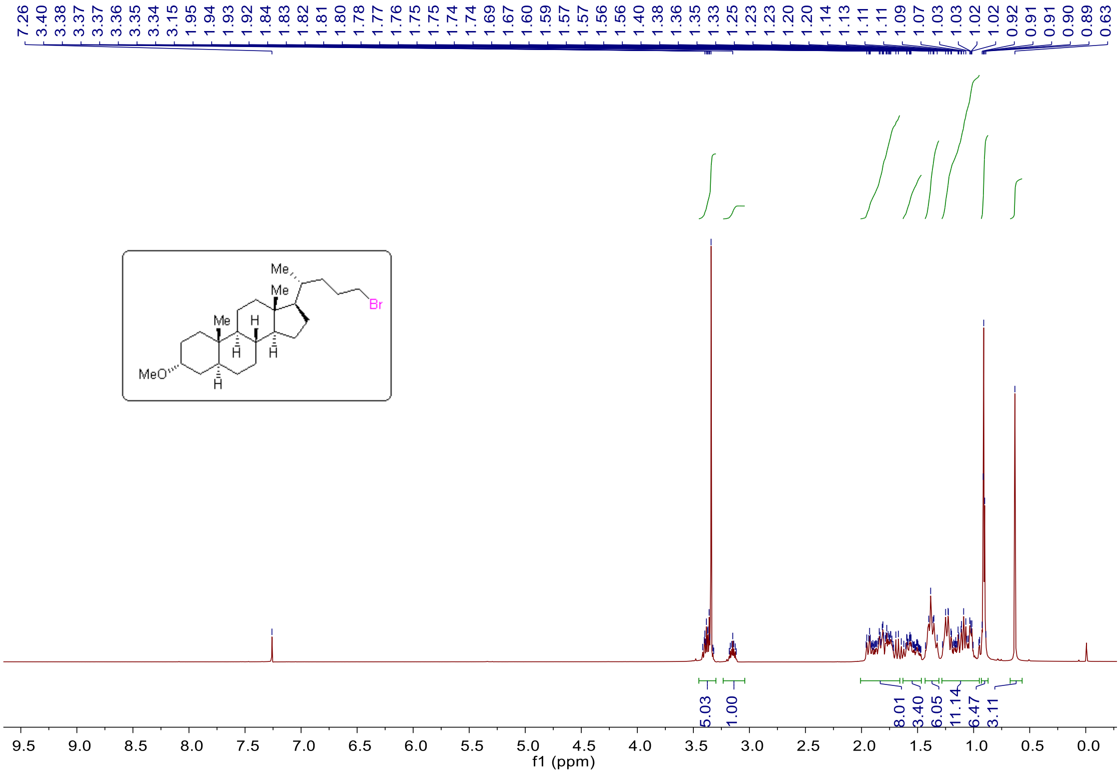

Supplementary Figure 118. ^{1}H NMR (500 MHz, $CDCl_3$) of **3ob'**

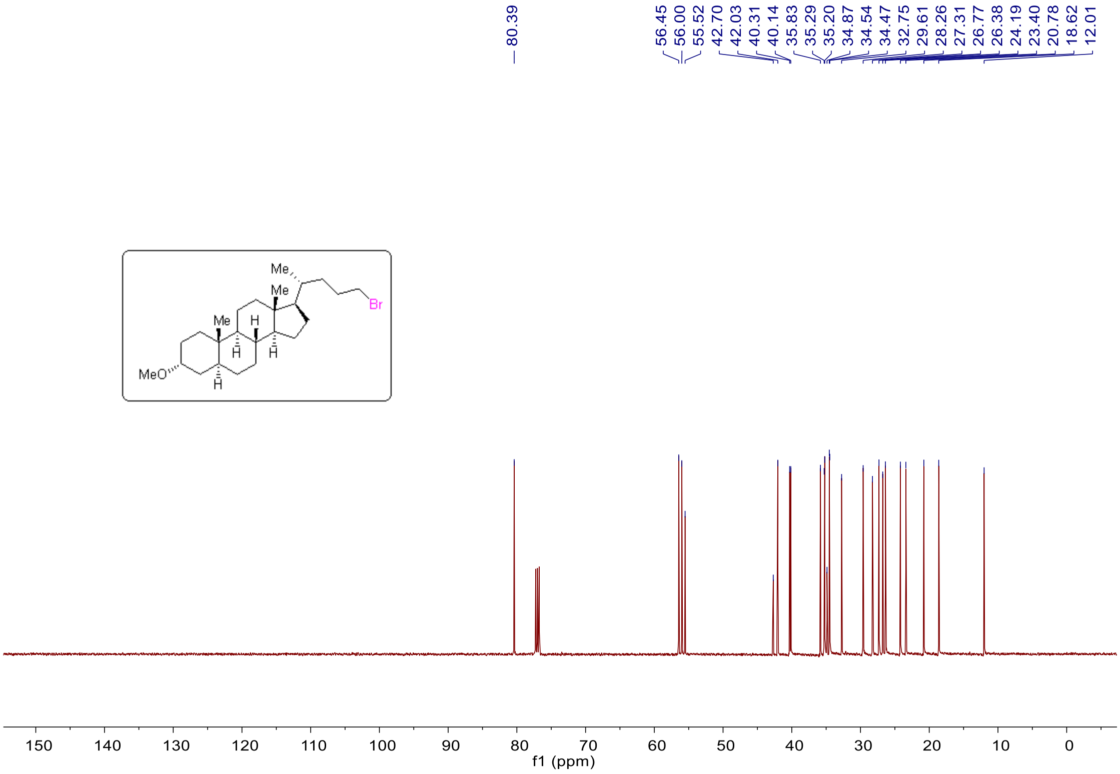

Supplementary Figure 119. ^{13}C NMR (126 MHz, $CDCl_3$) of **3ob'**

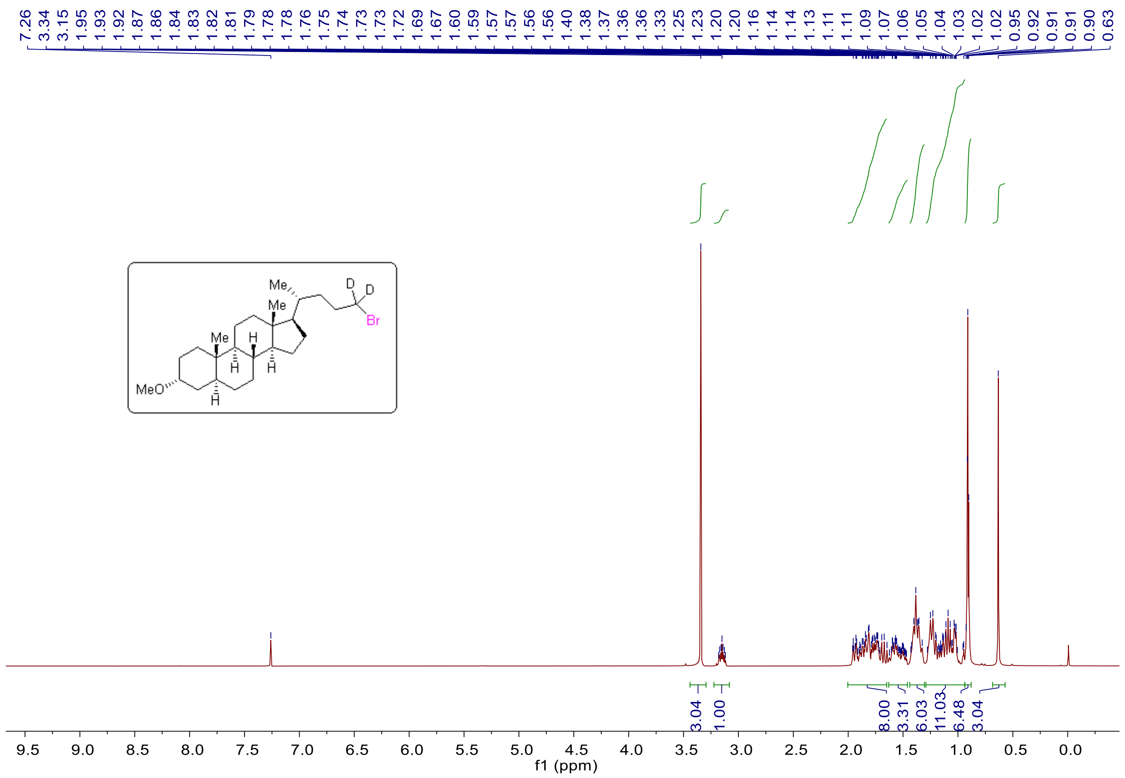

Supplementary Figure 120. ^{1}H NMR (500 MHz, $CDCl_3$) of **3ob**

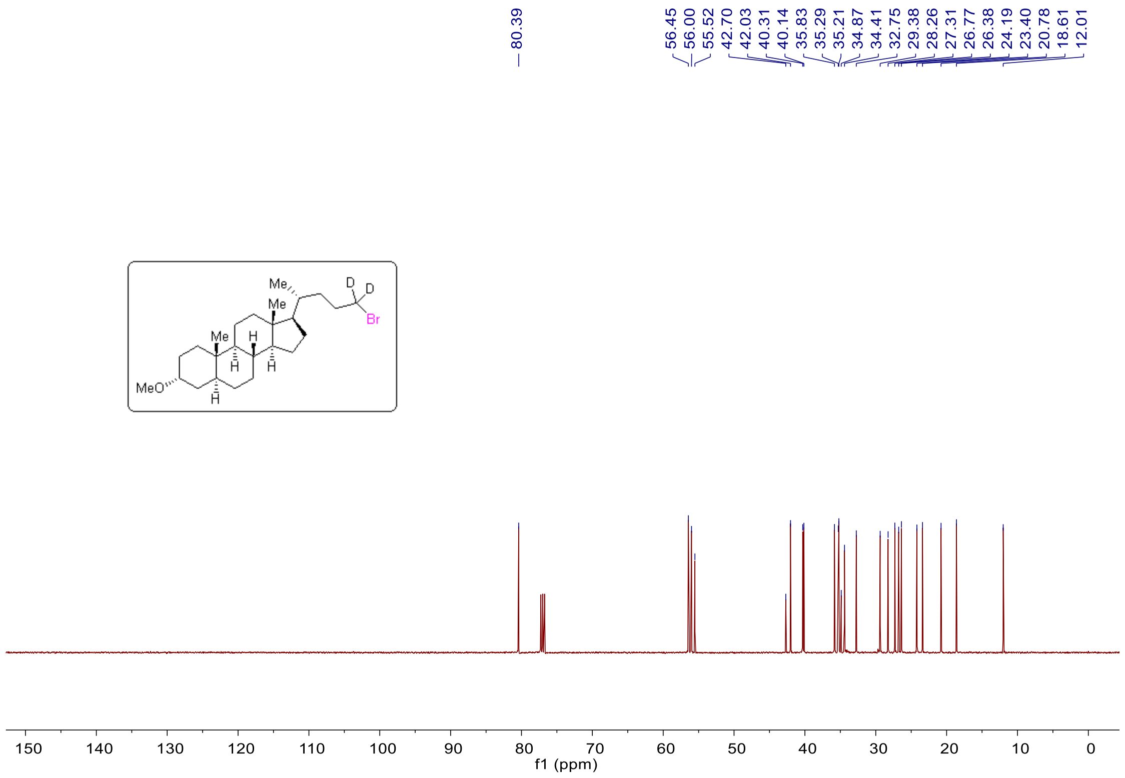

Supplementary Figure 121. ^{13}C NMR (126 MHz, $CDCl_3$) of **3ob**

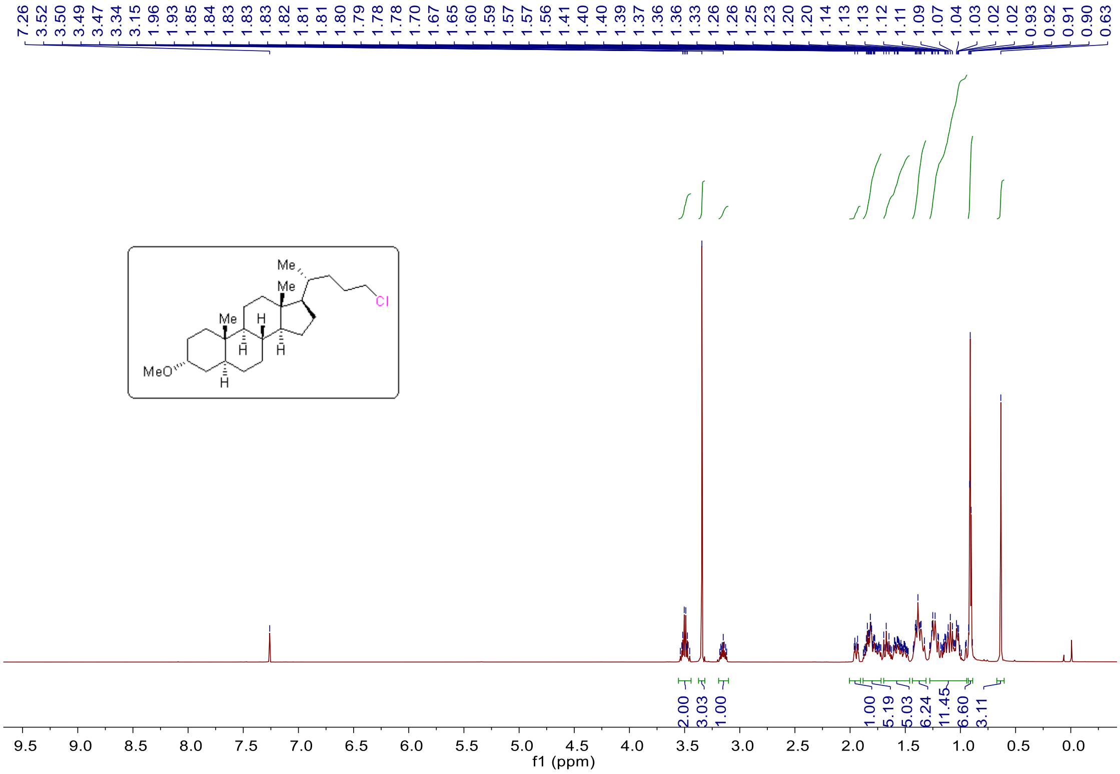

Supplementary Figure 122. ^{1}H NMR (500 MHz, $CDCl_3$) of **3oc'**

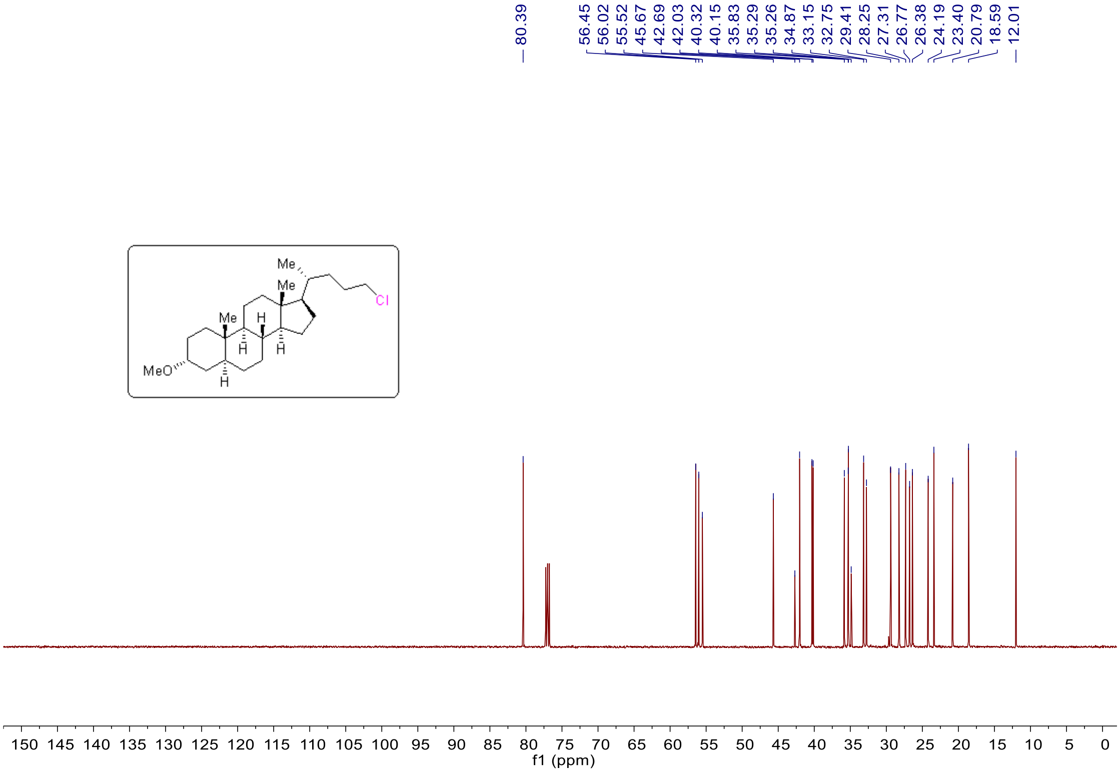

Supplementary Figure 123. ^{13}C NMR (126 MHz, $CDCl_3$) of **3oc'**

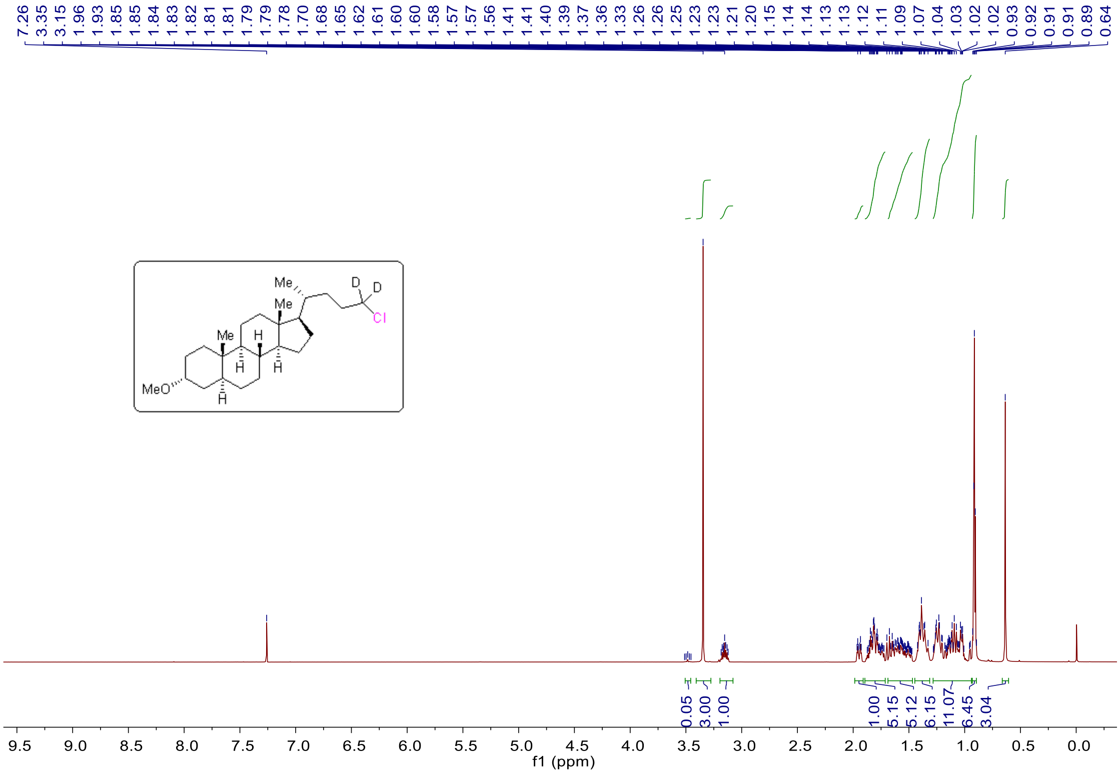

Supplementary Figure 124. ^{1}H NMR (500 MHz, $CDCl_3$) of **3oc**

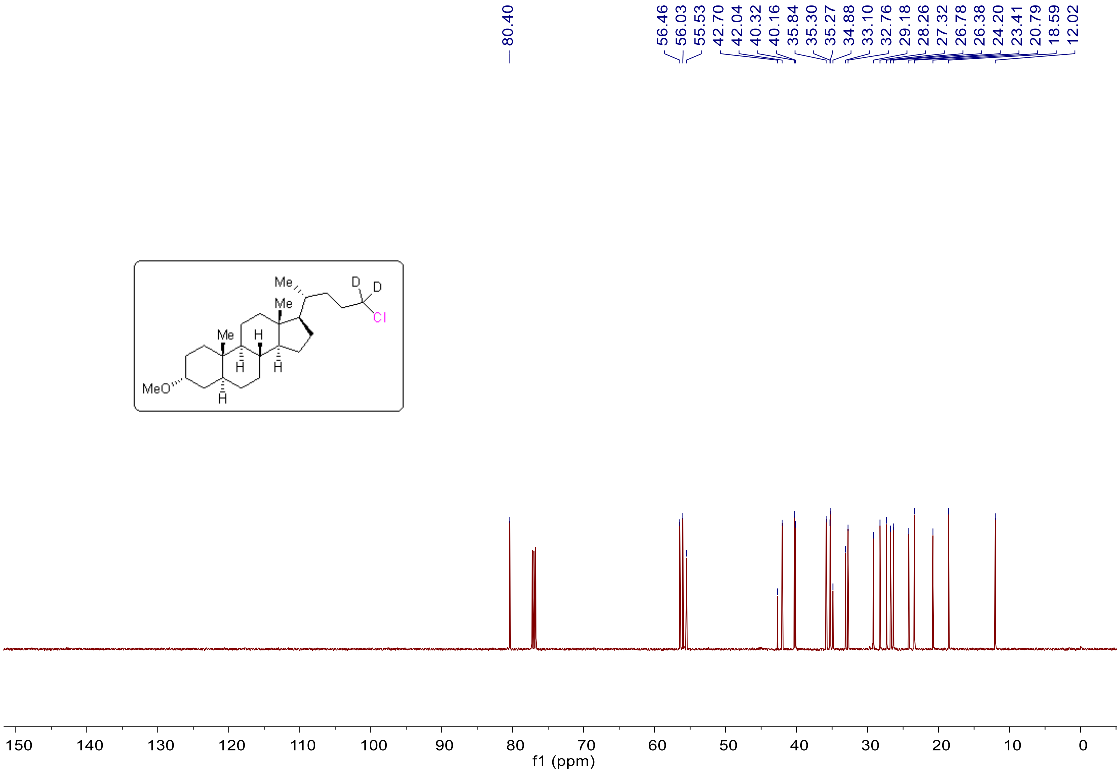

Supplementary Figure 125. ^{13}C NMR (126 MHz, $CDCl_3$) of **3oc**

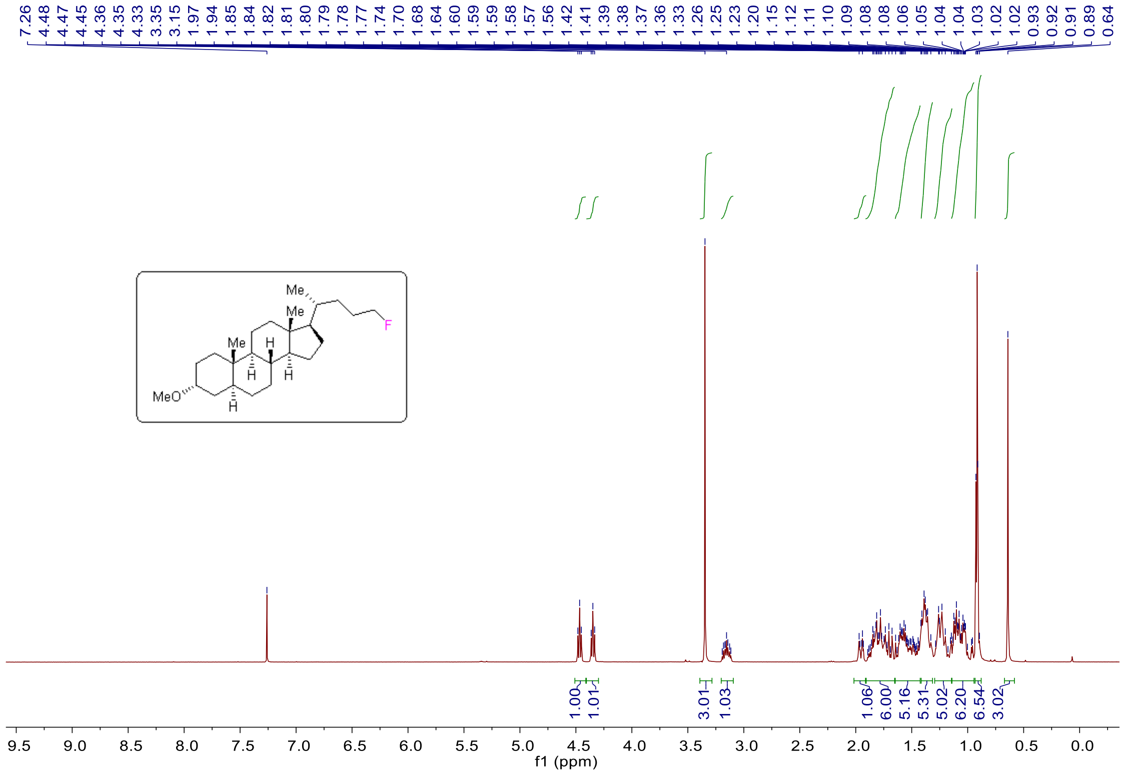

Supplementary Figure 126. ^{1}H NMR (400 MHz, $CDCl_3$) of **3od'**

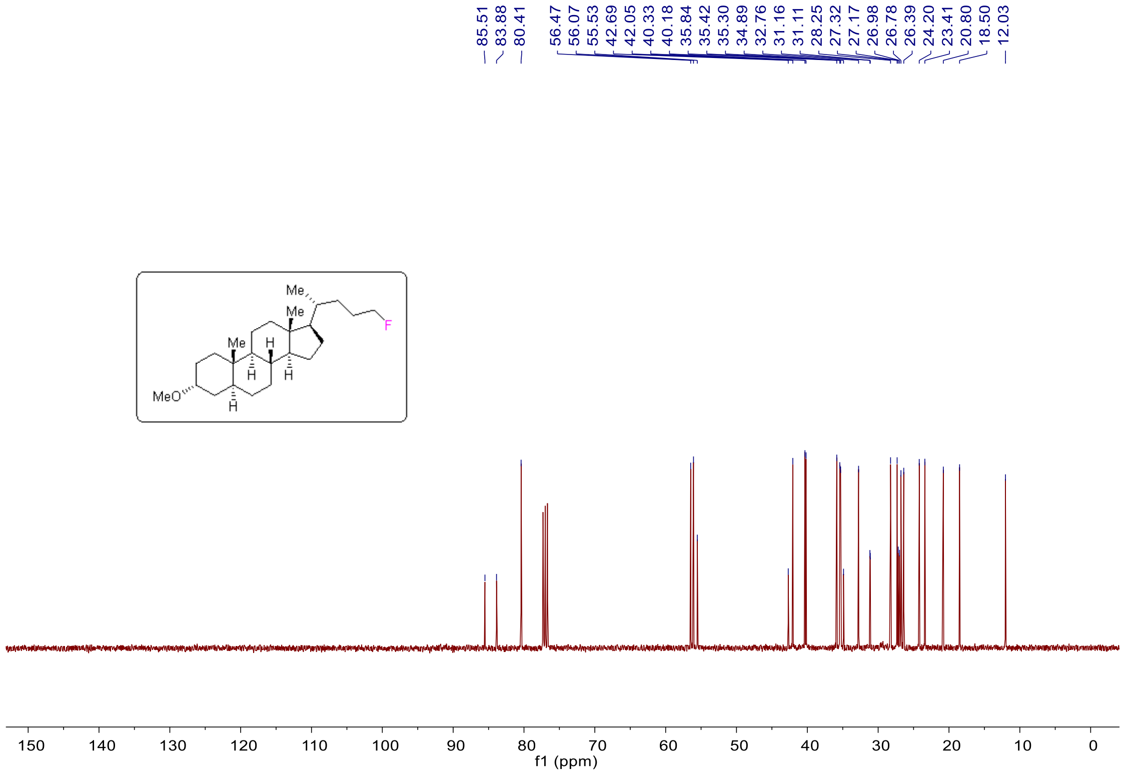

Supplementary Figure 127. ^{13}C NMR (101 MHz, $CDCl_3$) of **3od'**

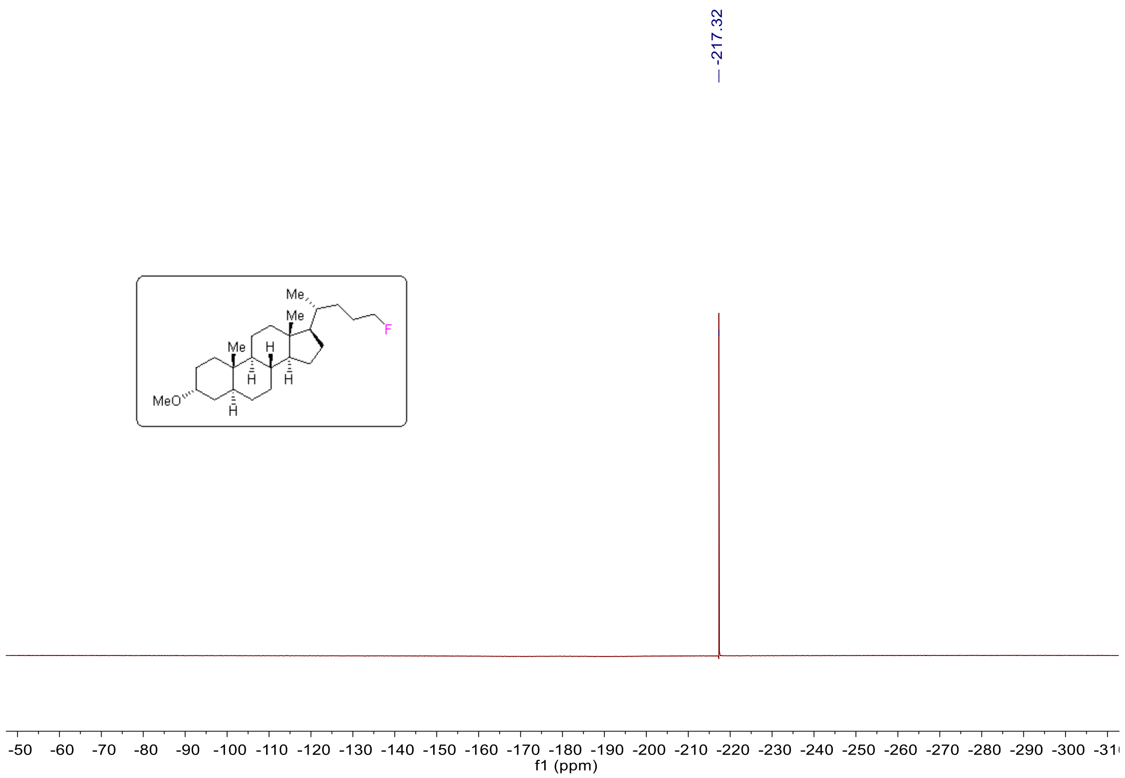

Supplementary Figure 128. ^{19}F NMR (471 MHz, CDCl$_3$) of **3od'**

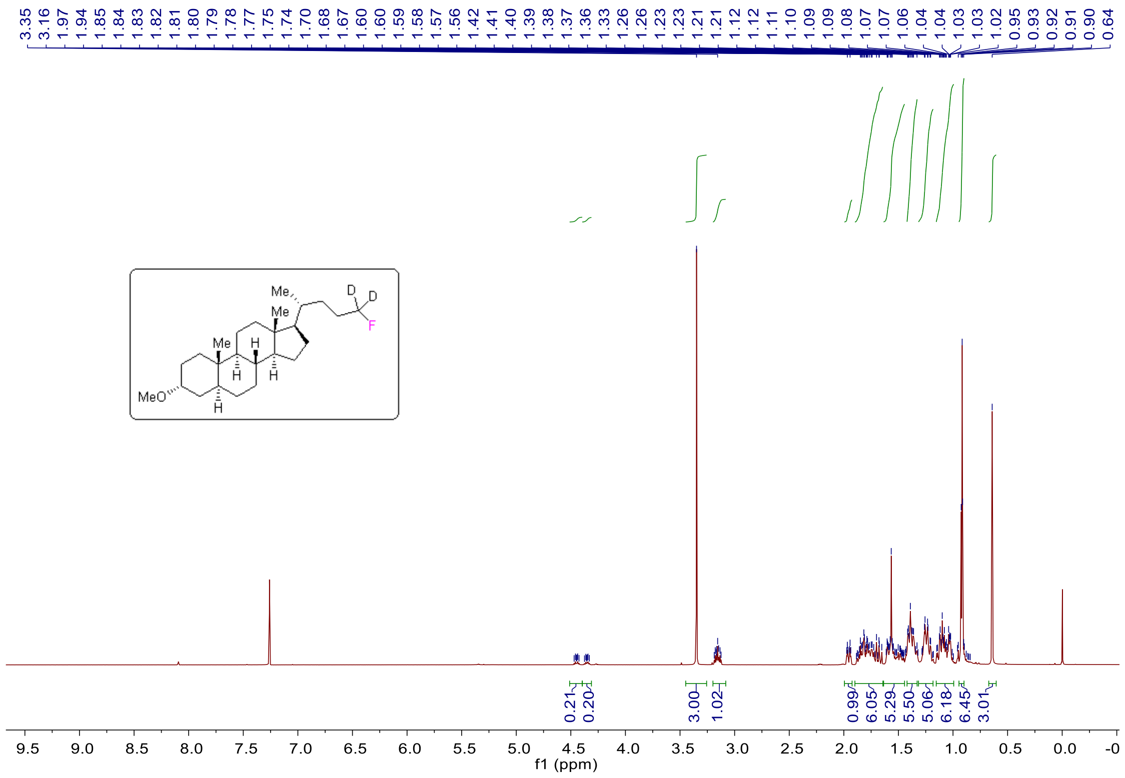

Supplementary Figure 129. ^{1}H NMR (500 MHz, CDCl$_3$) of **3od**

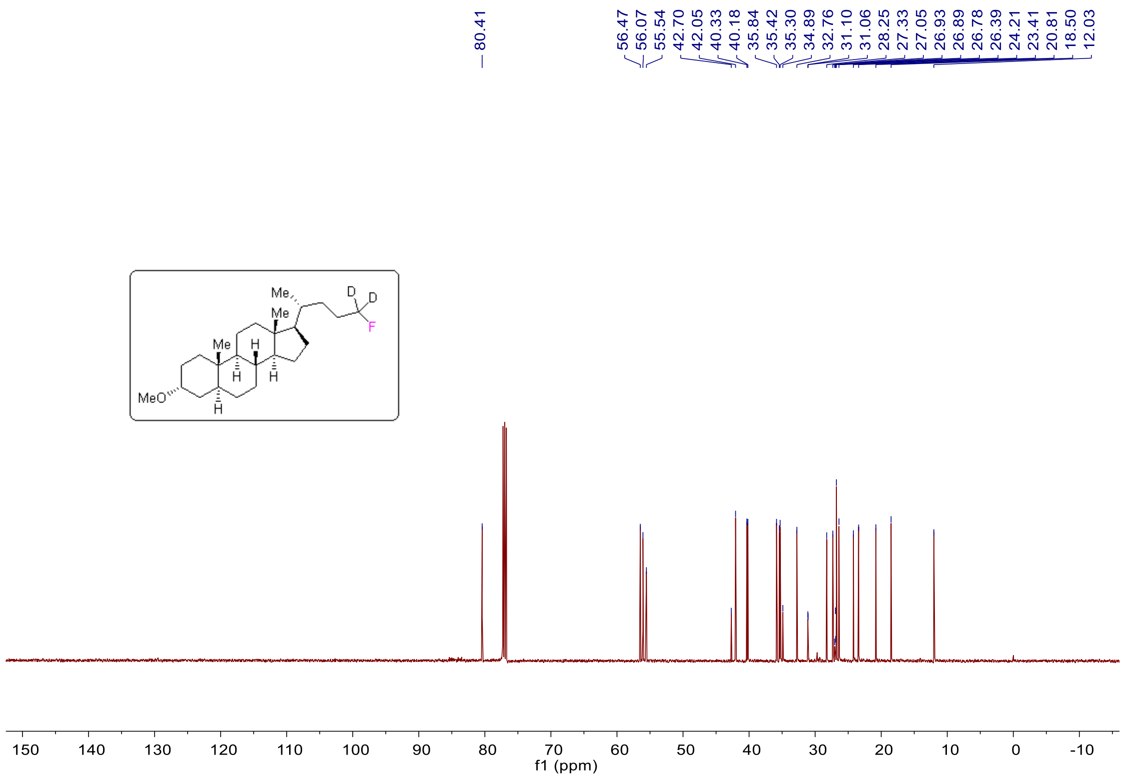

Supplementary Figure 130. ^{13}C NMR (126 MHz, $CDCl_3$) of **3od**

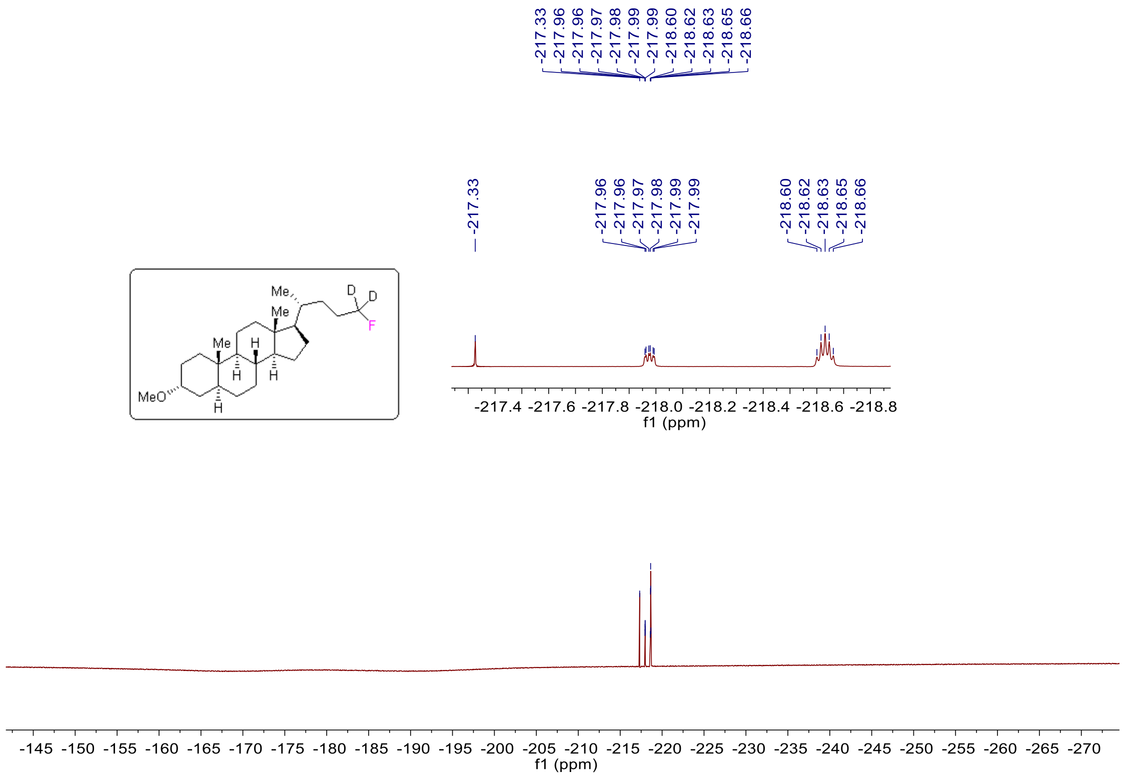

Supplementary Figure 131. ^{19}F NMR (471 MHz, $CDCl_3$) of **3od**

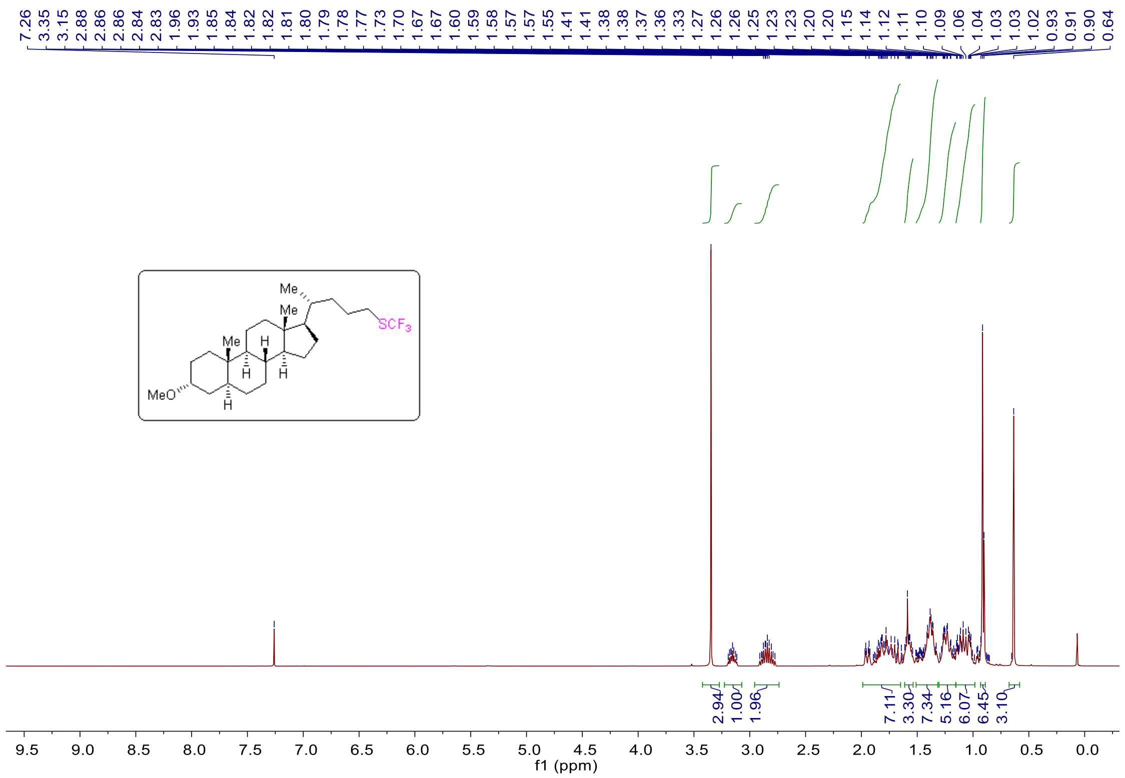

Supplementary Figure 132. ^{1}H NMR (400 MHz, $CDCl_3$) of **3oe'**

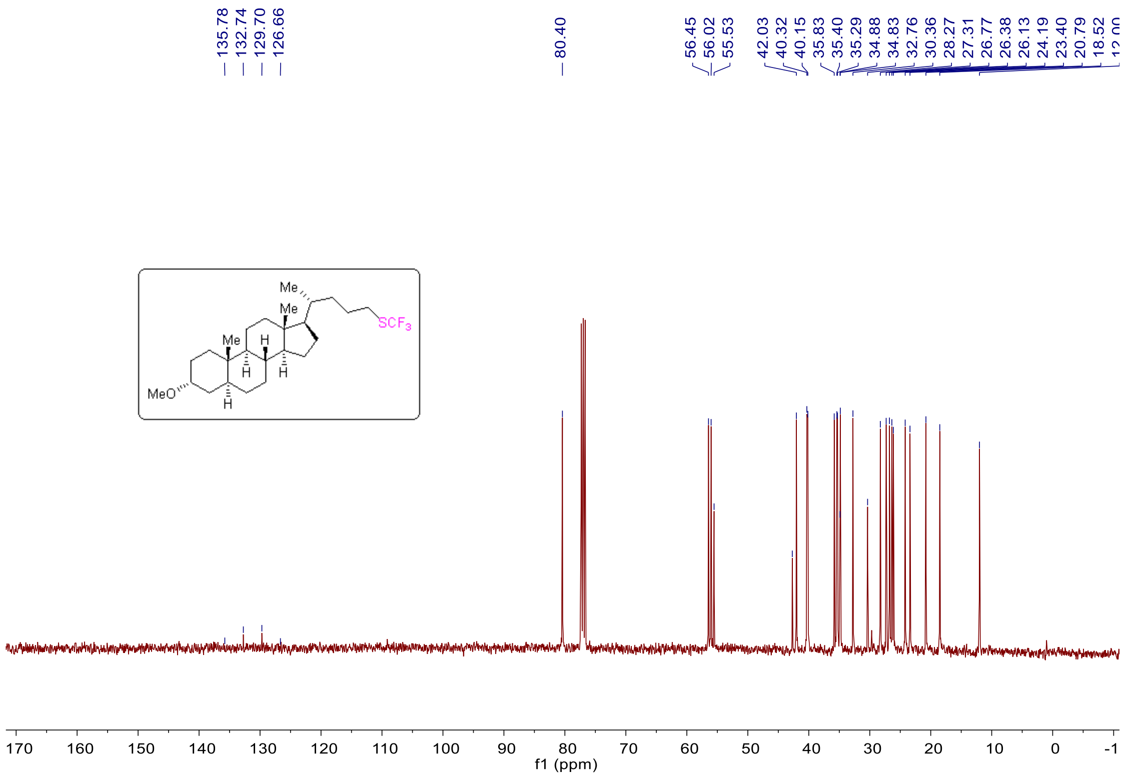

Supplementary Figure 133. ^{13}C NMR (101 MHz, $CDCl_3$) of **3oe'**

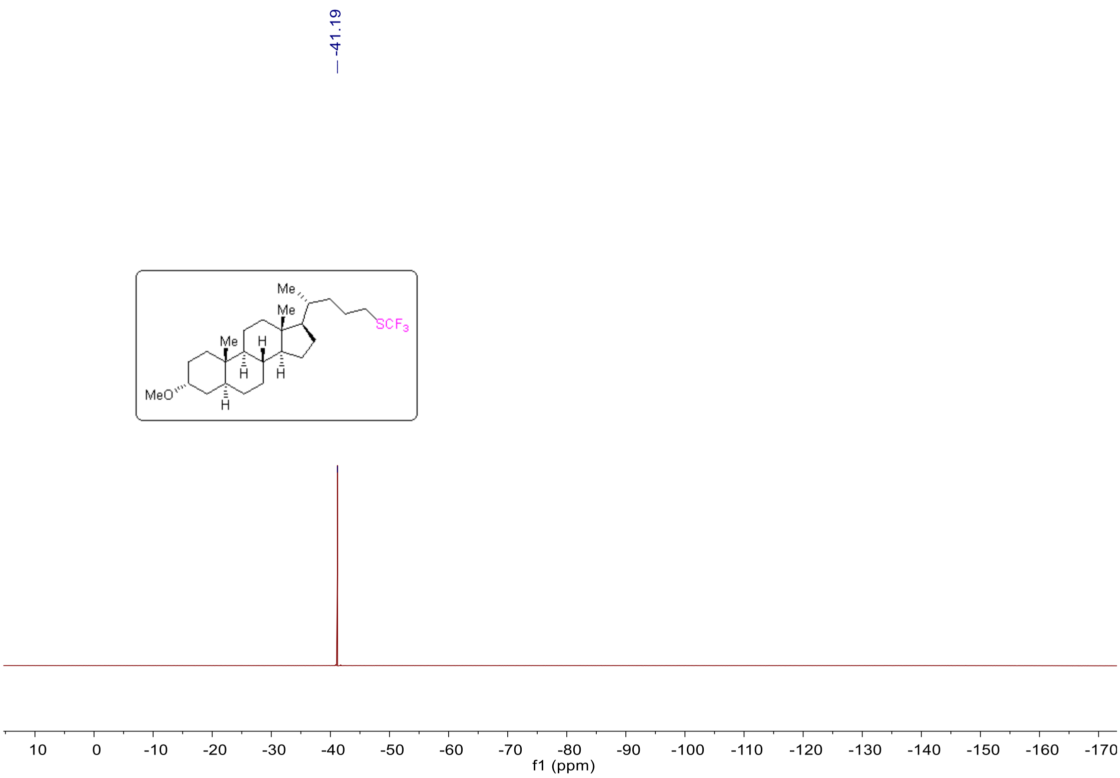

Supplementary Figure 134. ^{19}F NMR (376 MHz, $CDCl_3$) of **3oe'**

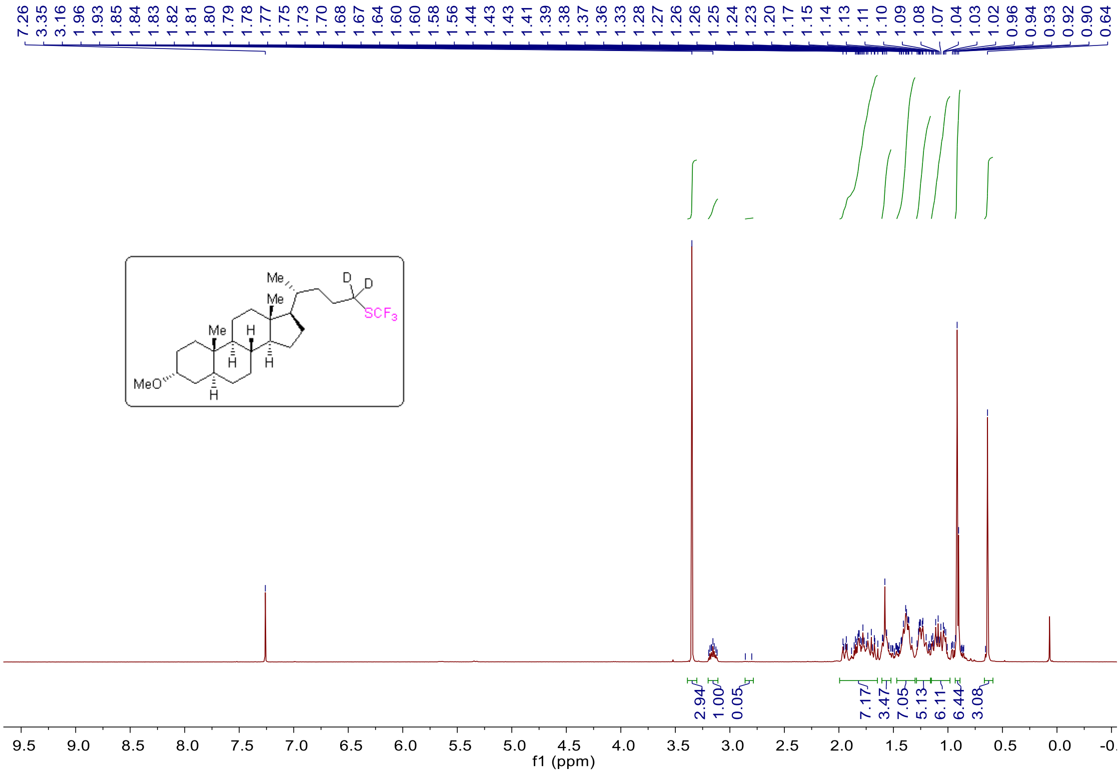

Supplementary Figure 135. ^{1}H NMR (400 MHz, $CDCl_3$) of **3oe**

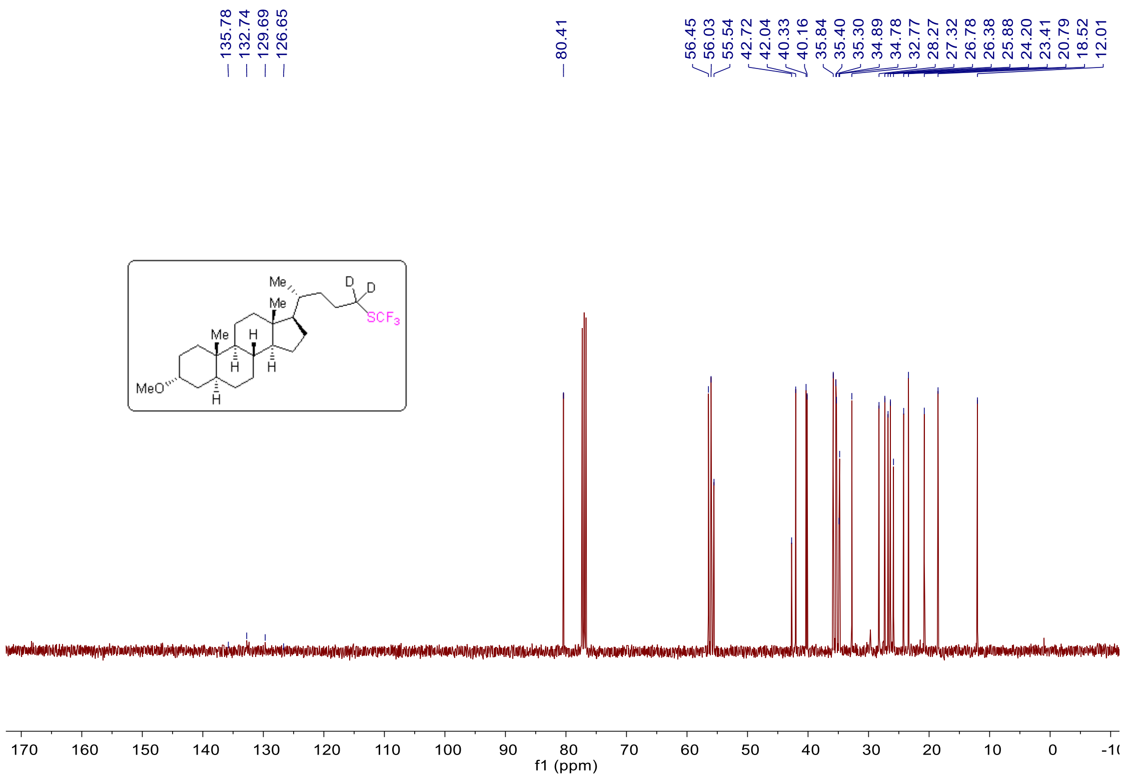

Supplementary Figure 136. ^{13}C NMR (101 MHz, $CDCl_3$) of **3oe**

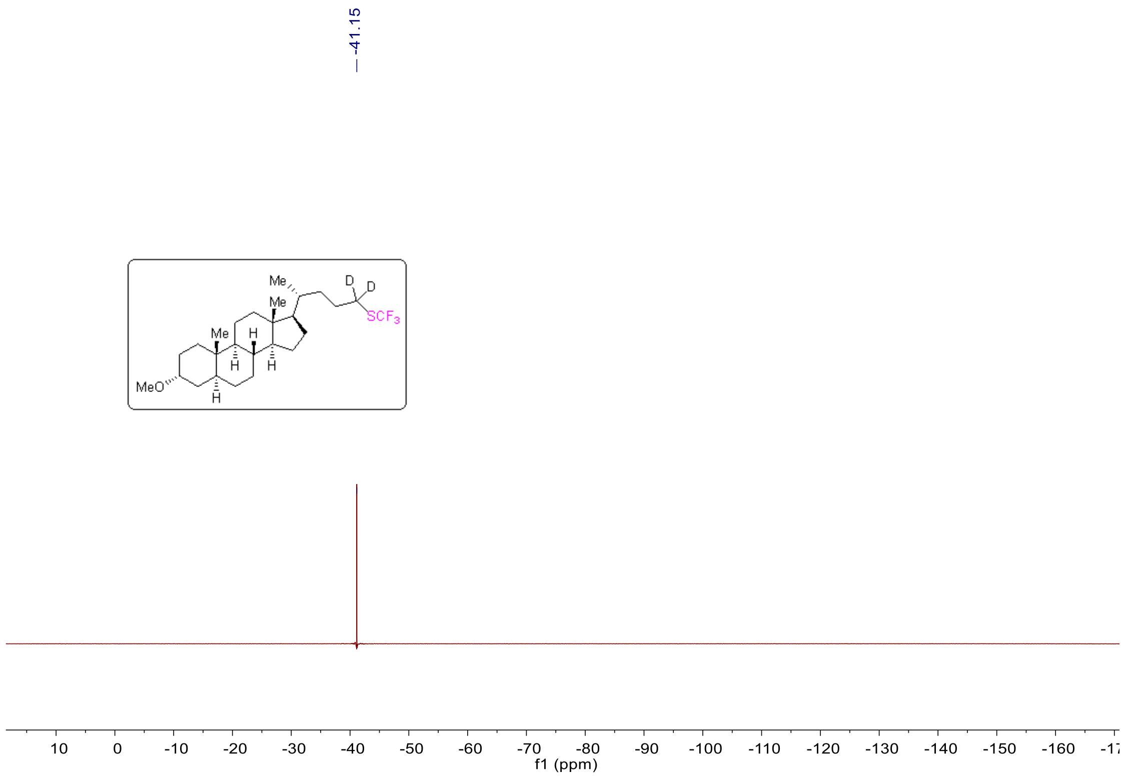

Supplementary Figure 137. ^{19}F NMR (376 MHz, $CDCl_3$) of **3oe**

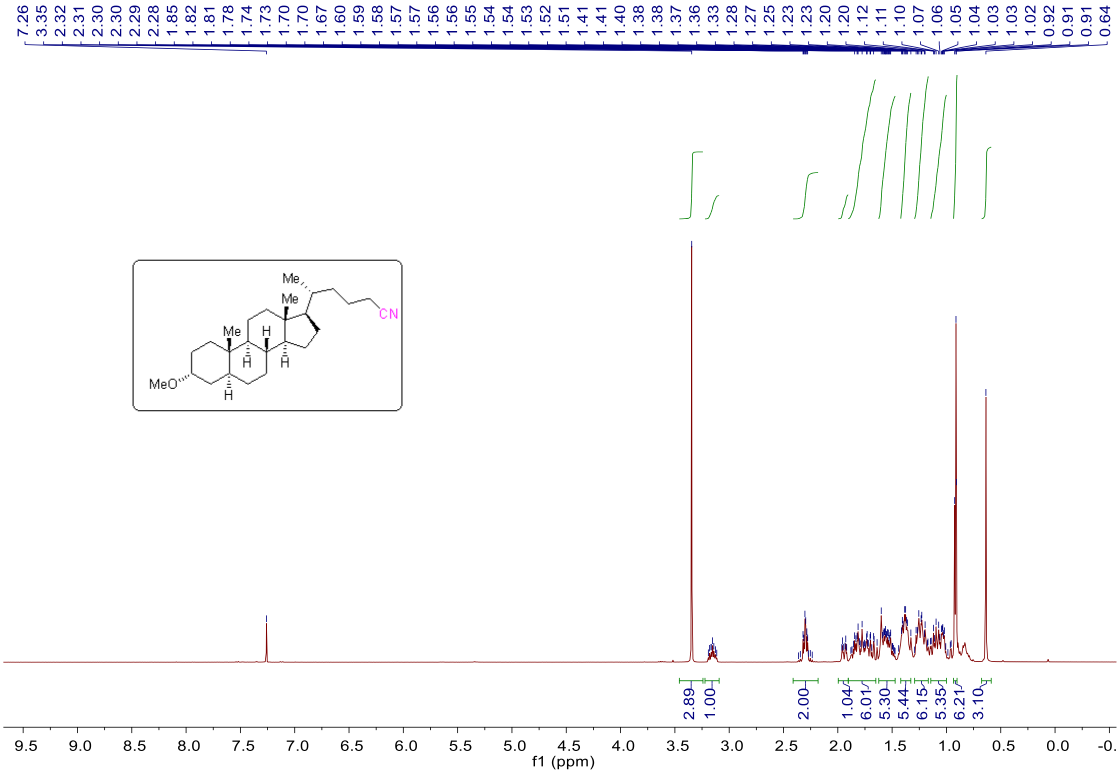

Supplementary Figure 138. ^{1}H NMR (400 MHz, $CDCl_3$) of **3of'**

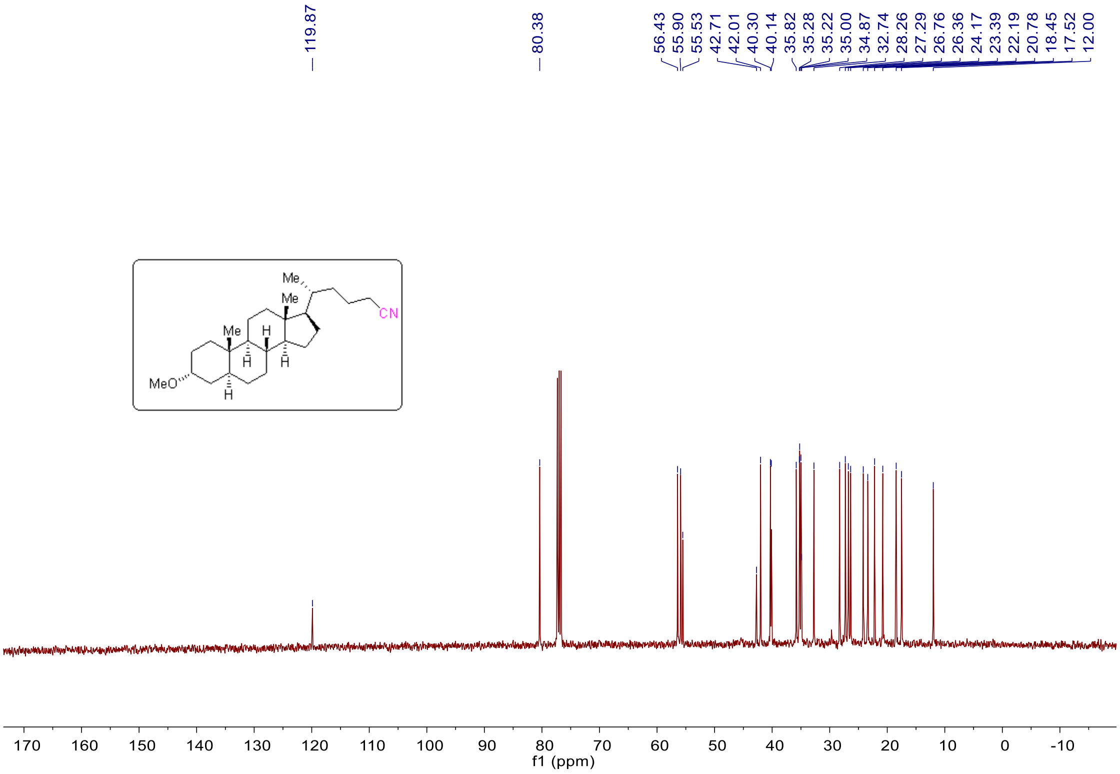

Supplementary Figure 139. ^{13}C NMR (101 MHz, $CDCl_3$) of **3of'**

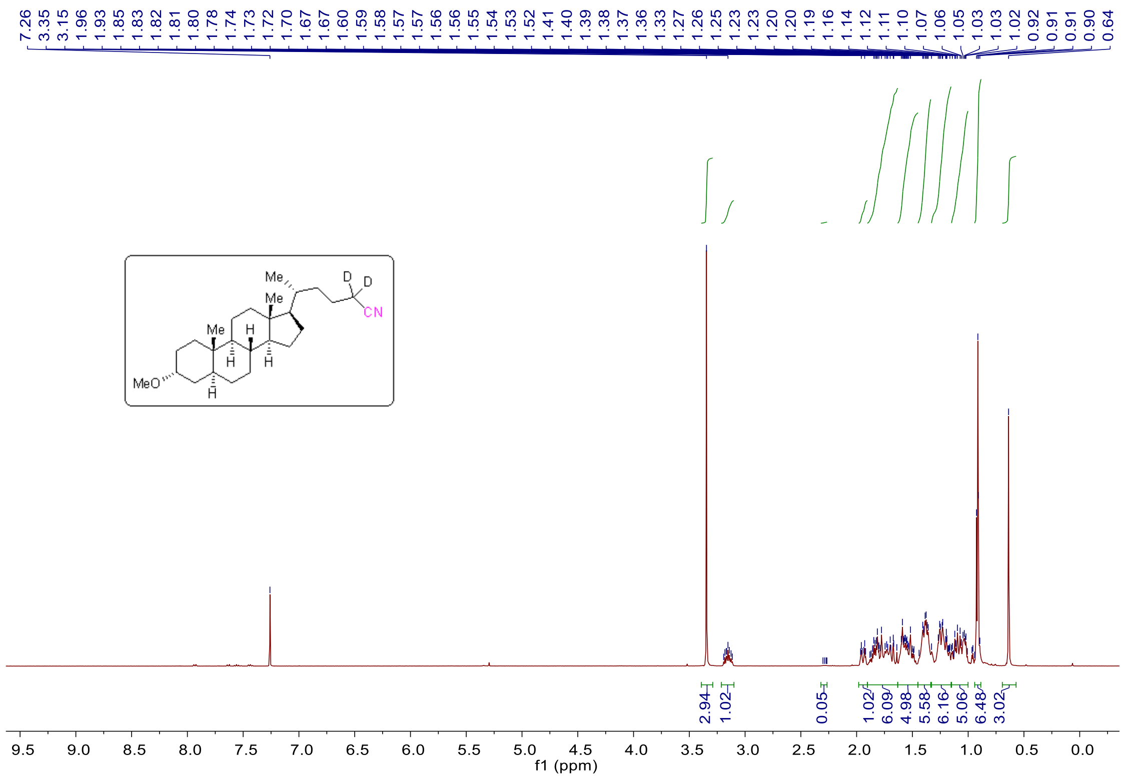

Supplementary Figure 140. ^{1}H NMR (400 MHz, $CDCl_3$) of **3of**

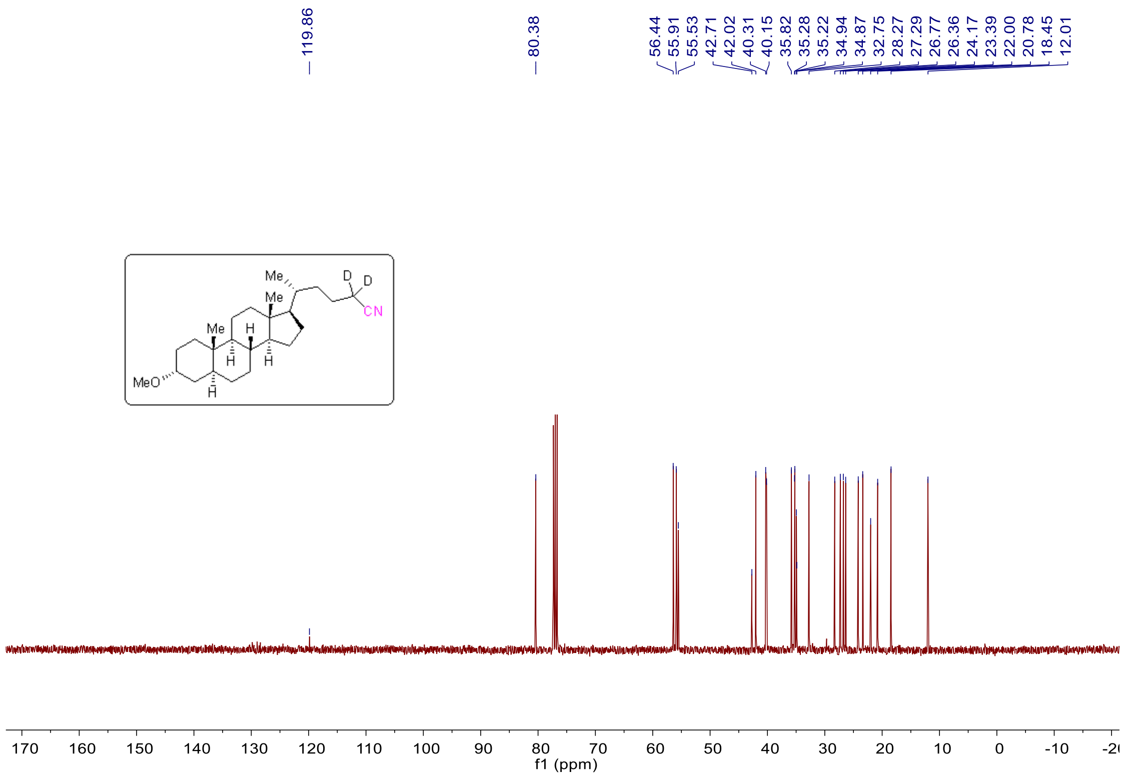

Supplementary Figure 141. ^{13}C NMR (101 MHz, $CDCl_3$) of **3of**

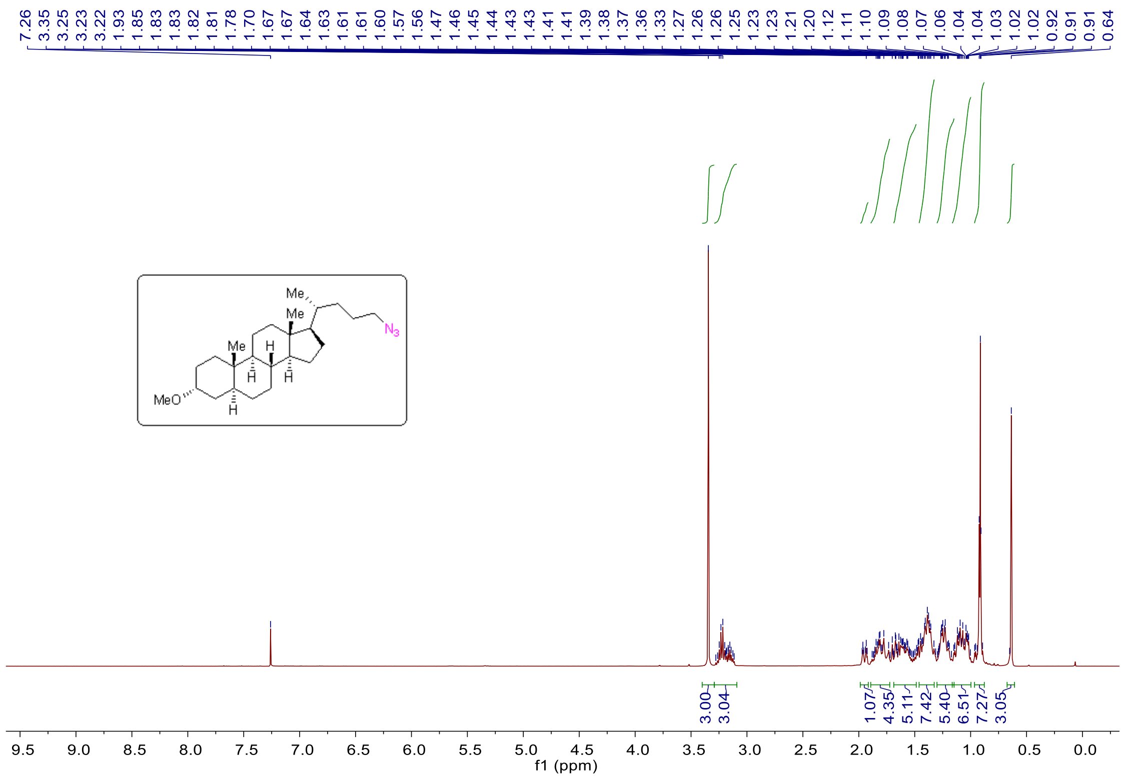

Supplementary Figure 142. ^{1}H NMR (400 MHz, $CDCl_3$) of **3og'**

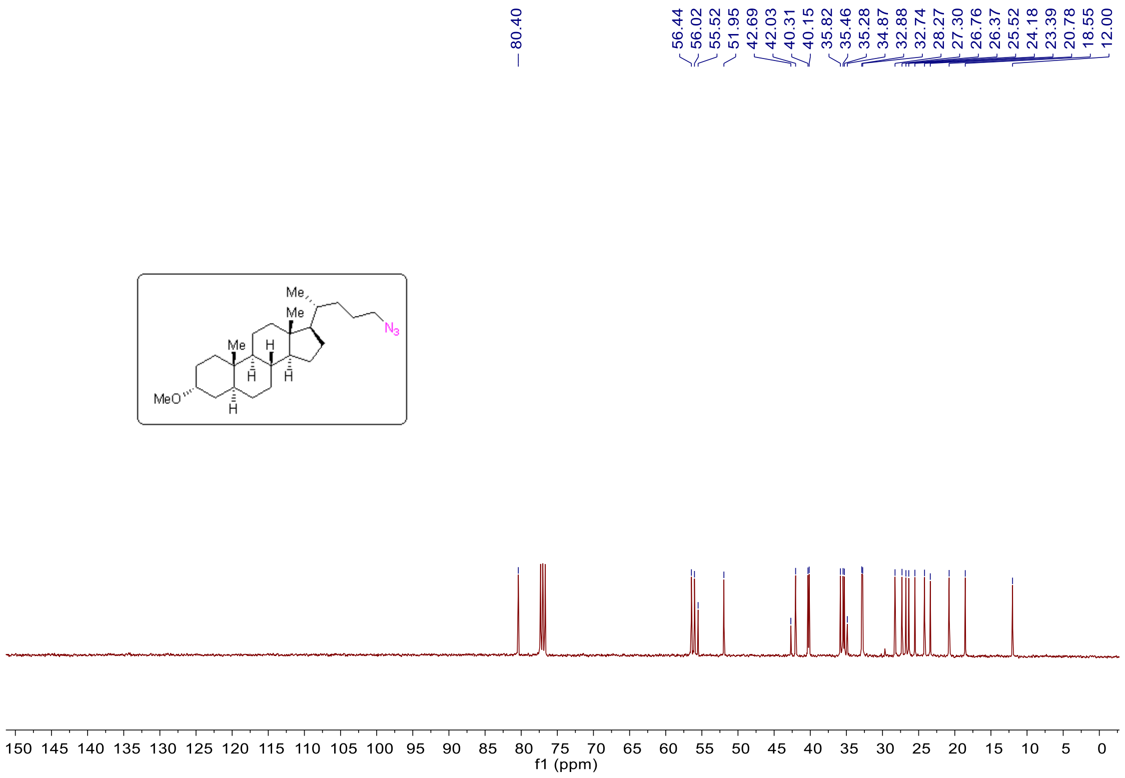

Supplementary Figure 143. ^{13}C NMR (101 MHz, $CDCl_3$) of **3og'**

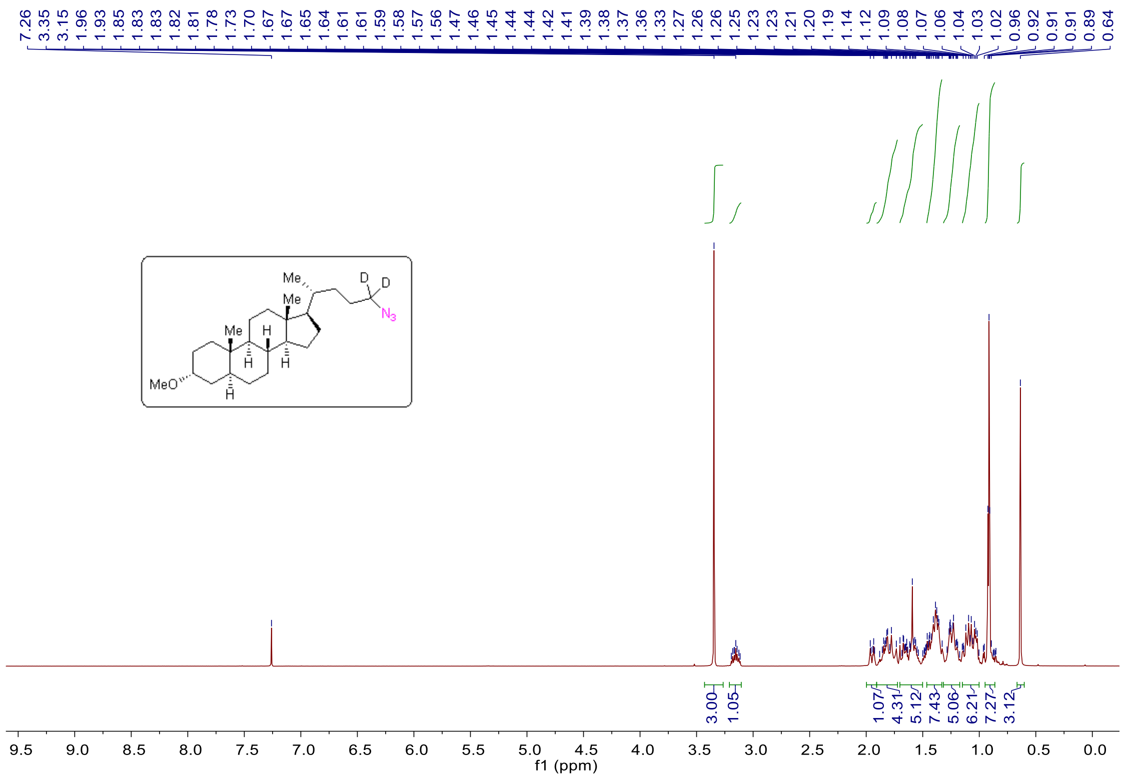

Supplementary Figure 144. ^{1}H NMR (400 MHz, $CDCl_3$) of **3og**

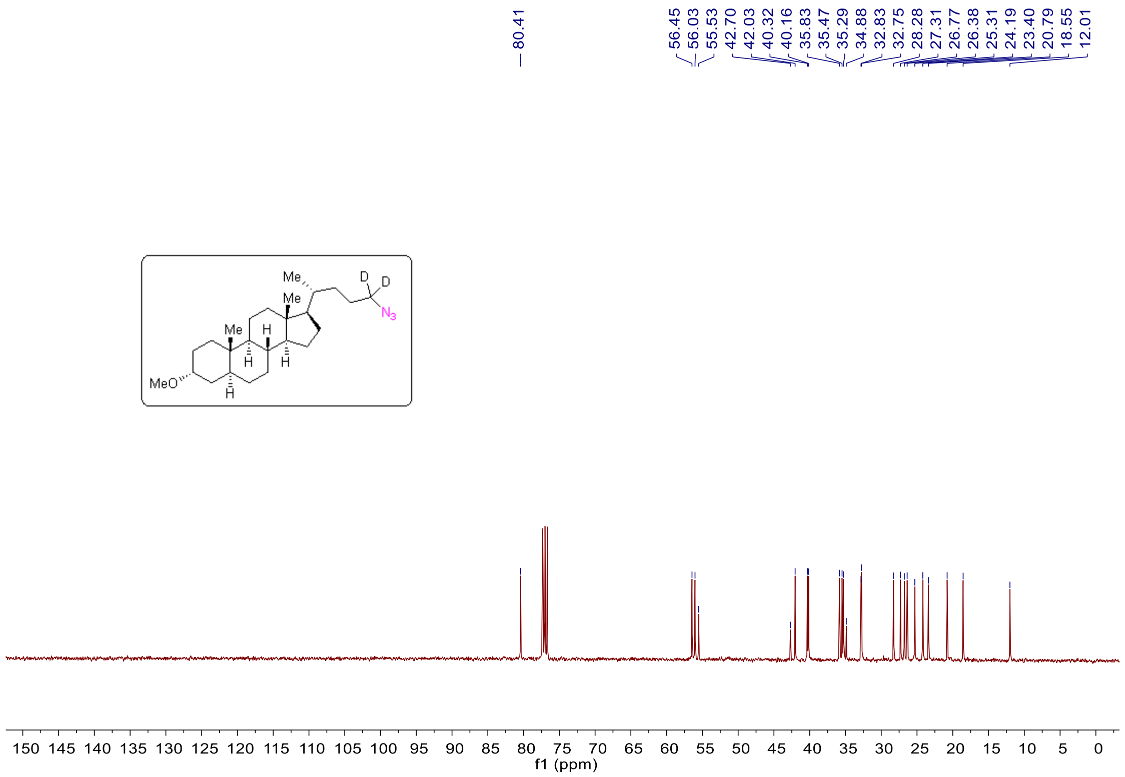

Supplementary Figure 145. ^{13}C NMR (101 MHz, $CDCl_3$) of **3og**

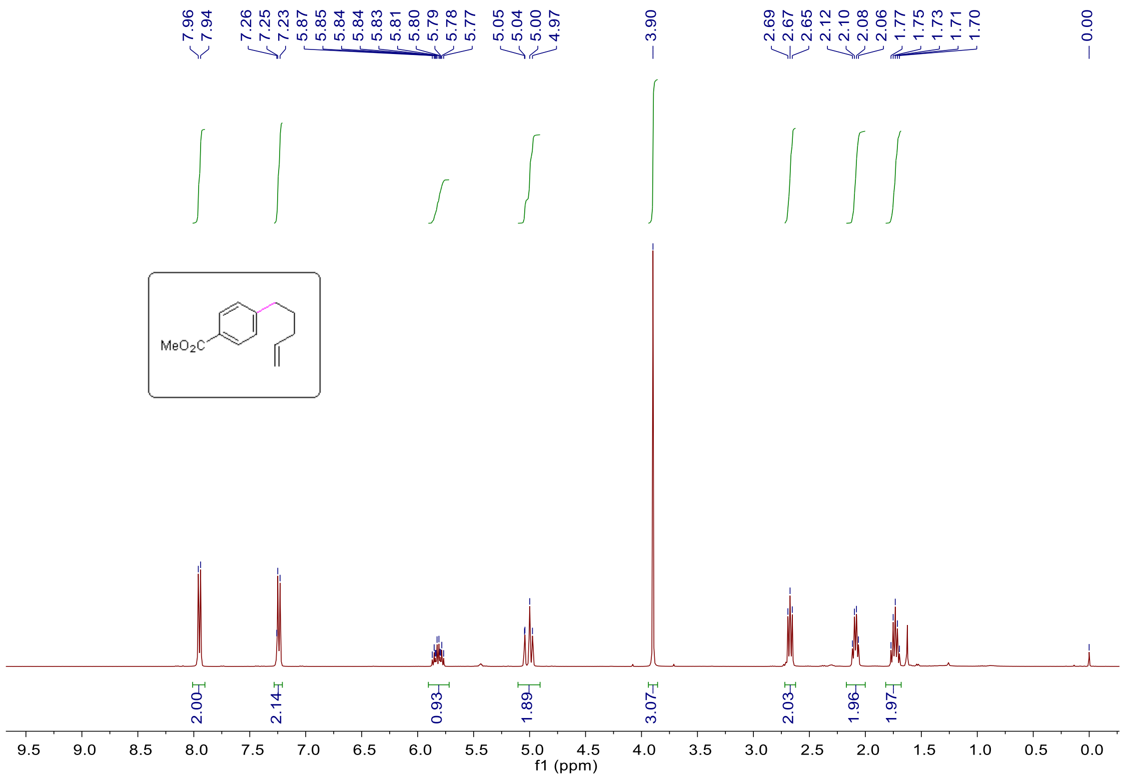

Supplementary Figure 146. ^{1}H NMR (400 MHz, $CDCl_3$) of **5aa'**

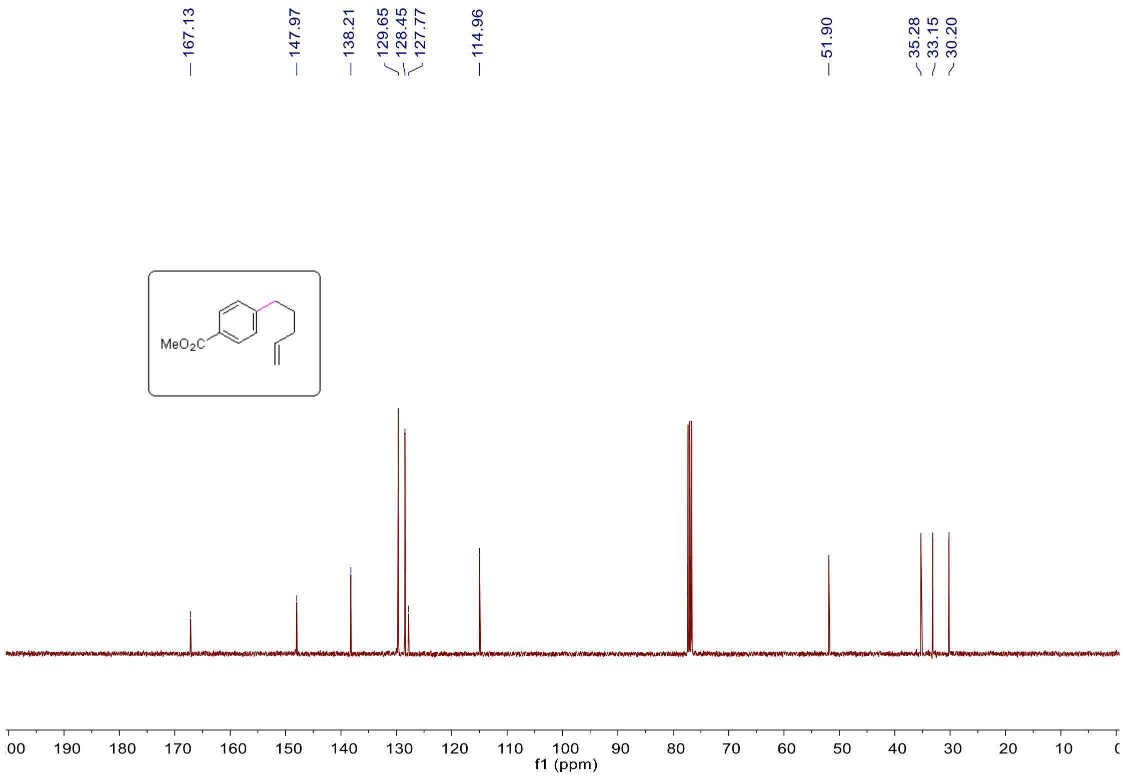

Supplementary Figure 147. ^{13}C NMR (101 MHz, $CDCl_3$) of **5aa'**

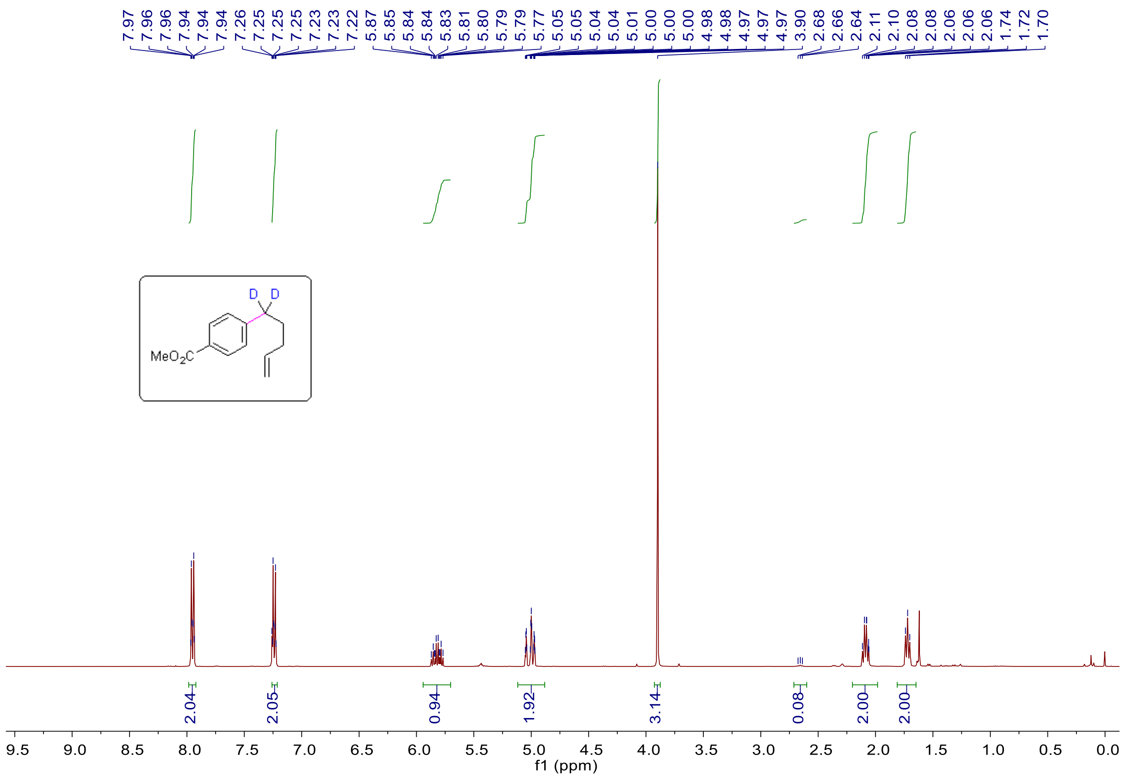

Supplementary Figure 148. ^{1}H NMR (400 MHz, $CDCl_3$) of **5aa**

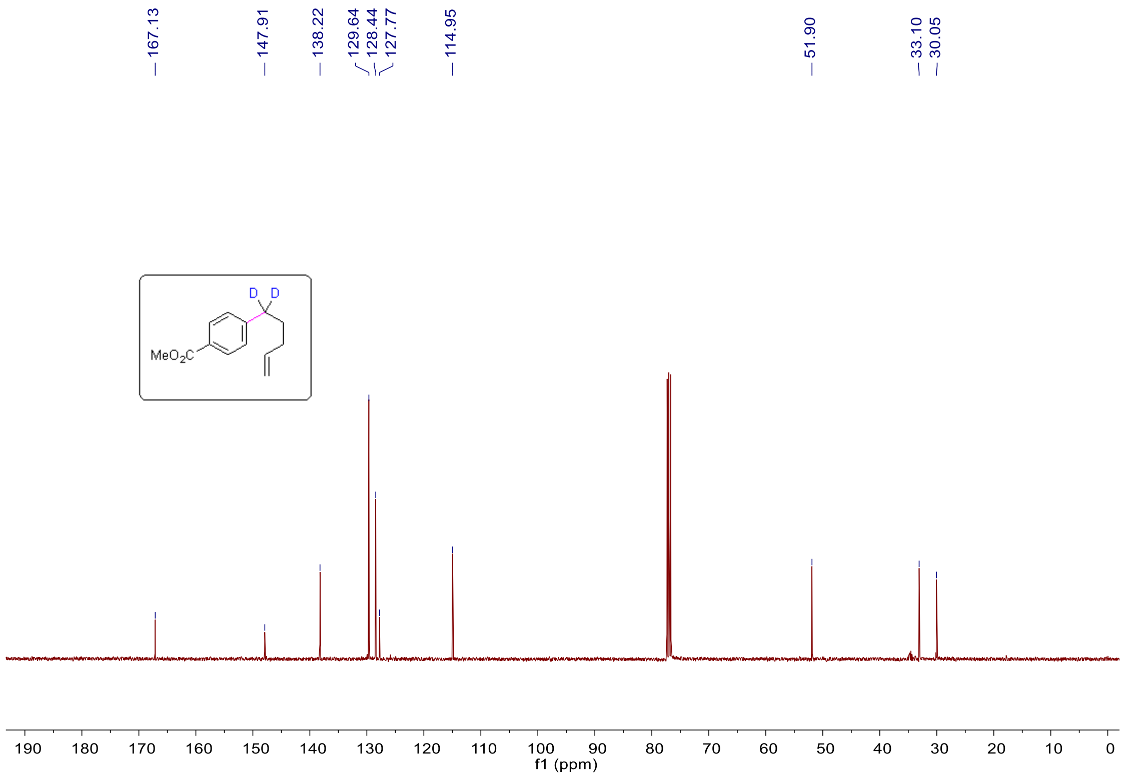

Supplementary Figure 149. ^{13}C NMR (101 MHz, $CDCl_3$) of **5aa**

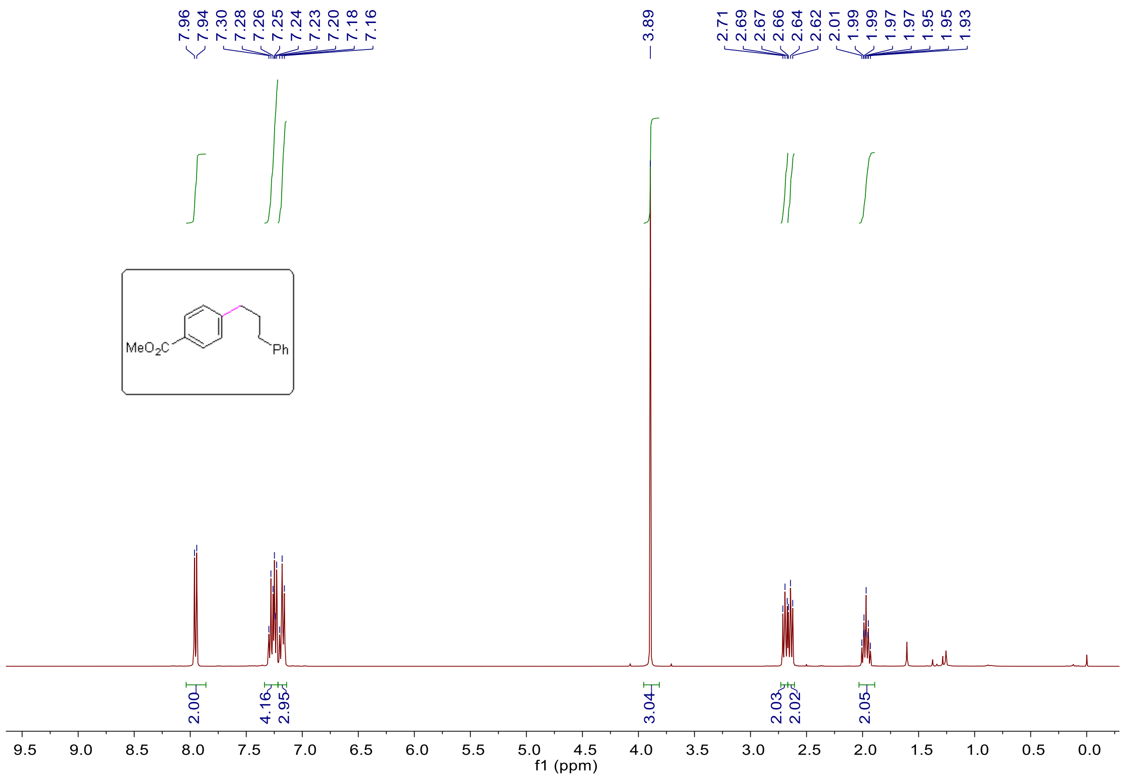

Supplementary Figure 150. ^{1}H NMR (400 MHz, $CDCl_3$) of **5ba'**

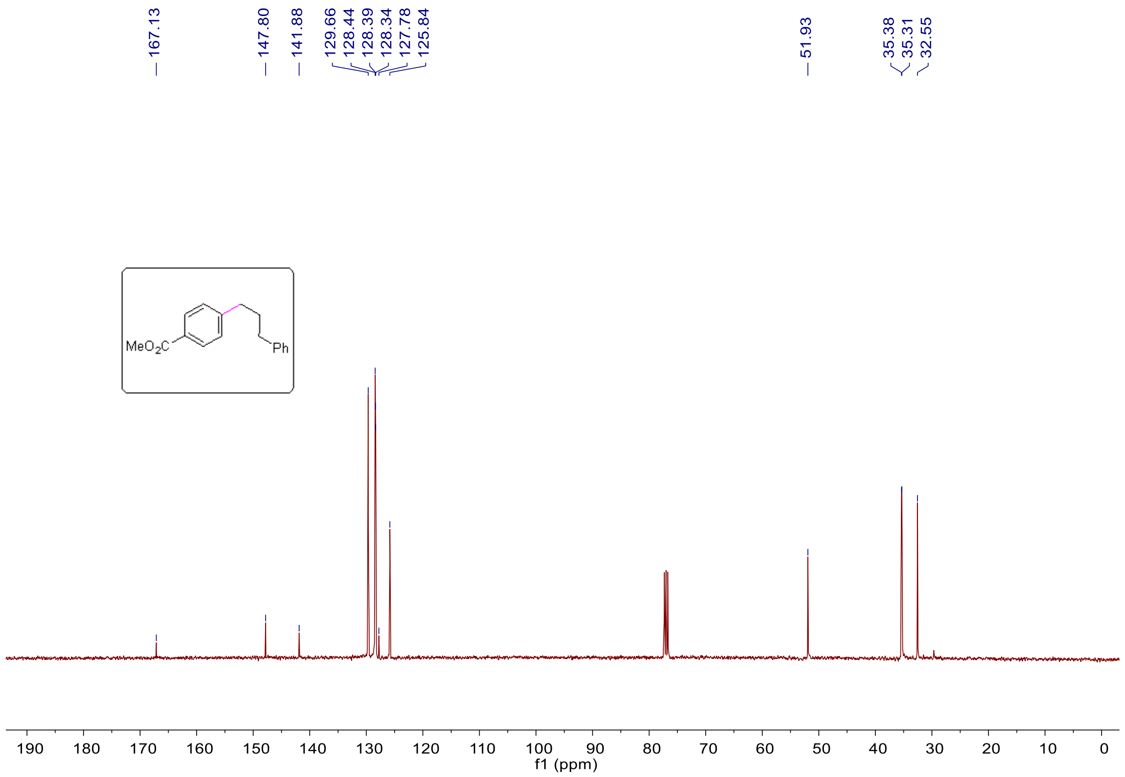

Supplementary Figure 151. ^{13}C NMR (101 MHz, $CDCl_3$) of **5ba'**

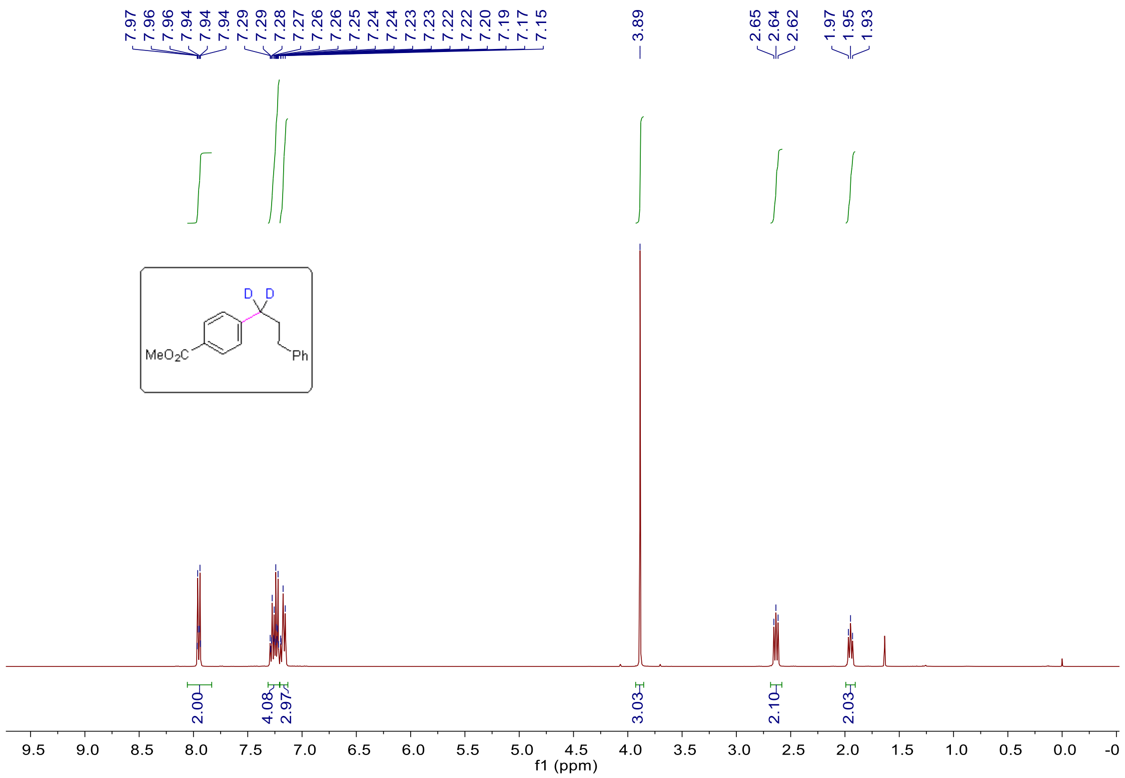

Supplementary Figure 152. ^{1}H NMR (400 MHz, $CDCl_3$) of **5ba**

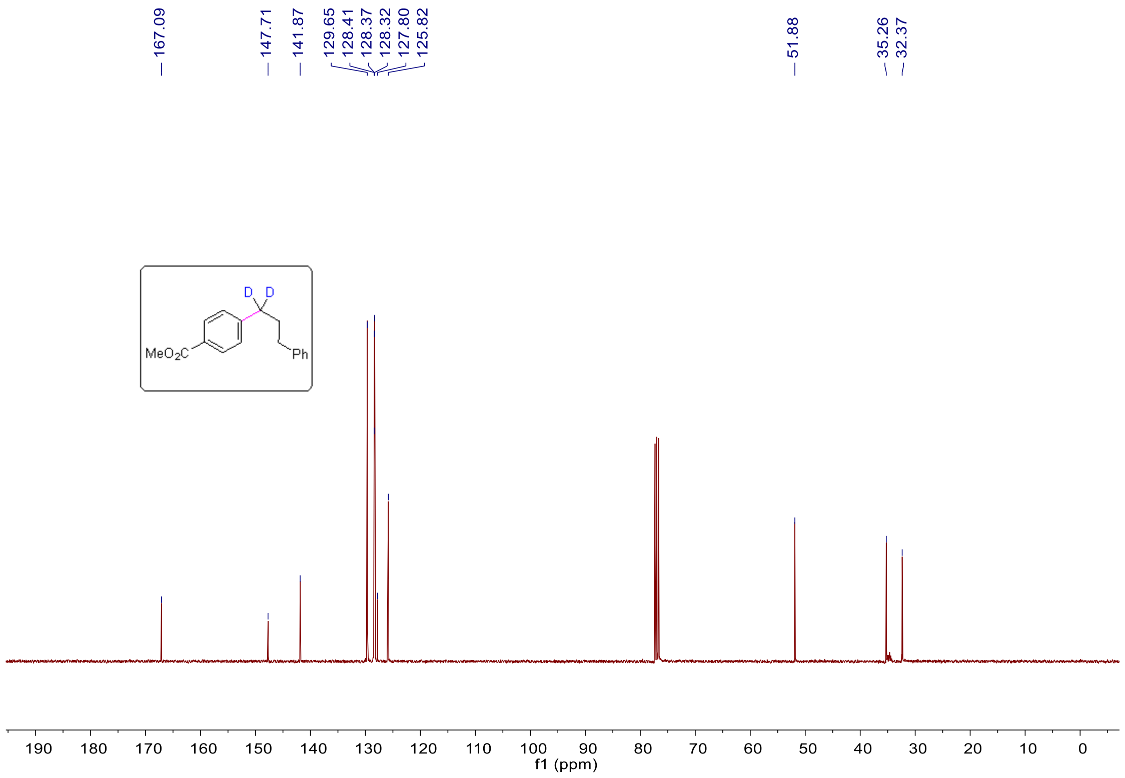

Supplementary Figure 153. ^{13}C NMR (101 MHz, $CDCl_3$) of **5ba**

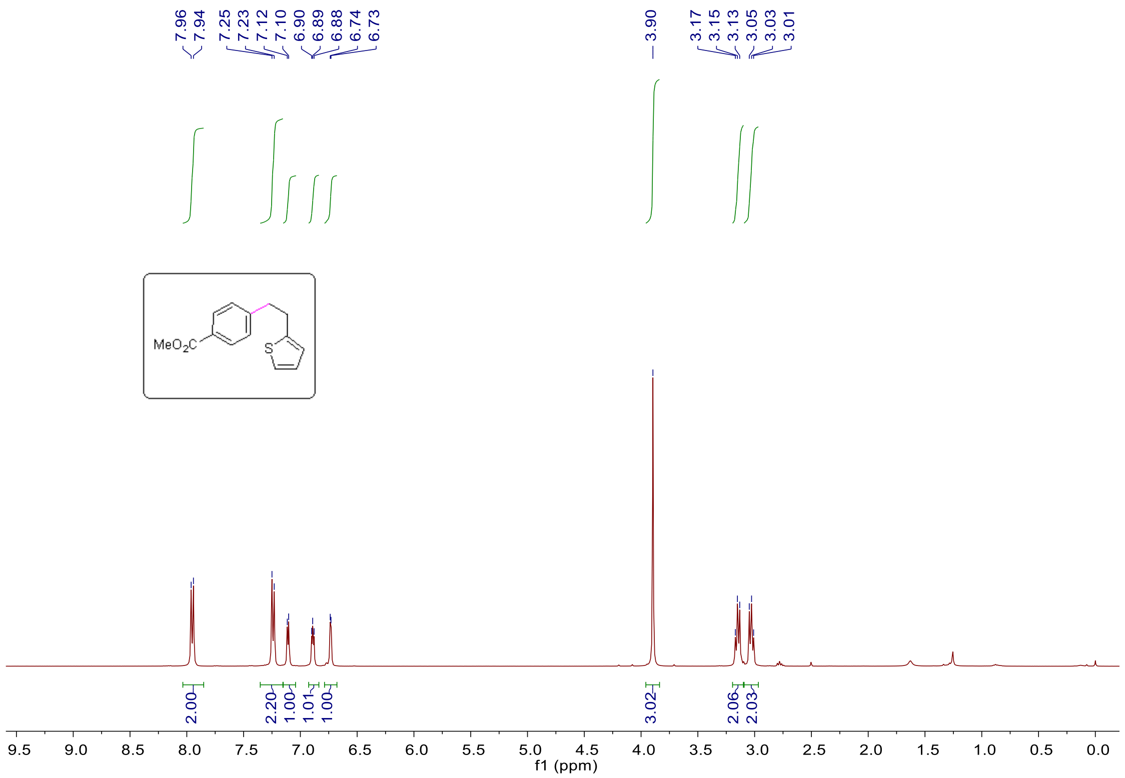

Supplementary Figure 154. ^{1}H NMR (400 MHz, $CDCl_3$) of **5ca'**

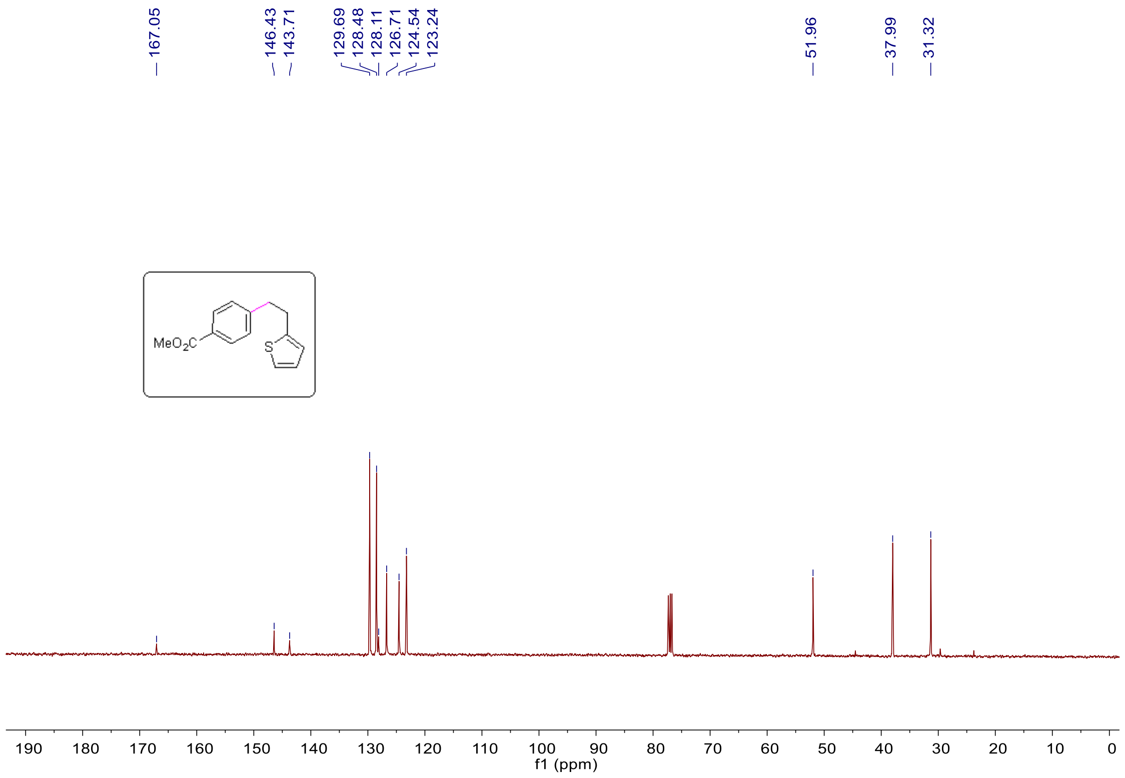

Supplementary Figure 155. ^{13}C NMR (101 MHz, $CDCl_3$) of **5ca'**

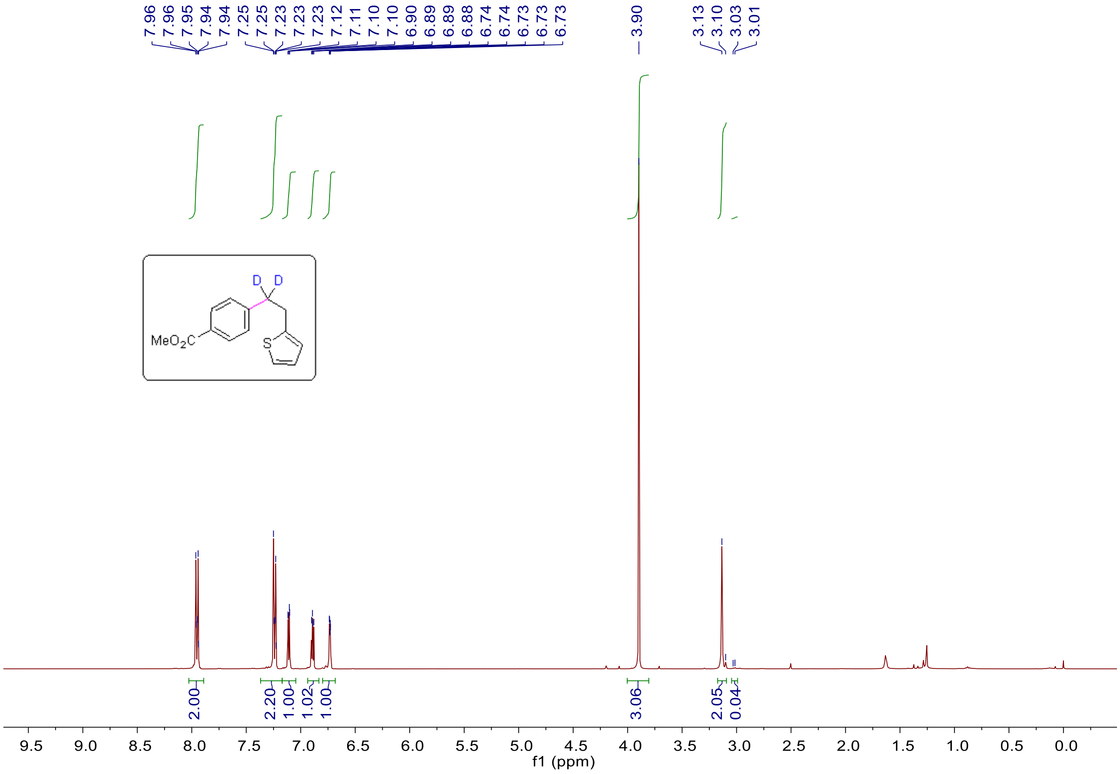

Supplementary Figure 156. ^{1}H NMR (400 MHz, $CDCl_3$) of **5ca**

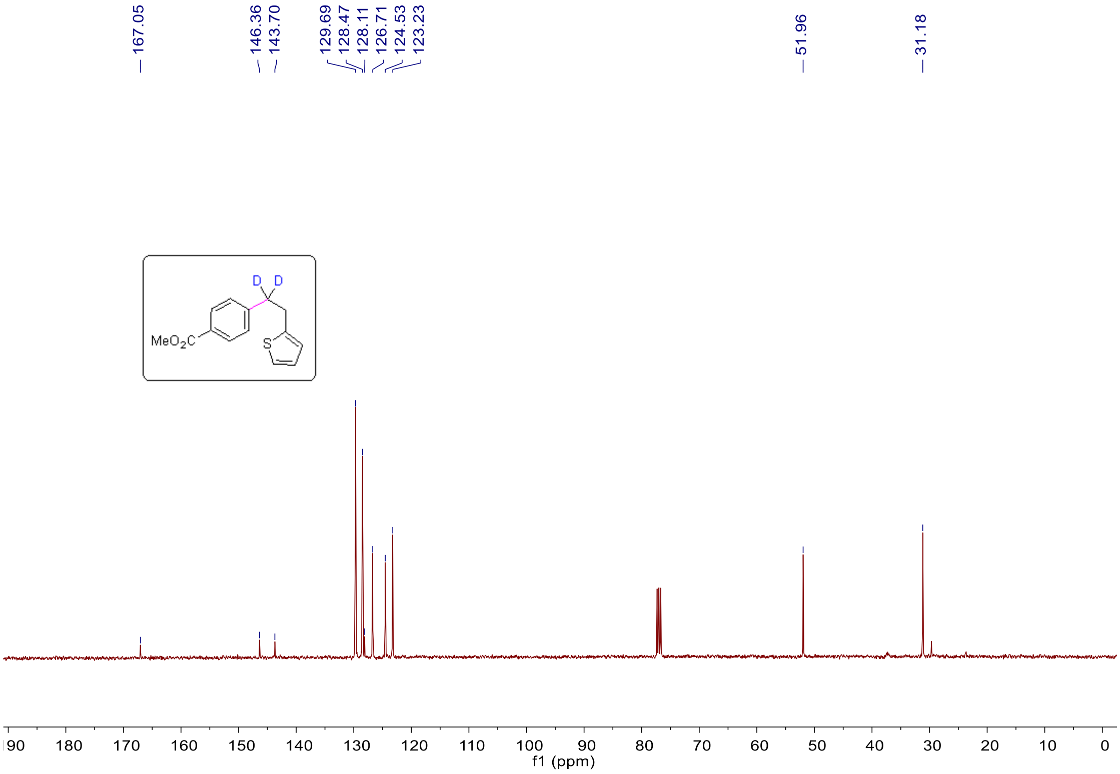

Supplementary Figure 157. ^{13}C NMR (101 MHz, $CDCl_3$) of **5ca**

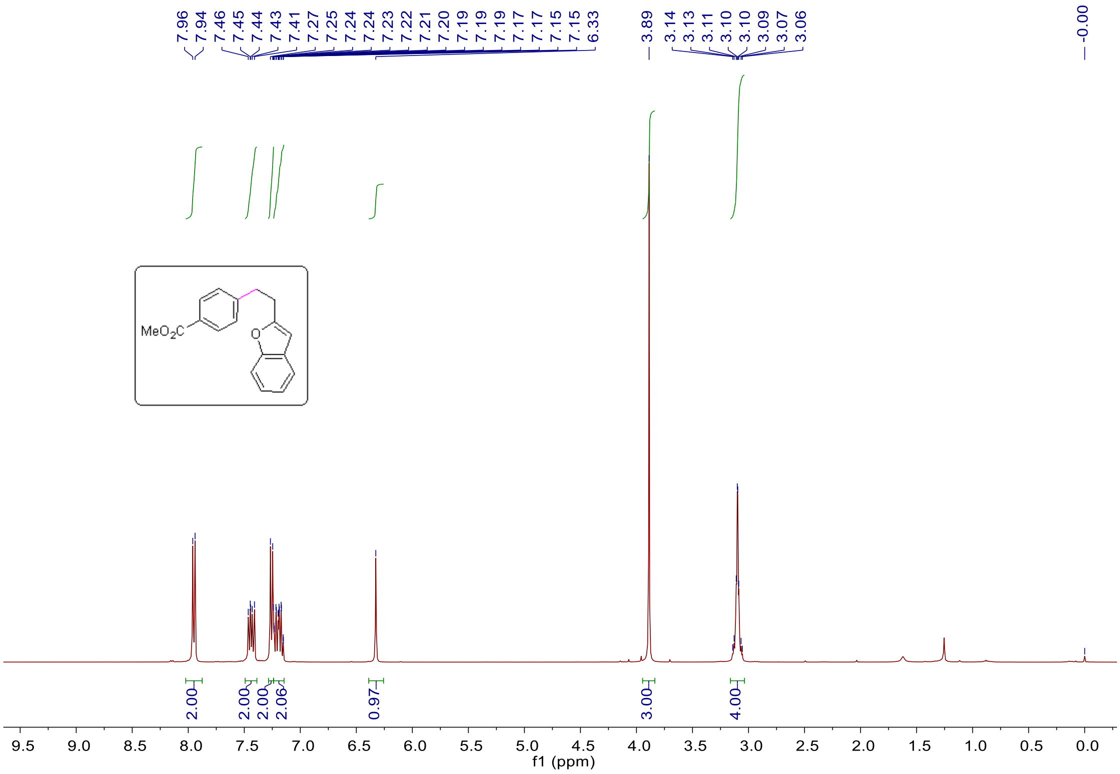

Supplementary Figure 158. ^{1}H NMR (400 MHz, $CDCl_3$) of **5da'**

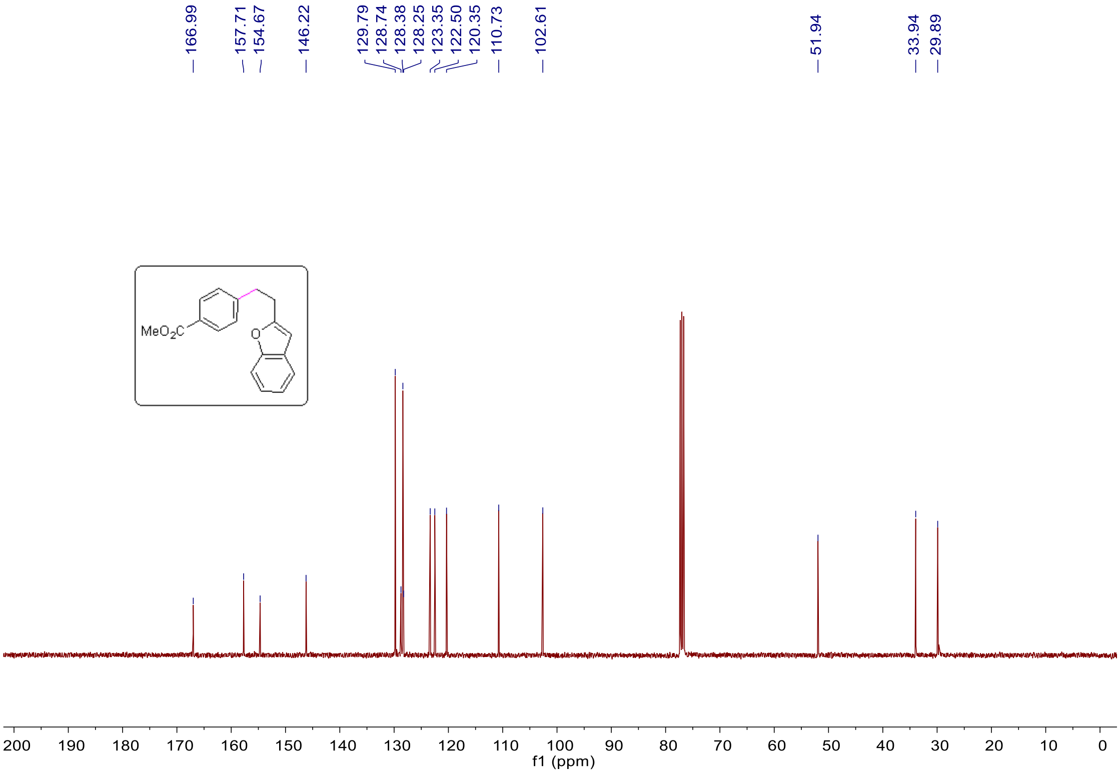

Supplementary Figure 159. ^{13}C NMR (101 MHz, $CDCl_3$) of **5da'**

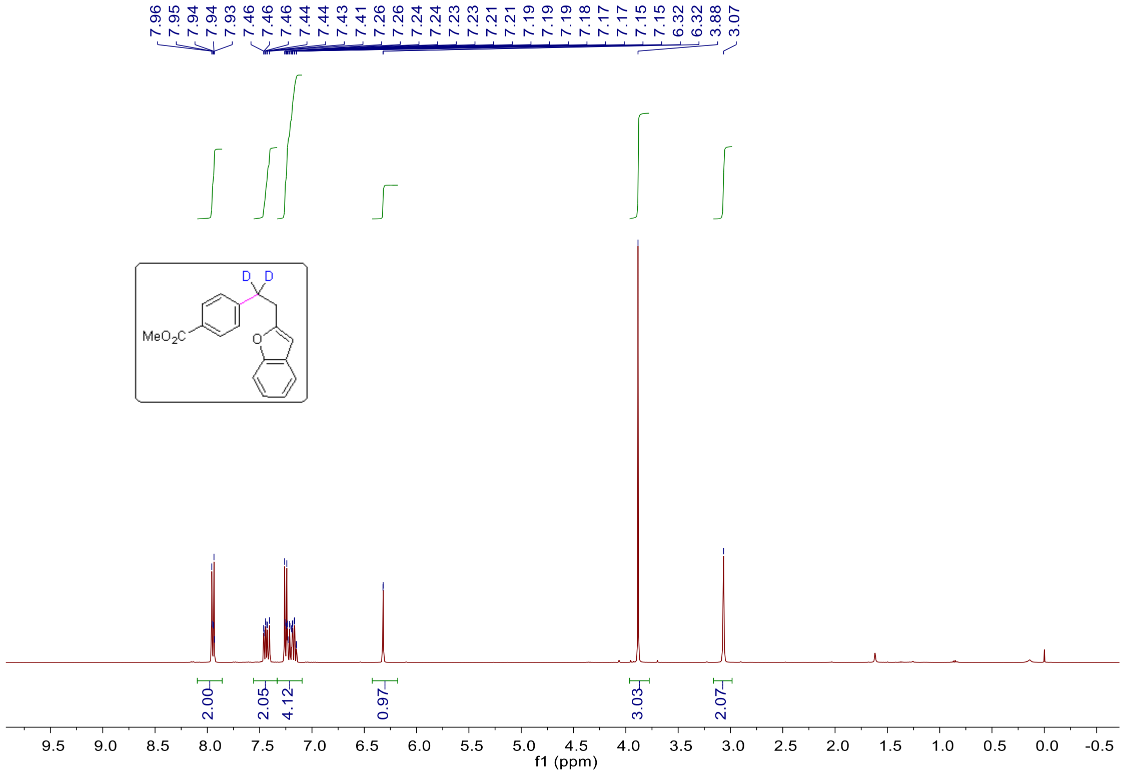

Supplementary Figure 160. ^{1}H NMR (400 MHz, $CDCl_3$) of **5da**

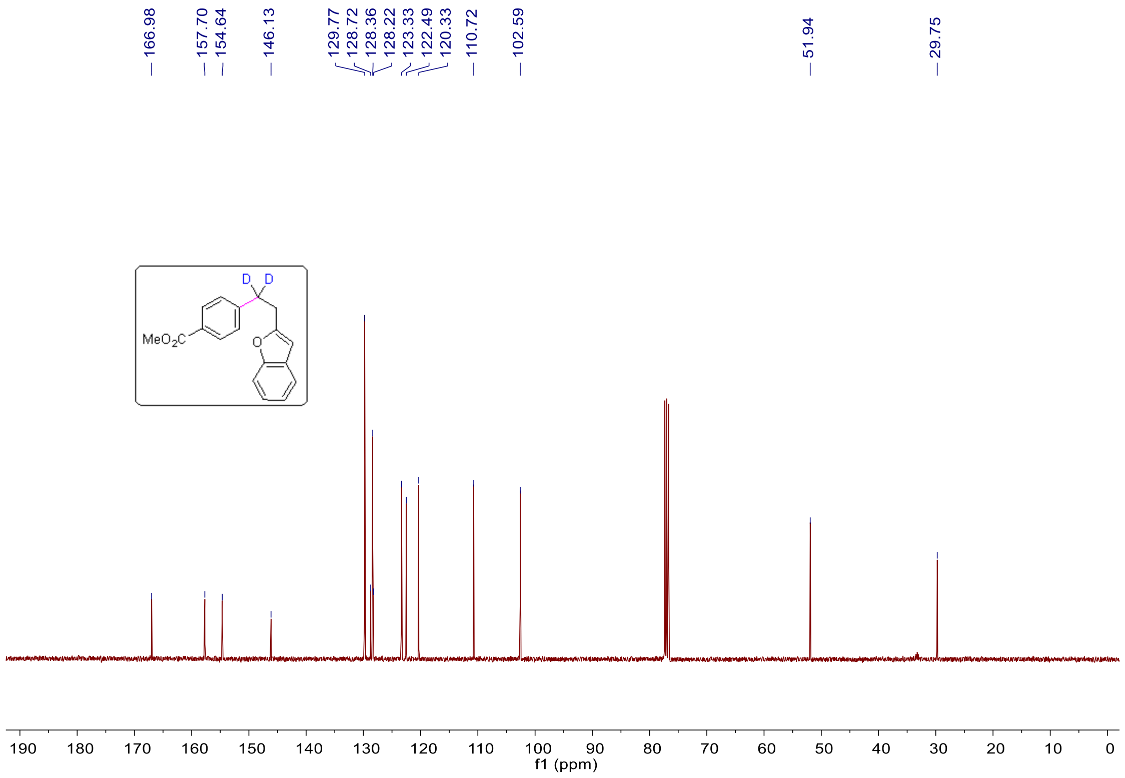

Supplementary Figure 161. ^{13}C NMR (101 MHz, $CDCl_3$) of **5da**

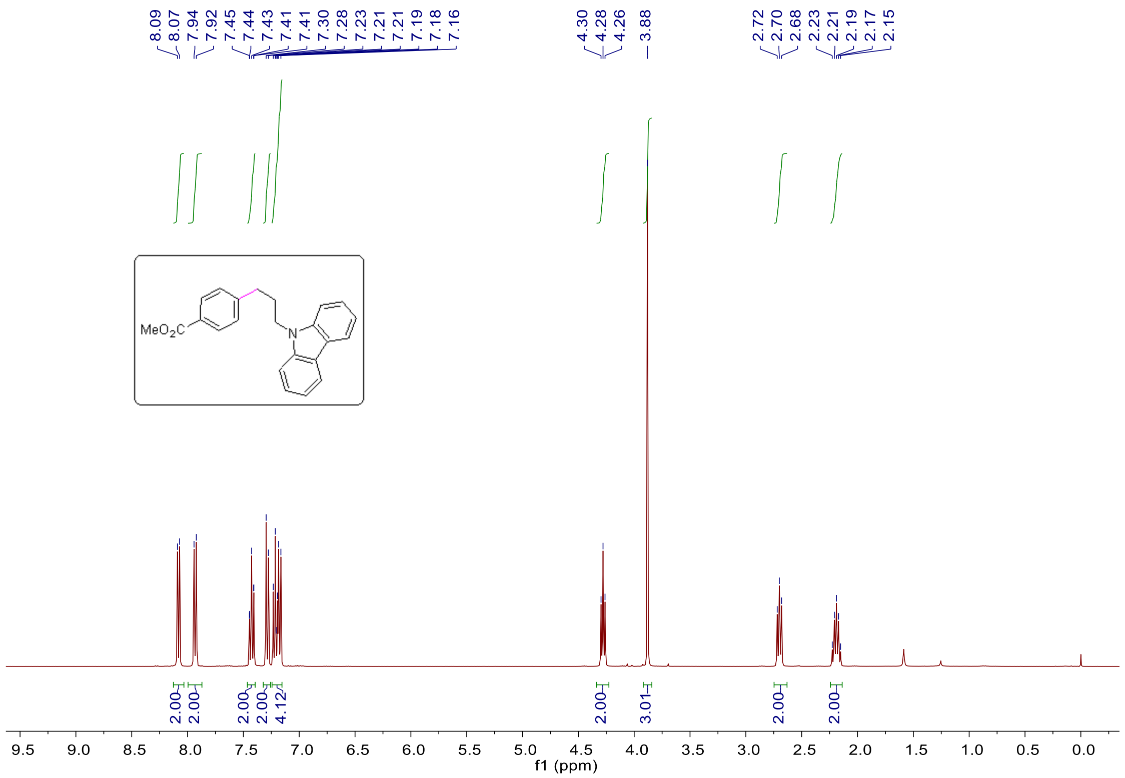

Supplementary Figure 162. ^{1}H NMR (400 MHz, $CDCl_3$) of **5ea'**

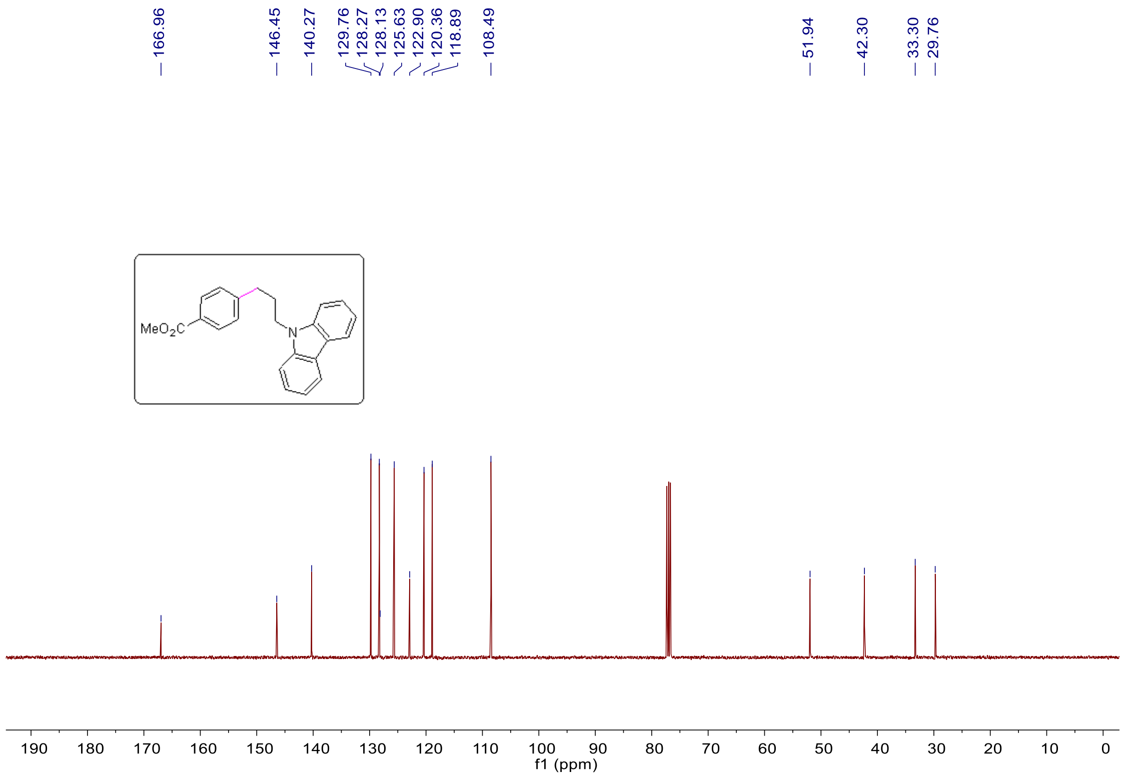

Supplementary Figure 163. ^{13}C NMR (101 MHz, $CDCl_3$) of **5ea'**

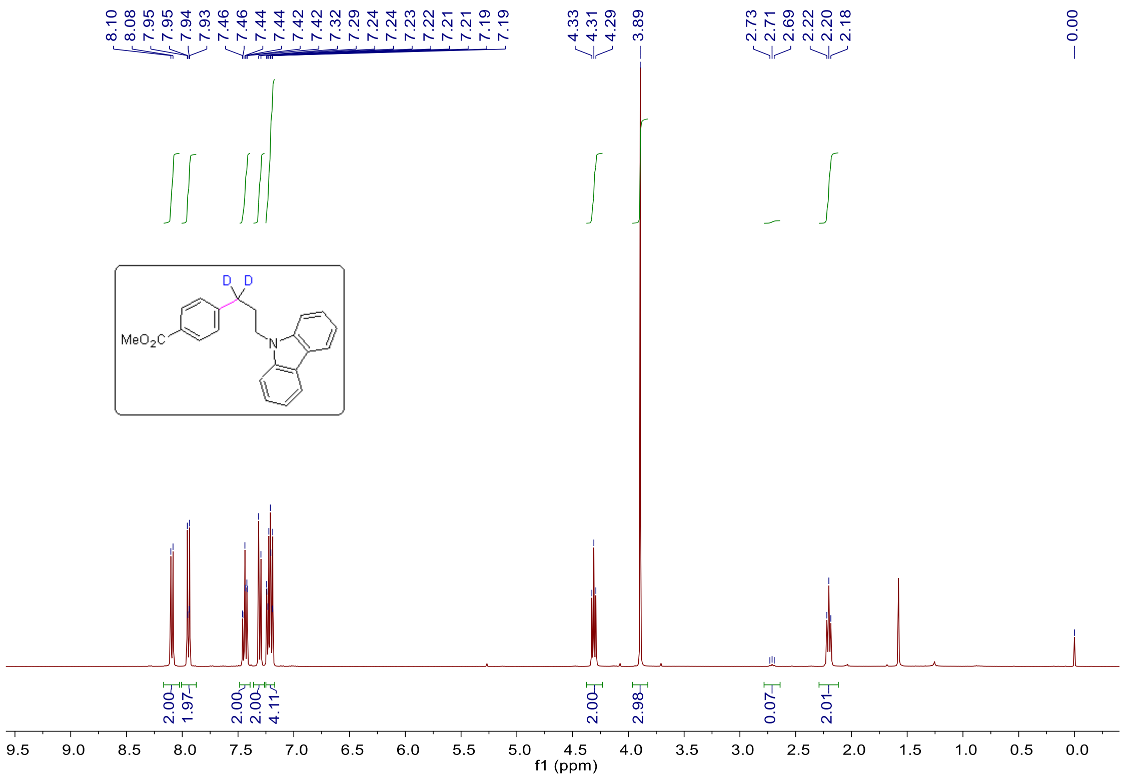

Supplementary Figure 164. ^{1}H NMR (400 MHz, $CDCl_3$) of **5ea**

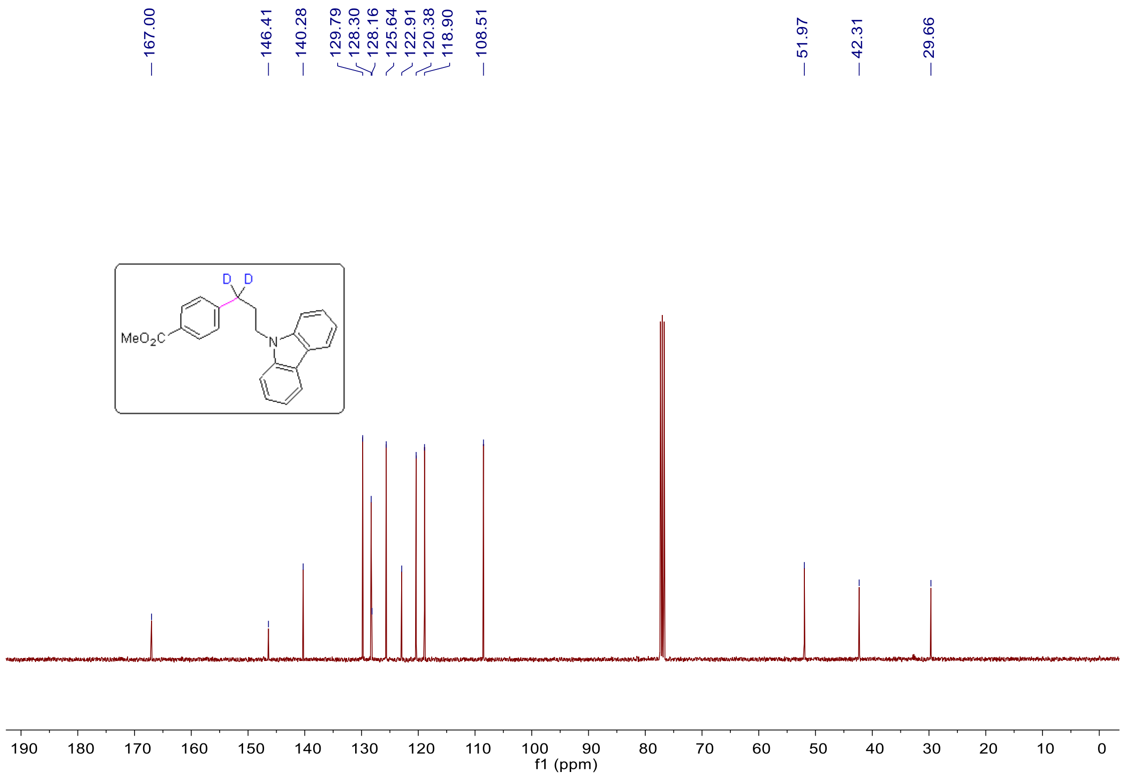

Supplementary Figure 165. ^{13}C NMR (101 MHz, $CDCl_3$) of **5ea**

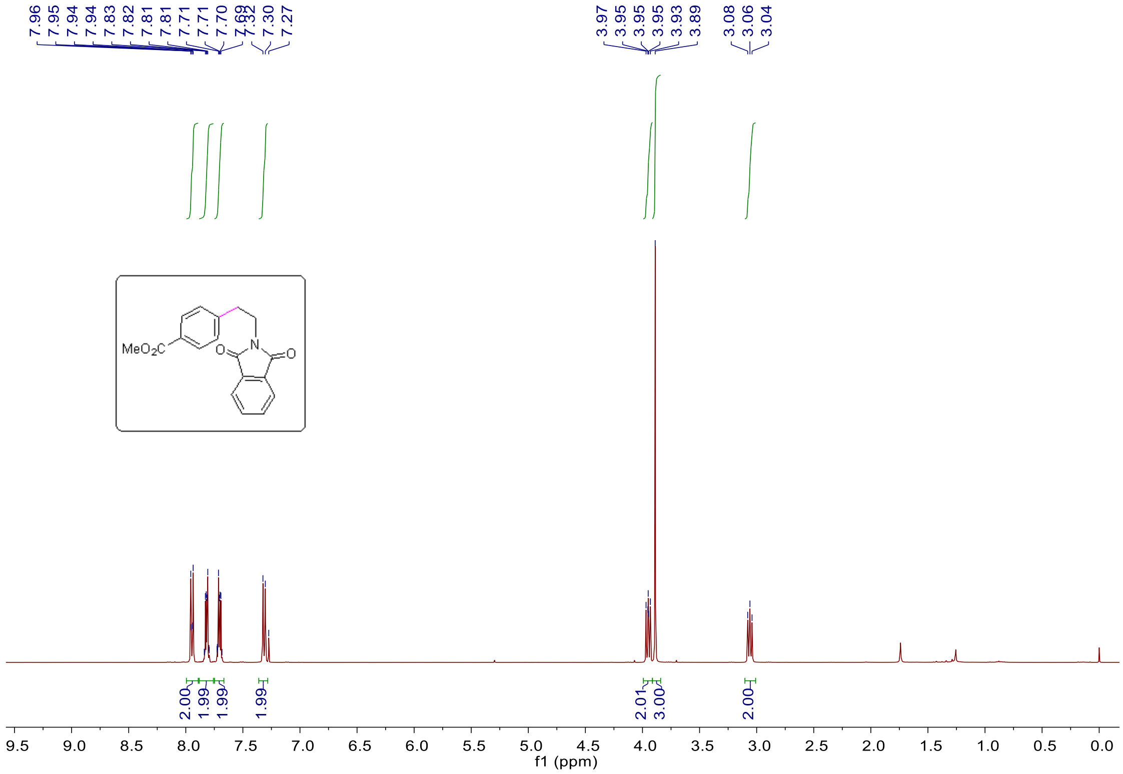

Supplementary Figure 166. ^{1}H NMR (400 MHz, $CDCl_3$) of **5fa'**

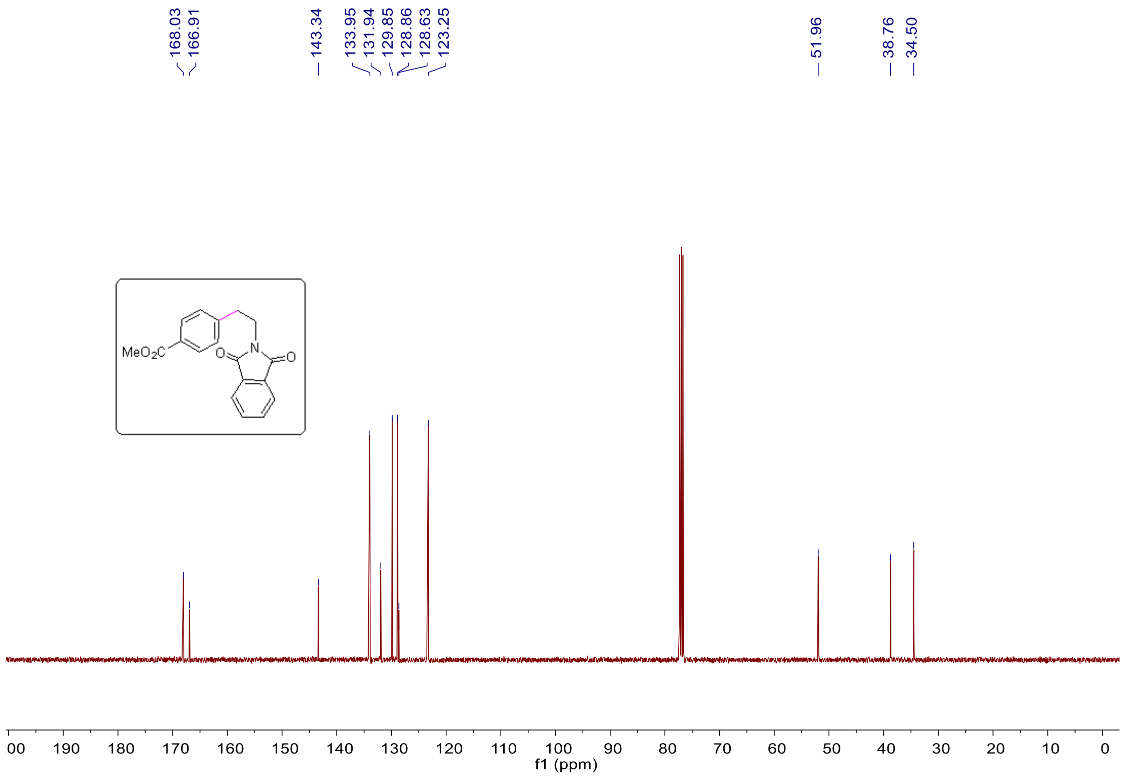

Supplementary Figure 167. ^{13}C NMR (101 MHz, $CDCl_3$) of **5fa'**

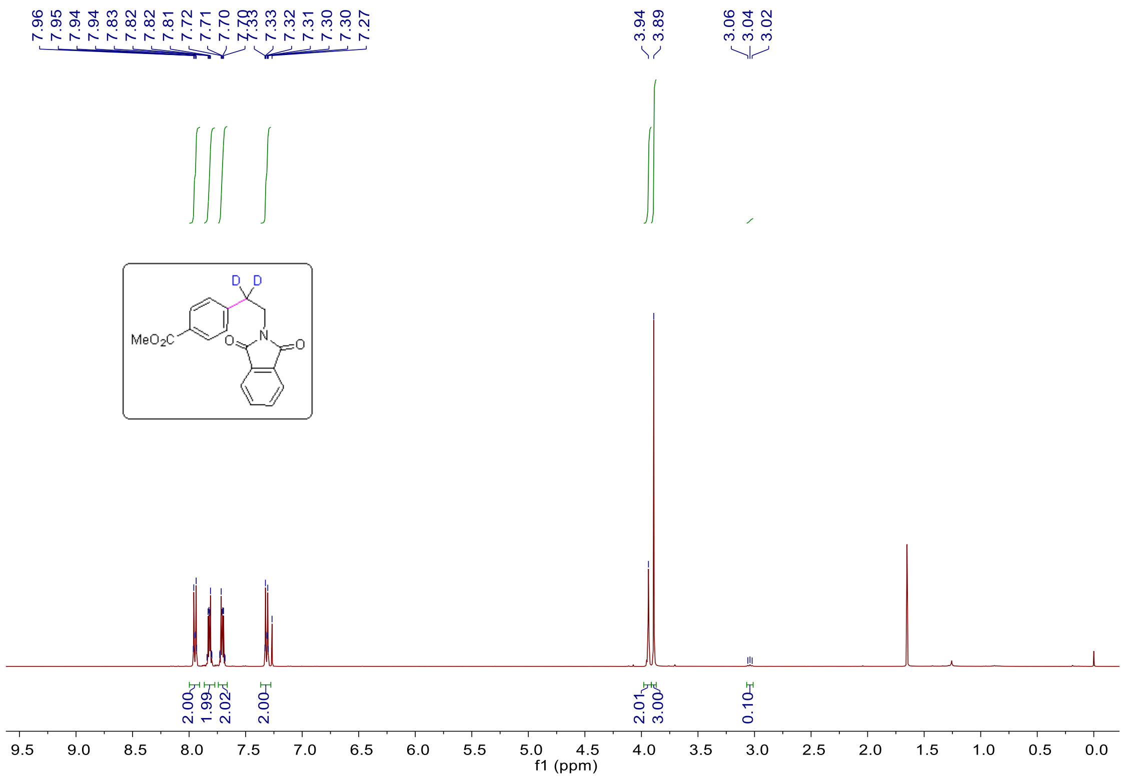

Supplementary Figure 168. ^{1}H NMR (400 MHz, $CDCl_3$) of **5fa**

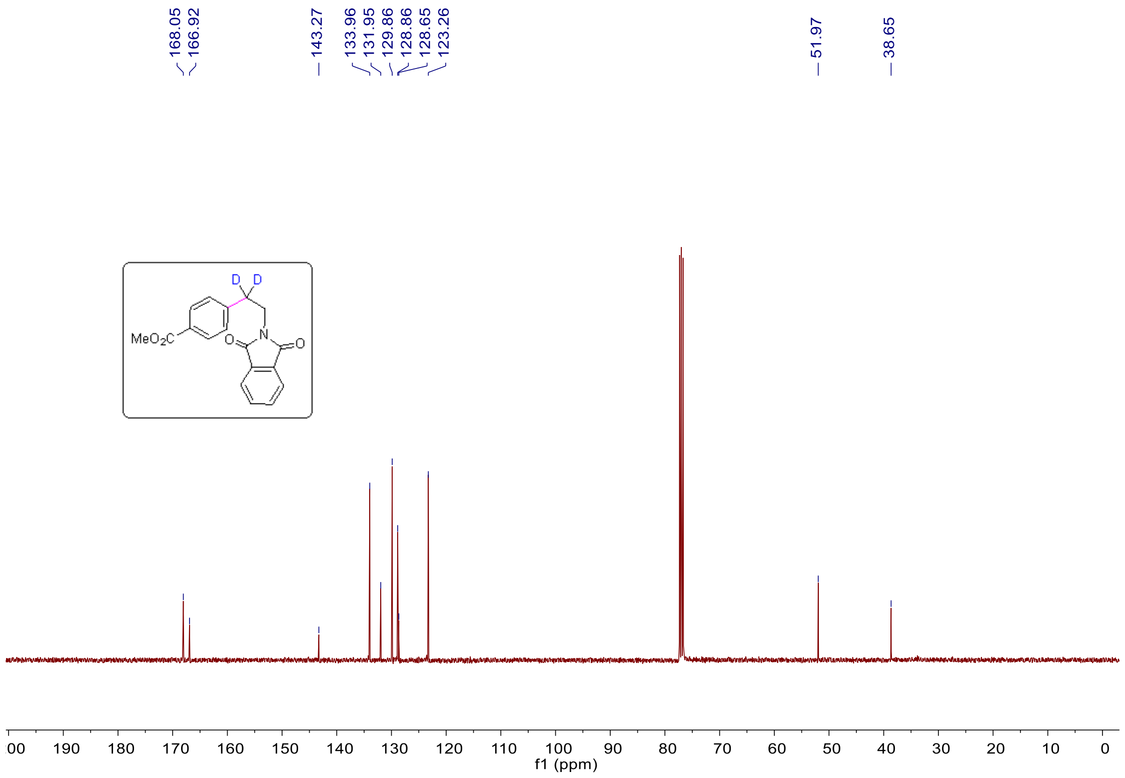

Supplementary Figure 169. ^{13}C NMR (101 MHz, $CDCl_3$) of **5fa**

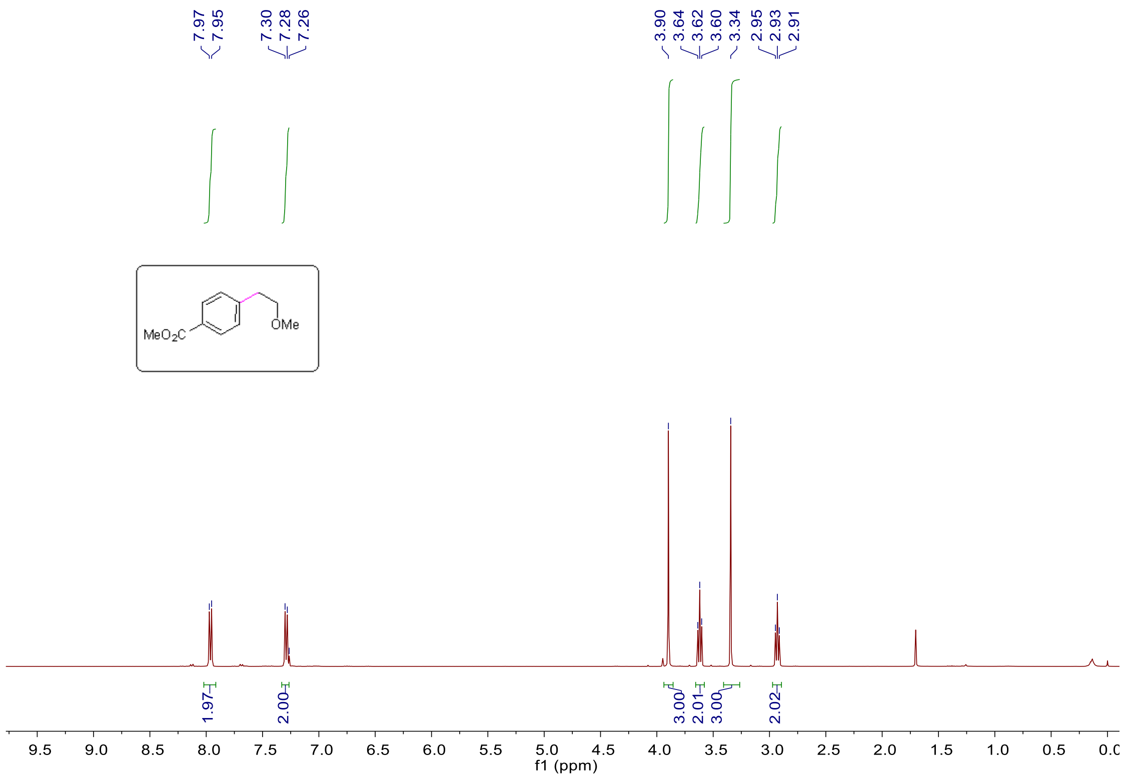

Supplementary Figure 170. ^{1}H NMR (400 MHz, $CDCl_3$) of **5ga'**

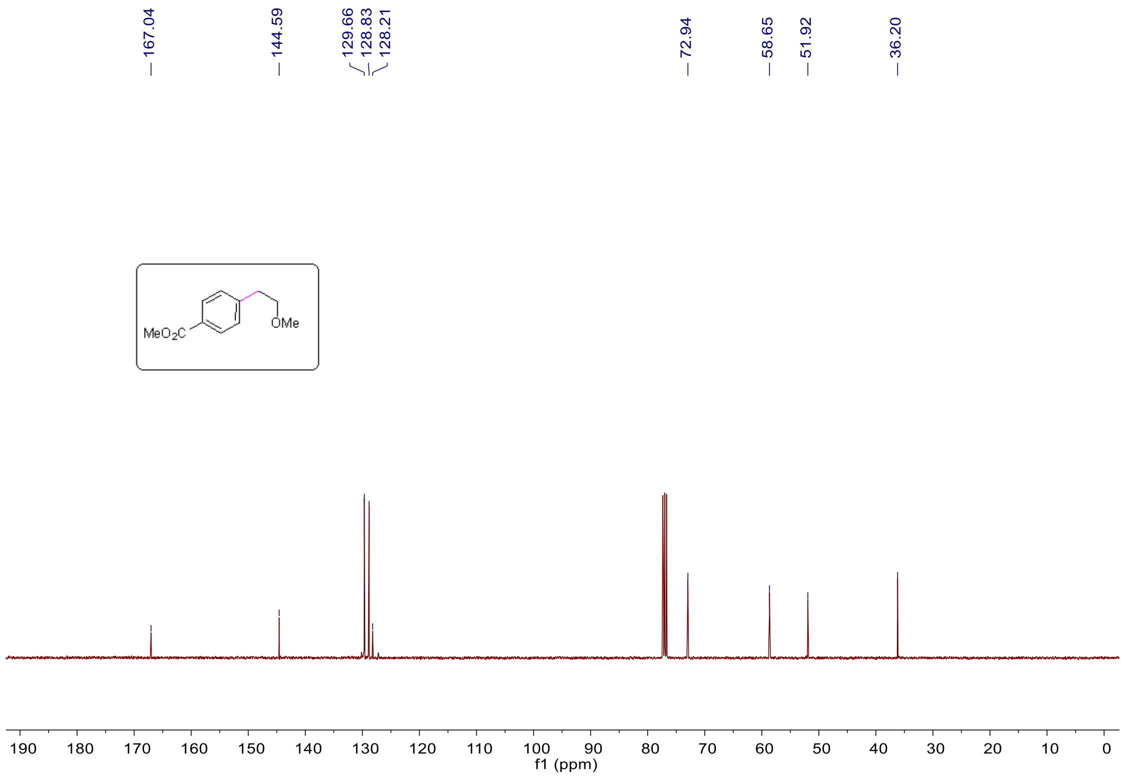

Supplementary Figure 171. ^{13}C NMR (101 MHz, $CDCl_3$) of **5ga'**

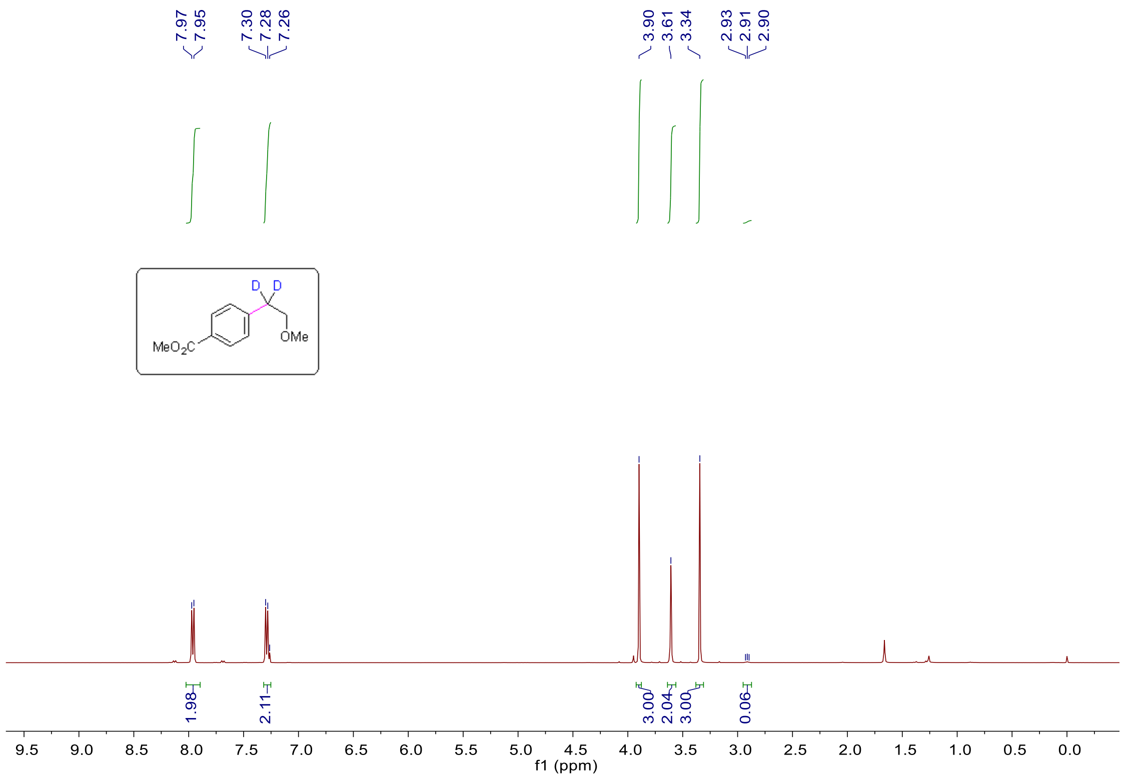

Supplementary Figure 172. ^{1}H NMR (400 MHz, $CDCl_3$) of **5ga**

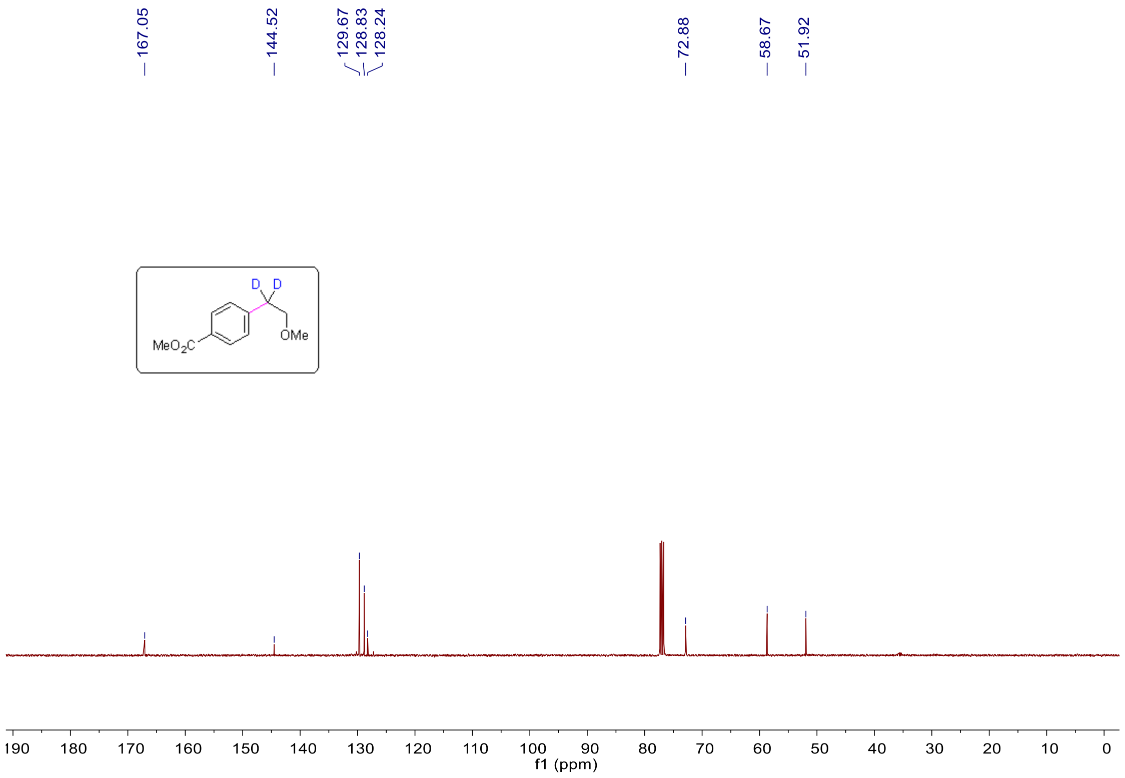

Supplementary Figure 173. ^{13}C NMR (101 MHz, $CDCl_3$) of **5ga**

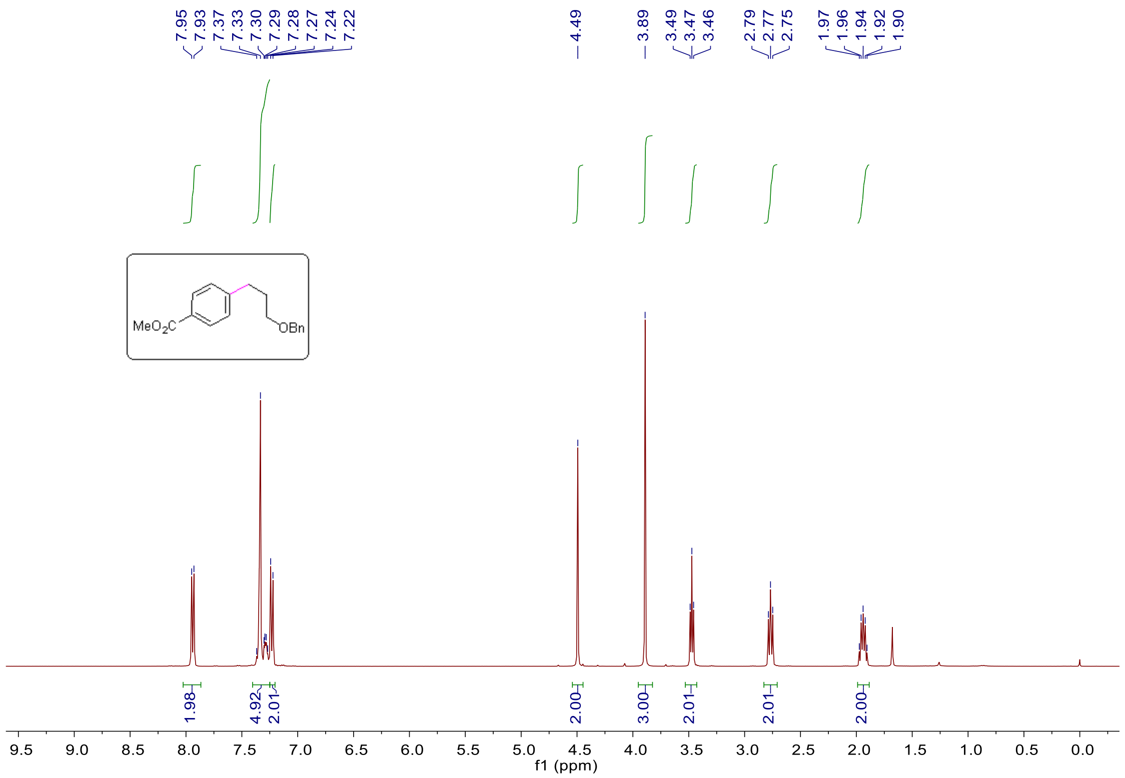

Supplementary Figure 174. ^{1}H NMR (400 MHz, $CDCl_3$) of **5ha'**

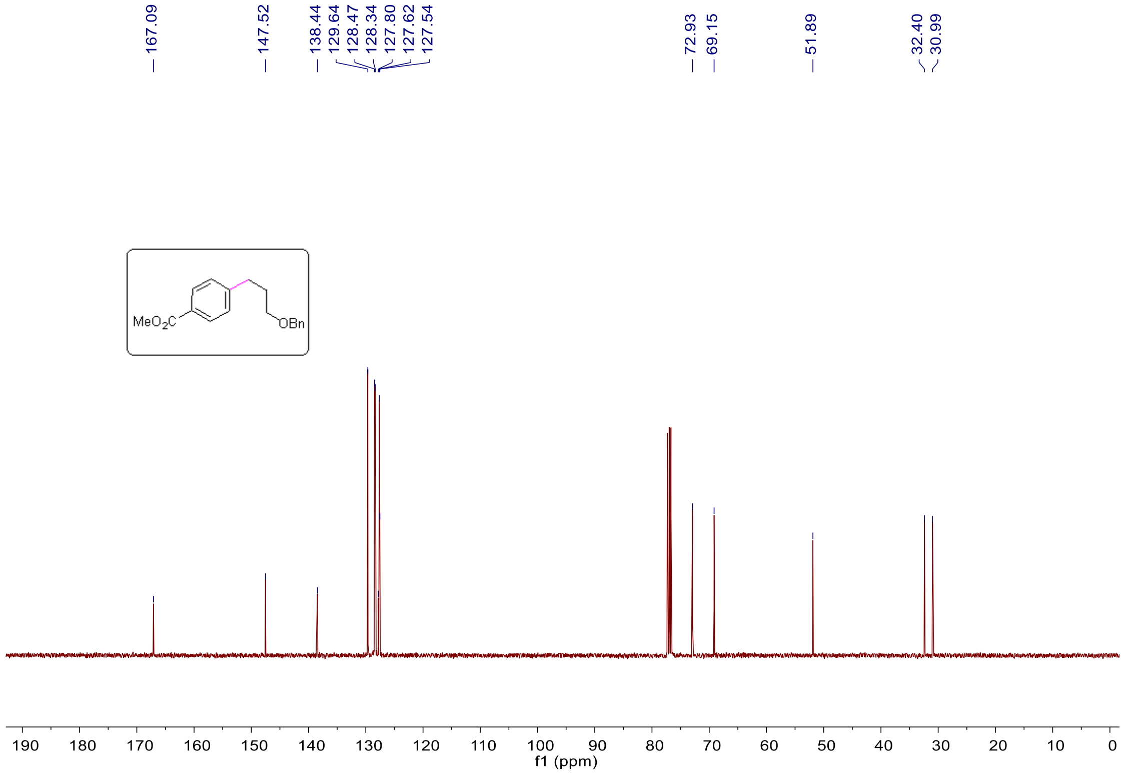

Supplementary Figure 175. ^{13}C NMR (101 MHz, $CDCl_3$) of **5ha'**

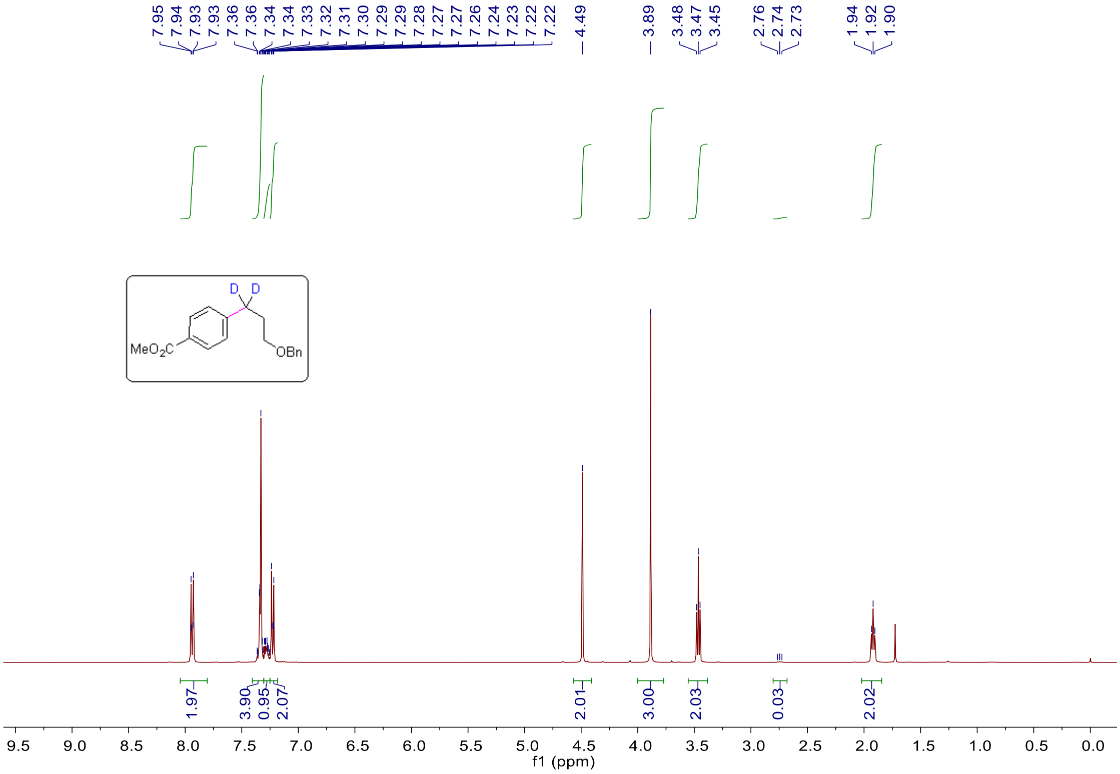

Supplementary Figure 176. ^{1}H NMR (400 MHz, $CDCl_3$) of **5ha**

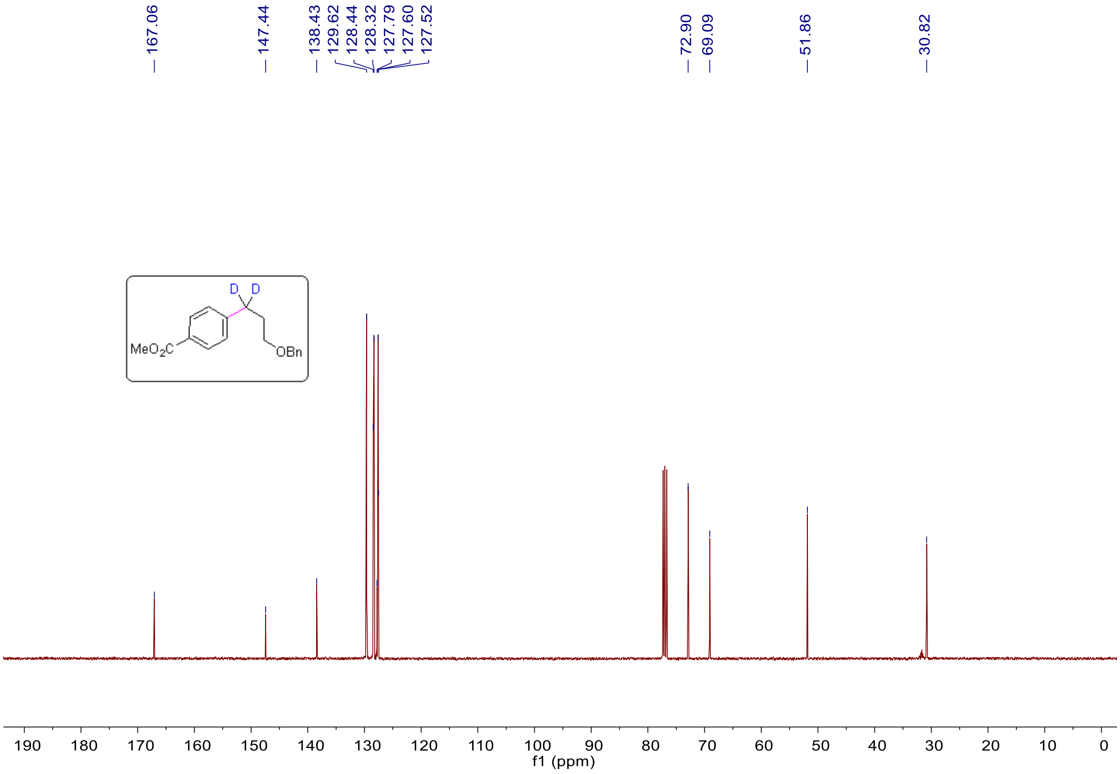

Supplementary Figure 177. ^{13}C NMR (101 MHz, $CDCl_3$) of **5ha**

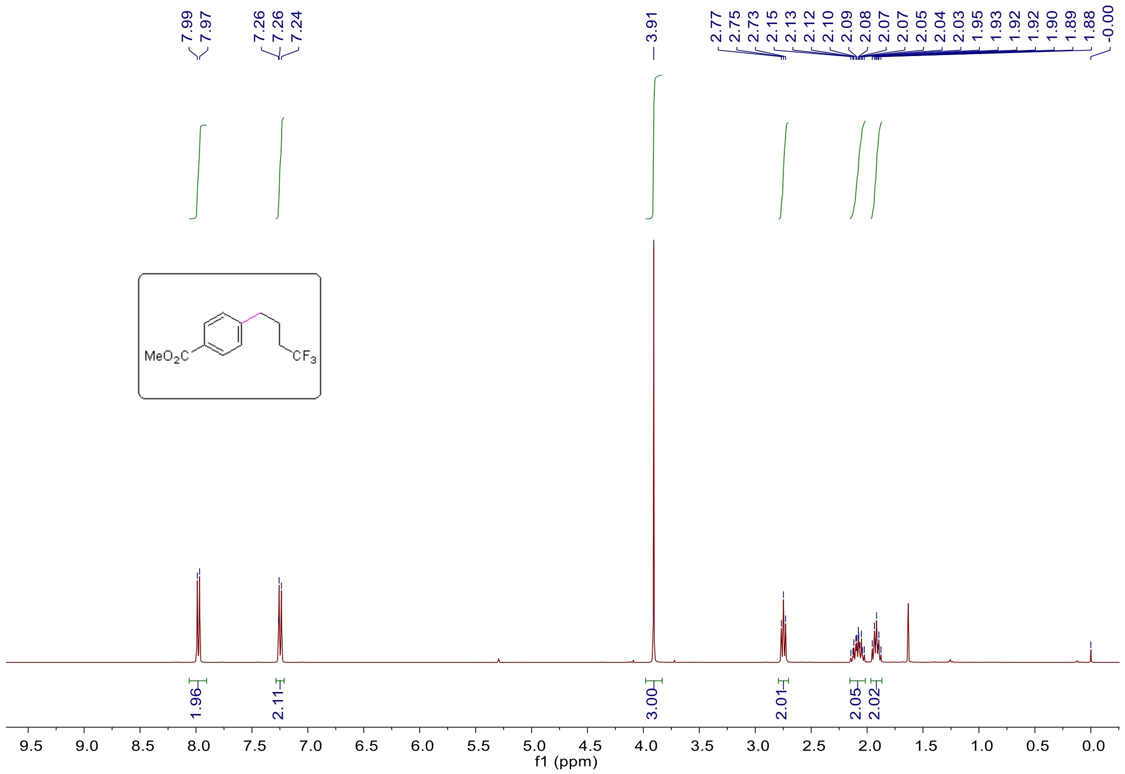

Supplementary Figure 178. ^{1}H NMR (400 MHz, $CDCl_3$) of **5ia'**

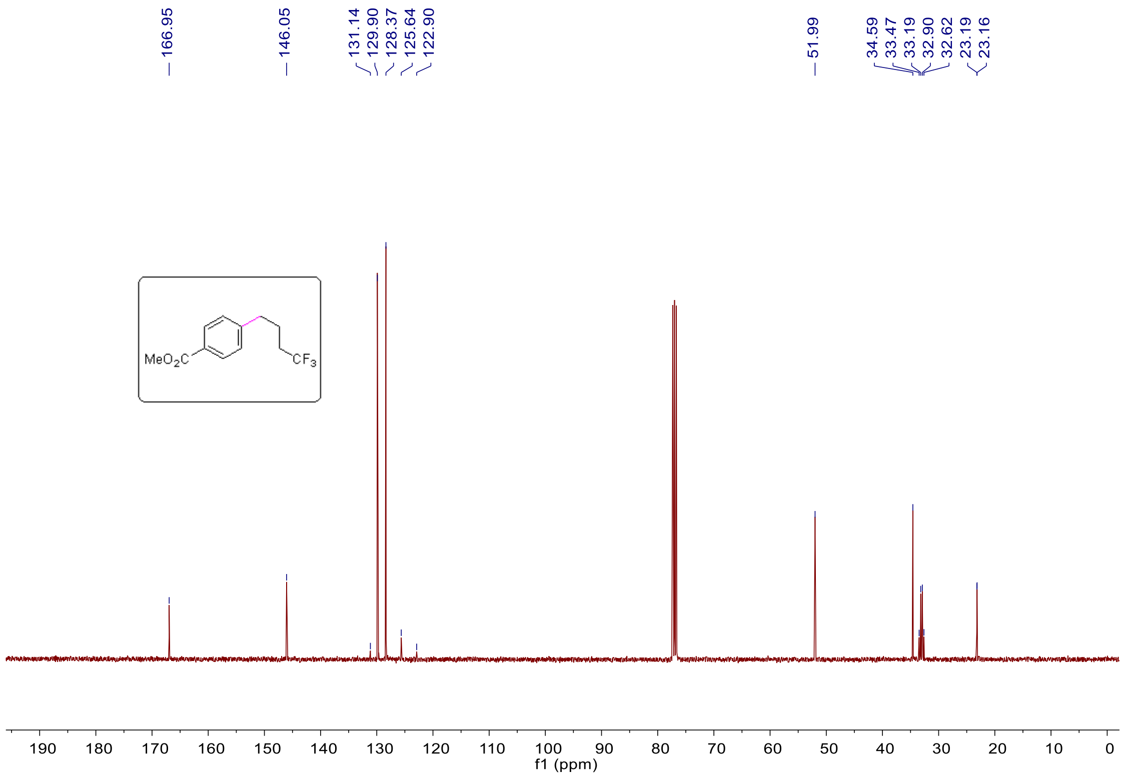

Supplementary Figure 179. ^{13}C NMR (101 MHz, $CDCl_3$) of **5ia'**

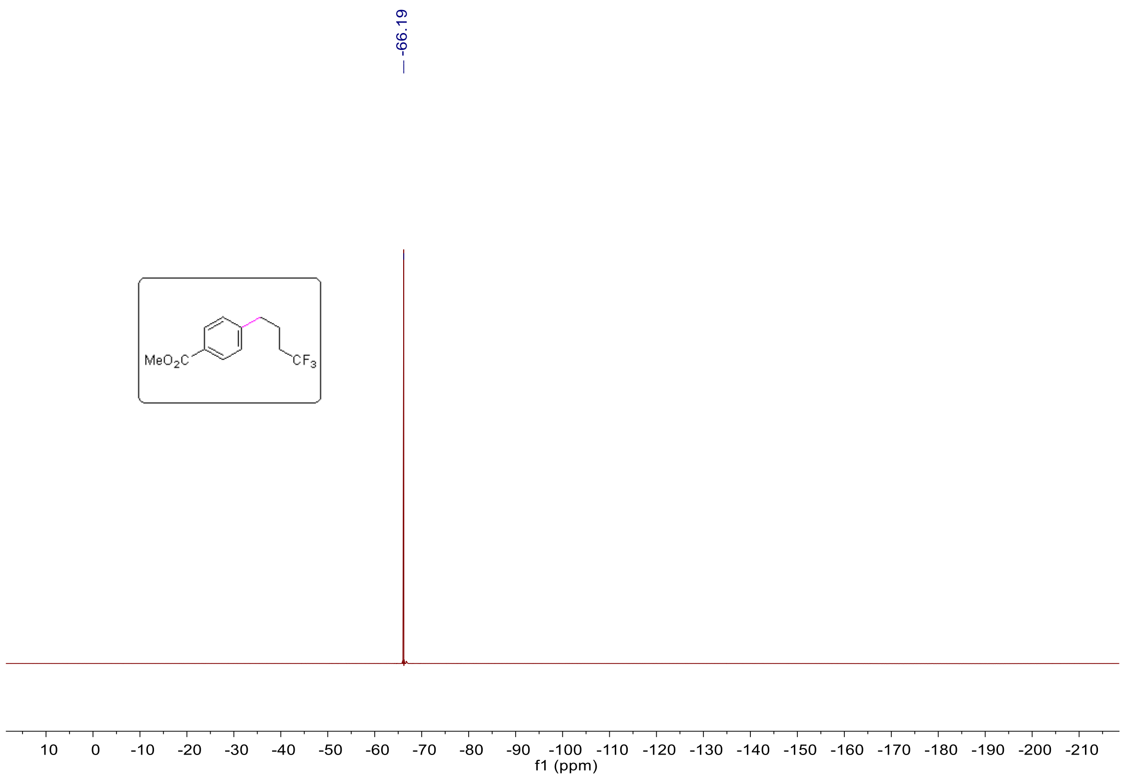

Supplementary Figure 180. ^{19}F NMR (376 MHz, $CDCl_3$) of **5ia'**

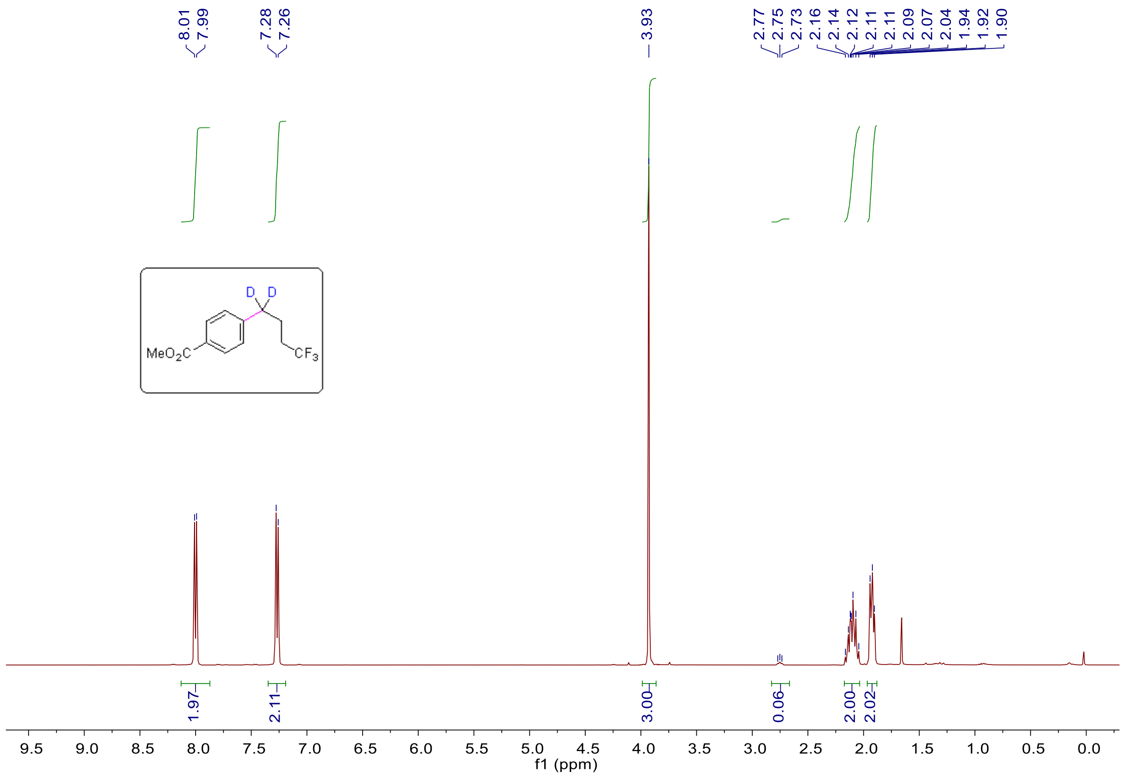

Supplementary Figure 181. ^{1}H NMR (400 MHz, $CDCl_3$) of **5ia**

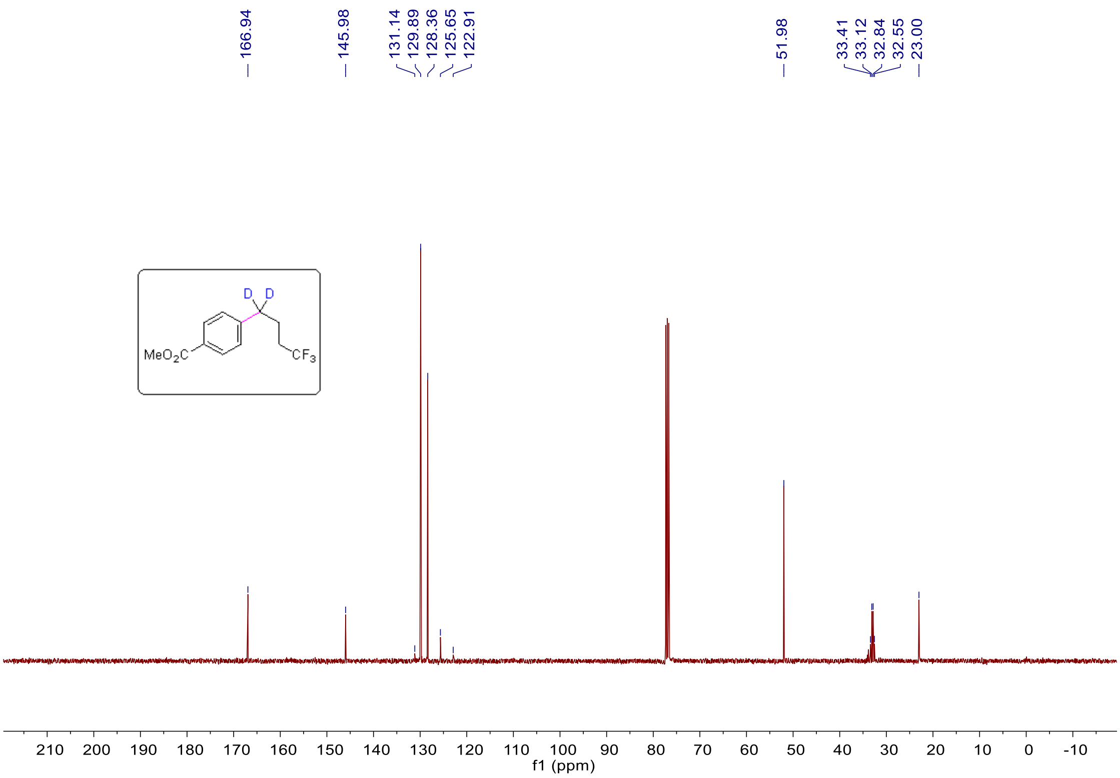

Supplementary Figure 182. ^{13}C NMR (101 MHz, $CDCl_3$) of **5ia**

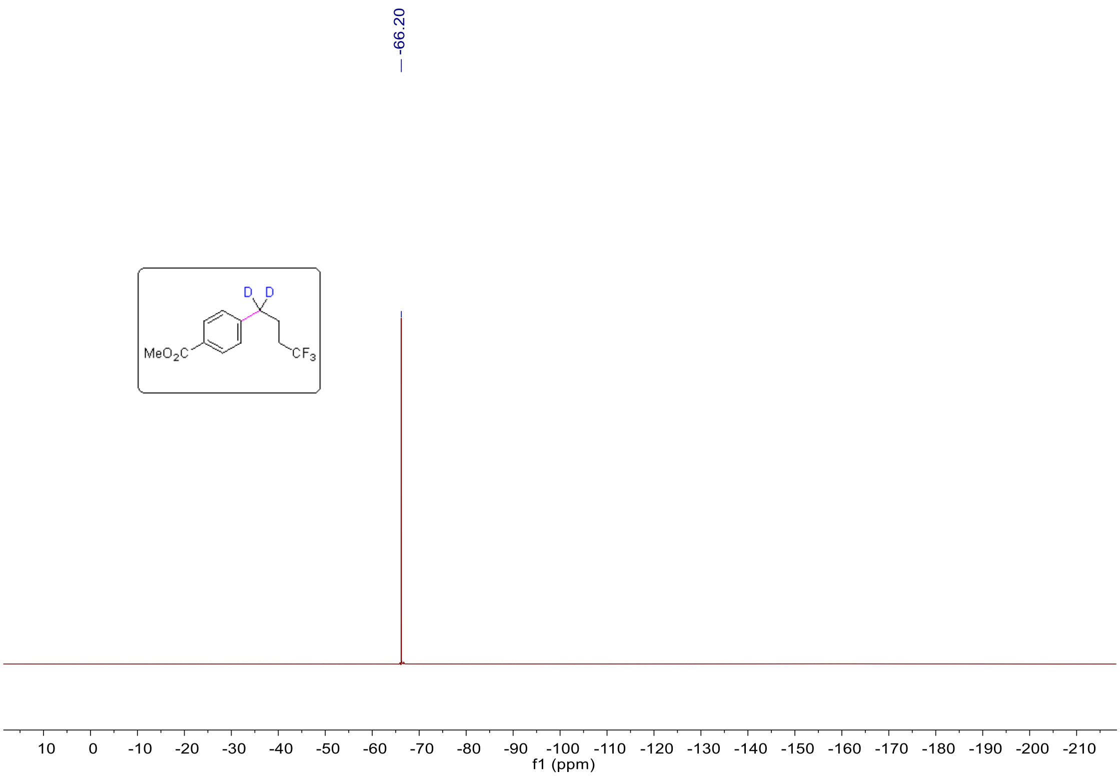

Supplementary Figure 183. ^{19}F NMR (376 MHz, $CDCl_3$) of **5ia**

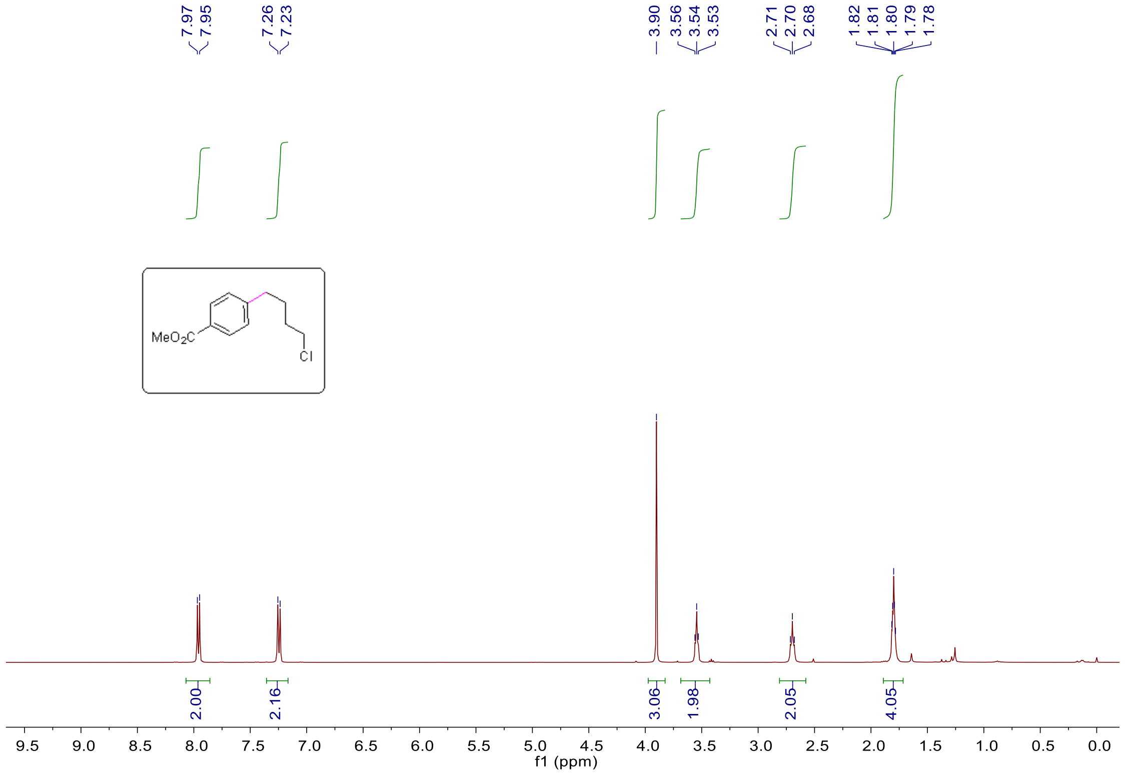

Supplementary Figure 184. ^{1}H NMR (400 MHz, $CDCl_3$) of **5ja'**

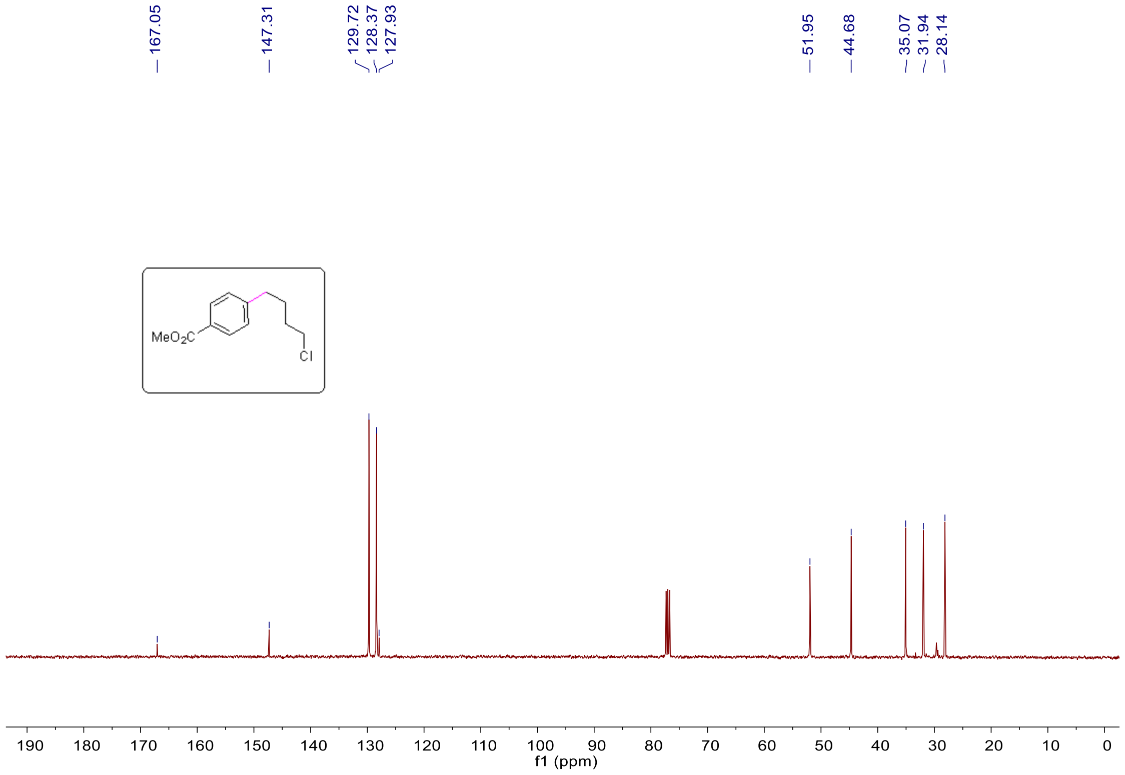

Supplementary Figure 185. ^{13}C NMR (101 MHz, $CDCl_3$) of **5ja'**

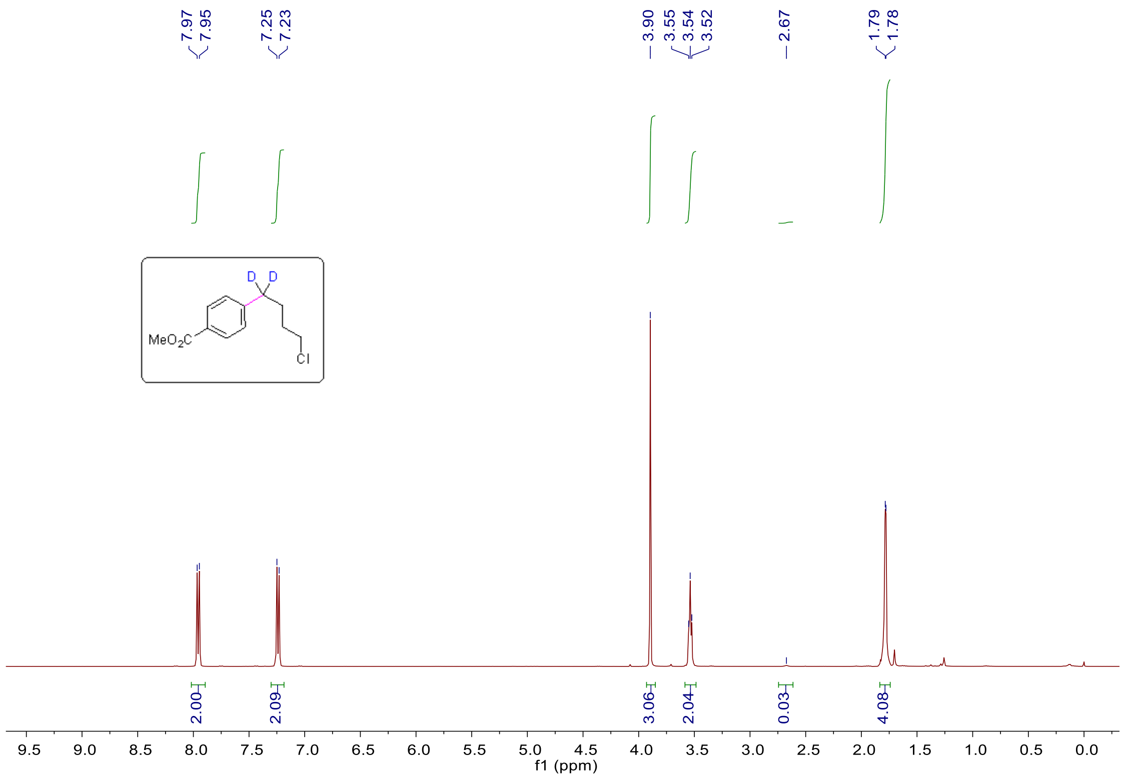

Supplementary Figure 186. ^{1}H NMR (400 MHz, $CDCl_3$) of **5ja**

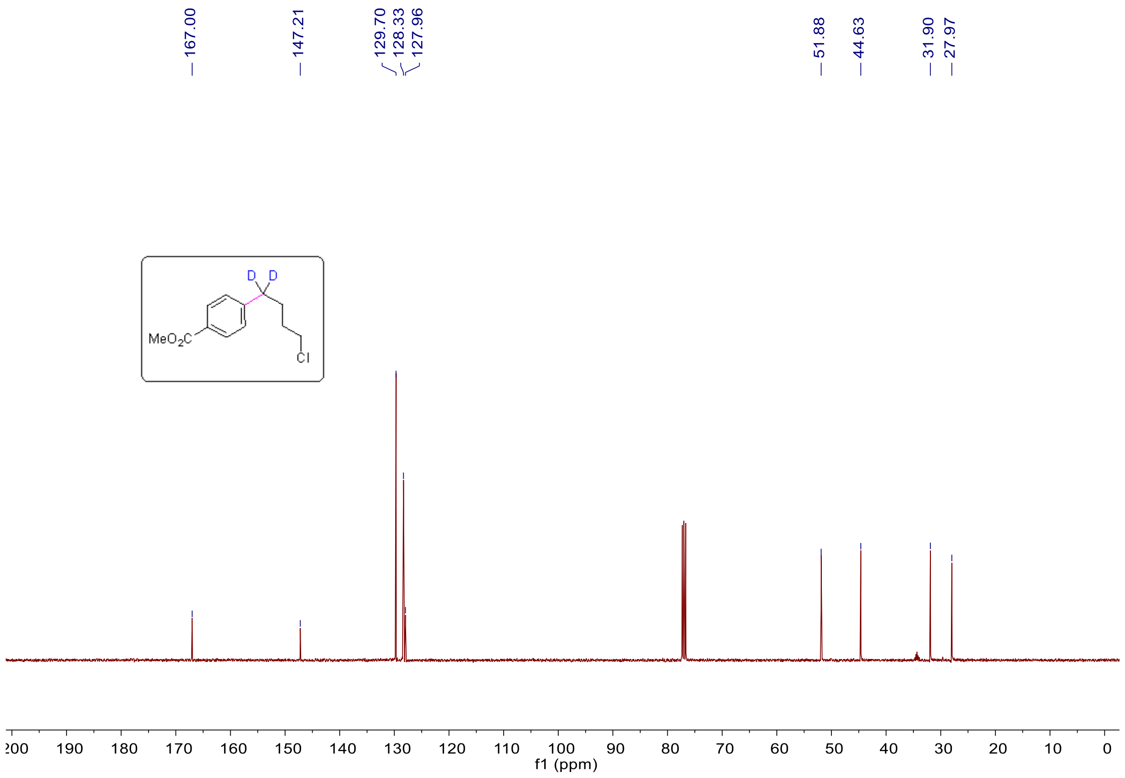

Supplementary Figure 187. ^{13}C NMR (101 MHz, $CDCl_3$) of **5ja**

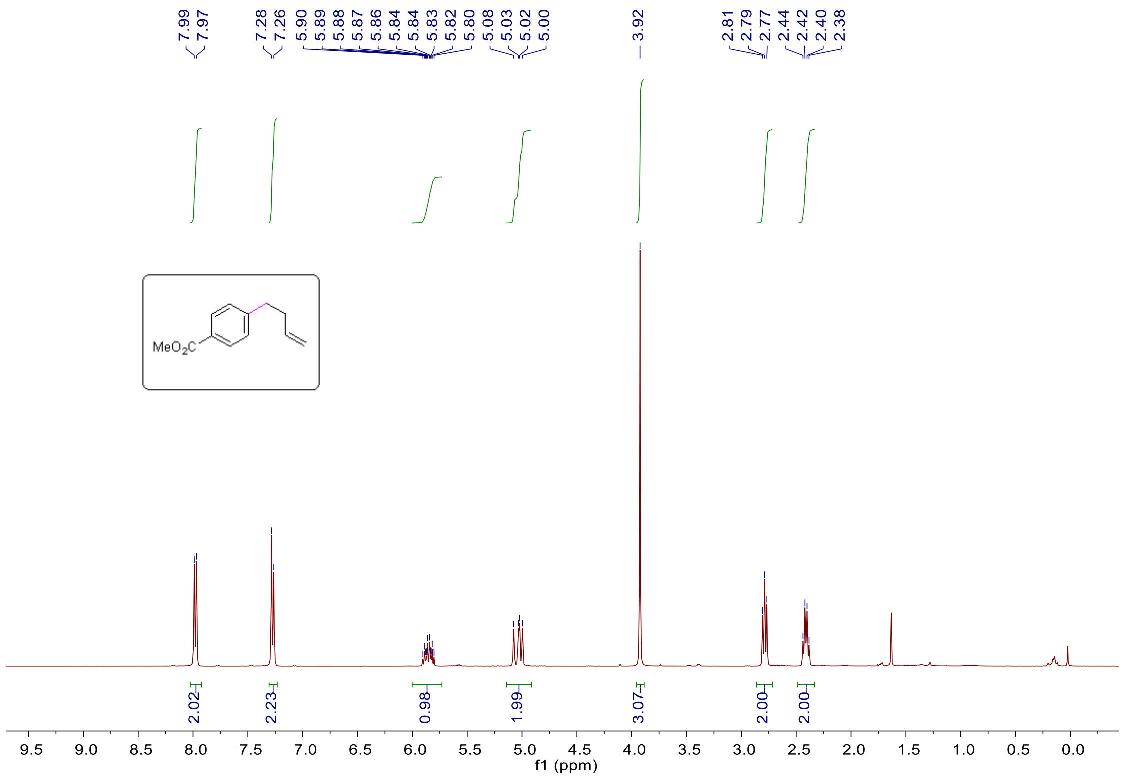

Supplementary Figure 188. ^{1}H NMR (400 MHz, $CDCl_3$) of **5ka'**

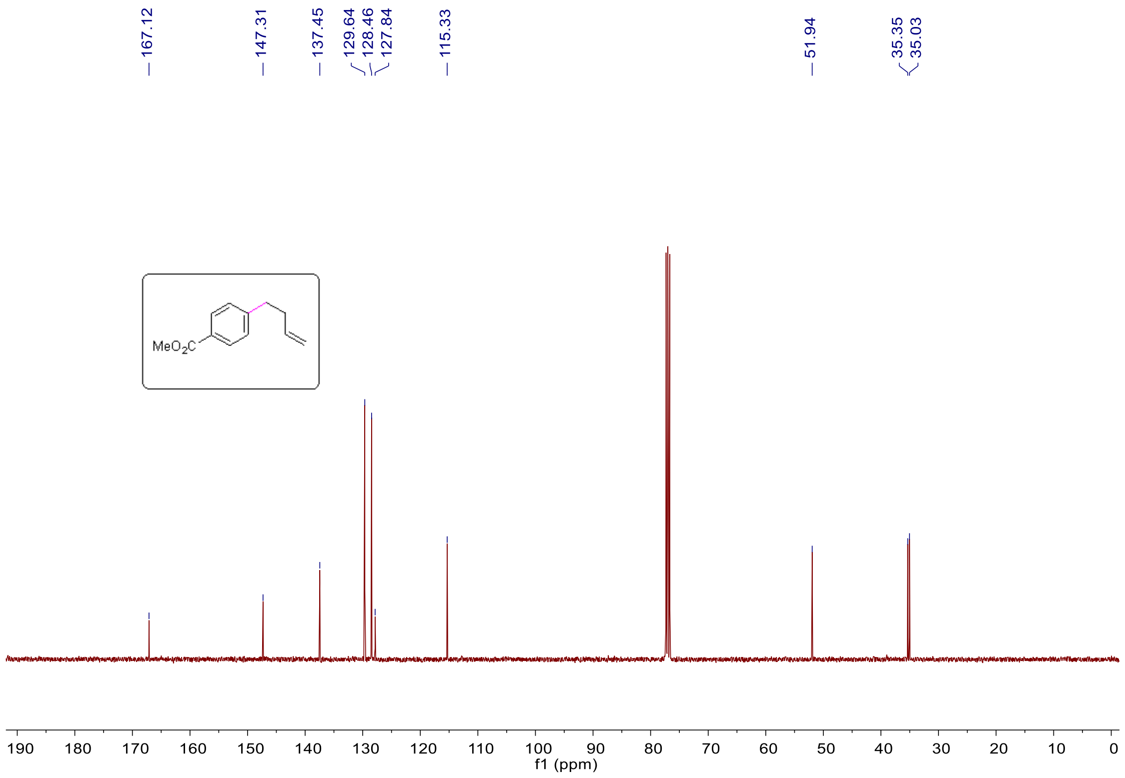

Supplementary Figure 189. ^{13}C NMR (101 MHz, $CDCl_3$) of **5ka'**

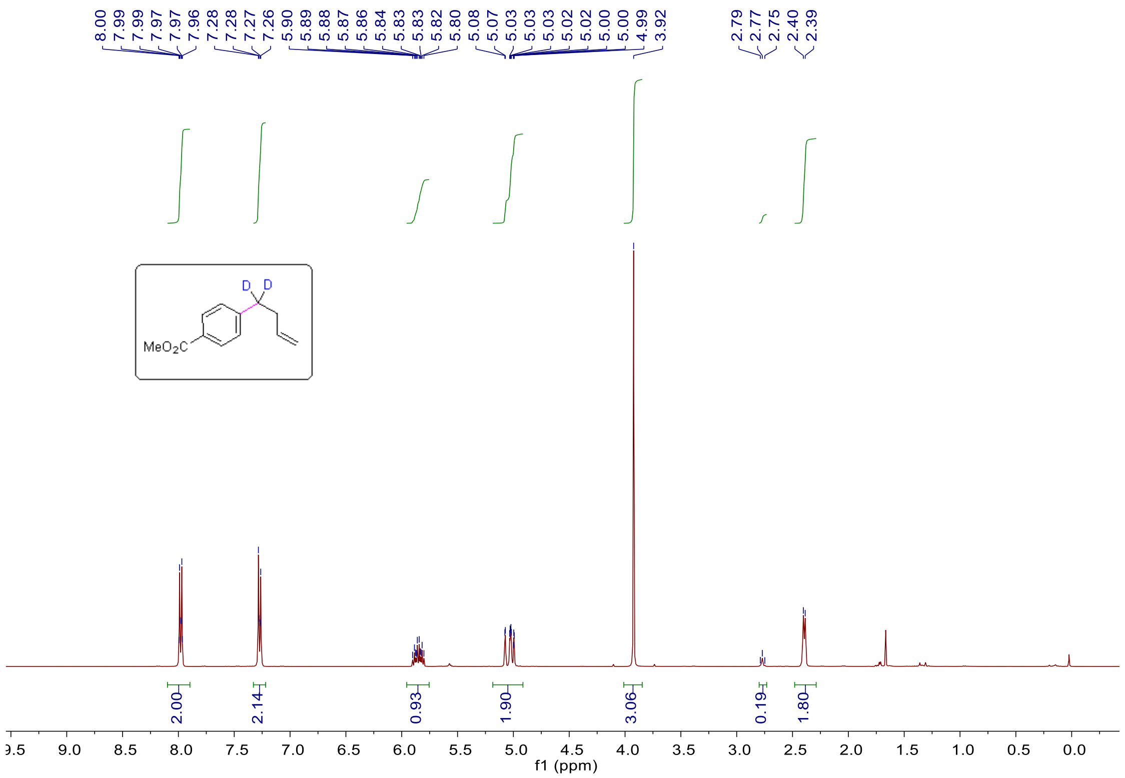

Supplementary Figure 190. ^{1}H NMR (400 MHz, $CDCl_3$) of **5ka**

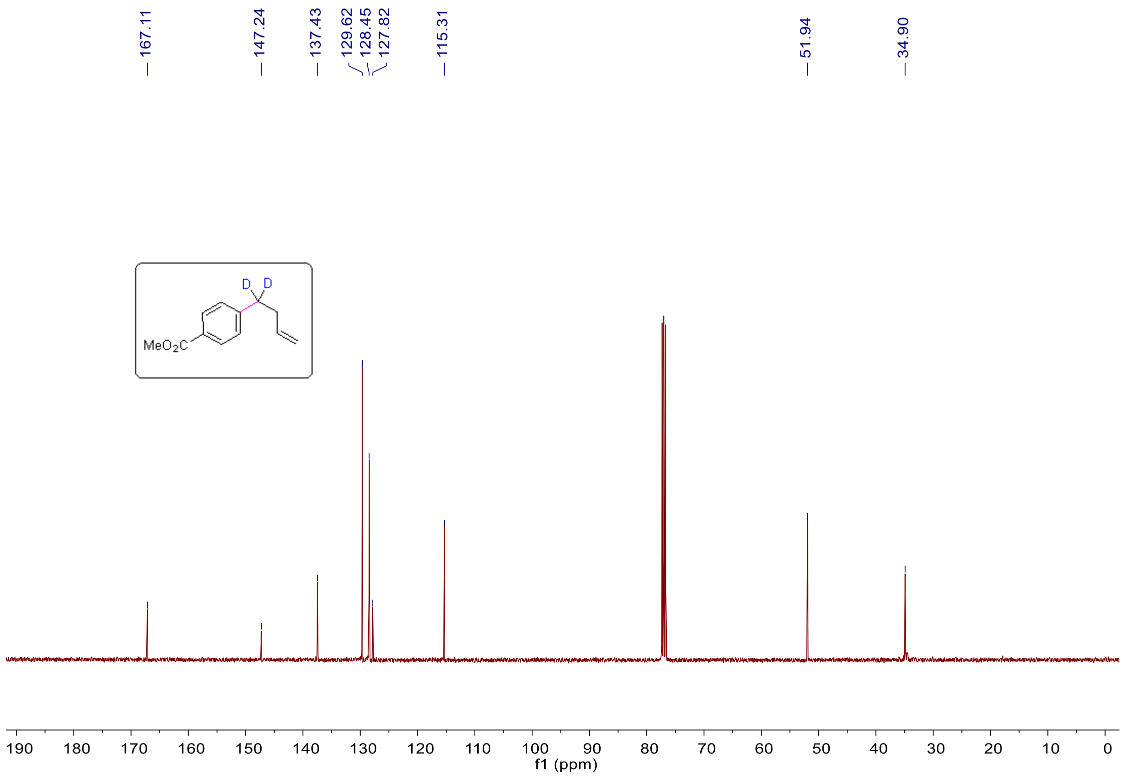

Supplementary Figure 191. ^{13}C NMR (101 MHz, $CDCl_3$) of **5ka**

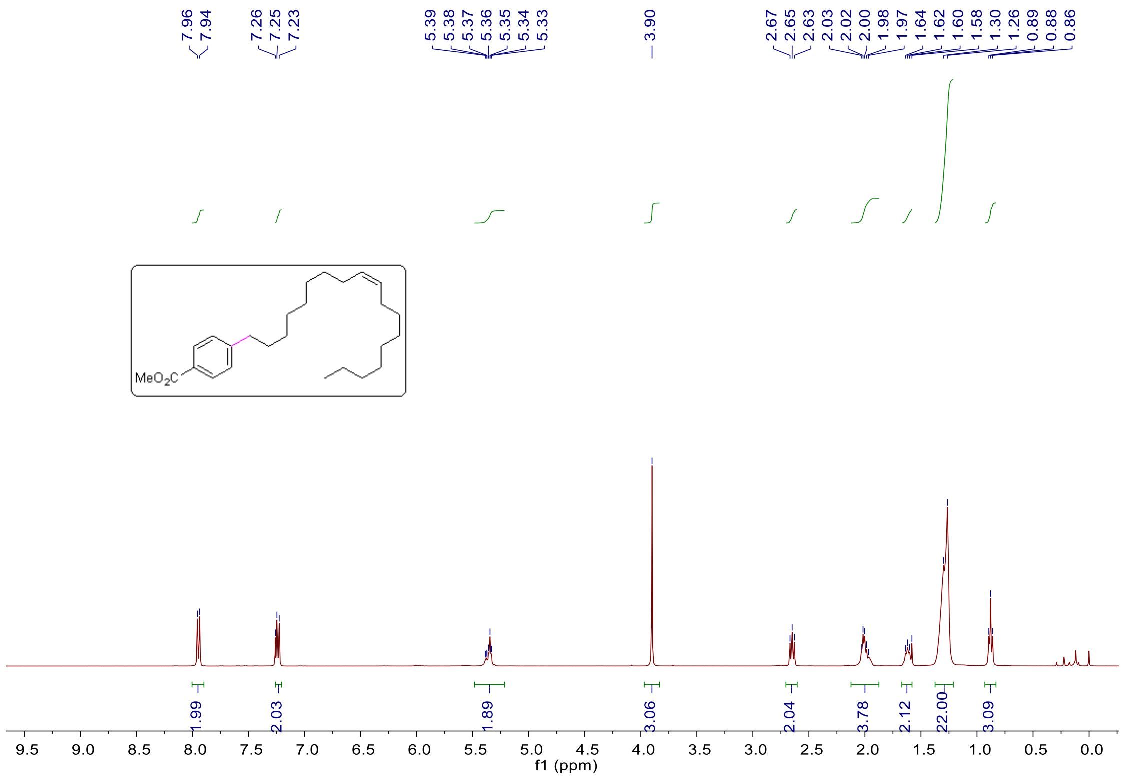

Supplementary Figure 192. ^{1}H NMR (400 MHz, CDCl$_3$) of **5la'**

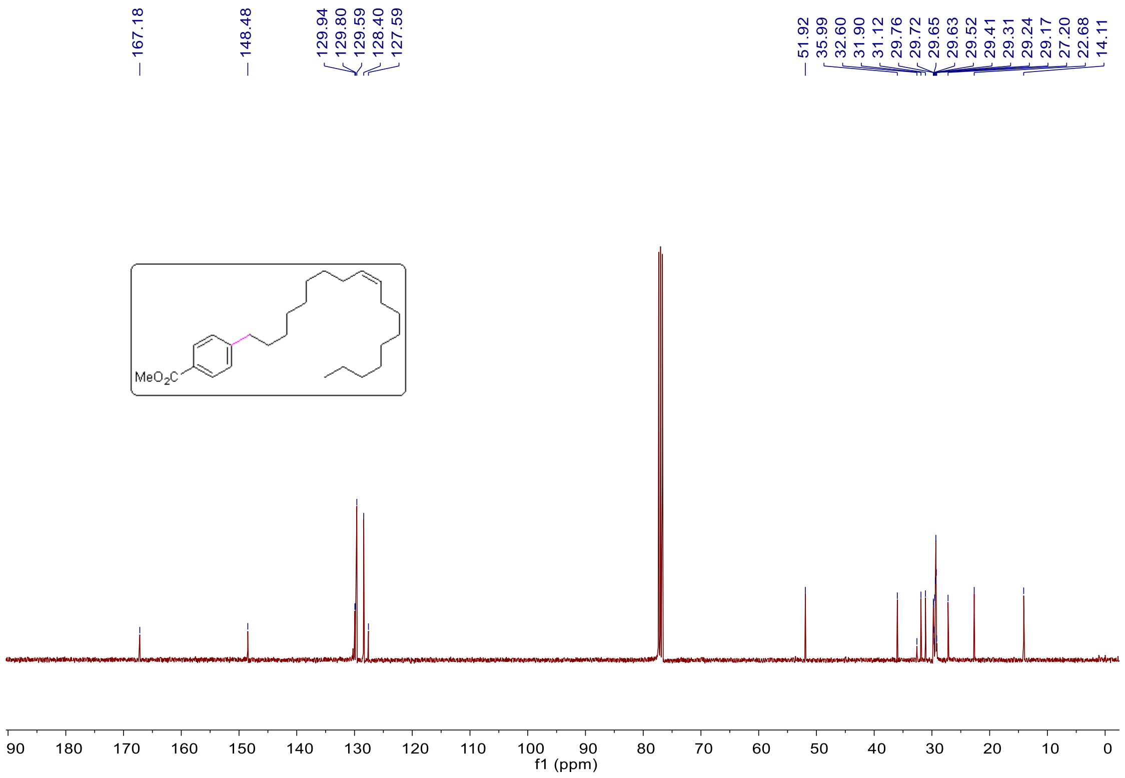

Supplementary Figure 193. ^{13}C NMR (101 MHz, CDCl$_3$) of **5la'**

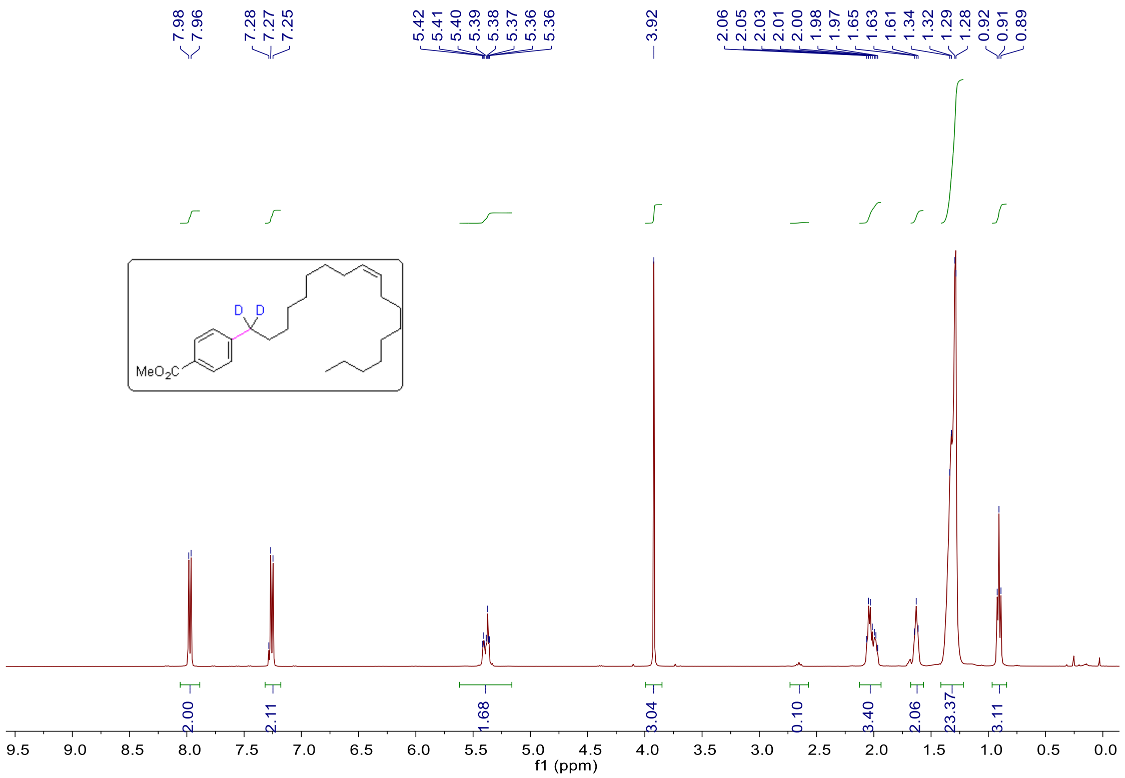

Supplementary Figure 194. ^{1}H NMR (400 MHz, $CDCl_3$) of **5la**

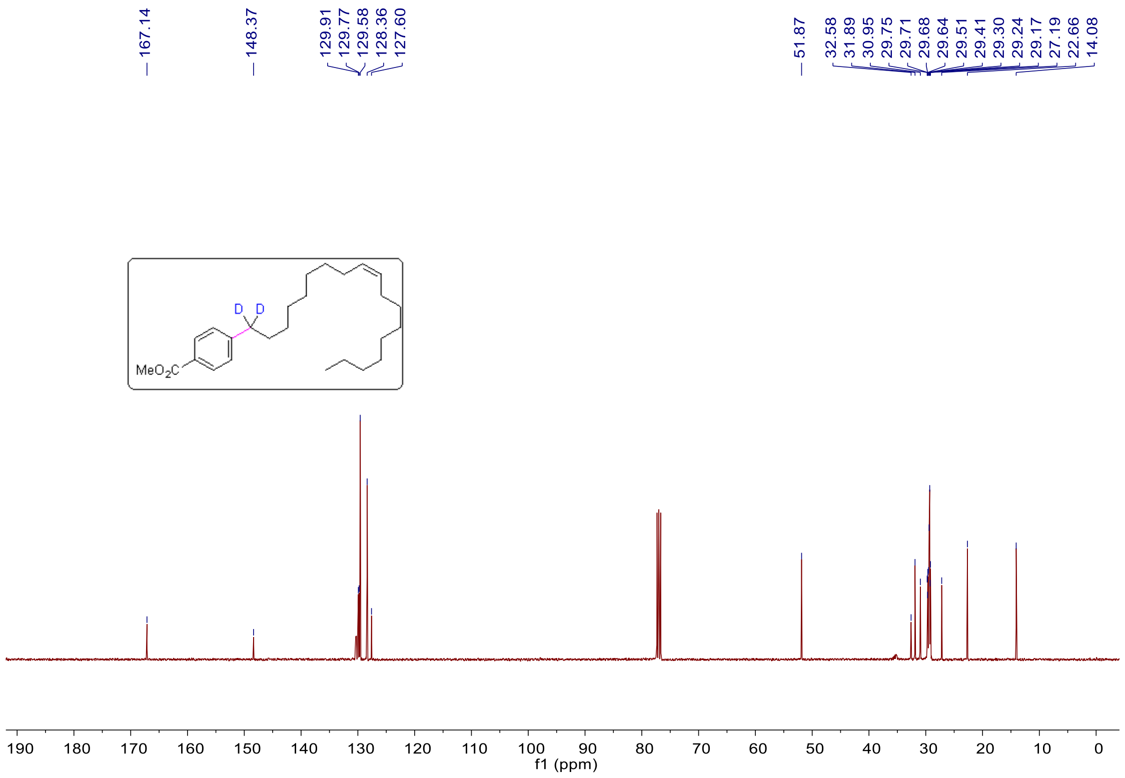

Supplementary Figure 195. ^{13}C NMR (101 MHz, $CDCl_3$) of **5la**

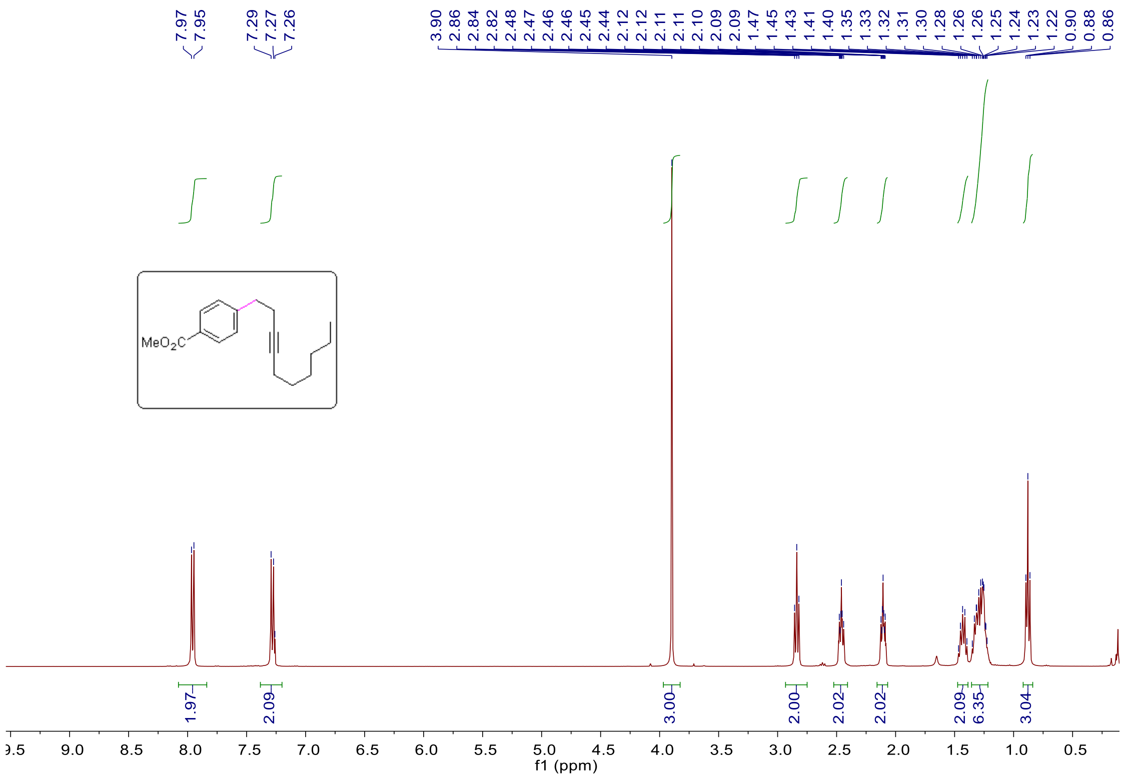

Supplementary Figure 196. ^{1}H NMR (400 MHz, $CDCl_3$) of **5ma'**

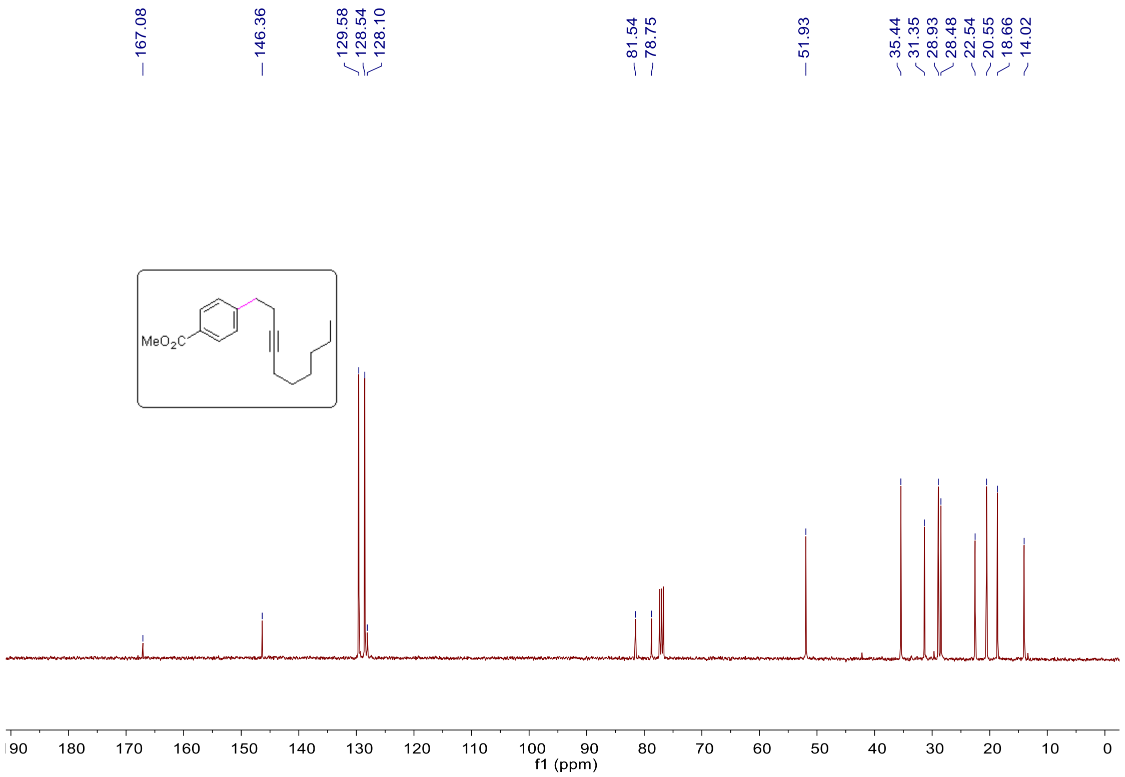

Supplementary Figure 197. ^{13}C NMR (101 MHz, $CDCl_3$) of **5ma'**

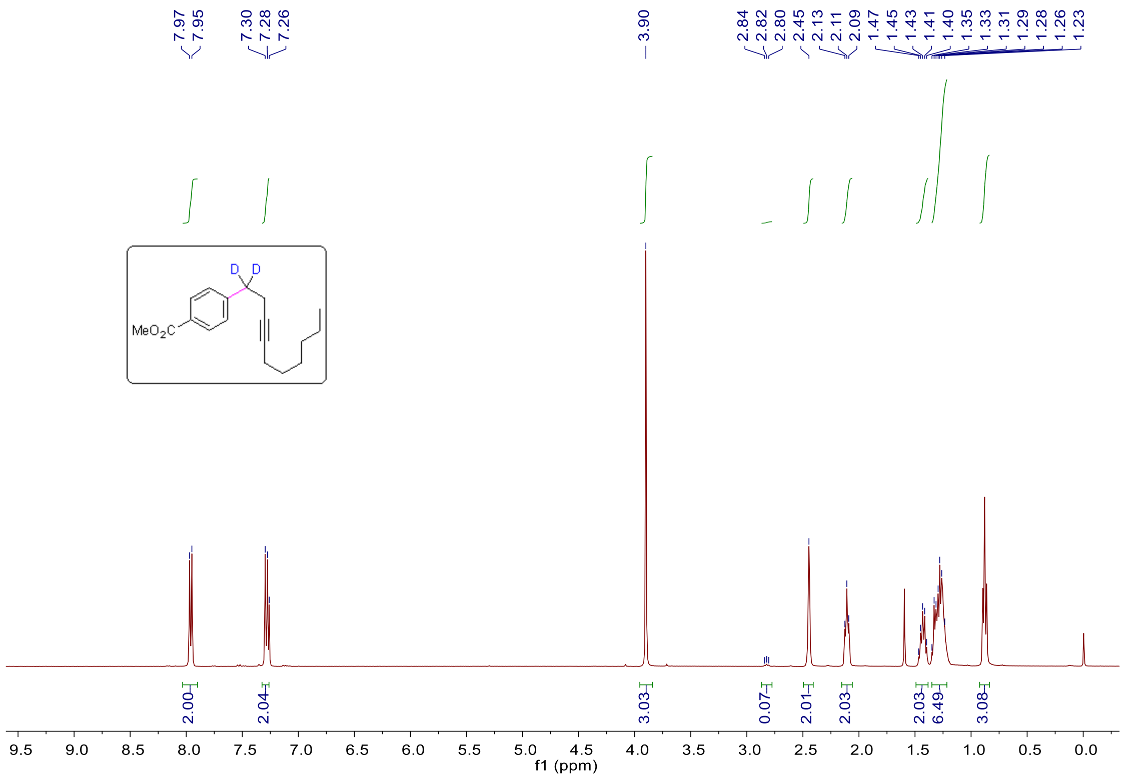

Supplementary Figure 198. ^{1}H NMR (400 MHz, $CDCl_3$) of **5ma**

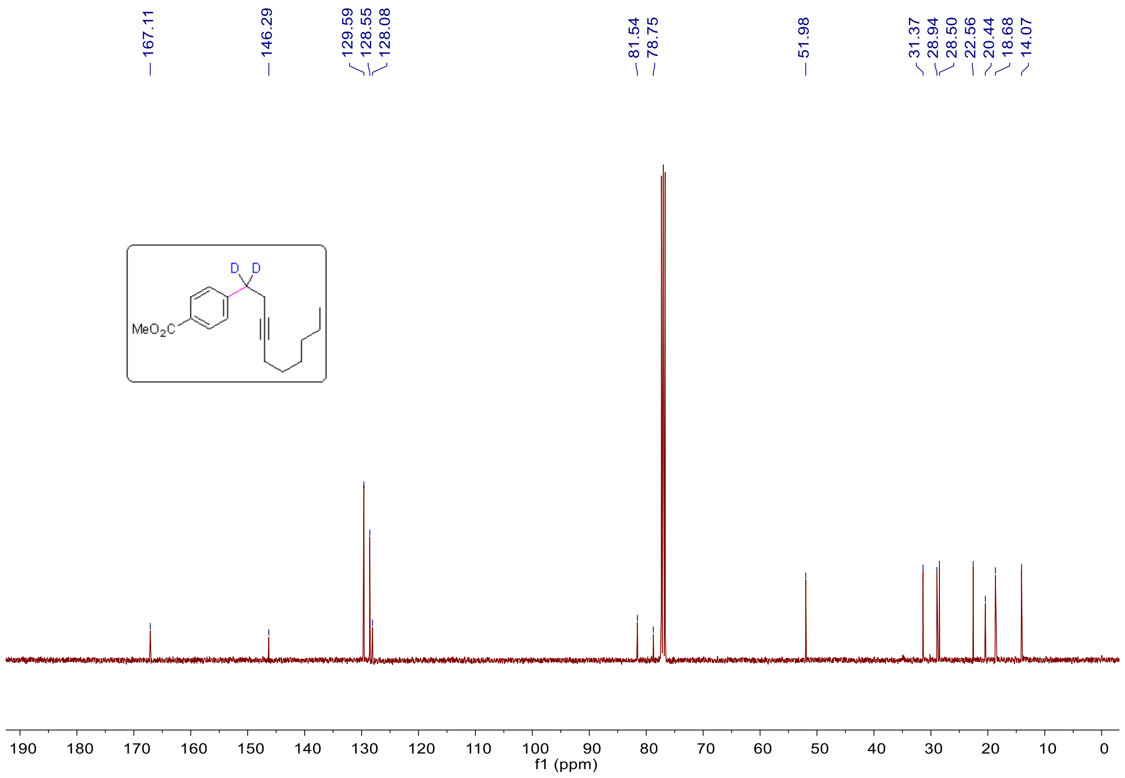

Supplementary Figure 199. ^{13}C NMR (101 MHz, $CDCl_3$) of **5ma**

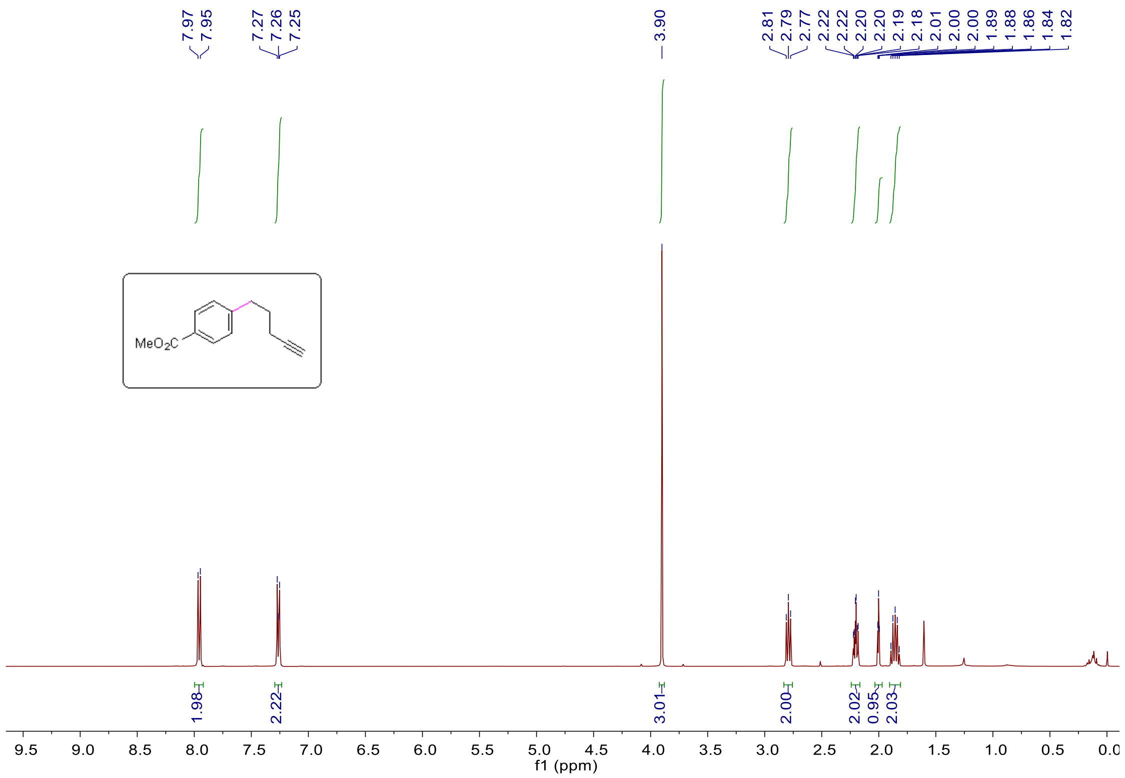

Supplementary Figure 200. ^{1}H NMR (400 MHz, $CDCl_3$) of **5na'**

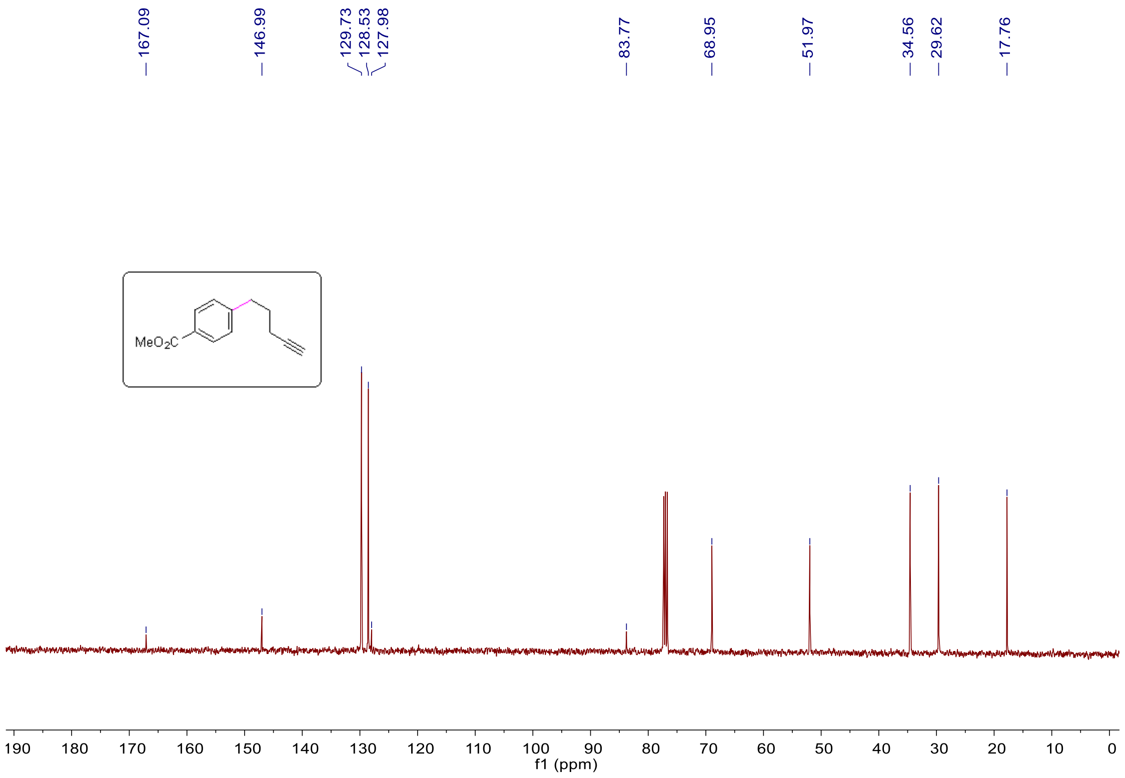

Supplementary Figure 201. ^{13}C NMR (101 MHz, $CDCl_3$) of **5na'**

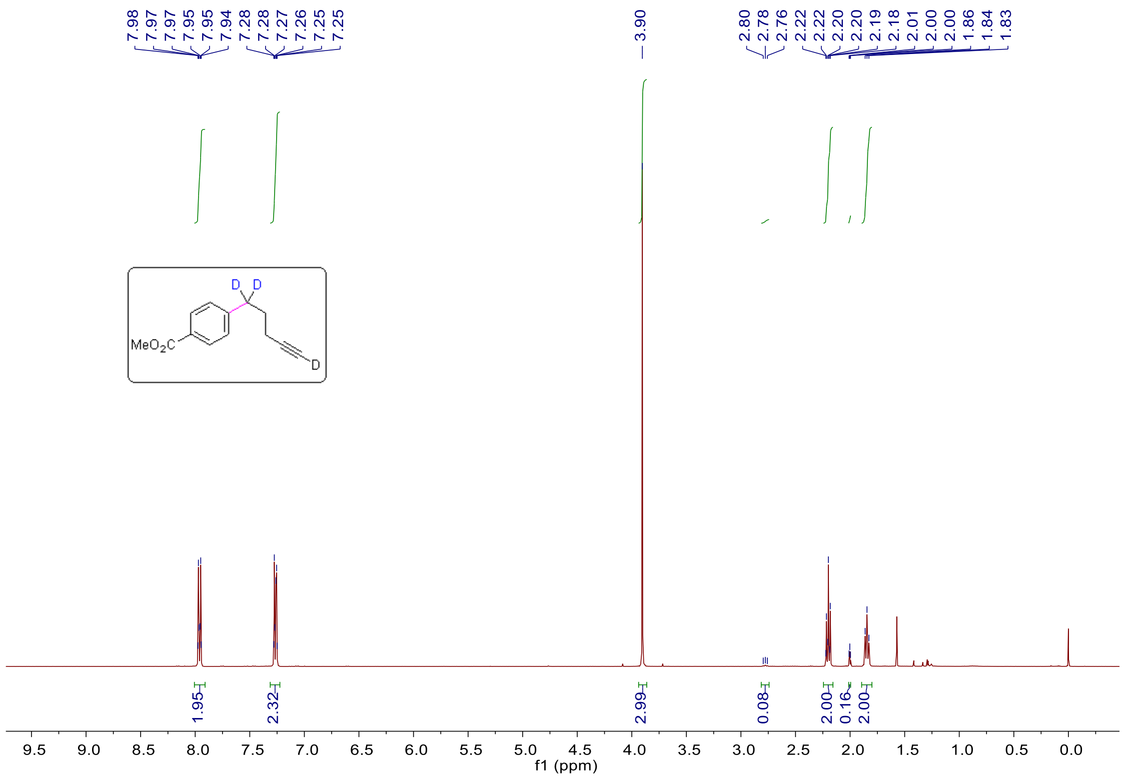

Supplementary Figure 202. ^{1}H NMR (400 MHz, $CDCl_3$) of **5na**

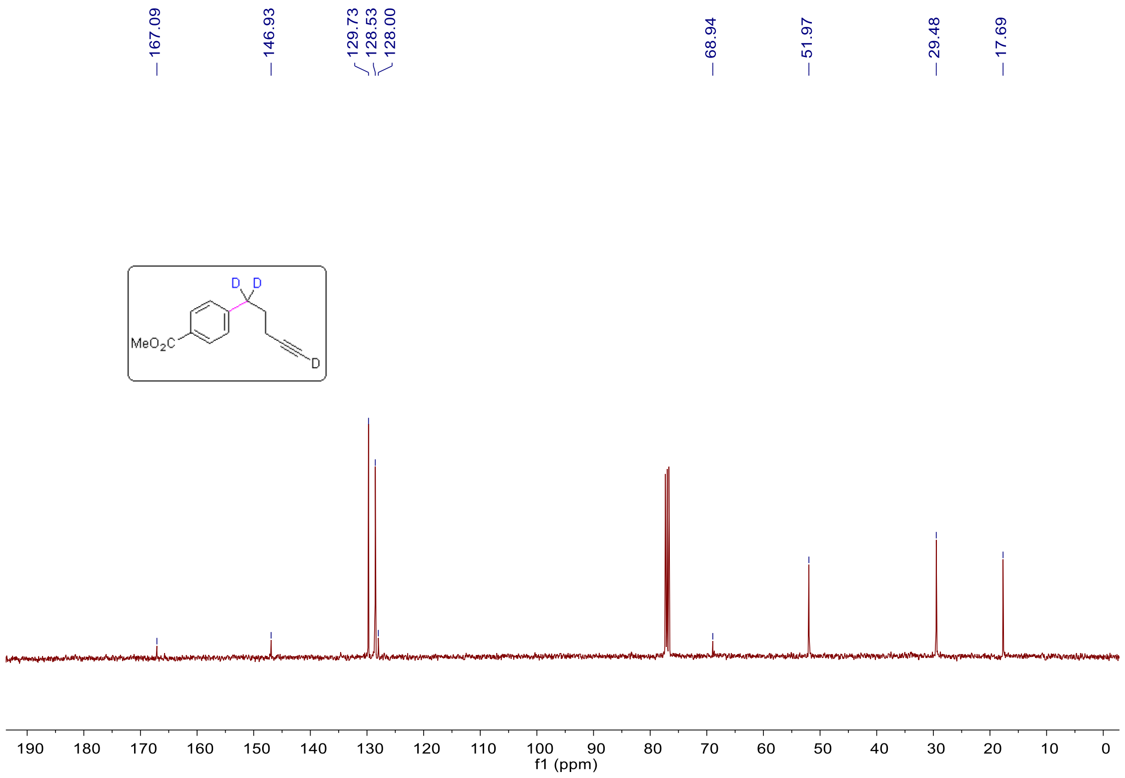

Supplementary Figure 203. ^{13}C NMR (101 MHz, $CDCl_3$) of **5na**

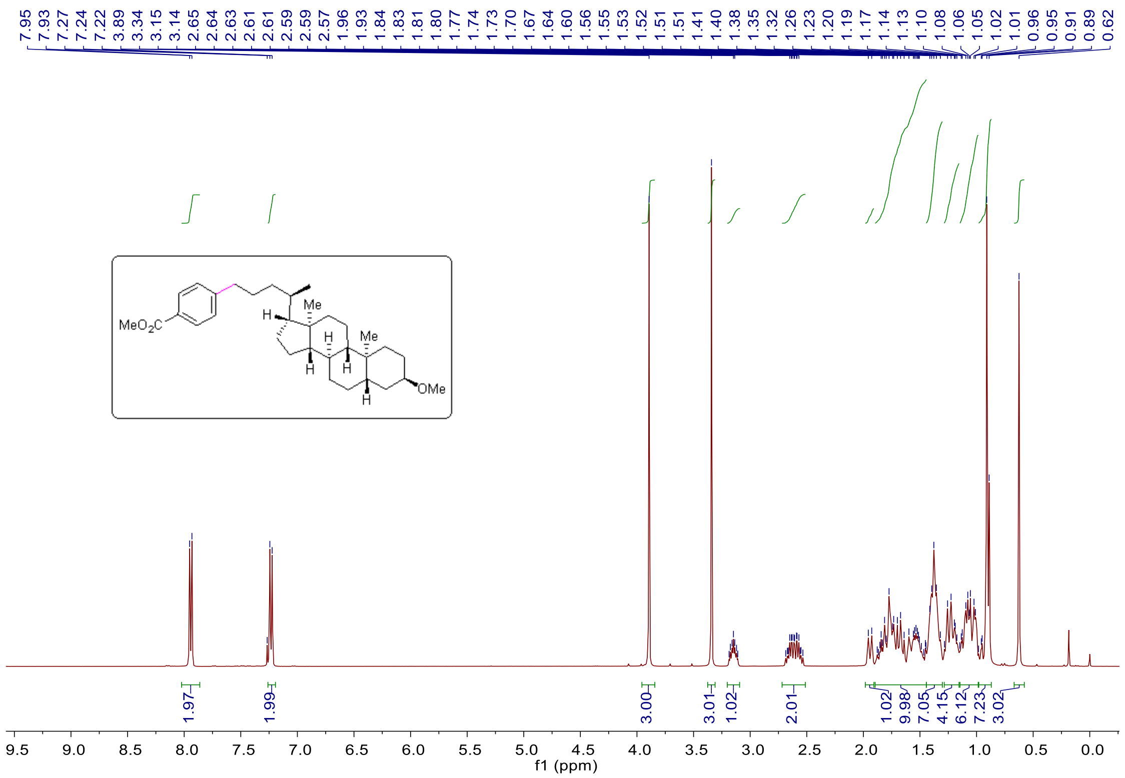

Supplementary Figure 204. ^{1}H NMR (400 MHz, $CDCl_3$) of **5oa'**

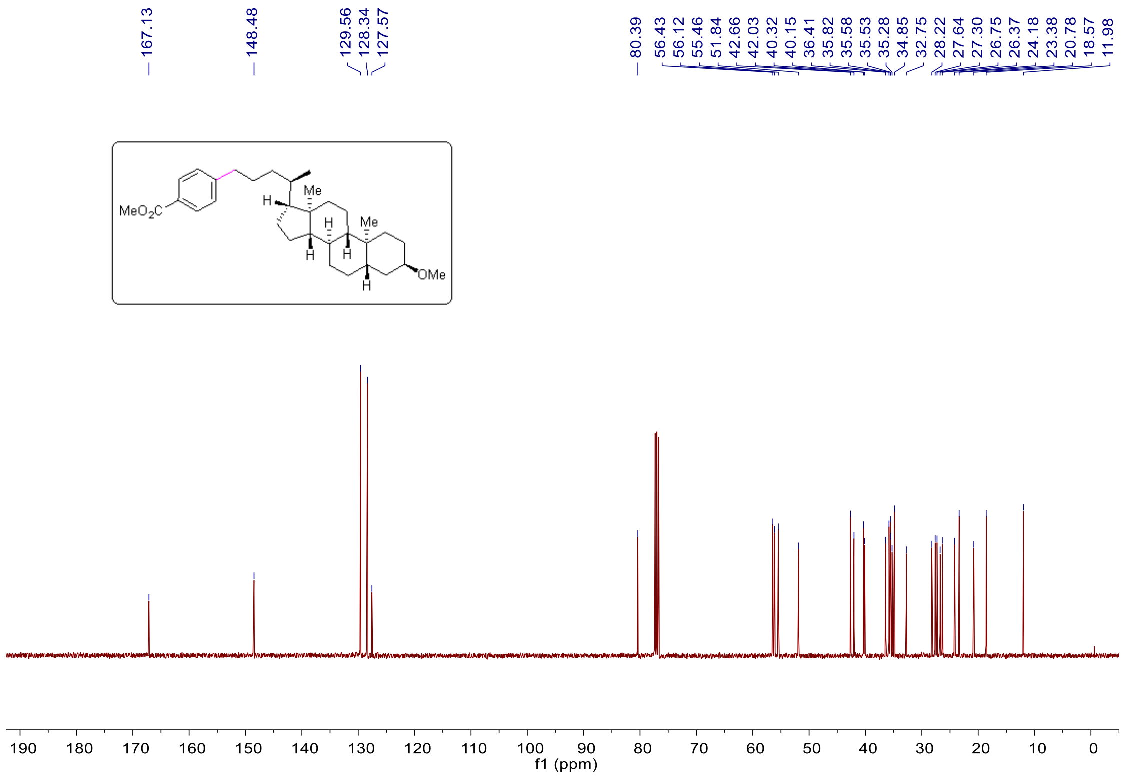

Supplementary Figure 205. ^{13}C NMR (101 MHz, $CDCl_3$) of **5oa'**

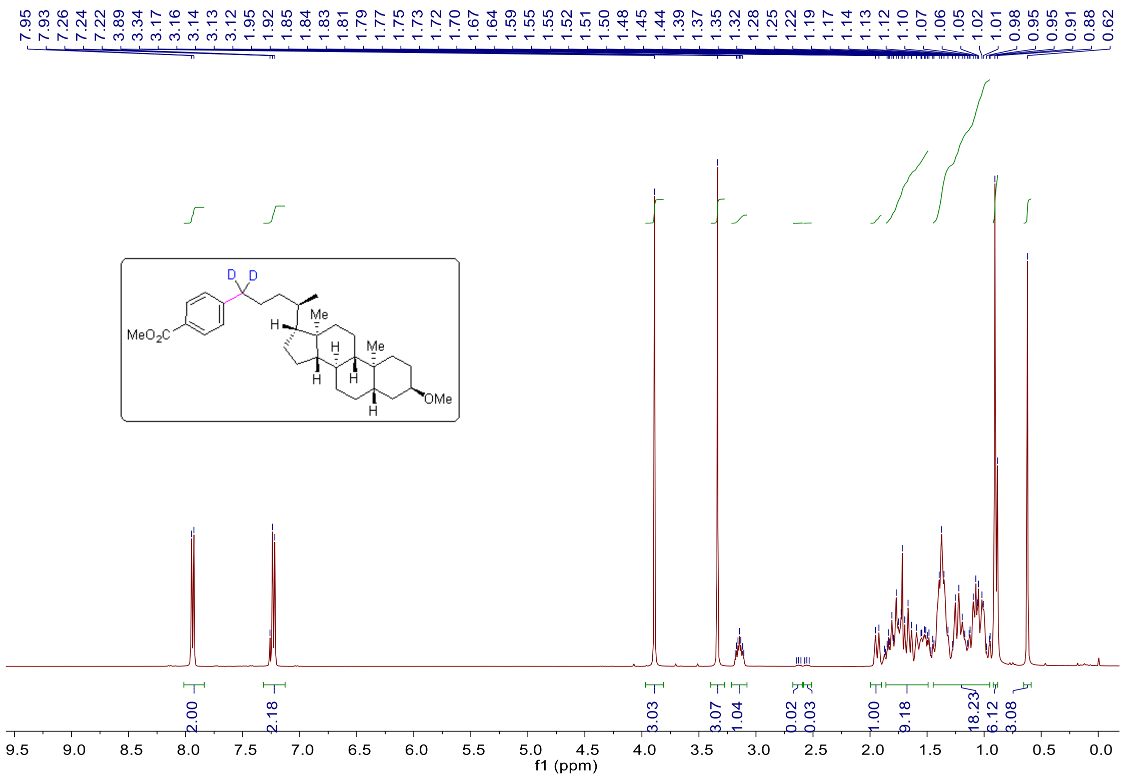

Supplementary Figure 206. ^{1}H NMR (400 MHz, $CDCl_3$) of **5oa**

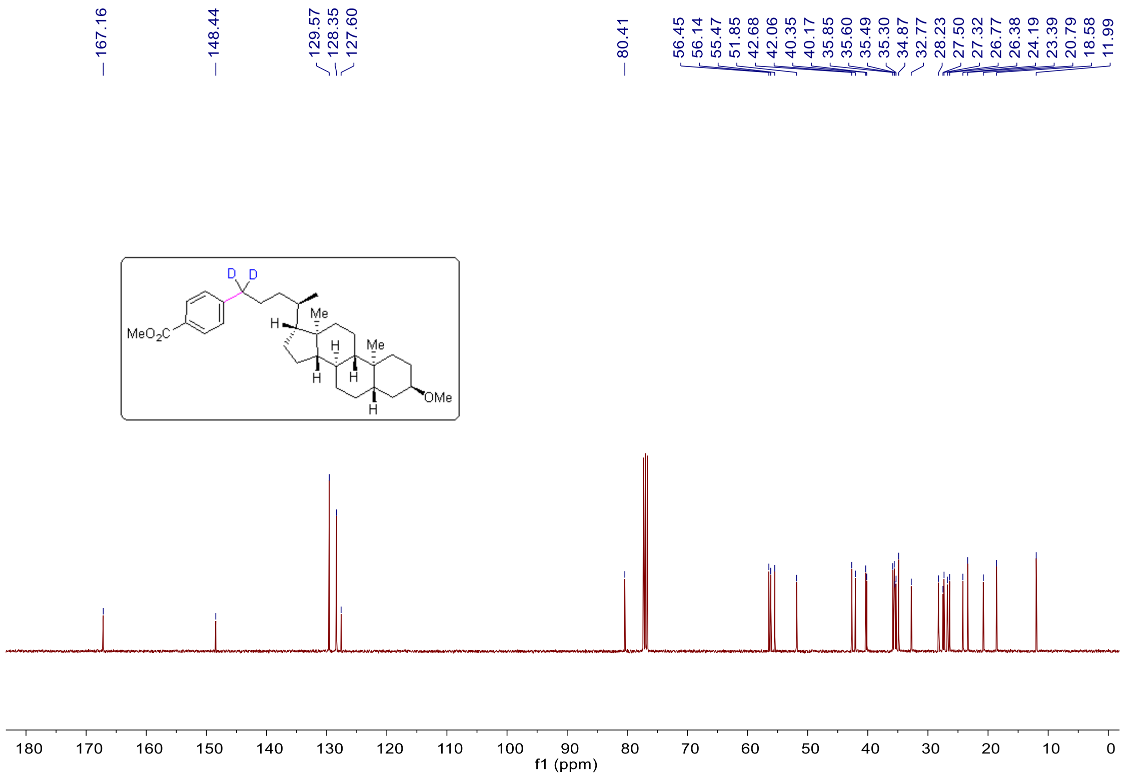

Supplementary Figure 207. ^{13}C NMR (101 MHz, $CDCl_3$) of **5oa**

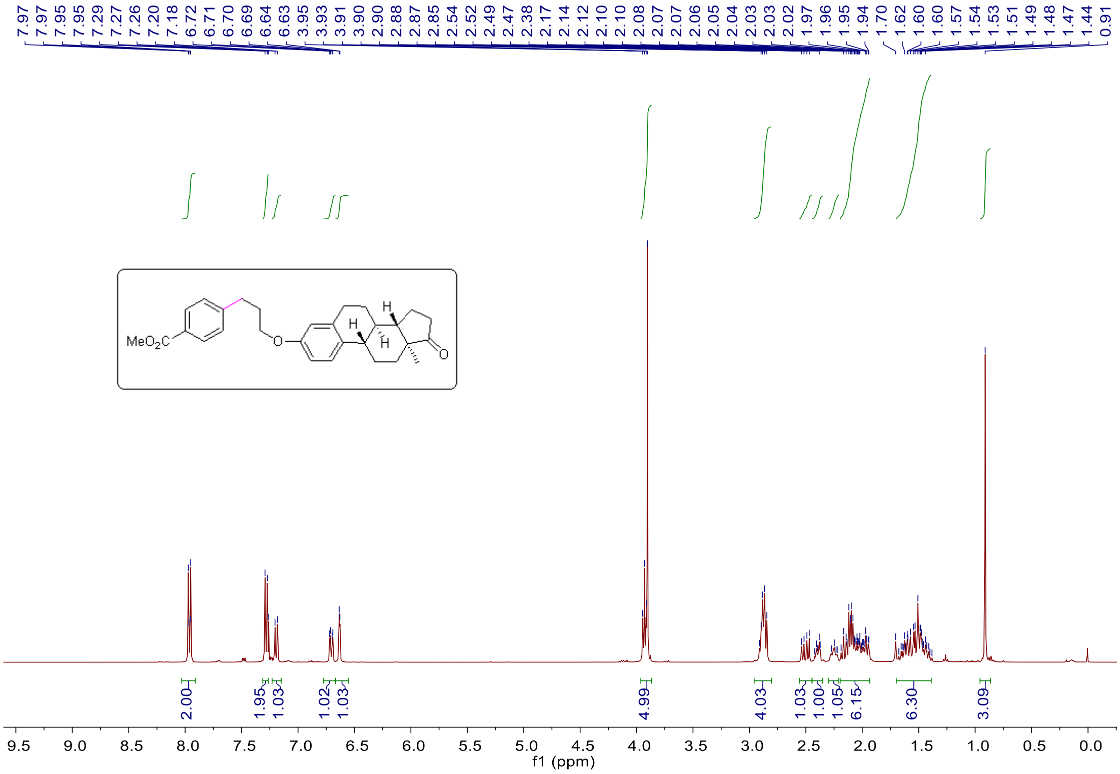

Supplementary Figure 208. ^{1}H NMR (400 MHz, $CDCl_3$) of **5pa'**

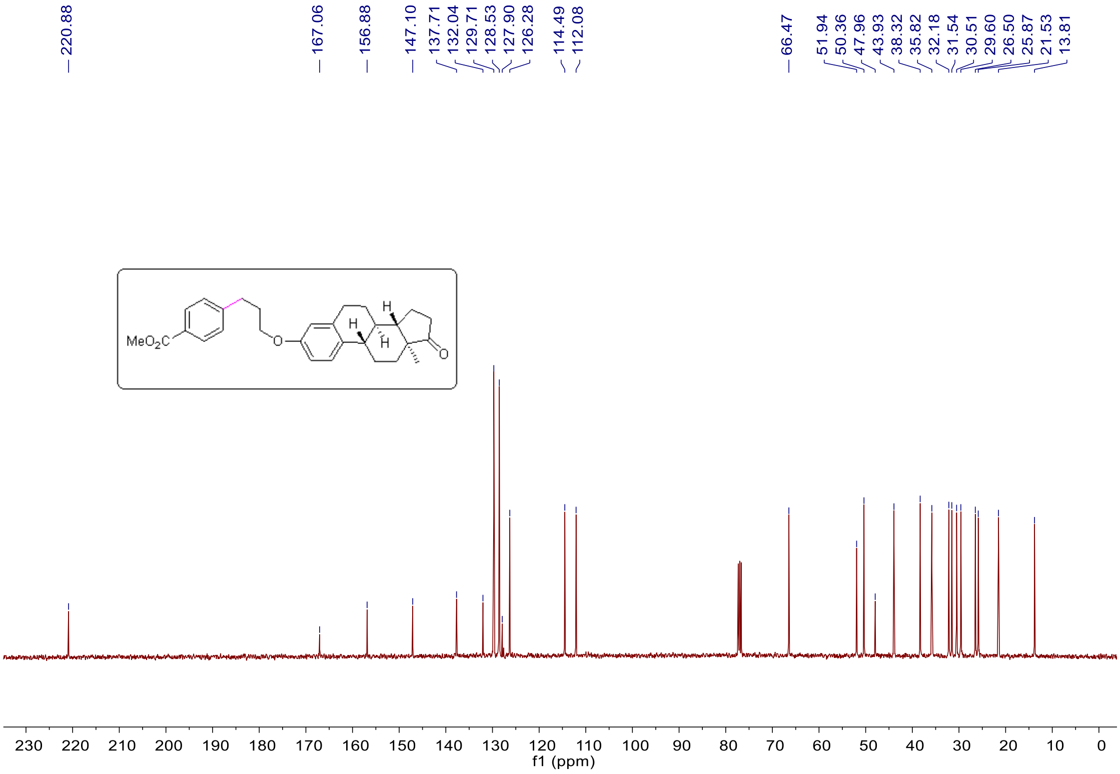

Supplementary Figure 209. ^{13}C NMR (101 MHz, $CDCl_3$) of **5pa'**

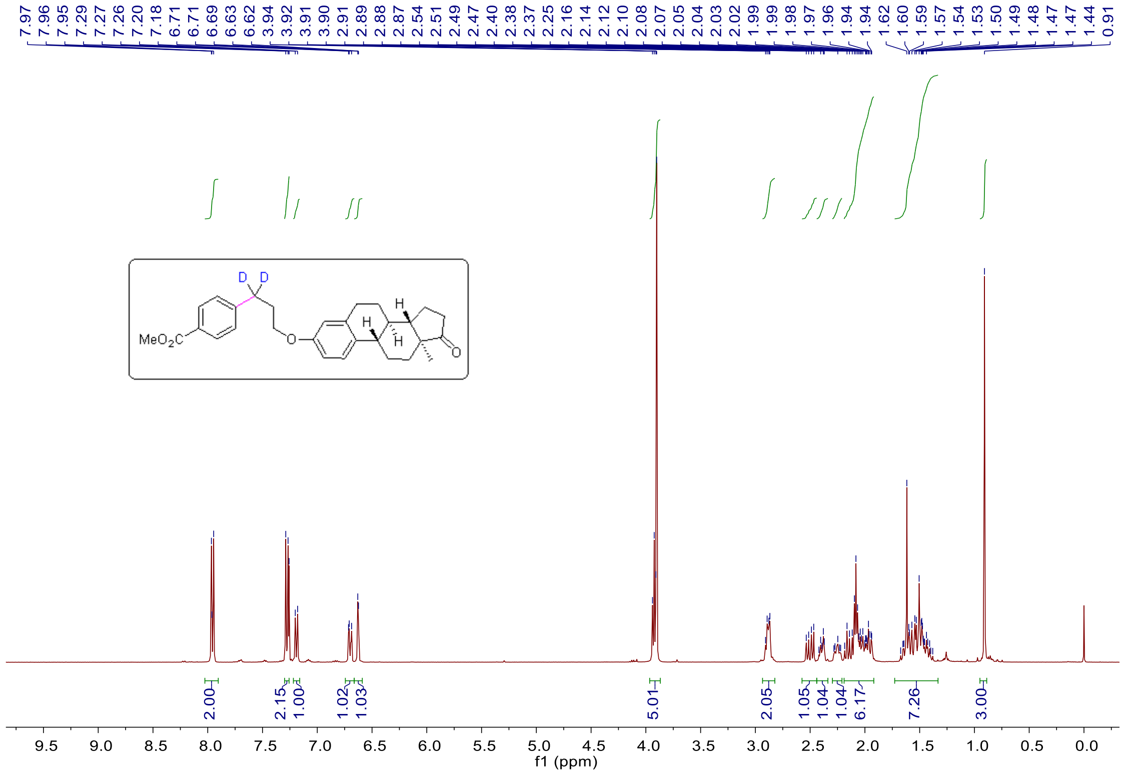

Supplementary Figure 210. ^{1}H NMR (400 MHz, $CDCl_3$) of **5pa**

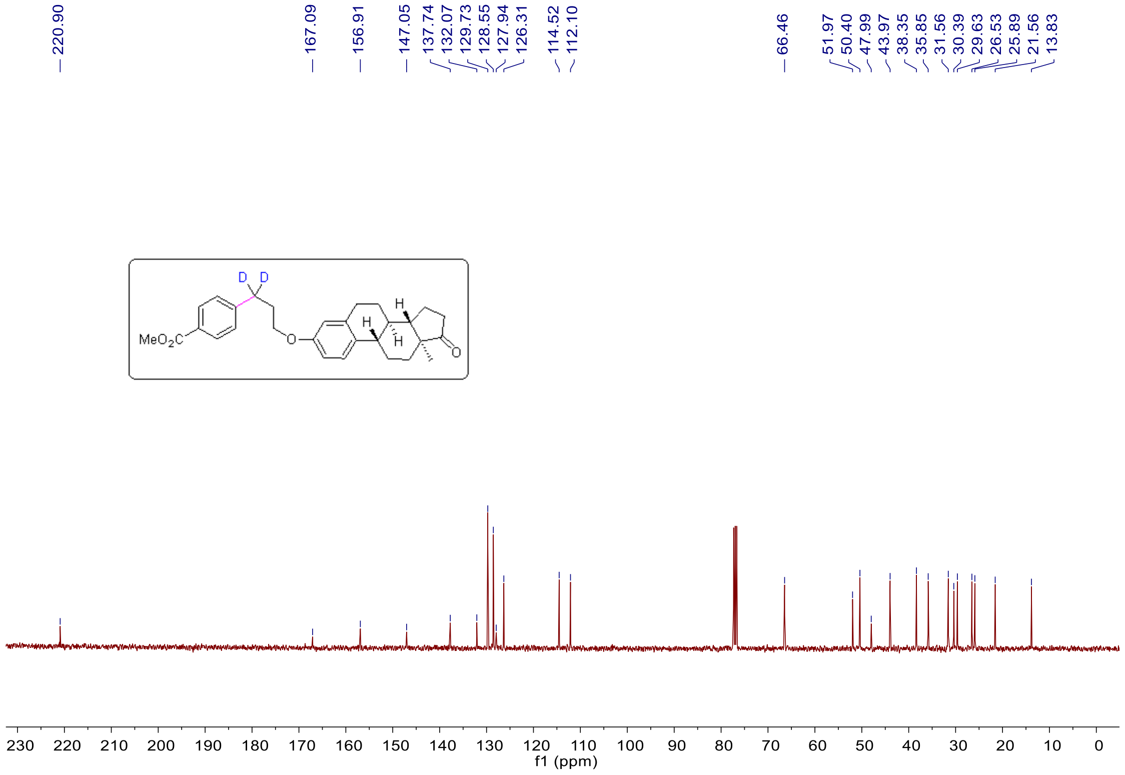

Supplementary Figure 211. ^{13}C NMR (101 MHz, $CDCl_3$) of **5pa**

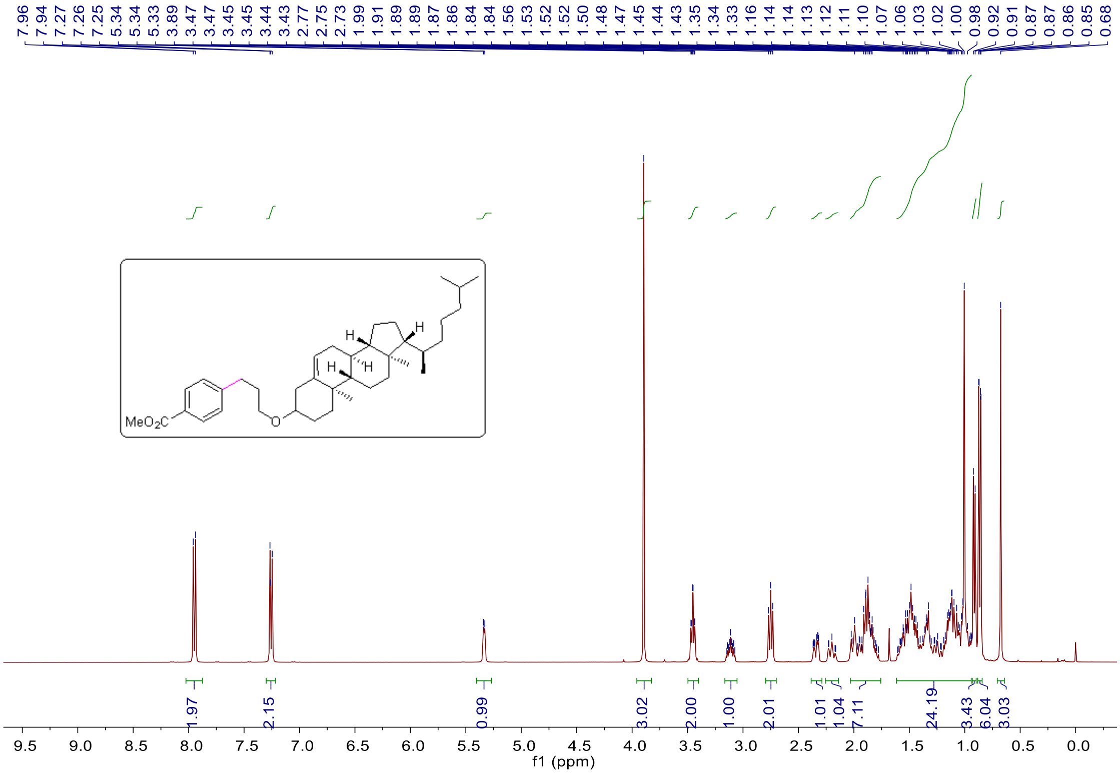

Supplementary Figure 212. ^{1}H NMR (400 MHz, $CDCl_3$) of **5qa'**

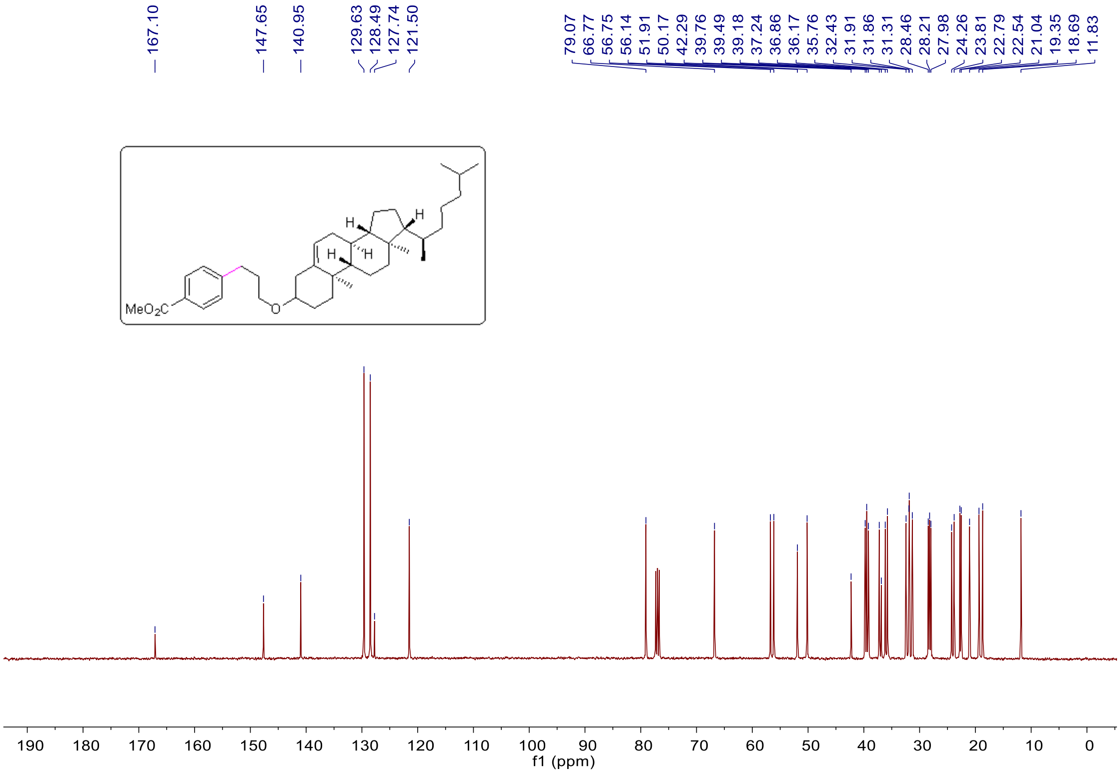

Supplementary Figure 213. ^{13}C NMR (101 MHz, $CDCl_3$) of **5qa'**

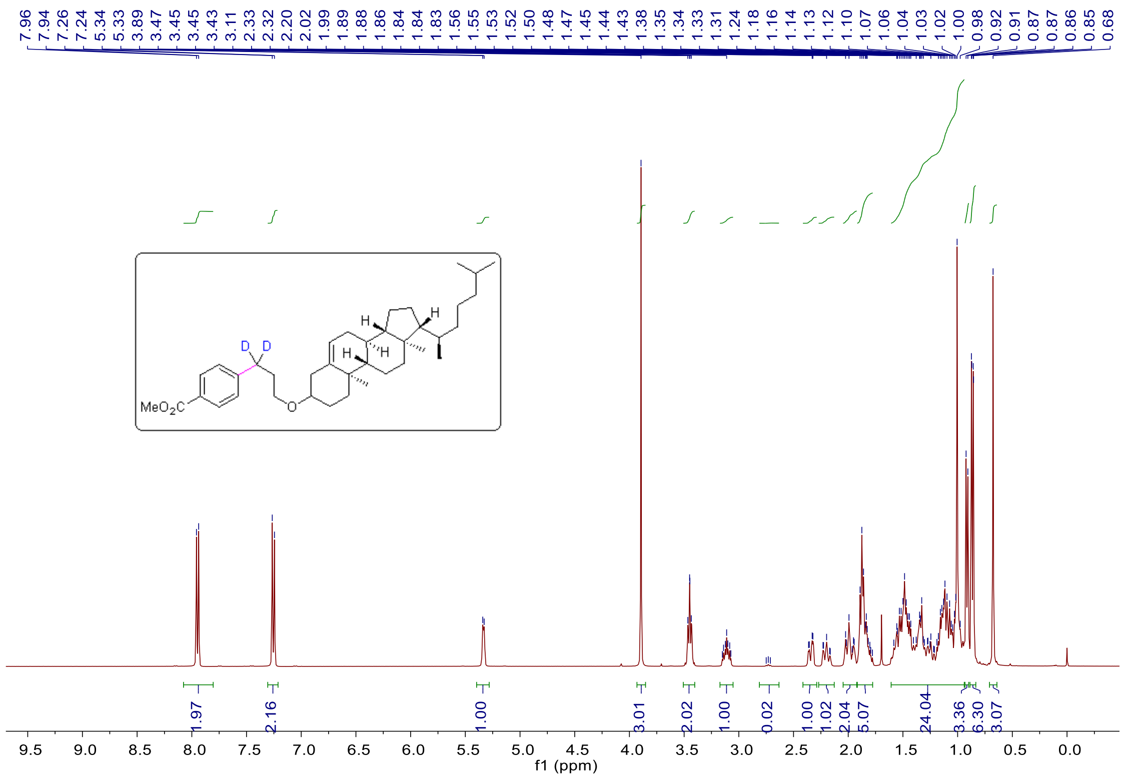

Supplementary Figure 214. ^{1}H NMR (400 MHz, $CDCl_3$) of **5qa**

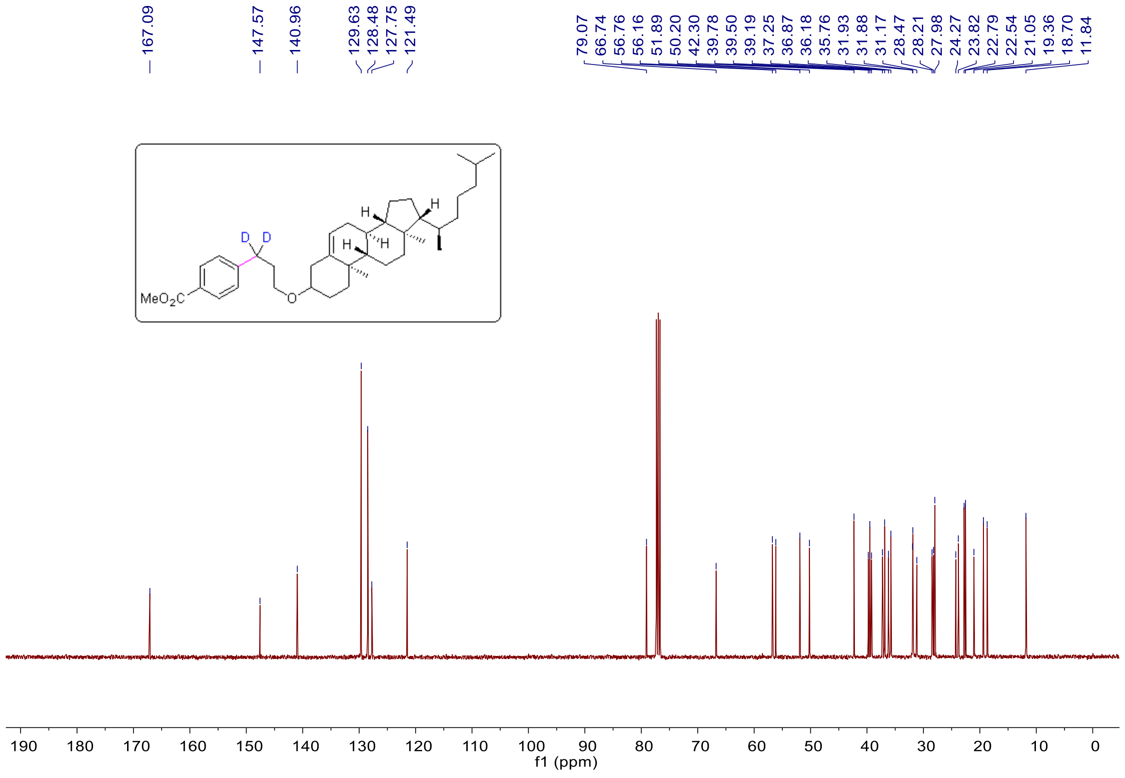

Supplementary Figure 215. ^{13}C NMR (101 MHz, $CDCl_3$) of **5qa**

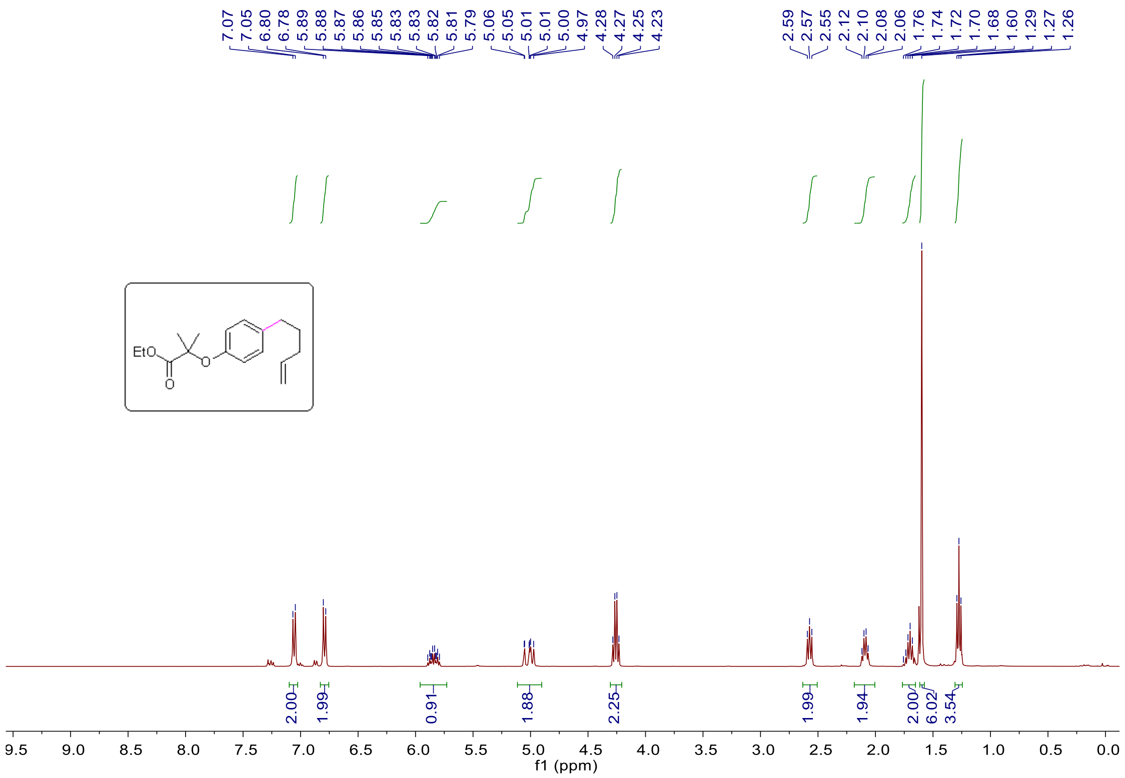

Supplementary Figure 216. ^{1}H NMR (400 MHz, $CDCl_3$) of **5ab'**

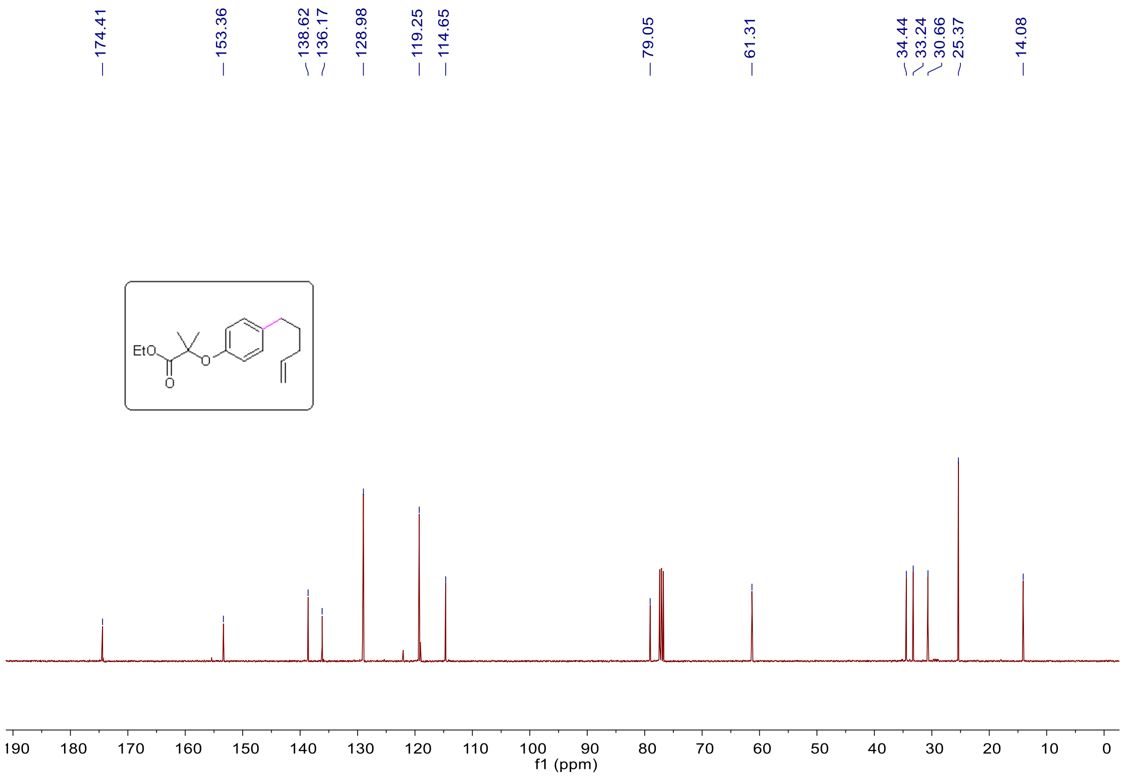

Supplementary Figure 217. ^{13}C NMR (101 MHz, $CDCl_3$) of **5ab'**

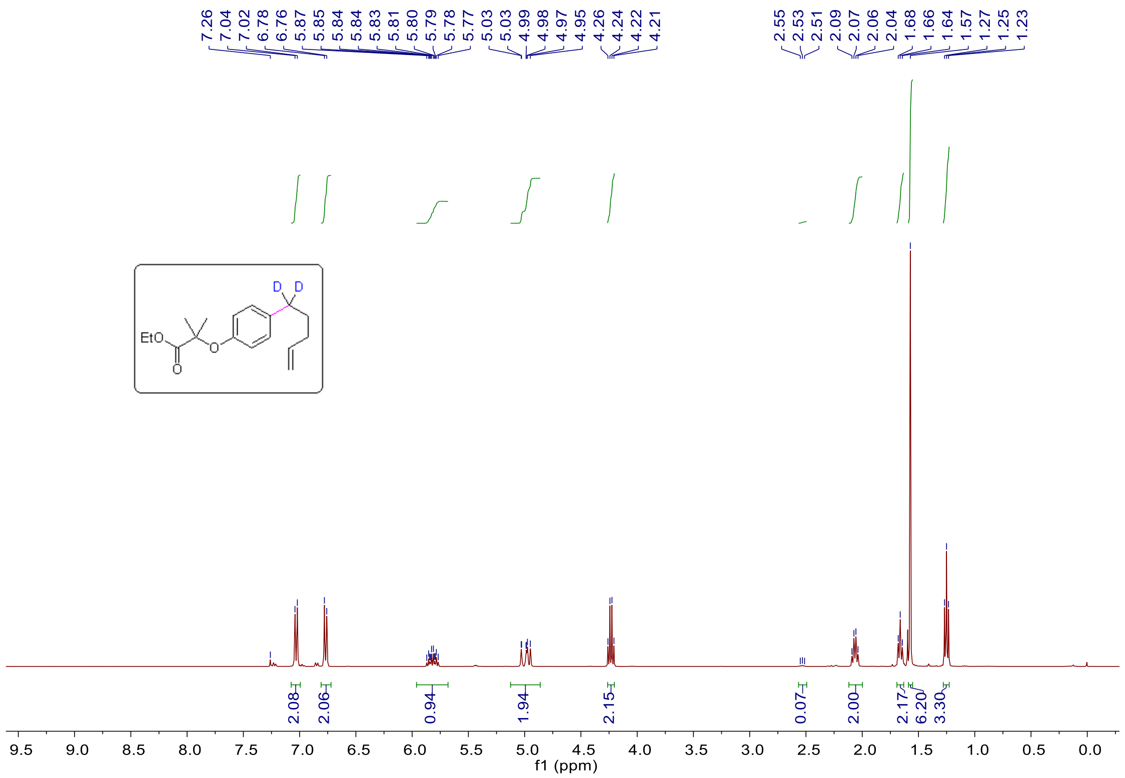

Supplementary Figure 218. ^{1}H NMR (400 MHz, $CDCl_3$) of **5ab**

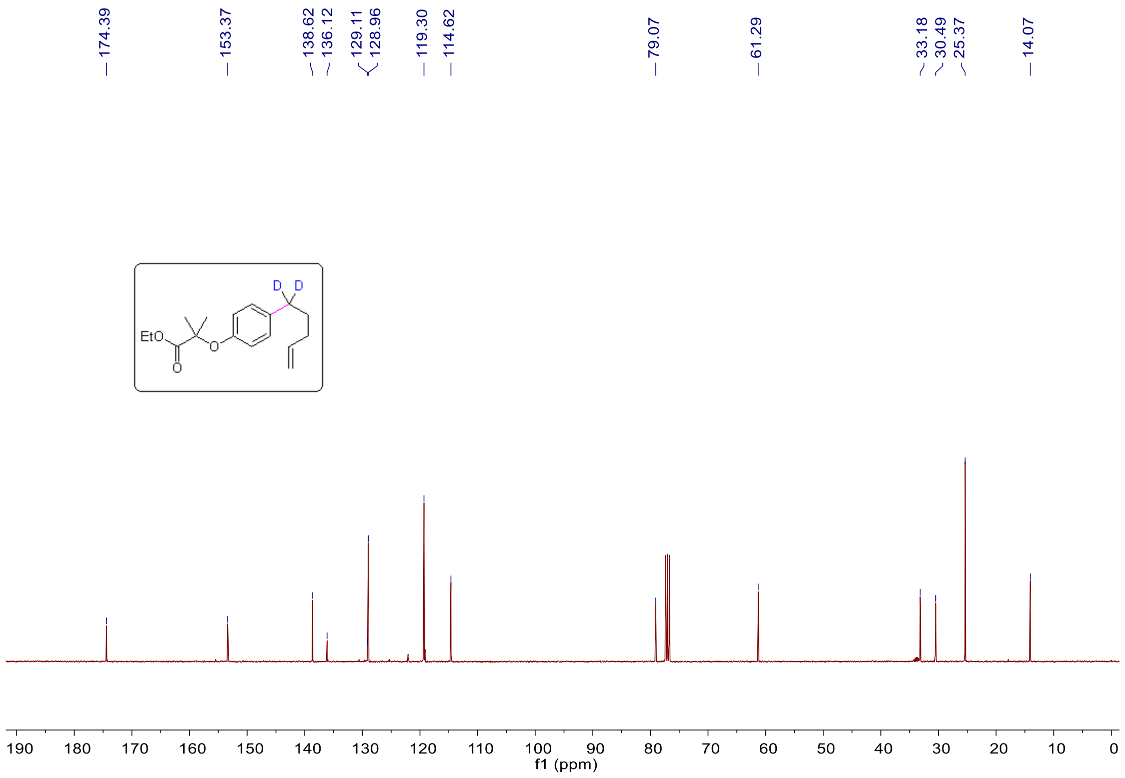

Supplementary Figure 219. ^{13}C NMR (101 MHz, $CDCl_3$) of **5ab**

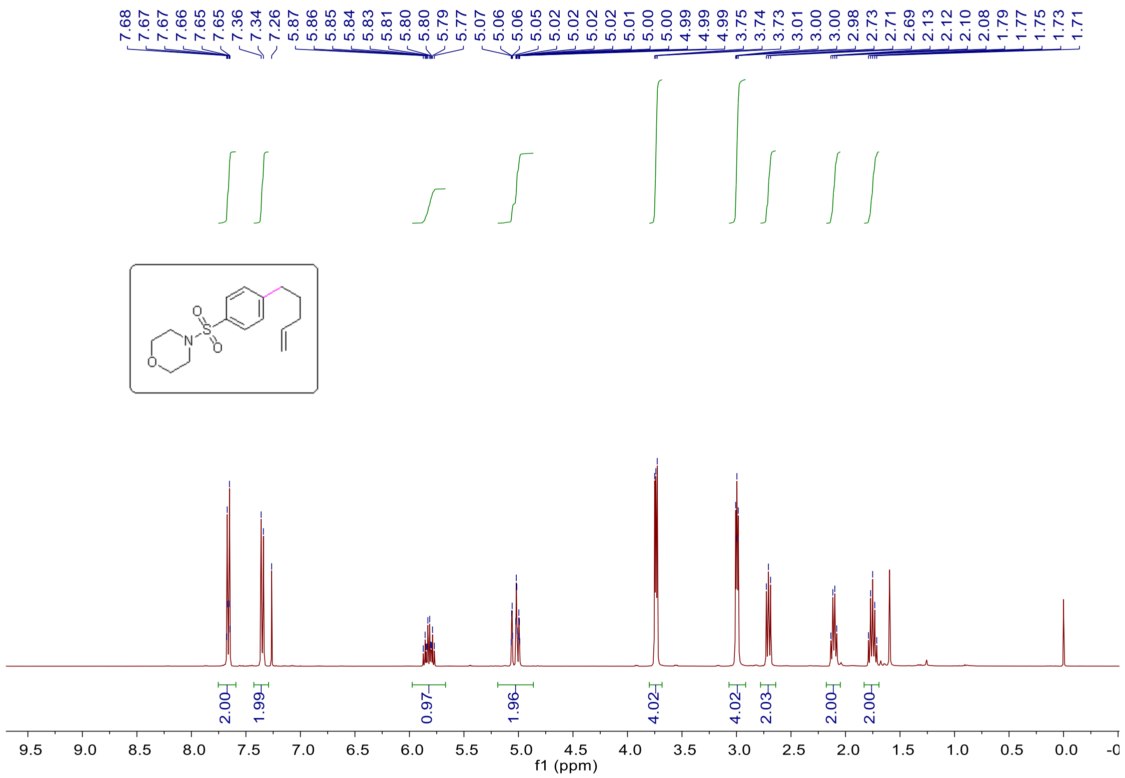

Supplementary Figure 220. ^{1}H NMR (400 MHz, $CDCl_3$) of **5ac'**

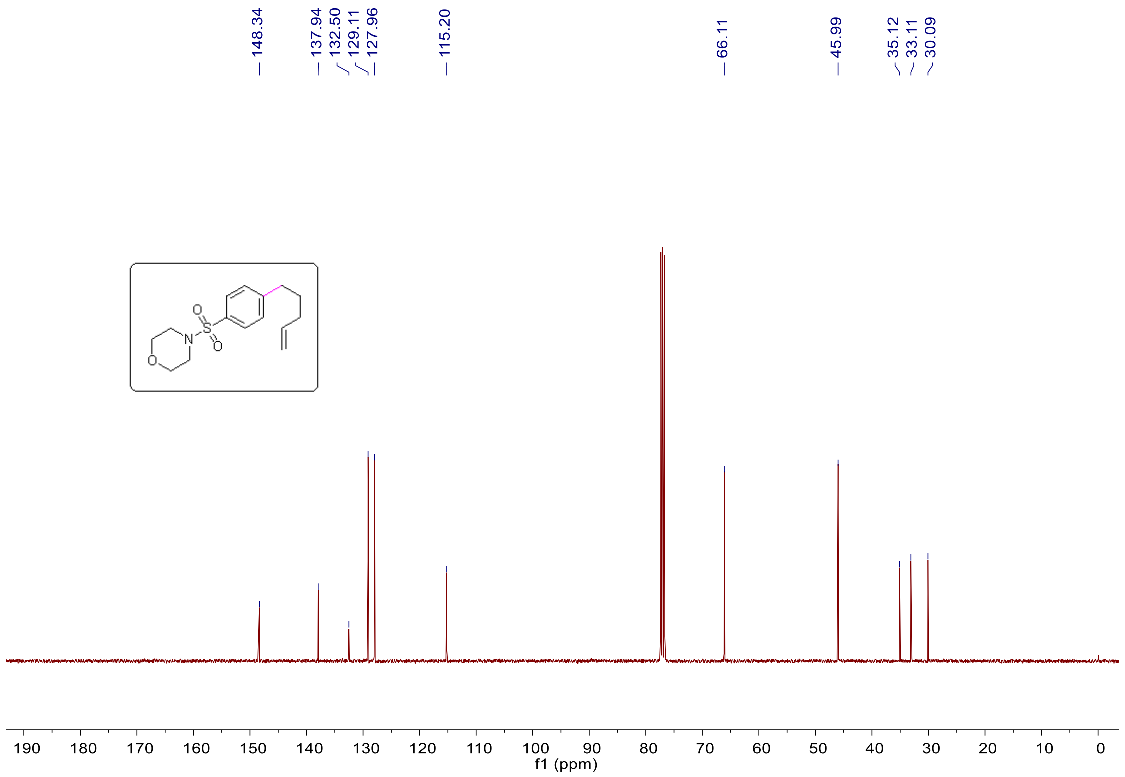

Supplementary Figure 221. ^{13}C NMR (101 MHz, $CDCl_3$) of **5ac'**

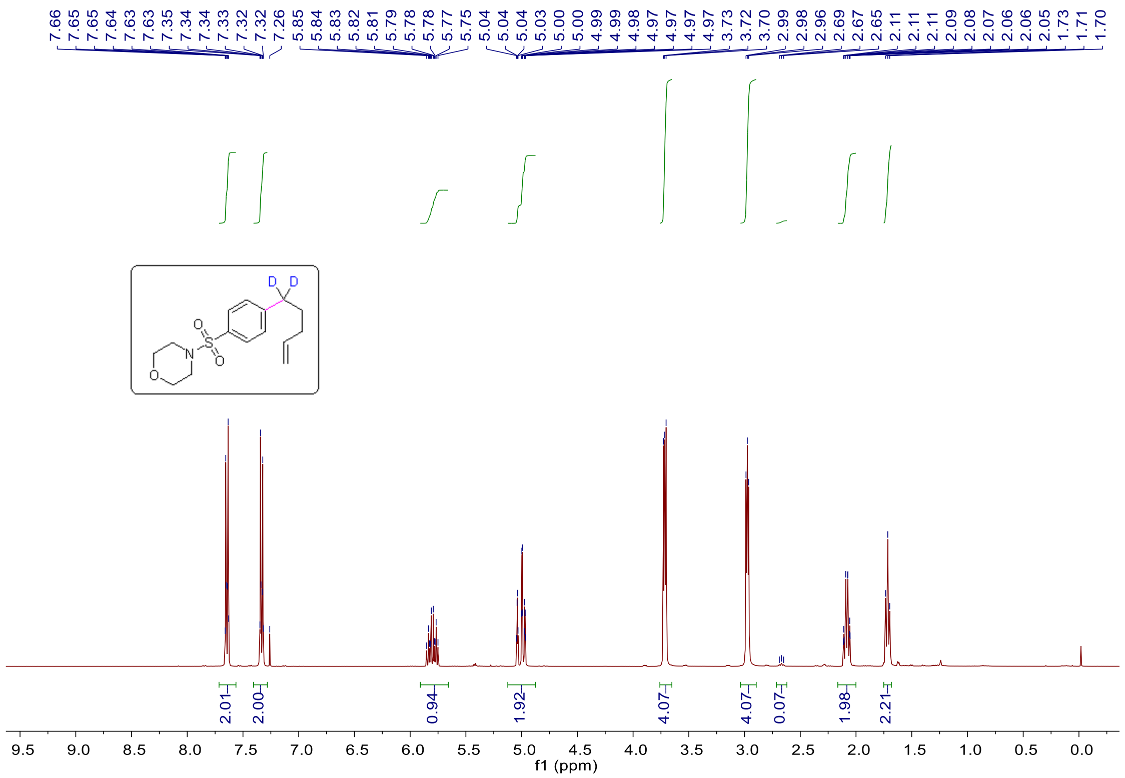

Supplementary Figure 222. ^{1}H NMR (400 MHz, $CDCl_3$) of **5ac**

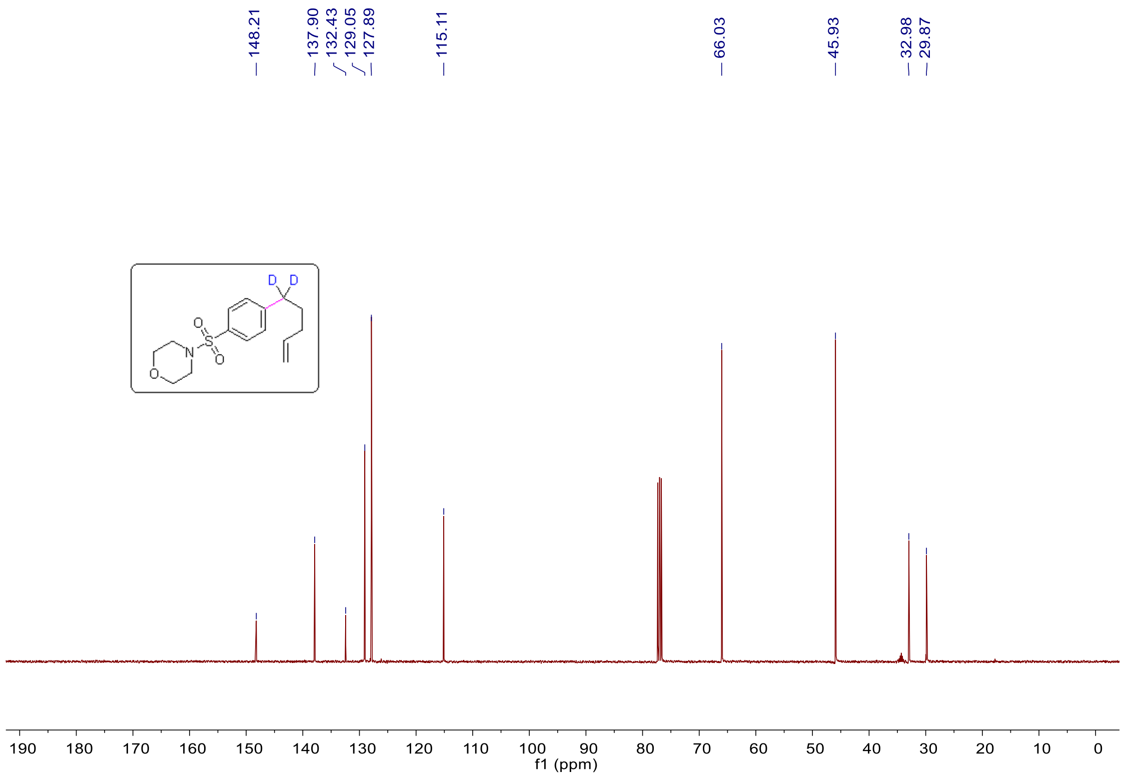

Supplementary Figure 223. ^{13}C NMR (101 MHz, $CDCl_3$) of **5ac**

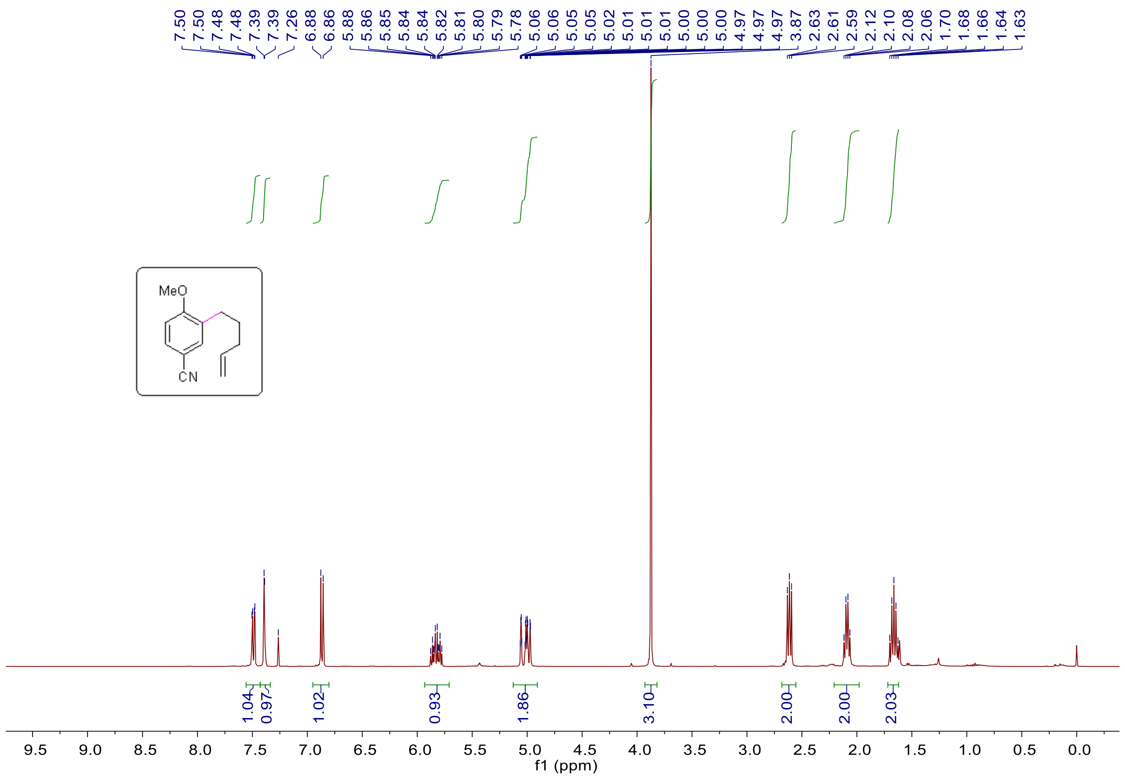

Supplementary Figure 224. ^{1}H NMR (400 MHz, $CDCl_3$) of **5ad'**

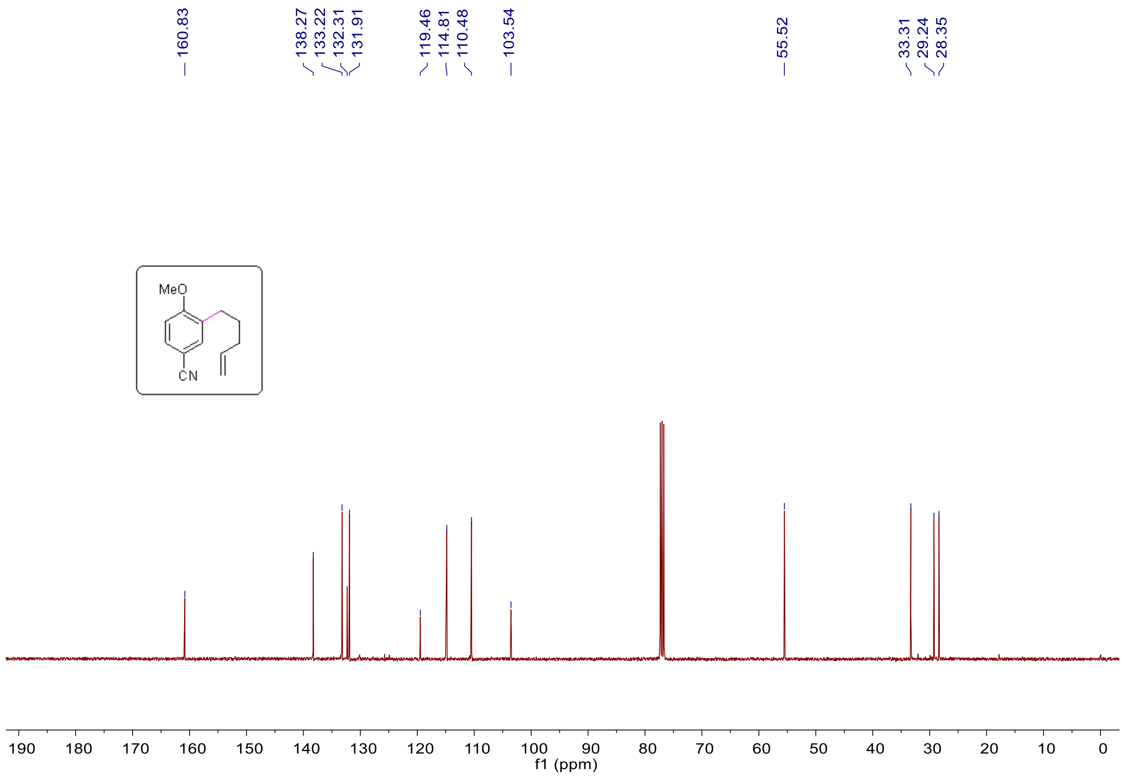

Supplementary Figure 225. ^{13}C NMR (101 MHz, $CDCl_3$) of **5ad'**

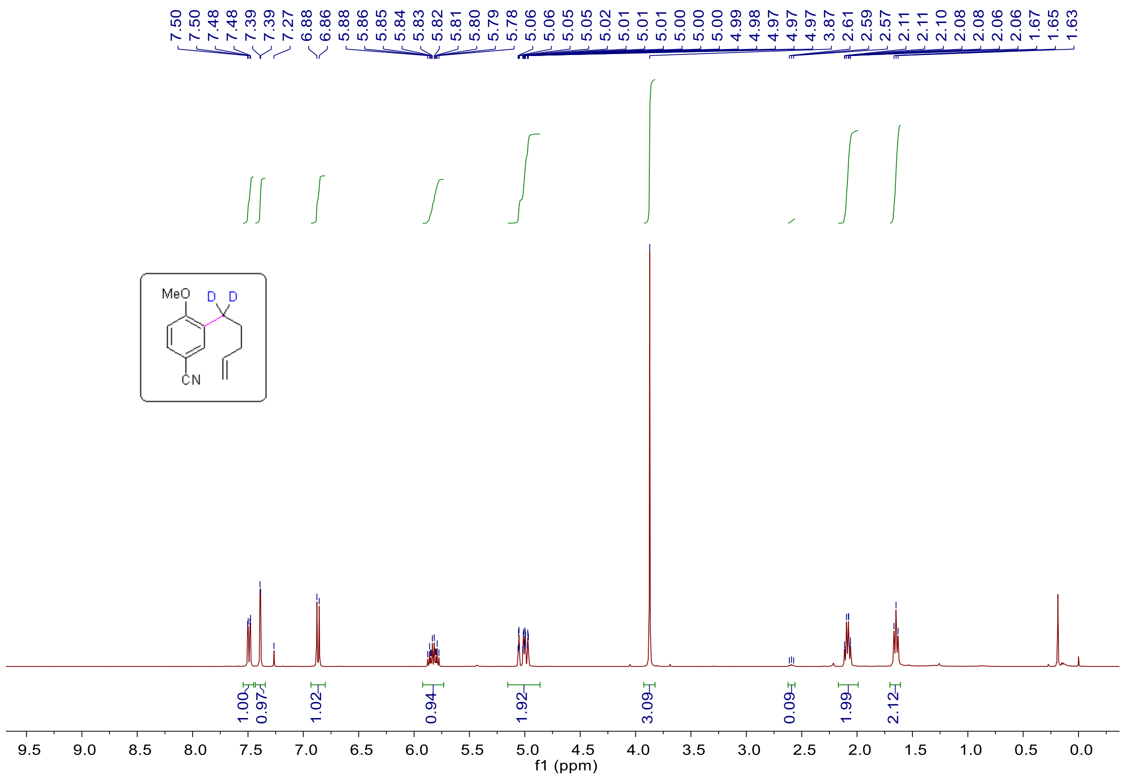

Supplementary Figure 226. ^{1}H NMR (400 MHz, $CDCl_3$) of **5ad**

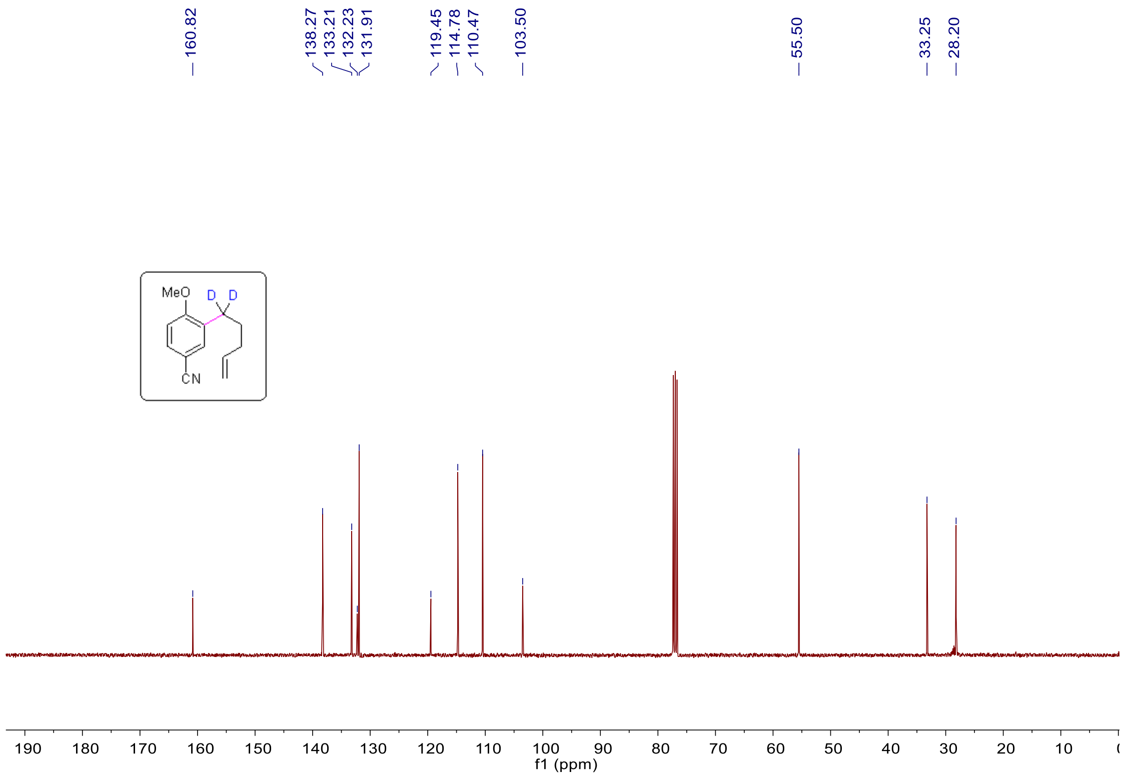

Supplementary Figure 227. ^{13}C NMR (101 MHz, $CDCl_3$) of **5ad**

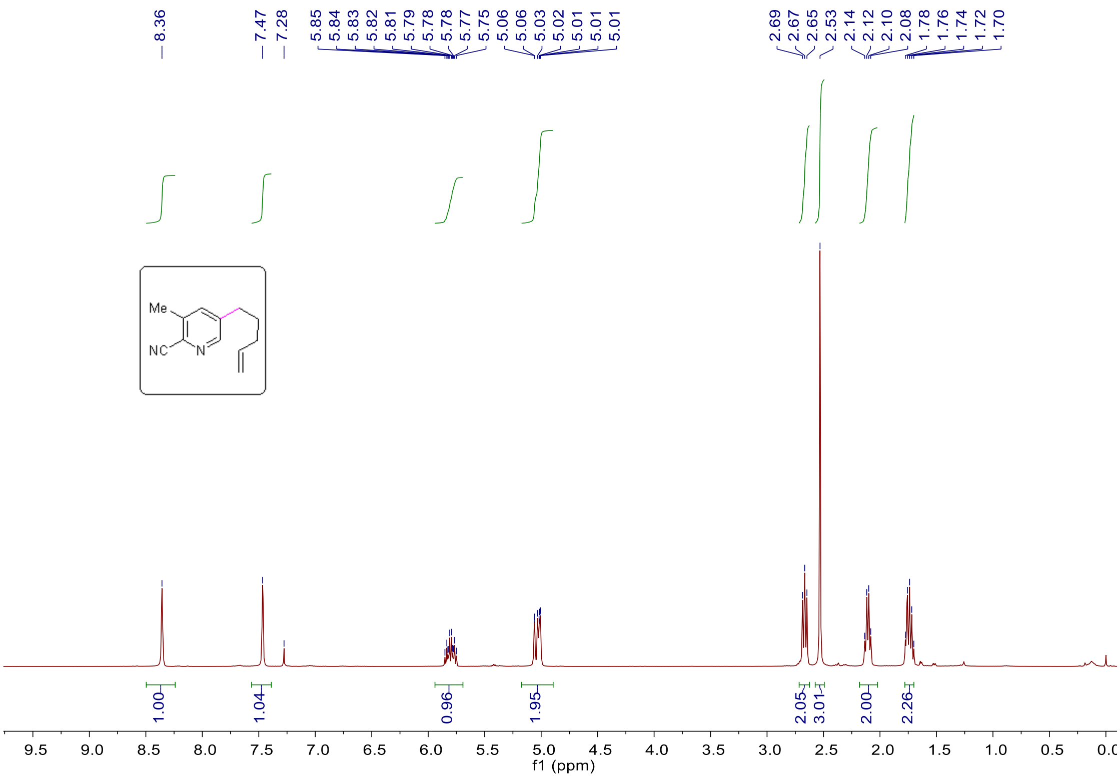

Supplementary Figure 228. ^{1}H NMR (400 MHz, $CDCl_3$) of **5ae'**

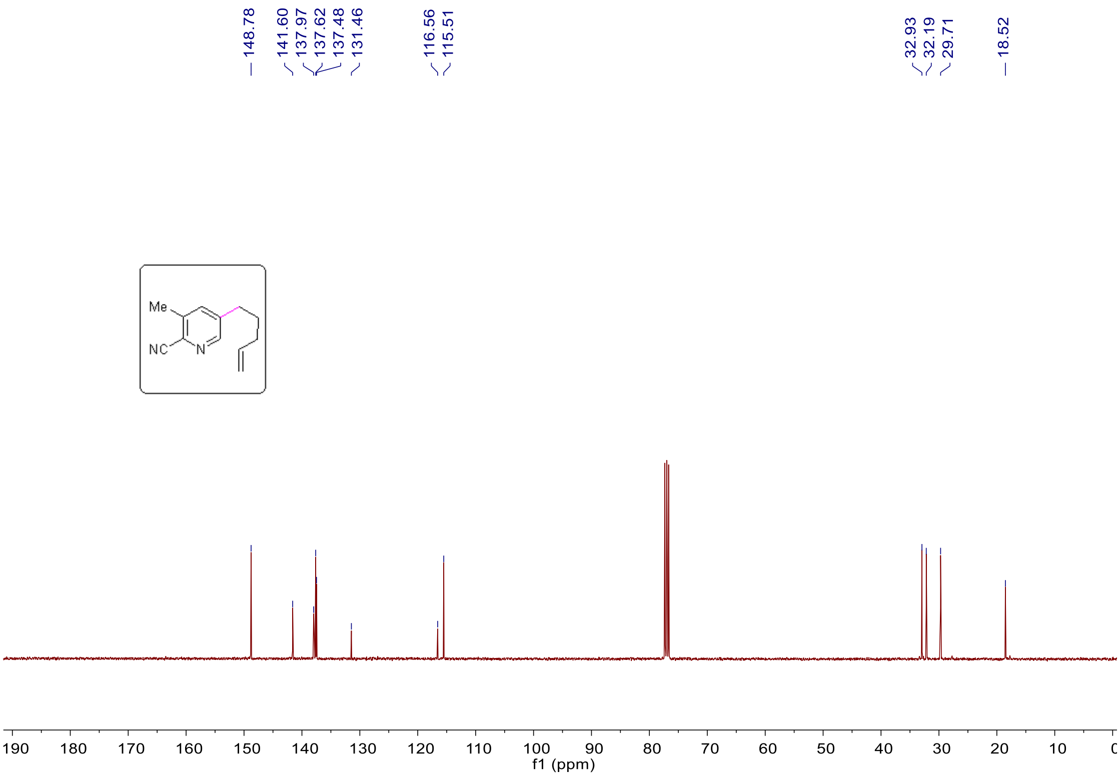

Supplementary Figure 229. ^{13}C NMR (101 MHz, $CDCl_3$) of **5ae'**

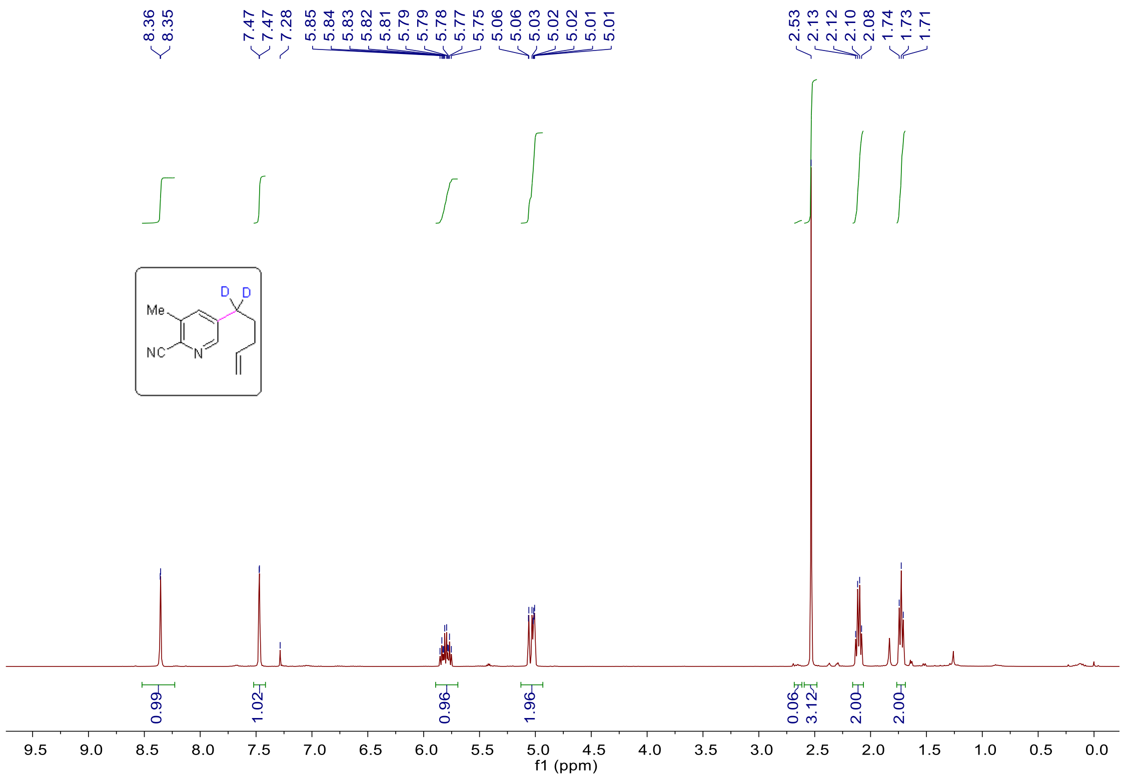

Supplementary Figure 230. ^{1}H NMR (400 MHz, $CDCl_3$) of **5ae**

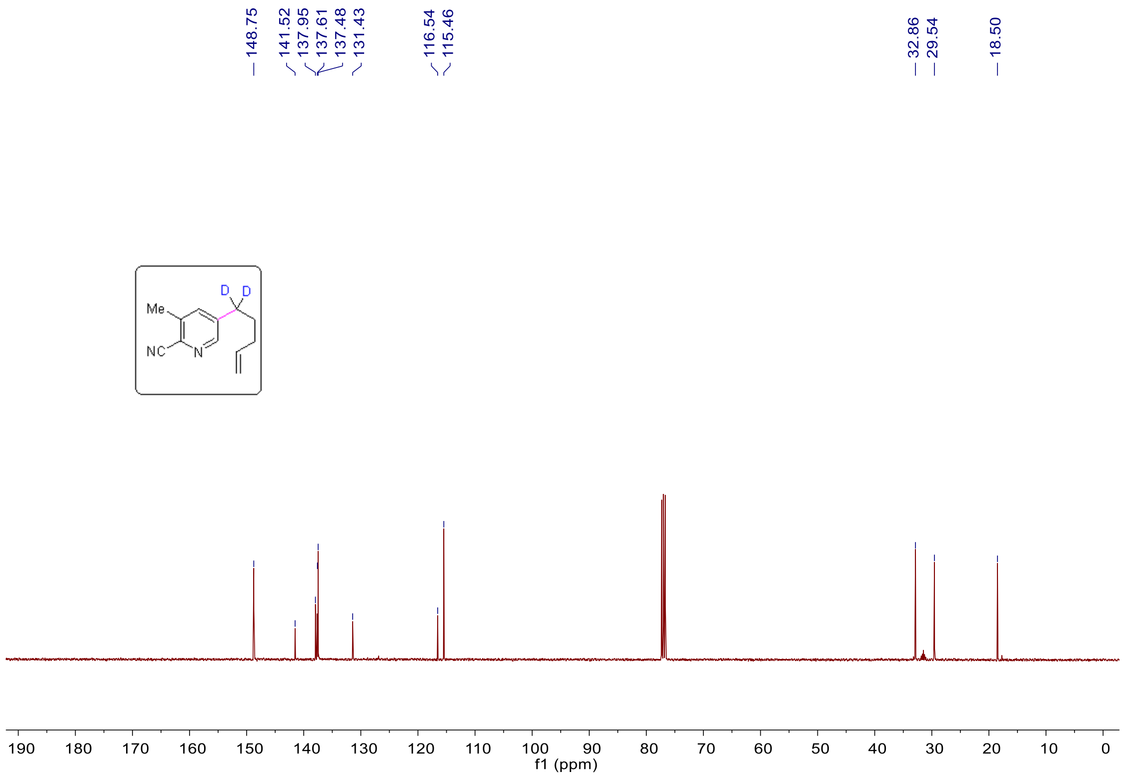

Supplementary Figure 231. ^{13}C NMR (101 MHz, $CDCl_3$) of **5ae**

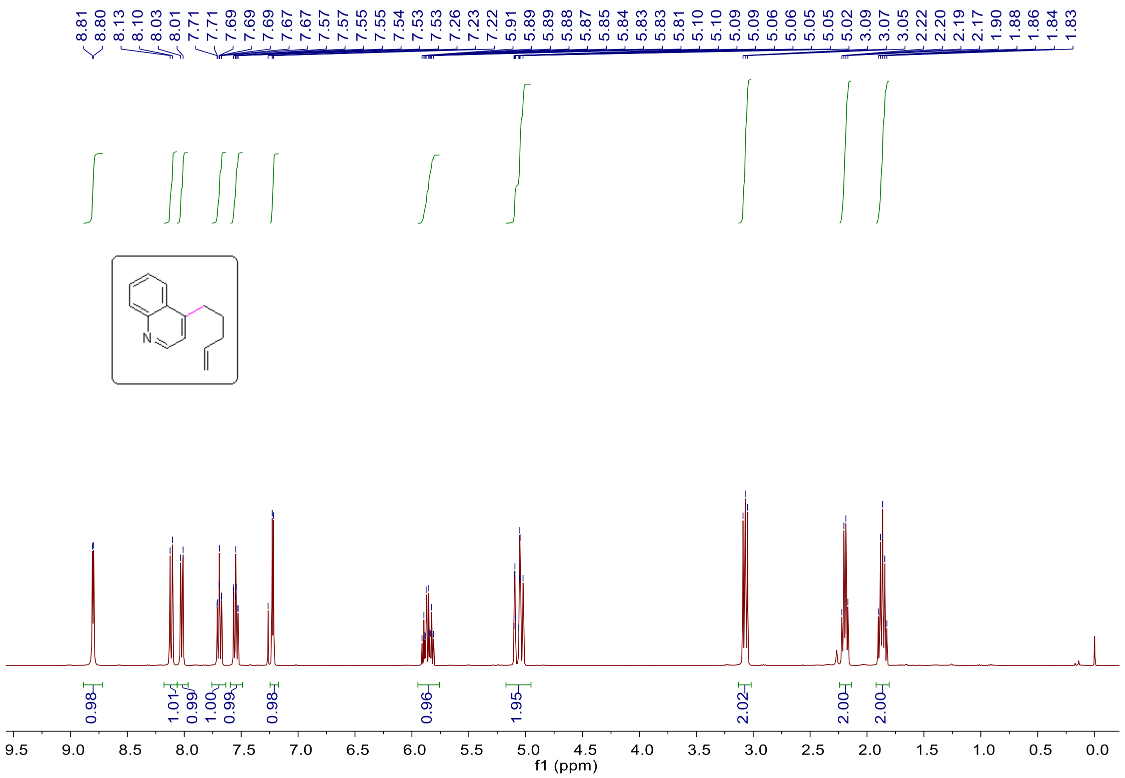

Supplementary Figure 232. ^{1}H NMR (400 MHz, $CDCl_3$) of **5af'**

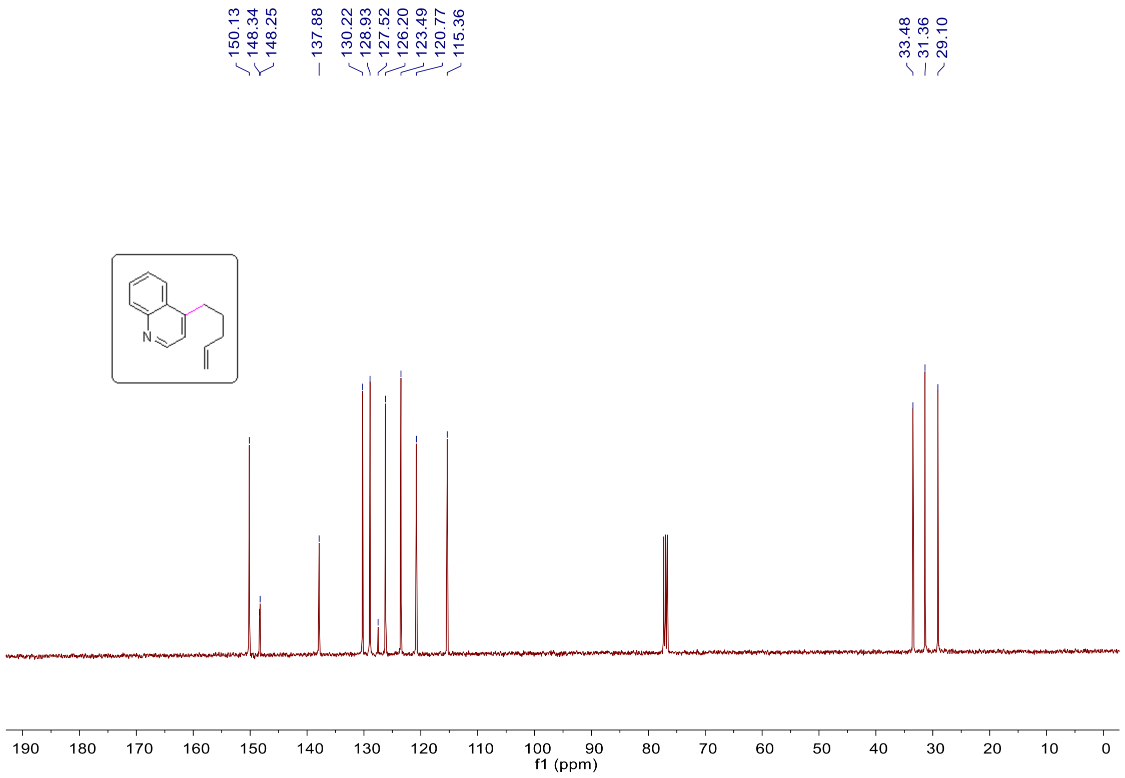

Supplementary Figure 233. ^{13}C NMR (101 MHz, $CDCl_3$) of **5af'**

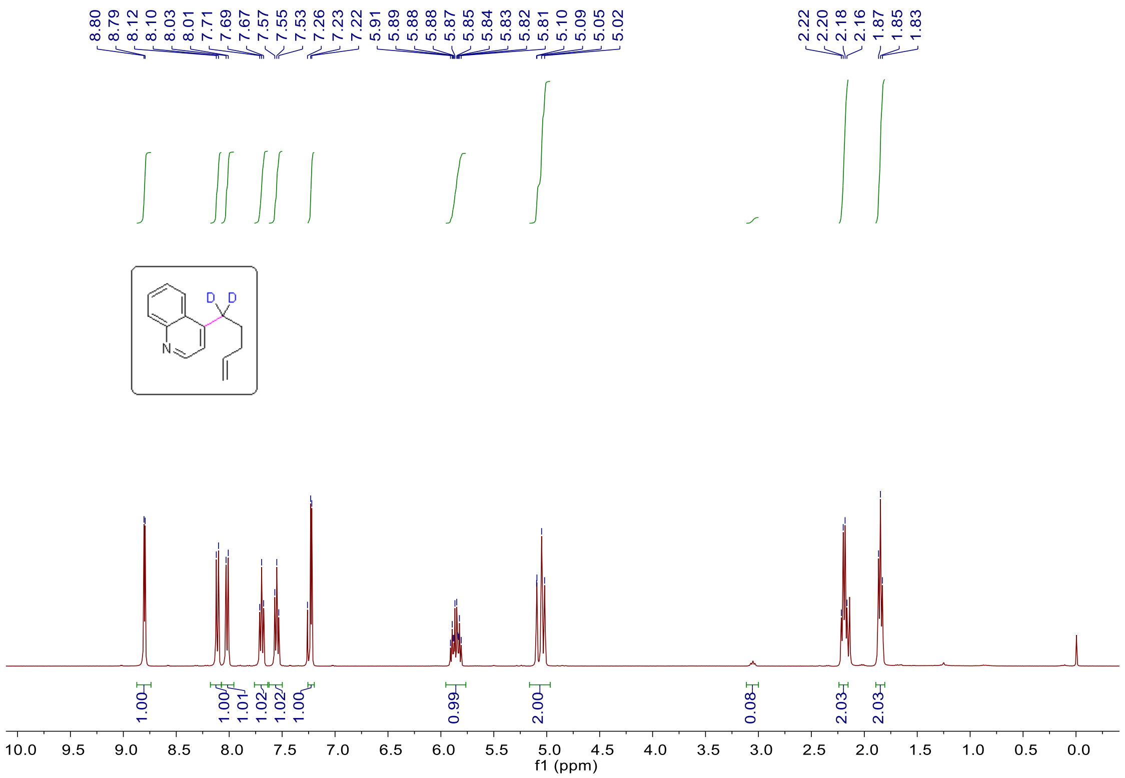

Supplementary Figure 234. ^{1}H NMR (400 MHz, $CDCl_3$) of **5af**

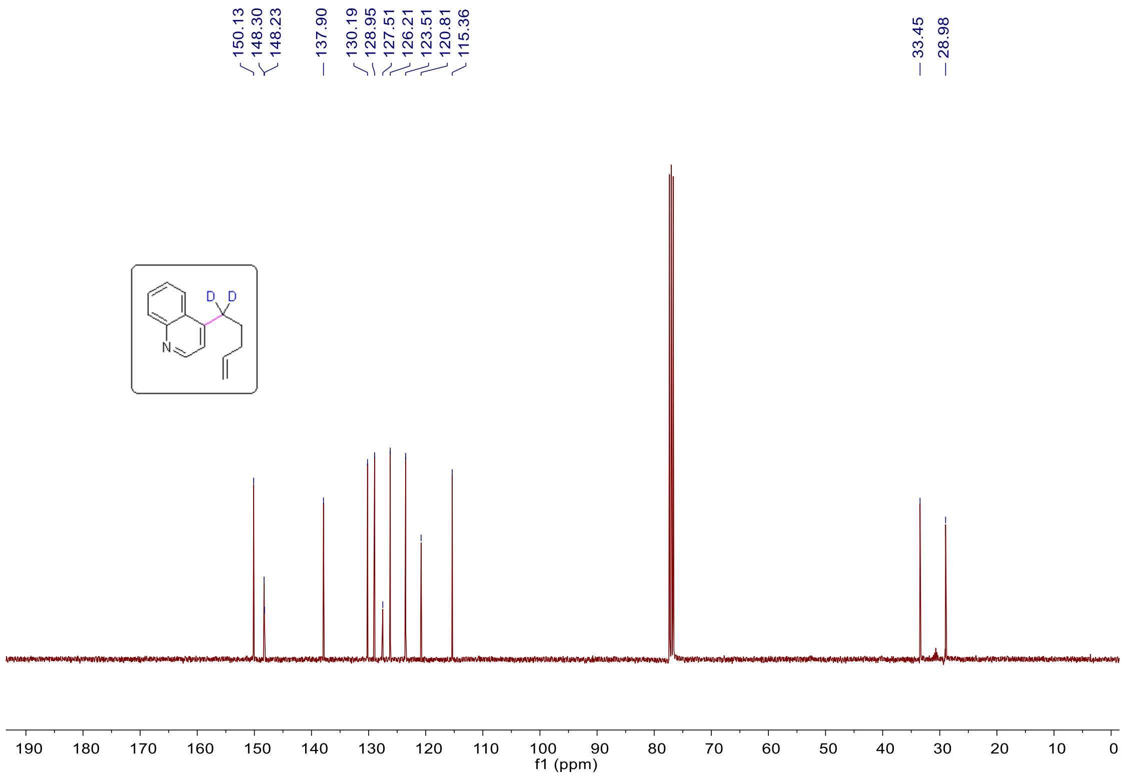

Supplementary Figure 235. ^{13}C NMR (101 MHz, $CDCl_3$) of **5af**

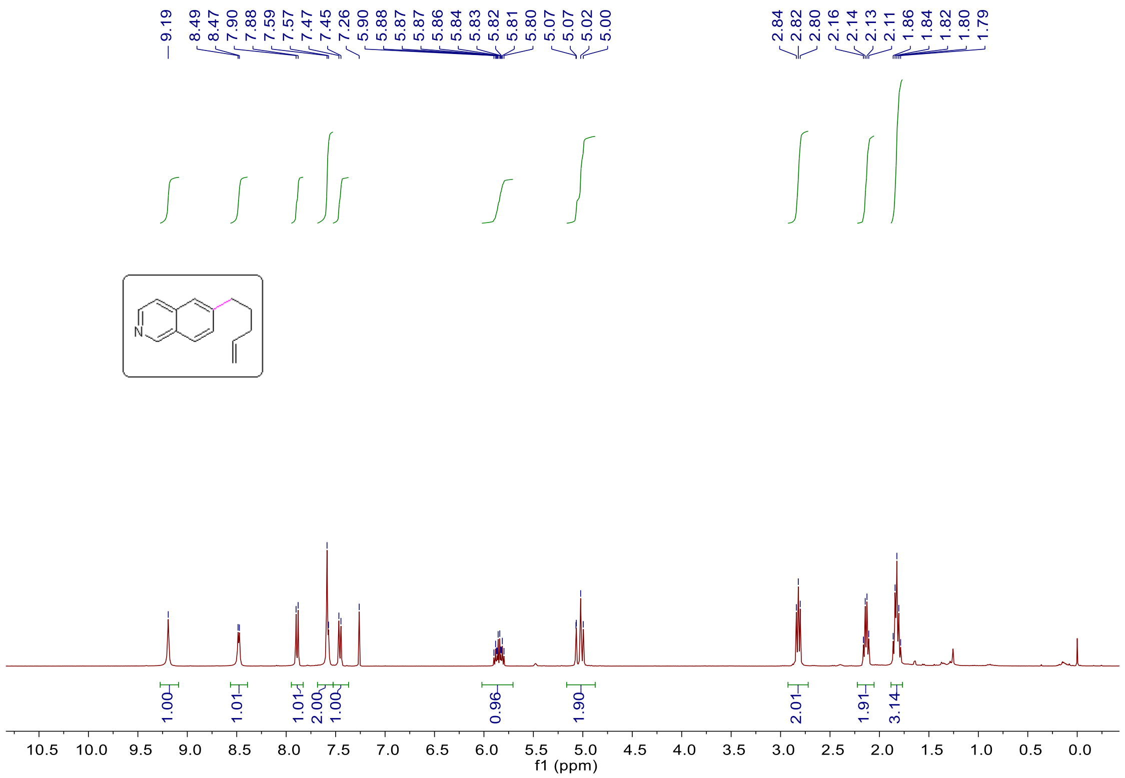

Supplementary Figure 236. ^{1}H NMR (400 MHz, $CDCl_3$) of **5ag'**

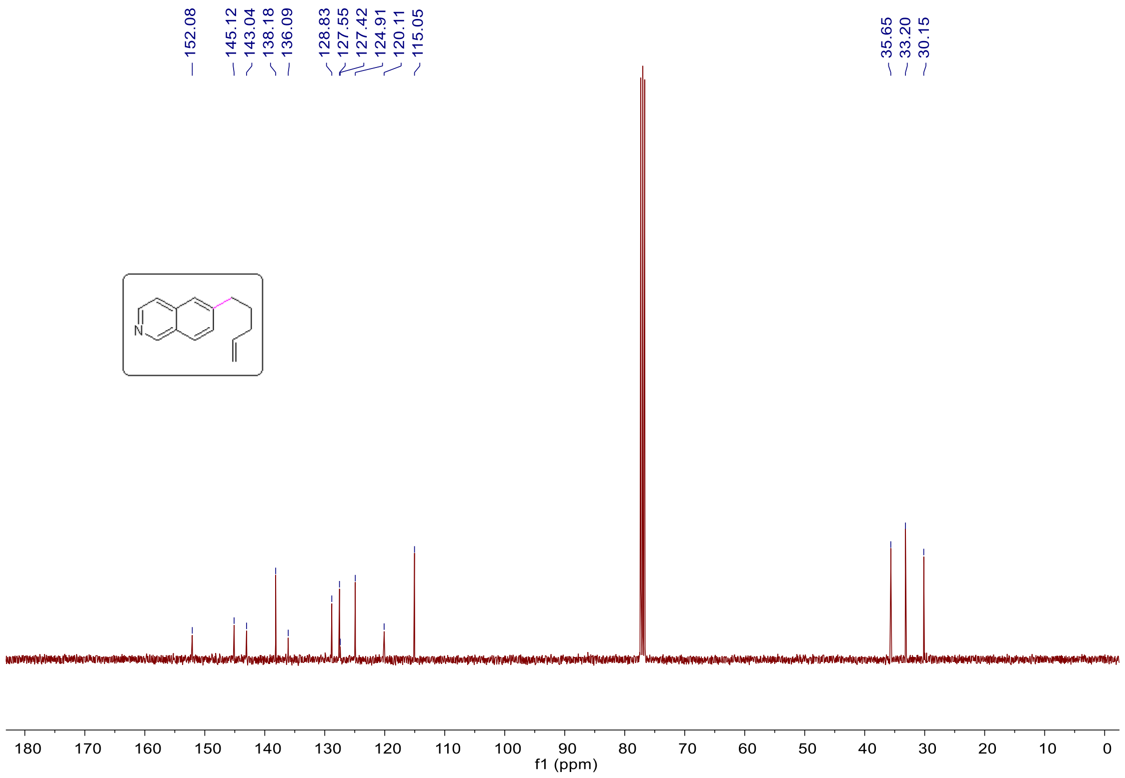

Supplementary Figure 237. ^{13}C NMR (101 MHz, $CDCl_3$) of **5ag'**

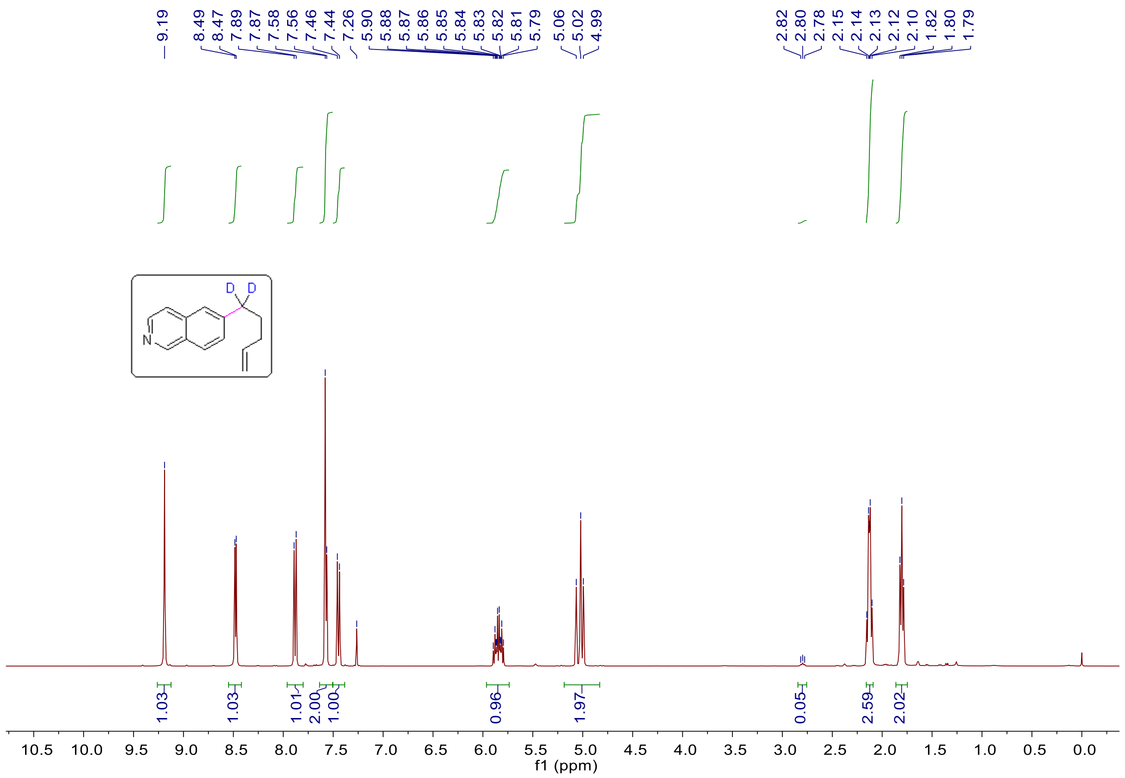

Supplementary Figure 238. ^{1}H NMR (400 MHz, $CDCl_3$) of **5ag**

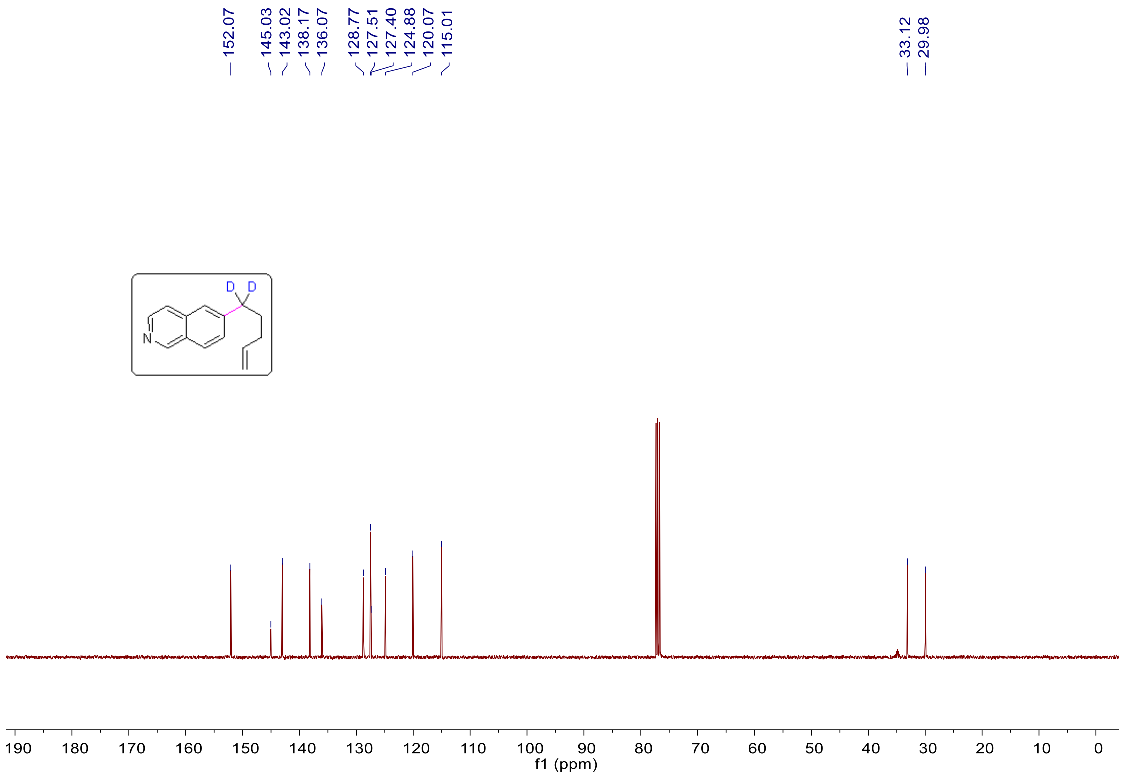

Supplementary Figure 239. ^{13}C NMR (101 MHz, $CDCl_3$) of **5ag**

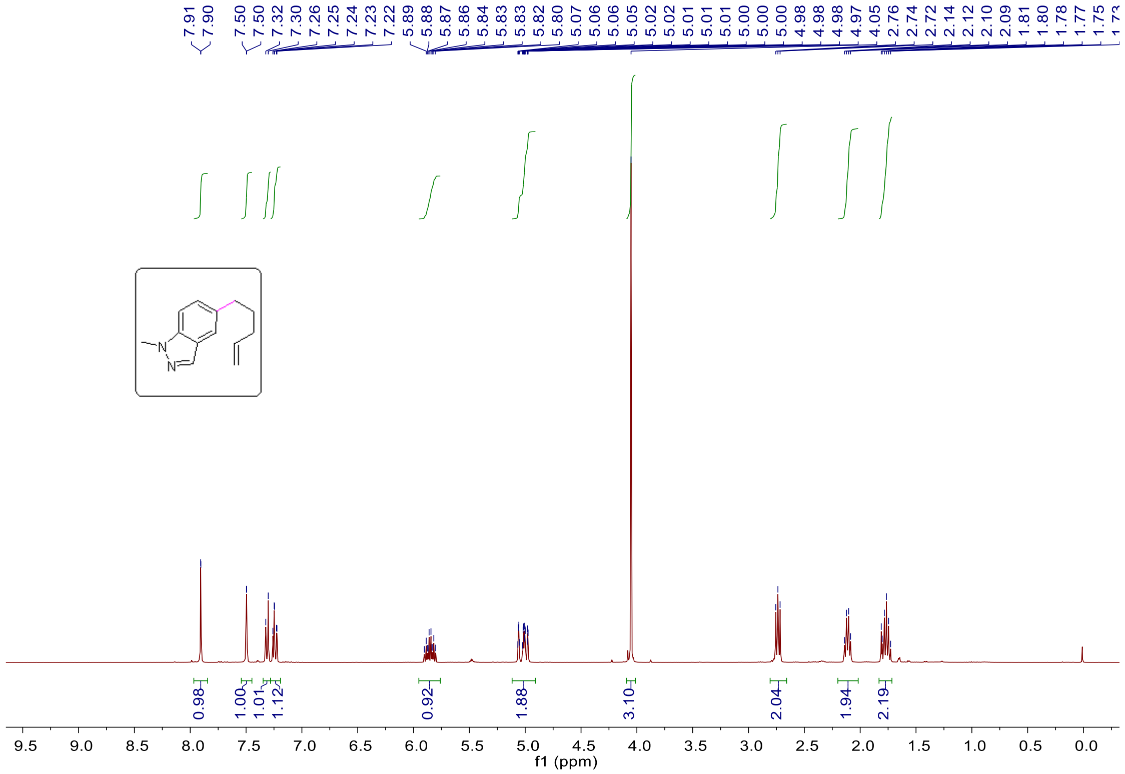

Supplementary Figure 240. ^{1}H NMR (400 MHz, $CDCl_3$) of **5ah'**

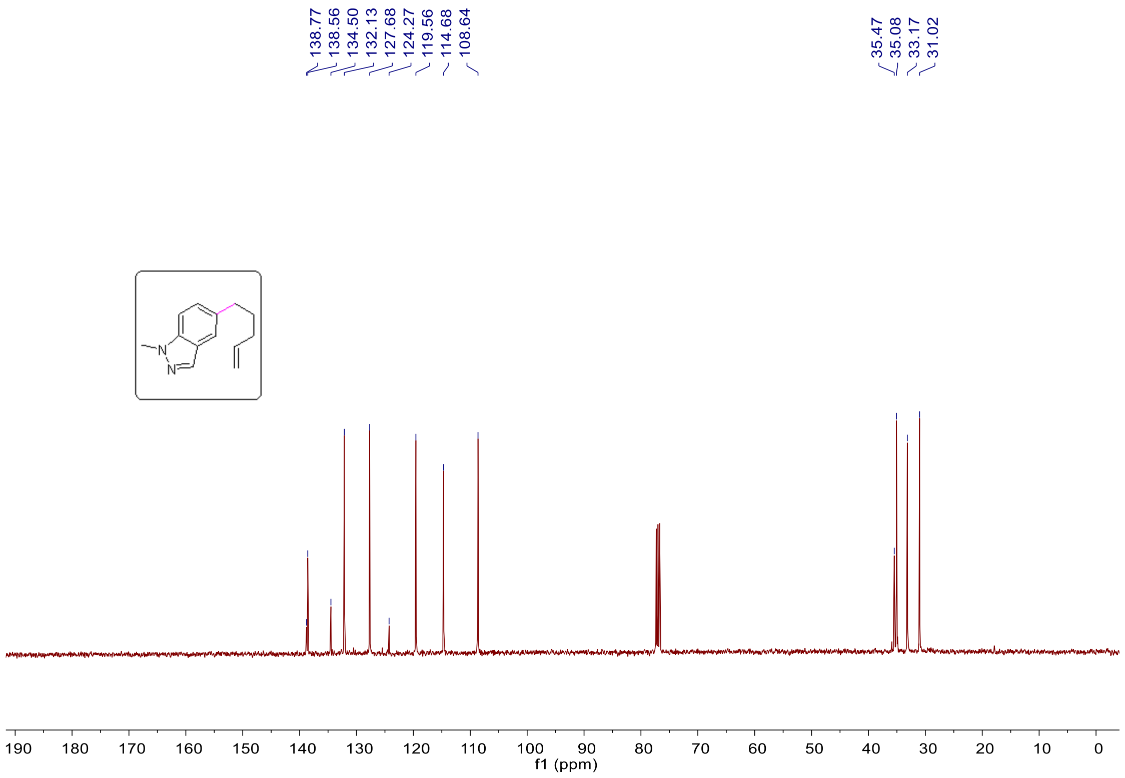

Supplementary Figure 241. ^{13}C NMR (101 MHz, $CDCl_3$) of **5ah'**

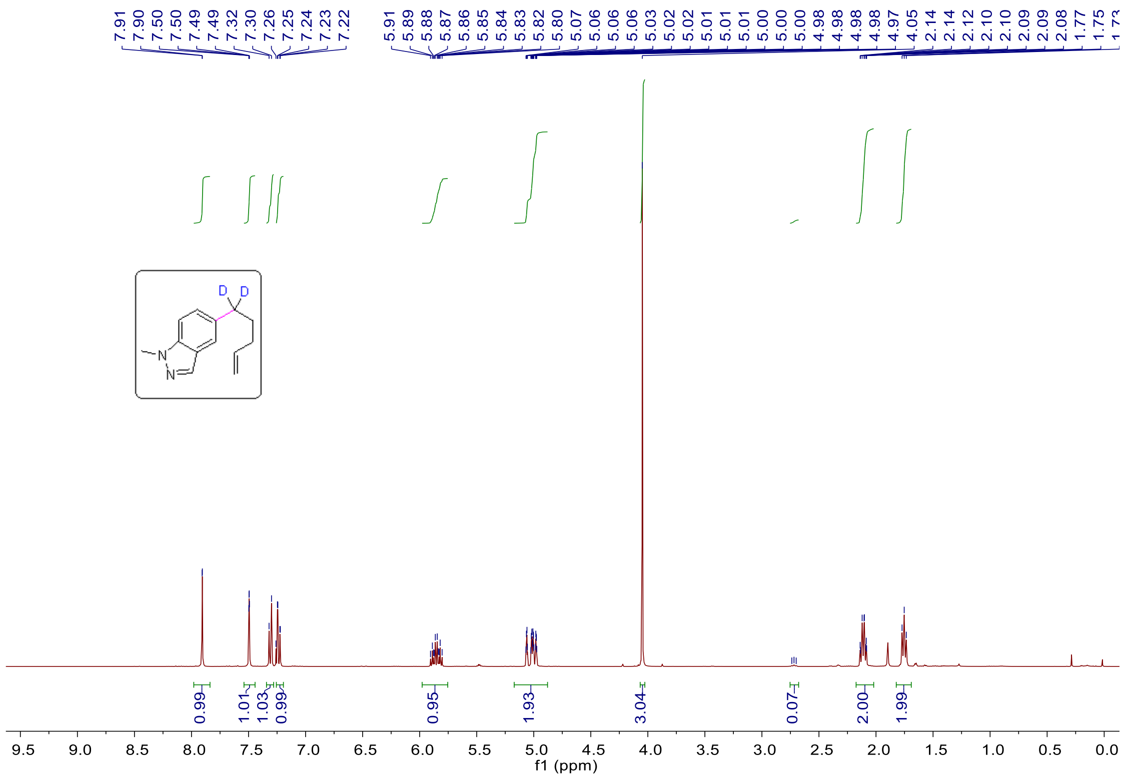

Supplementary Figure 242. ^{1}H NMR (400 MHz, $CDCl_3$) of **5ah**

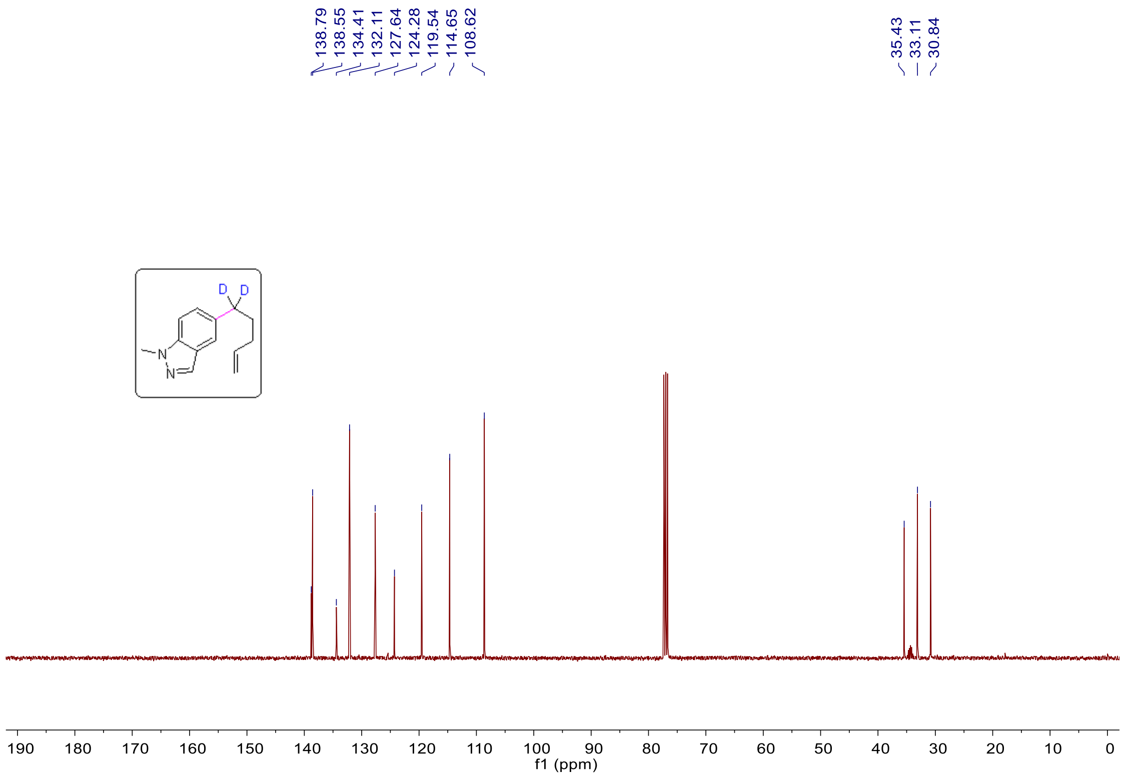

Supplementary Figure 243. ^{13}C NMR (101 MHz, $CDCl_3$) of **5ah**

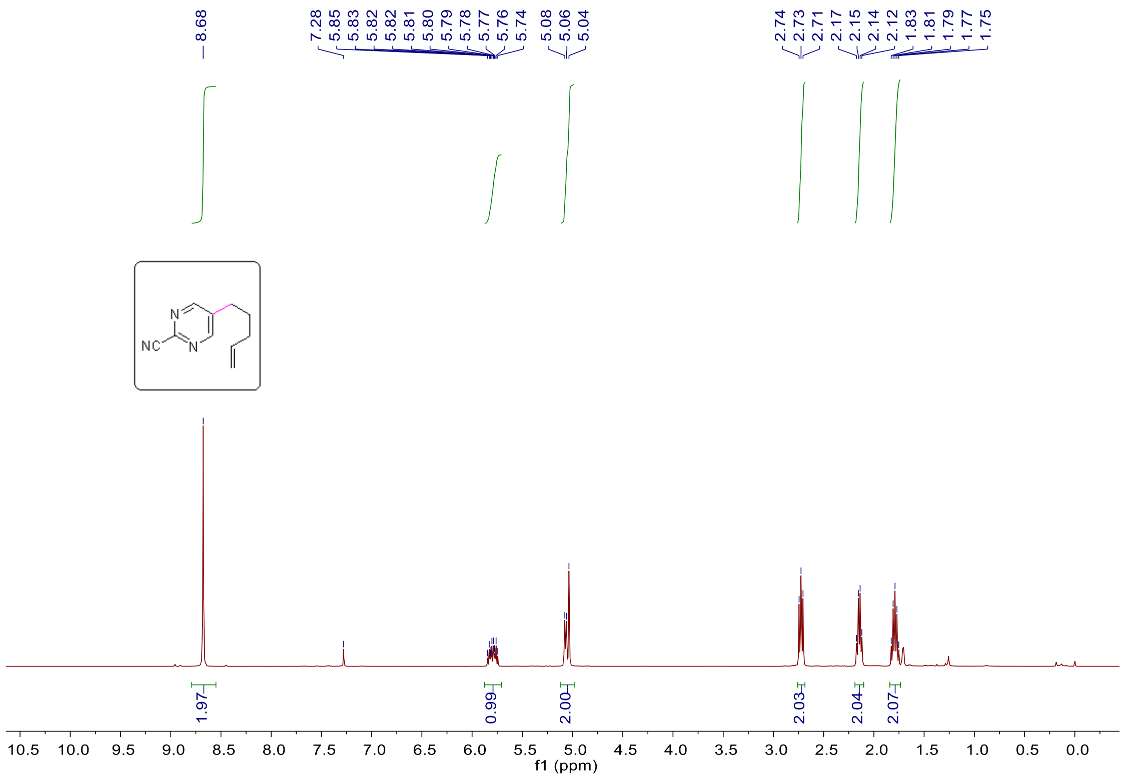

Supplementary Figure 244. ^{1}H NMR (400 MHz, $CDCl_3$) of **5ai'**

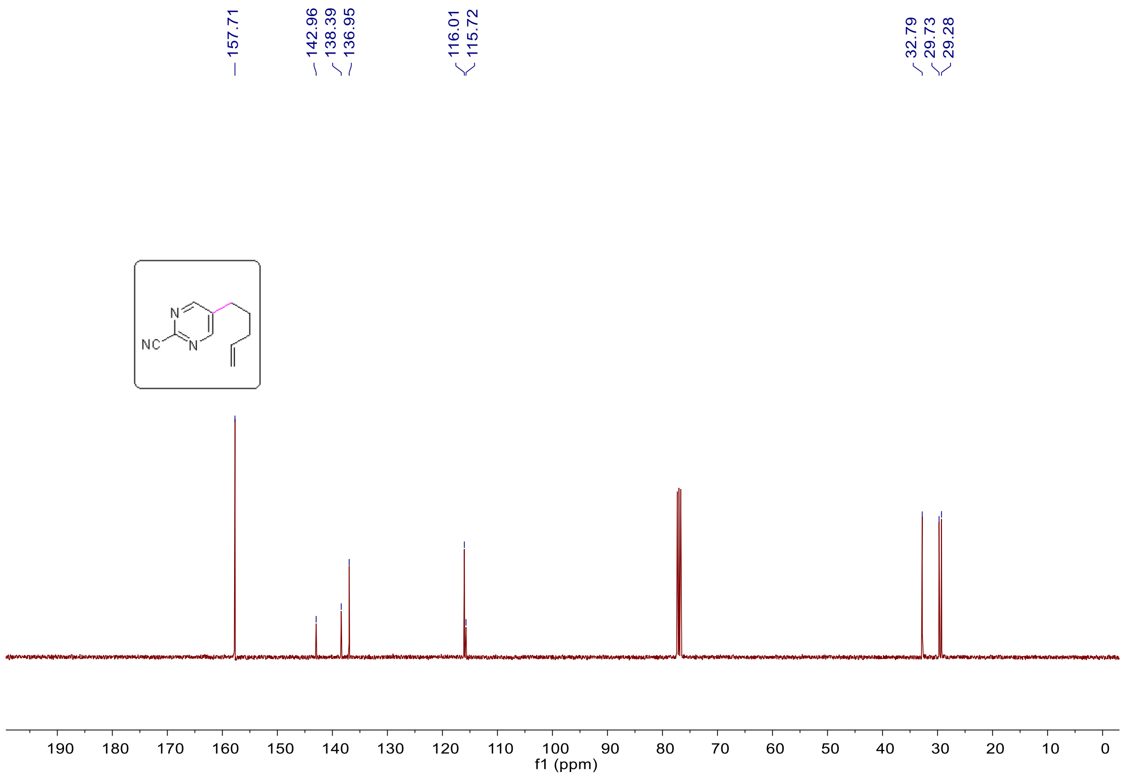

Supplementary Figure 245. ^{13}C NMR (101 MHz, $CDCl_3$) of **5ai'**

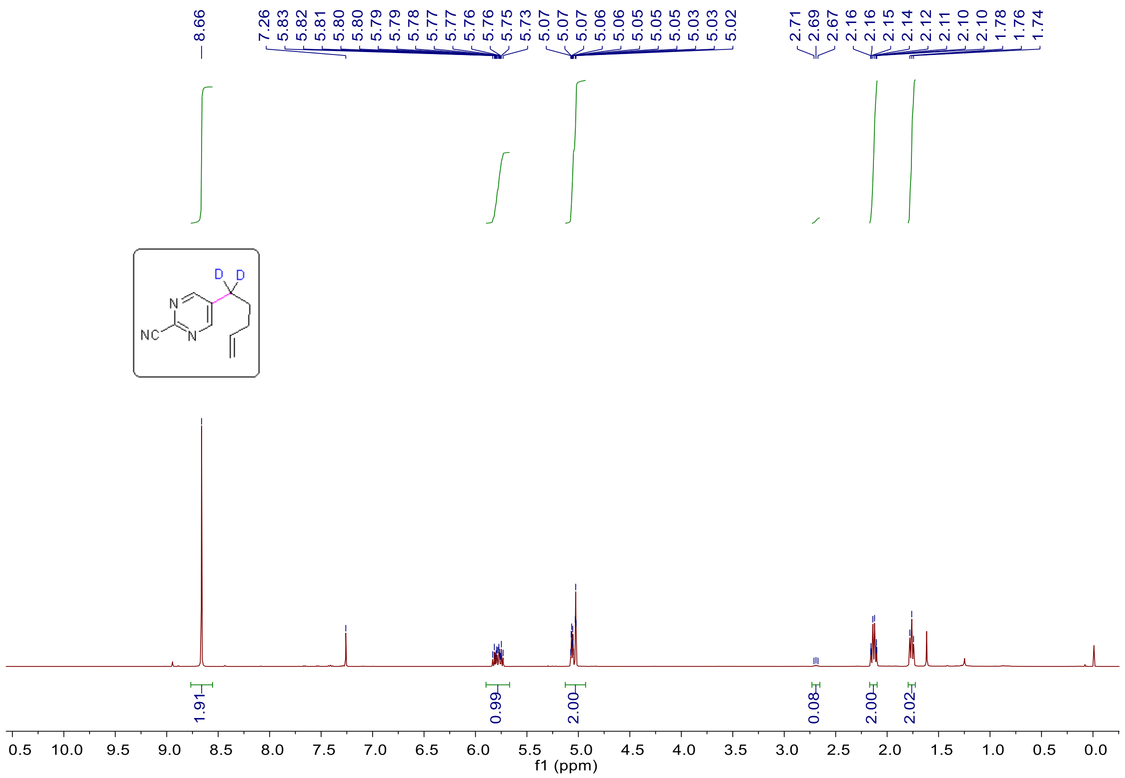

Supplementary Figure 246. ^{1}H NMR (400 MHz, $CDCl_3$) of **5ai**

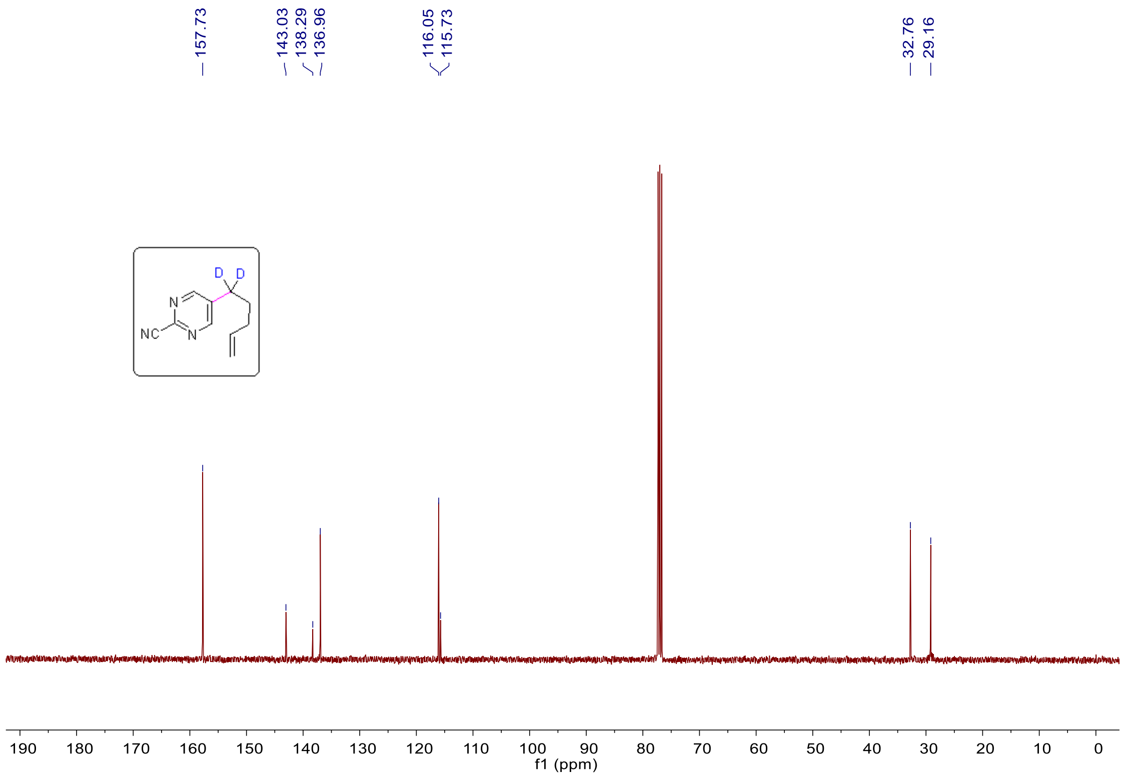

Supplementary Figure 247. ^{13}C NMR (101 MHz, $CDCl_3$) of **5ai**

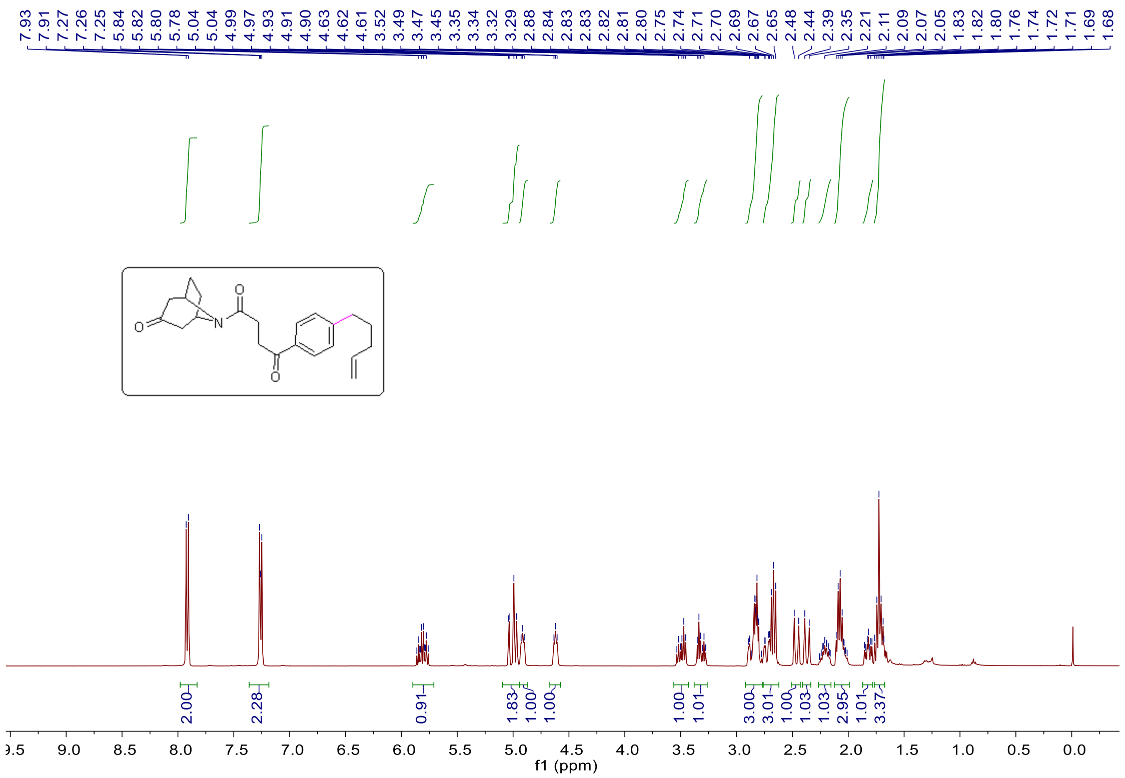

Supplementary Figure 248. ^{1}H NMR (400 MHz, $CDCl_3$) of **5aj'**

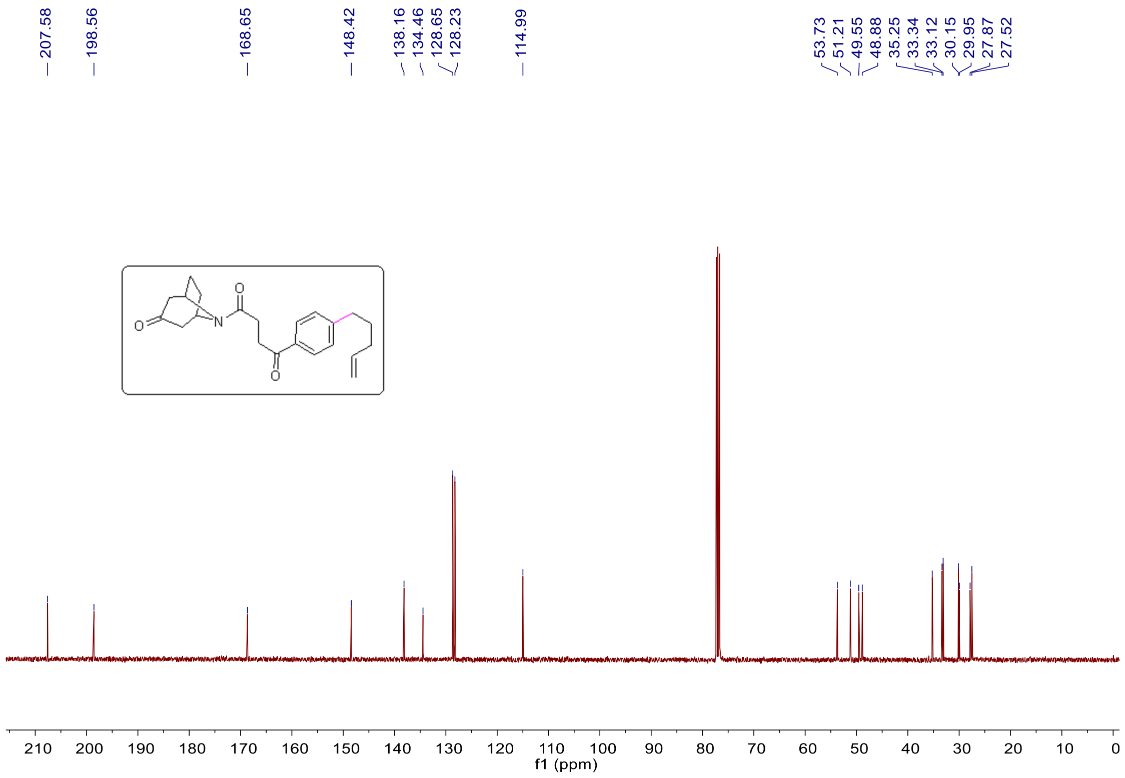

Supplementary Figure 249. ^{13}C NMR (101 MHz, $CDCl_3$) of **5aj'**

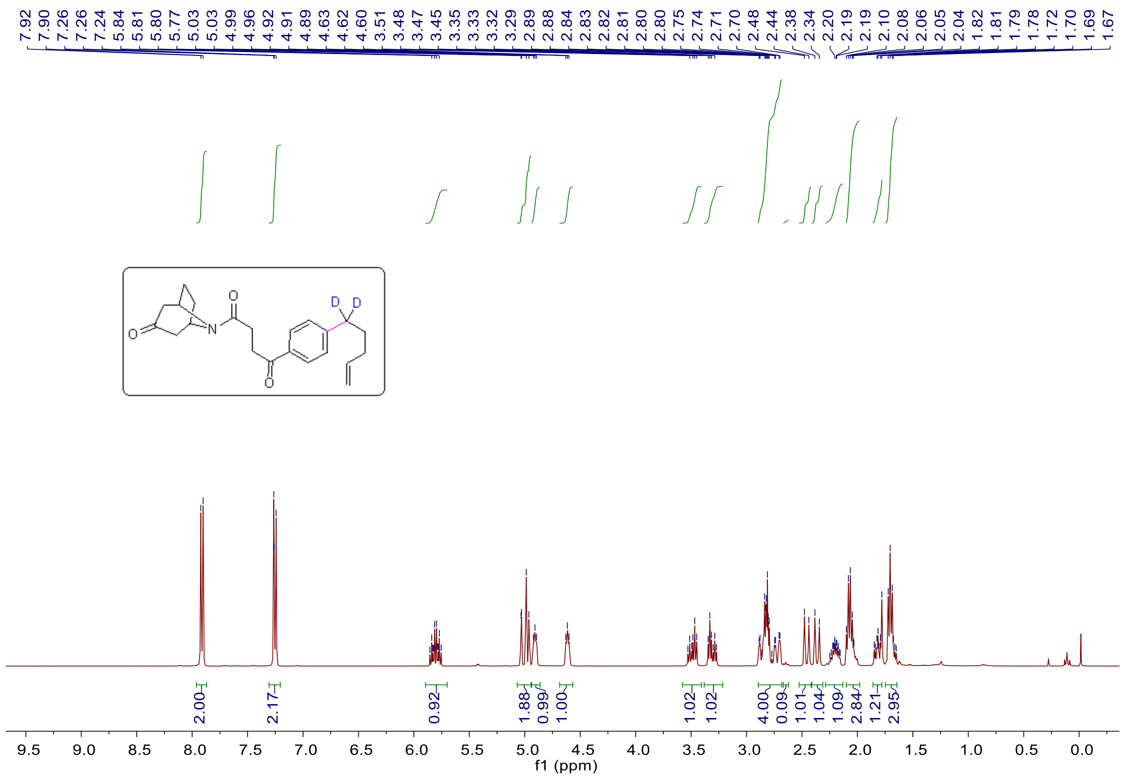

Supplementary Figure 250. ^{1}H NMR (400 MHz, $CDCl_3$) of **5aj**

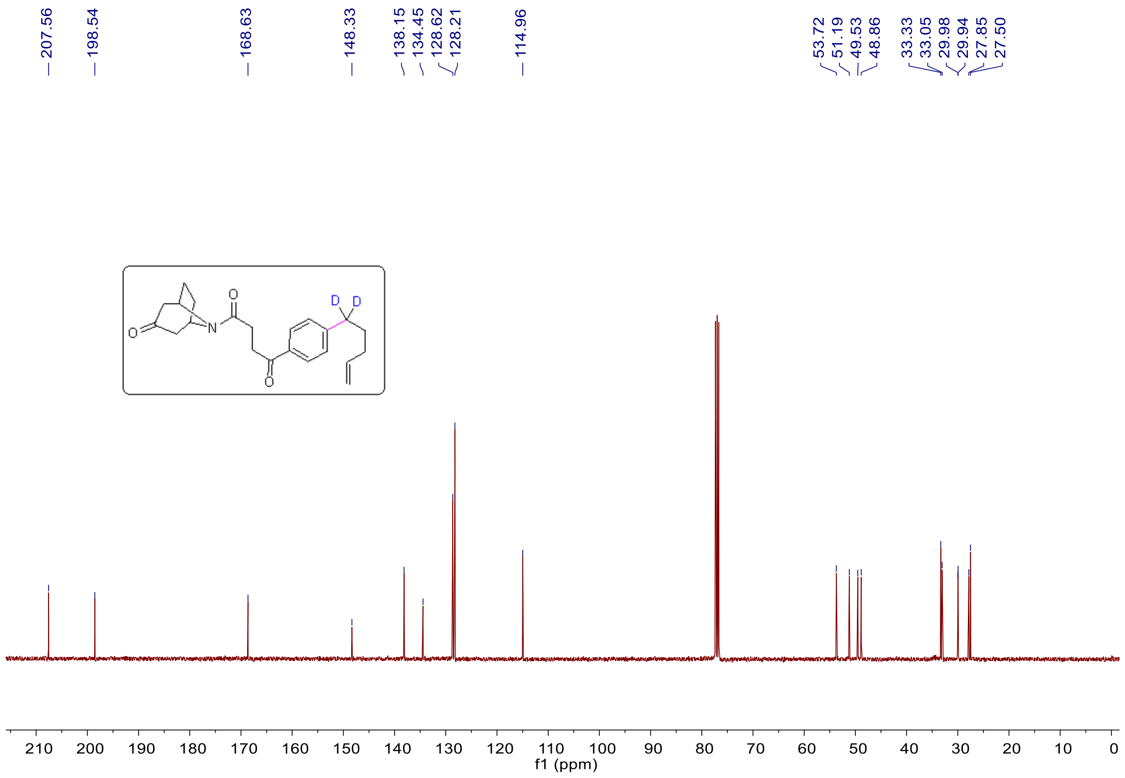

Supplementary Figure 251. ^{13}C NMR (101 MHz, $CDCl_3$) of **5aj**

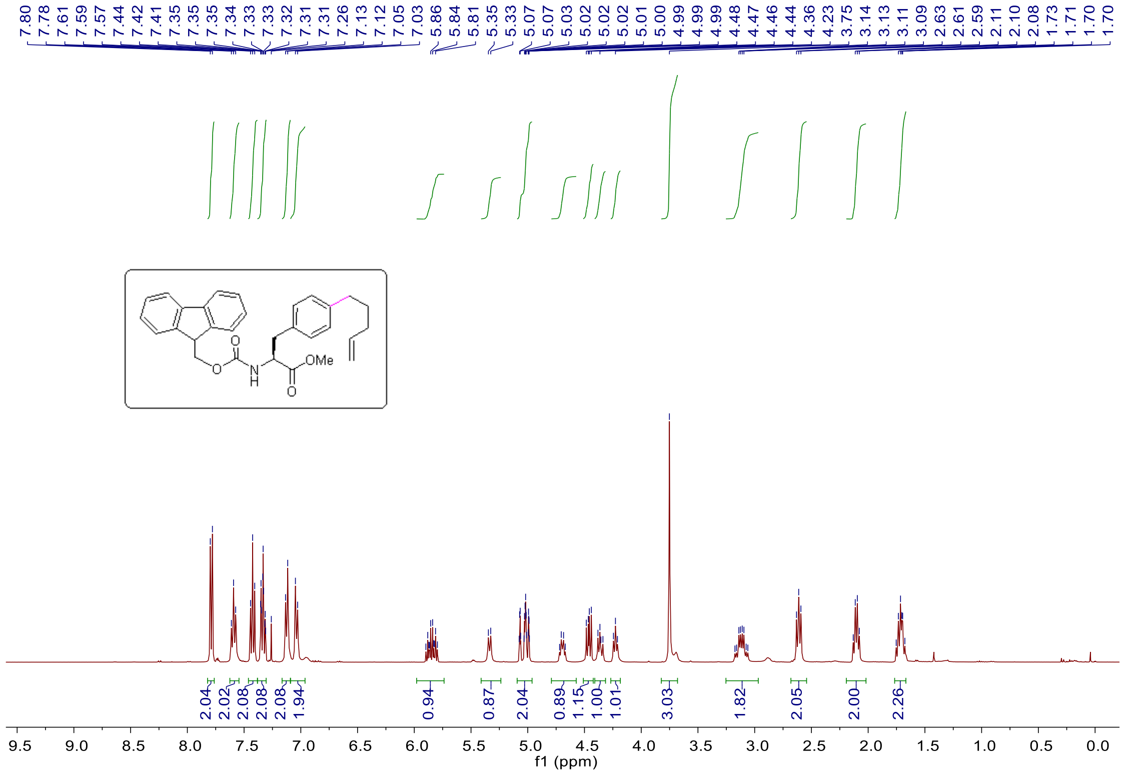

Supplementary Figure 252. ^{1}H NMR (400 MHz, $CDCl_3$) of **5ak'**

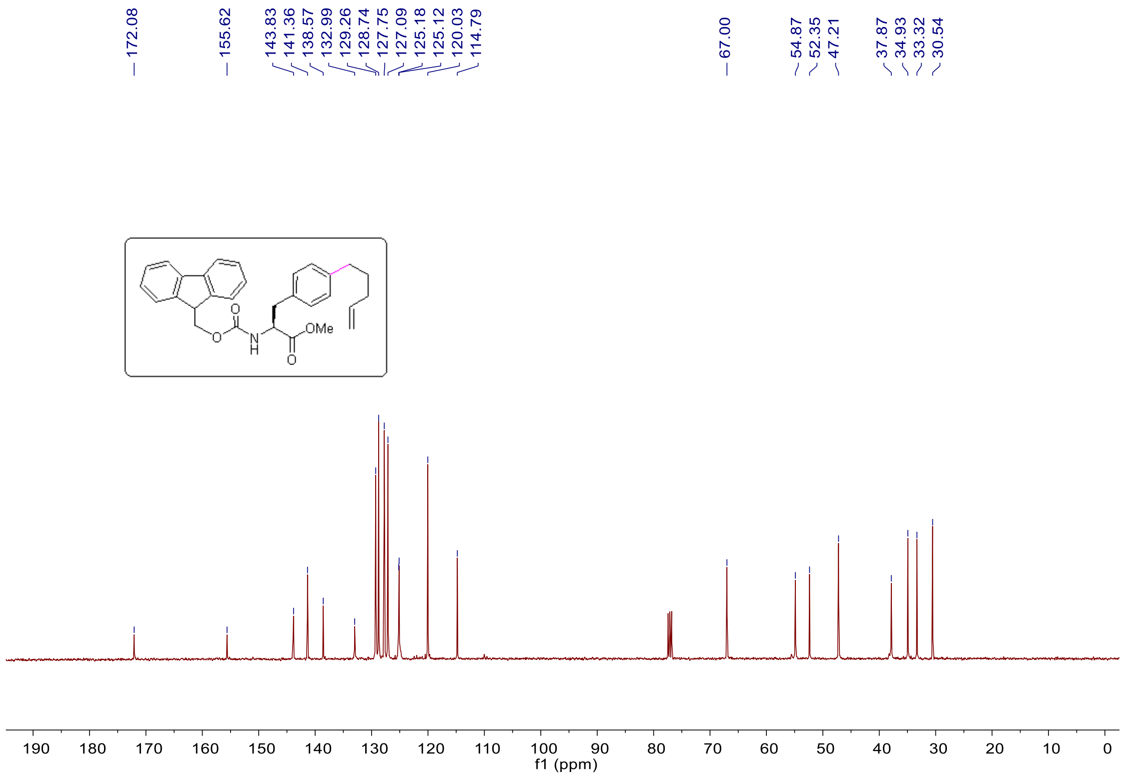

Supplementary Figure 253. ^{13}C NMR (101 MHz, $CDCl_3$) of **5ak'**

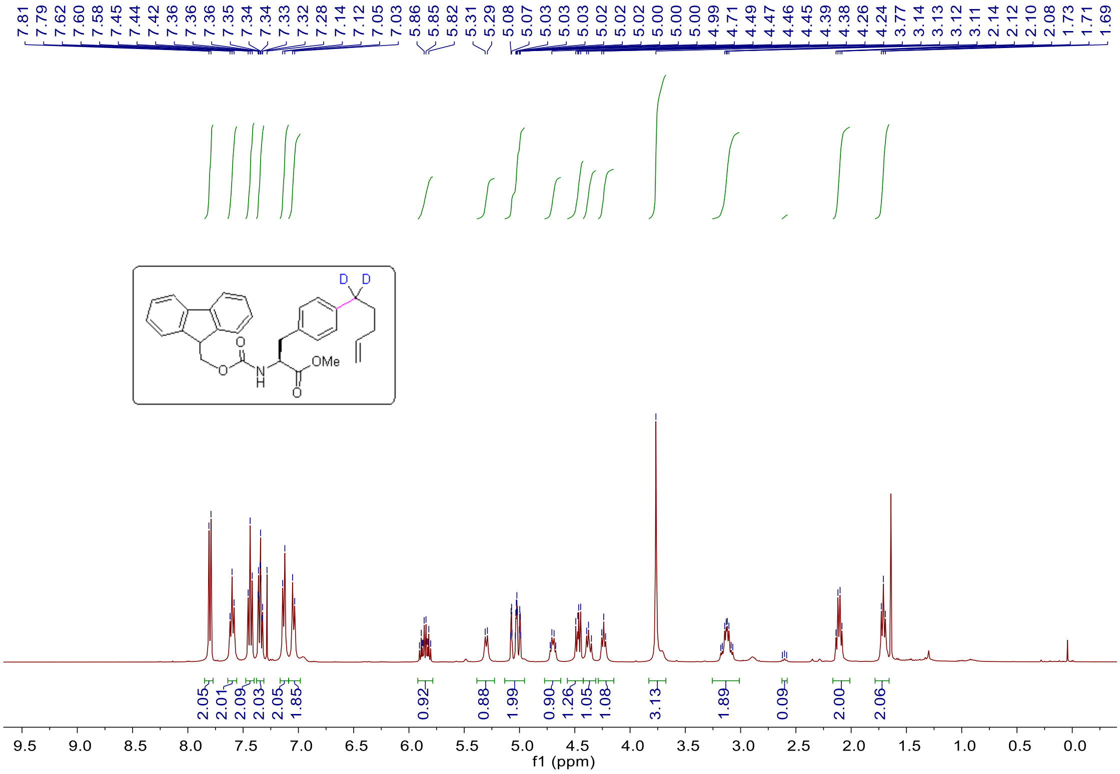

Supplementary Figure 254. ^{1}H NMR (400 MHz, $CDCl_3$) of **5ak**

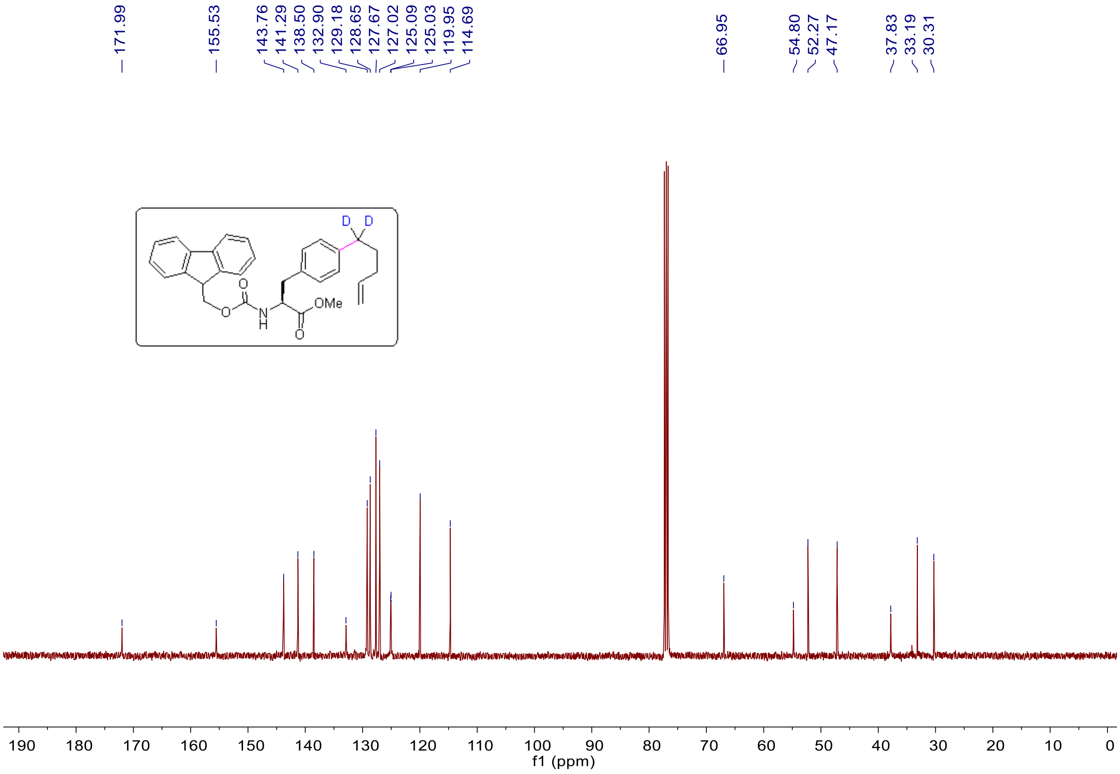

Supplementary Figure 255. ^{13}C NMR (101 MHz, $CDCl_3$) of **5ak**

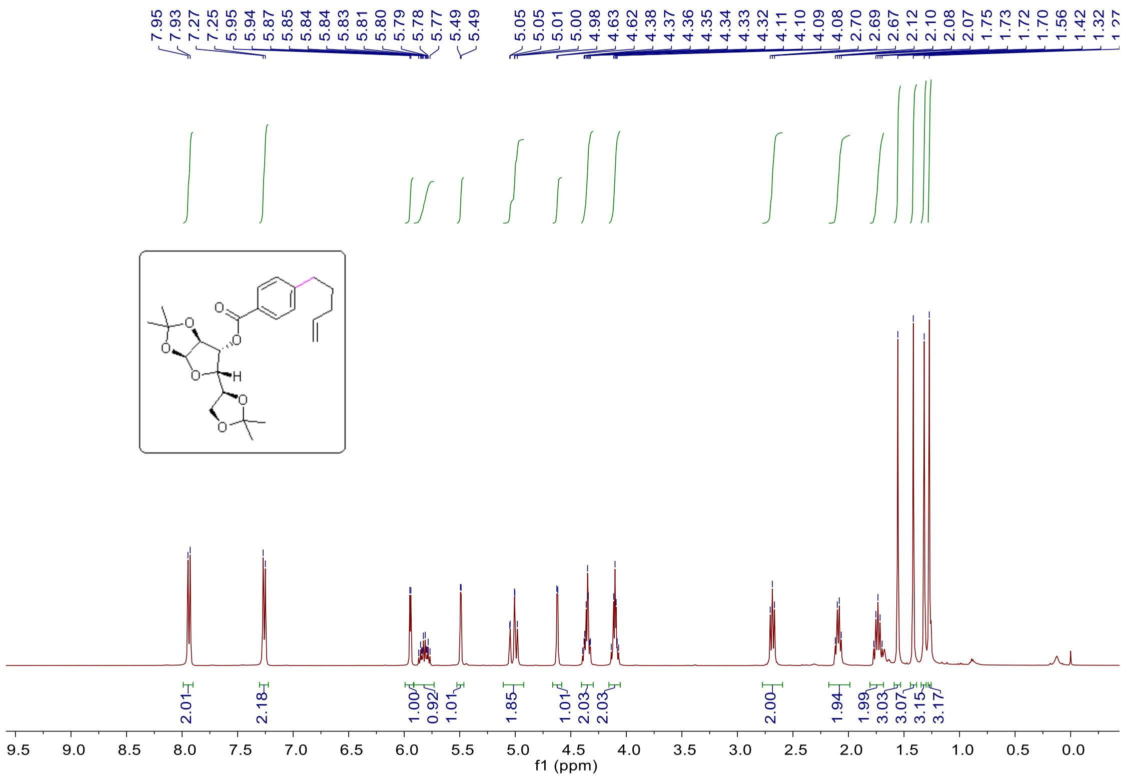

Supplementary Figure 256. ^{1}H NMR (400 MHz, $CDCl_3$) of **5al'**

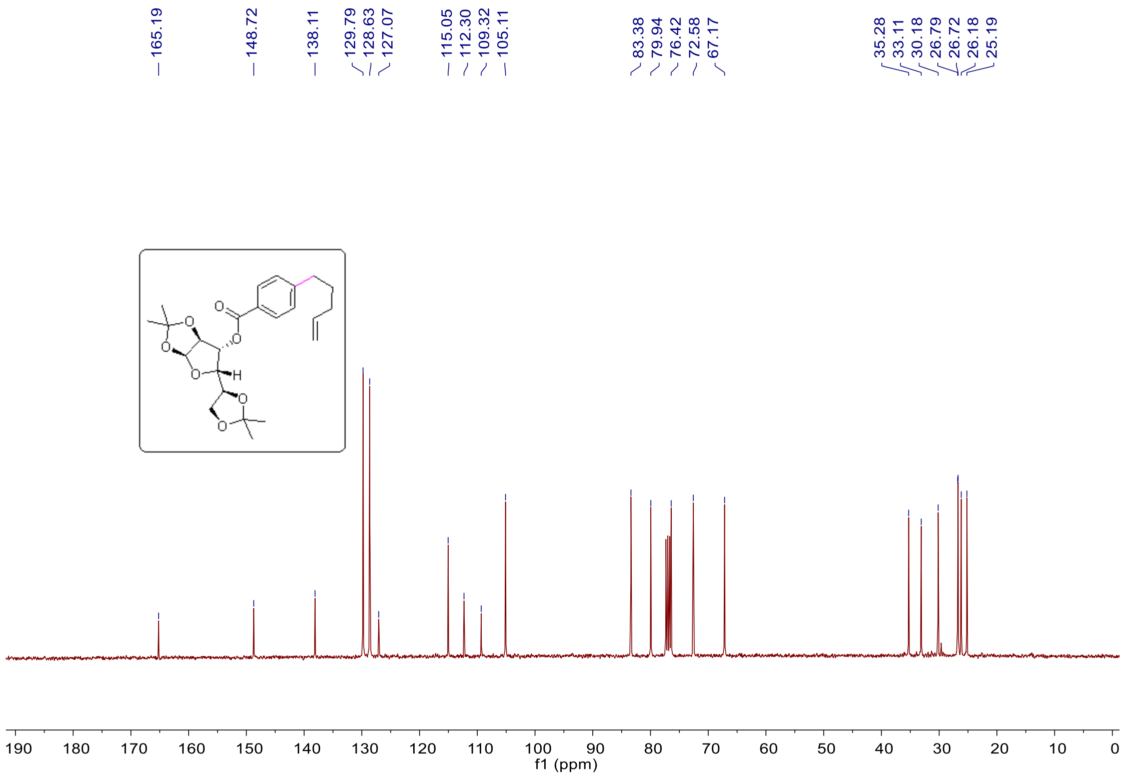

Supplementary Figure 257. ^{13}C NMR (101 MHz, $CDCl_3$) of **5al'**

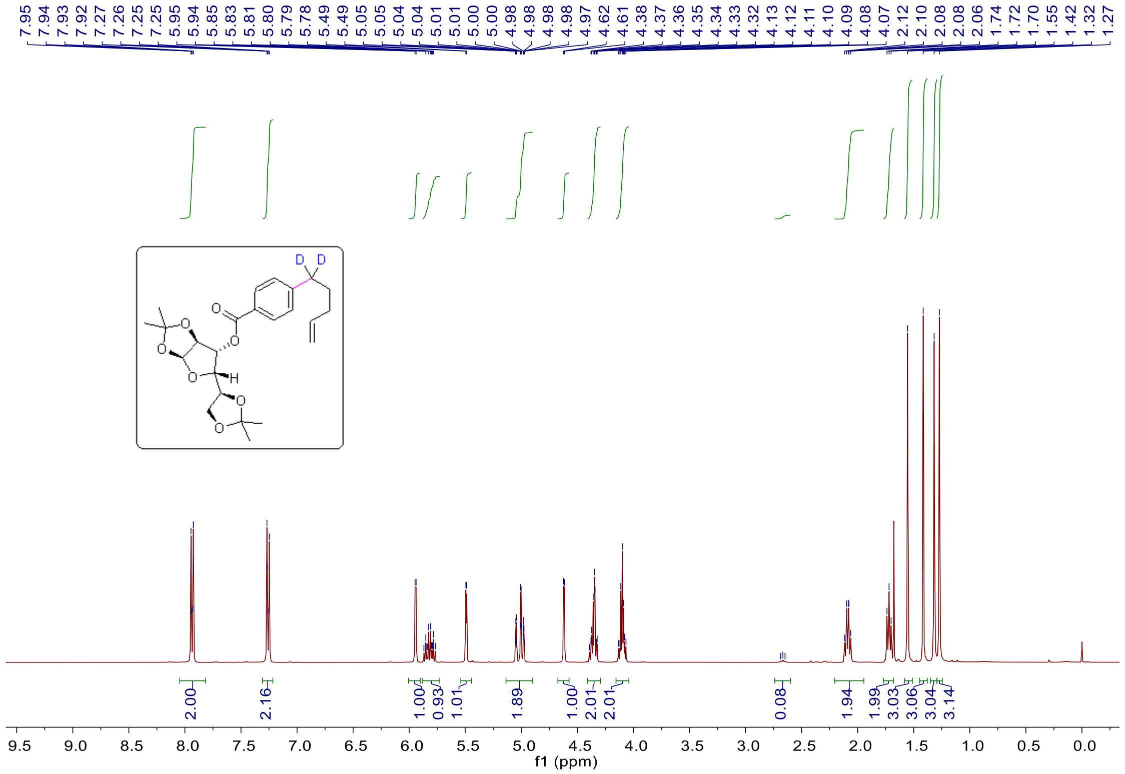

Supplementary Figure 258. ^{1}H NMR (400 MHz, $CDCl_3$) of **5al**

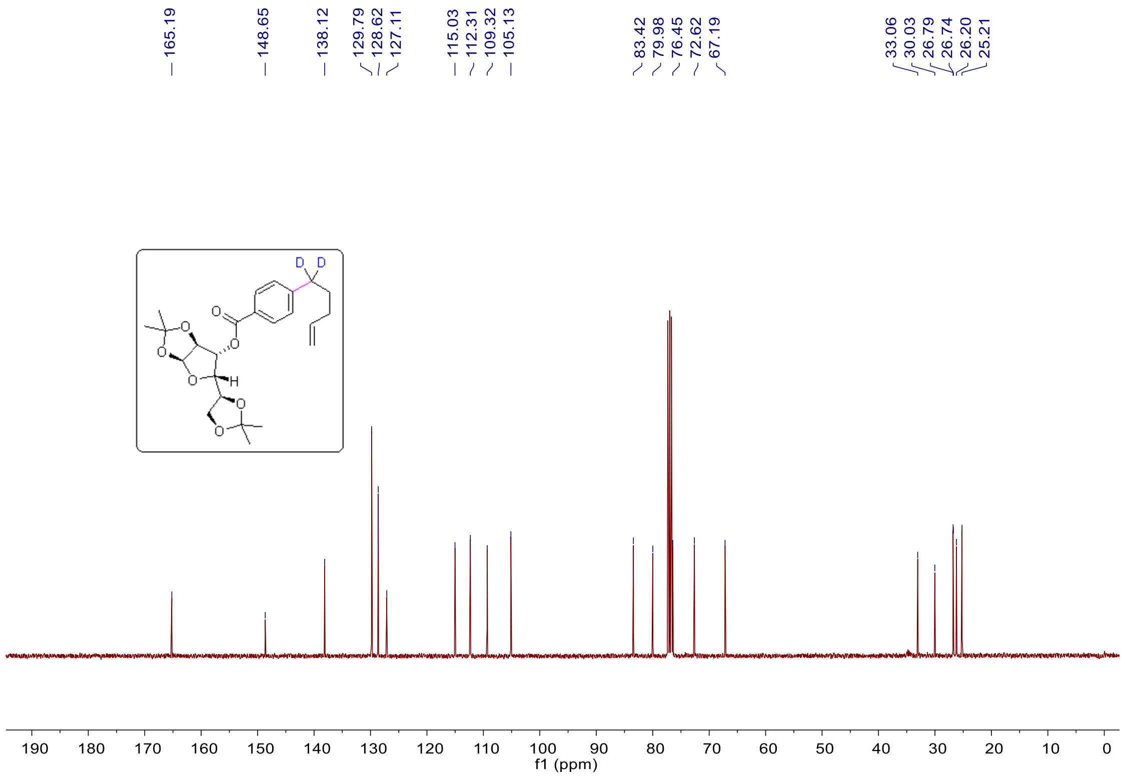

Supplementary Figure 259. ^{13}C NMR (101 MHz, $CDCl_3$) of **5al**

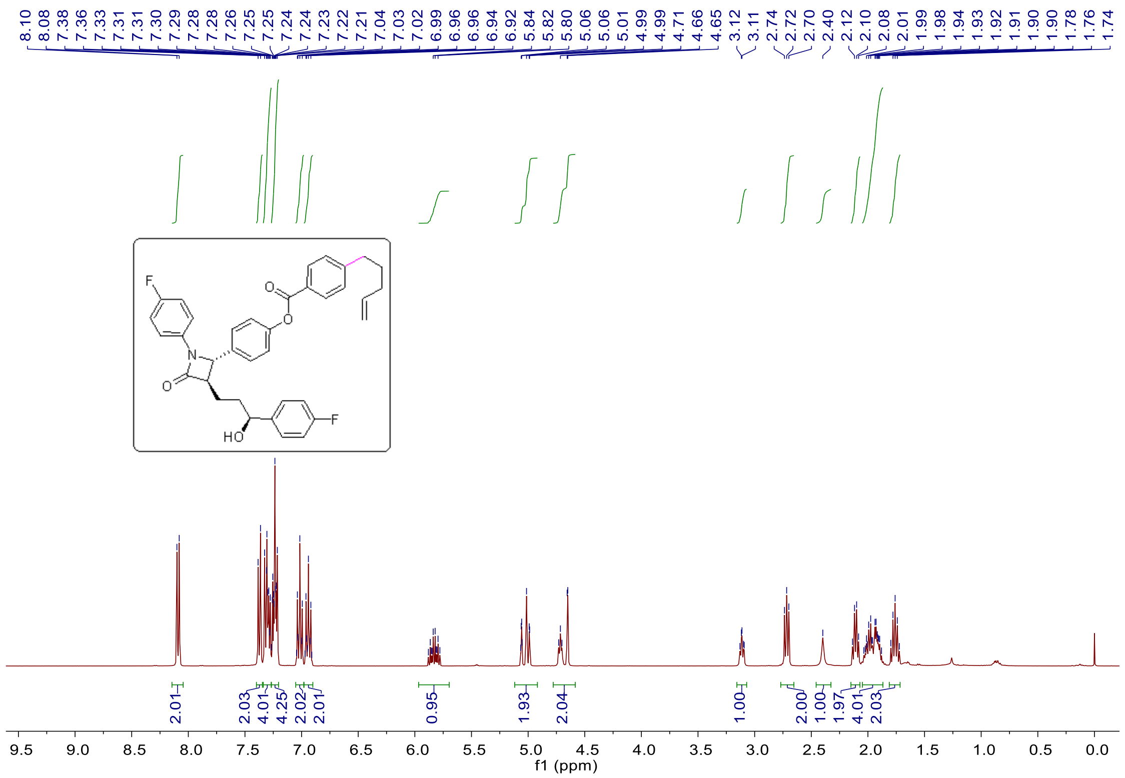

Supplementary Figure 260. ^{1}H NMR (400 MHz, $CDCl_3$) of **5am'**

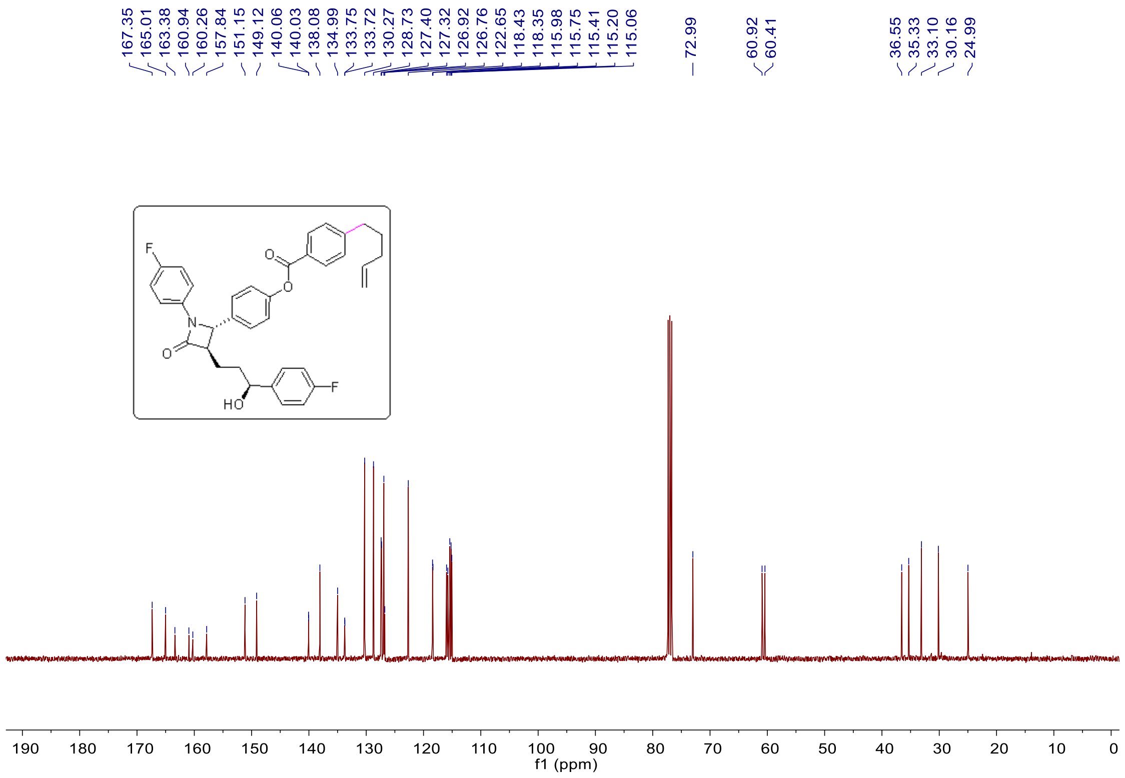

Supplementary Figure 261. ^{13}C NMR (101 MHz, $CDCl_3$) of **5am'**

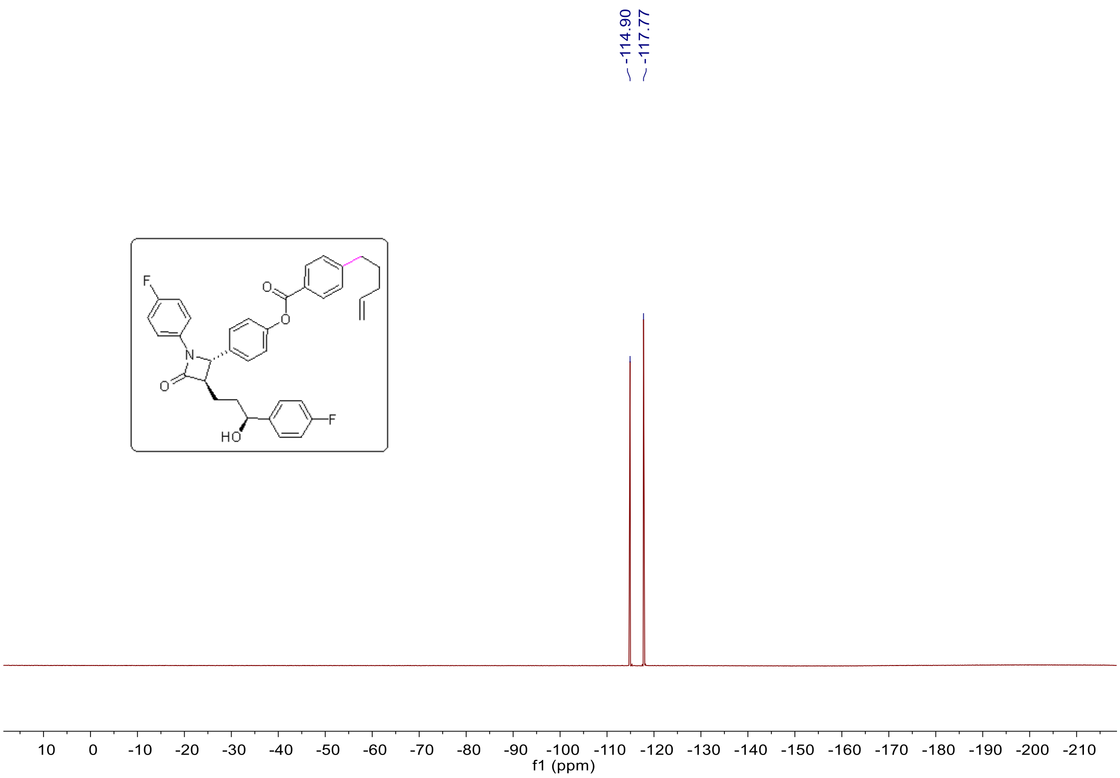

Supplementary Figure 262. ^{19}F NMR (376 MHz, $CDCl_3$) of **5am'**

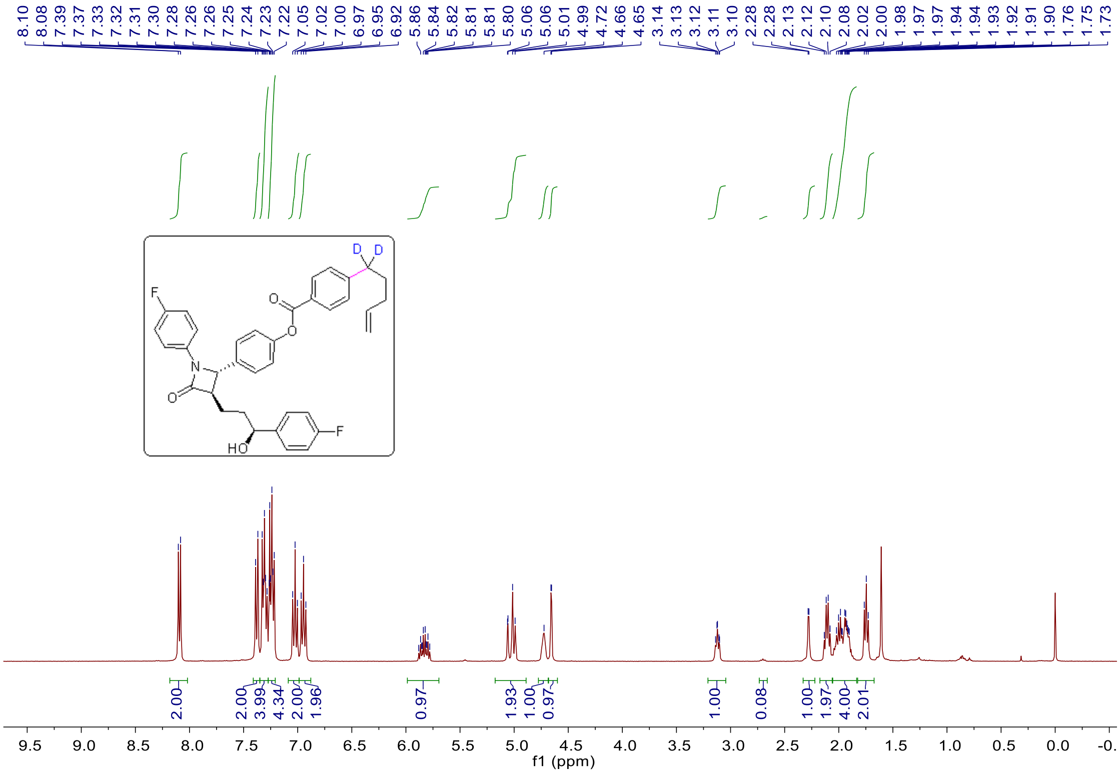

Supplementary Figure 263. ^{1}H NMR (400 MHz, $CDCl_3$) of **5am**

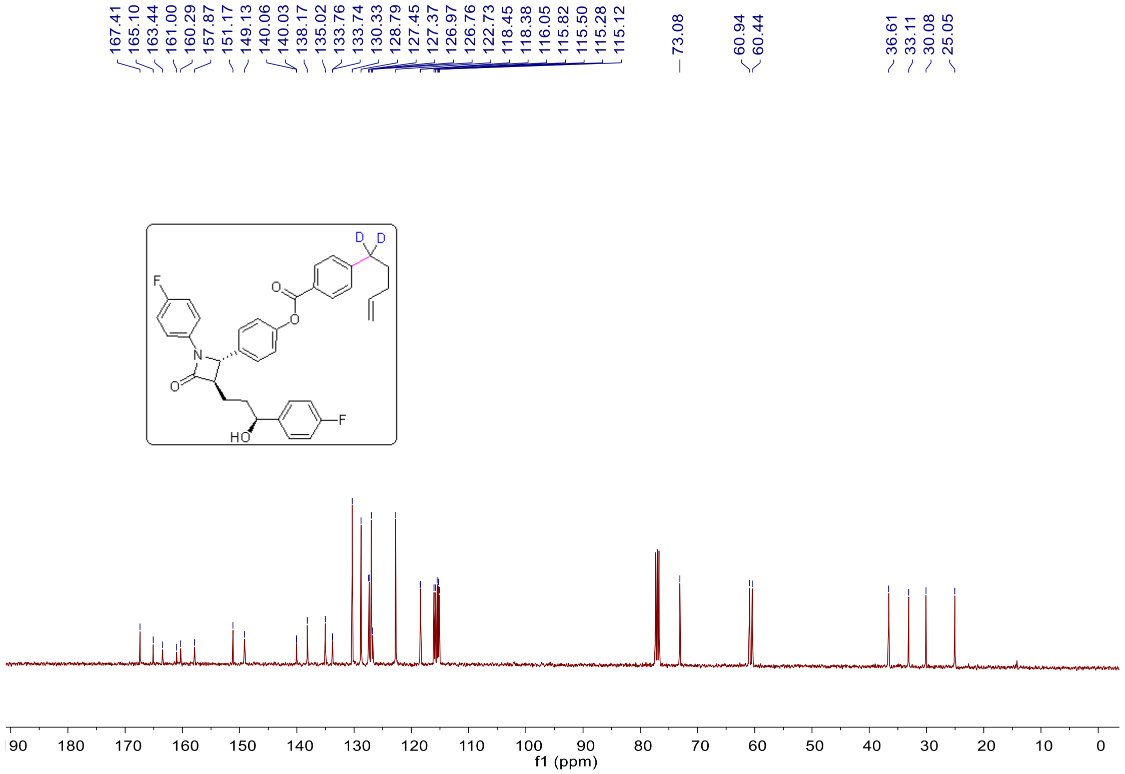

Supplementary Figure 264. ^{13}C NMR (101 MHz, $CDCl_3$) of **5am**

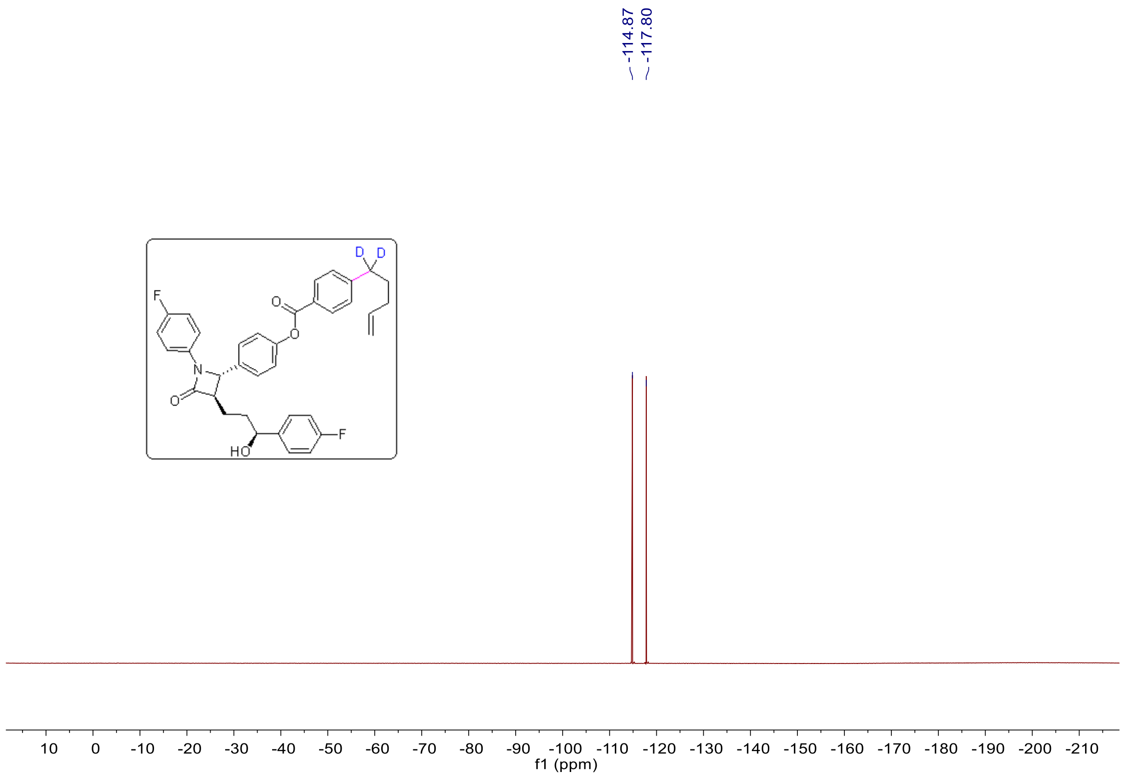

Supplementary Figure 265. ^{19}F NMR (376 MHz, $CDCl_3$) of **5am**

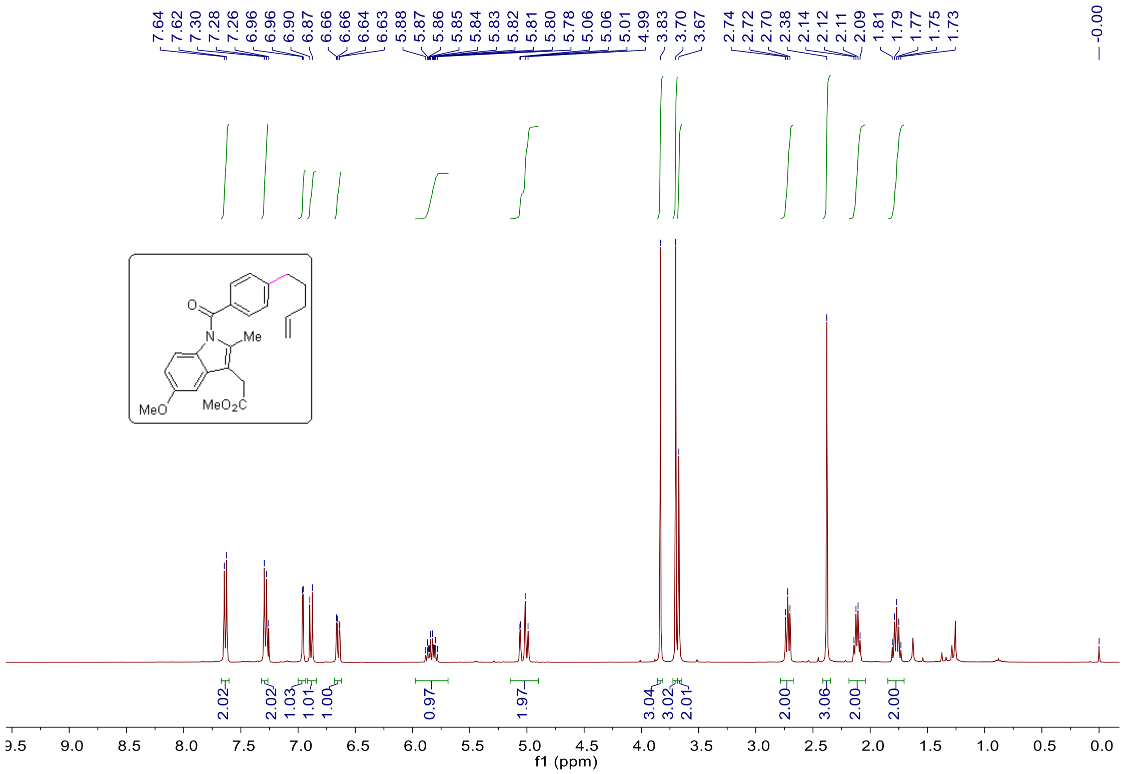

Supplementary Figure 266. ^{1}H NMR (400 MHz, $CDCl_3$) of **5an'**

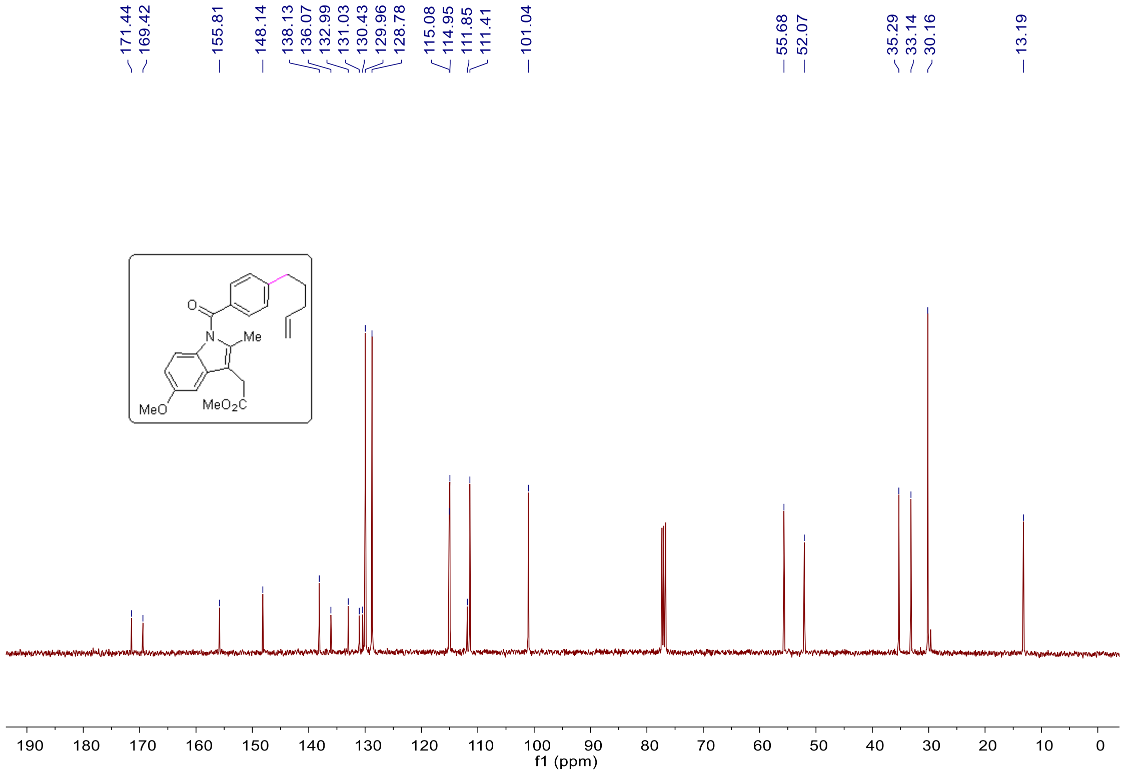

Supplementary Figure 267. ^{13}C NMR (101 MHz, $CDCl_3$) of **5an'**

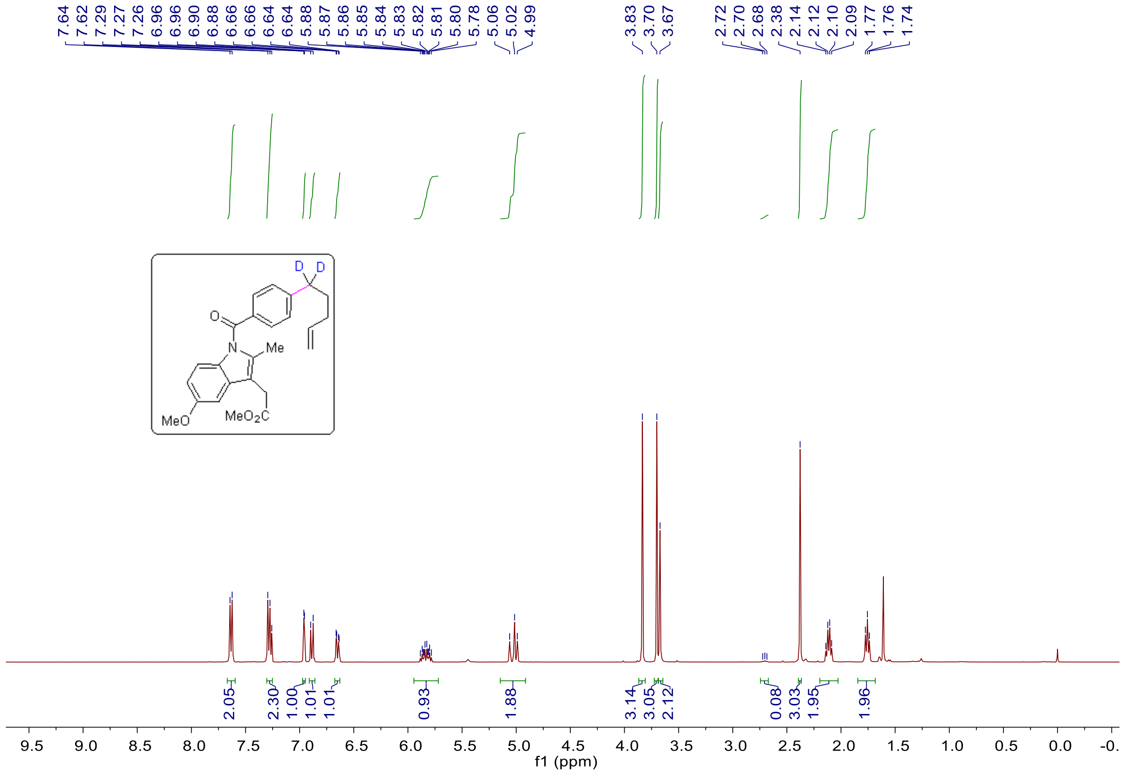

Supplementary Figure 268. ^{1}H NMR (400 MHz, $CDCl_3$) of **5an**

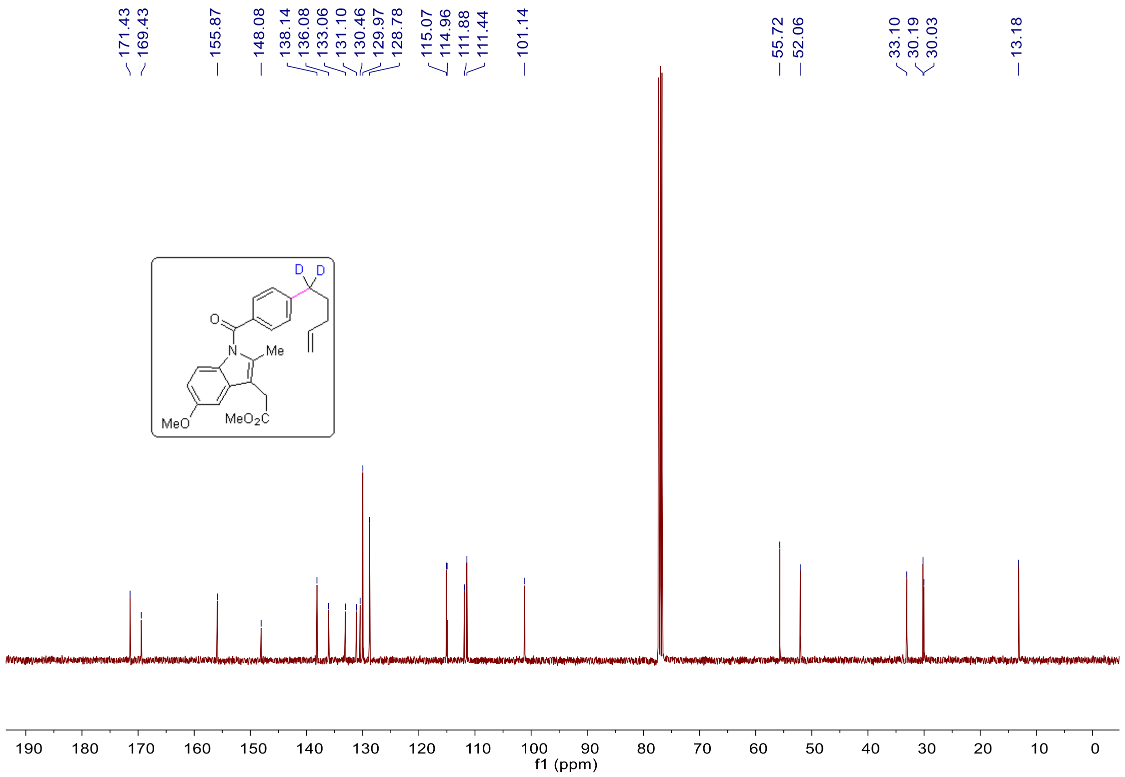

Supplementary Figure 269. ^{13}C NMR (101 MHz, $CDCl_3$) of **5an**

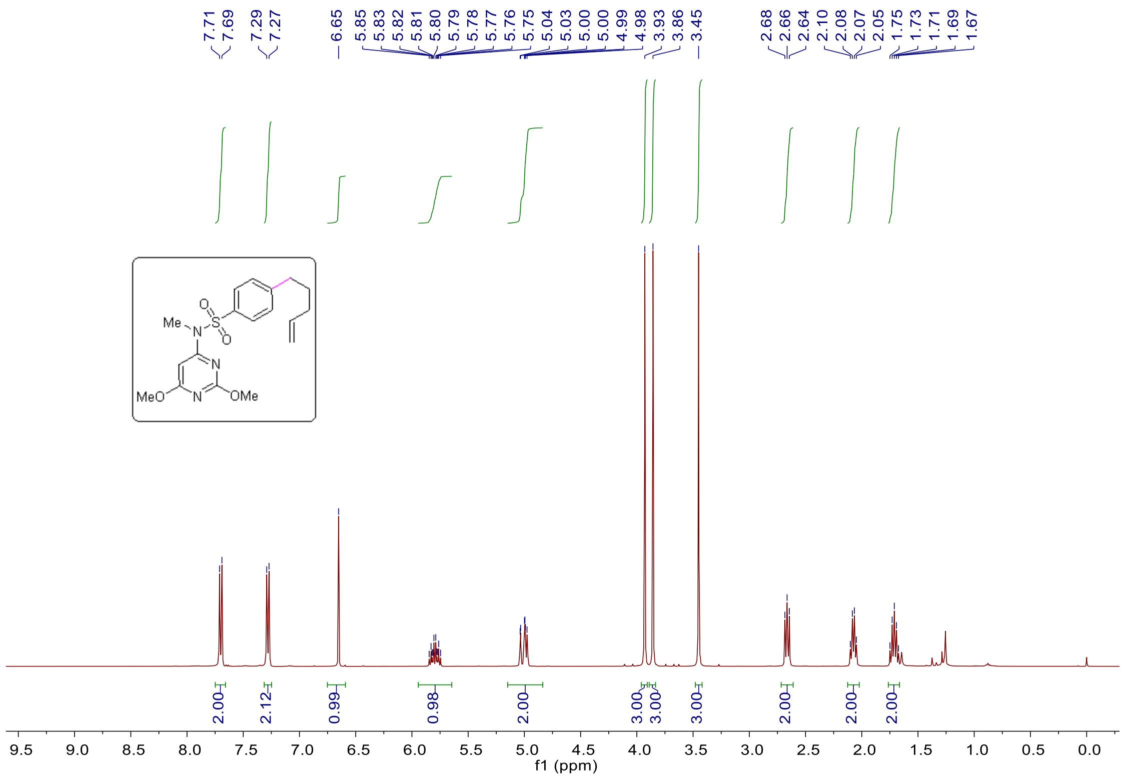

Supplementary Figure 270. ^{1}H NMR (400 MHz, $CDCl_3$) of **5ao'**

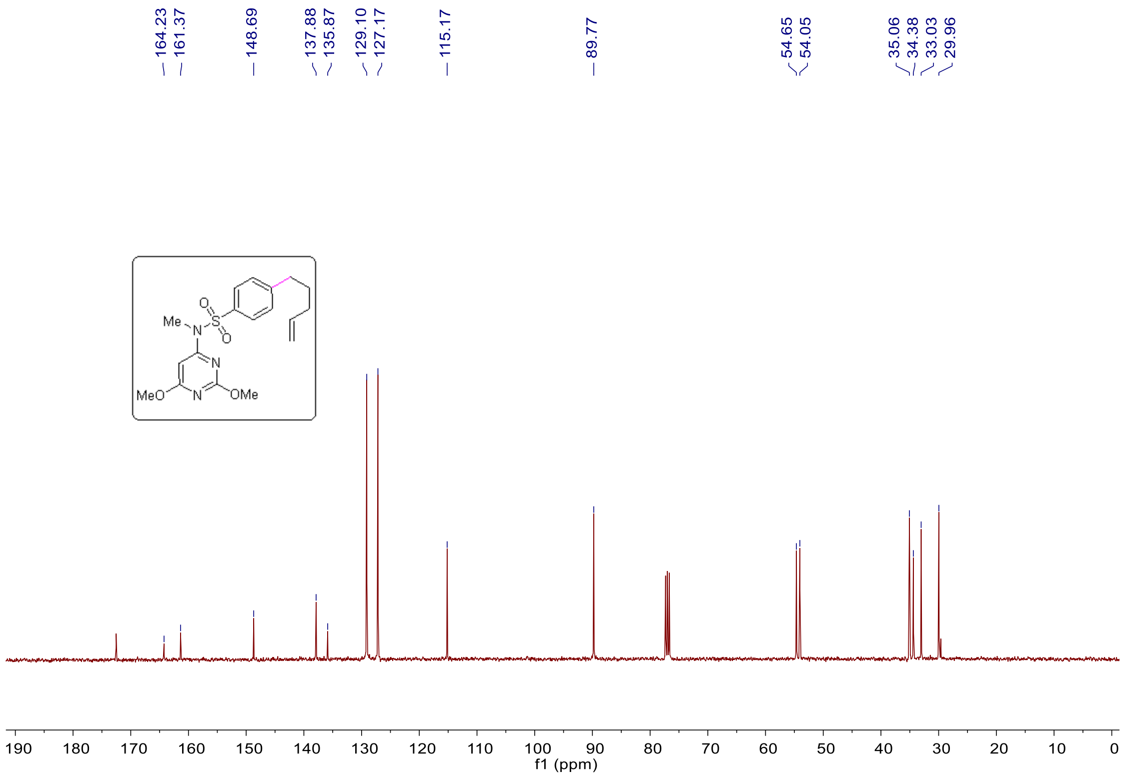

Supplementary Figure 271. ^{13}C NMR (101 MHz, $CDCl_3$) of **5ao'**

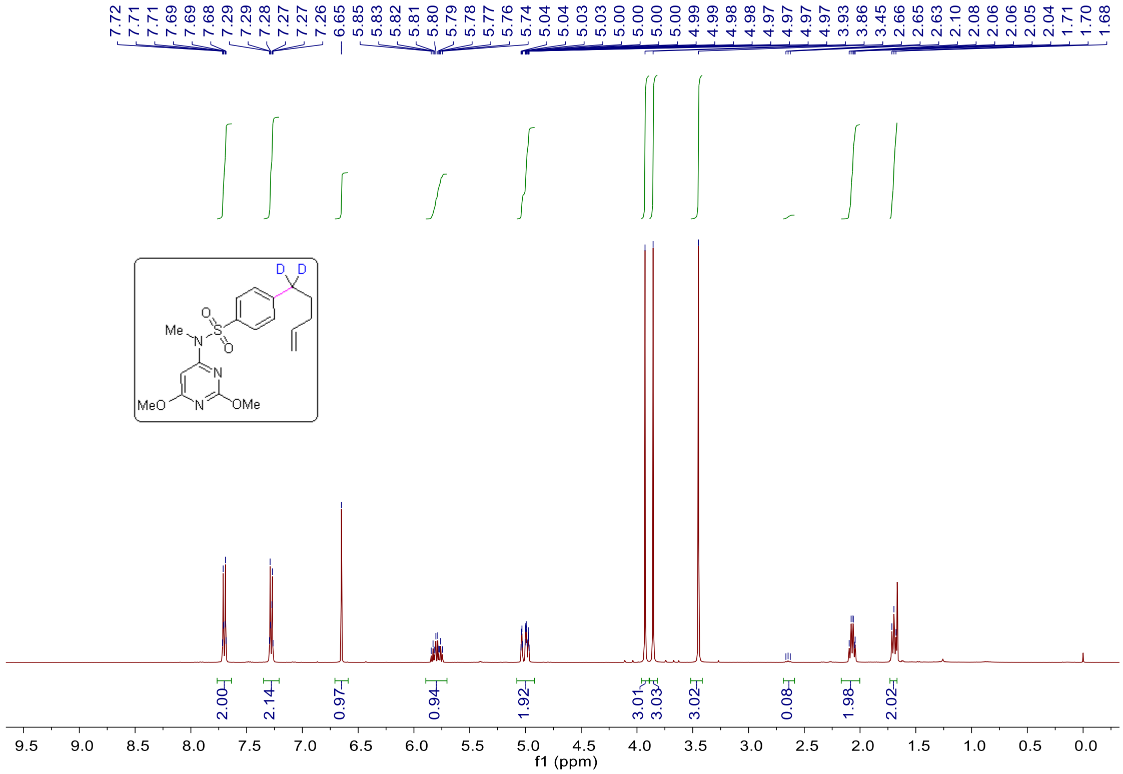

Supplementary Figure 272. ^{1}H NMR (400 MHz, $CDCl_3$) of **5ao**

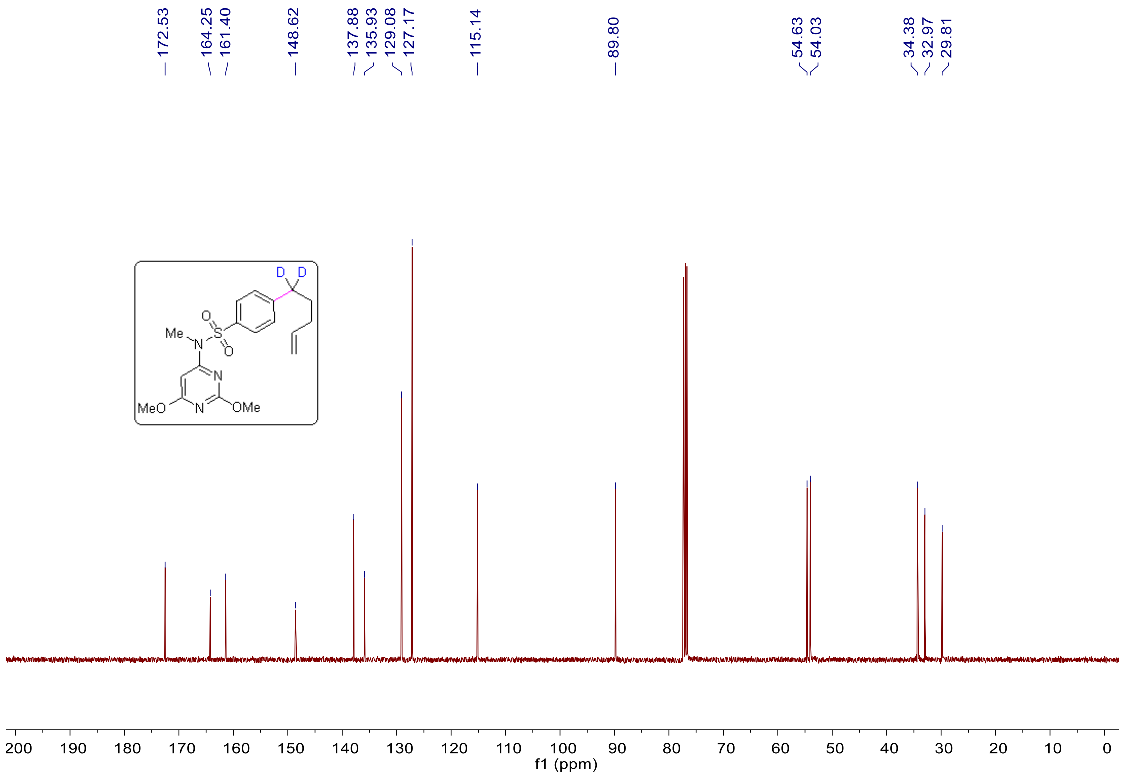

Supplementary Figure 273. ^{13}C NMR (101 MHz, $CDCl_3$) of **5ao**

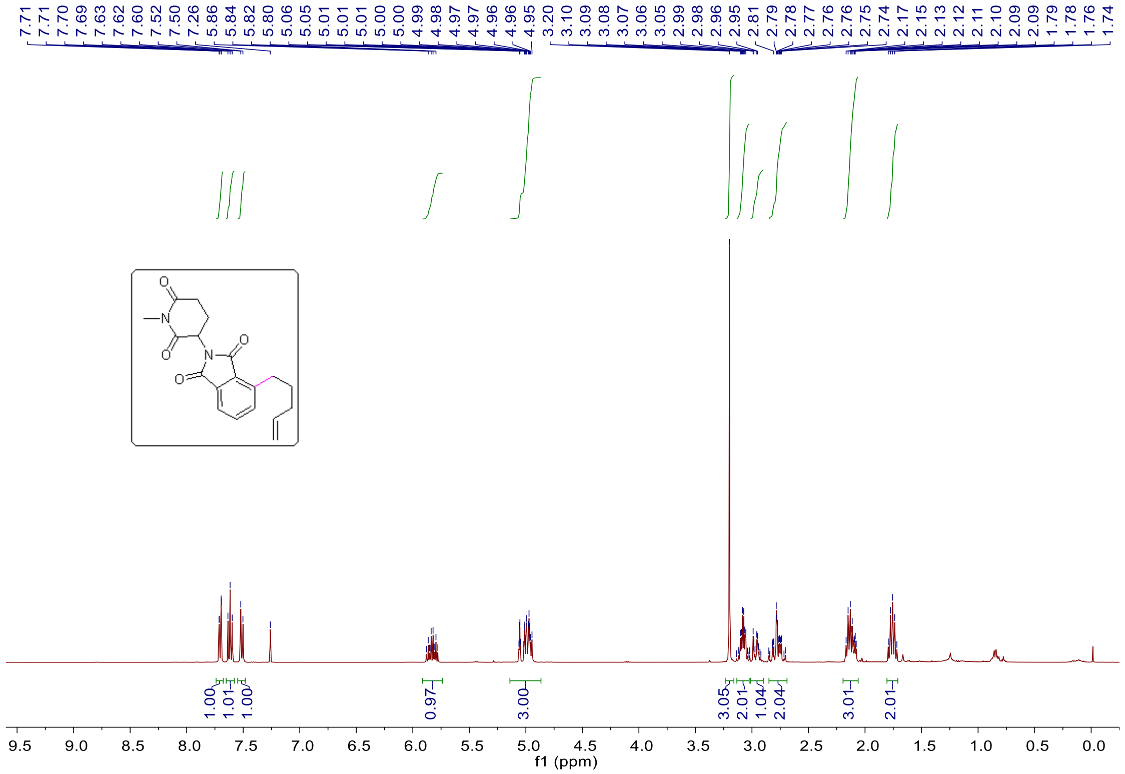

Supplementary Figure 274. ^{1}H NMR (400 MHz, $CDCl_3$) of **5ap'**

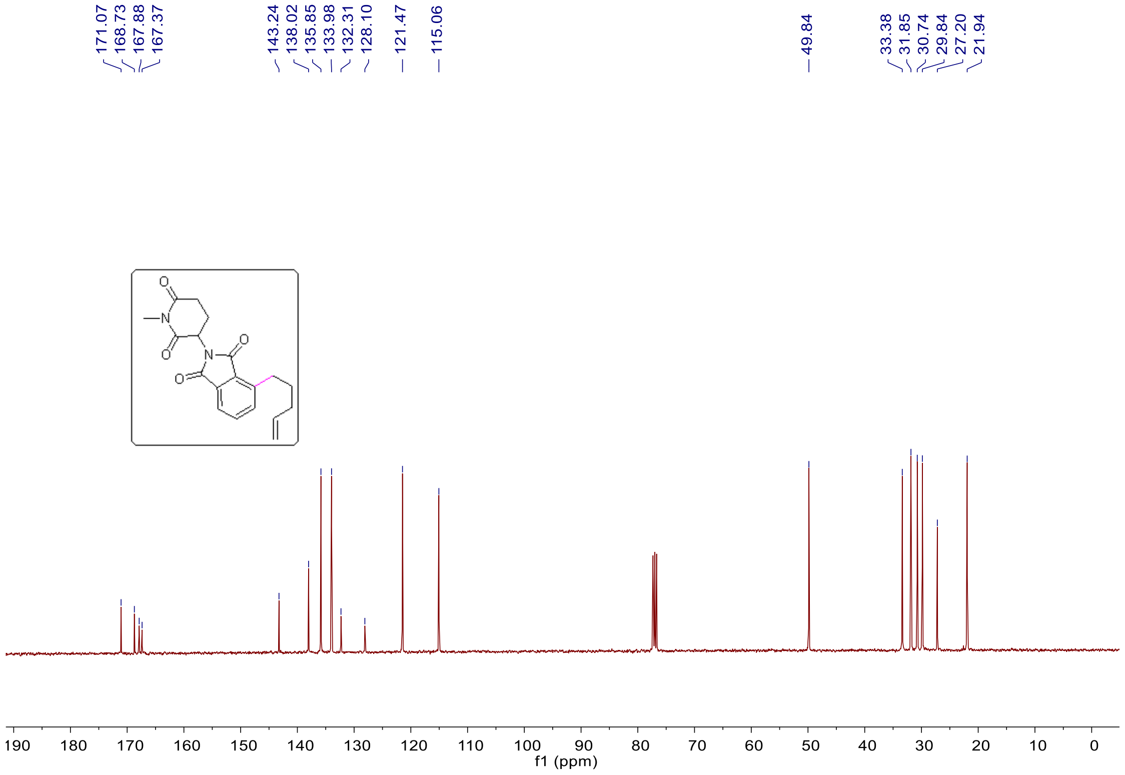

Supplementary Figure 275. ^{13}C NMR (101 MHz, $CDCl_3$) of **5ap'**

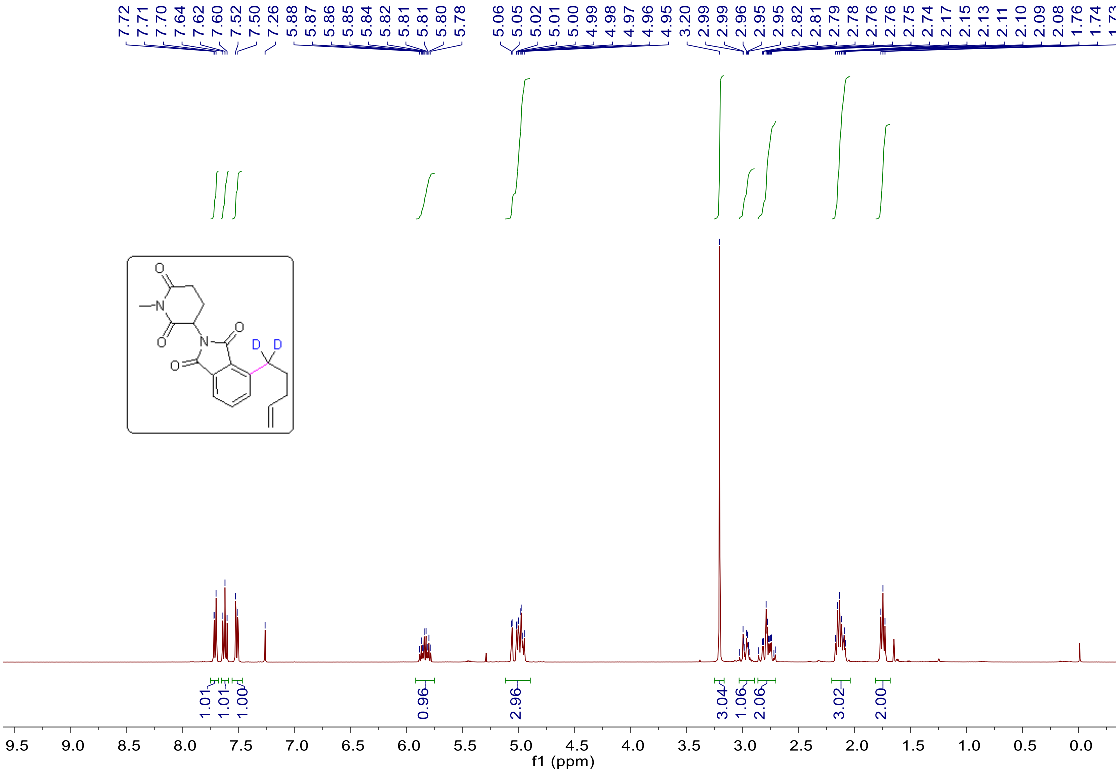

Supplementary Figure 276. ^{1}H NMR (400 MHz, $CDCl_3$) of **5ap**

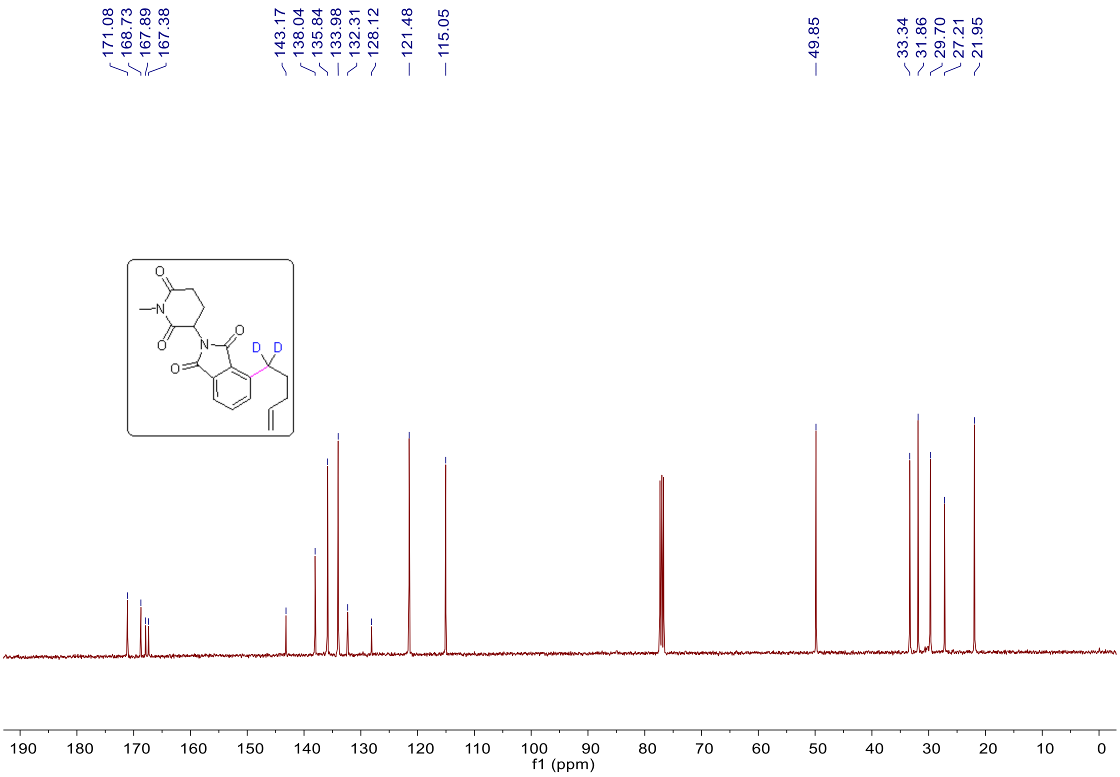

Supplementary Figure 277. ^{13}C NMR (101 MHz, $CDCl_3$) of **5ap**

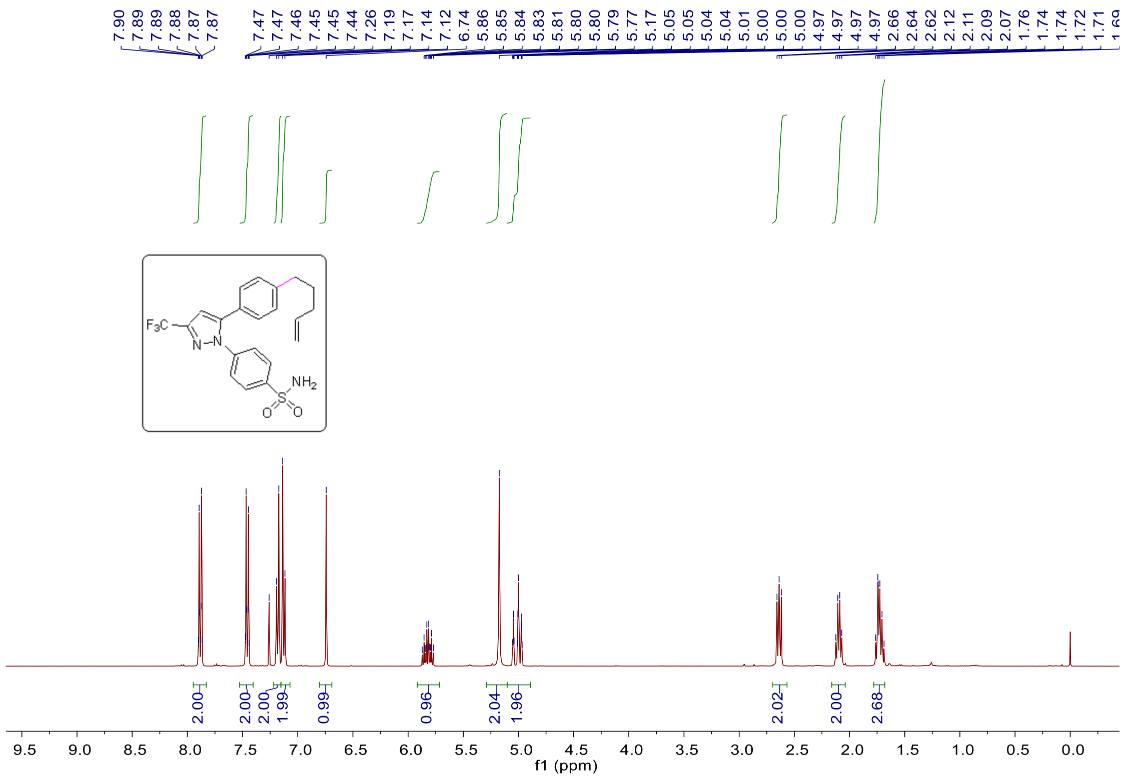

Supplementary Figure 278. ^{1}H NMR (400 MHz, $CDCl_3$) of **5aq'**

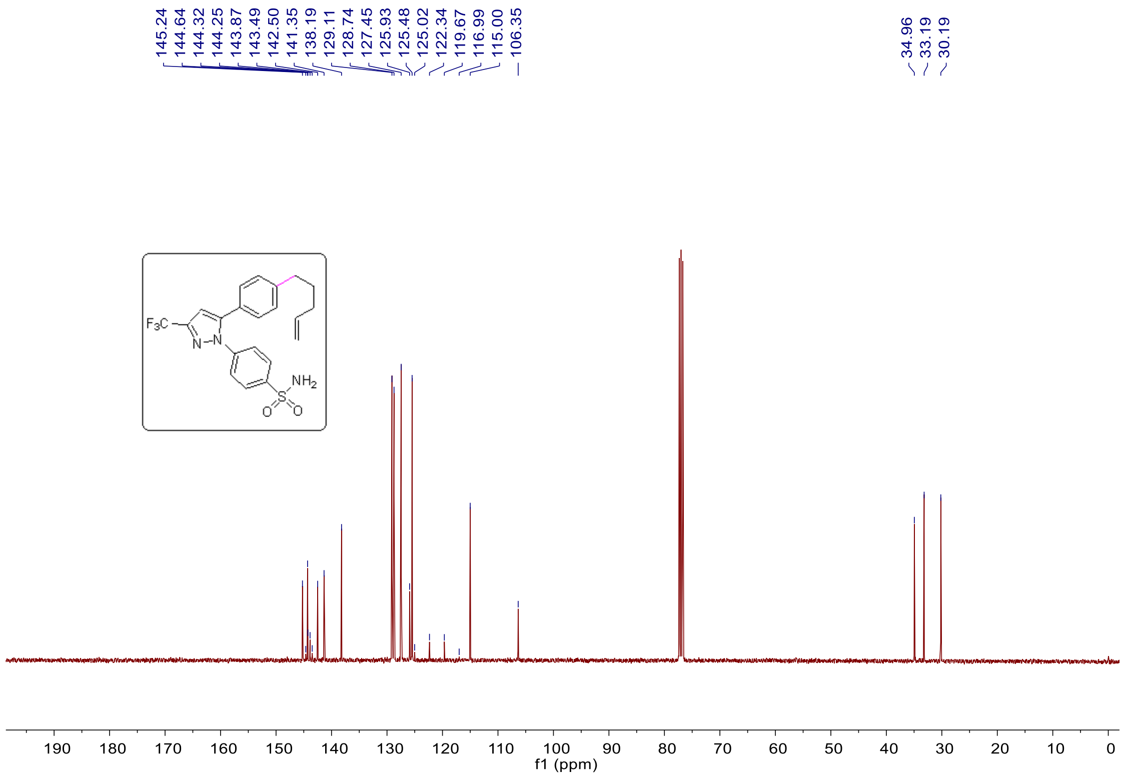

Supplementary Figure 279. ^{13}C NMR (101 MHz, $CDCl_3$) of **5aq'**

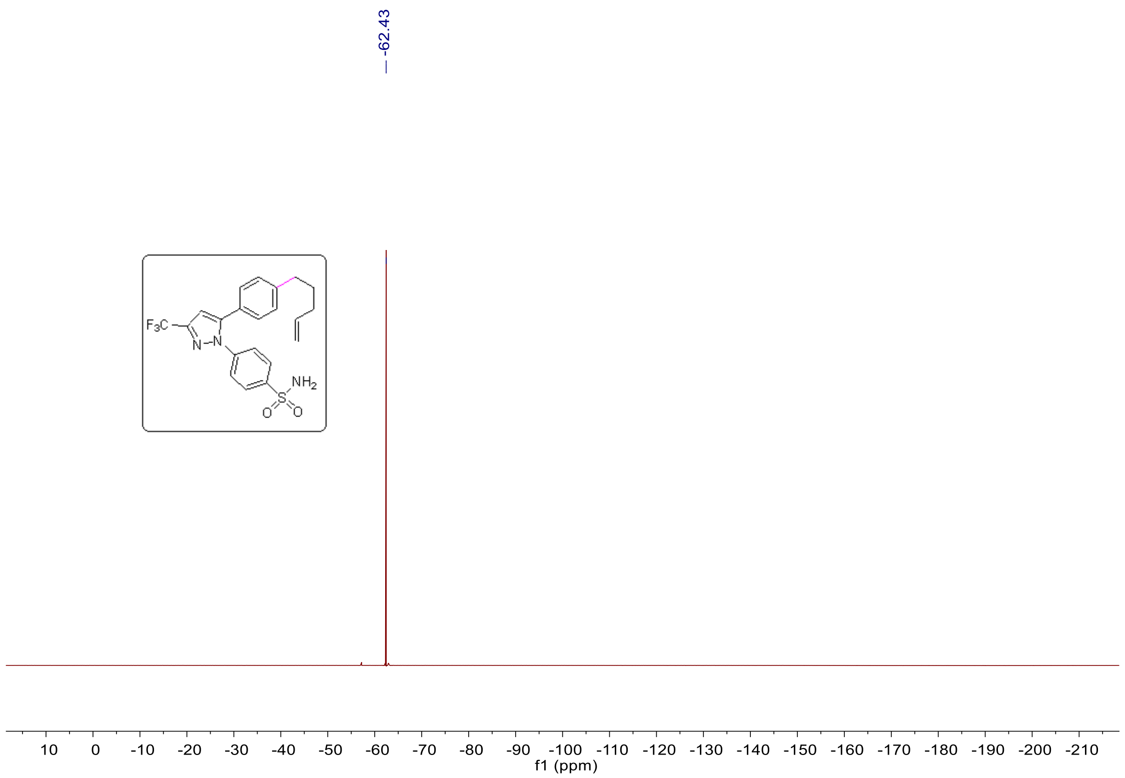

Supplementary Figure 280. ^{19}F NMR (376 MHz, $CDCl_3$) of **5aq'**

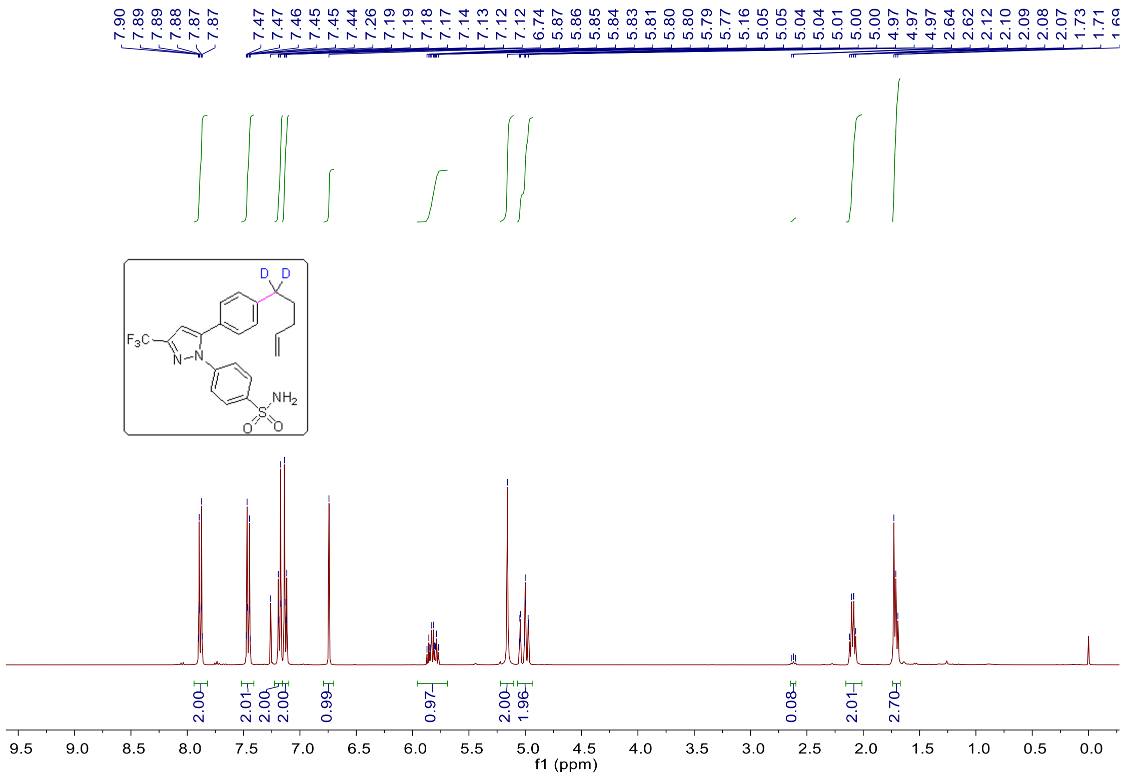

Supplementary Figure 281. ^{1}H NMR (400 MHz, $CDCl_3$) of **5aq**

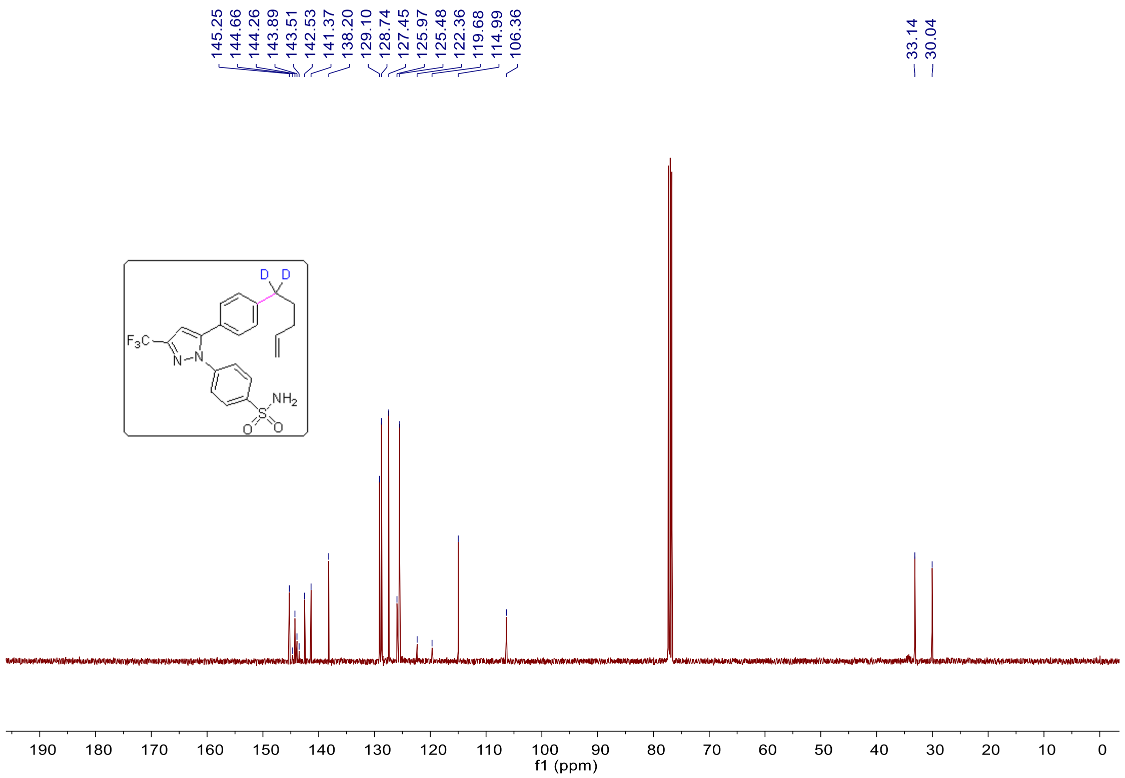

Supplementary Figure 282. ^{13}C NMR (101 MHz, $CDCl_3$) of **5aq**

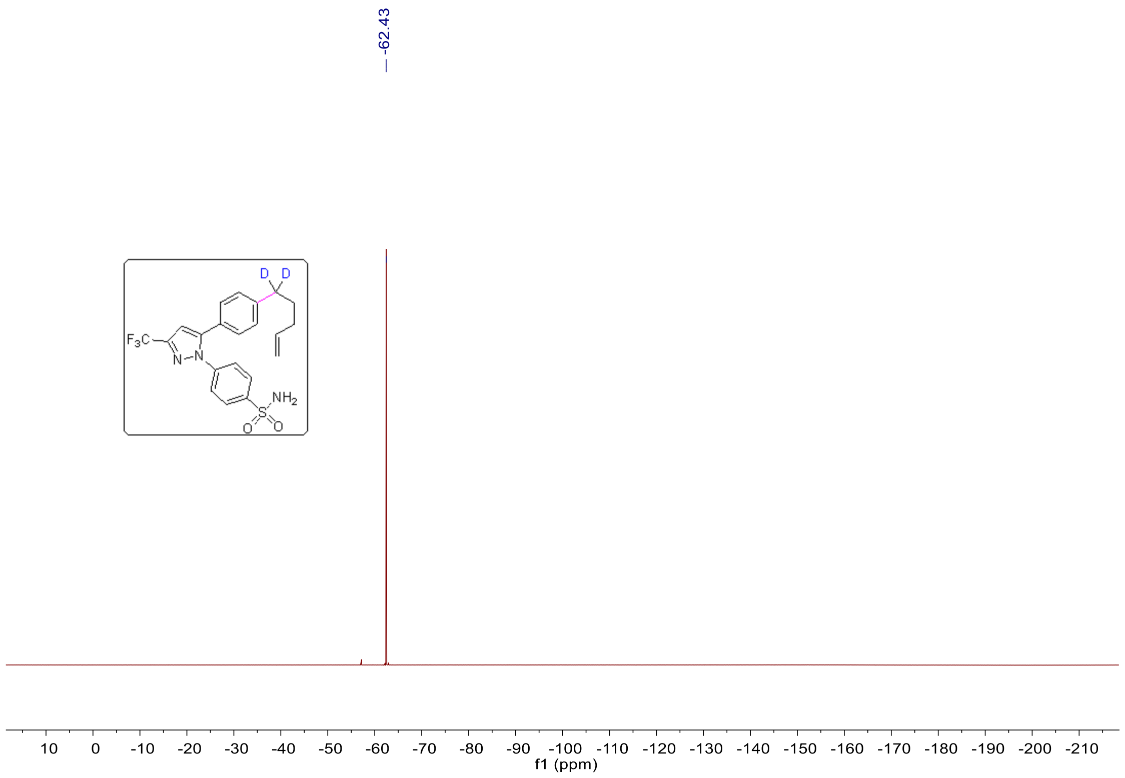

Supplementary Figure 283. ^{19}F NMR (376 MHz, $CDCl_3$) of **5aq**

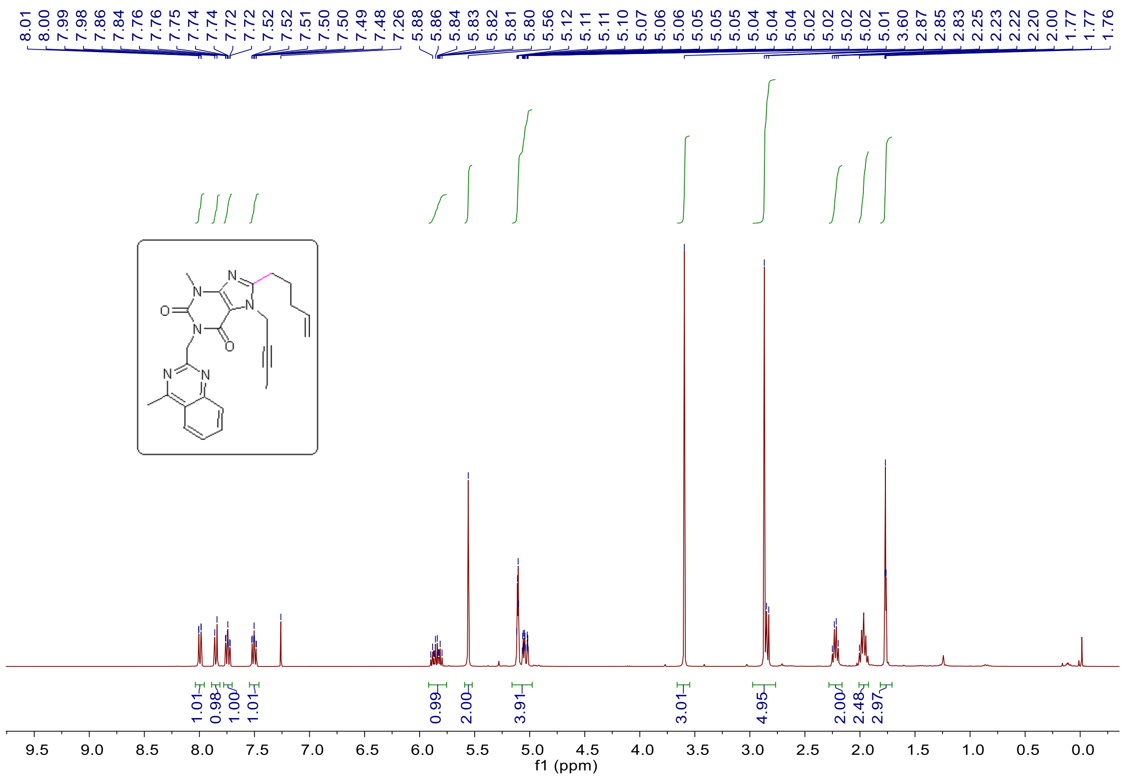

Supplementary Figure 284. ^{1}H NMR (400 MHz, $CDCl_3$) of **5ar'**

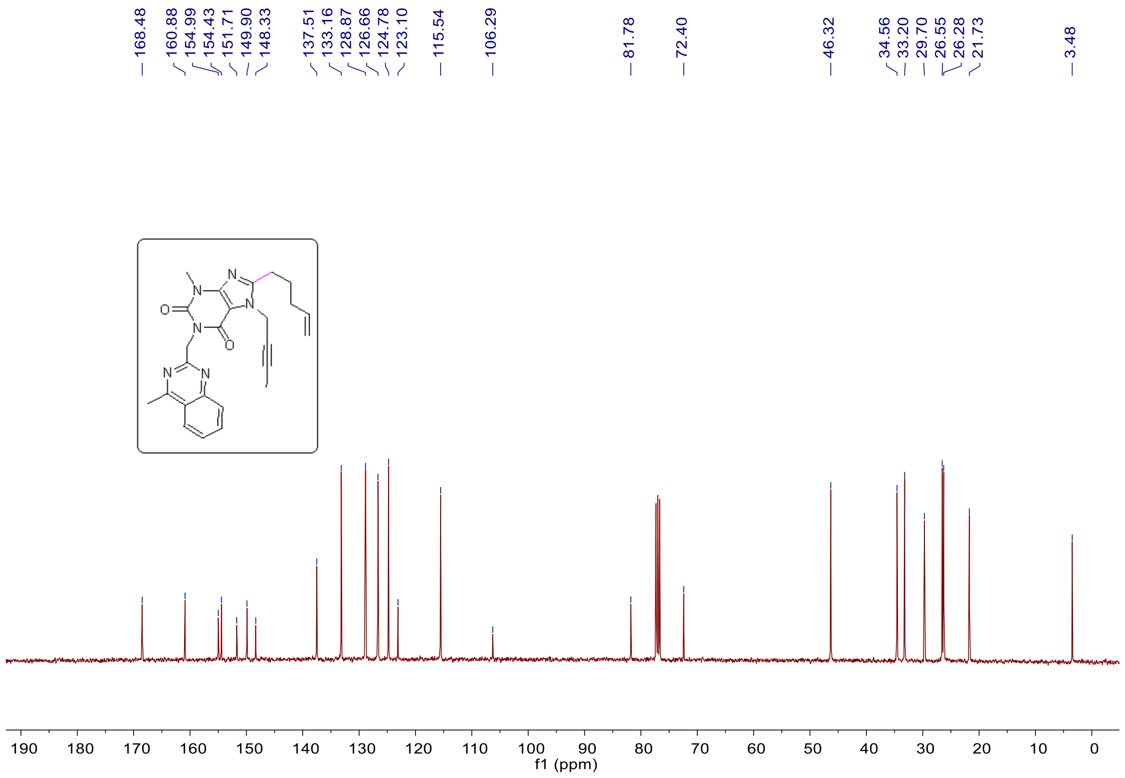

Supplementary Figure 285. ^{13}C NMR (101 MHz, $CDCl_3$) of **5ar'**

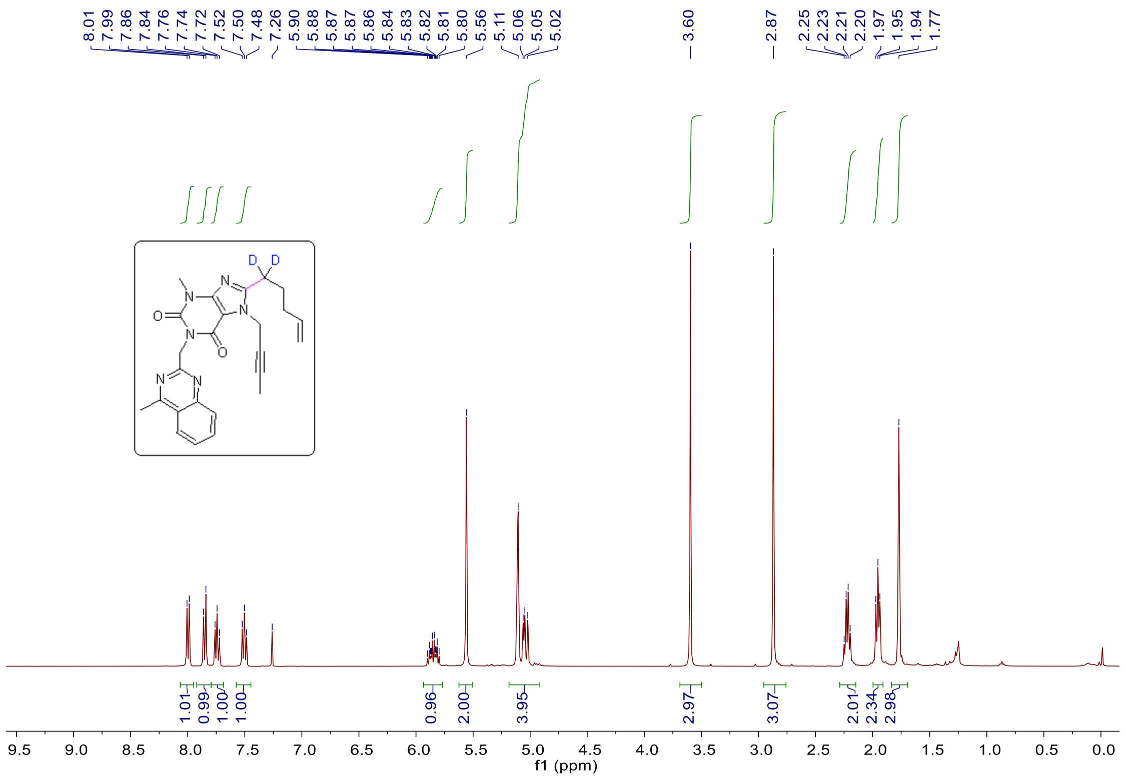

Supplementary Figure 286. ^{1}H NMR (400 MHz, $CDCl_3$) of **5ar**

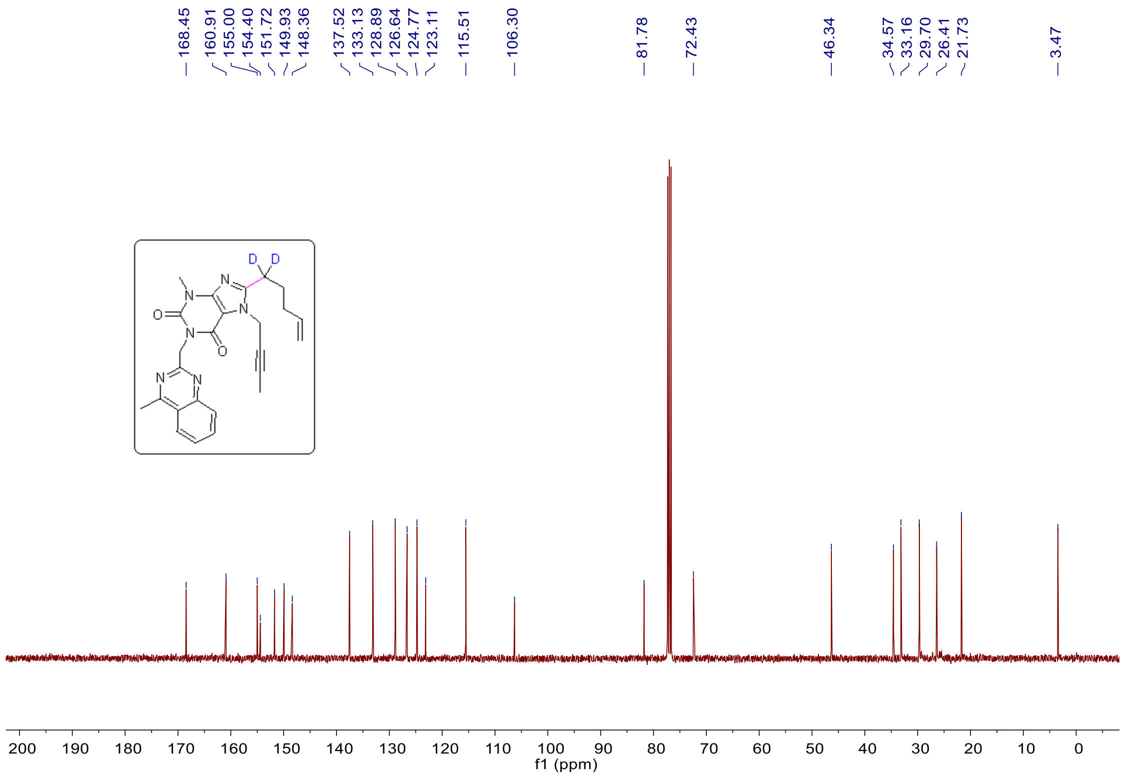

Supplementary Figure 287. ^{13}C NMR (101 MHz, $CDCl_3$) of **5ar**

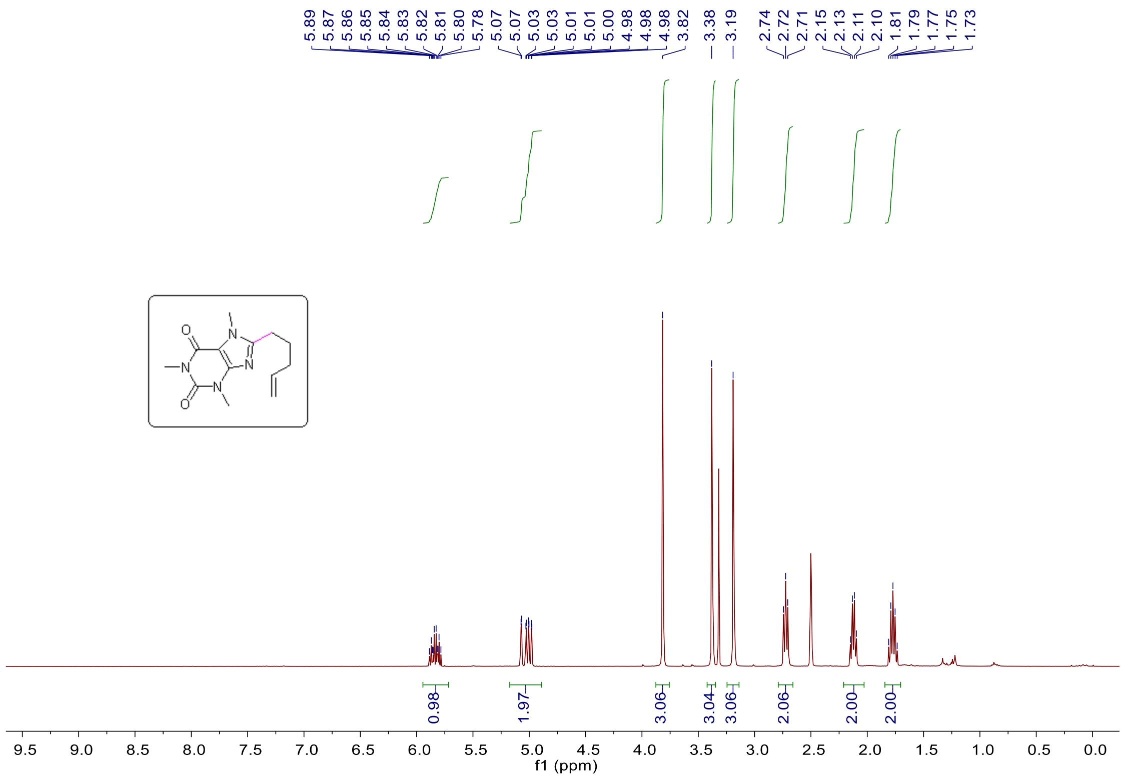

Supplementary Figure 288. ^{1}H NMR (400 MHz, DMSO-d_6) of **5as'**

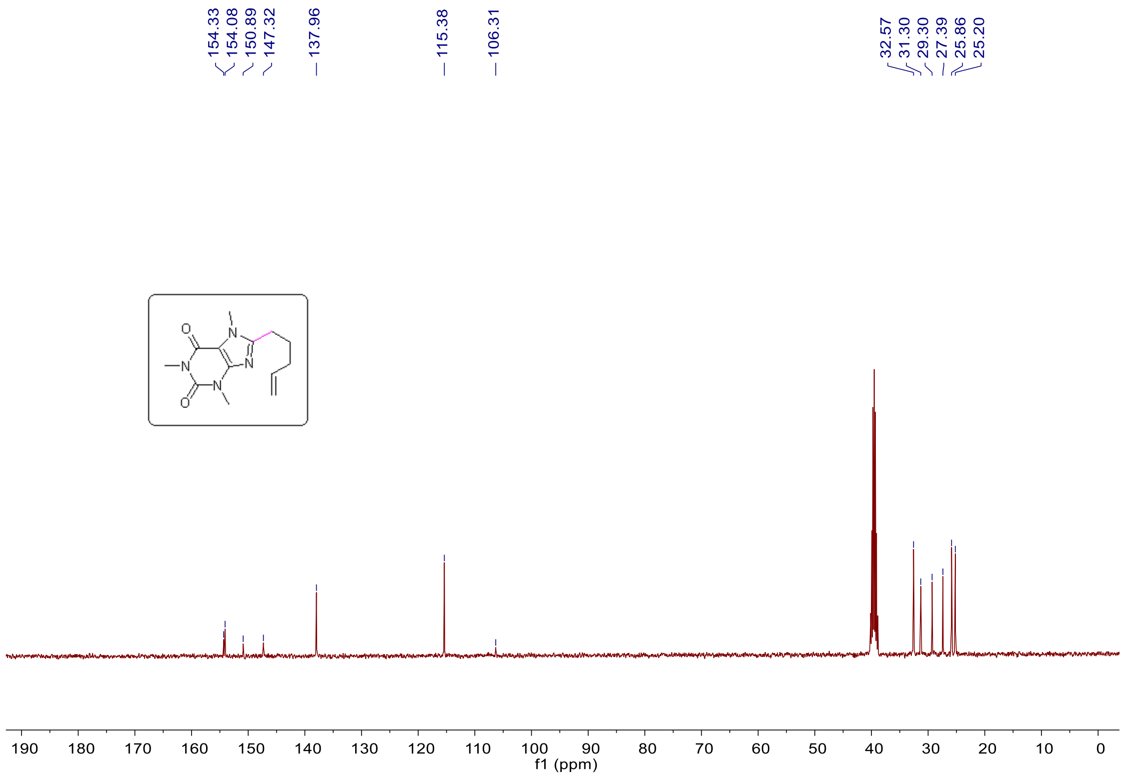

Supplementary Figure 289. ^{13}C NMR (101 MHz, DMSO-d_6) of **5as'**

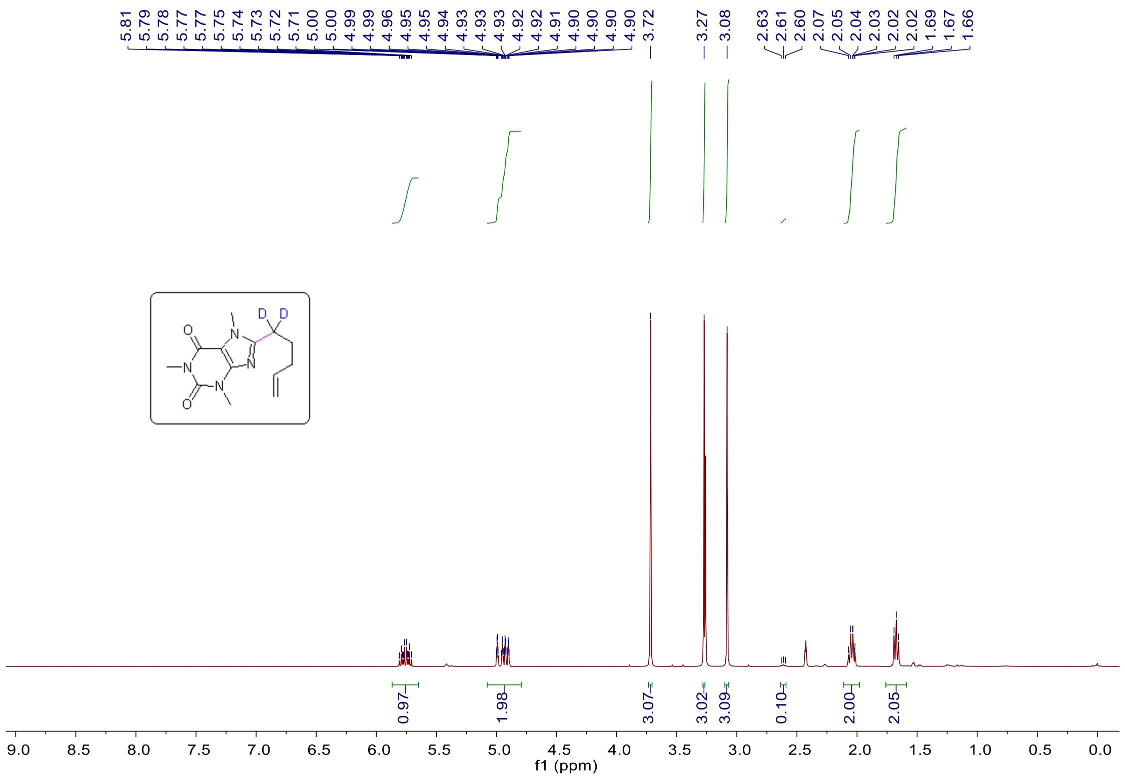

Supplementary Figure 290. ^{1}H NMR (400 MHz, DMSO-d_6) of **5as**

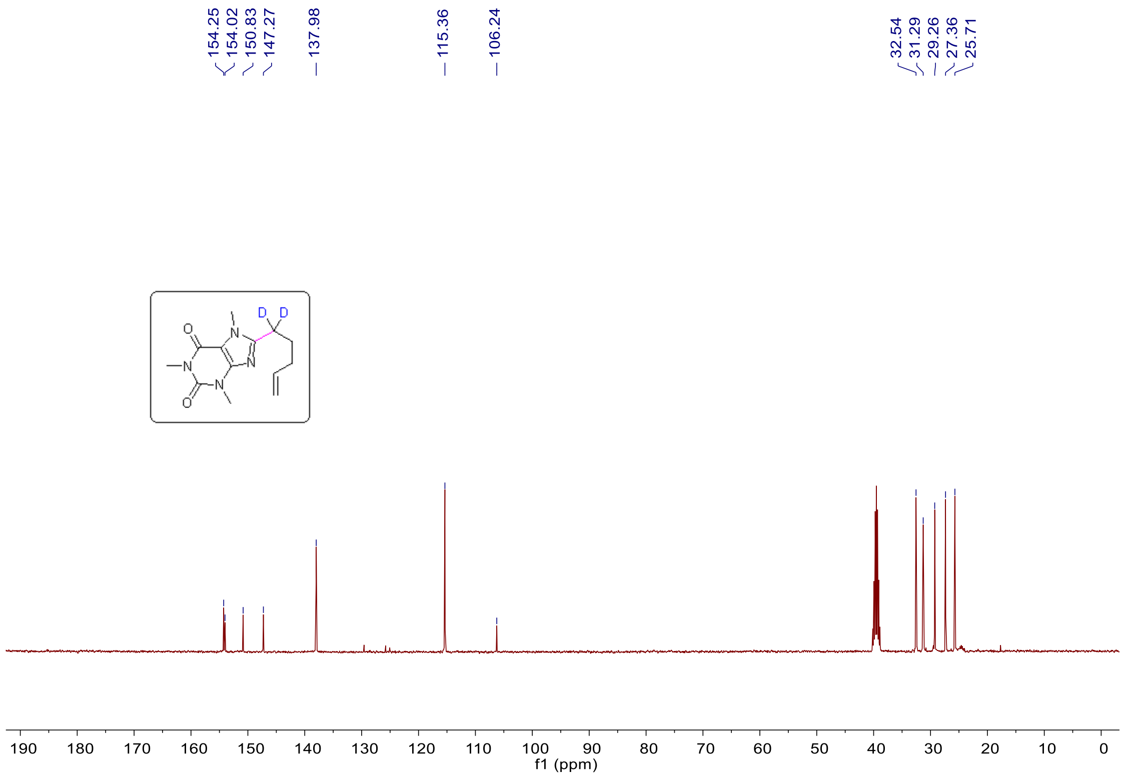

Supplementary Figure 291. ^{13}C NMR (101 MHz, DMSO-d_6) of **5as**

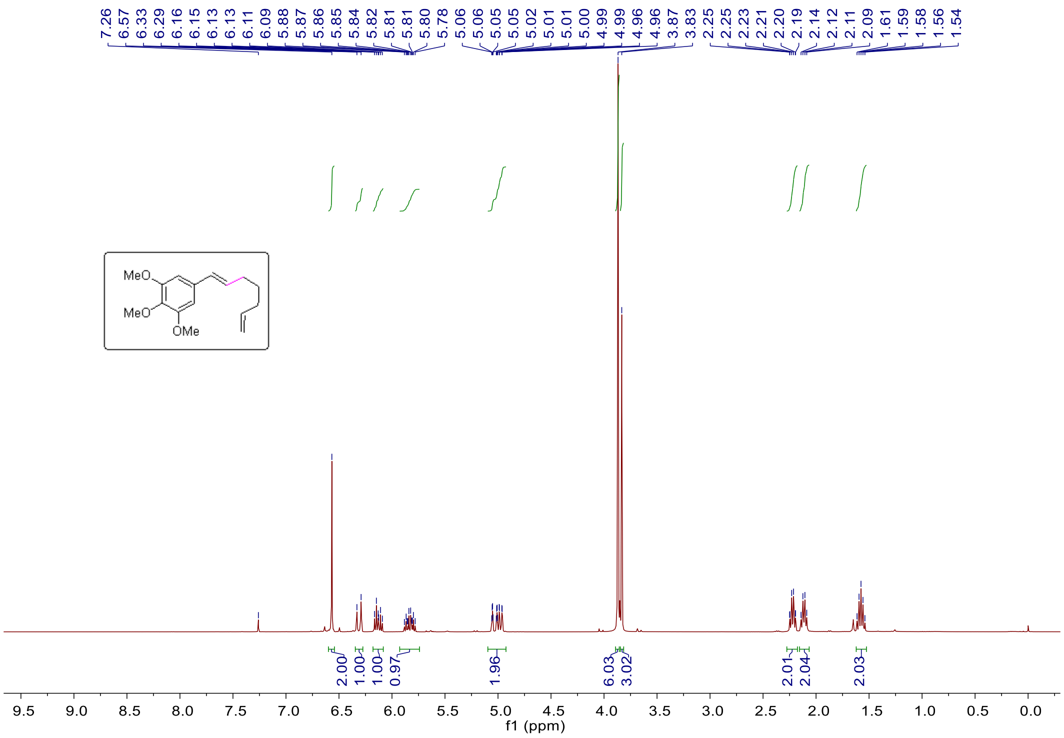

Supplementary Figure 292. ^{1}H NMR (400 MHz, $CDCl_3$) of **5at'**

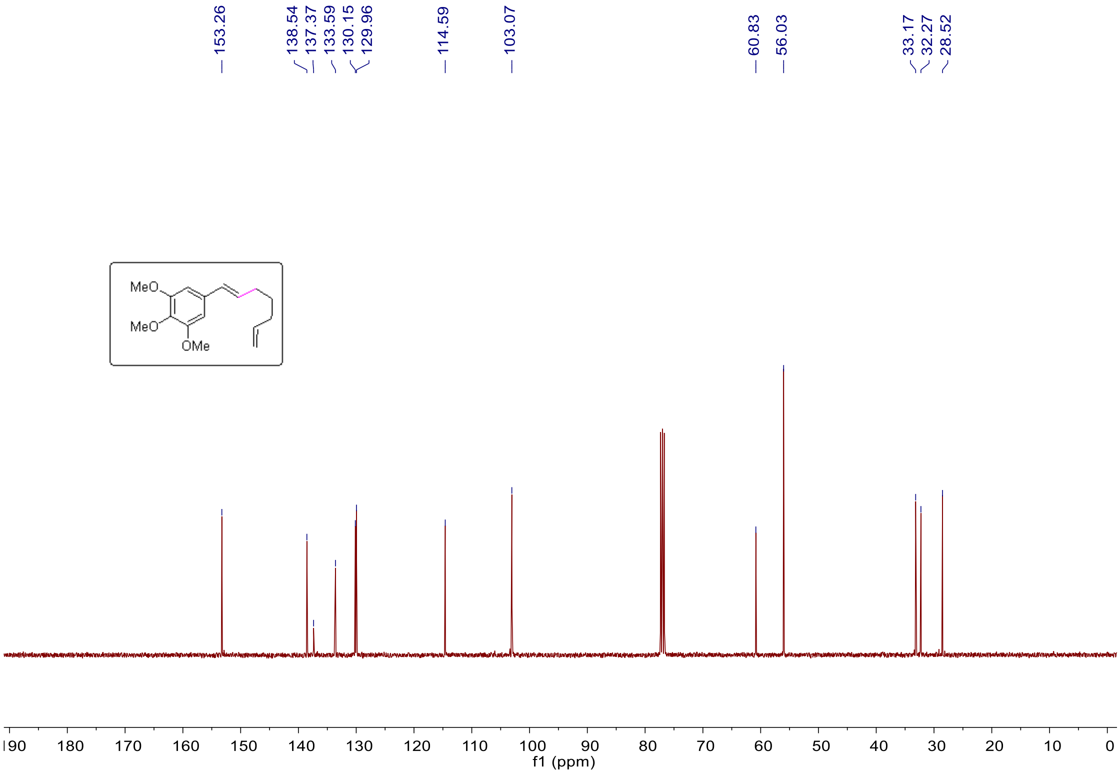

Supplementary Figure 293. ^{13}C NMR (101 MHz, $CDCl_3$) of **5at'**

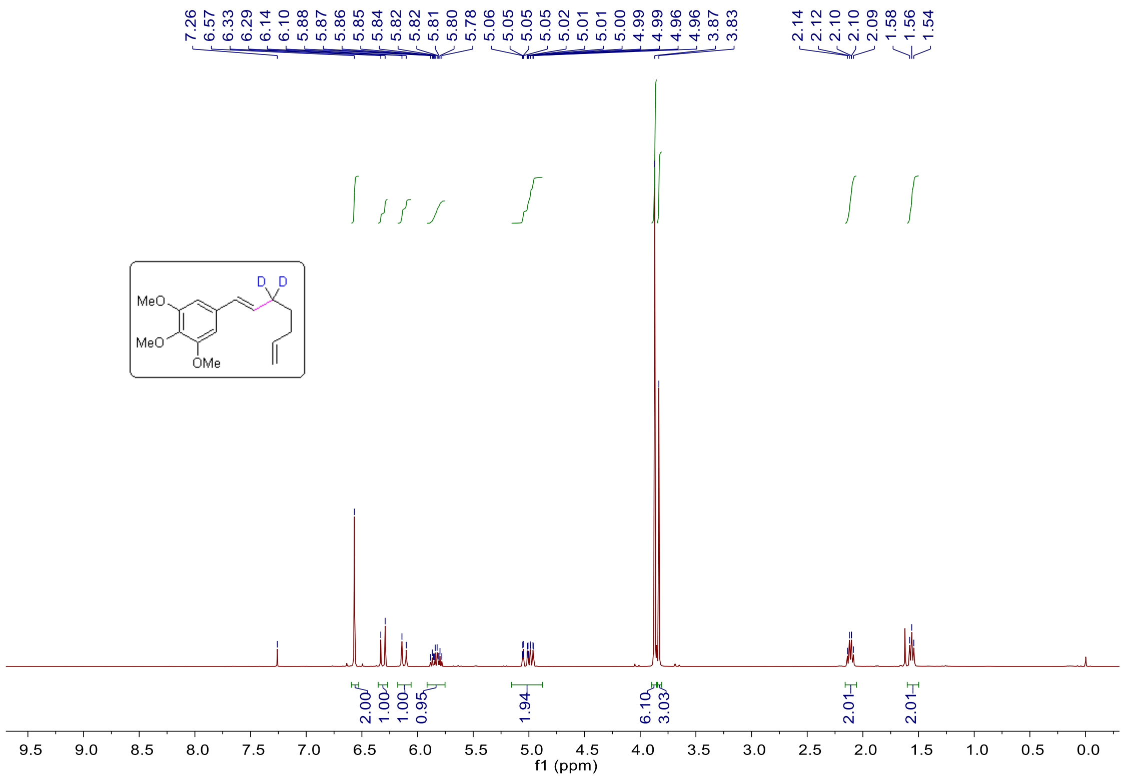

Supplementary Figure 294. ^{1}H NMR (400 MHz, $CDCl_3$) of **5at**

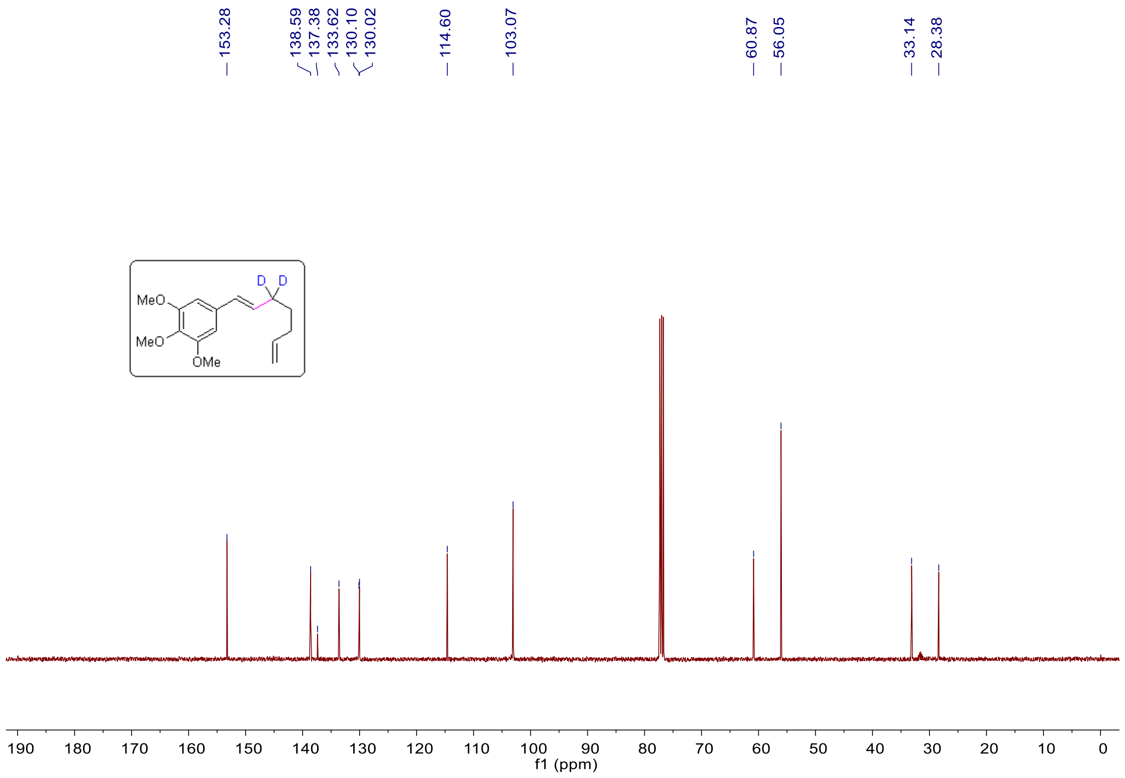

Supplementary Figure 295. ^{13}C NMR (101 MHz, $CDCl_3$) of **5at**

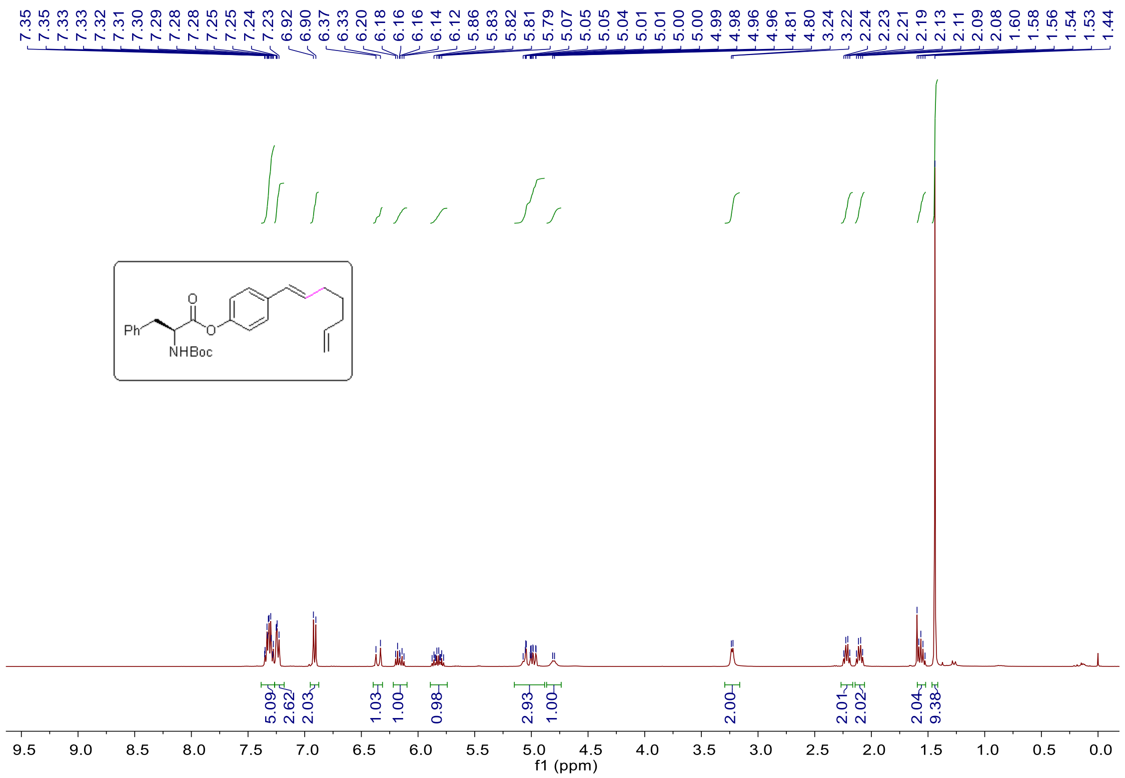

Supplementary Figure 296. ^{1}H NMR (400 MHz, $CDCl_3$) of **5au'**

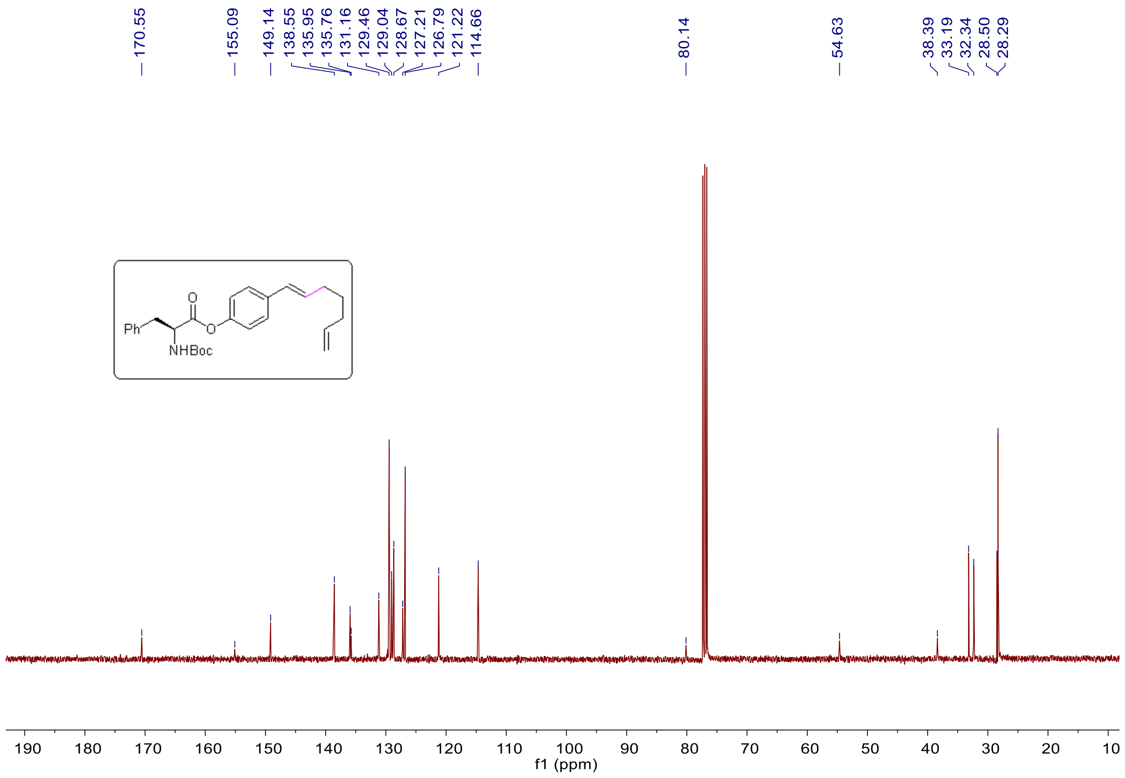

Supplementary Figure 297. ^{13}C NMR (101 MHz, $CDCl_3$) of **5au'**

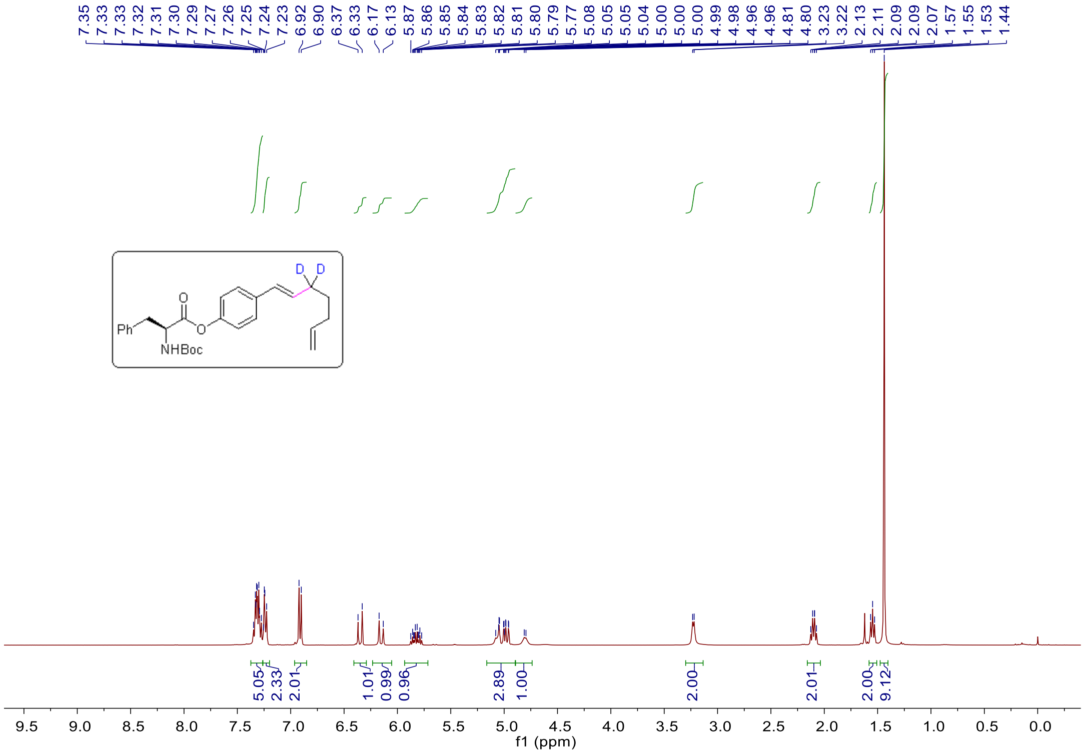

Supplementary Figure 298. ^{1}H NMR (400 MHz, $CDCl_3$) of **5au**

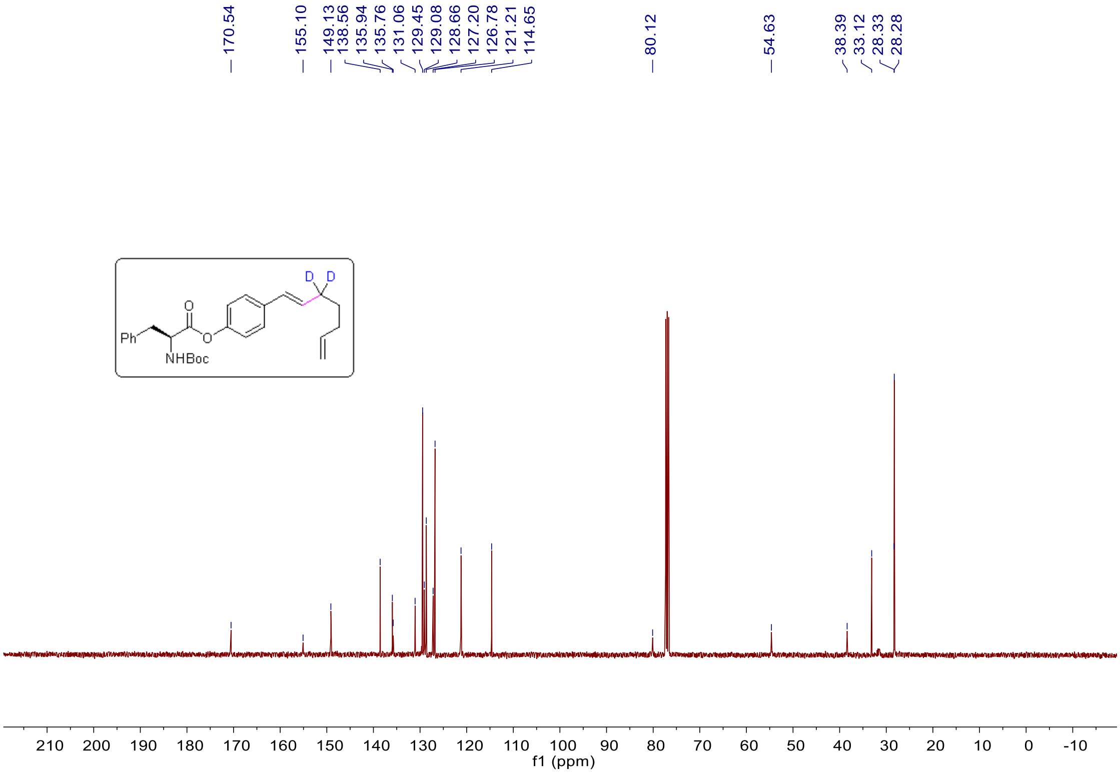

Supplementary Figure 299. ^{13}C NMR (101 MHz, $CDCl_3$) of **5au**

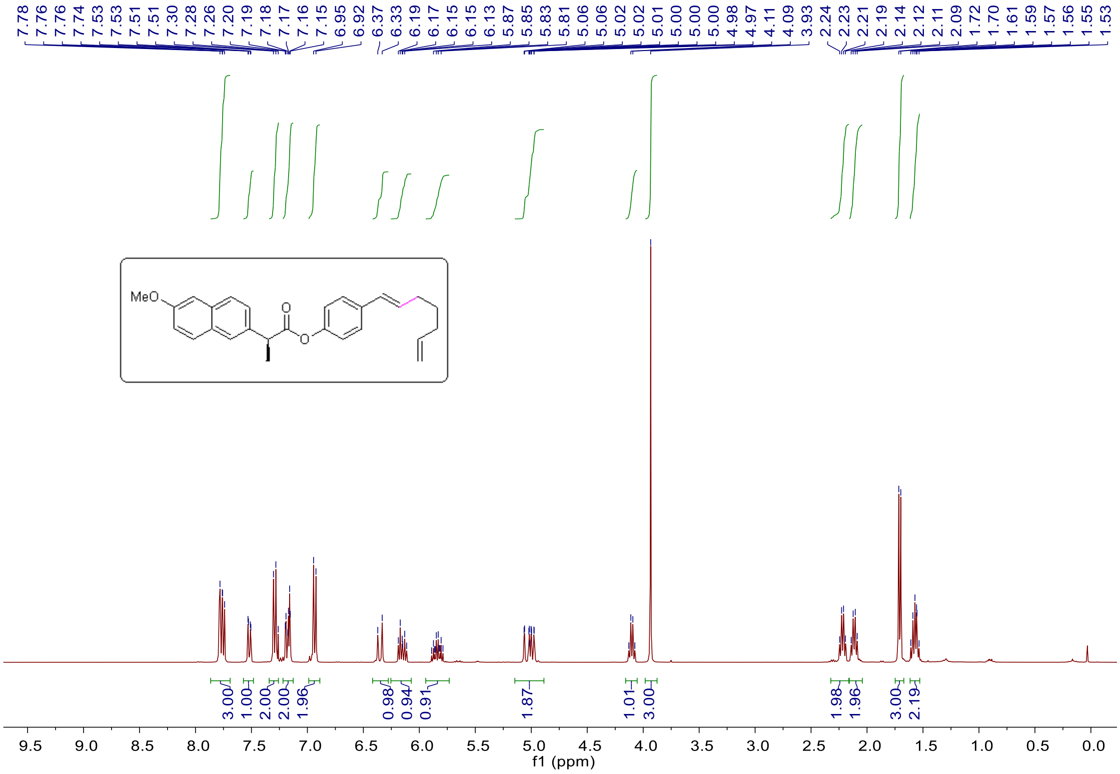

Supplementary Figure 300. ^{1}H NMR (400 MHz, $CDCl_3$) of **5av'**

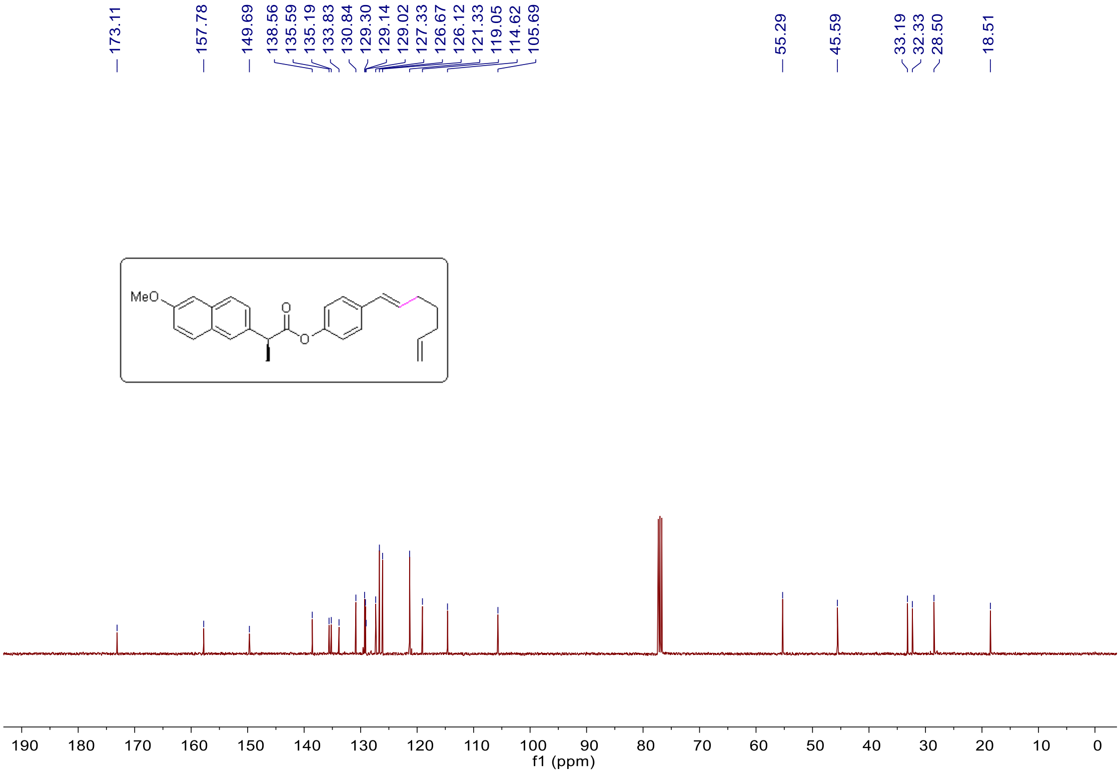

Supplementary Figure 301. ^{13}C NMR (101 MHz, $CDCl_3$) of **5av'**

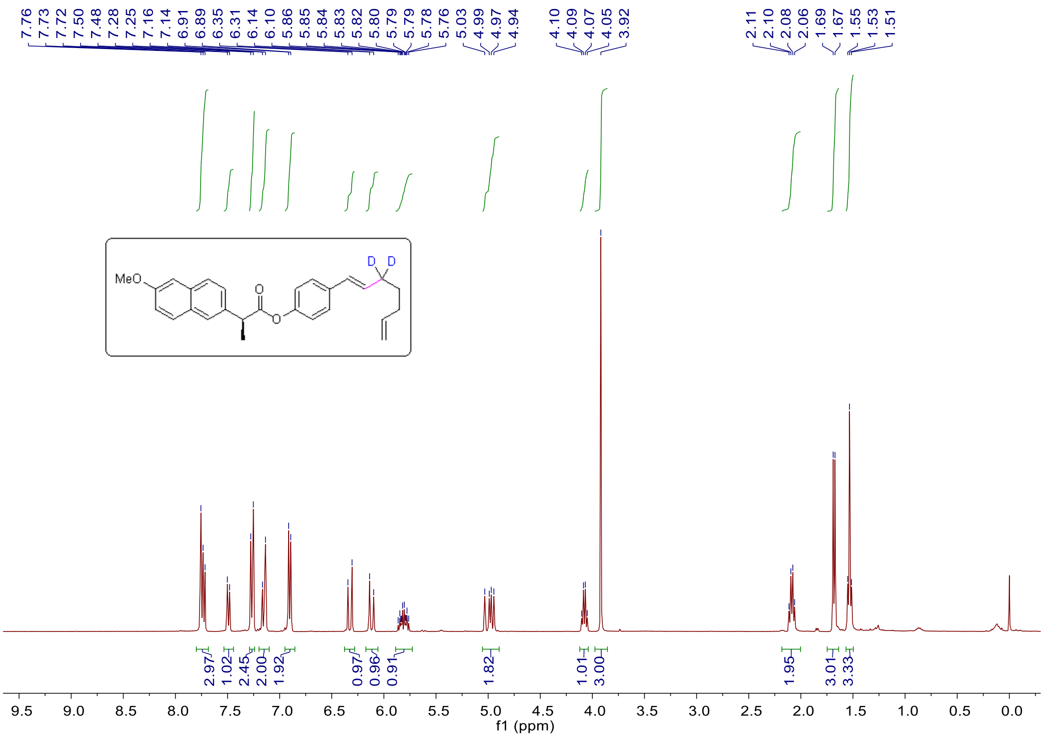

Supplementary Figure 302. ^{1}H NMR (400 MHz, $CDCl_3$) of **5av**

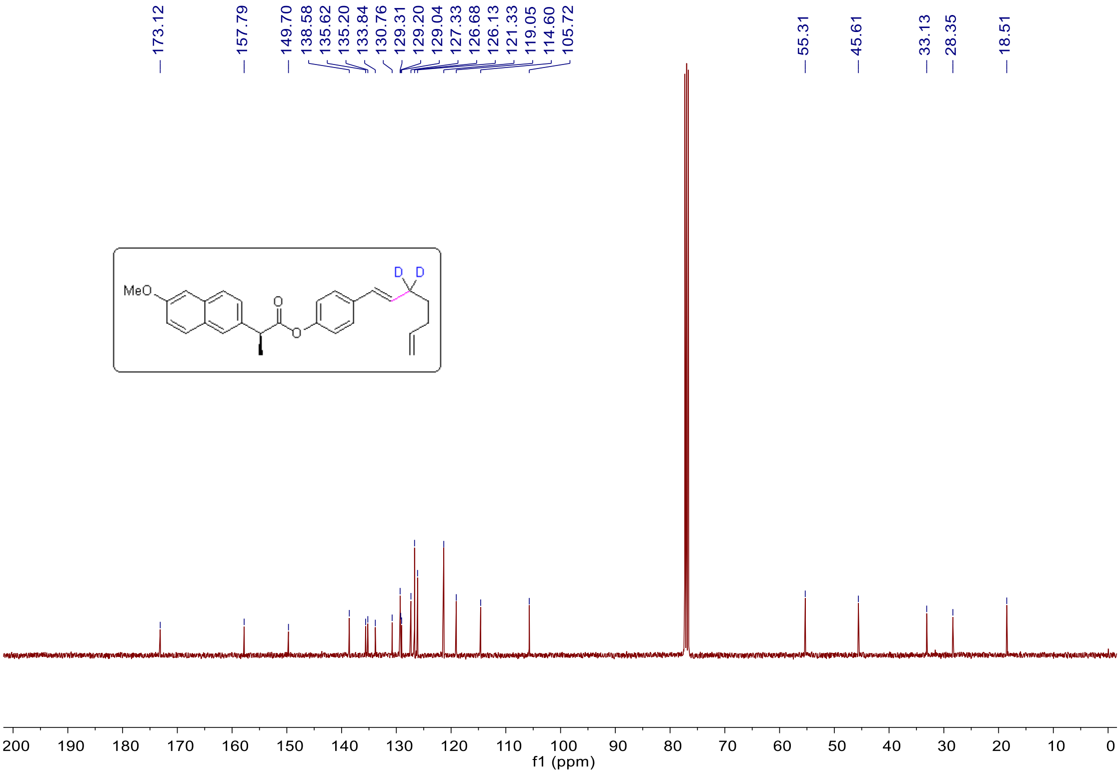

Supplementary Figure 303. ^{13}C NMR (101 MHz, $CDCl_3$) of **5av**

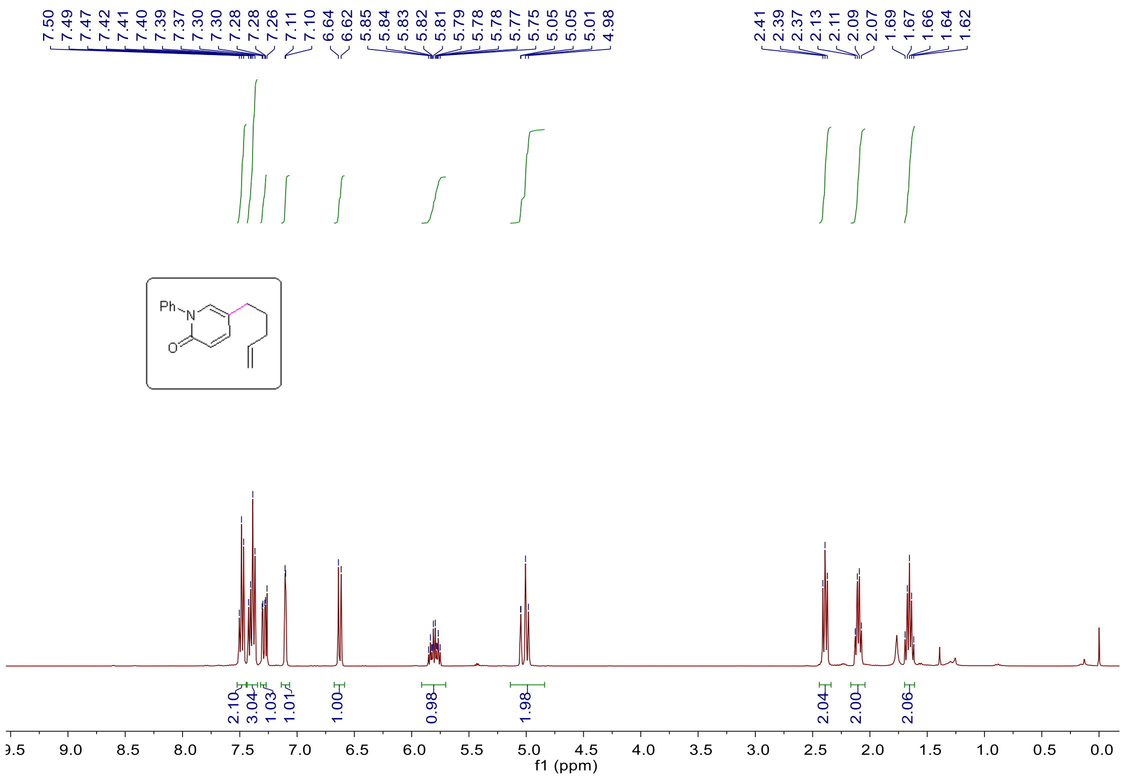

Supplementary Figure 304. ^{1}H NMR (400 MHz, $CDCl_3$) of **5aw'**

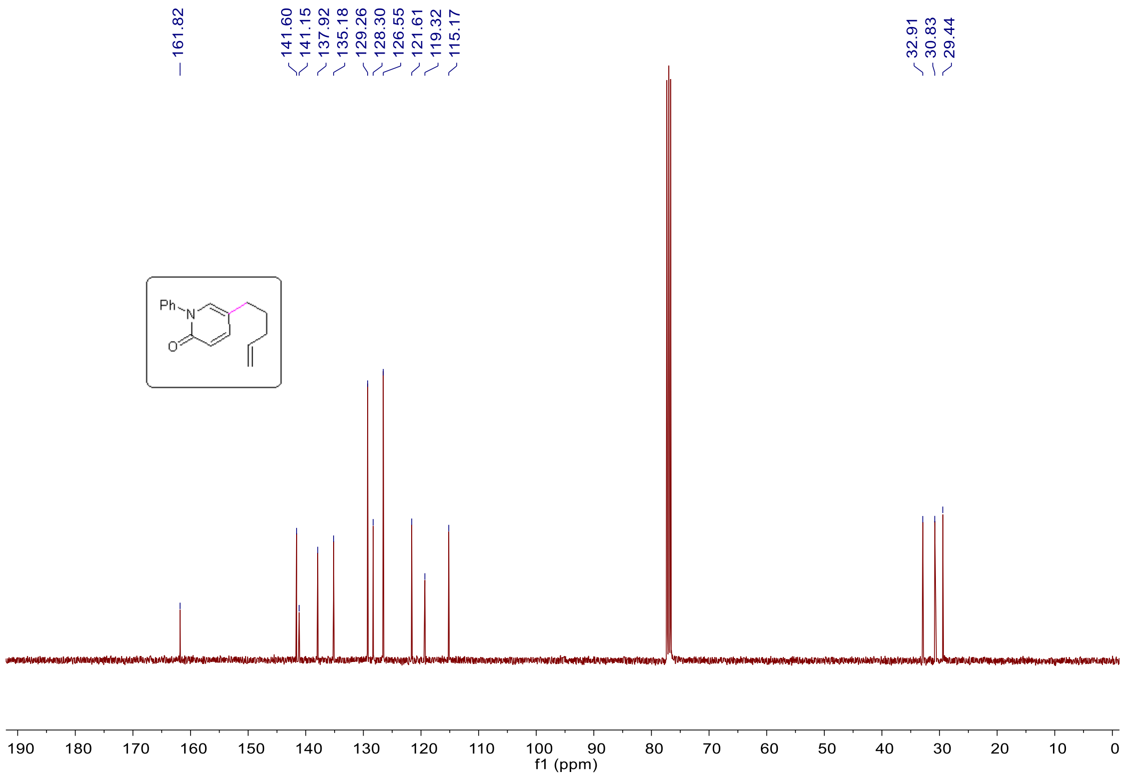

Supplementary Figure 305. ^{13}C NMR (101 MHz, $CDCl_3$) of **5aw'**

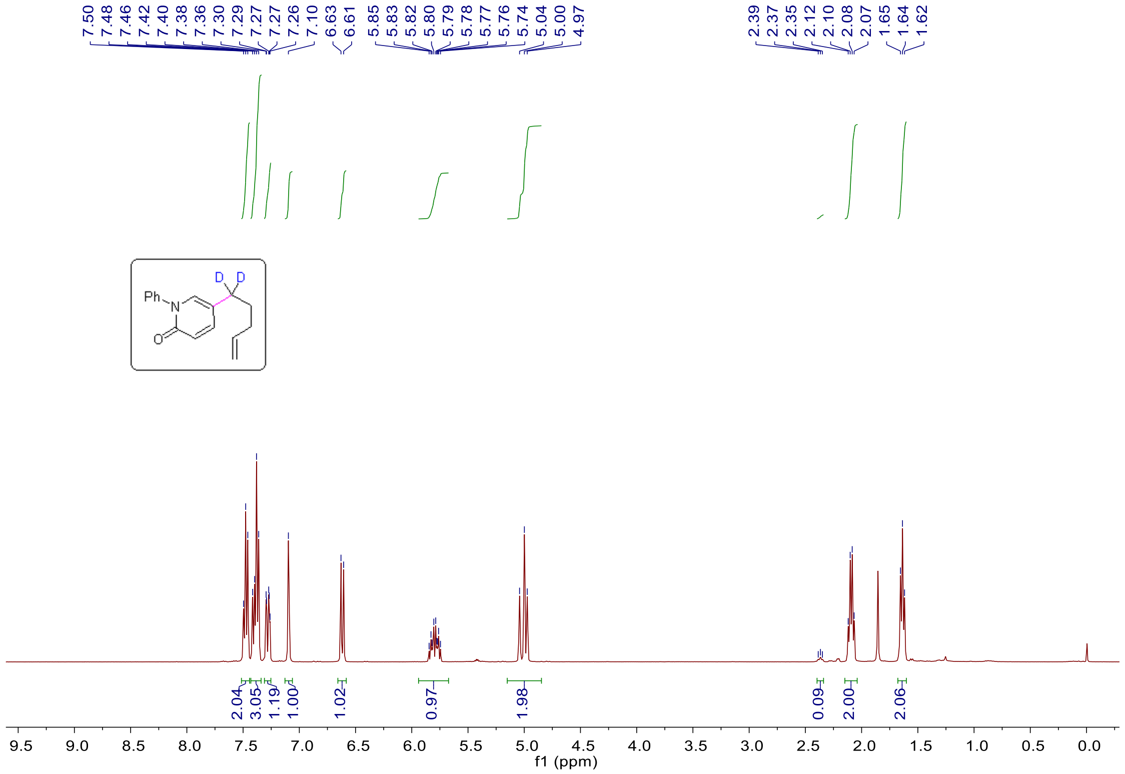

Supplementary Figure 306. ^{1}H NMR (400 MHz, $CDCl_3$) of **5aw**

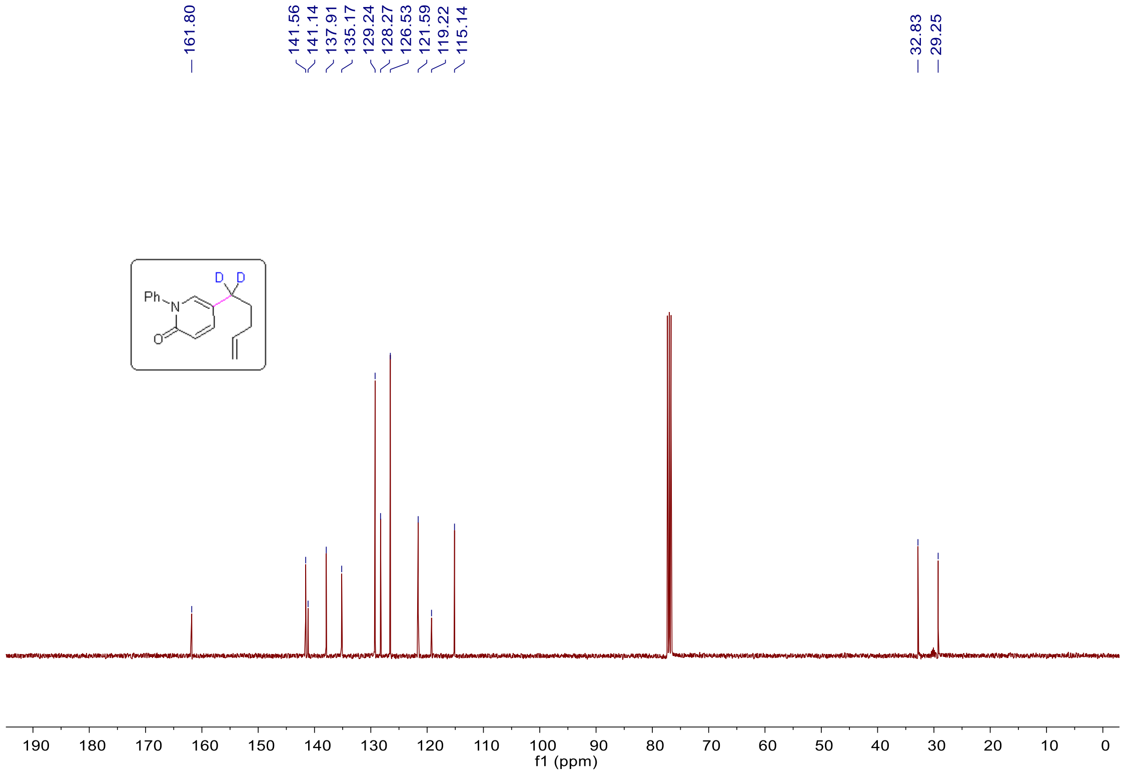

Supplementary Figure 307. ^{13}C NMR (101 MHz, $CDCl_3$) of **5aw**

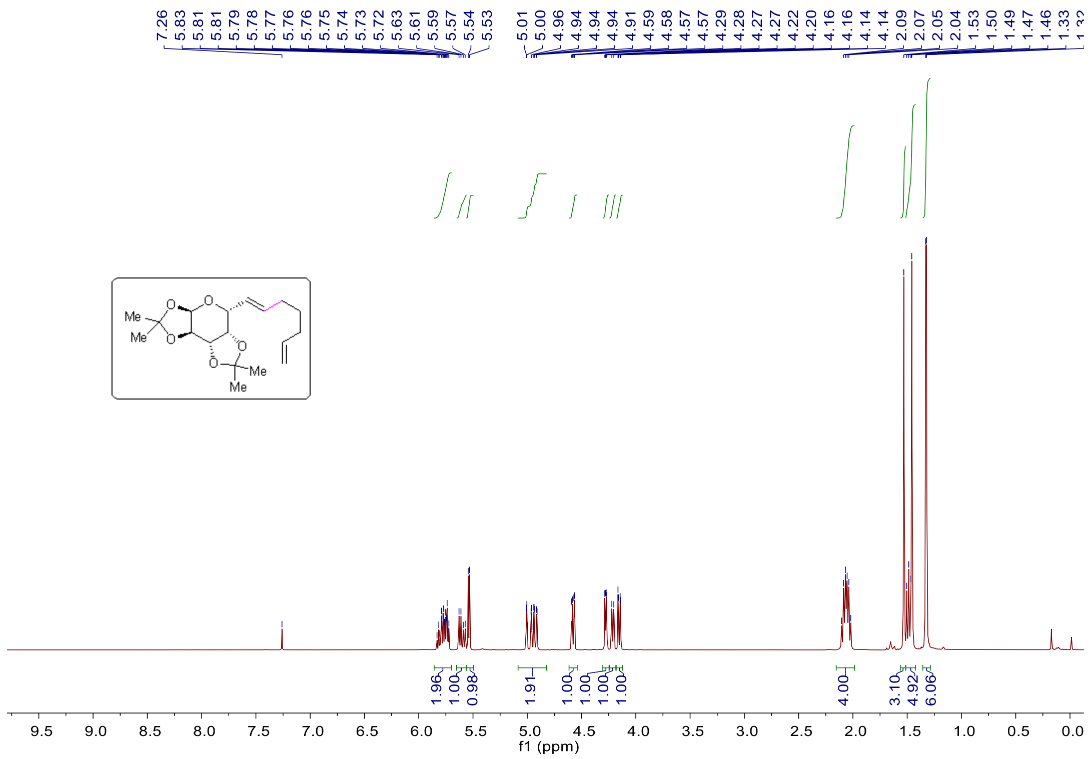

Supplementary Figure 308. ^{1}H NMR (400 MHz, $CDCl_3$) of **5ax'**

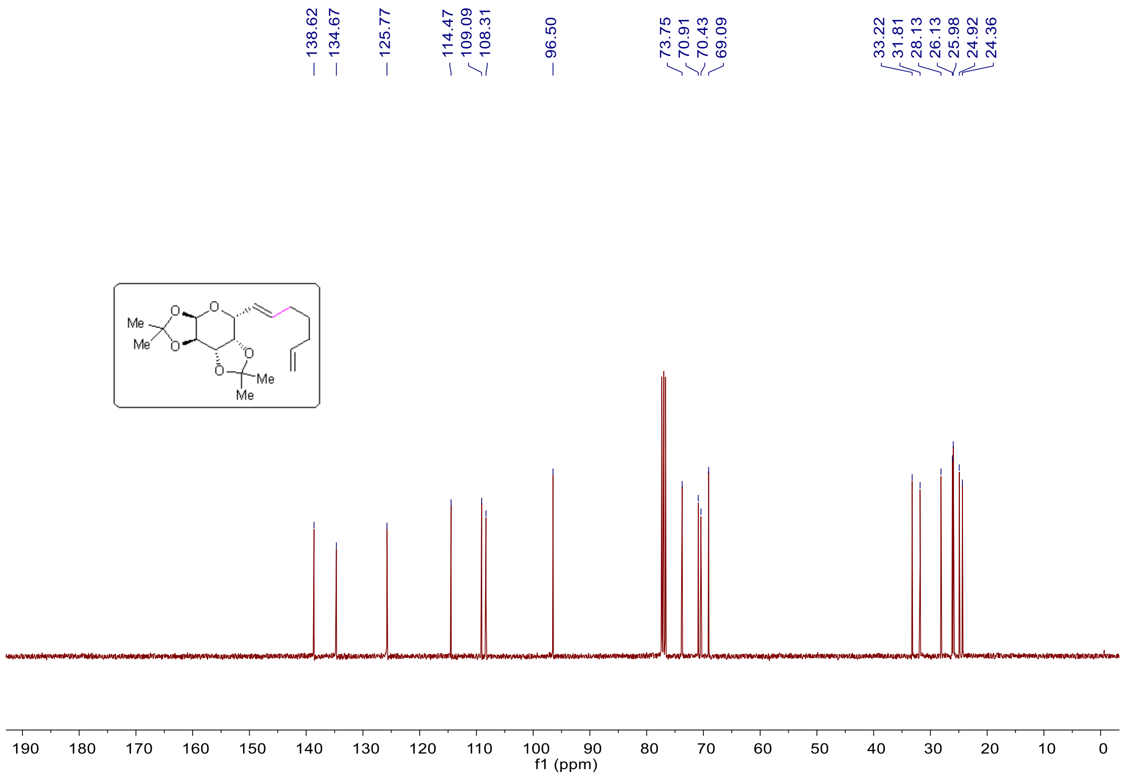

Supplementary Figure 309. ^{13}C NMR (101 MHz, $CDCl_3$) of **5ax'**

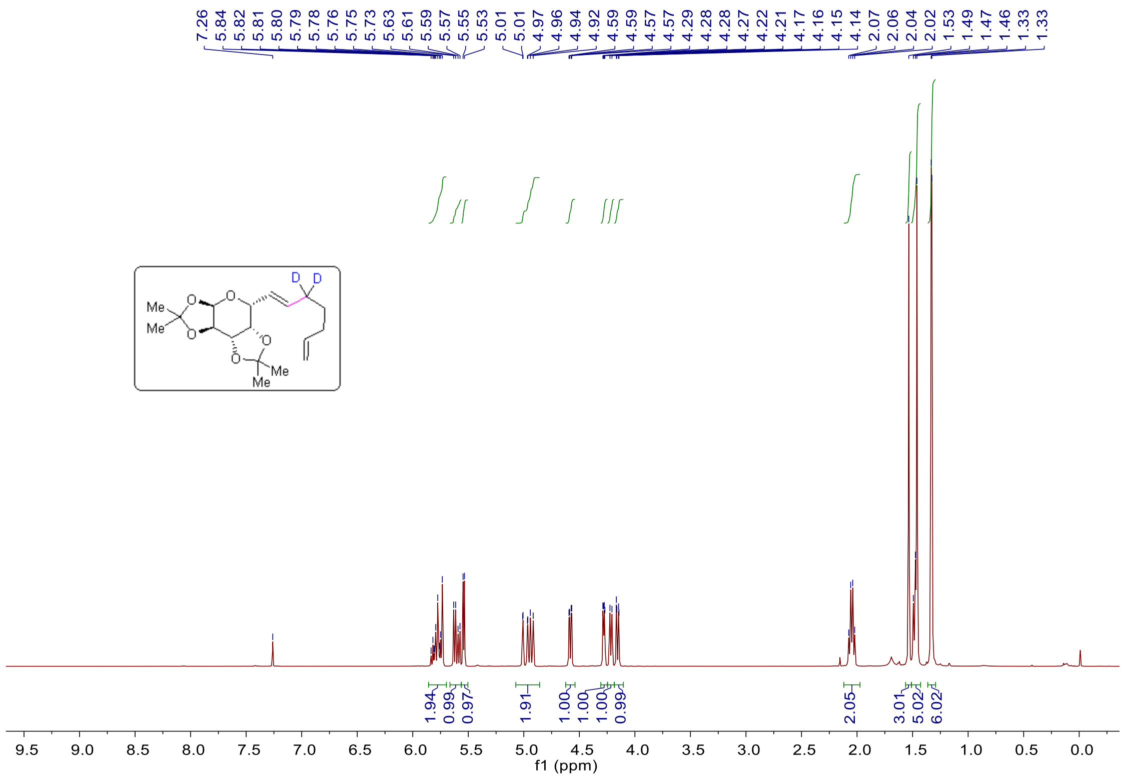

Supplementary Figure 310. ^{1}H NMR (400 MHz, $CDCl_3$) of **5ax**

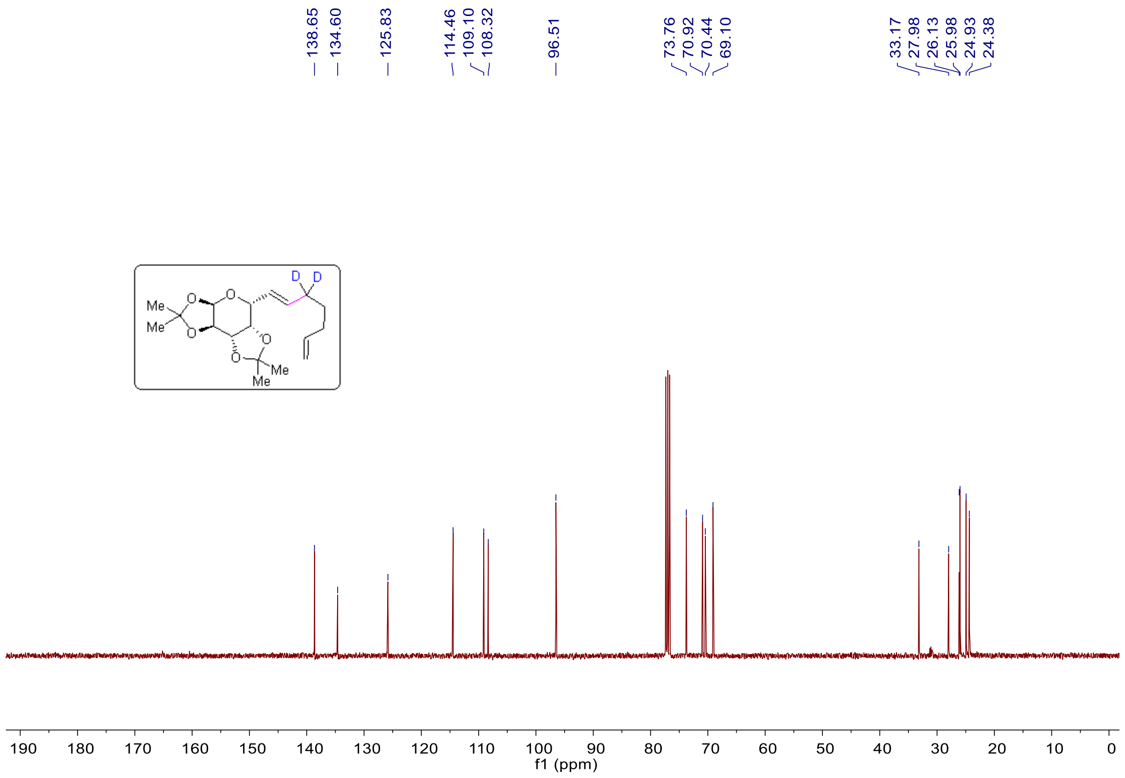

Supplementary Figure 311. ^{13}C NMR (101 MHz, $CDCl_3$) of **5ax**

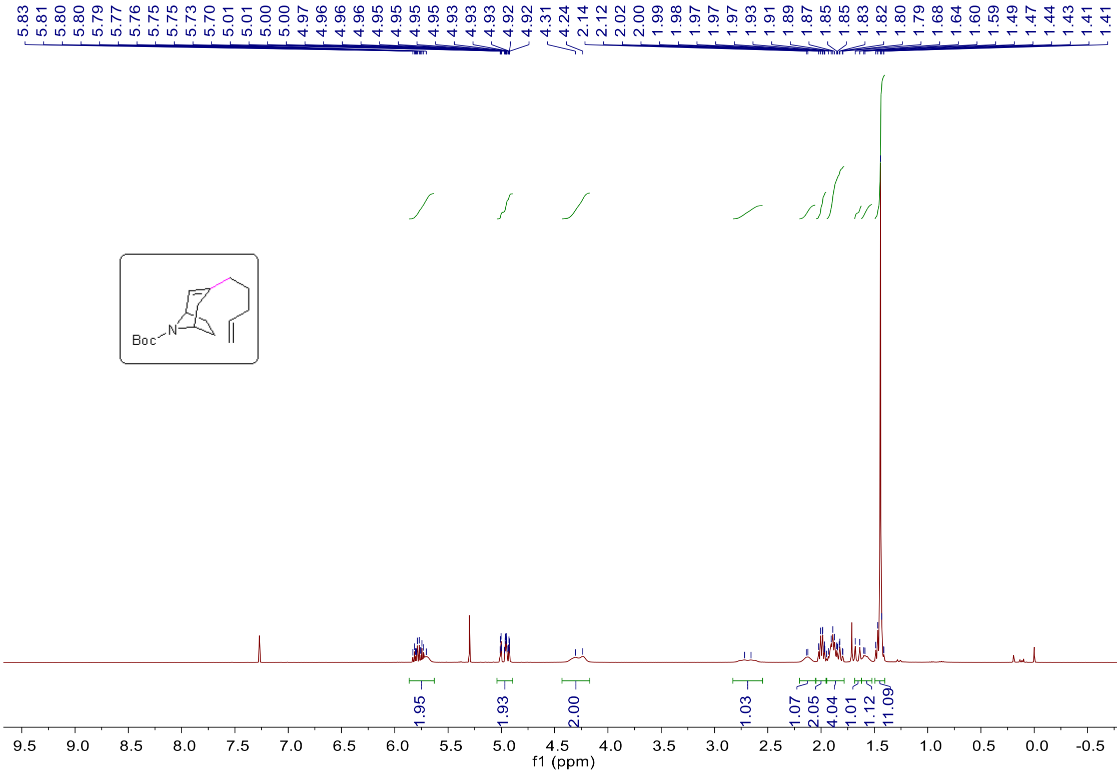

Supplementary Figure 312. ^{1}H NMR (400 MHz, $CDCl_3$) of **5ay'**

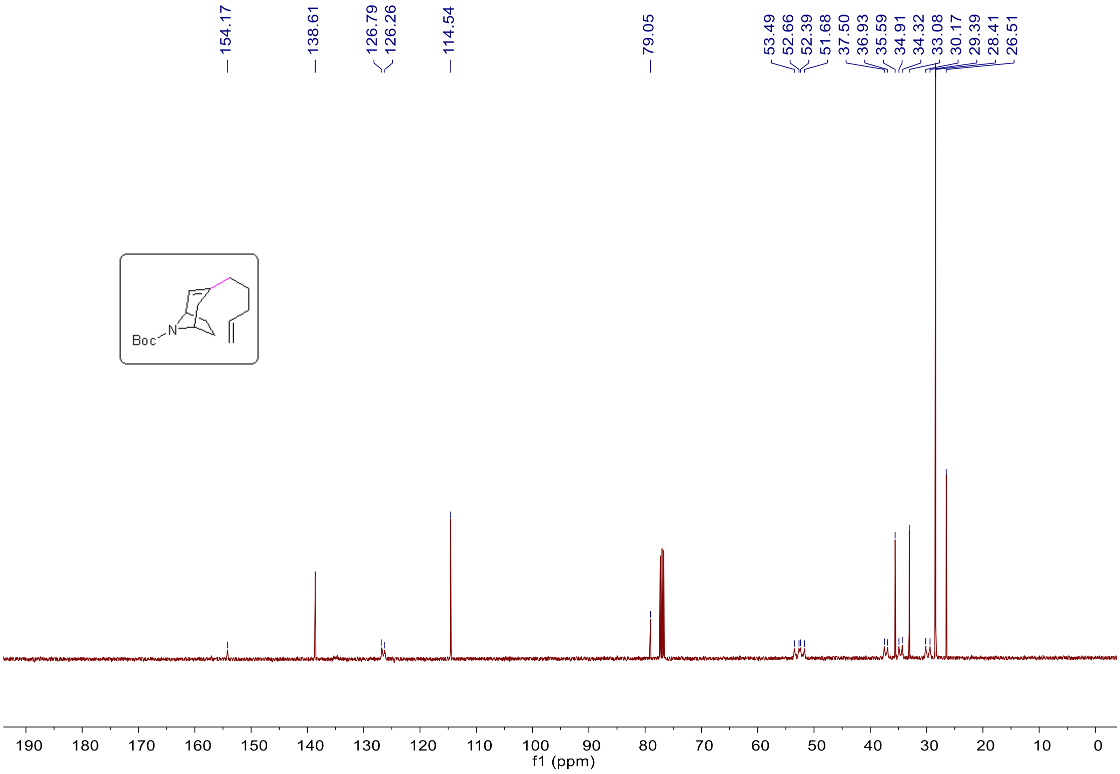

Supplementary Figure 313. ^{13}C NMR (101 MHz, $CDCl_3$) of **5ay'**

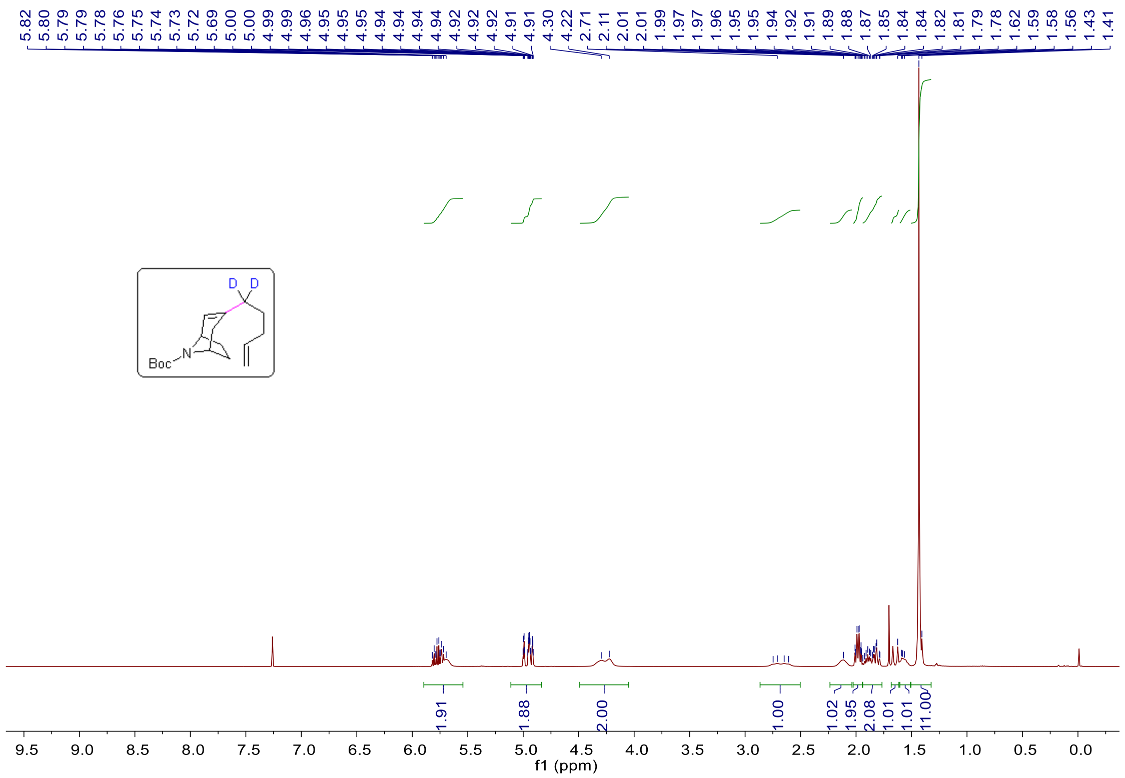

Supplementary Figure 314. ^{1}H NMR (400 MHz, $CDCl_3$) of **5ay**

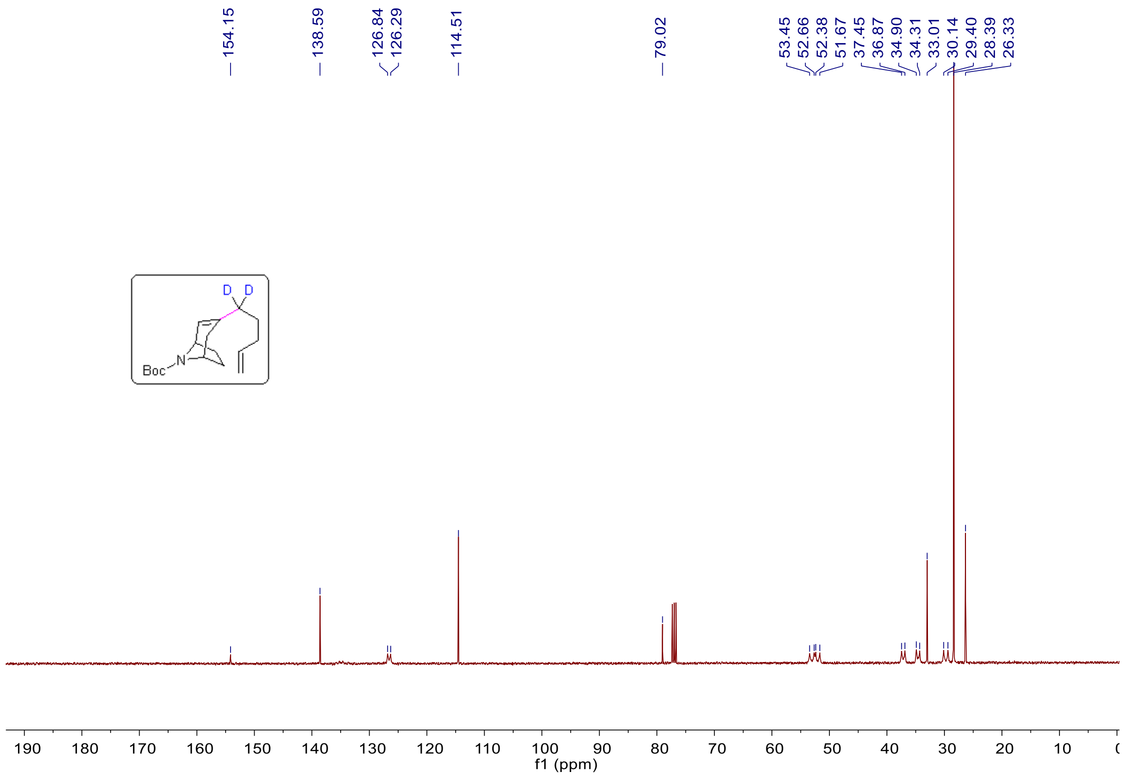

Supplementary Figure 315. ^{13}C NMR (101 MHz, $CDCl_3$) of **5ay**

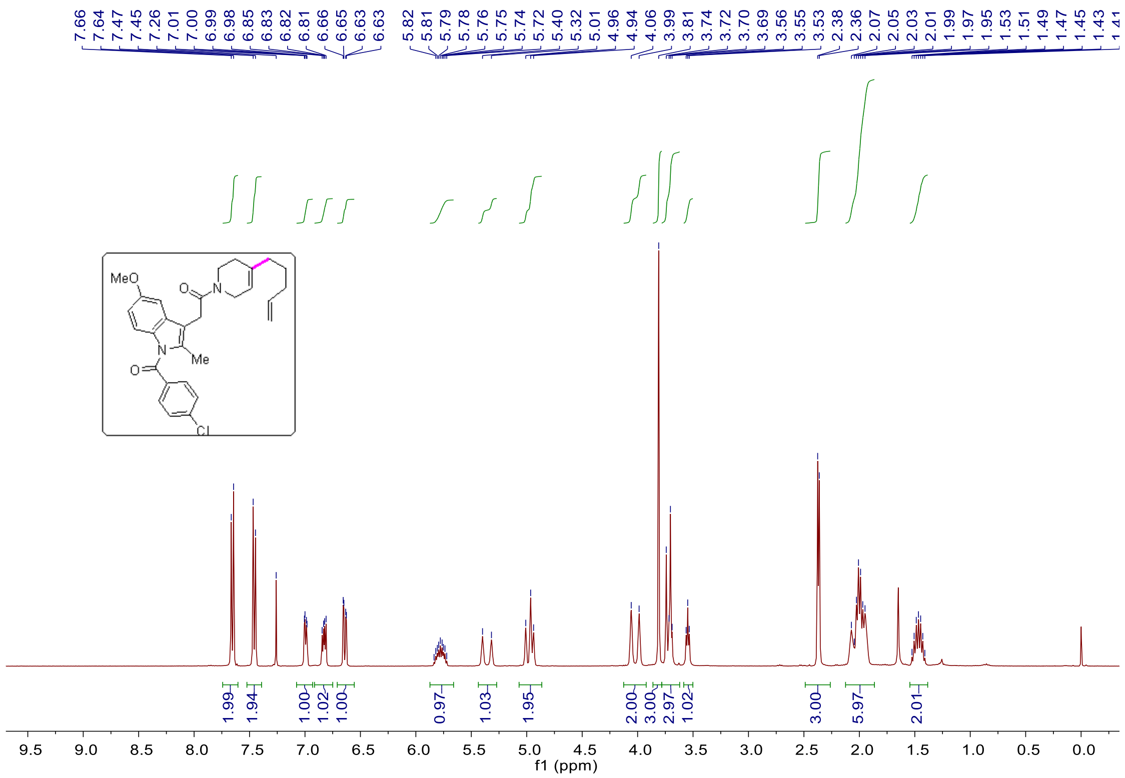

Supplementary Figure 316. ^{1}H NMR (400 MHz, $CDCl_3$) of **5az'**

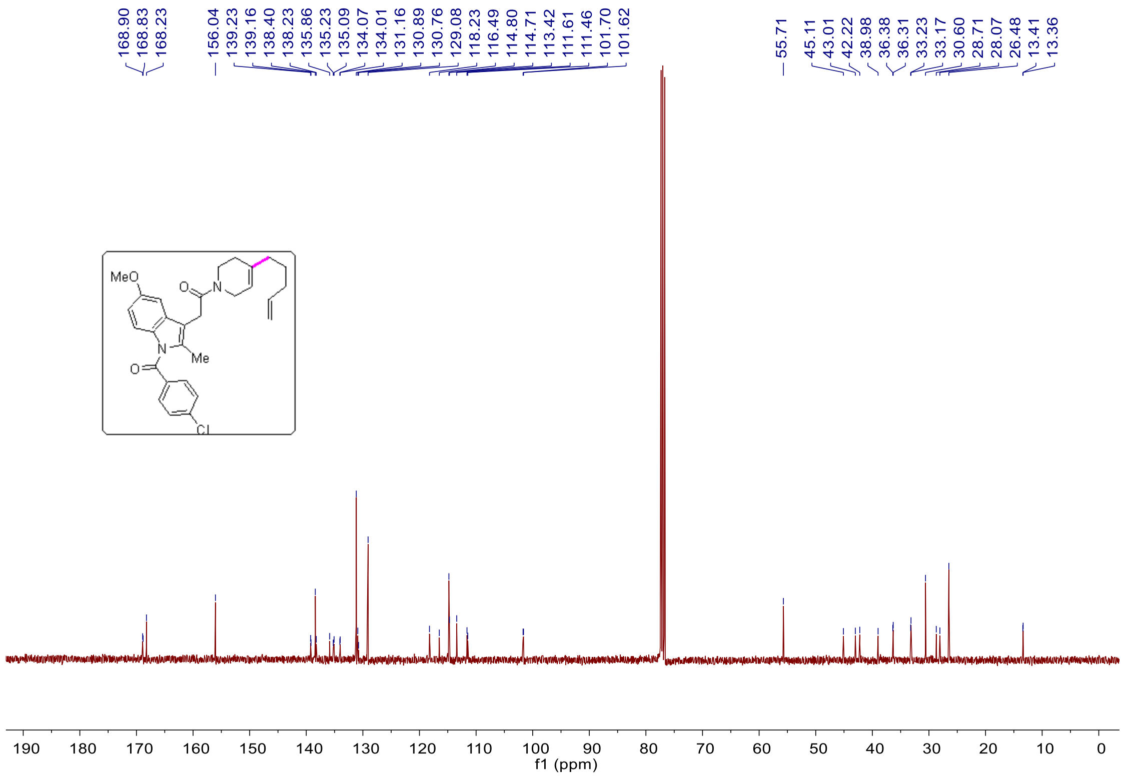

Supplementary Figure 317. ^{13}C NMR (101 MHz, $CDCl_3$) of **5az'**

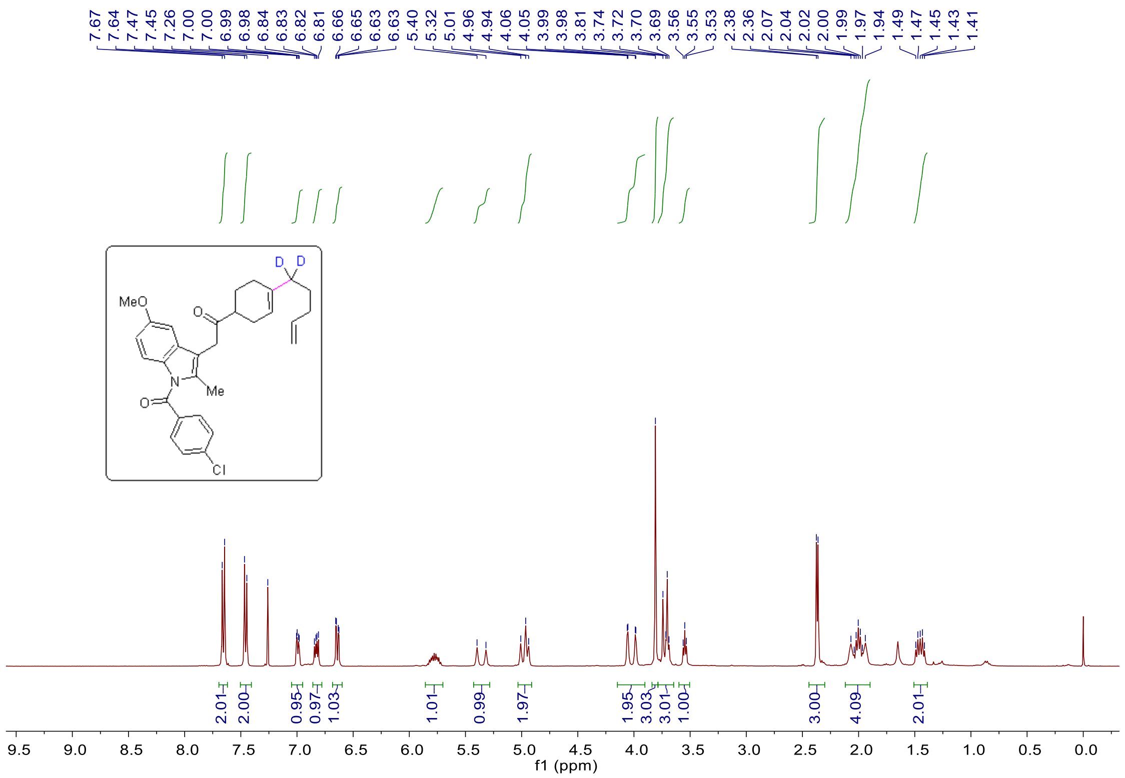

Supplementary Figure 318. ^{1}H NMR (400 MHz, $CDCl_3$) of **5az**

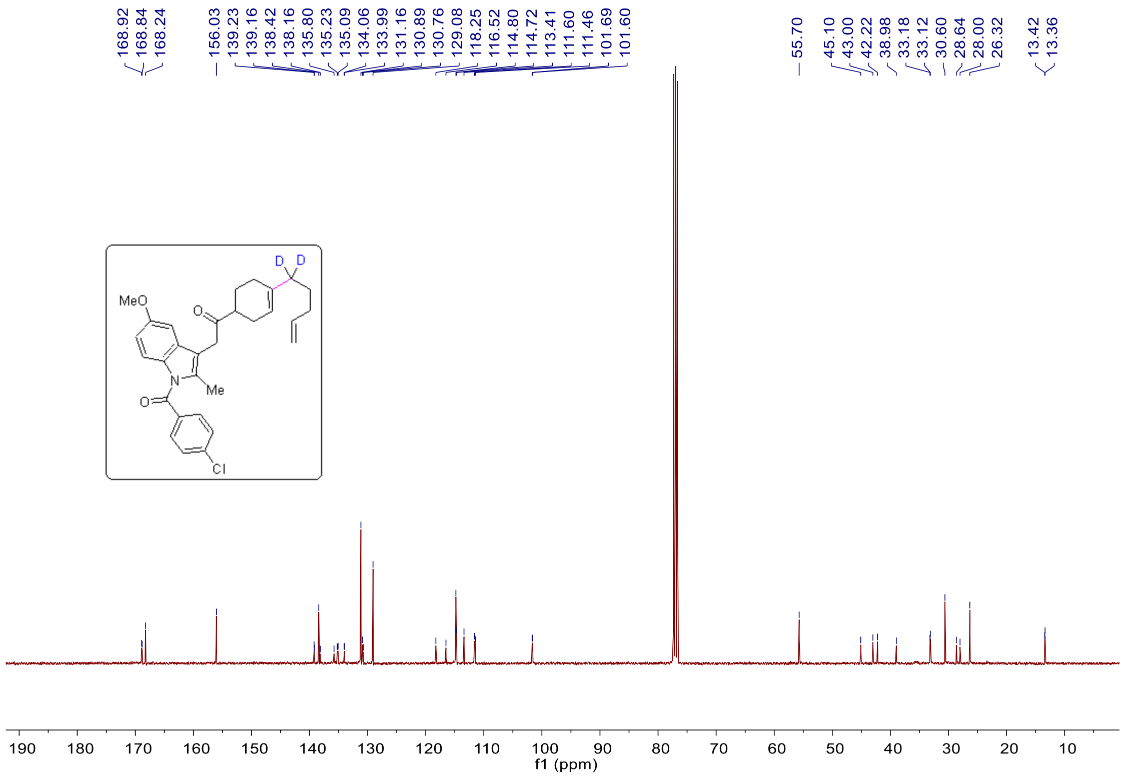

Supplementary Figure 319. ^{13}C NMR (101 MHz, $CDCl_3$) of **5az**

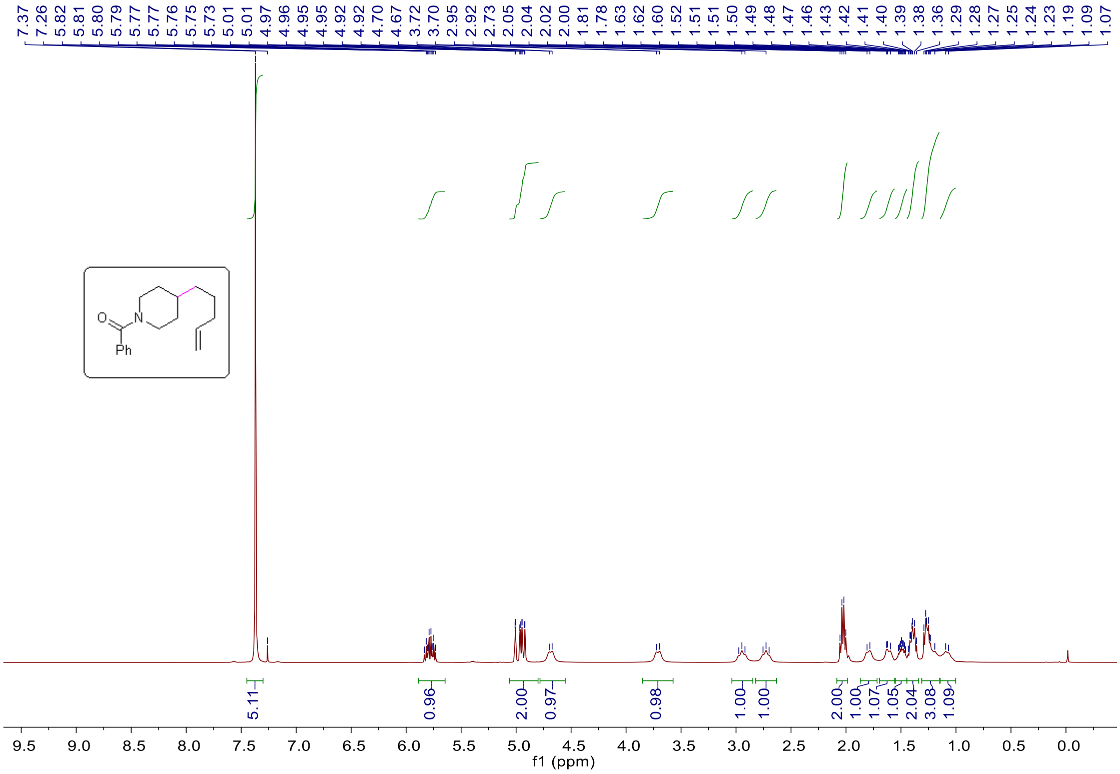

Supplementary Figure 320. ^{1}H NMR (400 MHz, $CDCl_3$) of **7aa'**

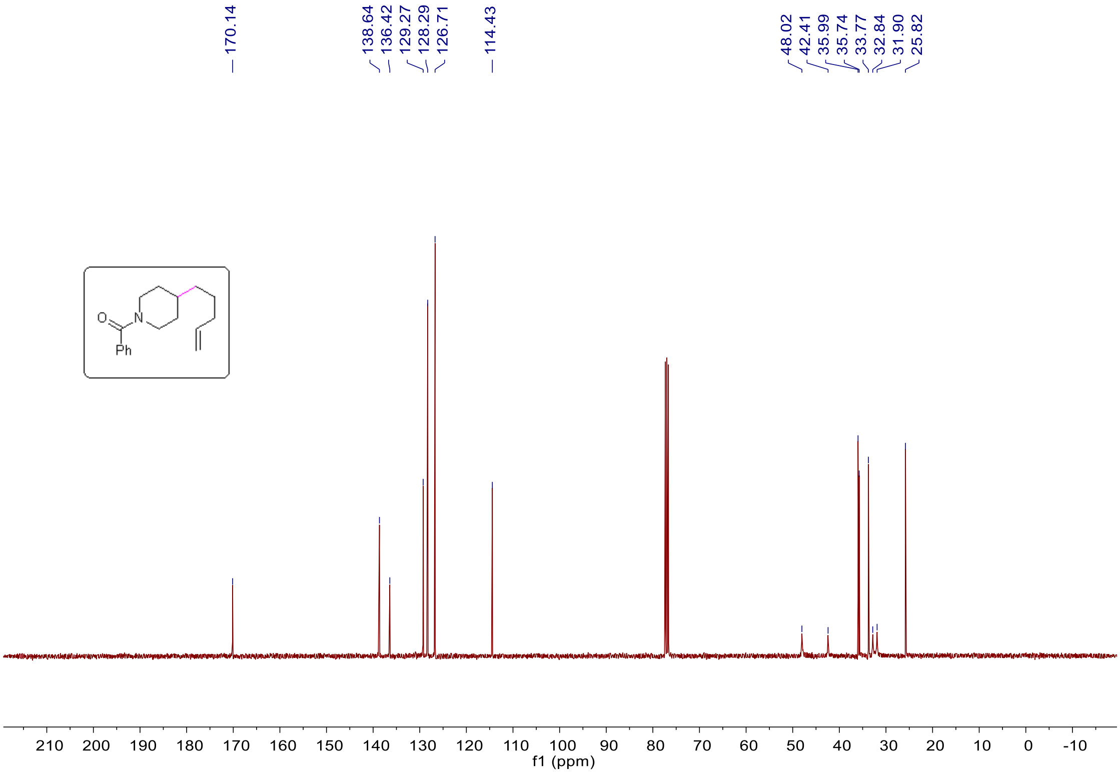

Supplementary Figure 321. ^{13}C NMR (101 MHz, $CDCl_3$) of **7aa'**

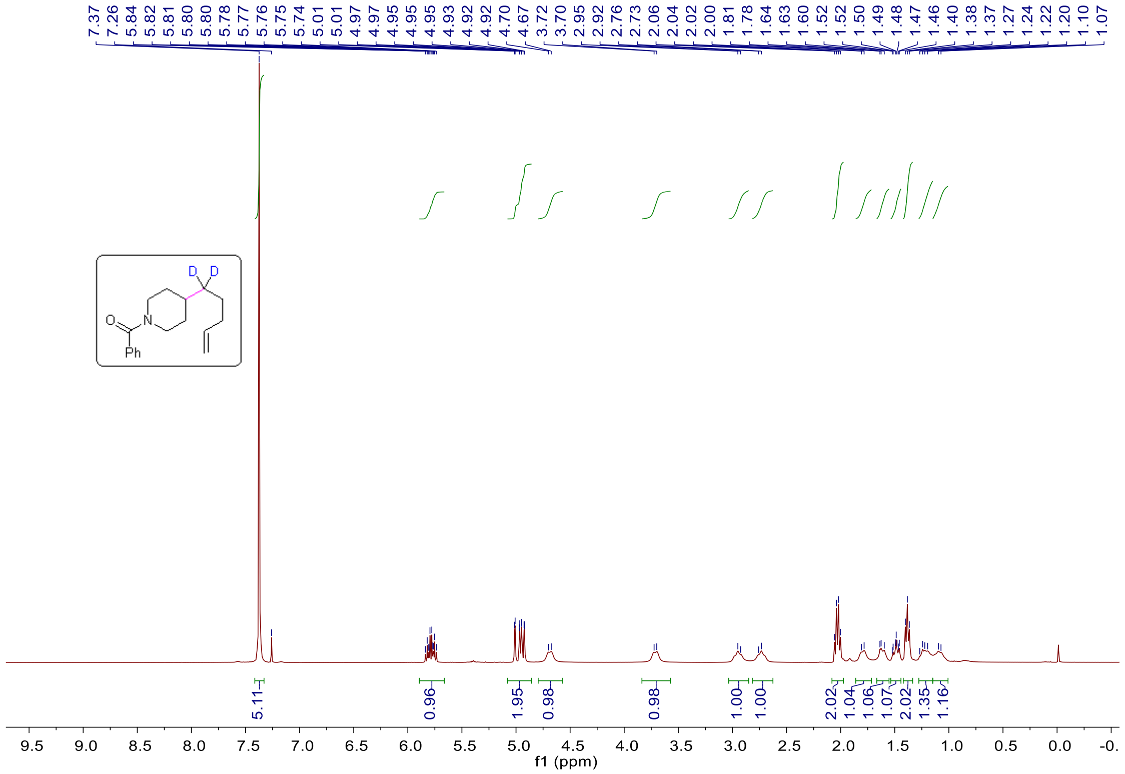

Supplementary Figure 322. ^{1}H NMR (400 MHz, $CDCl_3$) of **7aa**

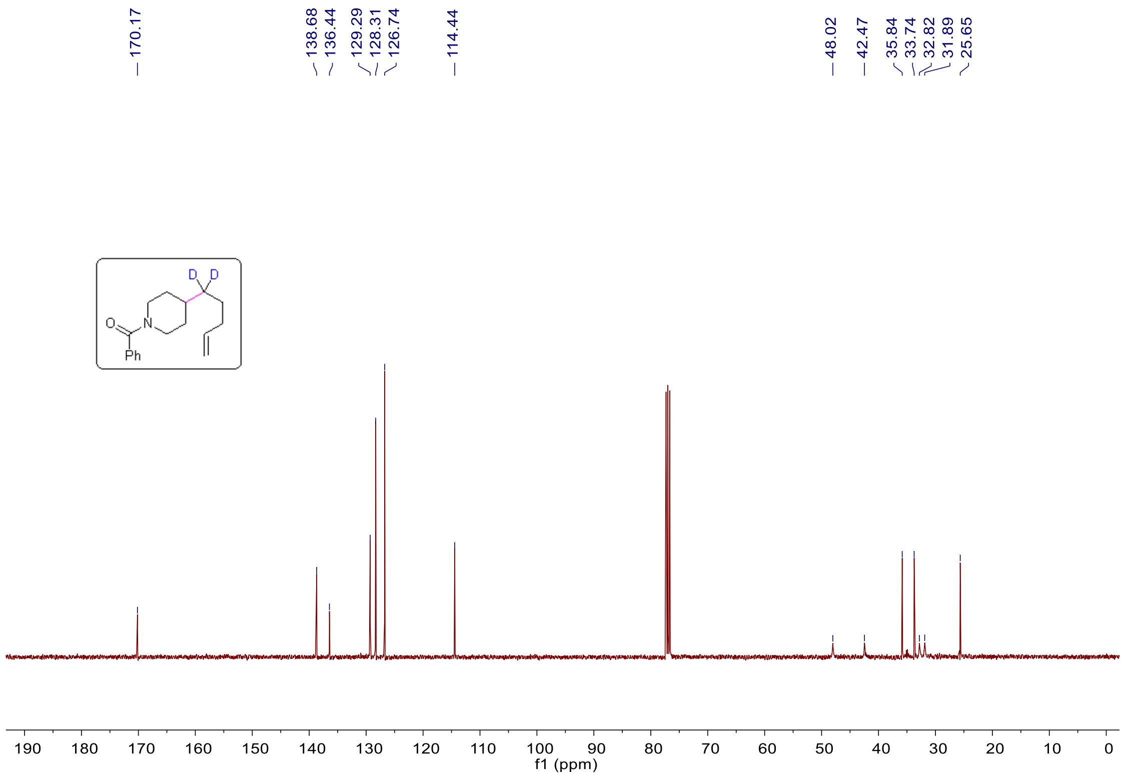

Supplementary Figure 323. ^{13}C NMR (101 MHz, $CDCl_3$) of **7aa**

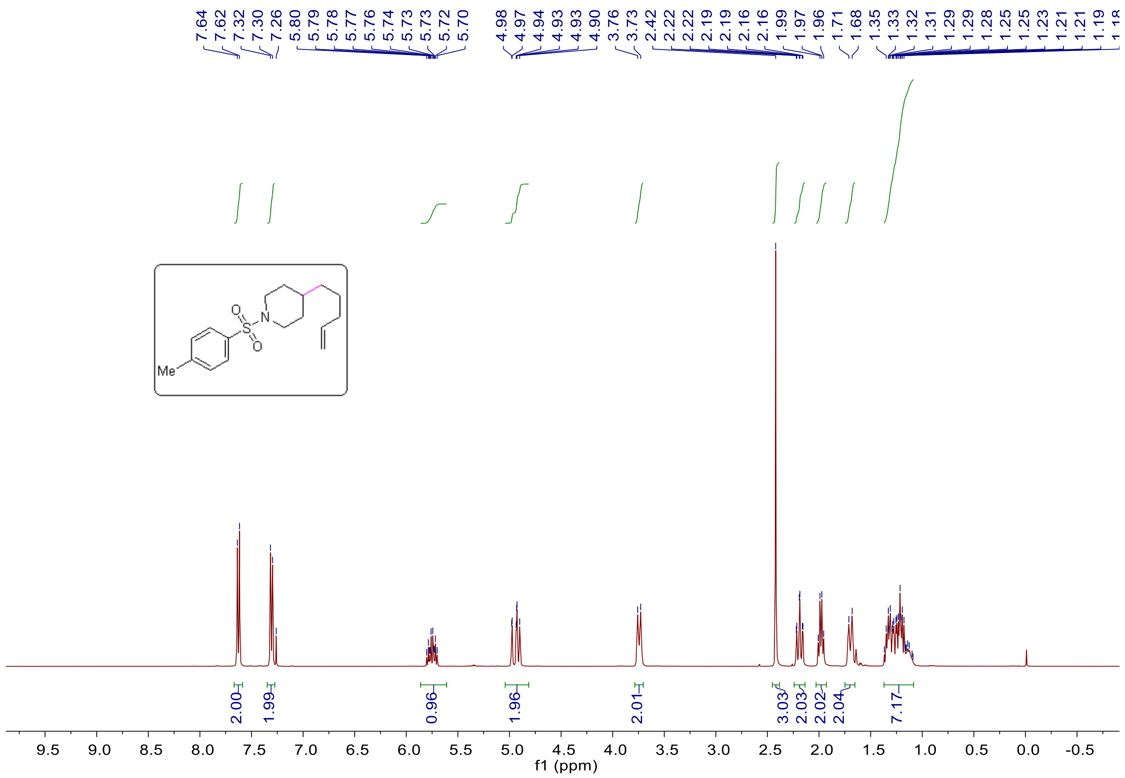

Supplementary Figure 324. ^{1}H NMR (400 MHz, $CDCl_3$) of **7ab'**

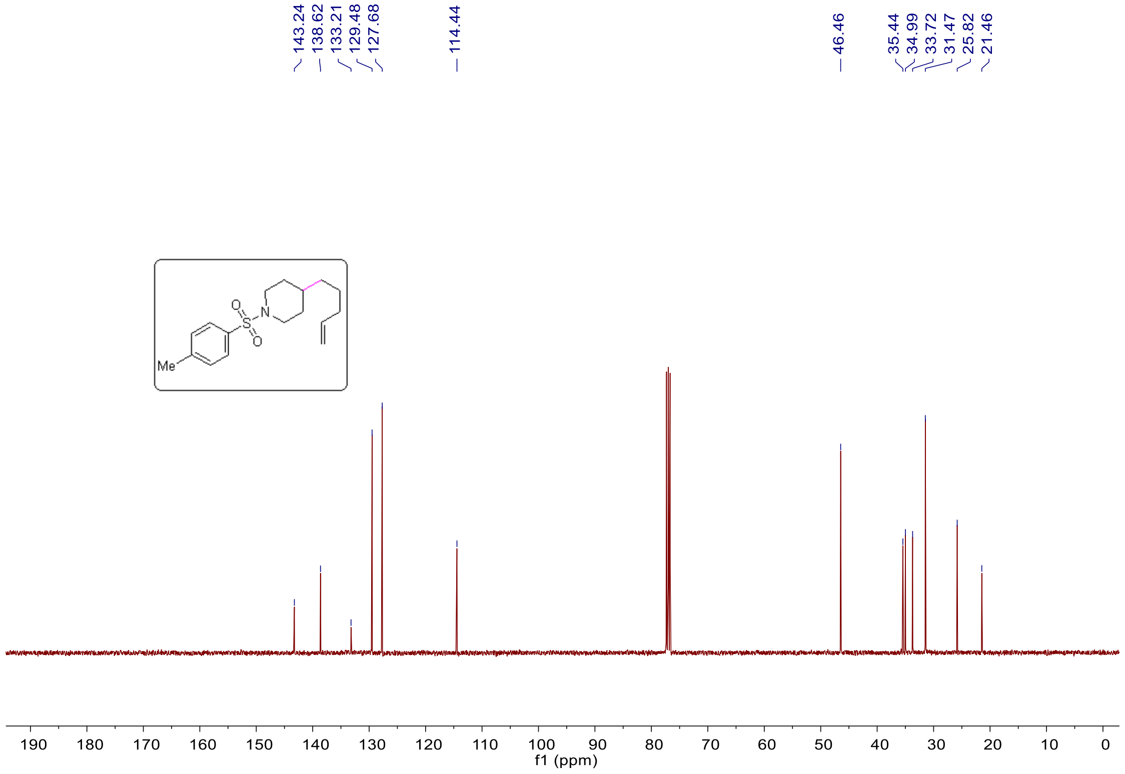

Supplementary Figure 325. ^{13}C NMR (101 MHz, $CDCl_3$) of **7ab'**

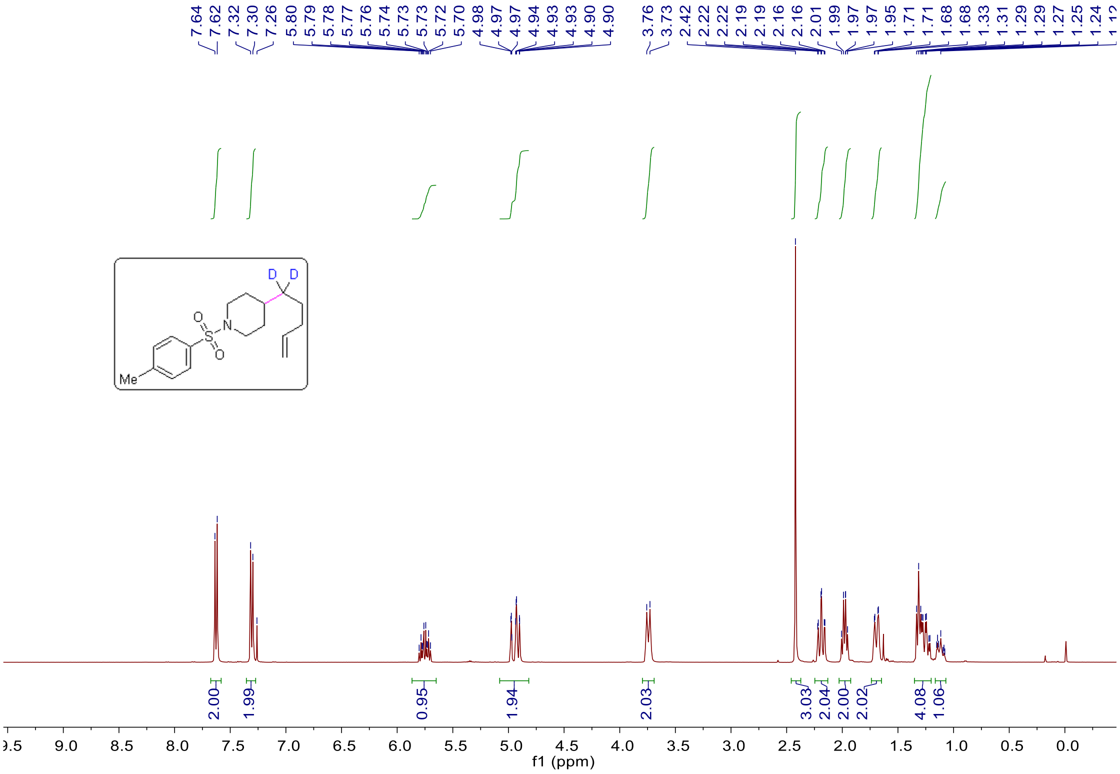

Supplementary Figure 326. ^{1}H NMR (400 MHz, $CDCl_3$) of **7ab**

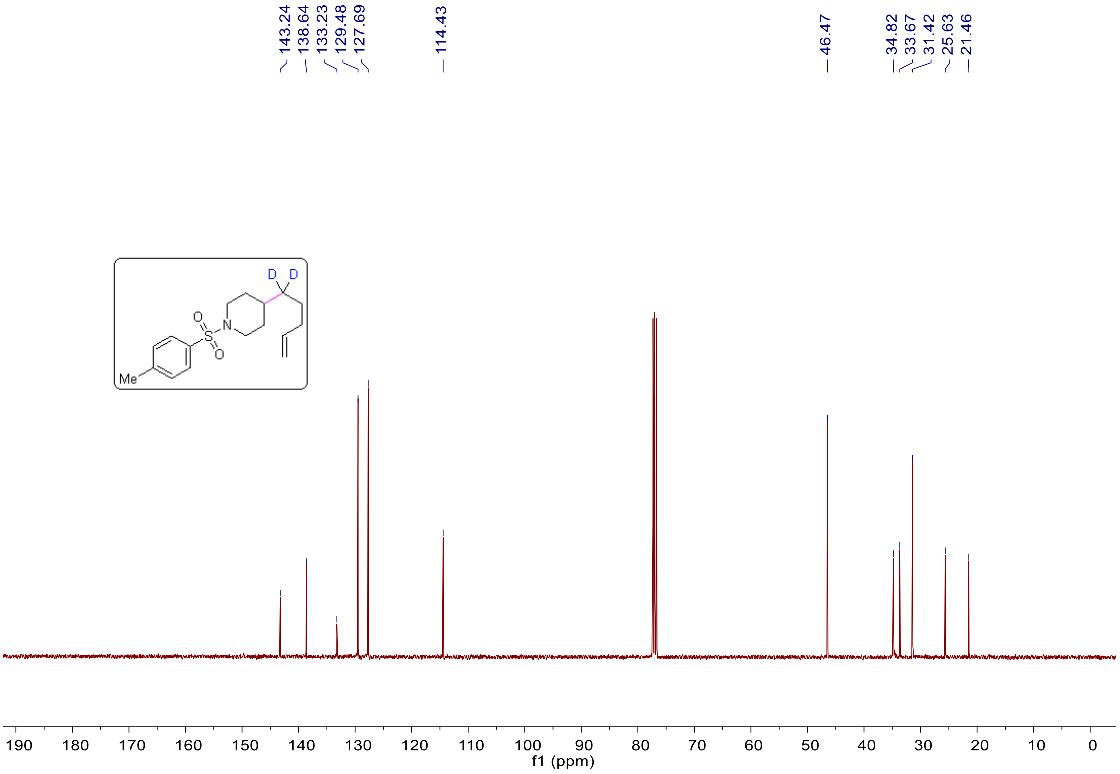

Supplementary Figure 327. ^{13}C NMR (101 MHz, $CDCl_3$) of **7ab**

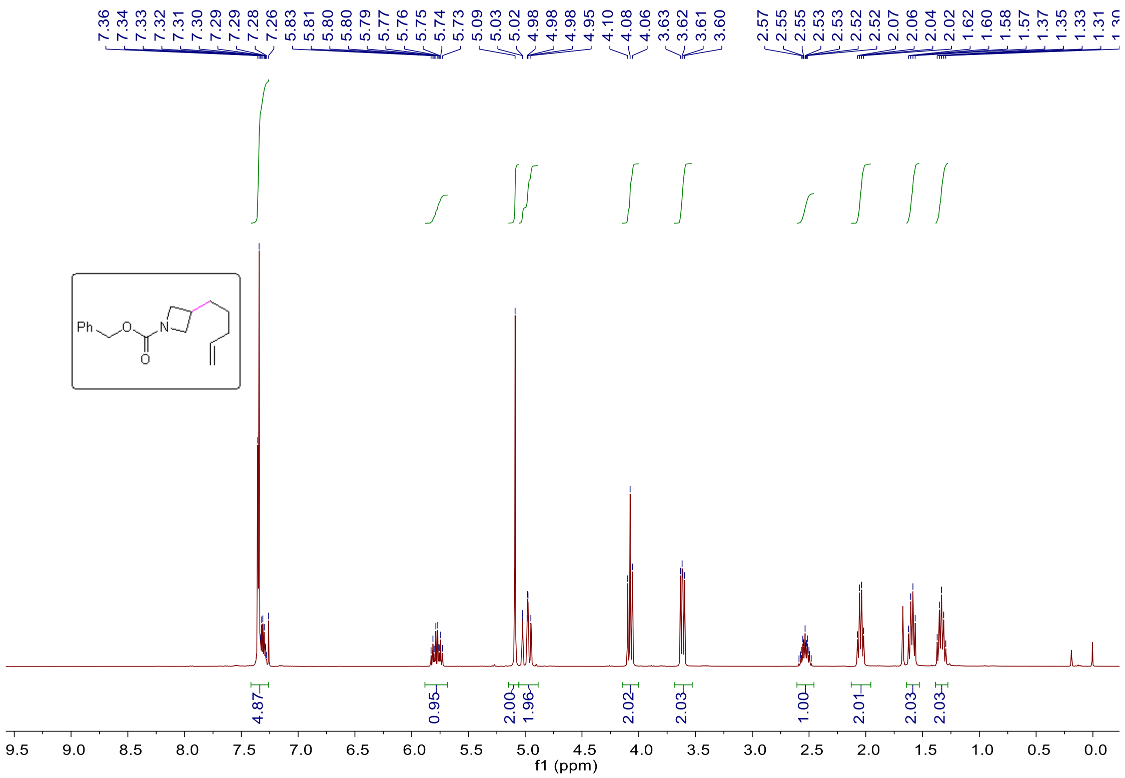

Supplementary Figure 328. ^{1}H NMR (400 MHz, $CDCl_3$) of **7ac'**

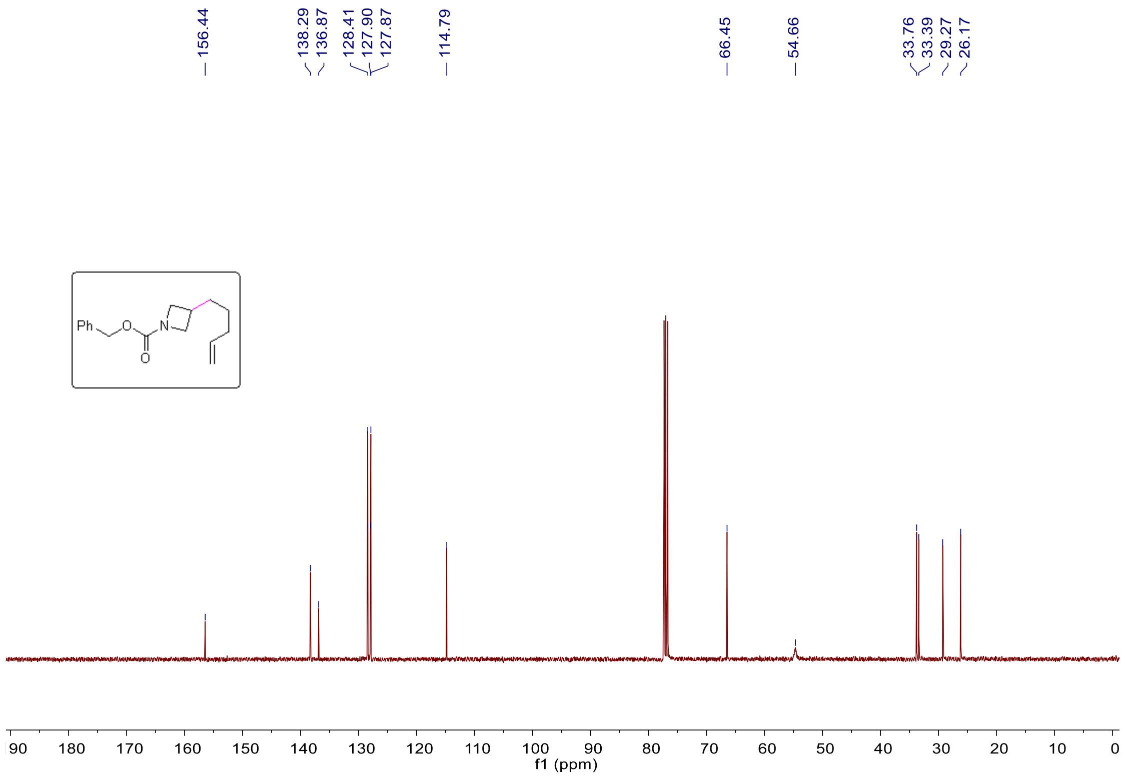

Supplementary Figure 329. ^{13}C NMR (101 MHz, $CDCl_3$) of **7ac'**

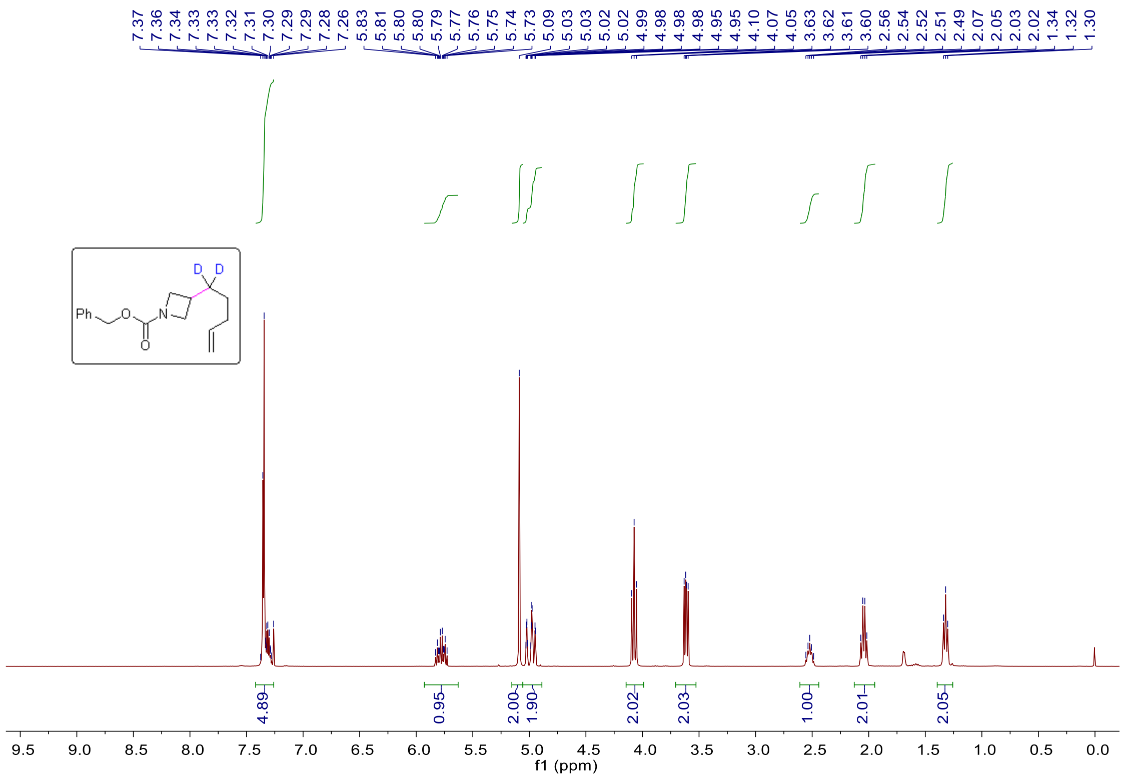

Supplementary Figure 330. ^{1}H NMR (400 MHz, $CDCl_3$) of **7ac**

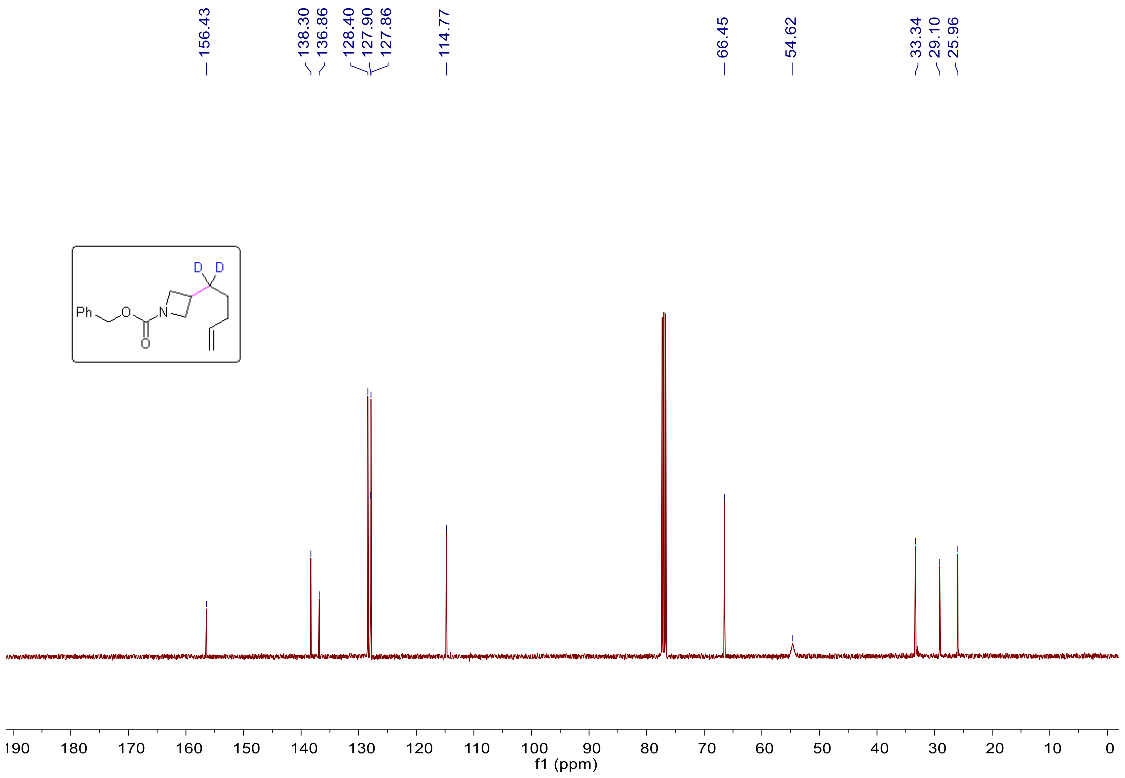

Supplementary Figure 331. ^{13}C NMR (101 MHz, $CDCl_3$) of **7ac**

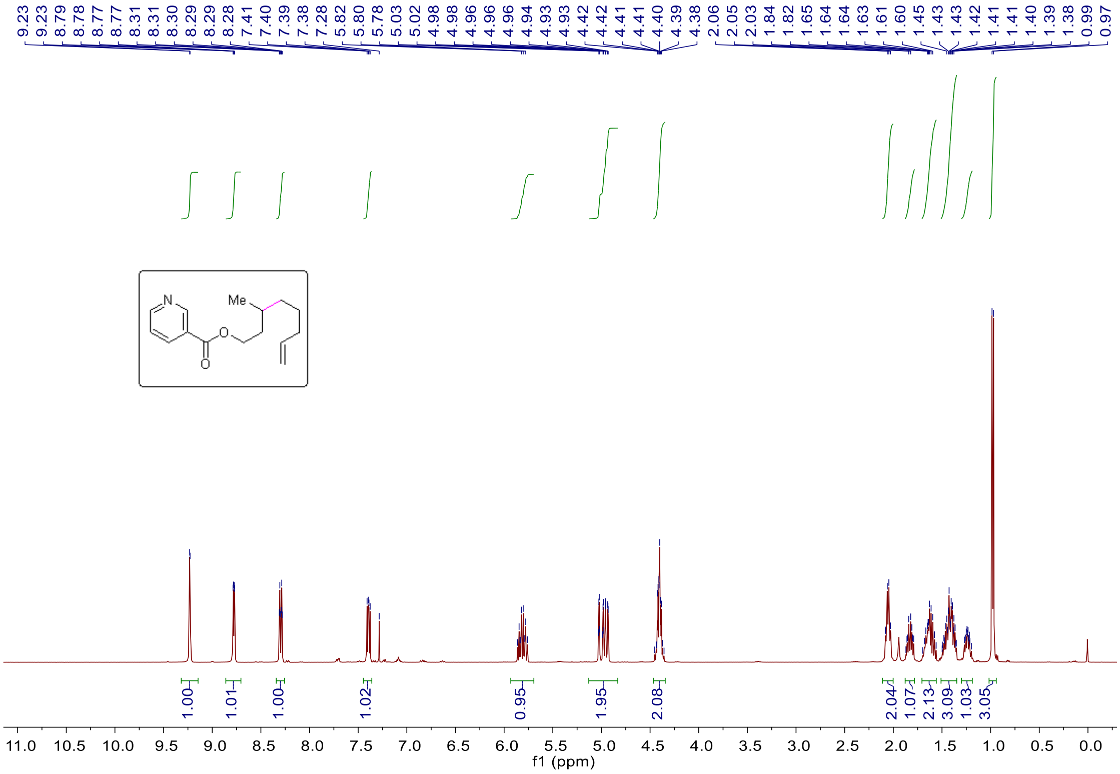

Supplementary Figure 332. ^{1}H NMR (400 MHz, $CDCl_3$) of **7ad'**

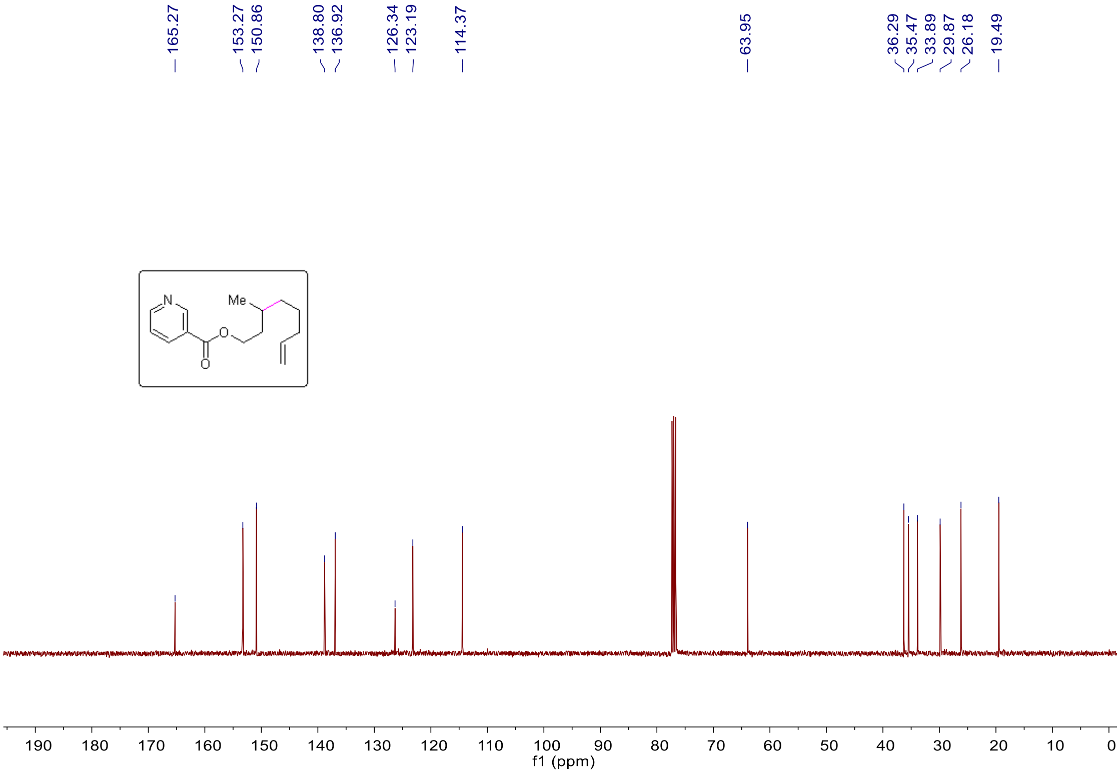

Supplementary Figure 333. ^{13}C NMR (101 MHz, $CDCl_3$) of **7ad'**

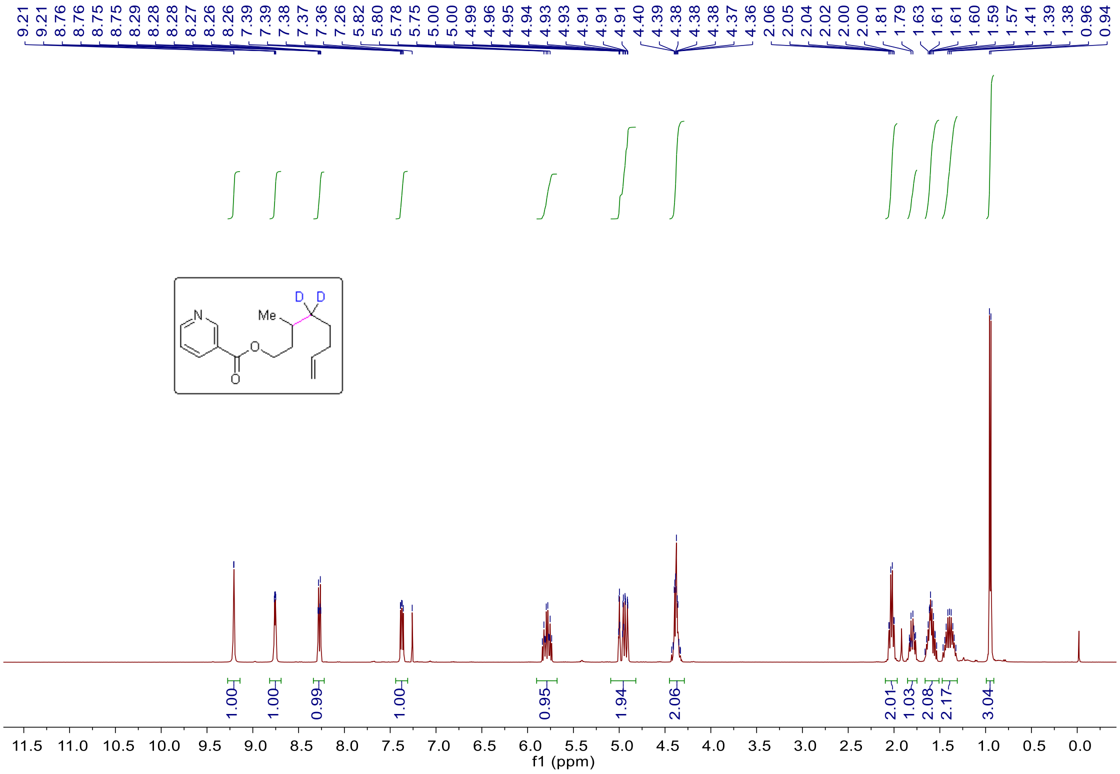

Supplementary Figure 334. ^{1}H NMR (400 MHz, $CDCl_3$) of **7ad**

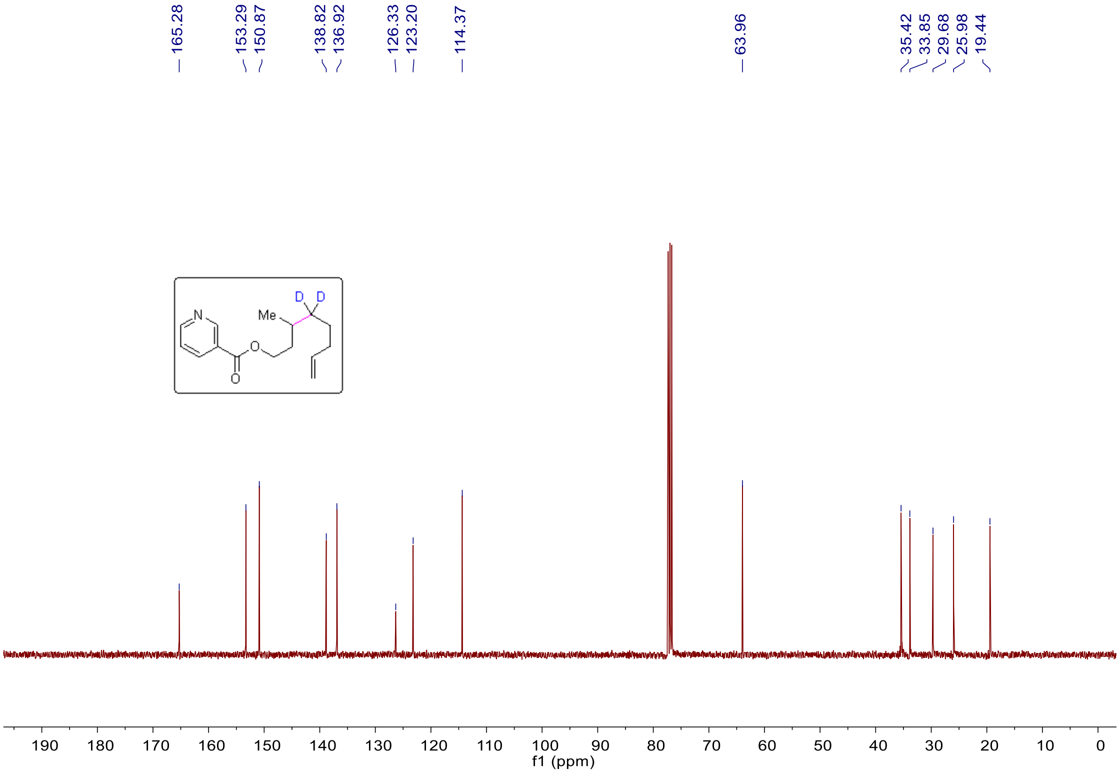

Supplementary Figure 335. ^{13}C NMR (101 MHz, $CDCl_3$) of **7ad**

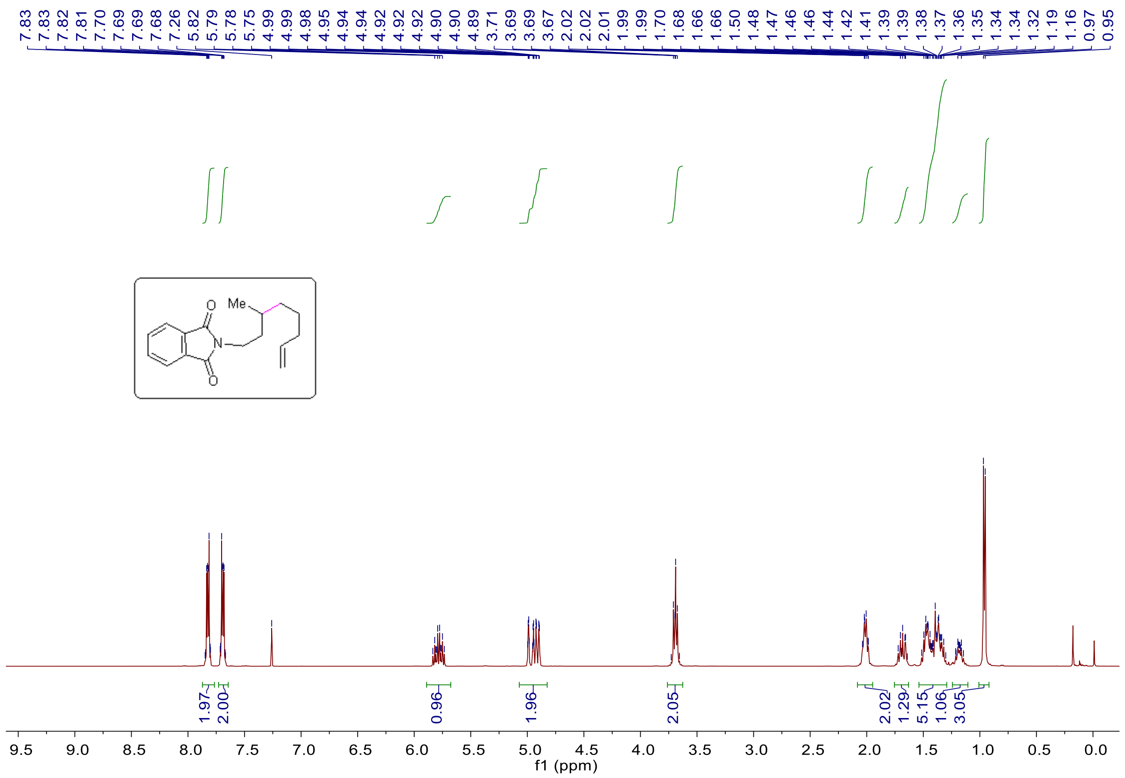

Supplementary Figure 336. ^{1}H NMR (400 MHz, $CDCl_3$) of **7ae'**

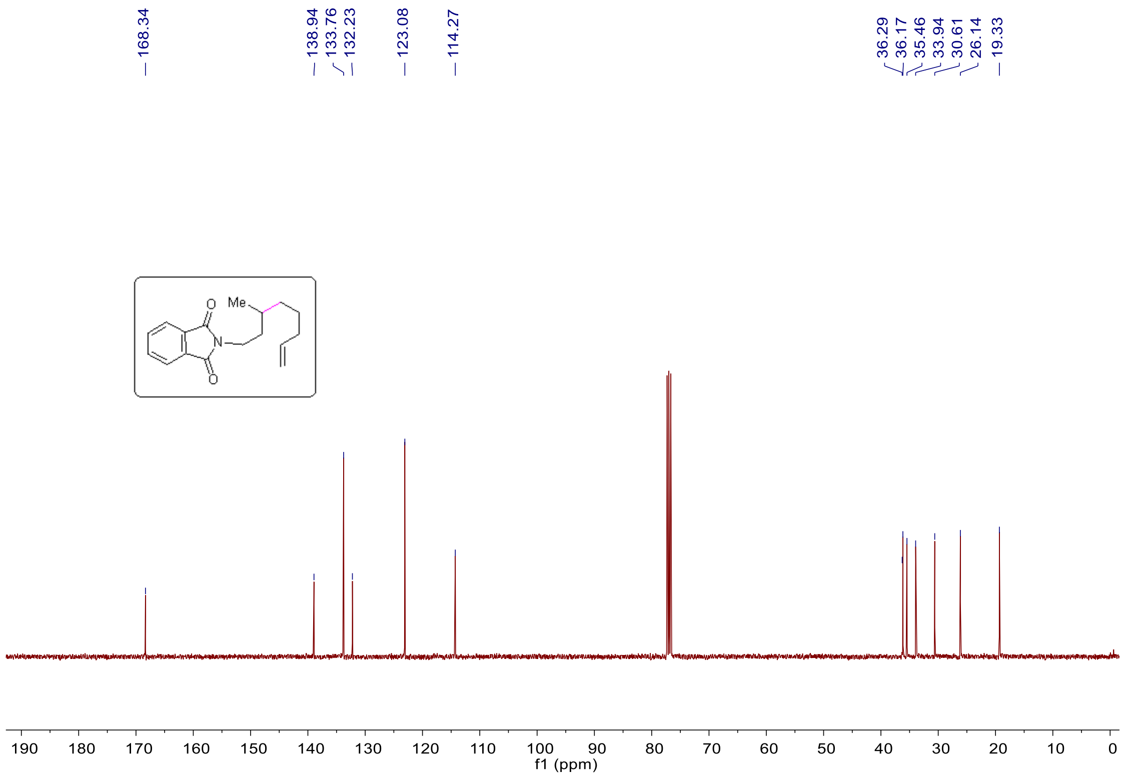

Supplementary Figure 337. ^{13}C NMR (101 MHz, $CDCl_3$) of **7ae'**

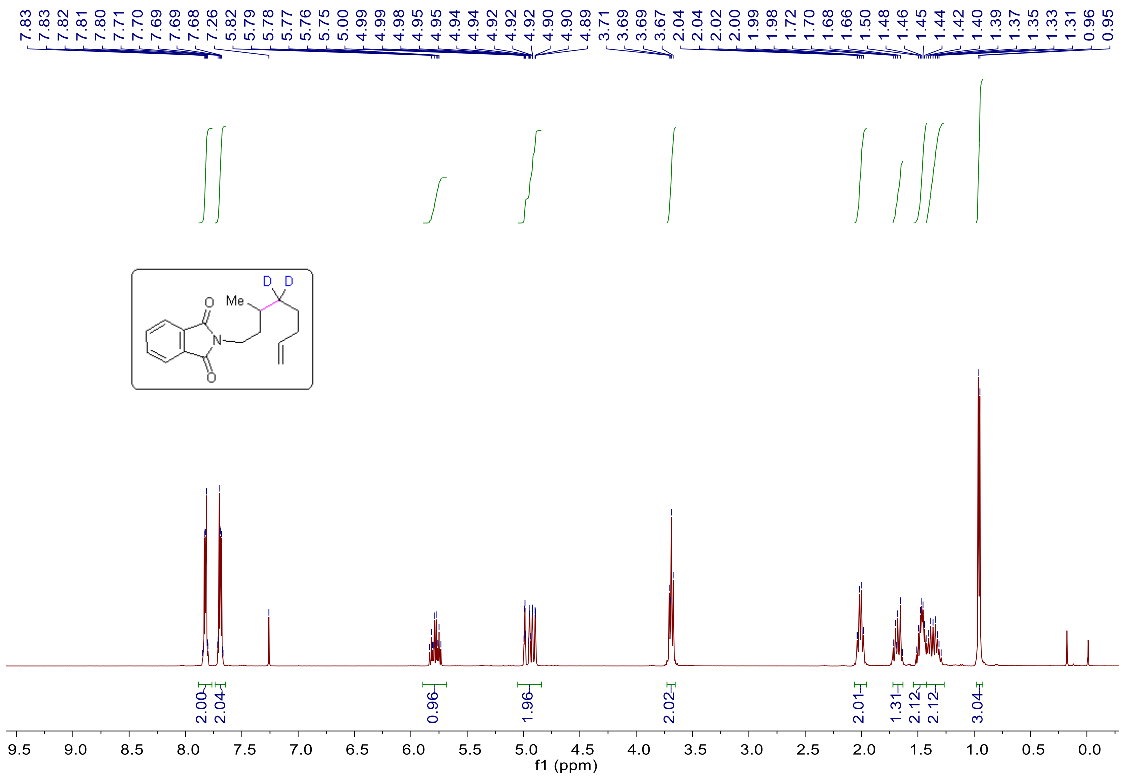

Supplementary Figure 338. ^{1}H NMR (400 MHz, $CDCl_3$) of **7ae**

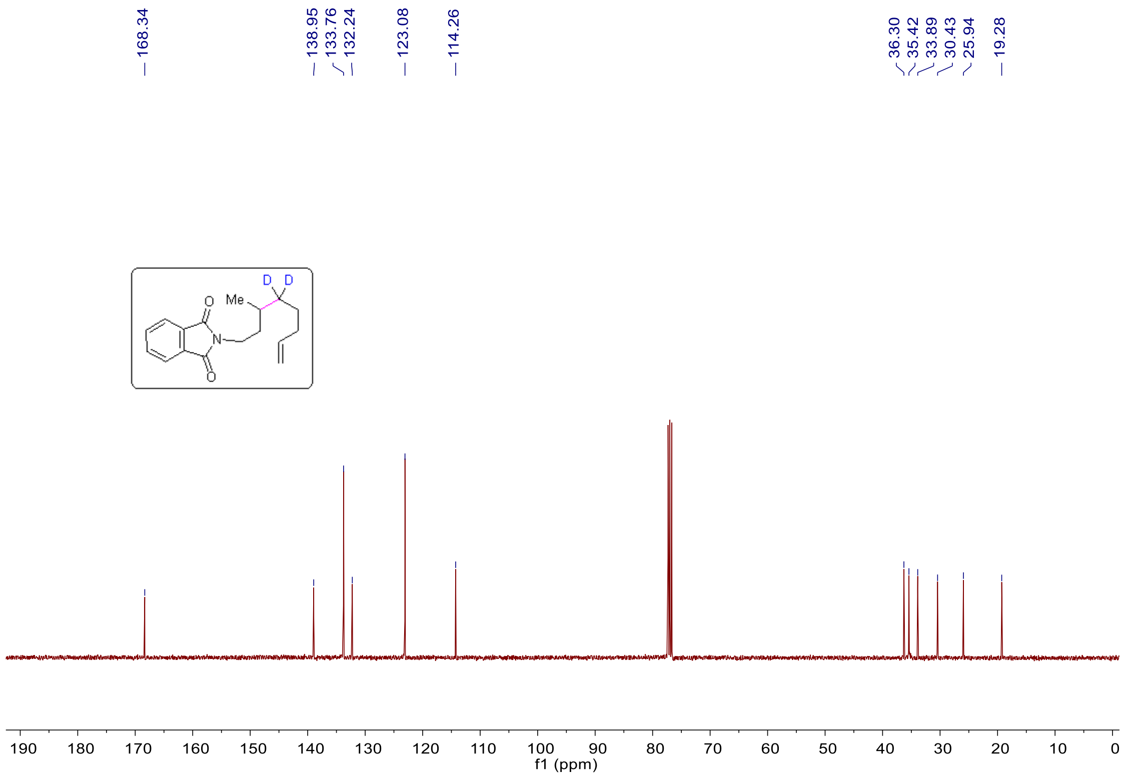

Supplementary Figure 339. ^{13}C NMR (101 MHz, $CDCl_3$) of **7ae**

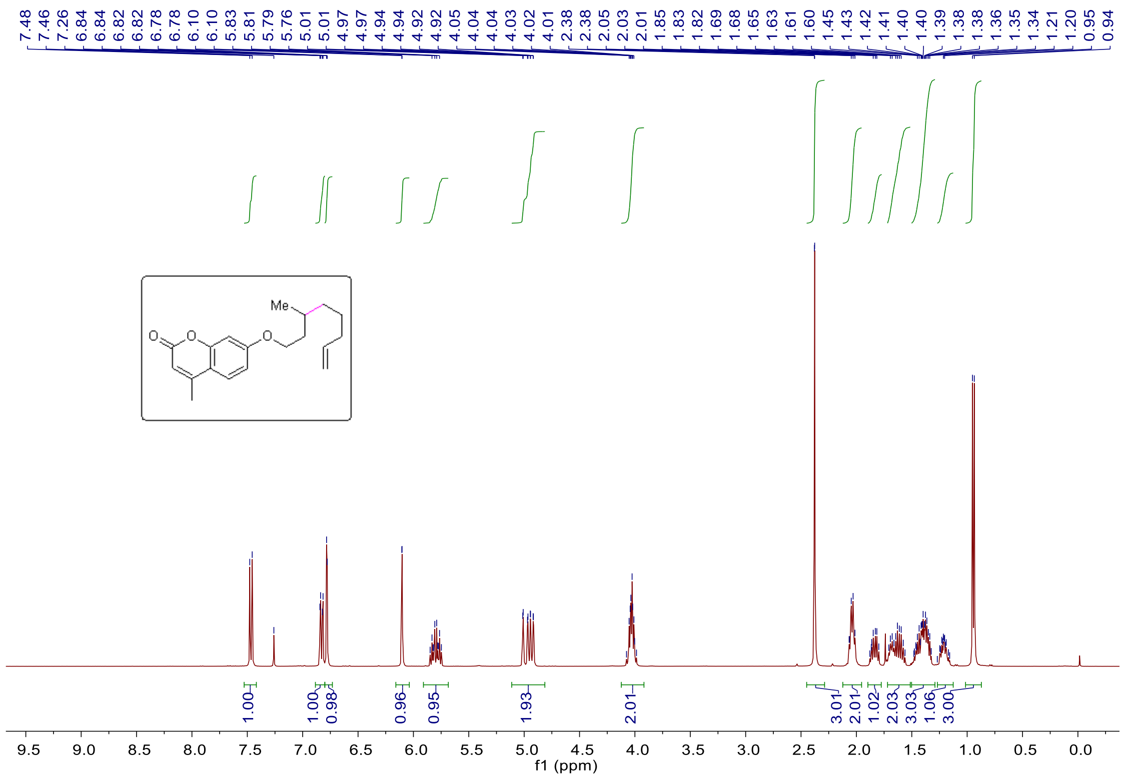

Supplementary Figure 340. ^{1}H NMR (400 MHz, $CDCl_3$) of **7af'**

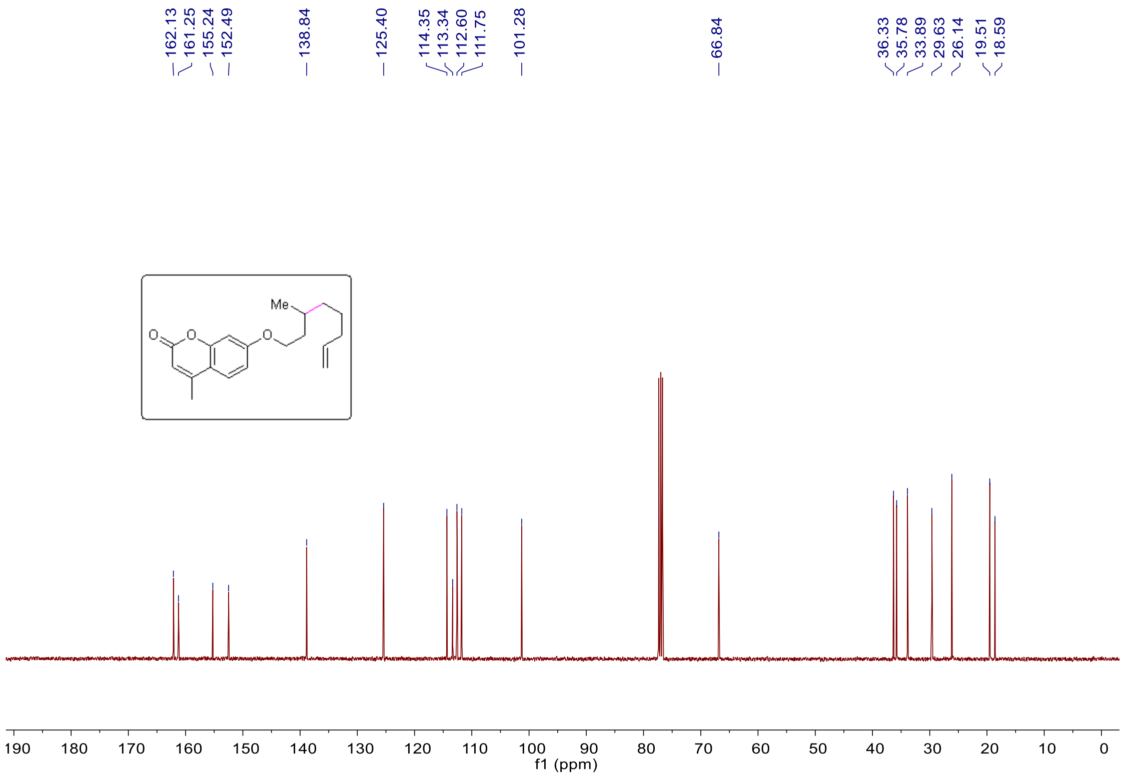

Supplementary Figure 341. ^{13}C NMR (101 MHz, $CDCl_3$) of **7af'**

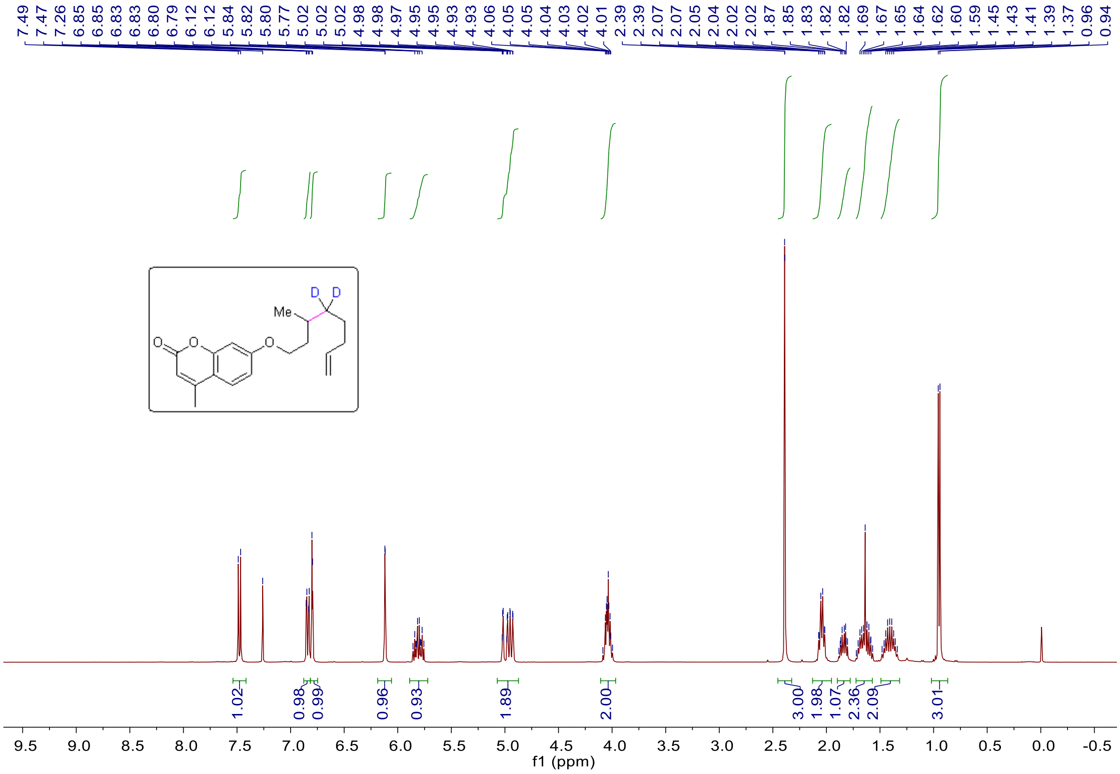

Supplementary Figure 342. ^{1}H NMR (400 MHz, $CDCl_3$) of **7af**

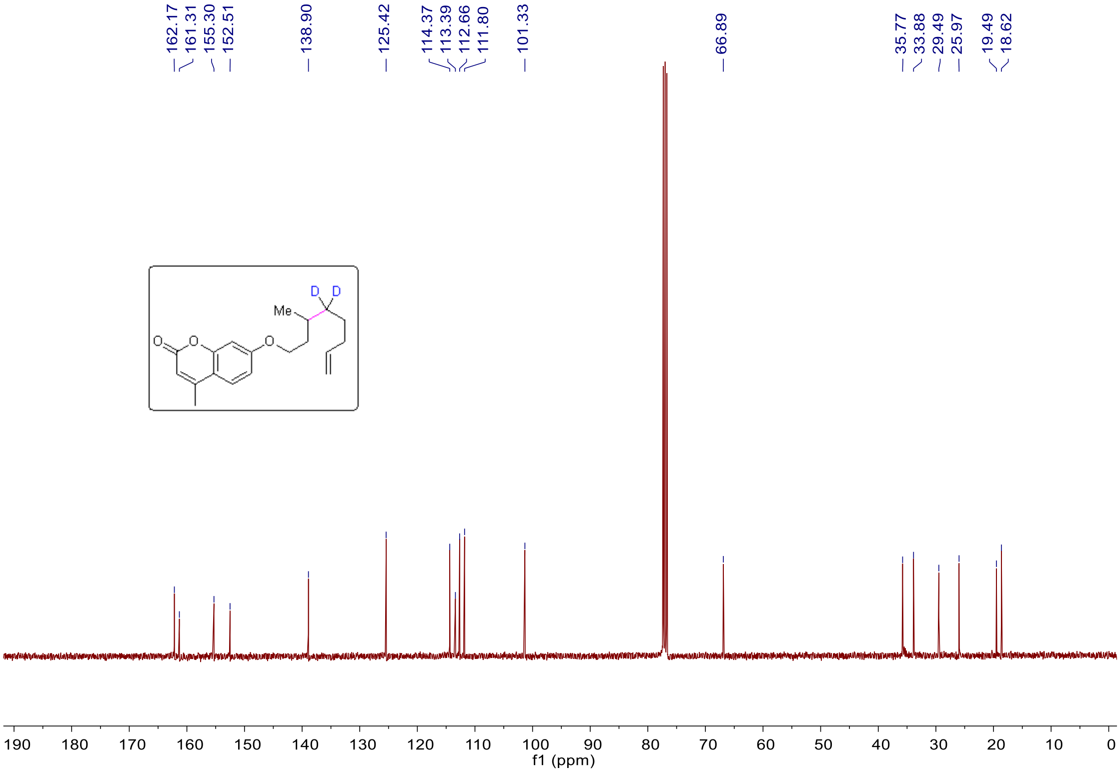

Supplementary Figure 343. ^{13}C NMR (101 MHz, $CDCl_3$) of **7af**

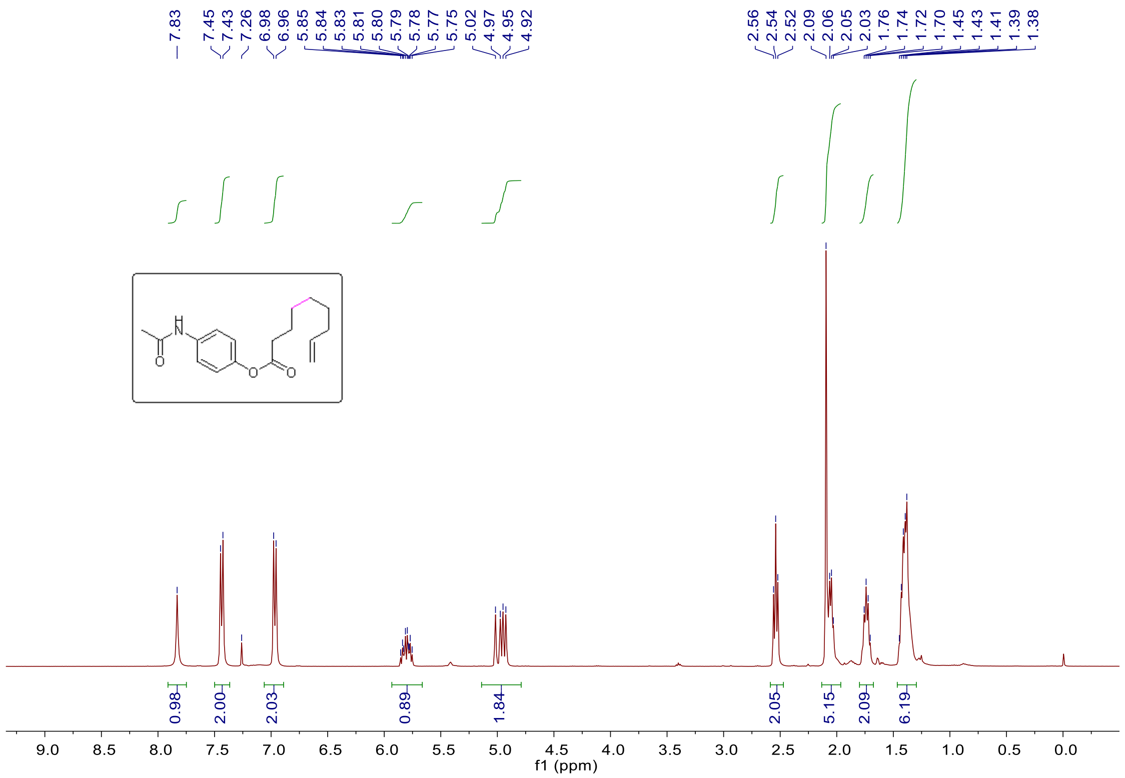

Supplementary Figure 344. ^{1}H NMR (400 MHz, $CDCl_3$) of **7ag'**

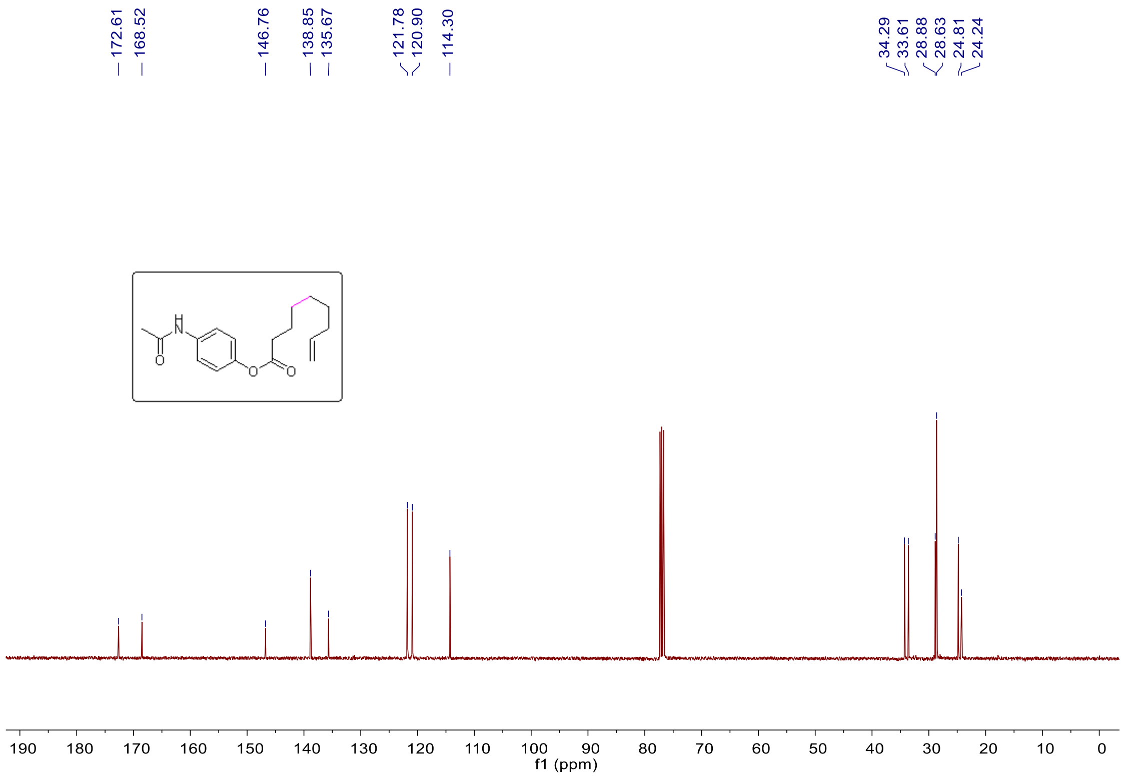

Supplementary Figure 345. ^{13}C NMR (101 MHz, $CDCl_3$) of **7ag'**

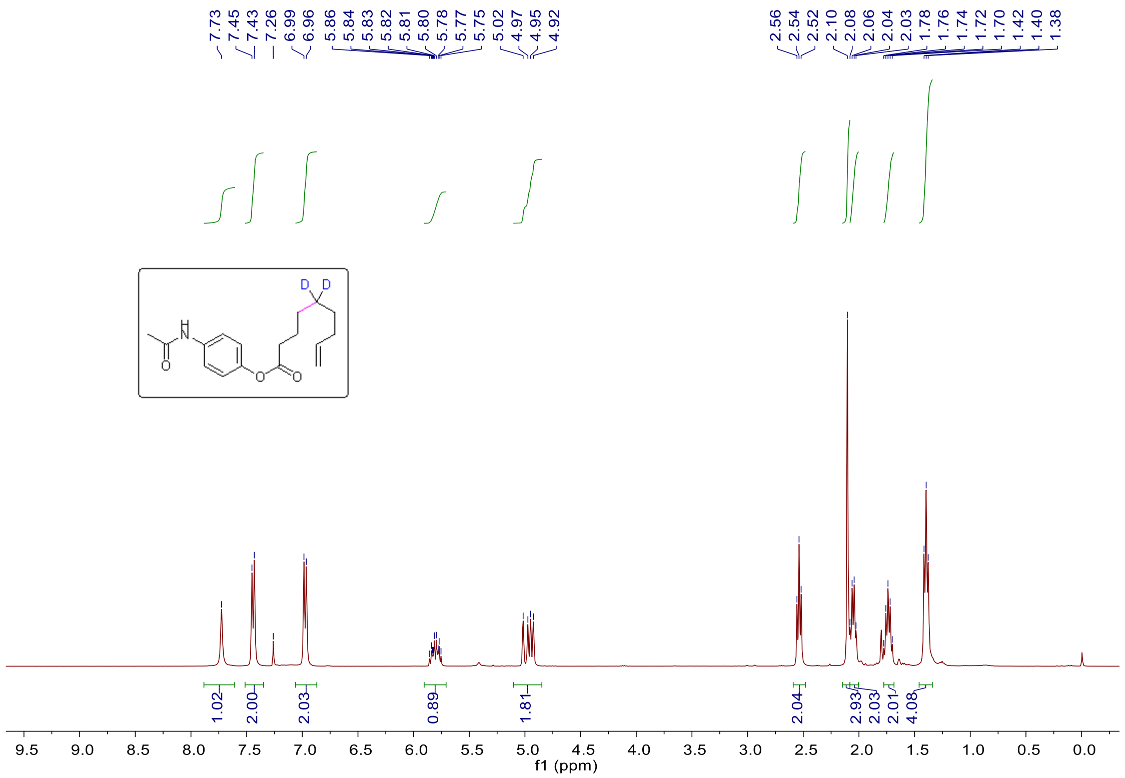

Supplementary Figure 346. ^{1}H NMR (400 MHz, $CDCl_3$) of **7ag**

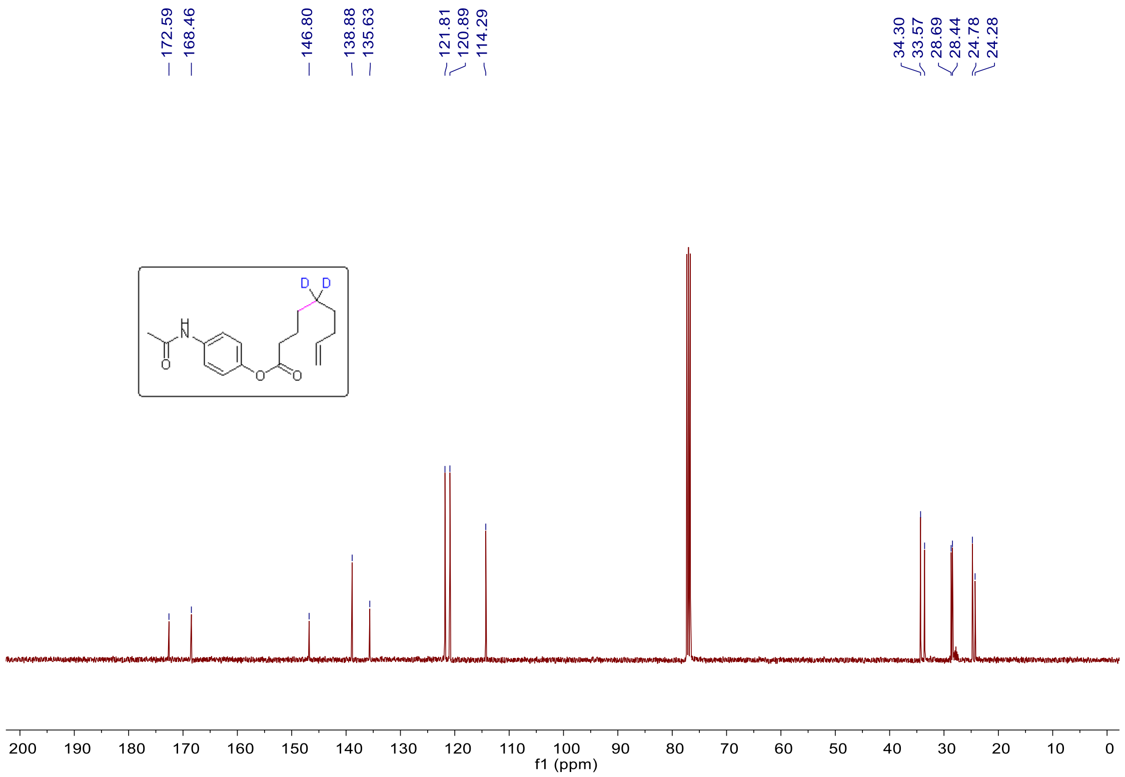

Supplementary Figure 347. ^{13}C NMR (101 MHz, $CDCl_3$) of **7ag**

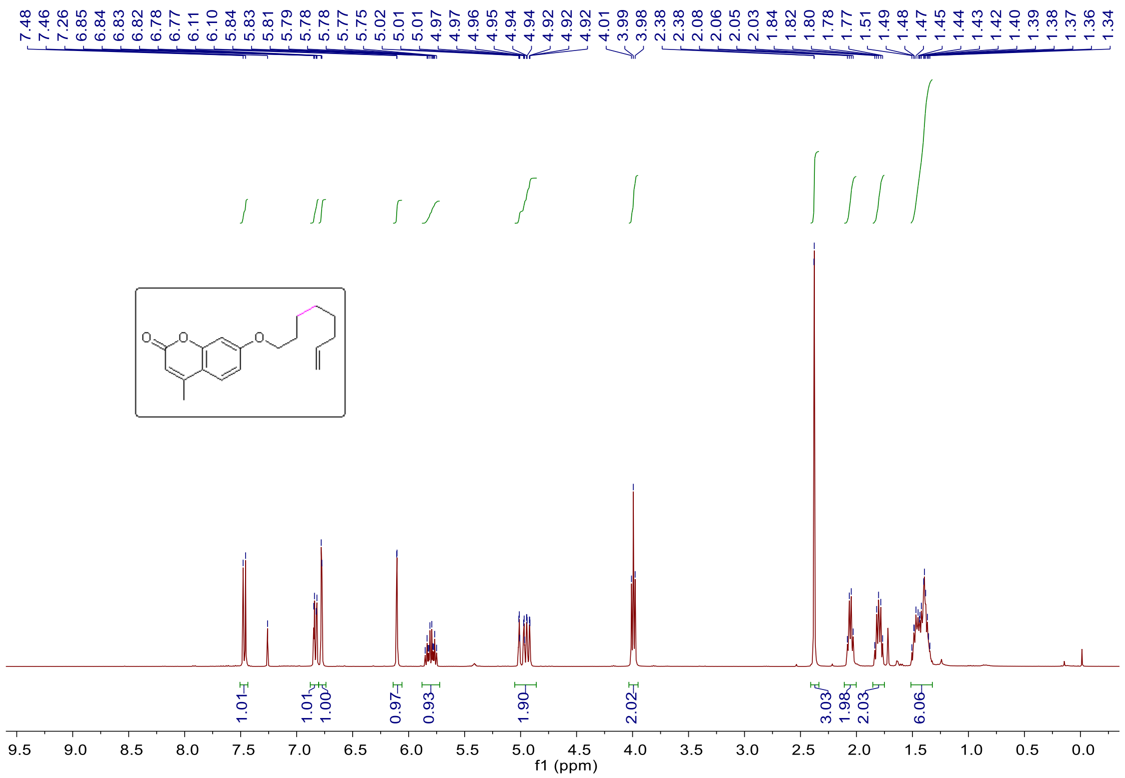

Supplementary Figure 348. ^{1}H NMR (400 MHz, $CDCl_3$) of **7ah'**

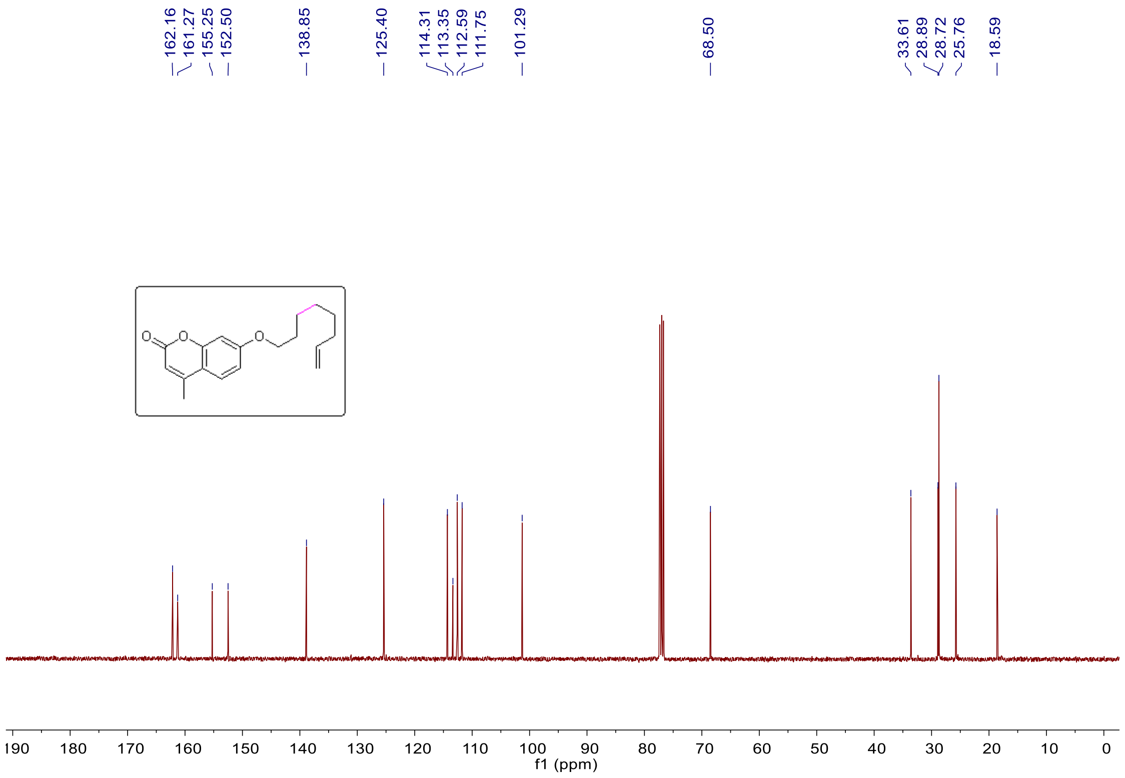

Supplementary Figure 349. ^{13}C NMR (101 MHz, $CDCl_3$) of **7ah'**

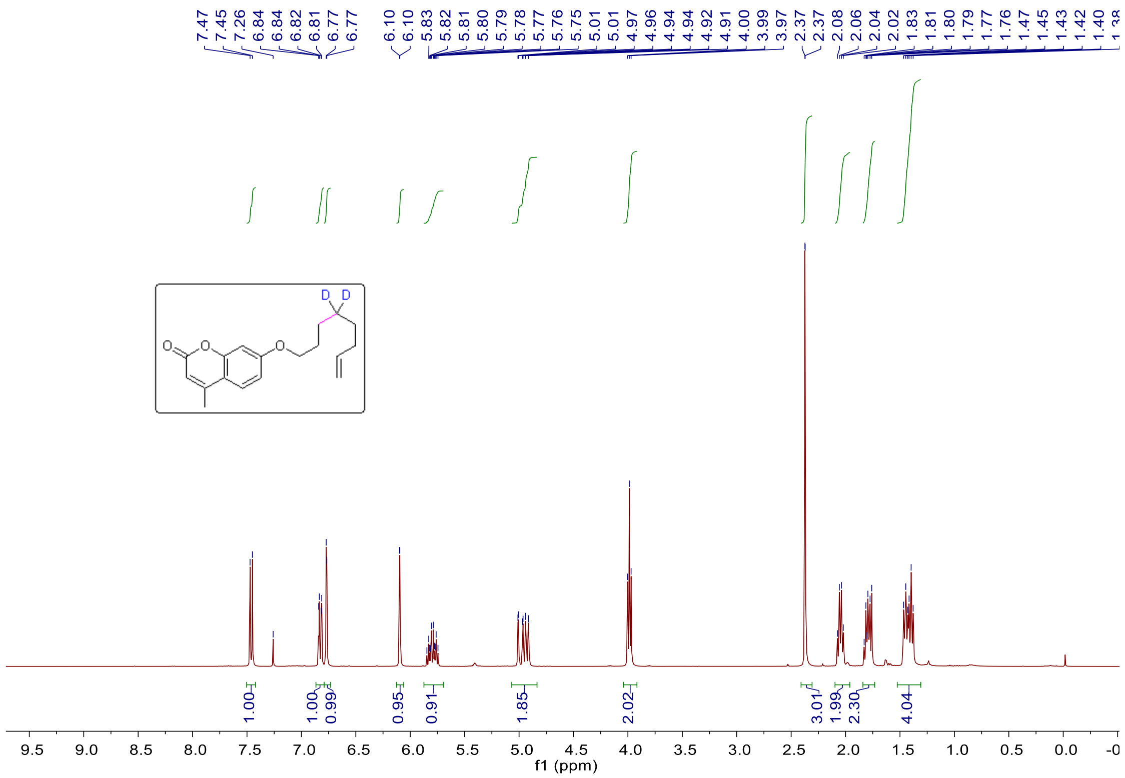

Supplementary Figure 350. ^{1}H NMR (400 MHz, $CDCl_3$) of **7ah**

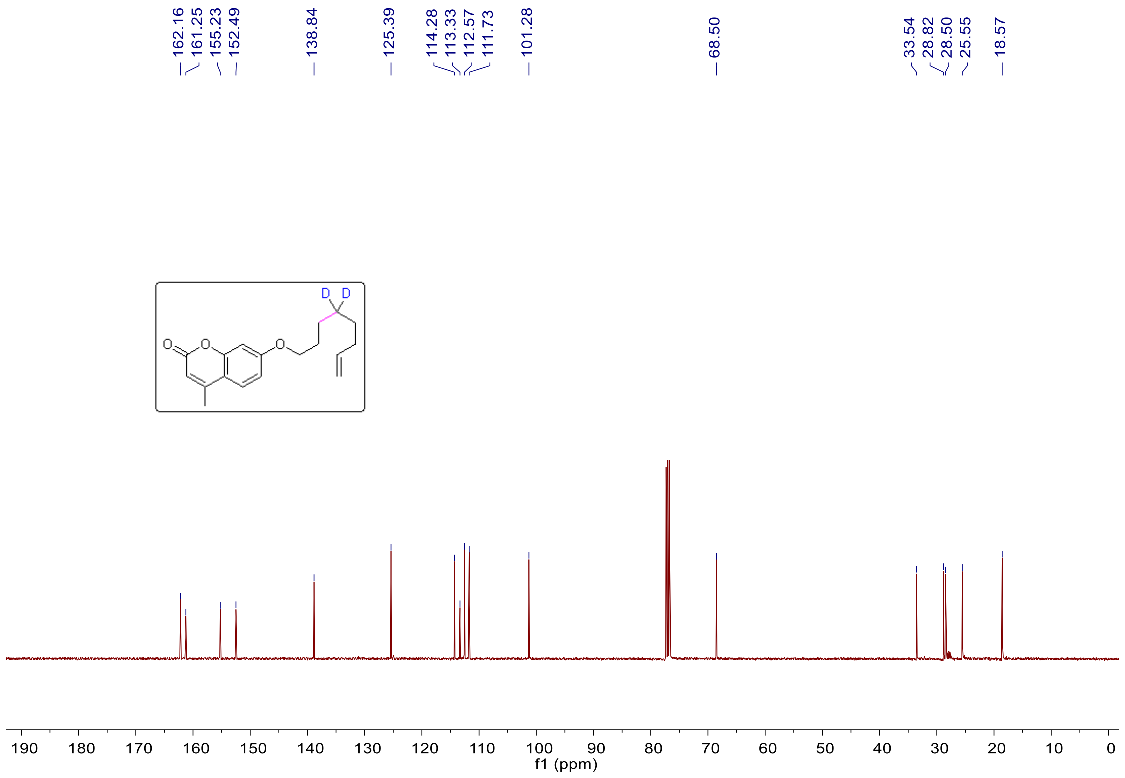

Supplementary Figure 351. ^{13}C NMR (101 MHz, $CDCl_3$) of **7ah**

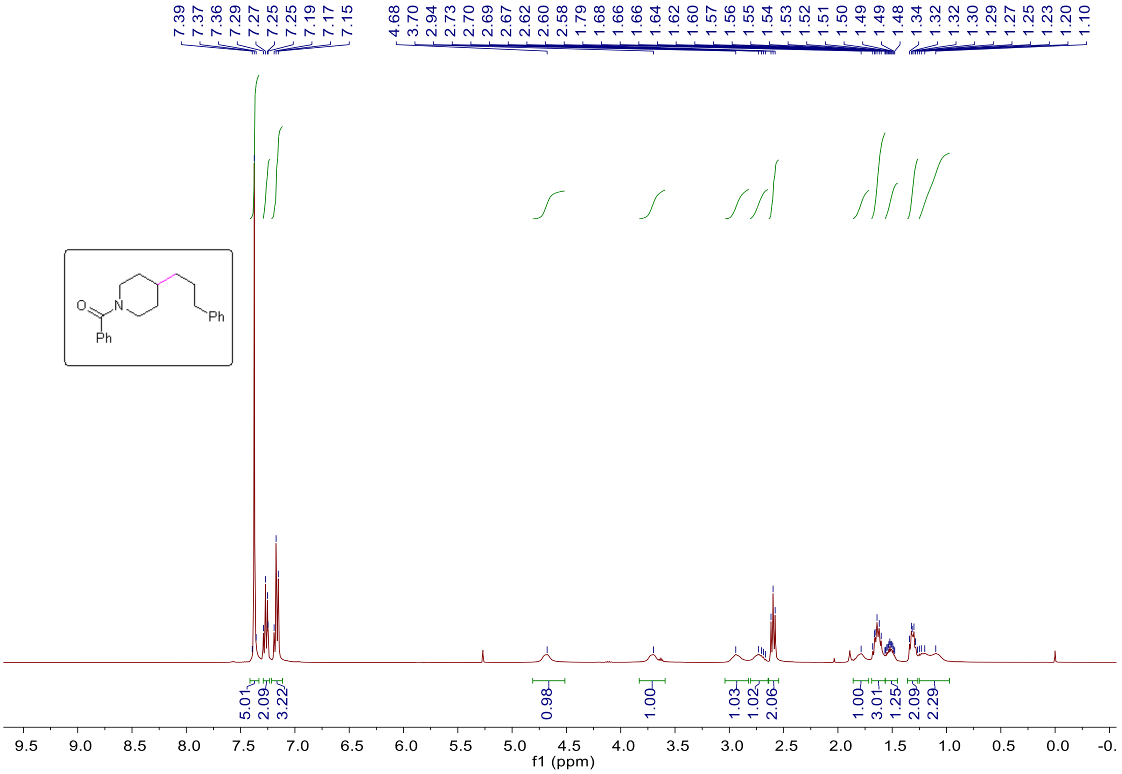

Supplementary Figure 352. ^{1}H NMR (400 MHz, $CDCl_3$) of **7ba'**

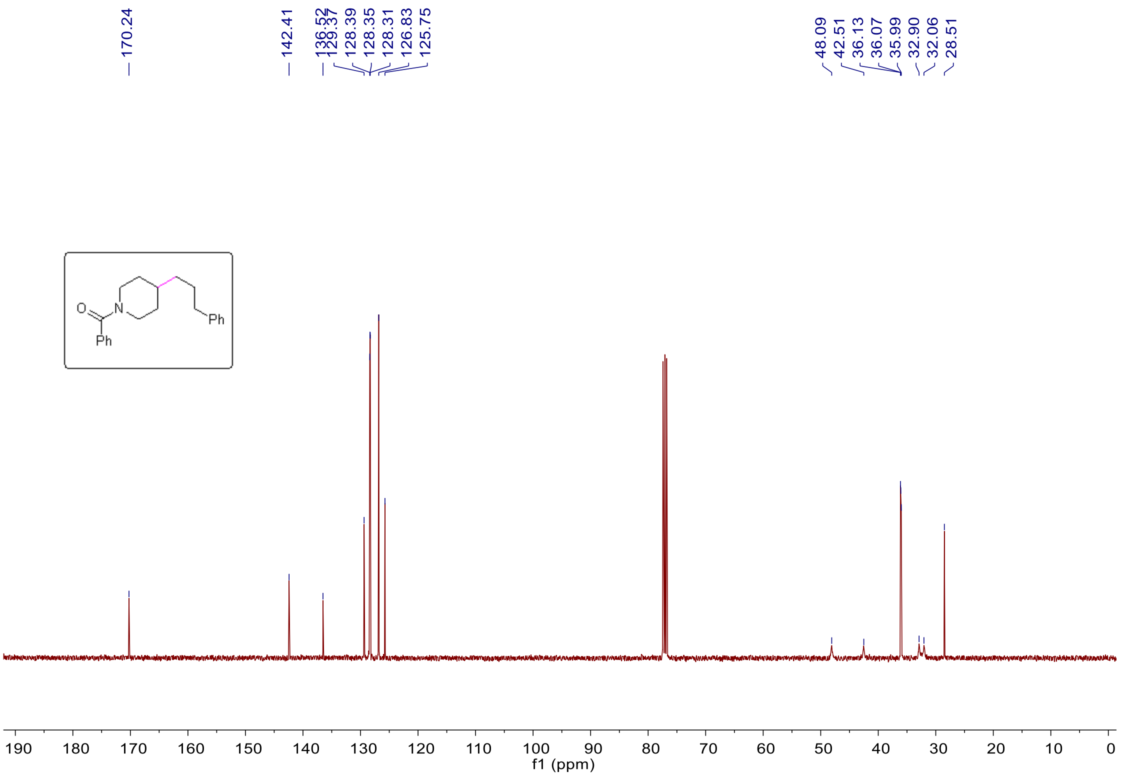

Supplementary Figure 353. ^{13}C NMR (101 MHz, $CDCl_3$) of **7ba'**

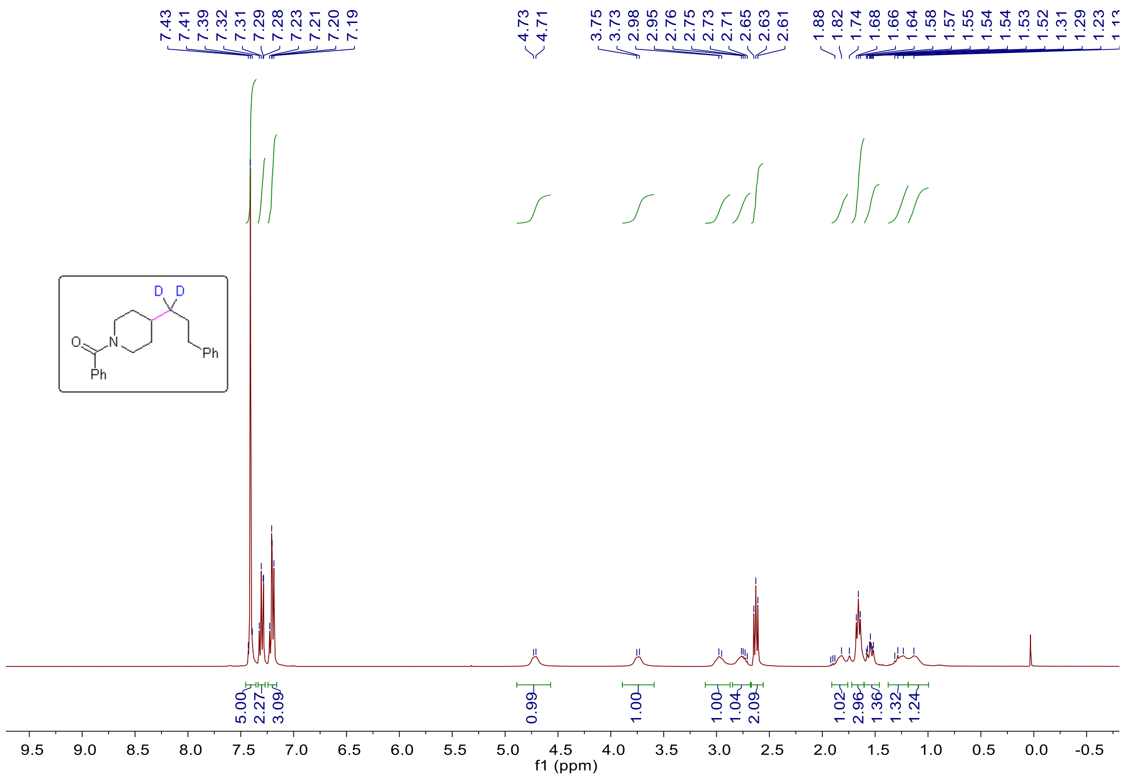

Supplementary Figure 354. ^{1}H NMR (400 MHz, $CDCl_3$) of **7ba**

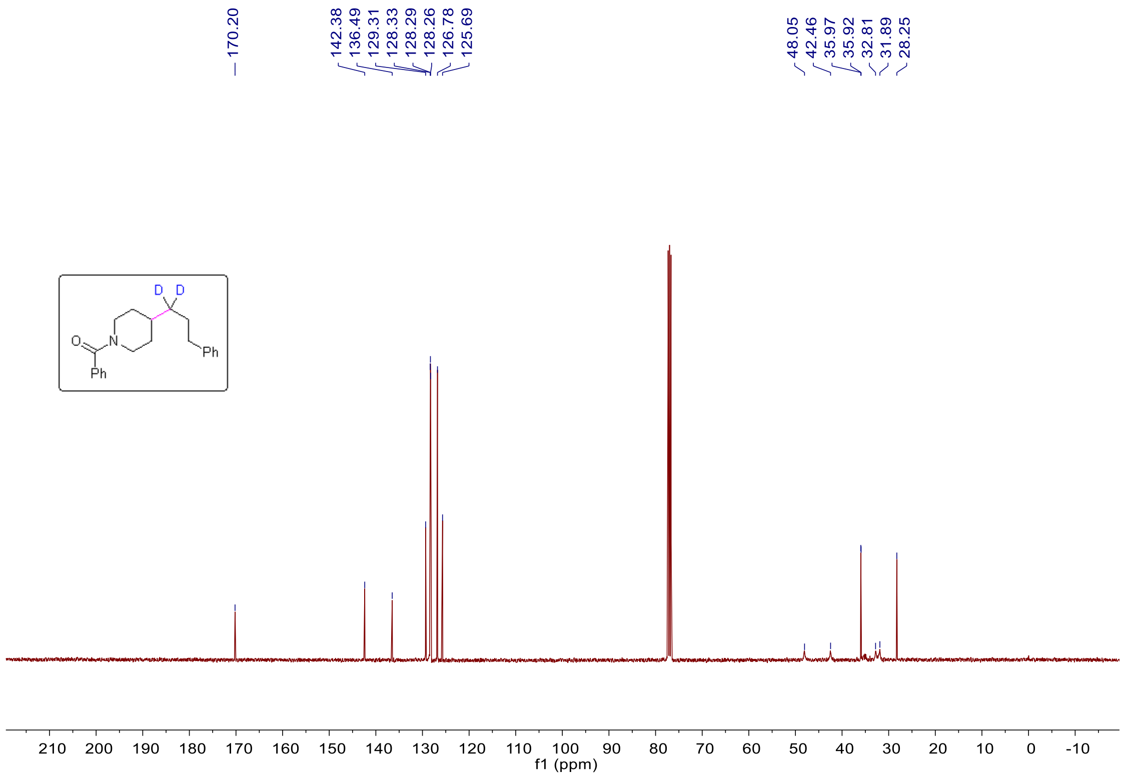

Supplementary Figure 355. ^{13}C NMR (101 MHz, $CDCl_3$) of **7ba**

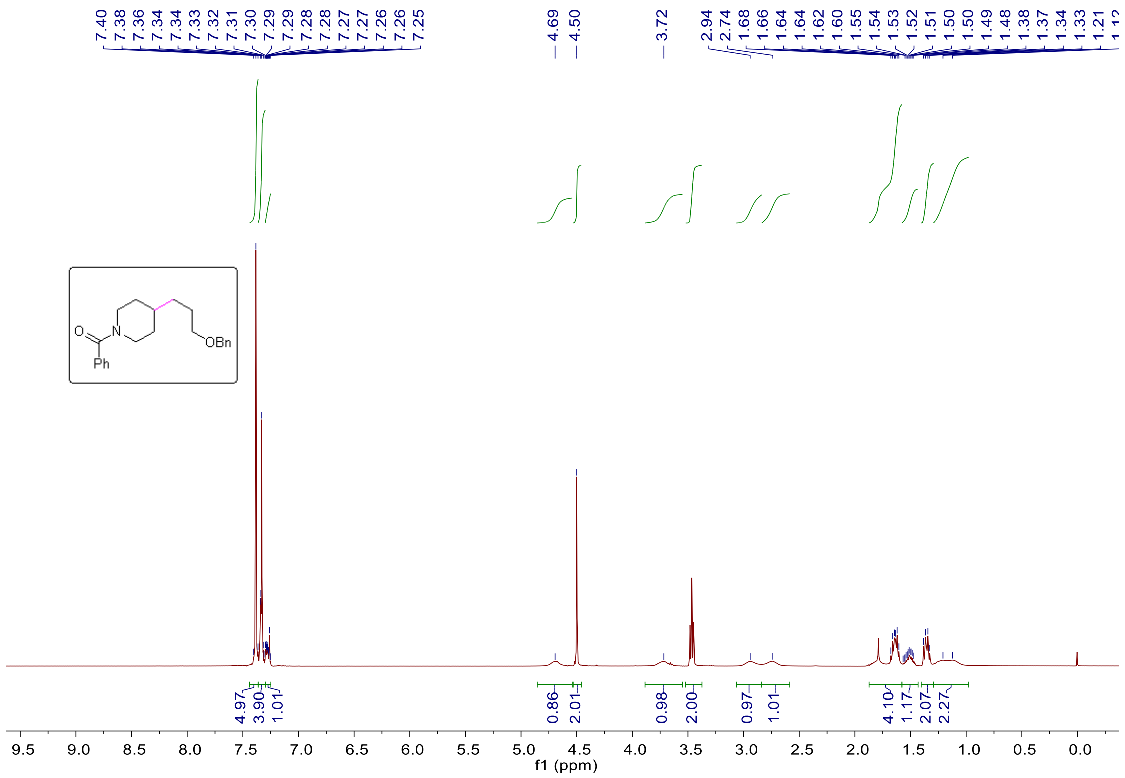

Supplementary Figure 356. ^{1}H NMR (400 MHz, $CDCl_3$) of **7ha'**

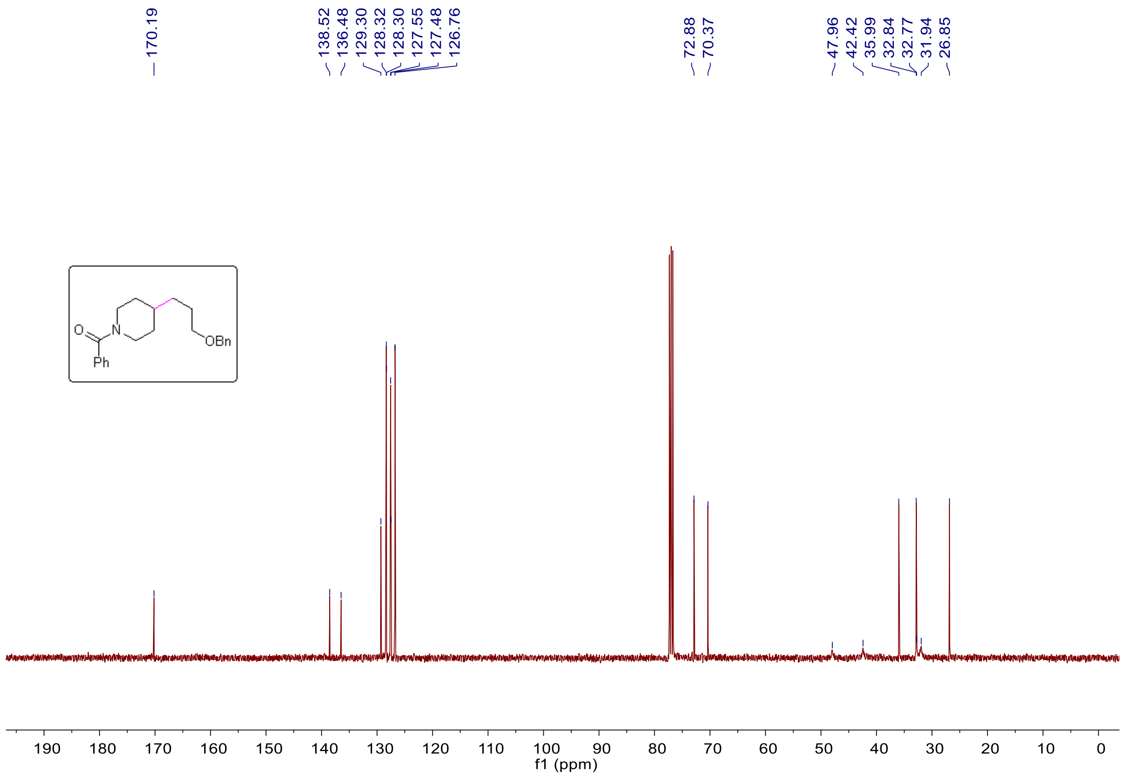

Supplementary Figure 357. ^{13}C NMR (101 MHz, $CDCl_3$) of **7ha'**

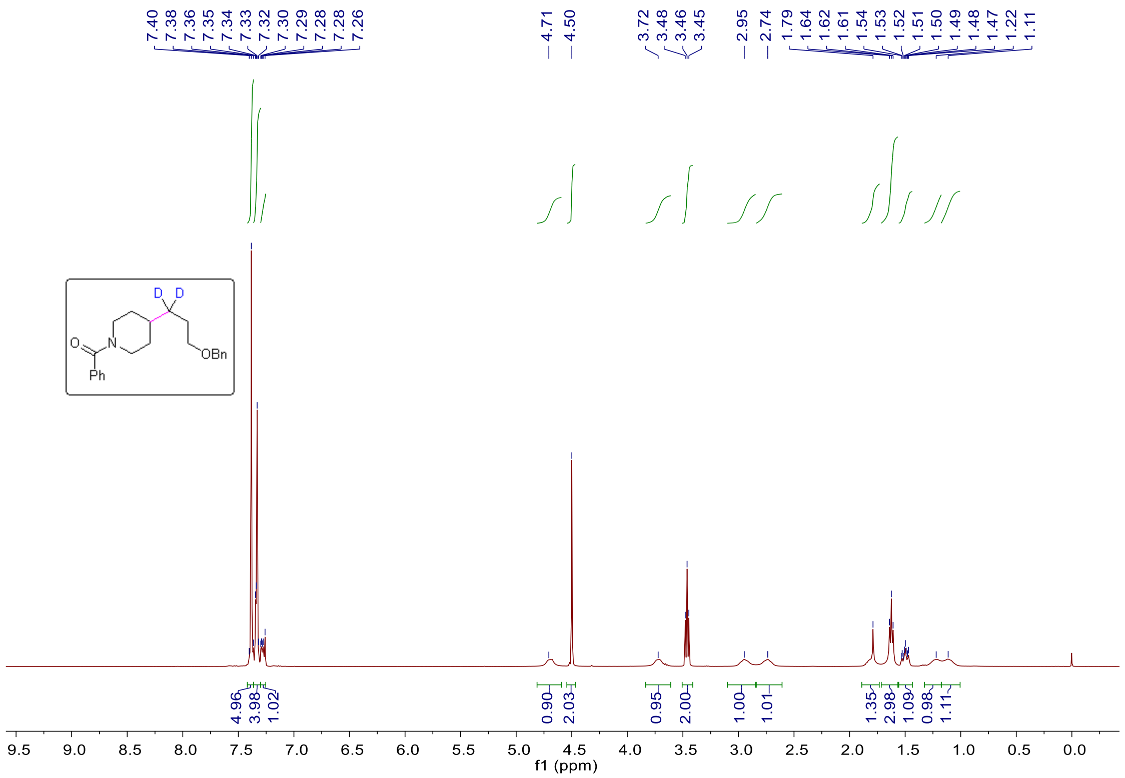

Supplementary Figure 358. ^{1}H NMR (400 MHz, $CDCl_3$) of **7ha**

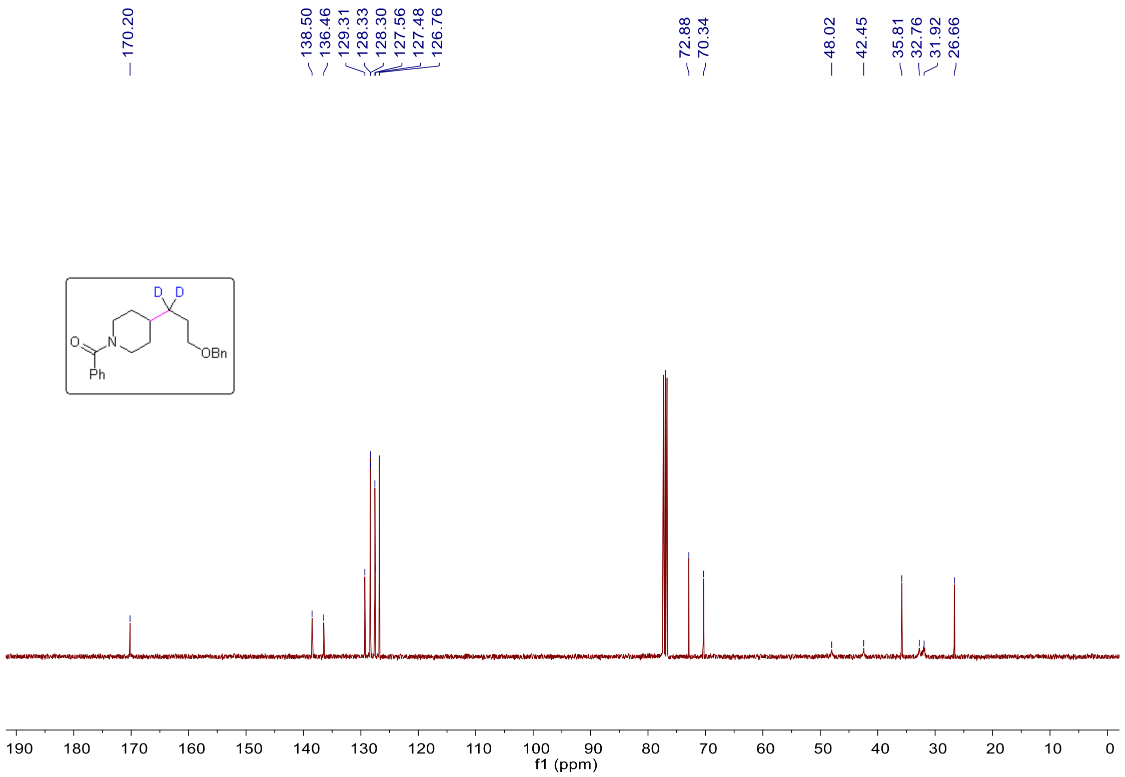

Supplementary Figure 359. ^{13}C NMR (101 MHz, $CDCl_3$) of **7ha**

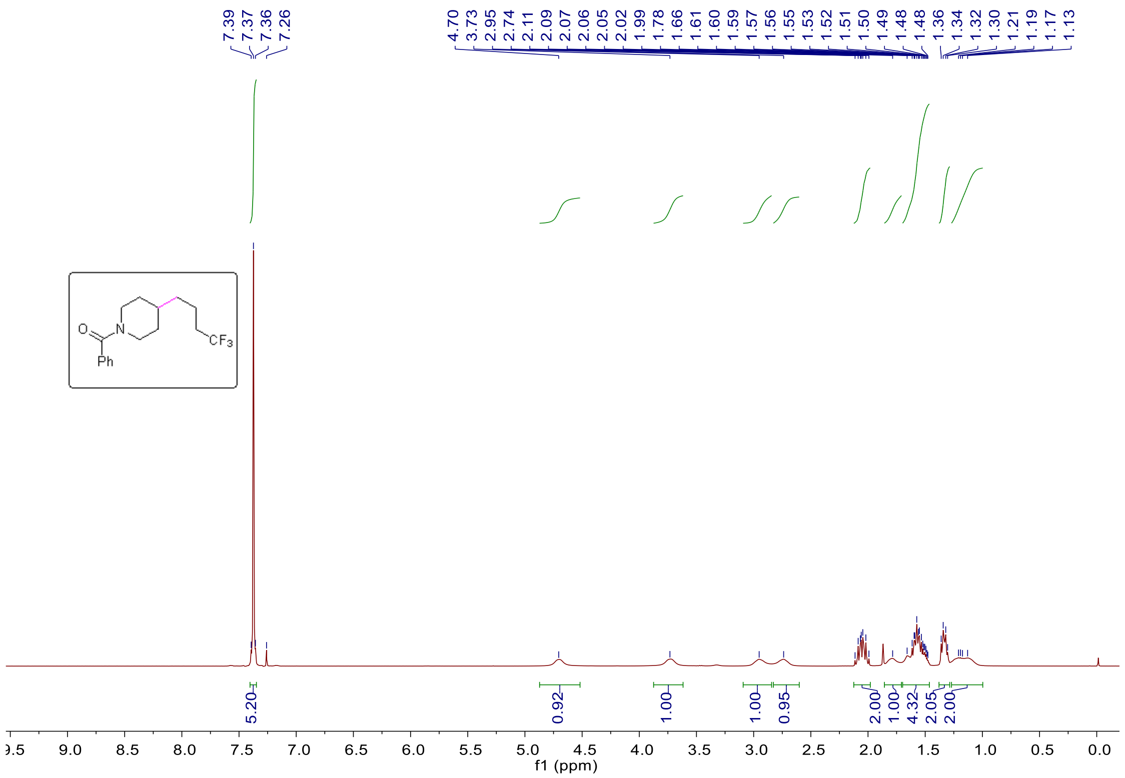

Supplementary Figure 360. ^{1}H NMR (400 MHz, $CDCl_3$) of **7ia'**

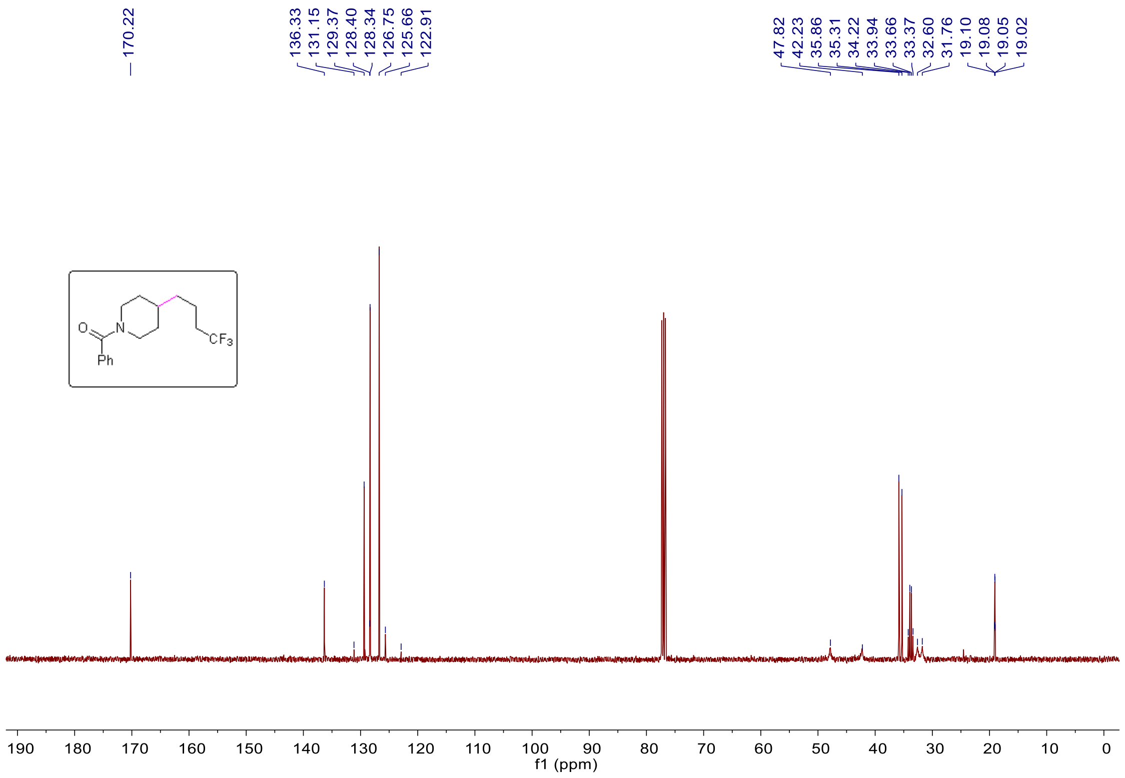

Supplementary Figure 361. ^{13}C NMR (101 MHz, $CDCl_3$) of **7ia'**

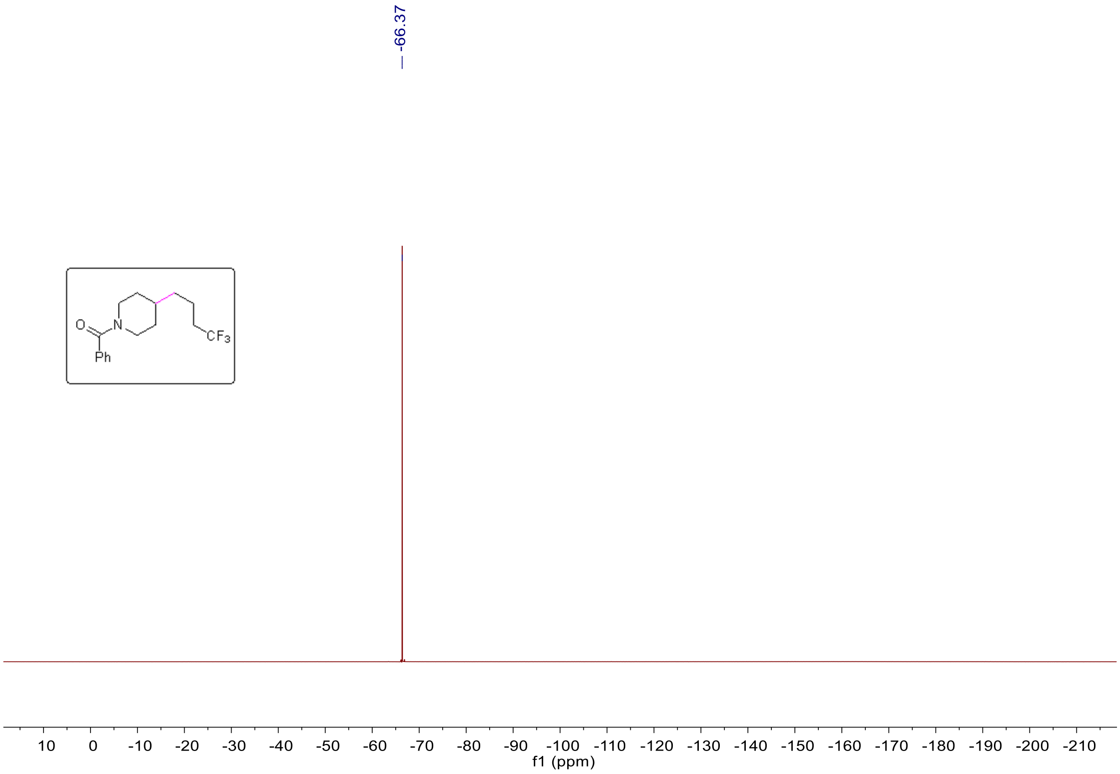

Supplementary Figure 362. ^{19}F NMR (376 MHz, $CDCl_3$) of **7ia'**

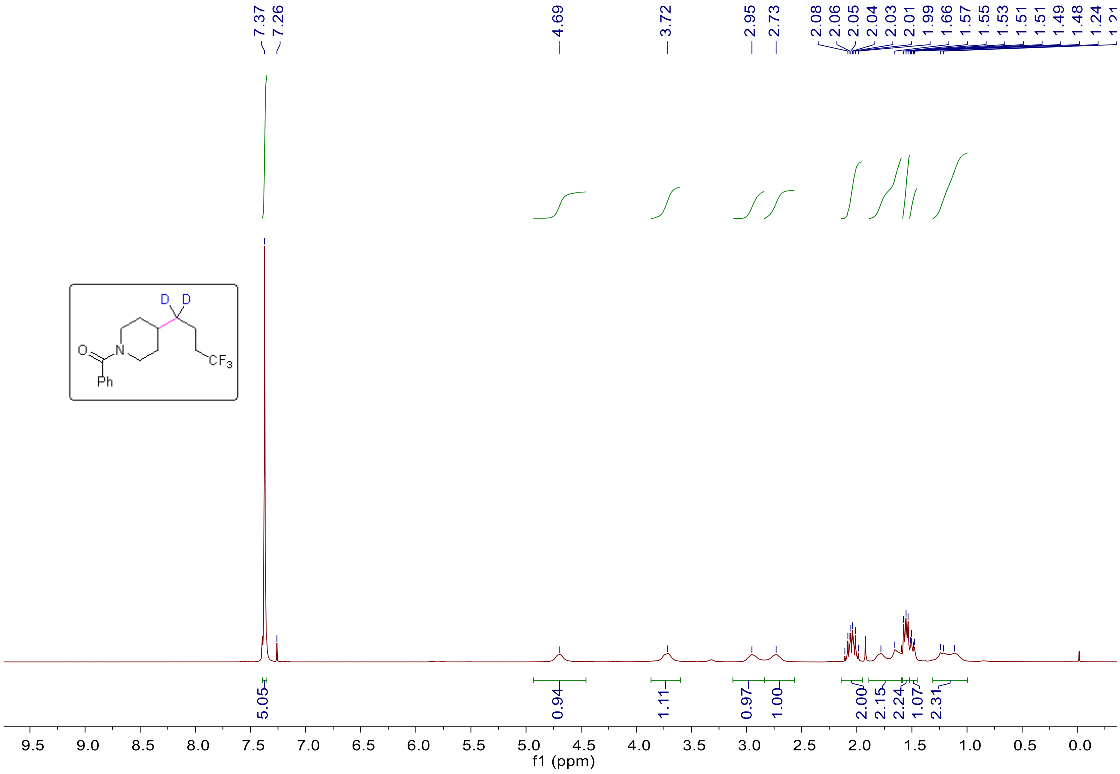

Supplementary Figure 363. ^{1}H NMR (400 MHz, $CDCl_3$) of **7ia**

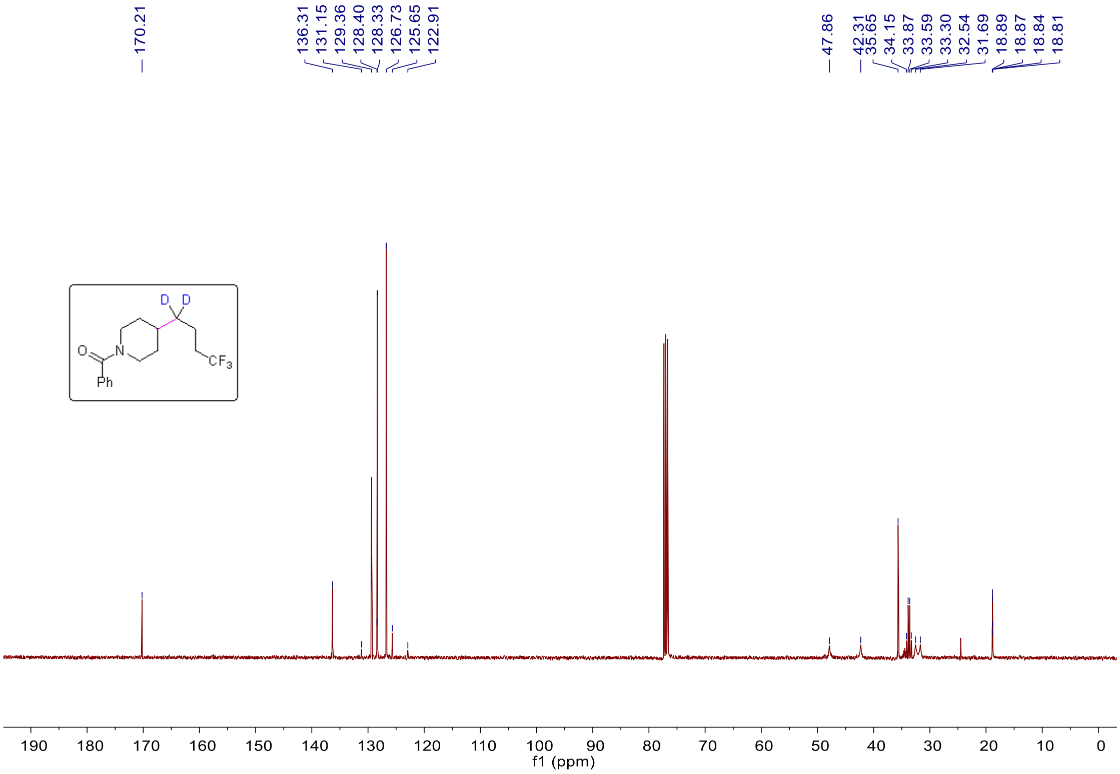

Supplementary Figure 364. ^{13}C NMR (101 MHz, $CDCl_3$) of **7ia**

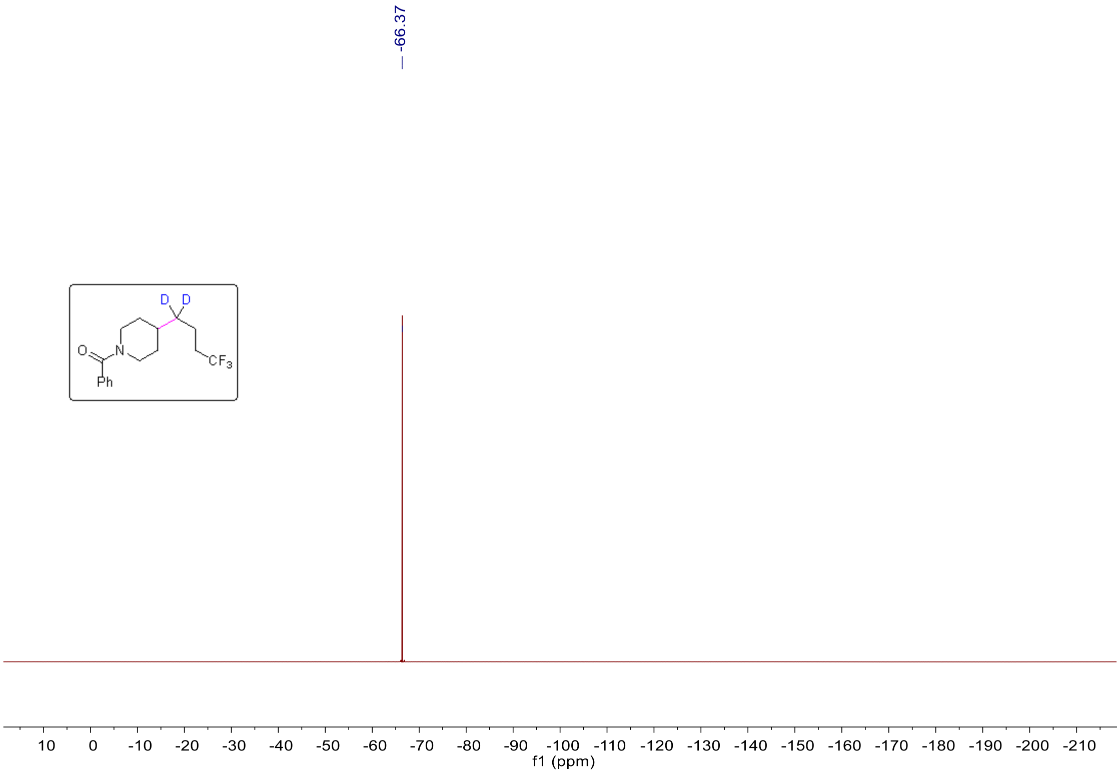

Supplementary Figure 365. ^{19}F NMR (376 MHz, $CDCl_3$) of **7ia**

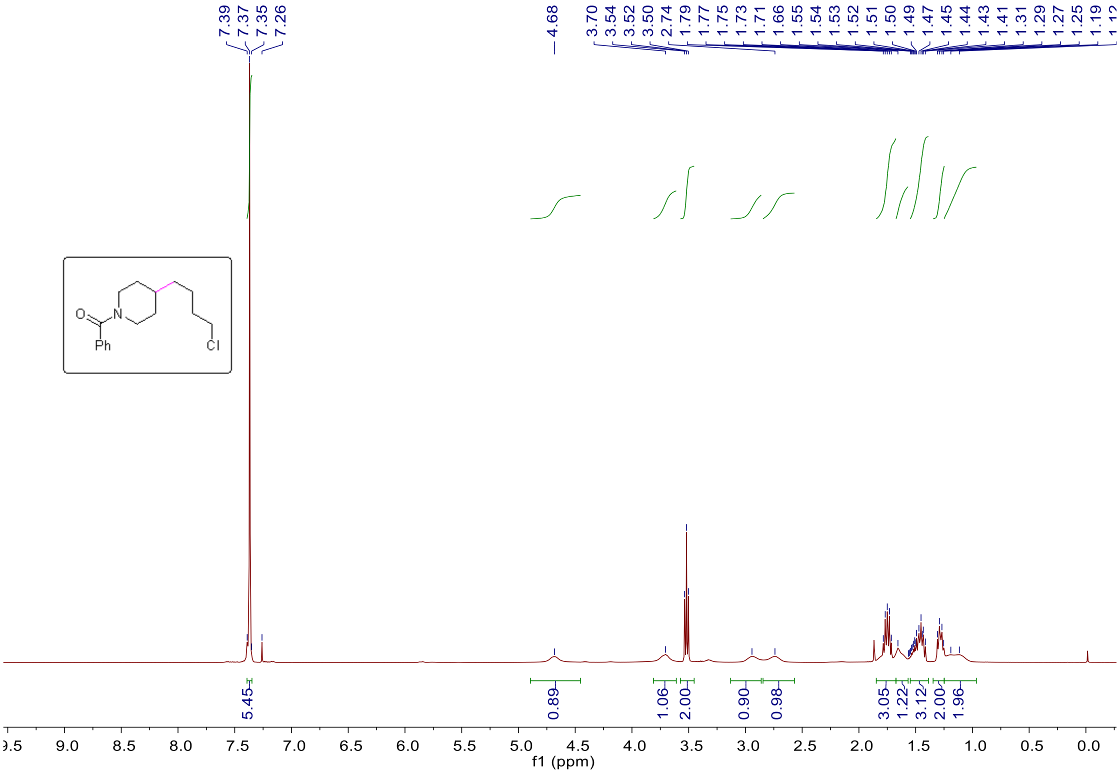

Supplementary Figure 366. ^{1}H NMR (400 MHz, $CDCl_3$) of **7ja'**

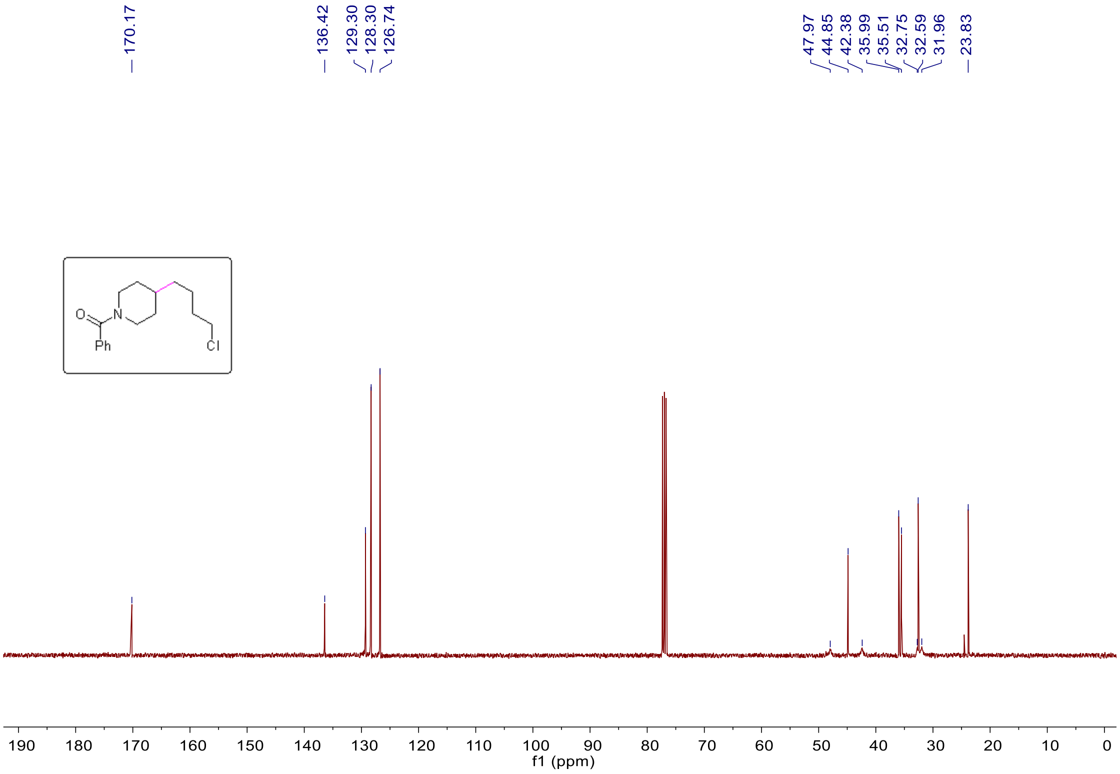

Supplementary Figure 367. ^{13}C NMR (101 MHz, $CDCl_3$) of **7ja'**

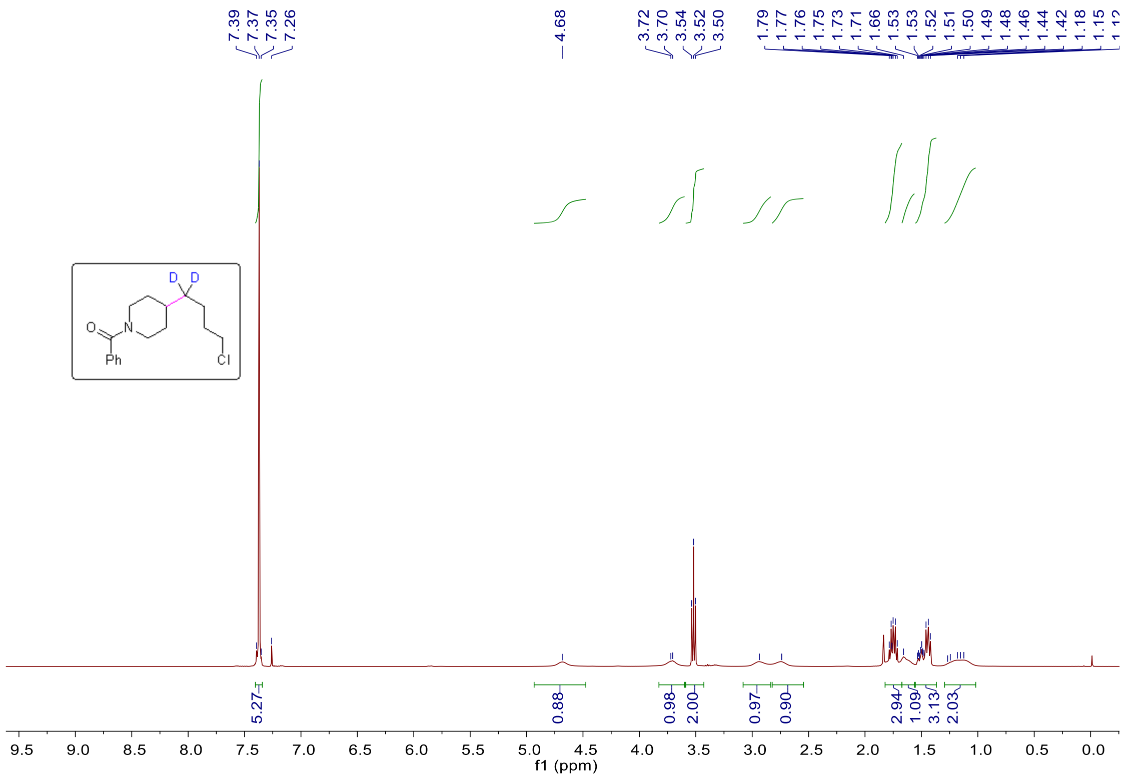

Supplementary Figure 368. ^{1}H NMR (400 MHz, $CDCl_3$) of **7ja**

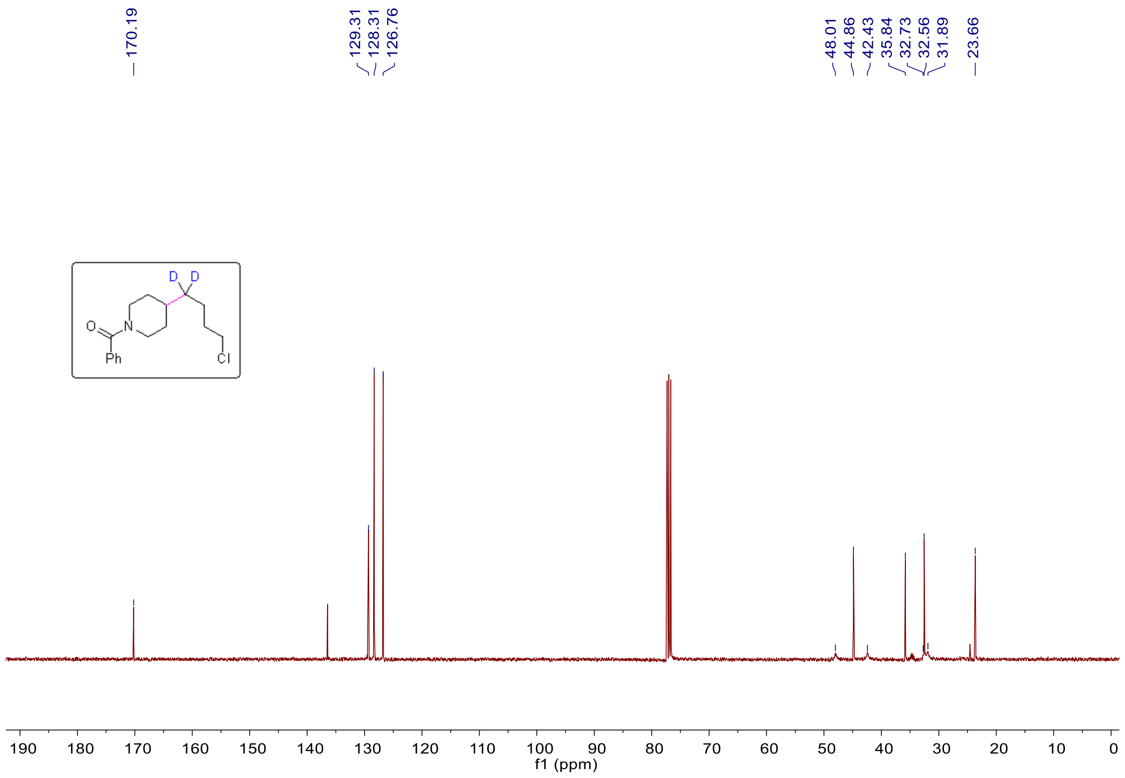

Supplementary Figure 369. ^{13}C NMR (101 MHz, $CDCl_3$) of **7ja**

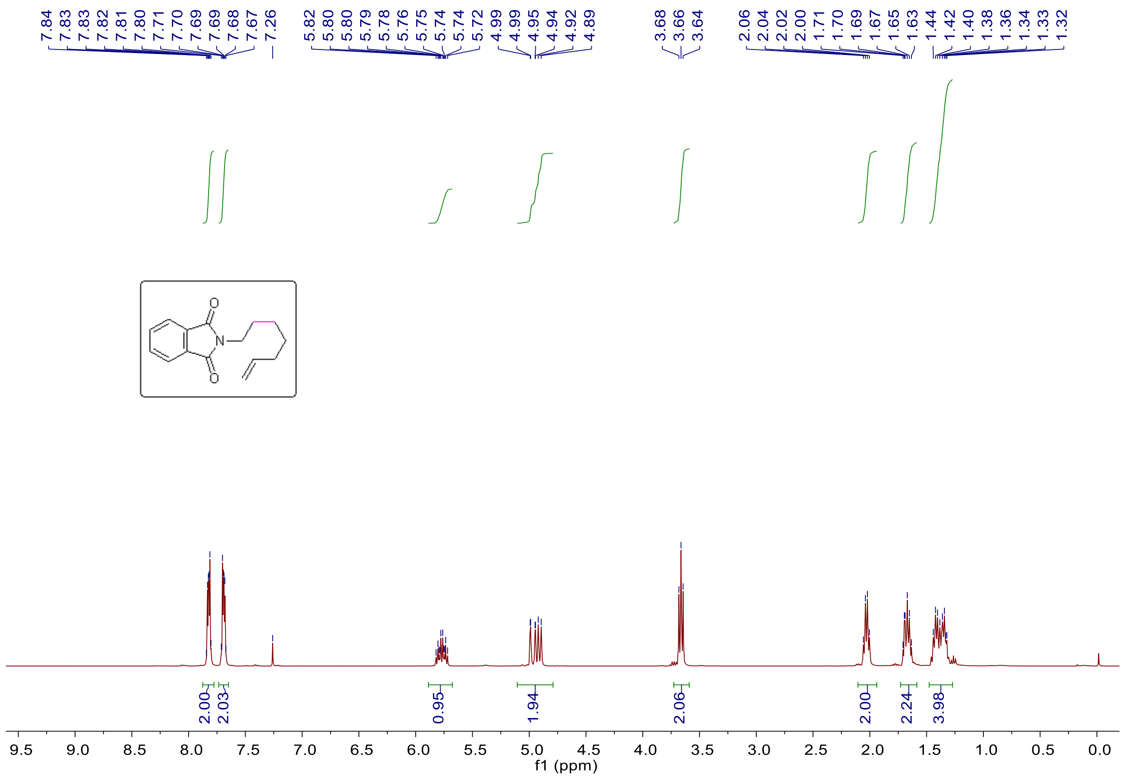

Supplementary Figure 370. ^{1}H NMR (400 MHz, $CDCl_3$) of **8**

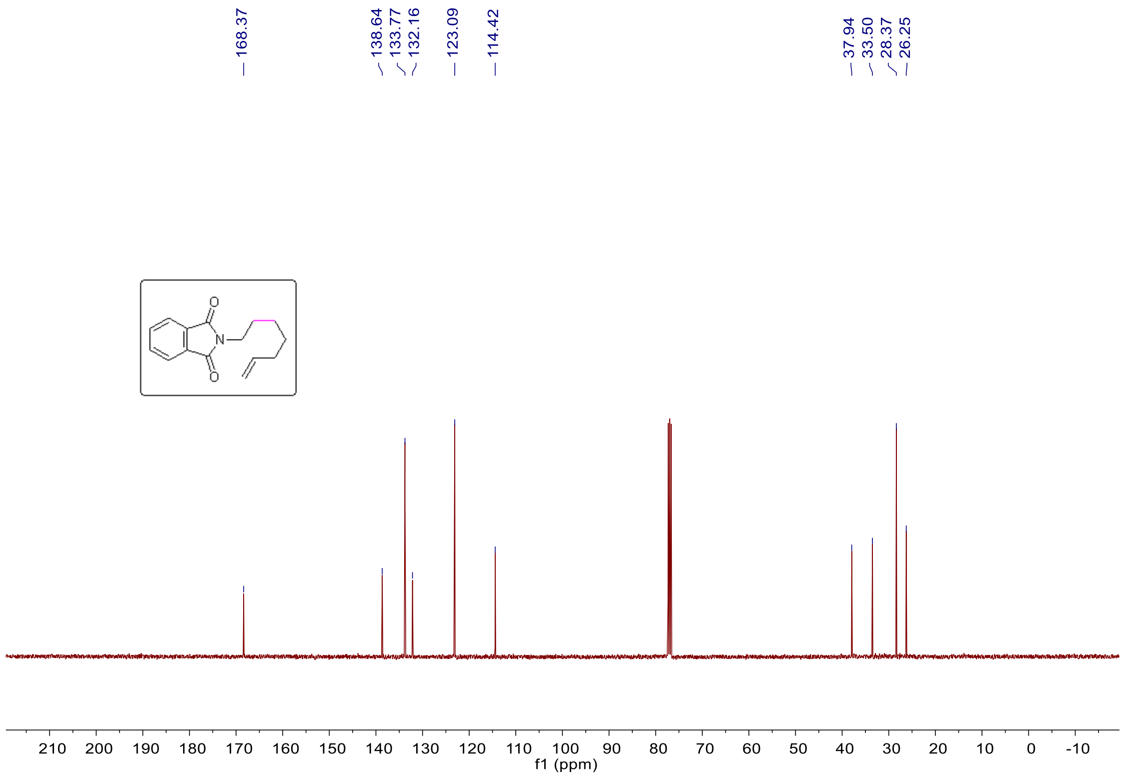

Supplementary Figure 371. ^{13}C NMR (101 MHz, $CDCl_3$) of **8**

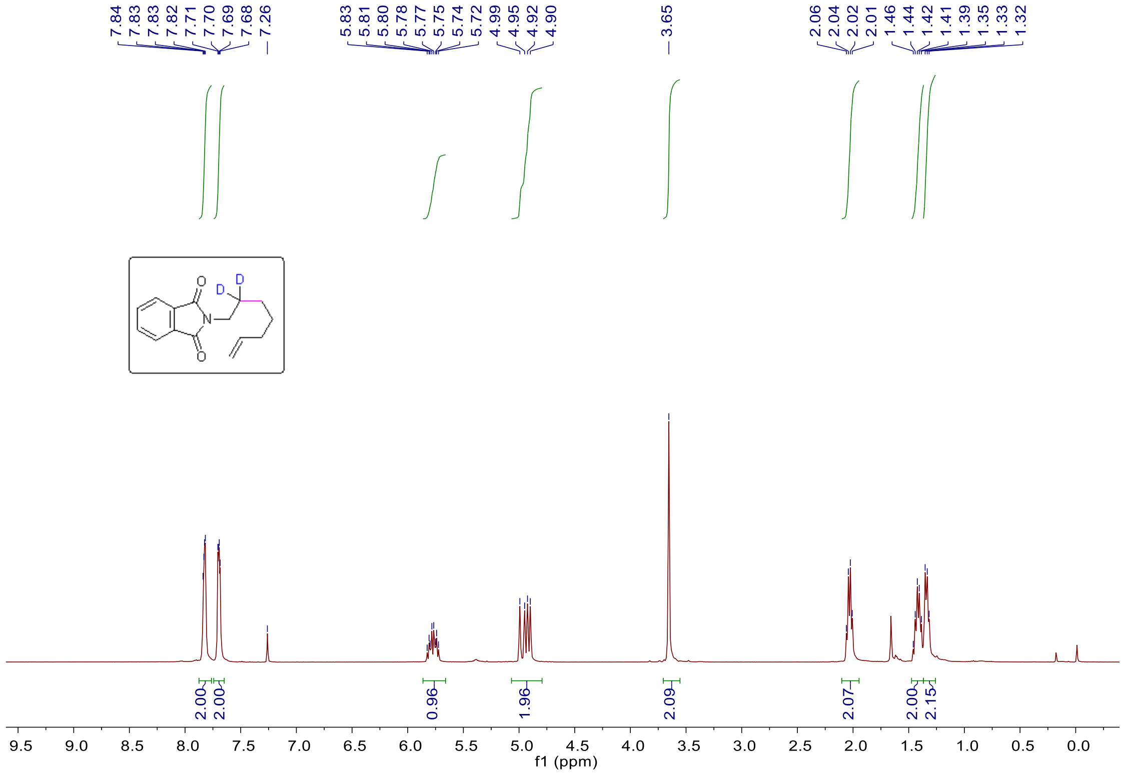

Supplementary Figure 372. ^{1}H NMR (400 MHz, $CDCl_3$) of **9**

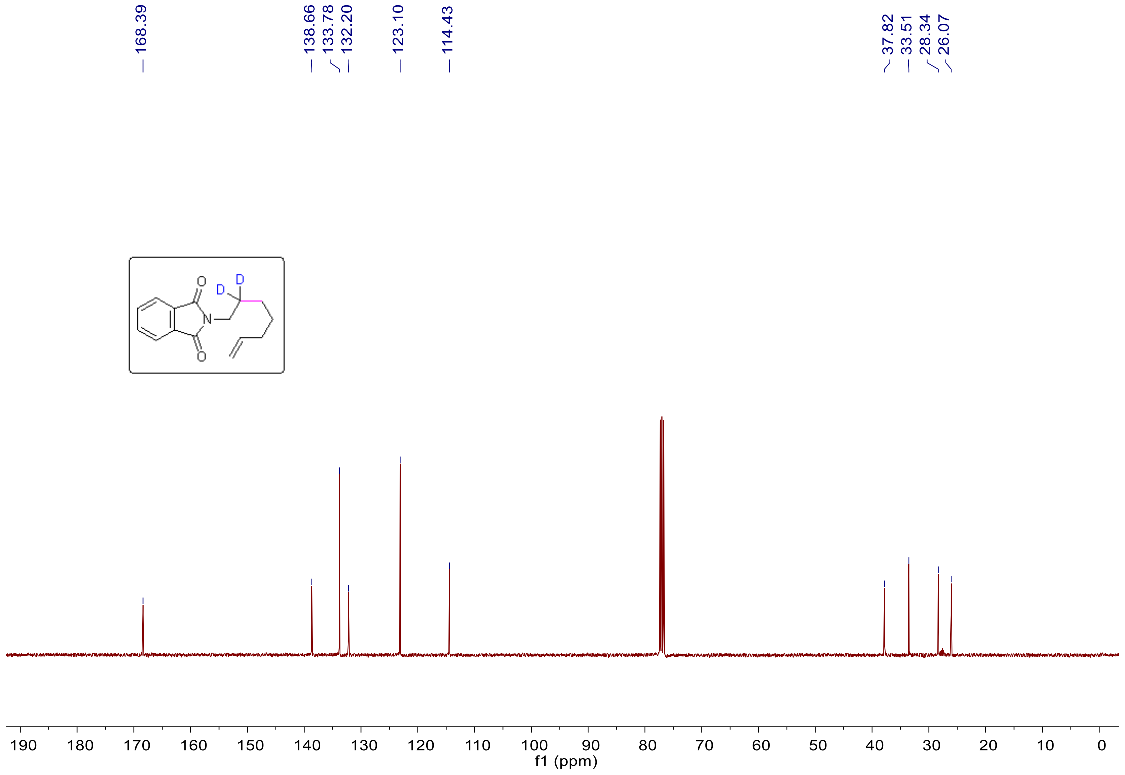

Supplementary Figure 373. ^{13}C NMR (101 MHz, $CDCl_3$) of **9**

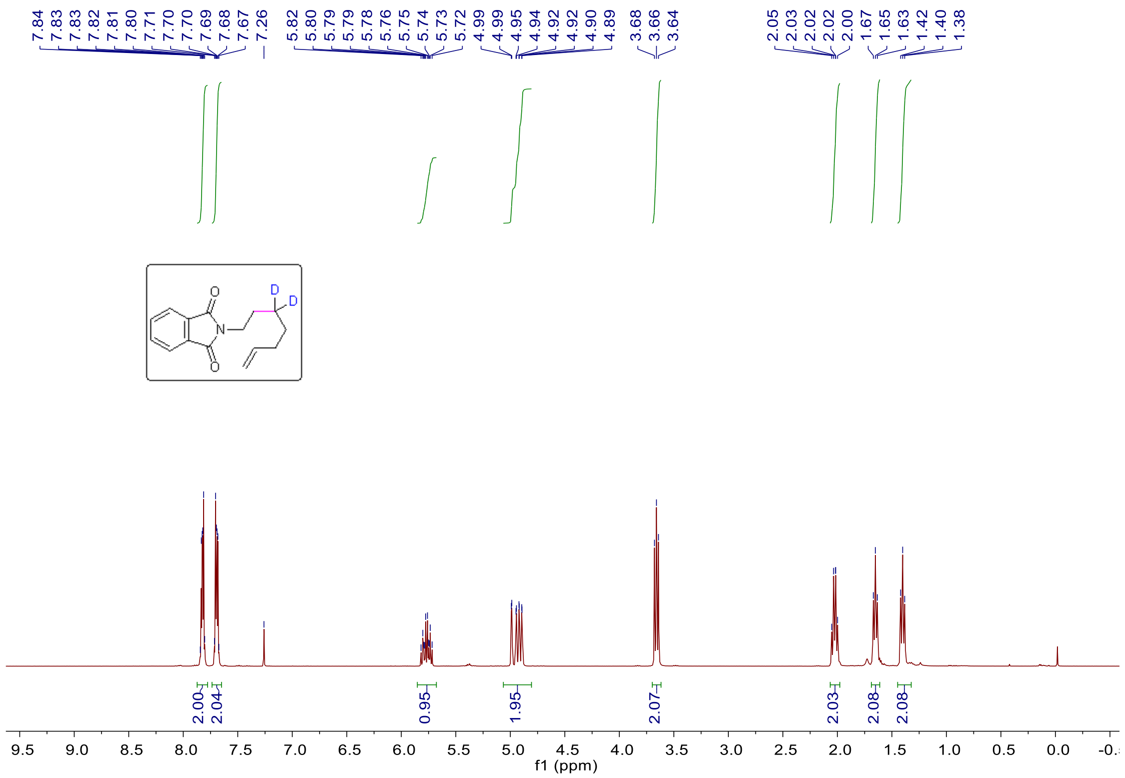

Supplementary Figure 374. ^{1}H NMR (400 MHz, $CDCl_3$) of **10**

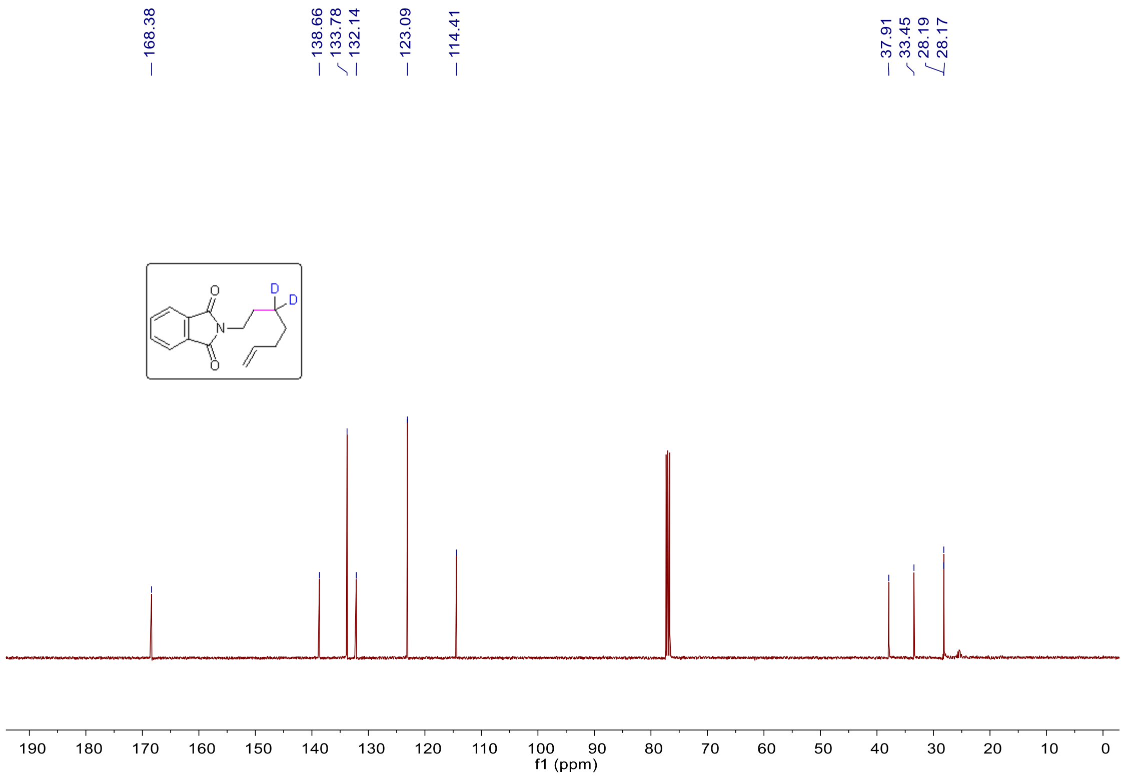

Supplementary Figure 375. ^{13}C NMR (101 MHz, $CDCl_3$) of **10**

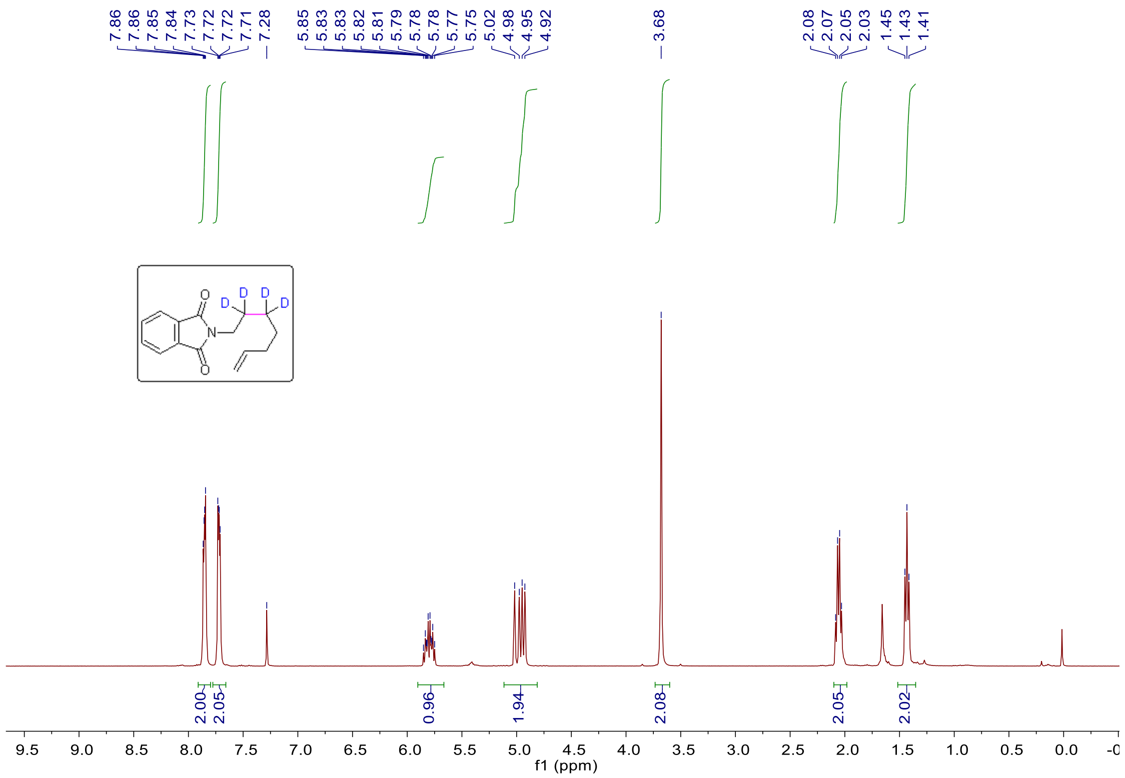

Supplementary Figure 376. ^{1}H NMR (400 MHz, $CDCl_3$) of **11**

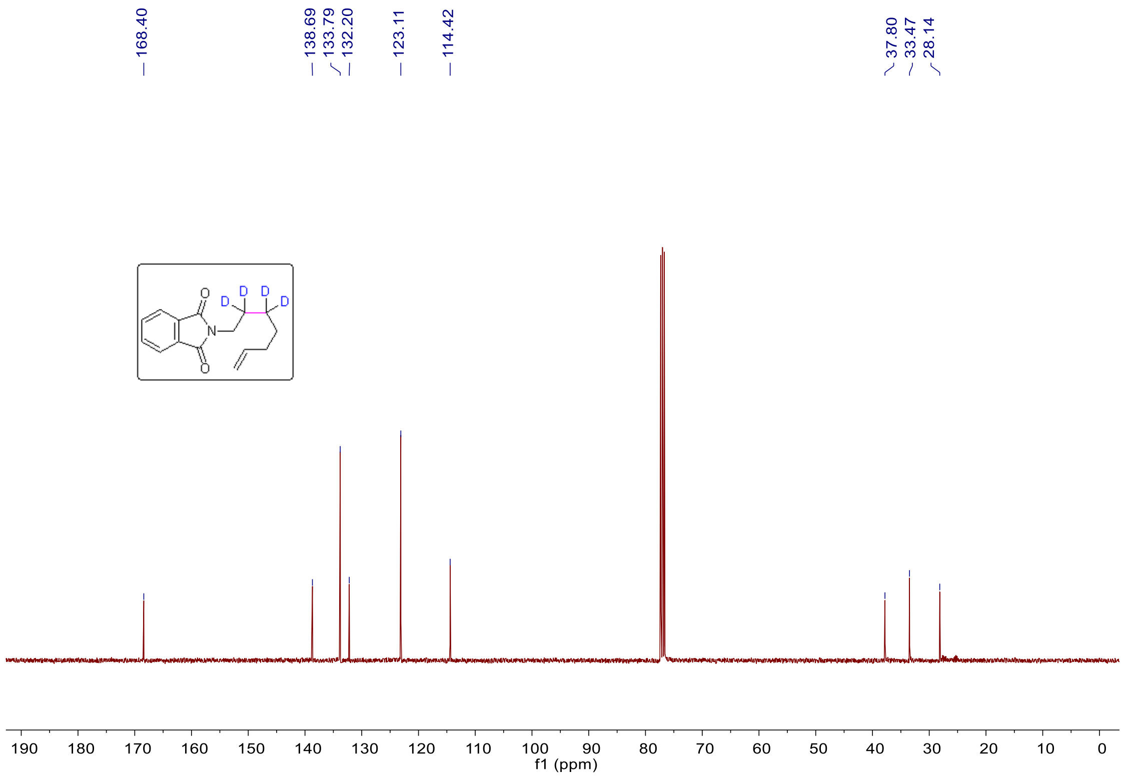

Supplementary Figure 377. ^{13}C NMR (101 MHz, $CDCl_3$) of **11**

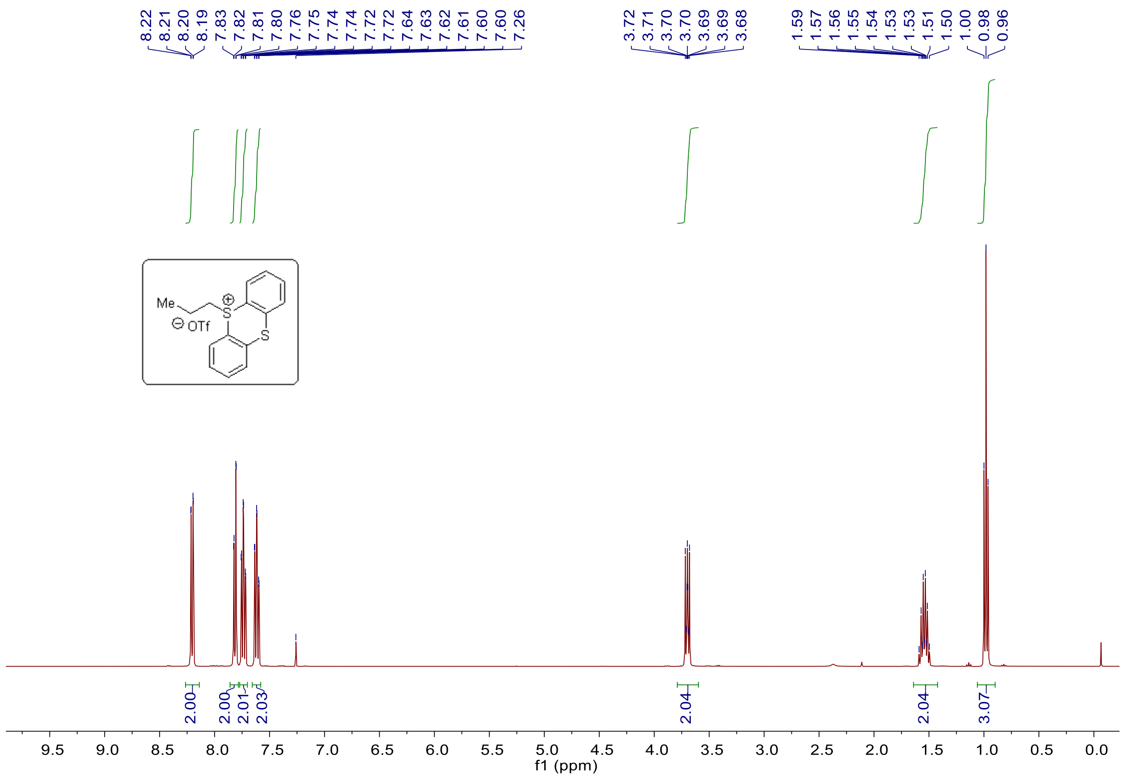

Supplementary Figure 378. ^{1}H NMR (400 MHz, $CDCl_3$) of **12'**

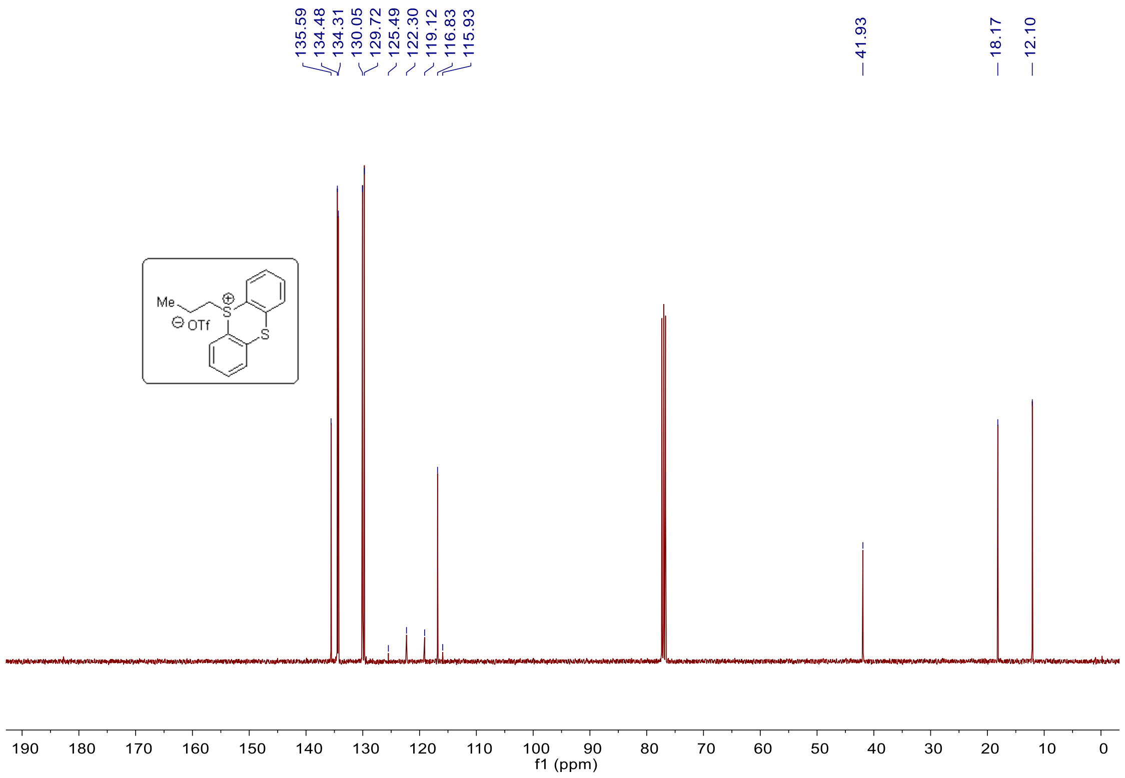

Supplementary Figure 379. ^{13}C NMR (101 MHz, $CDCl_3$) of **12'**

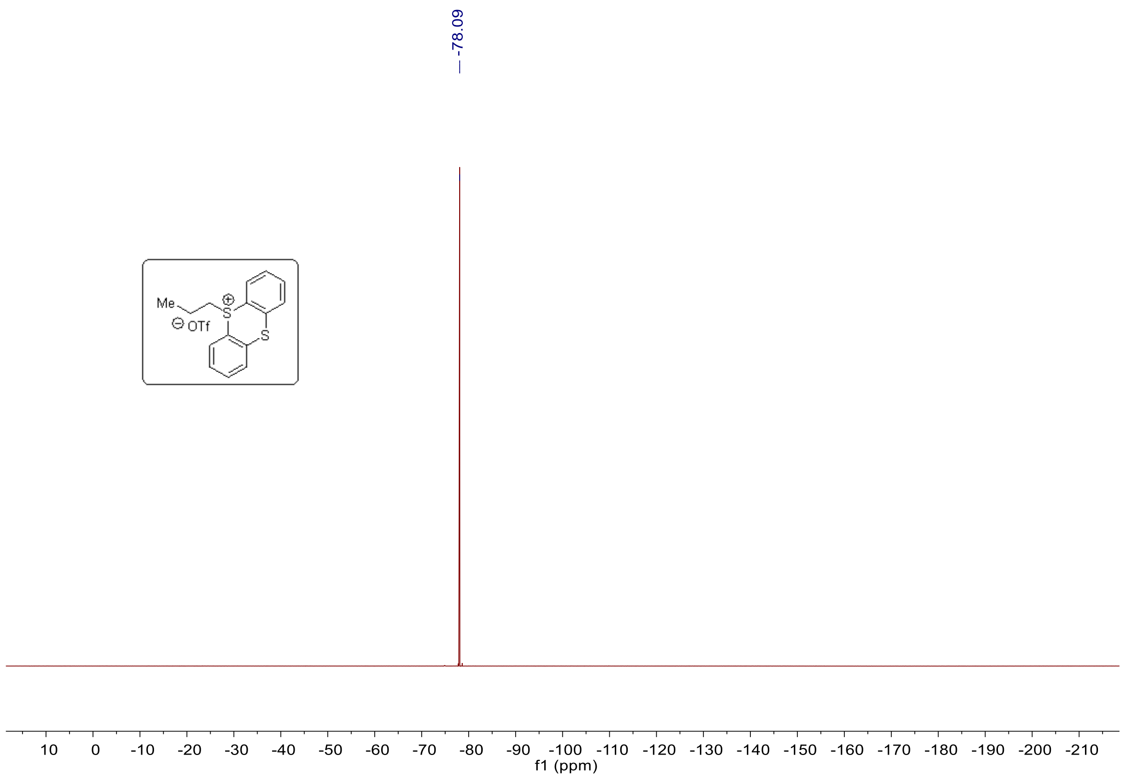

Supplementary Figure 380. ^{19}F NMR (376 MHz, $CDCl_3$) of **12'**

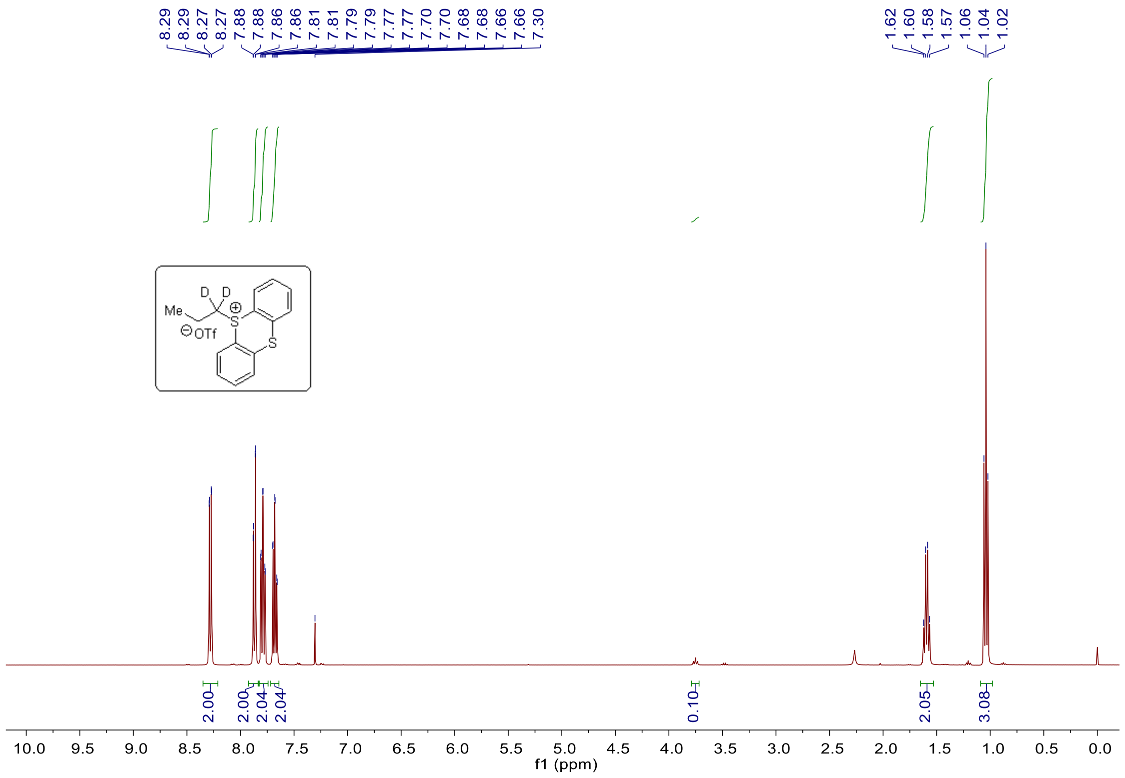

Supplementary Figure 381. ^{1}H NMR (400 MHz, $CDCl_3$) of **12**

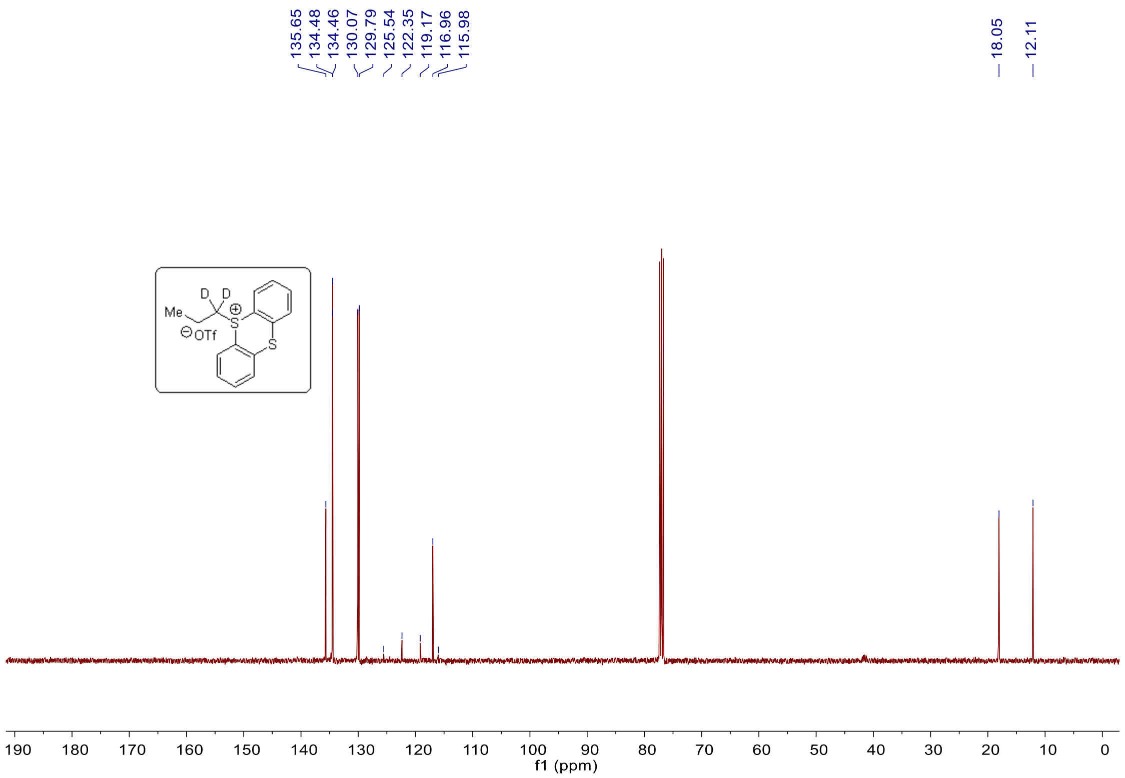

Supplementary Figure 382. ^{13}C NMR (101 MHz, $CDCl_3$) of **12**

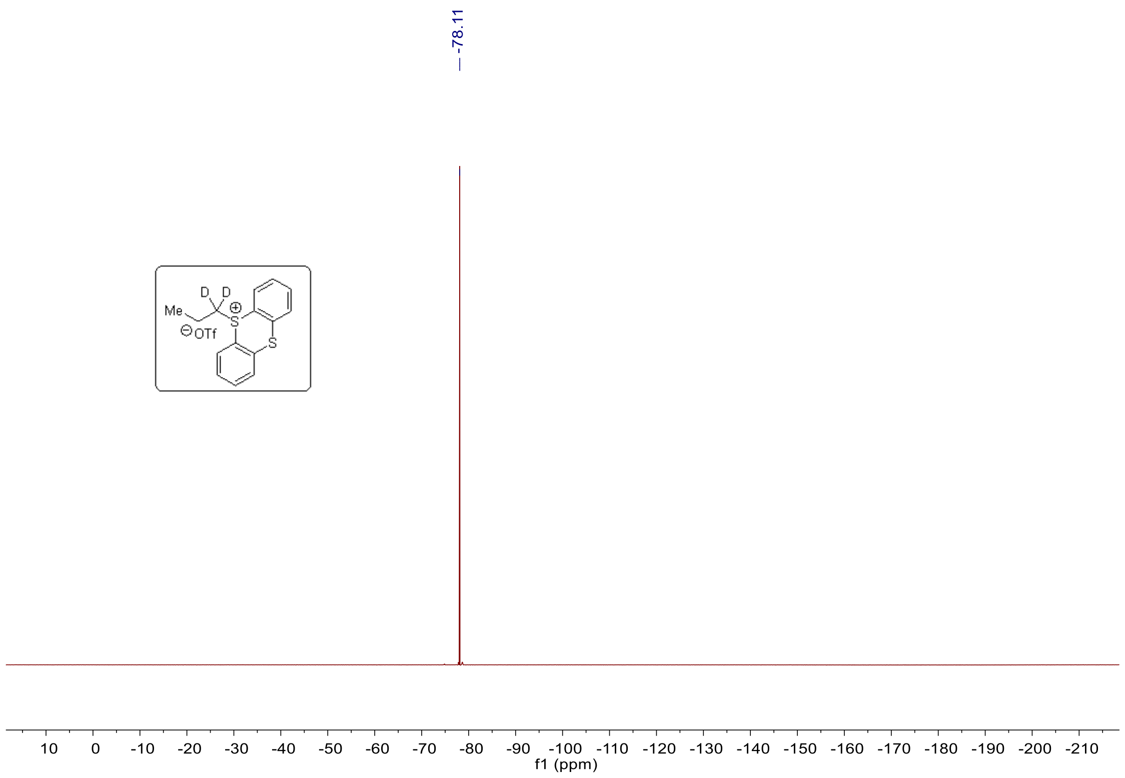

Supplementary Figure 383. ^{19}F NMR (376 MHz, $CDCl_3$) of **12**

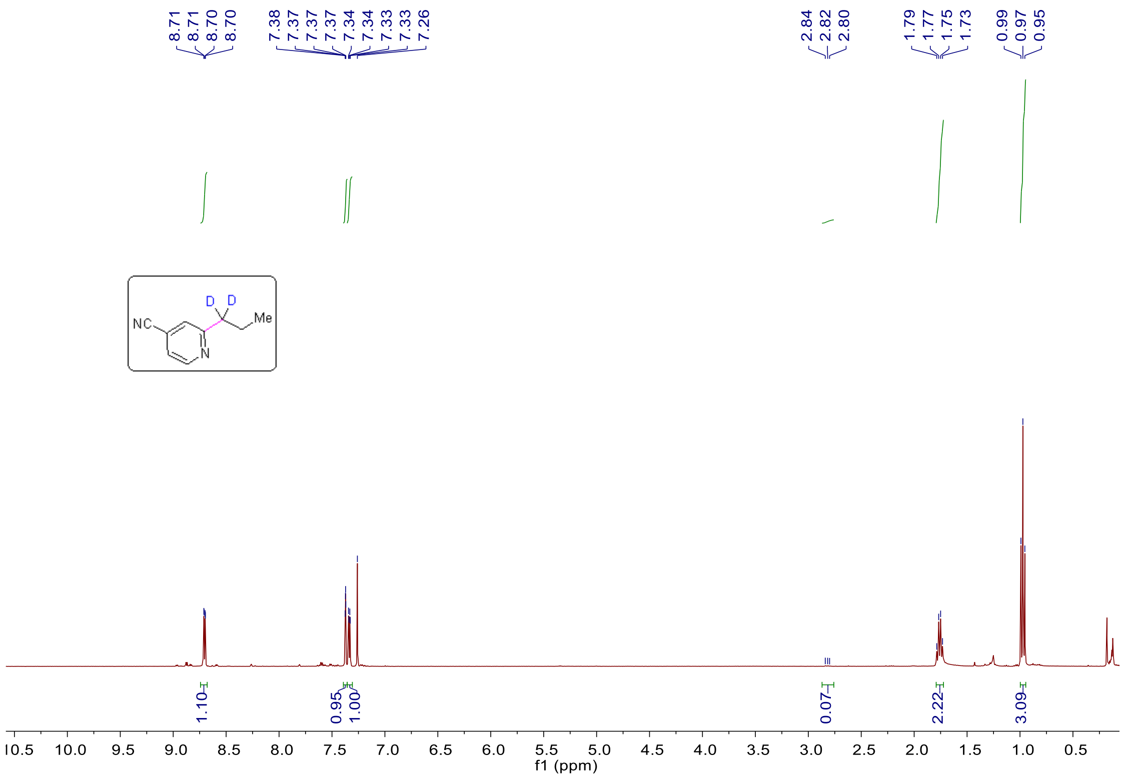

Supplementary Figure 384. ^{1}H NMR (400 MHz, $CDCl_3$) of **14**

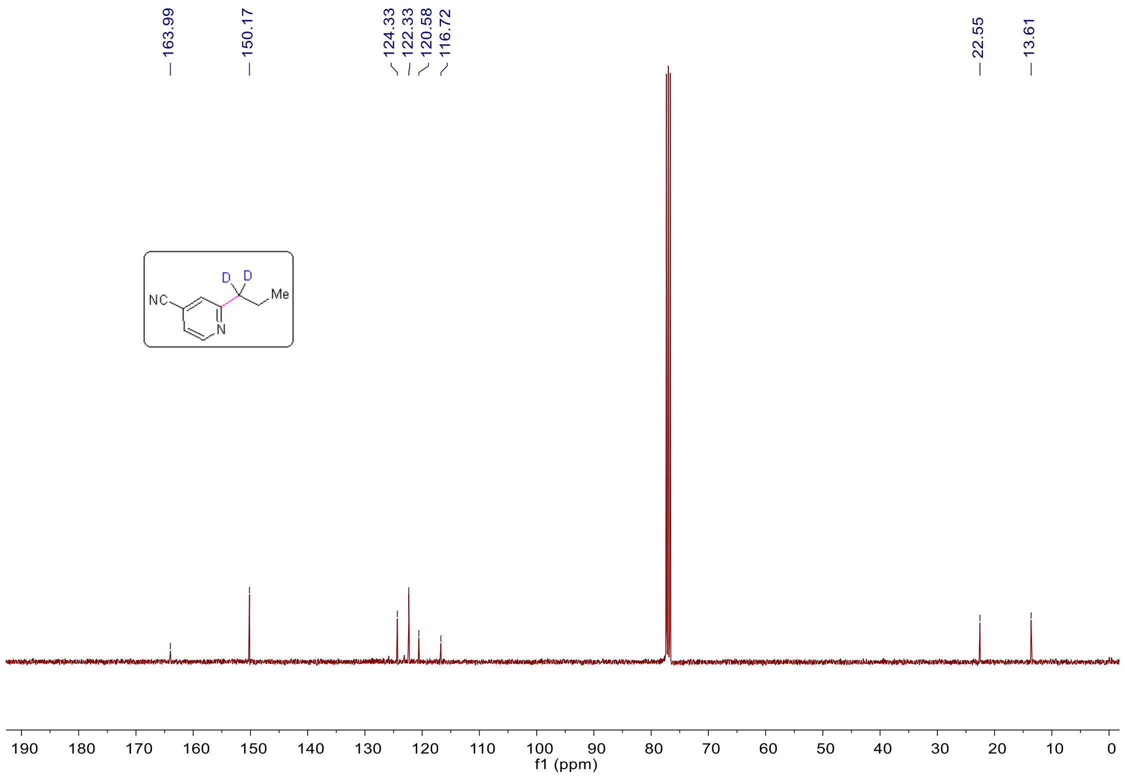

Supplementary Figure 385. ^{13}C NMR (101 MHz, $CDCl_3$) of **14**

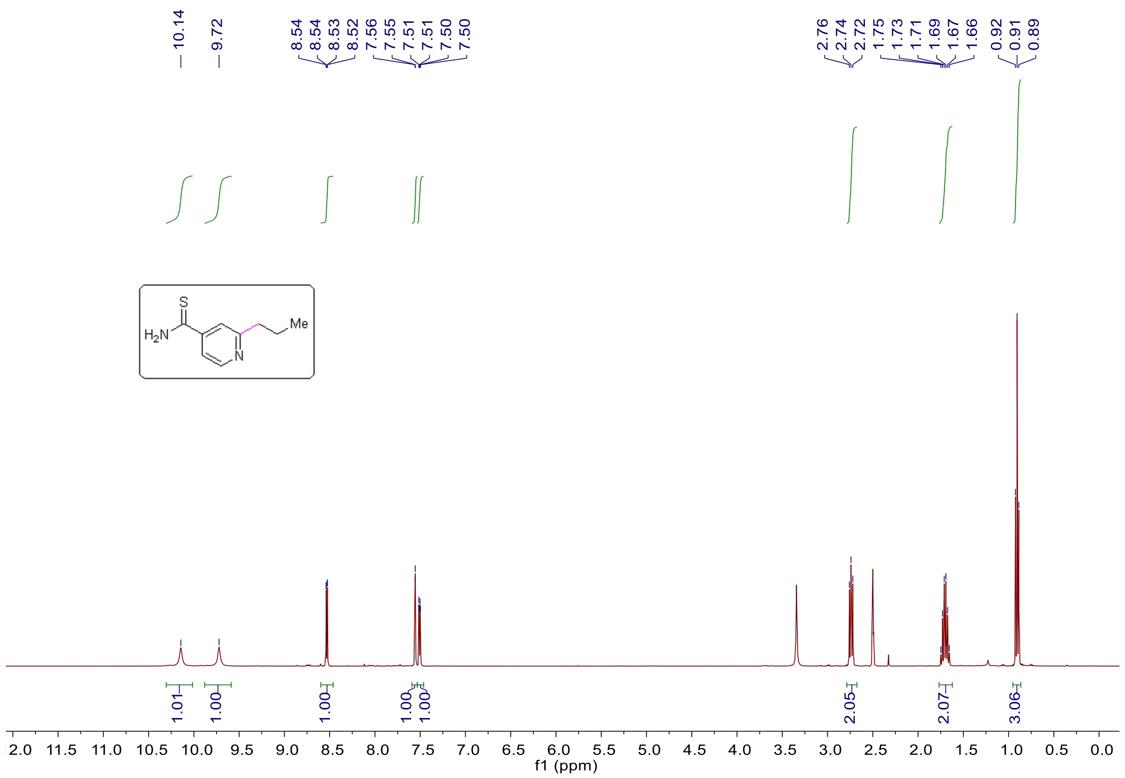

Supplementary Figure 386. ^{1}H NMR (400 MHz, DMSO-d_6) of **15'**

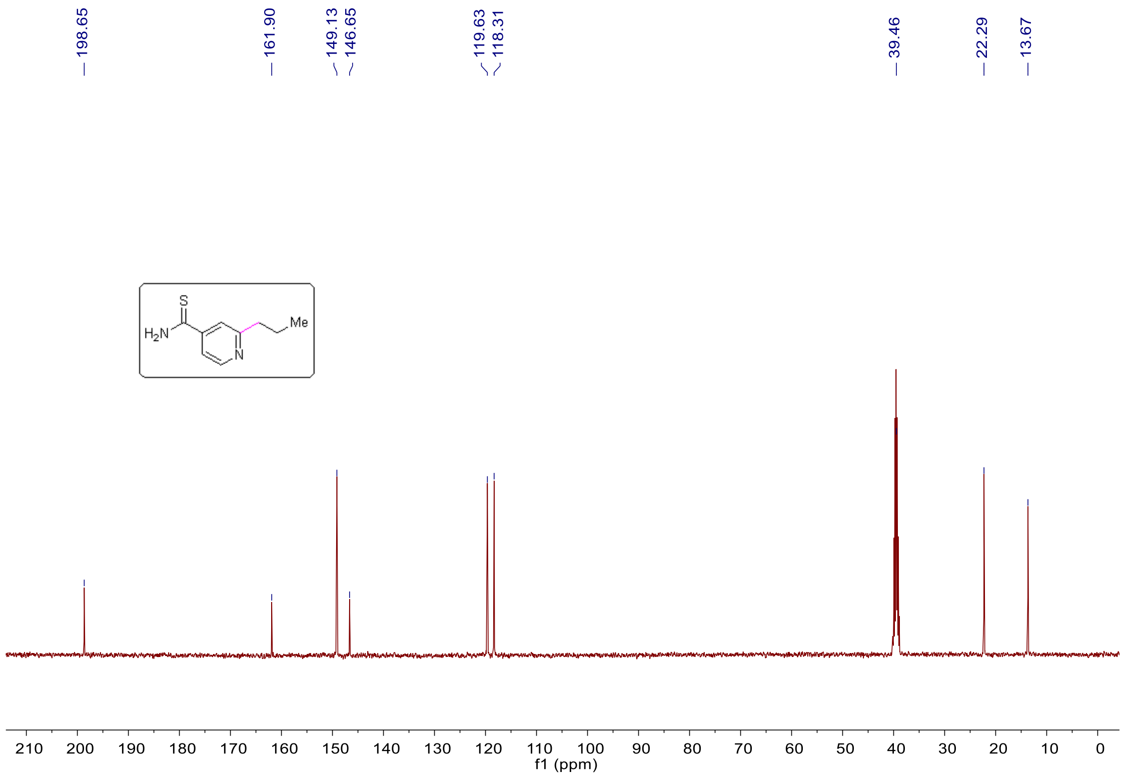

Supplementary Figure 387. ^{13}C NMR (101 MHz, DMSO-d_6) of **15'**

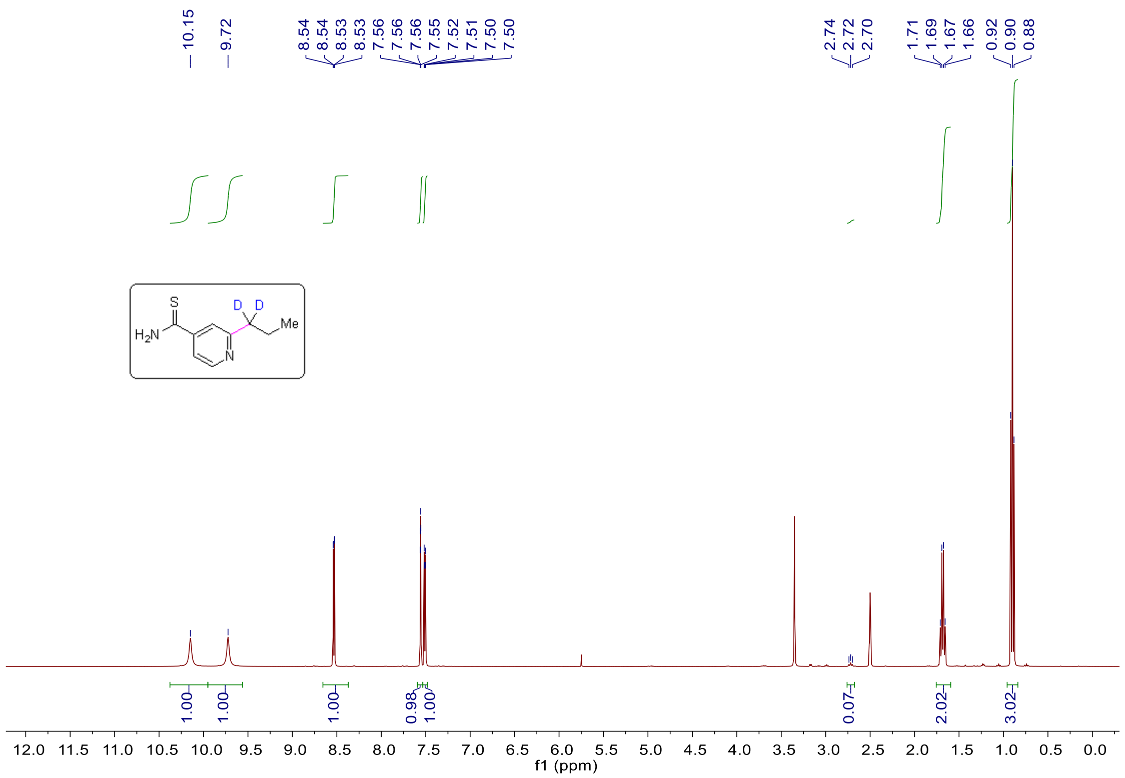

Supplementary Figure 388. ^{1}H NMR (400 MHz, DMSO-d_6) of **15**

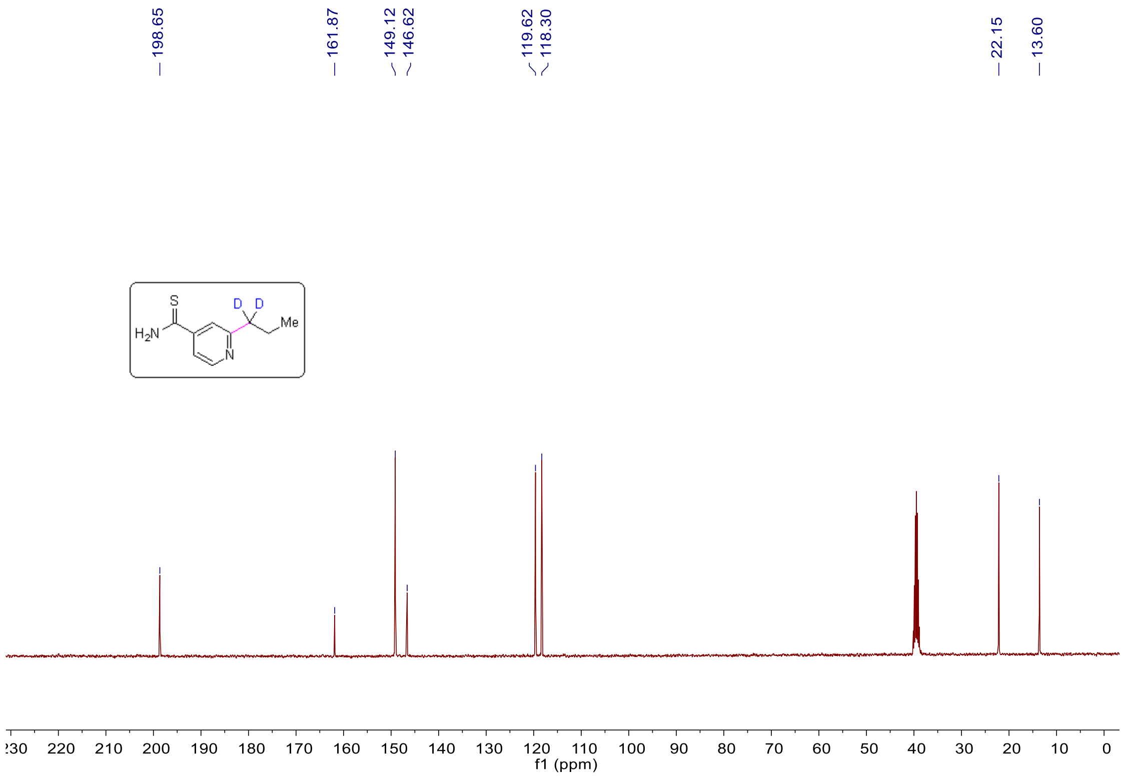

Supplementary Figure 389. ^{13}C NMR (101 MHz, DMSO-d_6) of **15**

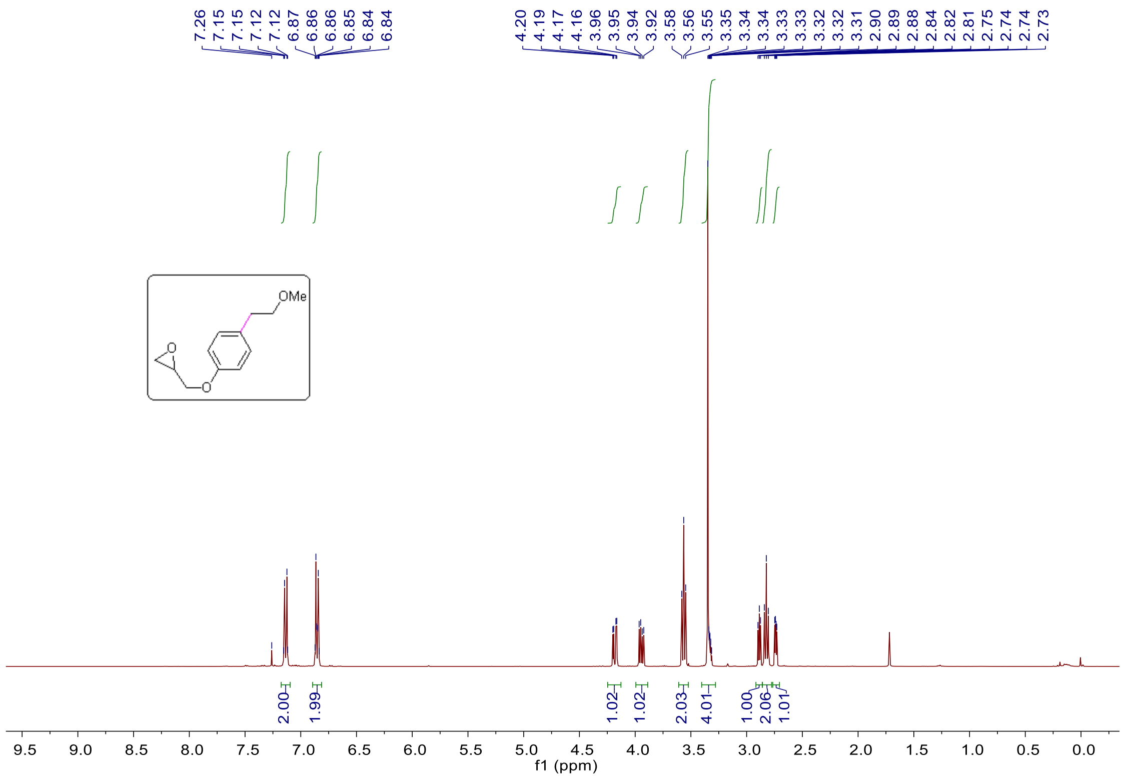

Supplementary Figure 390. ^{1}H NMR (400 MHz, $CDCl_3$) of **17'**

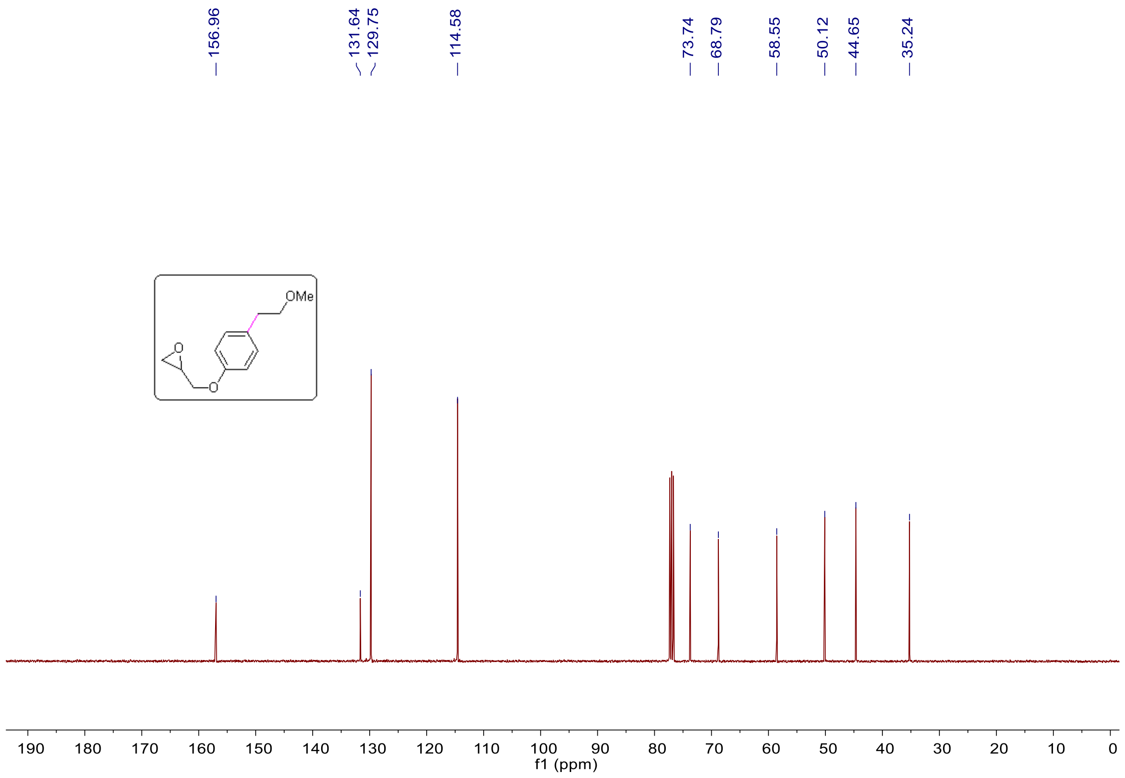

Supplementary Figure 391. ^{13}C NMR (101 MHz, $CDCl_3$) of **17'**

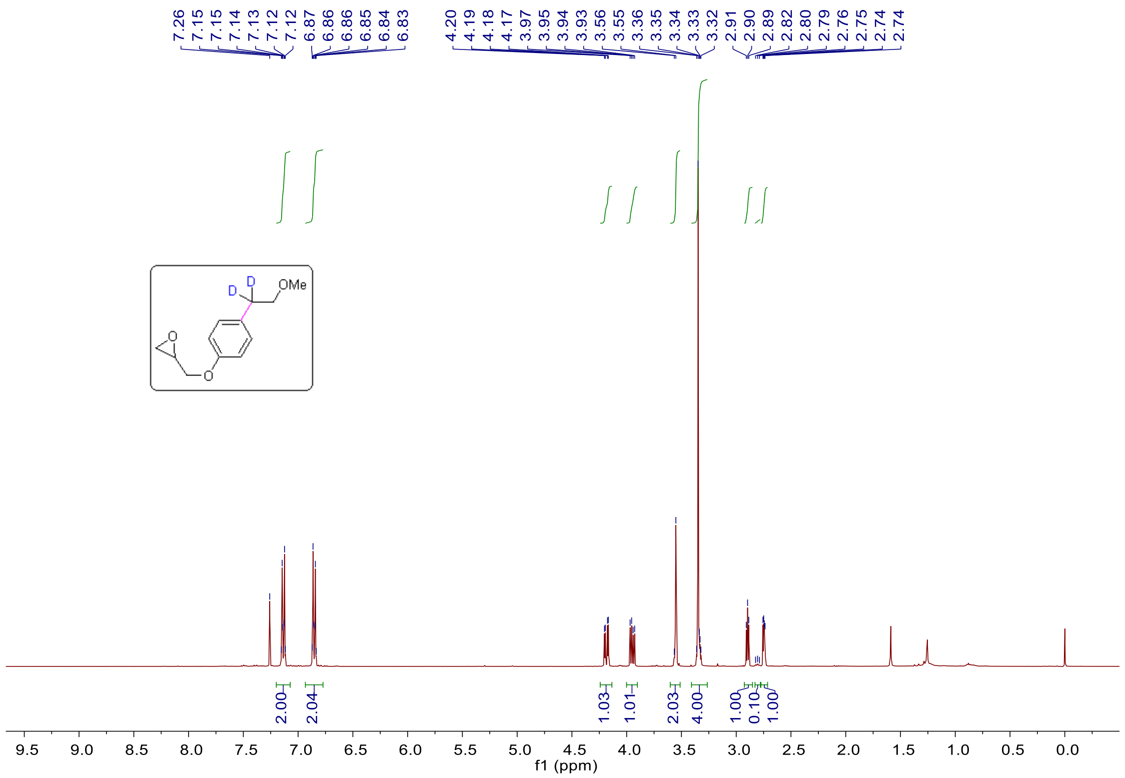

Supplementary Figure 392. ^{1}H NMR (400 MHz, $CDCl_3$) of **17**

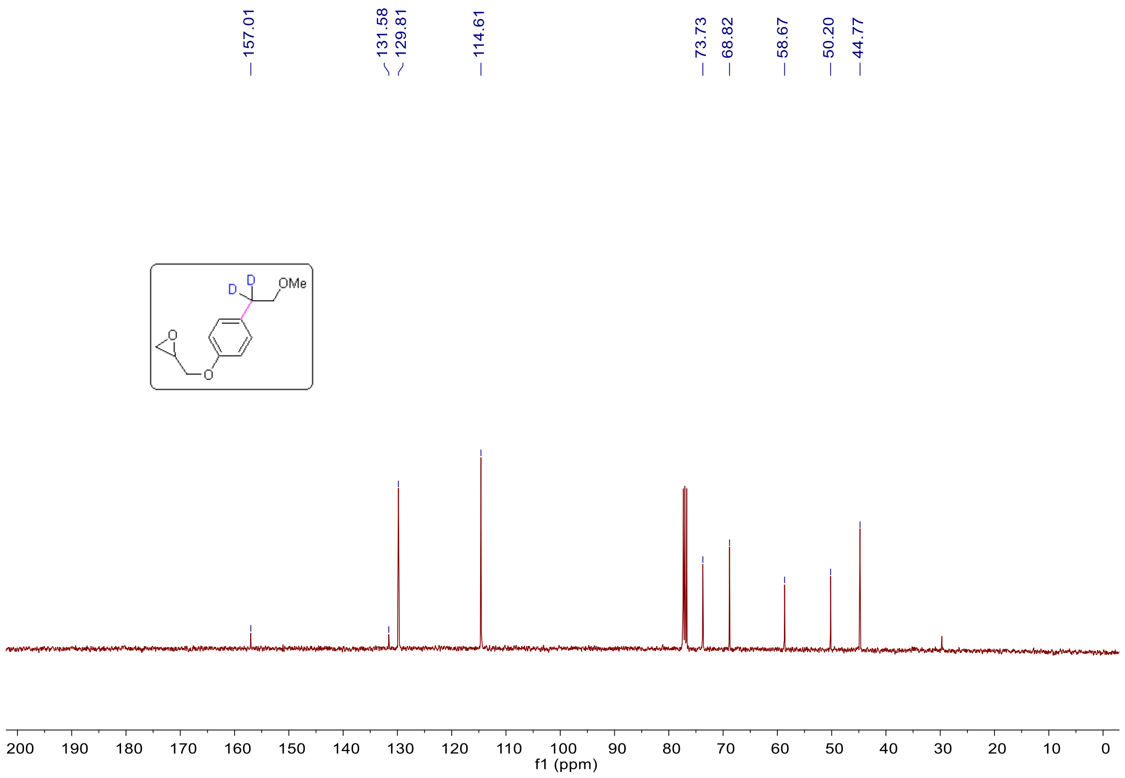

Supplementary Figure 393. ^{13}C NMR (101 MHz, $CDCl_3$) of **17**

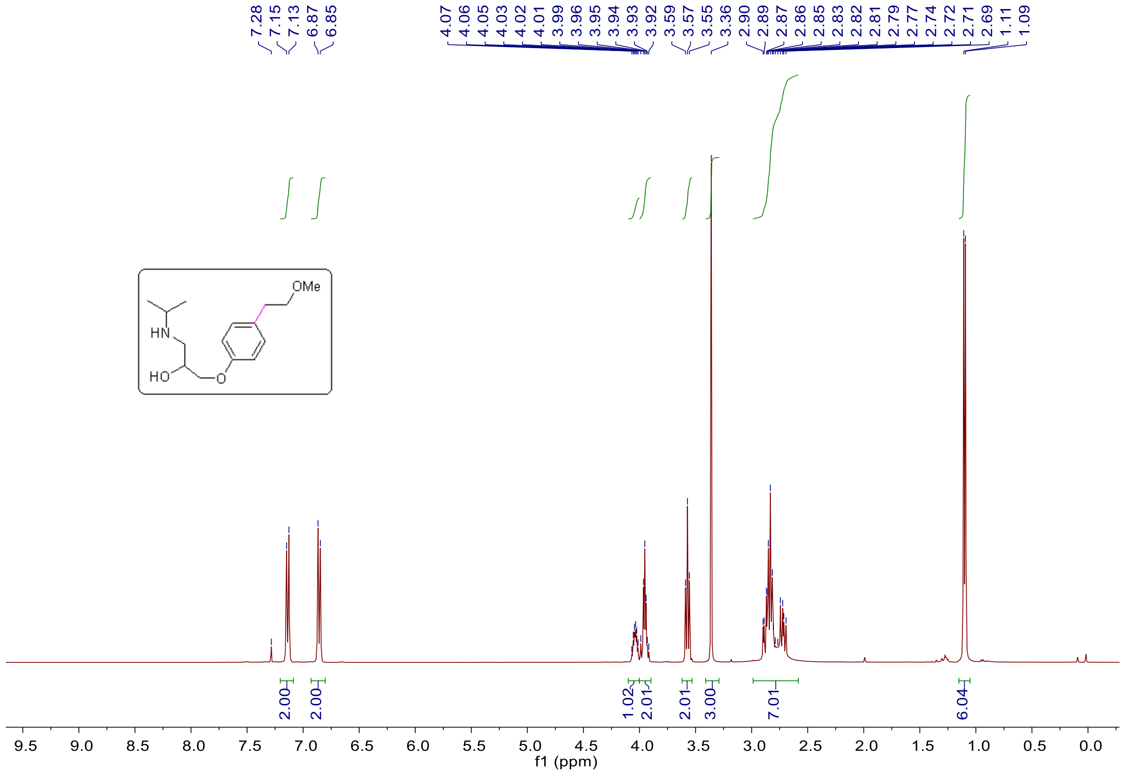

Supplementary Figure 394. ^{1}H NMR (400 MHz, $CDCl_3$) of **18'**

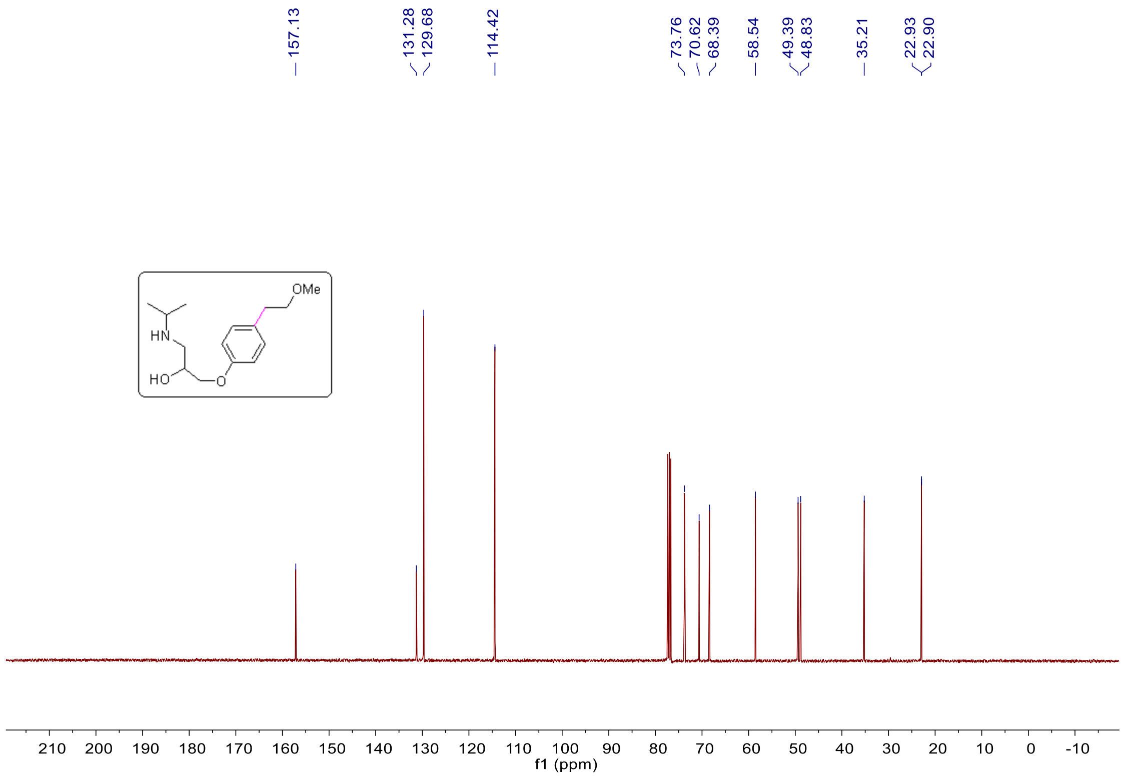

Supplementary Figure 395. ^{13}C NMR (101 MHz, $CDCl_3$) of **18'**

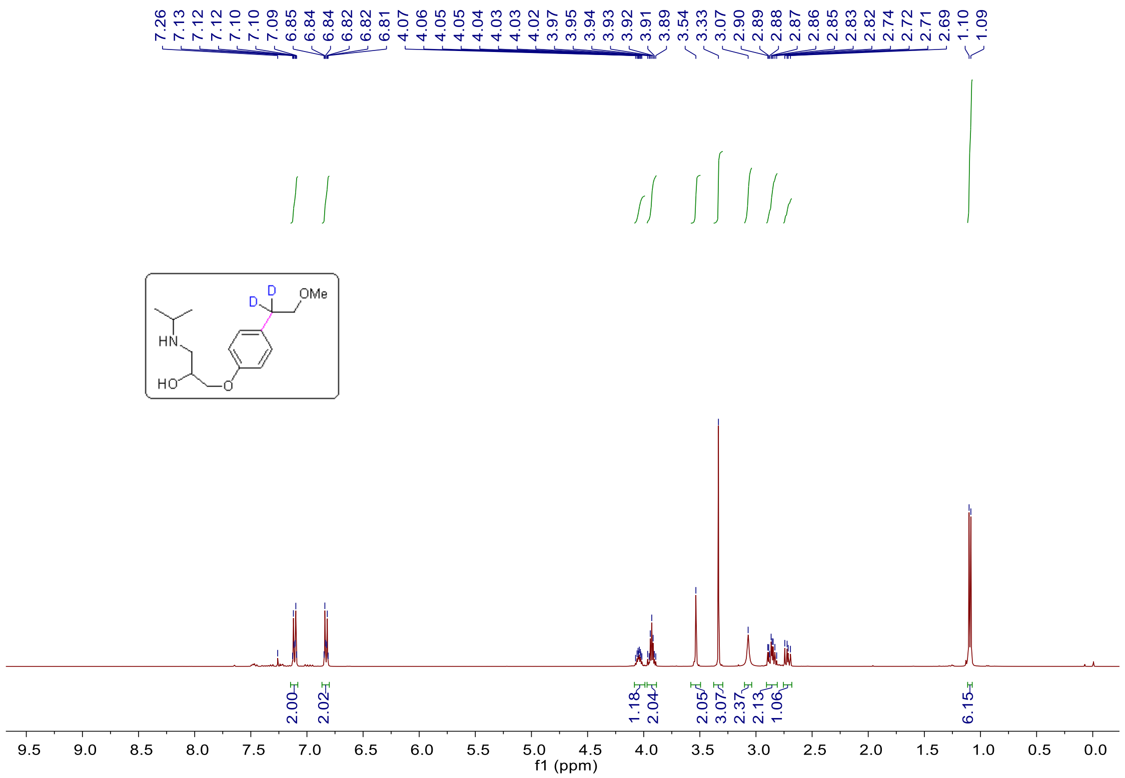

Supplementary Figure 396. ^{1}H NMR (400 MHz, $CDCl_3$) of **18**

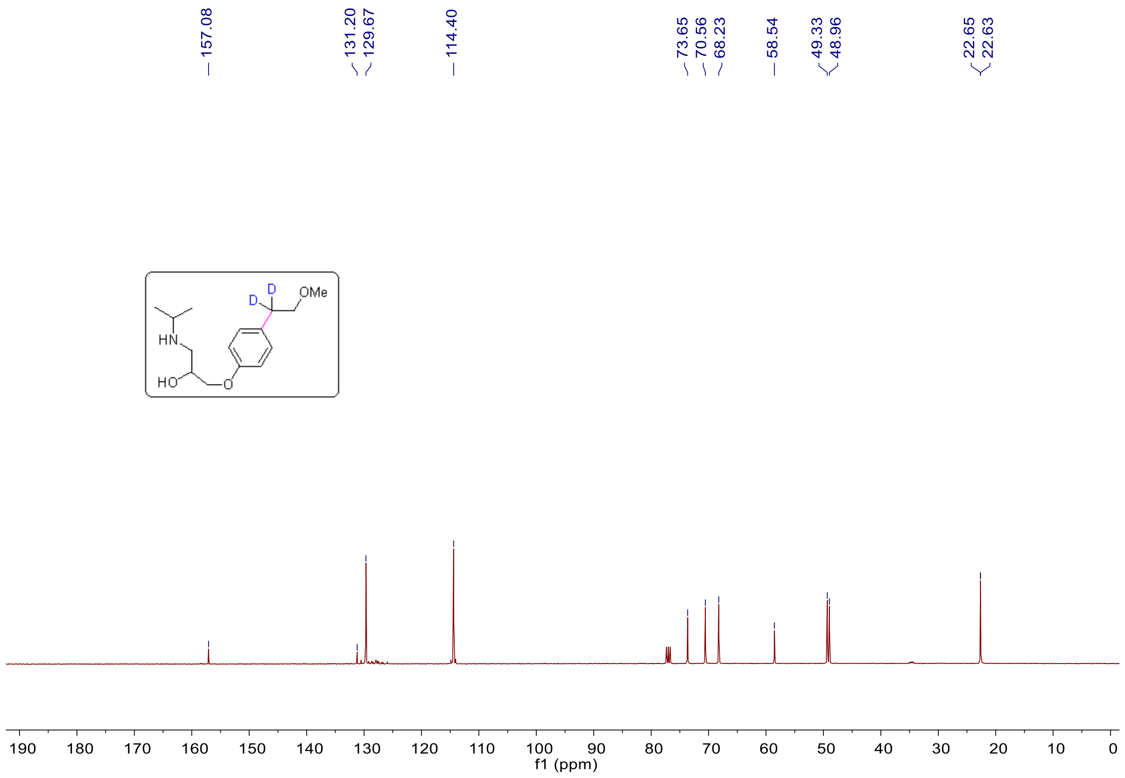

Supplementary Figure 397. ^{13}C NMR (101 MHz, $CDCl_3$) of **18**

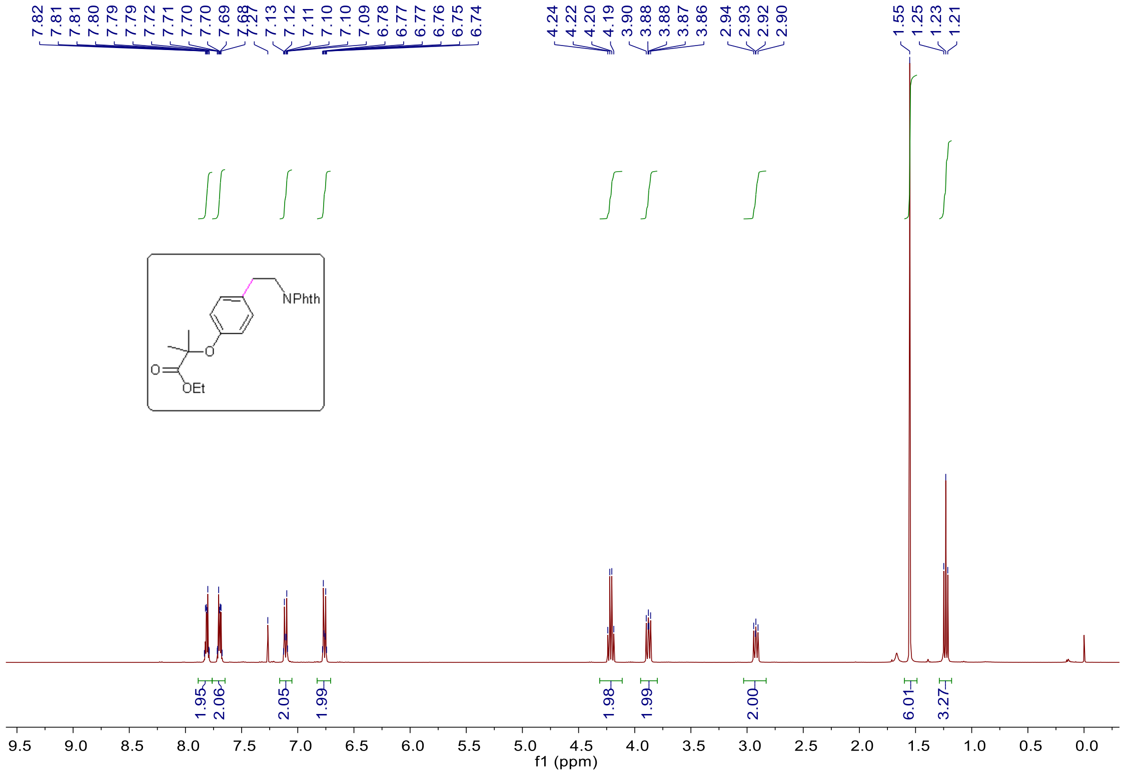

Supplementary Figure 398. ^{1}H NMR (400 MHz, $CDCl_3$) of **5fb'**

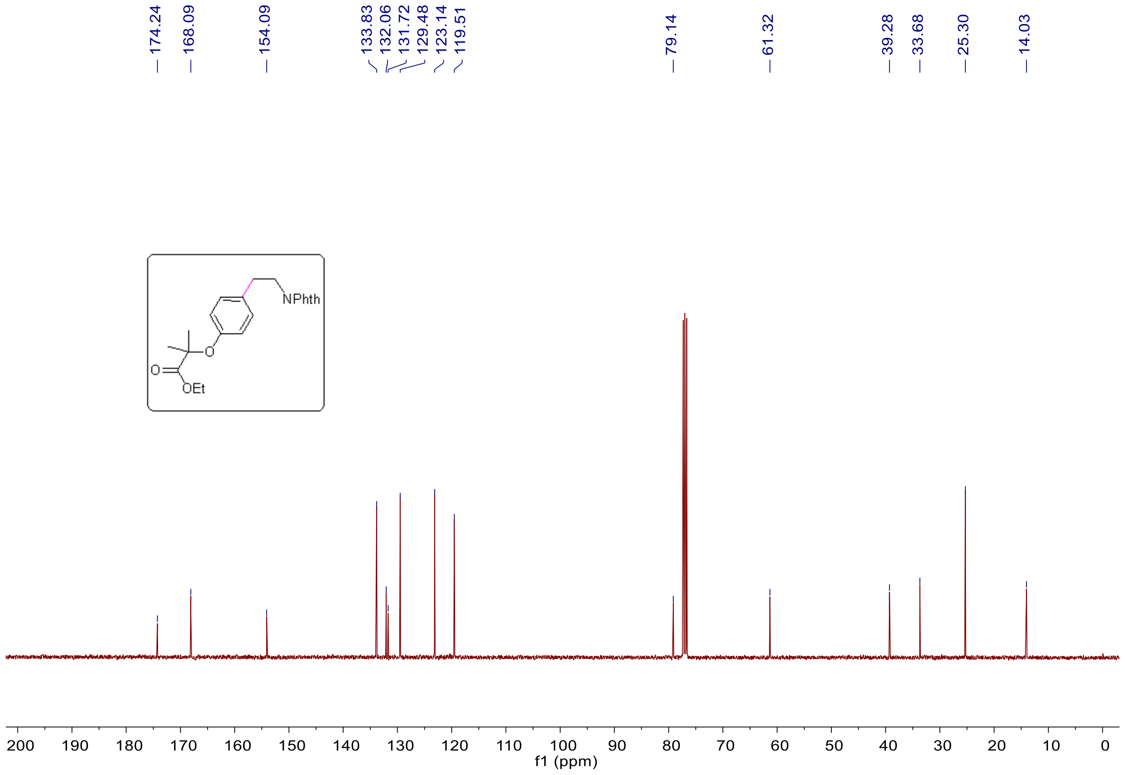

Supplementary Figure 399. ^{13}C NMR (101 MHz, $CDCl_3$) of **5fb'**

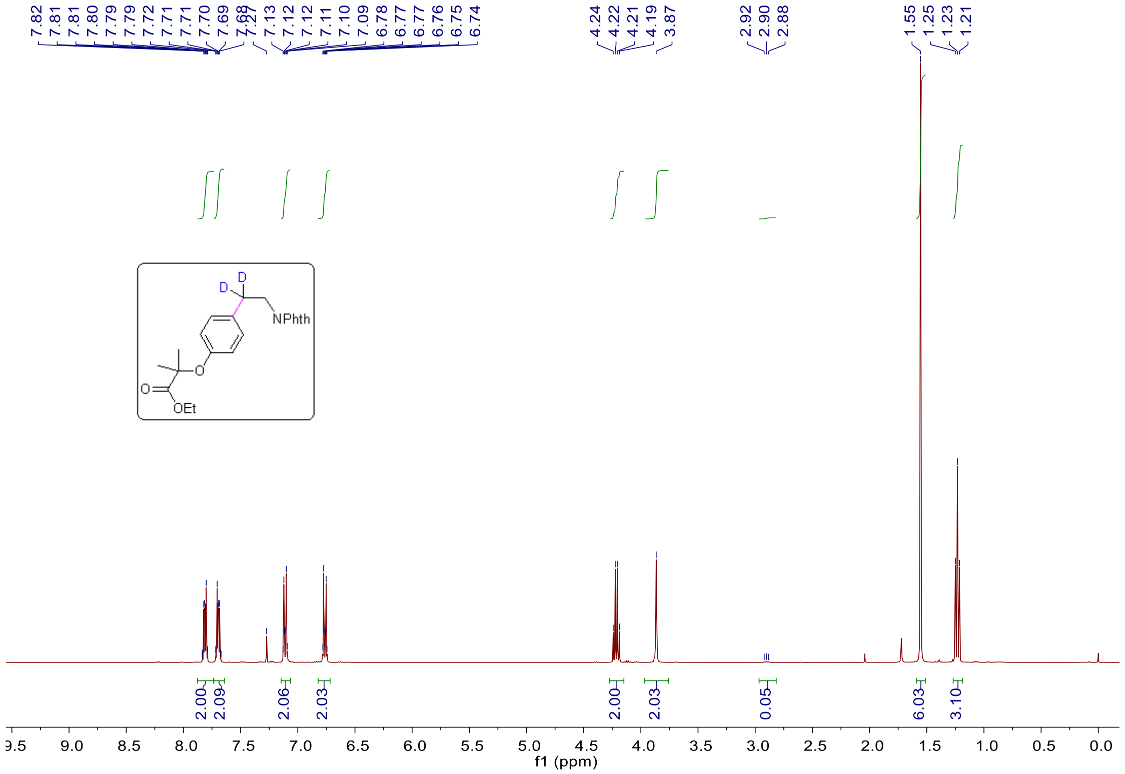

Supplementary Figure 400. ^{1}H NMR (400 MHz, $CDCl_3$) of **5fb**

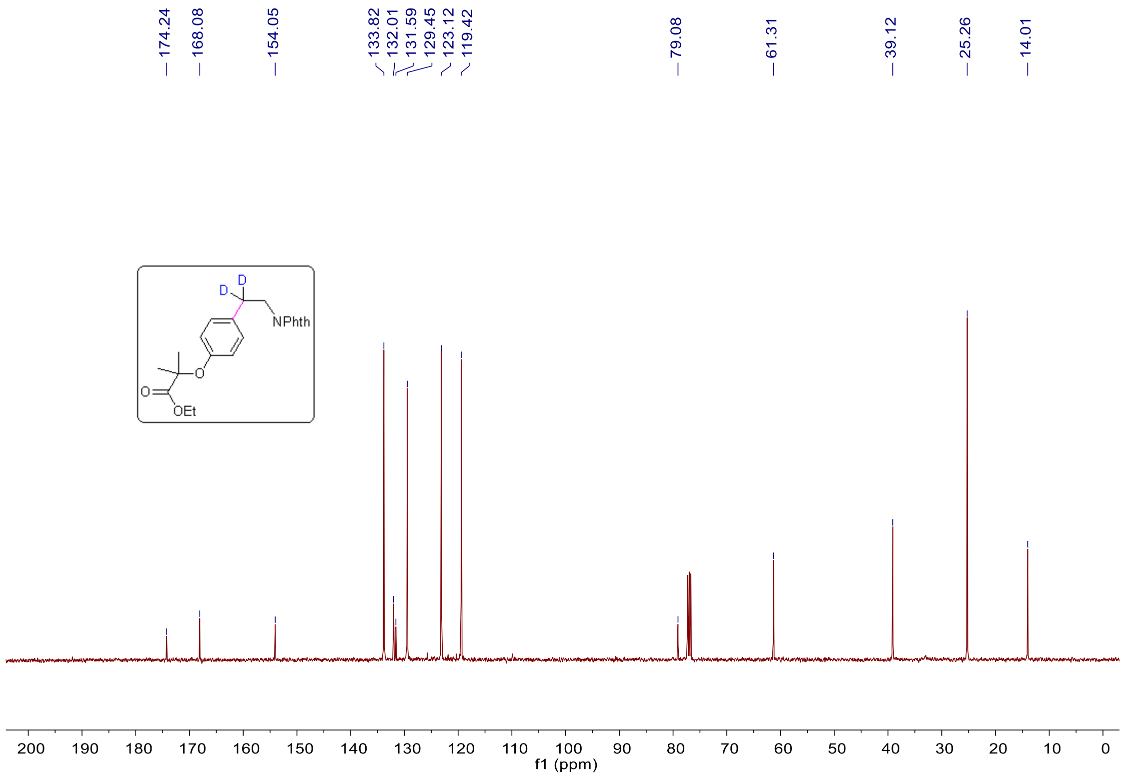

Supplementary Figure 401. ^{13}C NMR (101 MHz, $CDCl_3$) of **5fb**

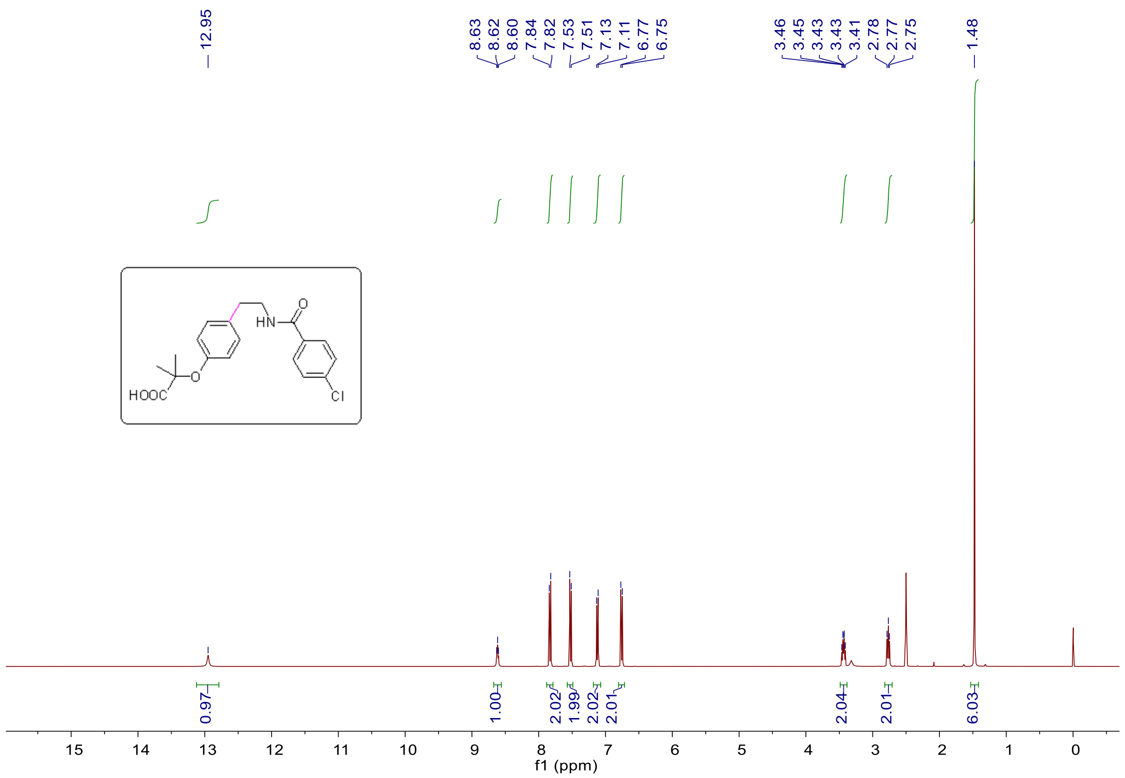

Supplementary Figure 402. ^{1}H NMR (400 MHz, DMSO-d_6) of **19'**

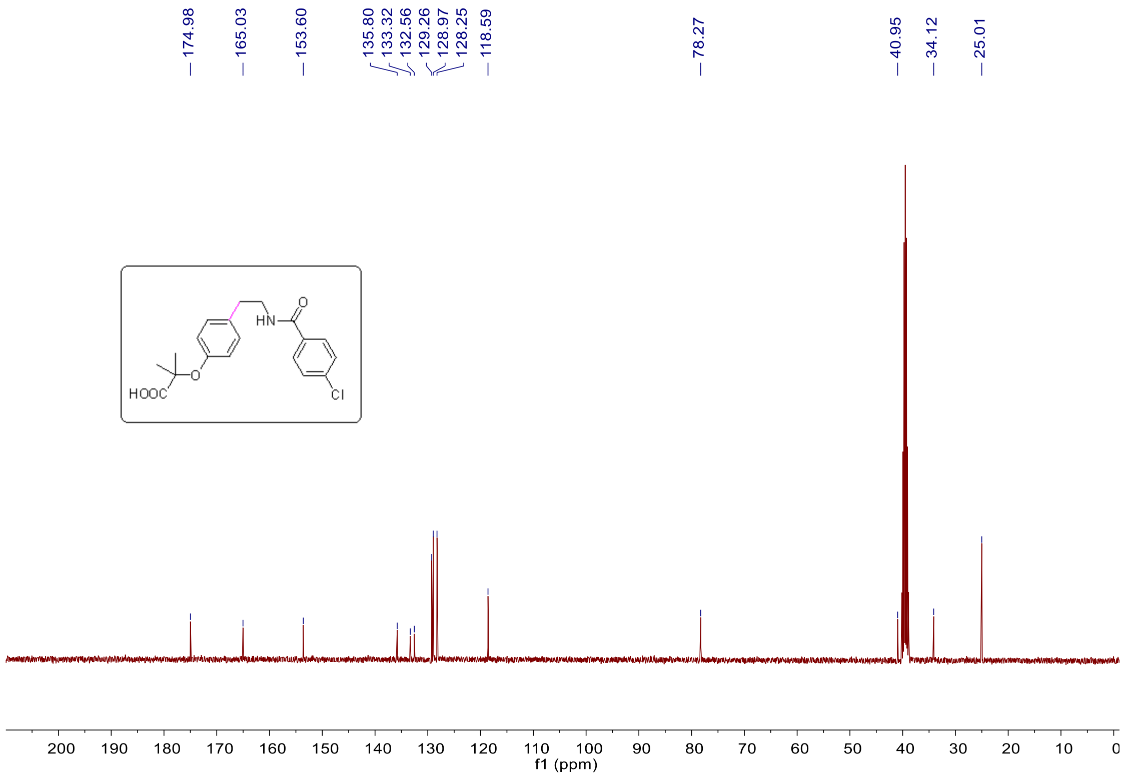

Supplementary Figure 403. ^{13}C NMR (101 MHz, DMSO-d_6) of **19'**

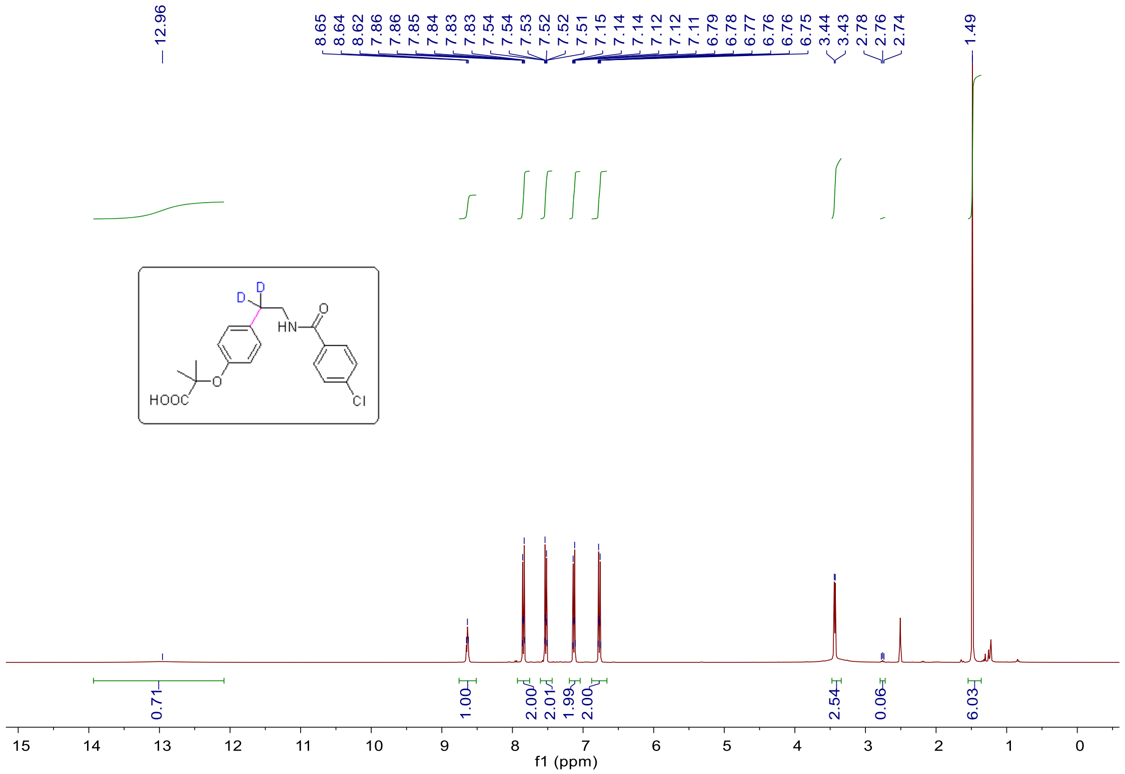

Supplementary Figure 404. ^{1}H NMR (400 MHz, DMSO-d_6) of **19**

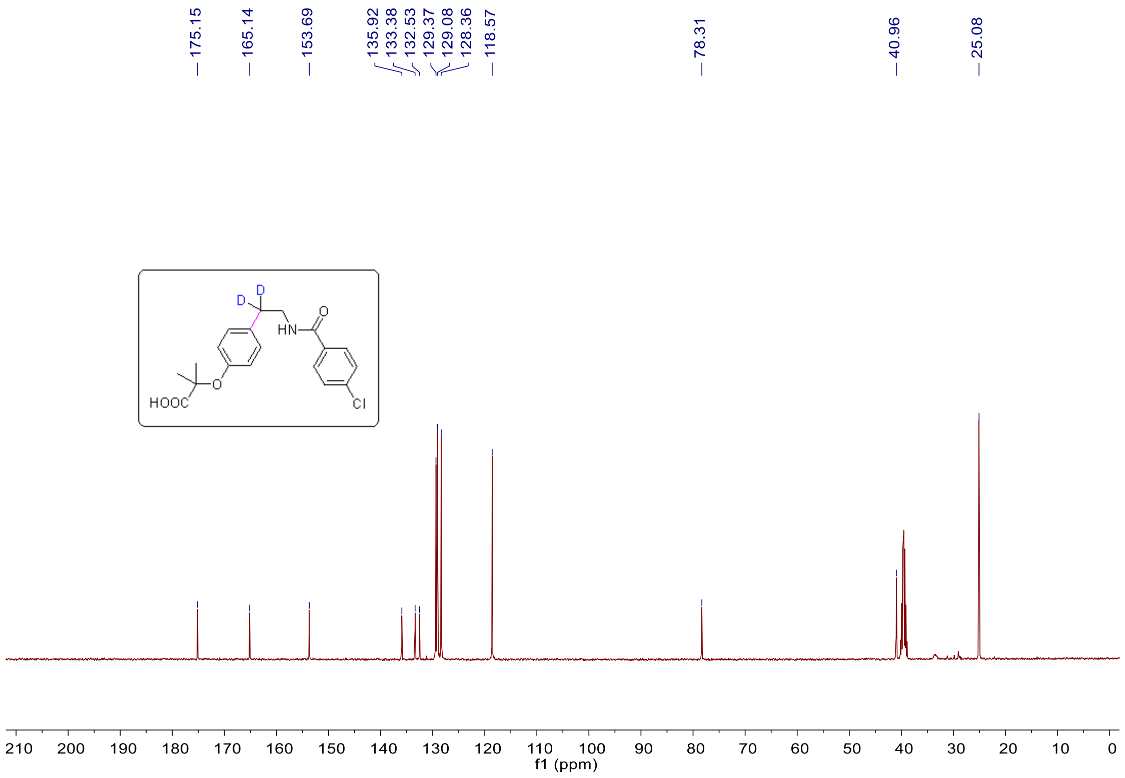

Supplementary Figure 405. ^{13}C NMR (101 MHz, DMSO-d_6) of **19**

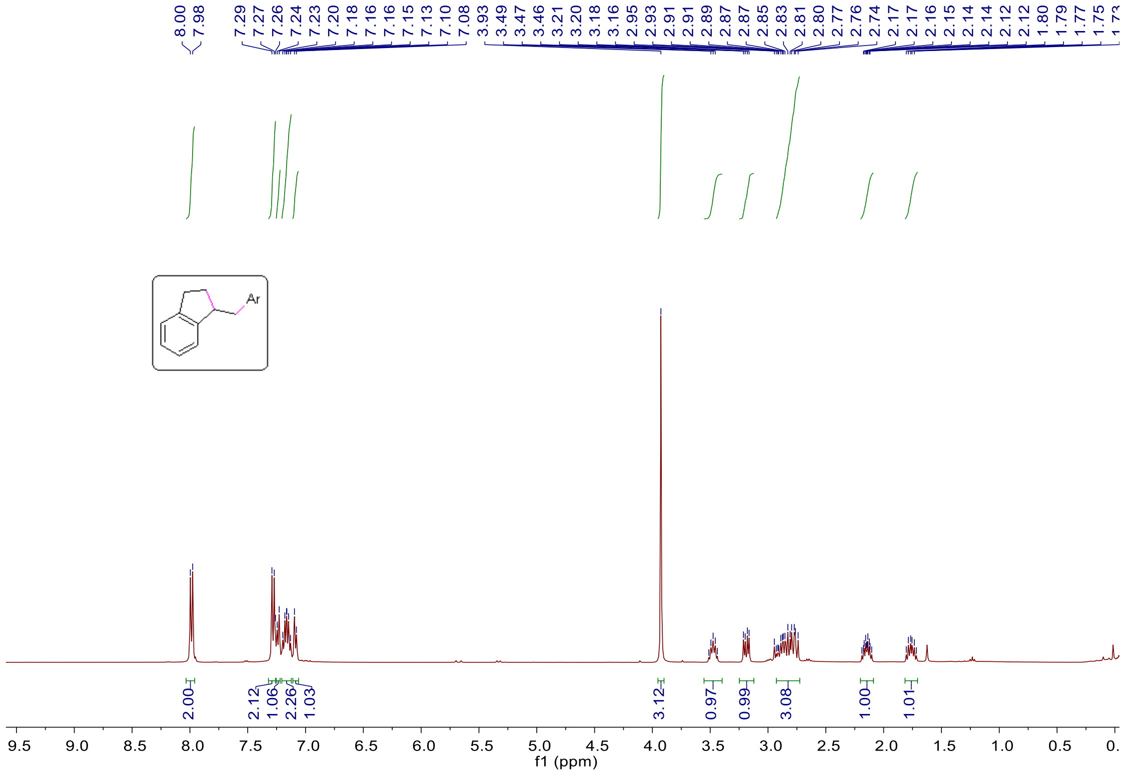

Supplementary Figure 406. ^{1}H NMR (400 MHz, $CDCl_3$) of **26**

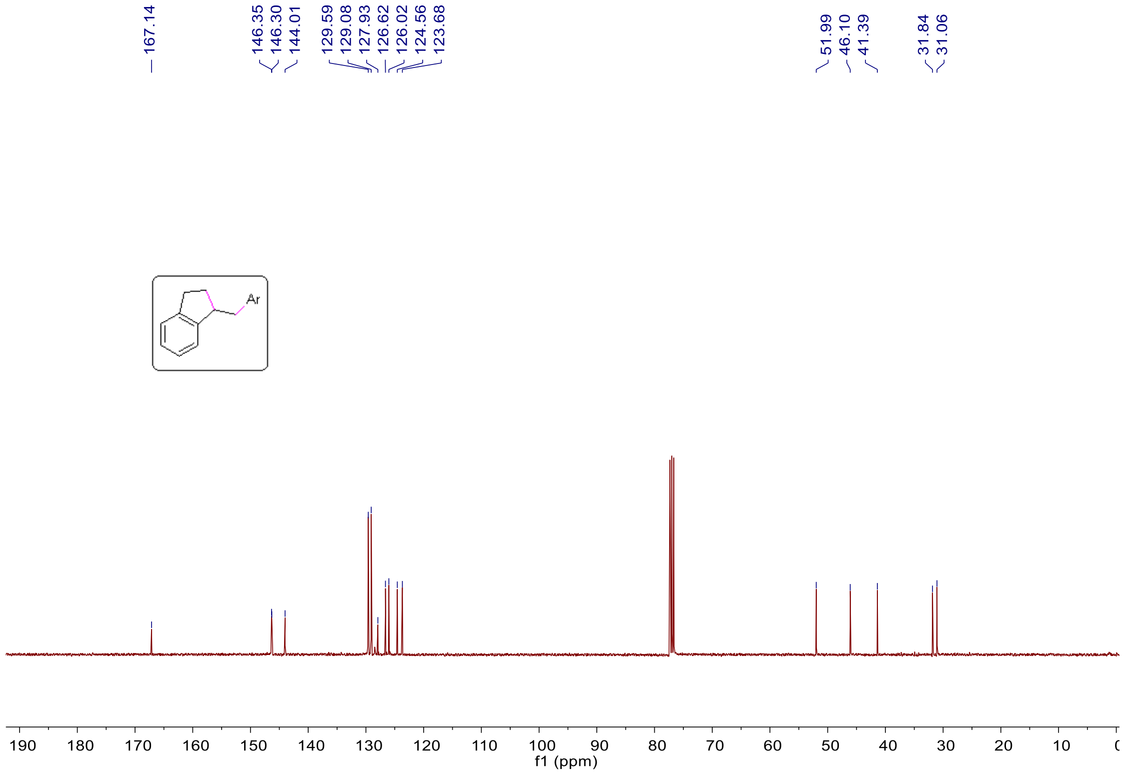

Supplementary Figure 407. ^{13}C NMR (101 MHz, $CDCl_3$) of **26**

Supplementary References

1. (a) Chen, C., Wang, Z.-J., Lu, H., Zhao, Y., Shi, Z. Generation of non-stabilized alkyl radicals from thianthrenium salts for C-B and C-C bond formation. *Nat. Commun.* **12**, 4526 (2021); (b) Chen, C., Wang, M., Lu, H., Zhao, B., Shi, Z., Enabling the use of alkyl thianthrenium salts in cross-coupling reactions by copper catalysis. *Angew. Chem. Int. Ed.* **60**, 21756–21760 (2021).
2. Luridiana, A., Mazzarella, D., Capaldo, L., Rincon, J. A., Garcia-Losada, P., Mateos, C., Frederick, M. O., Nuno, M., Buma, W. J., Noël, T. The Merger of Benzophenone HAT Photocatalysis and Silyl Radical-Induced XAT Enables Both Nickel-Catalyzed Cross-Electrophile Coupling and 1,2-Dicarbofunctionalization of Olefins. *ACS Catal.* **12**, 11216−11225 (2022).
3. Tobisu, M., Nakamura, R., Kita, Y., Chatani, N. Rhodium-Catalyzed Reductive Cleavage of Carbon-Cyano Bonds with Hydrosilane: A Catalytic Protocol for Removal of Cyano Groups. *J. Am. Chem. Soc.* **131**, 3174–3175 (2009).
4. Zhang, X., Yang, C., Alkylations of Arylboronic Acids including Difluoroethylation/Trifluoroethylation *via* Nickel-Catalyzed Suzuki Cross-Coupling Reaction. *Adv. Synth. Catal.* **357**, 2721–2727 (2015).
5. Li, G., Leow, D., Wan, L., Yu, J.-Q. Ether-Directed *ortho*-C–H Olefination with a Palladium(II)/Monoprotected Amino Acid Catalyst. *Angew. Chem. Int. Ed.* **52**, 1245–1247 (2013)
6. Dohle, W., Lindsay D. M., Knochel P. Copper-Mediated Cross-Coupling of Functionalized Arylmagnesium Reagents with Functionalized Alkyl and Benzylic Halides. *Org. Lett.* **3**, 2871–2873 (2001)
7. Lv, X.-Y., Abrams, R., Martin, R. Dihydroquinazolinones as adaptative C(*sp*^{3}) handles in arylations and alkylations via dual catalytic C–C bond-functionalization. *Nat. Commun.* **13**, 2394 (2022).
8. Qin, T., Cornella, J., Li, C., Malins, L. R., Edwards, J. T., Kawamura, S., Maxwell, B. D., Eastgate, M. D., Baran, P. S. A general alkyl-alkyl cross-coupling enabled by redox-active esters and alkylzinc reagents. *Science* **352**, 801–805 (2016).
9. Yu, X., Yang, T., Wang, S., Xu, H., Gong, H. Nickel-Catalyzed Reductive Cross-Coupling of Unactivated Alkyl Halides. *Org. Lett.* **13**, 2138–2141 (2011).
10. Mai, D. N., Baxter, R. D. Unprotected Amino Acids as Stable Radical Precursors for Heterocycle C−H Functionalization. *Org. Lett.* **18**, 3738−3741 (2016).
11. Bellale, E., Naik, M., VB, V., Ambady, A., Narayan, A., Ravishankar, S., Ramachandran, V., Kaur, P., McLaughlin, R., Whiteaker, J., Morayya, S., Guptha, S., Sharma, S., Raichurkar, A., Awasthy, D., Achar, V., Vachaspati, P., Bandodkar, B., Panda, M., Chatterji, M. Diarylthiazole: An Antimycobacterial Scaffold Potentially Targeting PrrB-PrrA Two-Component System. *J. Med. Chem.* **57**, 6572−6582 (2014).
12. Pujala, B., Rana, S., Chakraborti, A. K. Zinc Tetrafluoroborate Hydrate as a Mild Catalyst for Epoxide Ring Opening with Amines: Scope and Limitations of Metal Tetrafluoroborates and Applications in the Synthesis of Antihypertensive Drugs (RS)/(R)/(S)-Metoprolols. *J. Org. Chem.* **76**, 8768–8780 (2011).
13. Nobuta, T., Xiao, G., Ghislieri, D., Gilmore, K., Seeberger, P. H. Continuous and convergent access to vicinyl amino alcohols. *Chem. Commun.* **51**, 15133−15136 (2015).

14. Boyle, M., Livingstone, K., Henry, M. C., Elwood, J. M. L., Lopez-Fernandez, J. D., Jamieson, C. Amide Bond Formation via the Rearrangement of Nitrile Imines Derived from *N*-2-Nitrophenyl Hydrazonyl Bromides. *Org. Lett.* **24**, 334−338 (2022).
15. Gaussian 09, Revision E.01, Frisch, M. J., Trucks, G. W., Schlegel, H. B., Scuseria, G. E., Robb, M. A., Cheeseman, J. R., Scalmani, G., Barone, V., Mennucci, B., Petersson, G. A., Nakatsuji, H., Caricato, M., Li, X., Hratchian, H. P., Izmaylov, A. F., Bloino, J., Zheng, G., Sonnenberg, J. L., Hada, M., Ehara, M., Toyota, K., Fukuda, R., Hasegawa, J., Ishida, M., Nakajima, T., Honda, Y., Kitao, O., Nakai, H., Vreven, T., Montgomery, J. A., Peralta, Jr., J. E., Ogliaro, F., Bearpark, M., Heyd, J. J., Brothers, E., Kudin, K. N., Staroverov, V. N., Keith, T., Kobayashi, R., Normand, J., Raghavachari, K., Rendell, A., Burant, J. C., Iyengar, S. S., Tomasi, J., Cossi, M., Rega, N., Millam, J. M., Klene, M., Knox, J. E., Cross, J. B., Bakken, V., Adamo, C., Jaramillo, J., Gomperts, R., Stratmann, R. E., Yazyev, O., Austin, A. J., Cammi, R., Pomelli, C., Ochterski, J. W., Martin, R. L., Morokuma, K., Zakrzewski, V. G., Voth, G. A., Salvador, P., Dannenberg, J. J., Dapprich, S., Daniels, A. D., Farkas, O., Foresman, J. B., Ortiz, J. V., Cioslowski, J. and Fox, D. J., Gaussian, Inc., Wallingford CT, **2013**.
16. (a) Liptak, M. D., Shields, G. C.*J. Am. Chem. Soc.* **123**, 7314-7319 (2001); (b) Topol, I. A., Tawa, G. J., Caldwell, R. A., Eissenstat, M. A., Burt, S. K. *J. Phys. Chem. A* **104**, 9619 (2000); (c) Magill, A. M., Cavell, K. J., Yates, B. F. *J. Am. Chem. Soc.* **126**, 8717 (2004); (d) Lim, C., Bashford, D., Karplus, M. *J. Phys. Chem.* **95**, 5610 (1991); (e) Jorgensen, W. L., Briggs, J. M., Gao, J. *J. Am. Chem. Soc.* **109**, 6857 (1987).
17. (a) Zhao, Y., Truhlar, D. G. *J. Phys. Chem.* **110**, 5121 (2006); (b) Zhao, Y., Truhlar, D. G. *Theor. Chem. Accounts* **120**, 215 (2008); (c) Hohenstein, E. G., Samuel, S. T., Sherrill, C. D. *J. Chem. Theory. Comput.* **4**, 1996 (2008).
18. (a) Dolg, M., Wedig, U., Stoll, H., Preuss, H. *J. Chem. Phys.* **86**, 866 (1987); (b) Nicklass, A., Dolg, M., Stoll, H., Preuss, H. *J. Chem. Phys.* **102**, 8942 (1995).
19. (a) Krishnan, R., Binkley, J. S., Seeger, R., Pople, J. A. *J. Chem. Phys.* **72**, 650 (1980); (b) McLean, A. D., Chandler, G. S. *J. Chem. Phys.* **72**, 5639 (1980); (c) Francl, M. M., Pietro, W. J., Hehre, W. J., Binkley, J. S., Gordon, M. S., DeFrees, D. J., Pople, J. A. *J. Chem. Phys.* **77**, 3654 (1982); (d) Spitznagel, G. W., Clark, T., Schleyer, P. von R., Hehre, W. J. *J. Comput. Chem.* **8**, 1109 (1987).
20. Marenich, A. V., Cramer, C. J., Truhlar, D. G. *J. Phys. Chem. B*. **113**, 6378 (2009).
21. (a) Camaioni, D. M., Schwerdtfeger, C. A. *J. Phys. Chem. A* **109**, 10795 (2005); (b) Zhan, C.-G., Dixon, D. A. *J. Phys. Chem. A* **105**, 11534 (2001).
22. Blanksby, S. J., Ellison, G. B. *Acc. Chem. Res.* **36**, 255 (2003).
23. (a) Becke, A. D. *J. Chem. Phys.* **98**, 5648 (1993); (b) Lee, C., Yang, W., Parr, R. G. *Phys. Rev. B* **37**, 785 (1988).
24. (a) Ditchfield, R., Hehre, W. J., Pople, J. A. *J. Chem. Phys.* **54**, 724 (1971); (b) Hehre, W. J., Ditchfield, R., Pople, J. A.*J. Chem. Phys.* **56**, 2257 (1972); (c) Hariharan, P. C., Pople, J. A. *Theor. Chem. Acc.* **28**, 213 (1973).
25. Legault, C. Y. CYL View, version 1.0 b; Universite de Sherbrooke, Sherbrooke, Quebec, Canada, **2009**; http://www.cylview.org.
