# Supplementary material for: Metallaphotoredox deuteroalkylation utilizing thianthrenium salts
Source: Nat Commun. 2024 Jun 13;15:5067. doi: 10.1038/s41467-024-48590-w (PMC11176366; doi:10.1038/s41467-024-48590-w)
Supplement: Supplementary file 2 — Peer Review File [file 41467_2024_48590_MOESM2_ESM.pdf]

nature portfolio

Peer Review File

Metallaphotoredox deuteroalkylation utilizing thianthrenium salts

Open Access This file is licensed under a Creative Commons Attribution 4.0 International License, which permits use, sharing, adaptation, distribution and reproduction in any medium or format, as long as you give appropriate credit to the original author(s) and the source, provide a link to the Creative Commons license, and indicate if changes were made. In the cases where the authors are anonymous, such as is the case for the reports of anonymous peer reviewers, author attribution should be to 'Anonymous Referee' followed by a clear attribution to the source work. The images or other third party material in this file are included in the article's Creative Commons license, unless indicated otherwise in a credit line to the material. If material is not included in the article's Creative Commons license and your intended use is not permitted by statutory regulation or exceeds the permitted use, you will need to obtain permission directly from the copyright holder. To view a copy of this license, visit http://creativecommons.org/licenses/by/4.0/.

Editorial Note: This manuscript has been previously reviewed at another journal that is not operating a transparent peer review scheme. This document only contains reviewer comments and rebuttal letters for versions considered at *Nature Communications.*

REVIEWERS' COMMENTS

Reviewer #1 (Remarks to the Author):

The authors addressed most of the points of the reviewers. Especially the detailed discrimination of their work with already described TT methods is valuable.

With the made changes and the publication of the author response as supporting info I recommend publication in Nature Communication.

Reviewer #2 (Remarks to the Author):

Months ago, we have been invited as a referee to review this work reported by Shi et al for its publication on Nat. Catal. Then, for the previous version of the manuscript, we raised our concerns and comments regarding to its novelty and mechanistic studies. We are very pleased to say that the manuscript has been greatly improved, and more importantly, the author detailed the insight of the reaction process and demonstrated its promising utilizations in organic synthesis. So, the referee recommends its publication on NatCommun NOW.

Here are a few suggestions for the author’s consideration: 1) Please denote the linked bond of TT to aryl substrates in Fig.1C; 2) Please present your comments on the absence of self-coupling products of TTs in Fig 5b.

Comments from Referee #1

Regarding the comment: "The authors addressed most of the points of the reviewers. Especially the detailed discrimination of their work with already described TT methods is valuable. With the made changes and the publication of the author response as supporting info I recommend publication in Nature Communication."

Response: Thanks for such positive comments.

Comments from Referee #2

Regarding the comment: "Months ago, we have been invited as a referee to review this work reported by Shi et al for its publication on Nat. Catal. Then, for the previous version of the manuscript, we raised our concerns and comments regarding to its novelty and mechanistic studies. We are very pleased to say that the manuscript has been greatly improved, and more importantly, the author detailed the insight of the reaction process and demonstrated its promising utilizations in organic synthesis. So, the referee recommends its publication on Nat Commun NOW."

Response: Thanks for such positive comments.

Regarding the comment: "1) Please denote the linked bond of TT to aryl substrates in Fig.1C"

Response: The linked bond of TT to aryl substrates in Fig.1C has been denoted.

Regarding the comment: "2) Please present your comments on the absence of self-coupling products of TTs in Fig 5b."

Response: We added the related comments in the text.
